# Cyclic Peptide PROTACs Restore VHL-Mediated HIF-1α Degradation in Hypoxia

**DOI:** 10.1021/jacs.6c08218

**Published:** 2026-09-14

**Authors:** Alexander McDermott, Reece M. Gardner, Monika Papayova, Agnieszka B. Wisniewska, Cyrielle Doigneaux, Soran Mohammed, Ali Tavassoli

**Affiliations:** School of Chemistry and Chemical Engineering, 7423 University of Southampton, Southampton SO17 1BJ, U.K.

## Abstract

We report a systematic approach to convert cyclic peptide protein–protein interaction (PPI) inhibitors into functional degraders. We compare traditional PEG-based linkers with custom bifunctional amino acids that embed a Von Hippel-Lindau (VHL)-recruiting ligand and variable-length linkers for direct incorporation into cyclic peptides during solid-phase peptide synthesis (SPPS). Using an optimized split-intein circular ligation of peptides and proteins (SICLOPPS)-derived hypoxia-inducible factor 1 (HIF-1) inhibitor as the warhead, we generate cyclic peptide PROTACs (CP-PROTACs) that form a ternary complex with VHL and degrade HIF-1α across multiple cancer cell lines, suppressing hypoxia-response signaling. Degradation is oxygen-state dependent, being robust at 3% oxygen but lost at 1% oxygen. We show that the degrader remains engagement- and degradation-competent at 1% oxygen but is outpaced by HIF-1α resynthesis, revealing a kinetic ceiling on proteolytic-targeting chimeras (PROTAC) efficacy against rapidly replenished proteins. The modular amino acid building blocks reported provide a generalizable blueprint for converting cyclic peptides into degraders, extending targeted protein degradation to previously intractable proteins.

## Graphical abstract

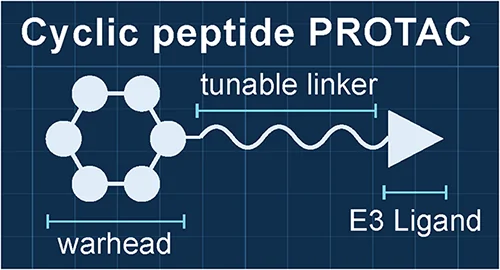

## Introduction

Proteolytic-targeting chimeras (PROTACs) offer an alternative approach to targeted therapy by circumventing the occupancy-driven pharmacology of traditional inhibitors.^1,2^ By harnessing the ubiquitin-proteasome system (UPS), PROTACs can deplete proteins through a catalytic, substoichiometric mechanism. Most reported PROTACs rely on small-molecule warheads, limiting applicability to the subset of proteins with druggable small-molecule binding pockets. Cyclic peptides occupy a unique chemical space between small molecules and biologics, offering high specificity and affinity for challenging protein targets.^3–5^ Our efforts have focused on the development and utilization of a method for the production of a library of millions of cyclic peptides named split-intein circular ligation of peptides and proteins (SICLOPPS) that uses split-inteins to generate head-to-tail cyclic peptide libraries in cells.^4^ SICLOPPS libraries have been combined with a variety of cell-based high-throughput screening assays to enable the identification of cyclic peptide modulators of a variety of protein–protein interactions (PPI).^6–8^ Cyclic peptide PROTACs (CP-PROTACs) have the potential to expand targeted protein degradation to proteins not accessible to conventional small-molecule ligands.^9,10^

Hypoxia-inducible factor 1 (HIF-1) orchestrates cellular adaptation to hypoxic microenvironments in solid tumors by regulating transcription of hundreds of genes involved in metabolism, proliferation, angiogenesis, and metastasis.^11^ In normoxia, HIF-1α has a half-life of less than 5 min; prolyl hydroxylation of P402 and P564 creates a binding motif for the Von Hippel-Lindau (VHL) tumor suppressor protein, which recruits Elongins B/C, Cullin 2, and Rbx1 to form an E3 ligase complex that polyubiquitinates HIF-1α and targets it to the 26S proteasome.^12,13^ Because HIF-1 signaling promotes aggressive cancer phenotypes that are resistant to therapeutic intervention,^14,15^ HIF-1 has been a challenging and longstanding drug discovery target.^16,17^ We have previously used SICLOPPS screening to identify an isoform-selective inhibitor of the HIF-1α/HIF-1β protein–protein interaction (PPI) and a dual inhibitor of the interaction of HIF-1α and HIF-2α with HIF-1β.^18,19^ Here, we report CP-PROTACs that restore VHL-mediated proteasomal degradation of HIF-1α in hypoxia. These compounds prevent HIF-1-mediated hypoxia-response signaling in cancer cells. The modular amino acid building blocks and synthetic workflow are directly applicable to diverse cyclic peptide scaffolds, enabling a route for the development of a wide range of CP-PROTACs.

## Results and Discussion

### Optimization of *cyclo*-CLLFVY by SAR Studies

We selected our previously reported HIF-1α/HIF-1β PPI inhibitor *cyclo*-CLLFVY (**1**) as the PROTAC warhead. We synthesized *cyclo*-CLLFVY and measured its binding affinity for the HIF-1α PAS-B domain by MST, observing a *K*_D_ of 35 ± 7 μM (Figure 1a), consistent with previous reports.^20^ To improve affinity, we systematically substituted residues in the parent cyclic peptide with natural and unnatural analogues. We first focused on the cysteine in position 1, preparing seven analogues (Figure 1b). Most substitutions abolished detectable binding to HIF-1α; only analogues retaining a free thiol showed measurable affinity (**2** and **7**), indicating that the thiol is critical for interaction and that the position of the sulfur relative to the α-carbon strongly influences binding (Figure 1b). We therefore retained the cysteine residue at position 1. We next optimized the phenylalanine at position 4; eighteen derivatives containing phenylalanine analogues in position 4 were synthesized (**9-26**) and evaluated for their affinity to the PAS-B domain of HIF-1α (Figure 1c). A preference for bulky hydrophobic substituents, e.g., NaI (**11**), (*p*-Ph)F (**24**), and (*p*-*t*Bu)F (**25**) was observed. The (*p*-CF_3_)­F-containing analogue (**22**) with a *K*_D_ of 1.8 ± 0.7 μM represented a ∼20-fold improvement over **1**, and this residue was retained in position 4 in subsequent analogues. We then replaced tyrosine at position 6 with non-natural amino acids (**27-40**) (Figure 1d). Affinity varied only modestly across this series, indicating that position 6 tolerates diverse substituents without substantially perturbing HIF-1α binding. Notably, polar side chains, e.g., (*p*-NO_2_)F (**40**), were tolerated, whereas analogous substitutions at position 4 were detrimental to binding, e.g., (**26**), consistent with enrichment of tyrosine over phenylalanine at this site in our original SICLOPPS screen.^18^ The highest affinity derivative in this series was (*p*-Br)F (**35**) with a *K*_D_ of 1.4 ± 0.4 μM, which was retained in position 6.

**Figure 1:**
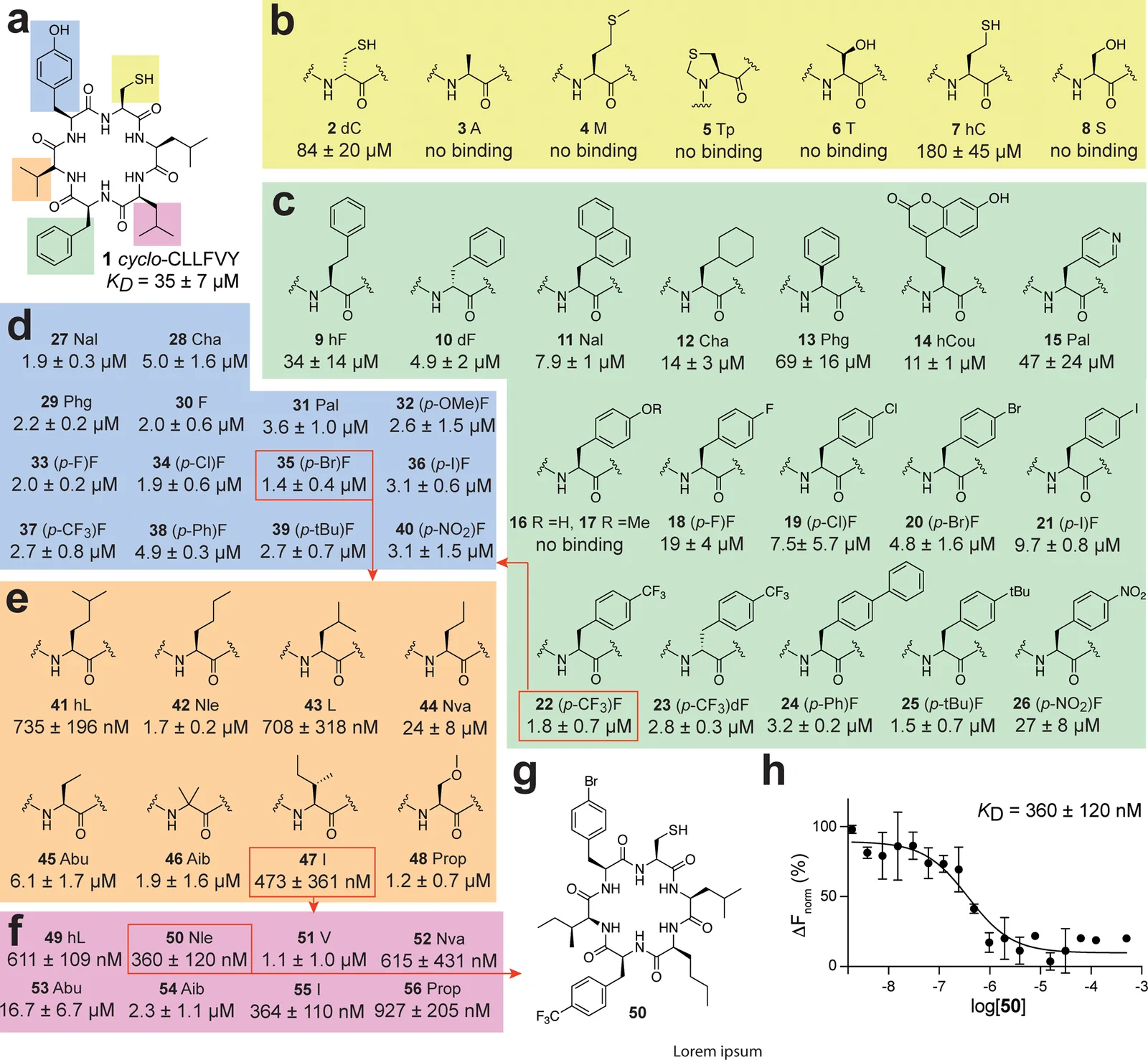
Optimizing the activity of *cyclo*-CLLFVY. (a) Structure and affinity of *cyclo*-CLLFVY. (b) Cysteine analogues incorporated into position 1 and their affinity. (c) Phenylalanine analogues incorporated into position 4 and their affinity. (d) Phenylalanine analogues incorporated into position 6 and their affinity. (e) Aliphatic amino acids incorporated into position 5 and their affinity. (f) Aliphatic amino acids incorporated into position 3 and their affinity. (g) The structure of *cyclo*-CLNle­(*p*-CF_3_)­FI­(*p*-Br)­F. (h) Binding affinity of *cyclo*-CLNle­(*p*-CF_3_)­FI­(*p*-Br)F to HIF-1α by MST. Binding curves for the above data are in Figures S1–S5; data are mean (*n* = 3) ± SD.

We next investigated the effect of altering valine at position 5. A panel of eight substituents containing aliphatic amino acids (**41-48**) was prepared and tested (Figure 1e). Short, linear side chains reduced binding, e.g., Nve (**44**) and Abu (**45**), whereas branched side chains improved affinity, e.g., hL (**41**), L (**43**), and I (**47**). Isoleucine (**47**) gave the strongest binding with a *K*_D_ of 473 ± 361 nM and was therefore selected for retention in this position. The same aliphatic substituents were then introduced at position 3 of the analogues (**49-56**). Substitutions at this site had minimal impact on affinity, other than Abu (**53**), which caused a 35-fold loss in affinity, likely due to an effect on the conformation of the cyclic peptide (Figure 1f). This data suggests that position 3 is solvent-exposed and contributes by maintaining peptide conformation rather than directly engaging HIF-1α, making it a suitable site for linker incorporation. The Nle derivative (**50**) gave the best affinity in this set, but was not significantly different from the I-containing analogue (**55**). After optimization, the most potent molecule was *cyclo*-CLNle­(*p*-CF_3_)­FI­(*p*-Br)F (**50**) (Figure 1g) with a *K*_D_ of 360 ± 120 nM by MST (Figure 1h).

### Evaluating the *In Vitro* and Cell-Based Activity of the Optimized HIF-1 Inhibitors

To assess functional activity, we developed a fluorescence polarization (FP) assay that reports binding of the HIF-1α/HIF-1β heterodimer (bHLH/PAS-A/PAS-B domains) to a fluorophore-labeled hypoxia-response element (HRE). Disruption of the HIF-1α/HIF-1β PPI reduced HRE binding and produced a measurable FP change. The optimized cyclic peptide *cyclo*-CLNle­(*p*-CF_3_)­FI­(*p*-Br)­F (**50**) inhibited HIF-1-HRE binding with an IC_50_ of 7.9 ± 0.6 μM (Figure 2a). To test whether MST activity correlated to functional inhibition, we also evaluated the parent peptide *cyclo*-CLLFVY (**1**) and an inactive analogue *cyclo*-ALLFVY (**3**). We measured an IC_50_ of 76 ± 8 μM for *cyclo*-CLLFVY (Figure S6a) while *cyclo*-ALLFVY was inactive (Figure S6b), supporting a relationship between target binding affinity and functional PPI inhibition. To directly measure disruption of the HIF-1α/HIF-1β PPI, we developed an HIF-1 ELISA using isolated PAS-B domains. Because the limited aqueous solubility of **50** prevented its use in this format, we generated a more soluble analogue by substituting leucine in position 2 with lysine. The resulting peptide, *cyclo*-CKNle­(*p*-CF_3_)­FI­(*p*-Br)F (**57**), bound HIF-1α with a *K*_D_ of 1.60 ± 0.3 μM by MST (Figure 2b) and inhibited the HIF-1α/HIF-1β PPI by ELISA with an IC_50_ of 27 ± 14 μM (Figure 2c). We next assessed the cell permeability of these molecules using a fluorescent homocoumaryl-containing derivative (*cyclo*-CLLhCouVY) (**14**), which binds HIF-1α with a *K*_D_ of 11 ± 1 μM (Figure 1c). Confocal imaging of HeLa cells treated with **14** (50 μM) showed homocoumarin fluorescence in both the cytoplasm and nucleus under normoxia and hypoxia (Figure 2d, lower panel blue channel), indicating cell permeability. Alexa568-conjugated HIF-1α antibody showed nuclear localization (of HIF-1α) in hypoxic cells, but a distribution resembling normoxia in cells treated with **14** (50 μM) (Figure 2d, red channel). This is consistent with inhibition of HIF-1 complex formation by **14**, which would prevent hypoxia-induced nuclear accumulation of HIF-1α.^21^ Finally, **57** (10 μM) reduced hypoxia-induced yellow fluorescent protein levels by ∼75% (c.f. normoxia) in a fluorescent-reporter cell assay (Figure S6c).^6^ Collectively, these orthogonal assays demonstrate that the above optimized cyclic peptides disrupt the HIF-1α/HIF-1β PPI and suppress HIF-1 activity.

**Figure 2:**
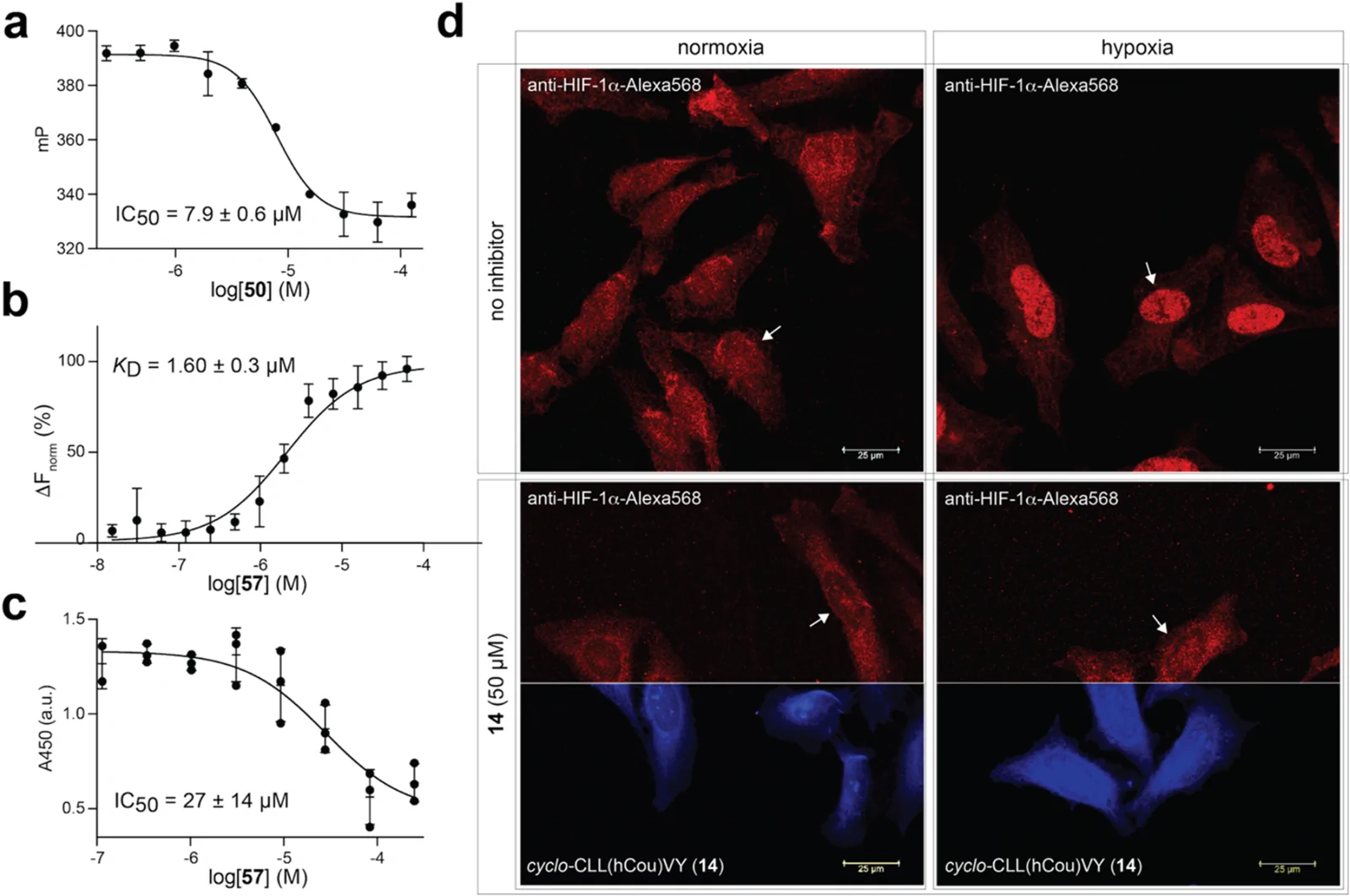
Assessing the activity of the optimized HIF-1 inhibitors. (a) Cyclic peptide **50** disrupts the HIF-1/HRE complex by FP with an IC_50_ of 7.9 ± 0.6 μM. Data is normalized such that 100% activity corresponds to HIF-1α/HIF-1β complex formation in the absence of inhibitor and 0% to the fluorescence polarization of free labeled HRE probe. (b) Cyclic peptide **57** binds HIF-1α by MST with a *K*_D_ of 1.60 ± 0.3 μM. (c) Cyclic Peptide **57** disrupts the HIF-1α/HIF-1β PPI by ELISA with an IC_50_ of 27 ± 14 μM. Data is normalized such that 100% corresponds to HIF-1α/HIF-1β binding in the absence of competitor cyclic peptide, and 0% to background absorbance in the absence of HIF-1β. (d) Visualizing the permeability and effect of coumarin-containing cyclic peptide **14** on HIF-1α localization in HeLa cells using Alexa568-conjugated anti-HIF-1α. Data are mean (*n* = 3) ± SD.

### Synthesis of VHL-Recruiting CP-PROTACs Targeting HIF-1α

We next asked whether the optimized *cyclo*-CLLFVY analogues could serve as warheads for PROTACs that inhibit HIF-1-mediated hypoxia-response by restoring HIF-1α degradation in hypoxic cells. We targeted positions 2 and 3 for linker installation as our SAR indicated that these leucines do not directly interact with HIF-1α, and so may provide distinct geometries for E3 ligase recruitment. Our PROTAC design focused on VHL recruitment to enable HIF-1α degradation in hypoxia, where prolyl hydroxylation is inactive. A key design goal was to develop bifunctional amino acids compatible with solid-phase peptide synthesis (SPPS) that incorporate both variable-length linkers and the VHL-binding ligand VH032.^22^ We synthesized these building blocks by cross-metathesis of benzyl-protected Fmoc-allylglycine with unsaturated esters of varying length, followed by one-pot hydrogenation (Figure S7a).

The affinities of **47**, **50**, and **55** for HIF-1α were statistically indistinguishable; we selected **47** as the warhead for CP-PROTAC synthesis because it offered marginally better solubility and synthetic tractability, which was considered advantageous for the synthesis of multiple CP-PROTACs. The unnatural amino acid linkers were incorporated at either position 2 or 3 in *cyclo*-CLL­(*p*-CF_3_)­FI­(*p*-BrF) (**47**) and coupled to VH032 post cyclization (Figure S7b) to give ten CP-PROTACs (**58-67**) (Figure 3). To benchmark these linkers against conventional alternatives, we prepared an additional eight CP-PROTACs containing PEG linkers (**68-75**) by incorporating glutamic acid at position 2 or 3 of **47**; after cyclization and side chain deprotection, an amino-(PEG)*_n_*-*t*-butyl ester (*n* = 4, 5, 6, or 7) was conjugated to the glutamic acid, followed by tBu removal and coupling of VH032 (Figure S7c). This yielded a total of eighteen CP-PROTACs (**58-75**) with varying linkers at the L2 and L3 positions (Figure 3).

**Figure 3:**
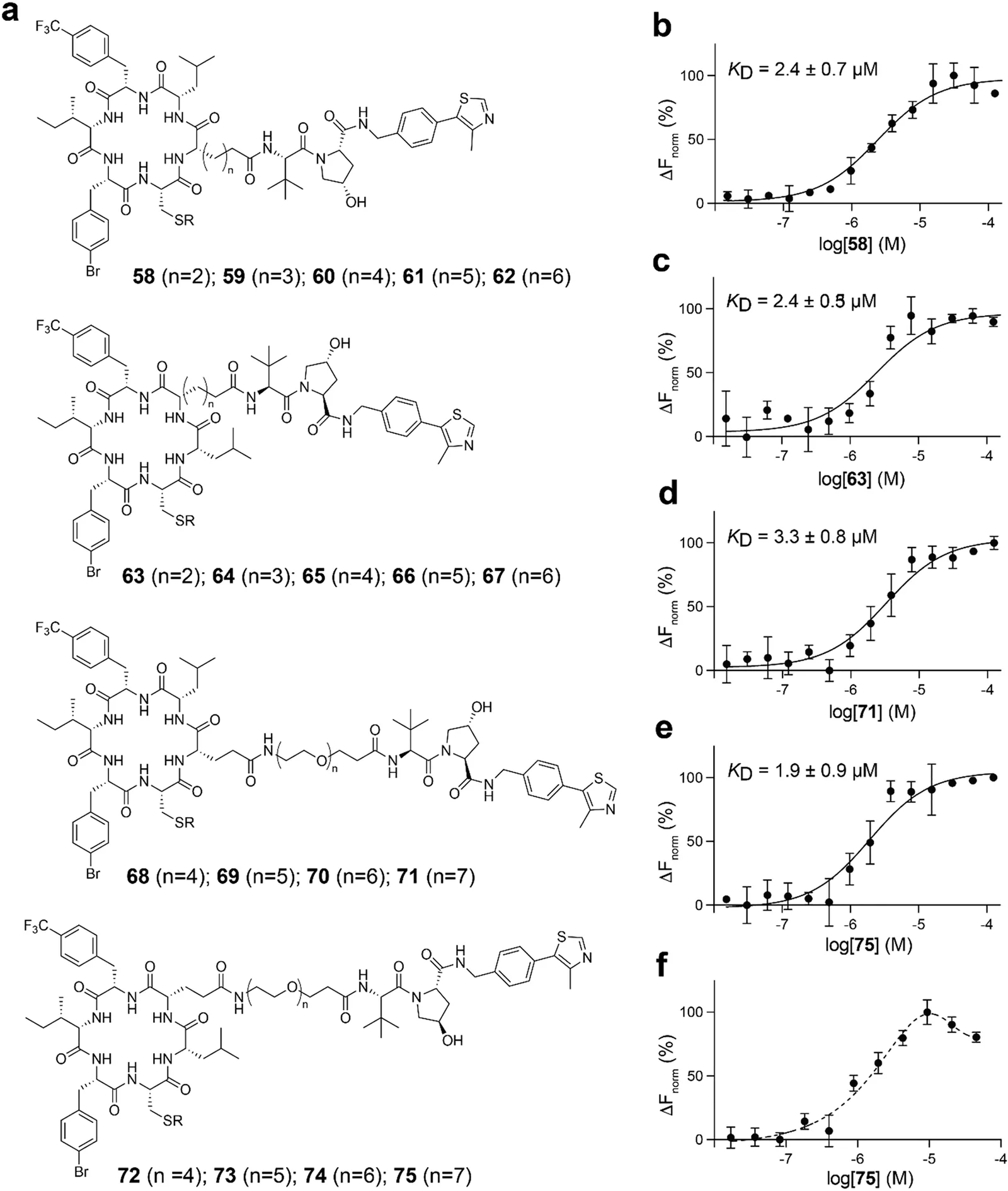
Structure and function of the CP-PROTACs tested in this study. (a) Structure of the molecules generated for this study. (b) CP-PROTAC **58** binds the PAS-B domain of HIF-1α with a *K*_D_ of 2.4 ± 0.7 μM. (c) CP-PROTAC **63** binds the PAS-B domain of HIF-1α with a *K*_D_ of 2.4 ± 0.5 μM. (d) CP-PROTAC **71** binds the PAS-B domain of HIF-1α with a *K*_D_ of 3.3 ± 0.8 μM. (e) CP-PROTAC **75** binds the PAS-B domain of HIF-1α with a *K*_D_ of 1.9 ± 0.9 μM. (f) MST titration of CP-PROTAC **75** into fluorescently labeled HIF-1α PAS-B in the presence of VHL–Elongin B/C, showing ternary complex formation with a hook effect at high **75** concentration. R = H for MST, and R = Tat (CGRKKRRQRRRPPQ) in cell experiments. Data are mean (*n* = 3) ± SD.

We confirmed that incorporation of the VHL ligand and linker into the cyclic peptide did not abrogate HIF-1α binding by testing representative CP-PROTAC with the shortest (**58** and **63**) and longest linkers (**71** and **75**) by MST. All four retained low-micromolar affinity for the HIF-1α PAS-B domain (Figure 3b–e), an 4-7-fold reduction relative to the parent cyclic peptide, and were judged sufficient for cellular evaluation. Notably, different linkers, their length, and attachment position have little to no effect on target affinity, allowing the contribution of attachment geometry to be assessed independently of target affinity.

To confirm that **75** bridges HIF-1α and VHL, we measured ternary complex formation by MST. Titration of **75** into fluorescently labeled HIF-1α PAS-B (10 nM) in the presence of the VHL–Elongin B/C complex (VCB, 50 nM) produced a dose-dependent change in thermophoresis that increased to a maximum at ∼1 μM **75** and declined at higher concentrations (Figure 3f). This descending phase is the hook effect expected for a bridged ternary complex, arising as excess PROTAC saturates the HIF-1α–**75** and **75**–VCB binary interactions at the expense of the bridged species. The MST trace of **75** binding to HIF-1α PAS-B (Figure 3e) and the matched control MST in the absence of VCB (Figure S8) both show a binary binding isotherm that saturates without a hook effect, confirming the formation of a HIF-1α–**75**–VHL ternary complex, with an abundance governed by CP-PROTAC concentration as expected for a productive degrader.

### Evaluating the Effect of CP-PROTACs on HIF-1α Levels in Cells

We next assessed the effect of CP-PROTACs on HIF-1α levels in HCT116 colorectal carcinoma cells, which show robust HIF-1α accumulation in hypoxia and in which HIF-1α inhibition has been linked to reduced tumor growth in model systems.^23^ HIF-1 upregulation and hypoxia-response was chemically induced with the prolyl hydroxylase inhibitor deferoxamine (DFX) (10 μM), followed by treatment with **58-75** (Figure 3, R = H), and quantification of HIF-1α levels by Western blot. None of the CP-PROTAC affected HIF-1α levels, likely due to insufficient cellular permeability. To facilitate internalization, we conjugated a cell-penetrating Tat peptide (CGRKKRRQRRRPPQ) to the cysteine present in position 1 of all our CP-PROTACs *via* a disulfide bond that would be reduced upon internalization.^18^ The resulting Tat-tagged CP-PROTACs **58-75** (Figure 3, R = Tat) were evaluated at 10 μM and 20 μM in HCT116 cells treated with DFX (10 μM). Most CP-PROTACs with the bifunctional amino acid linkers (**58-67**) showed dose-dependent HIF-1α degradation with activity increasing with linker length (Figure 4a,4b). The most potent degrader in the series was **67**, with the amino acid linker in the position 3, which reduced HIF-1α levels by ∼70% at 20 μM. All Tat-CP-PROTACs containing a PEG linker (**68-75**) degraded HIF-1α in hypoxic HCT116 cells (Figure 4c,d). In both series, linkers conjugated to position 3 (**63-67** and **72-75**) outperformed equivalent CP-PROTACs with the linker in position 2 (**58-62** and **68-71**). The most active compound overall was **75**, which achieved ∼70% degradation of HIF-1α at 10 μM and > 80% at 20 μM (Figure 4c). A dose–response experiment (100 nM to 40 μM) gave a DC_50_ of 1.4 ± 0.1 μM for this compound in HCT116 cells (Figure 4e). Similar activity was observed in U2OS osteosarcoma cells (DC_50_ = 2.6 ± 0.1 μM) (Figure 4f), indicating that degradation was not cell-line specific. In the presence of the proteasome inhibitor MG132 (20 μM),^24^ **75** had no effect on HIF-1α (Figure S10c), consistent with UPS-dependent degradation. A negative control lacking the VH032 ligand did not degrade HIF-1α (Figure S10d), confirming the requirement for VHL recruitment. Together, these controls establish that **75** induces UPS- and VHL-dependent HIF-1α degradation.

**Figure 4:**
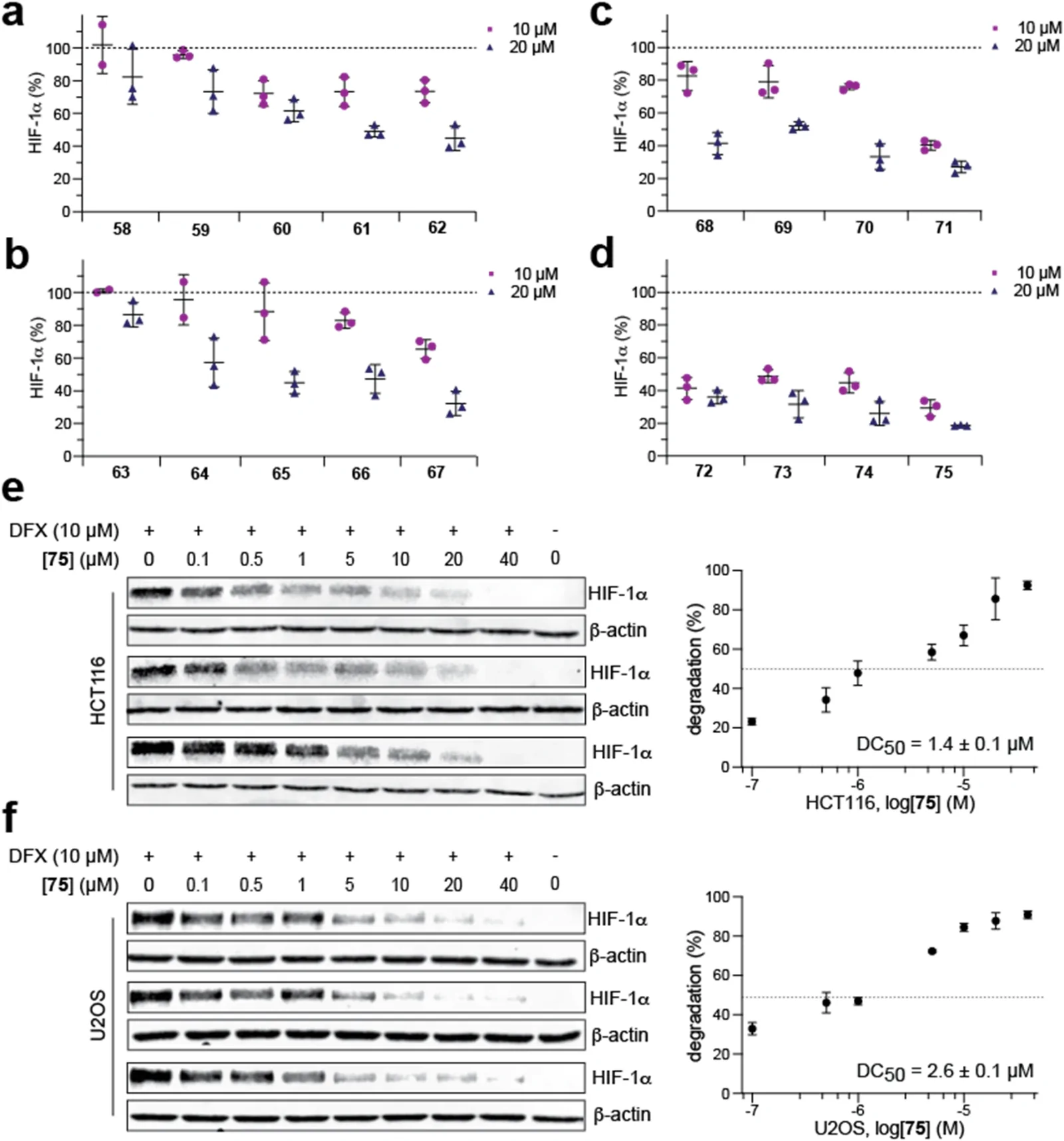
Assessing the activity of CP-PROTACs in cells treated with 10 μM DFX. (a) Quantification of HIF-1α protein levels in hypoxic HCT116 cells treated with CP-PROTACs **58-62** by Western blot. (b) Quantification of HIF-1α protein levels in hypoxic HCT116 cells treated with CP-PROTACs **63-67** by Western blot. (c) Quantification of HIF-1α protein levels in hypoxic HCT116 cells treated with CP-PROTACs **68-71** by Western blot. (d) Quantification of HIF-1α protein levels in hypoxic HCT116 cells treated with CP-PROTACs **72-75** by Western blot. (e) Western blot for HIF-1α in HCT116 cells treated with varying doses of **75** (triplicate), and corresponding degradation curve with calculated DC_50_. (f) Western blot for HIF-1α in U2OS cells treated with varying doses of **75** (triplicate), and corresponding degradation curve with calculated DC_50_. HIF-1α levels were quantified by densitometry, normalized to the β-actin loading control, and expressed as a percentage where 100% corresponds to HIF-1α in untreated (DMSO) cells under hypoxia (DFX, 10 μM) and 0% corresponds to HIF-1α in normoxic cells; cells were treated with CP-PROTAC and incubated in normoxia for 1 h, followed by the addition of DFX and incubation for 6 h; Western blots underpinning data in panels (a–d) are in Figure S9; data are mean (*n* = 3) ± SD.

### Evaluation of Cyclic Peptide PROTACs in Hypoxia

We next evaluated CP-PROTAC activity under physiologically relevant hypoxic conditions. We initially tested **75** in HCT116 cells incubated in 1% oxygen for 6 h; however, **75** did not reduce HIF-1α levels at 1% oxygen at doses up to 40 μM. We considered two possibilities: that the warhead fails to engage the form of HIF-1α present at 1% O_2_, or that continuous resynthesis of HIF-1α outpaces degrader-mediated clearance. To probe this, we first measured HIF-1α levels in HCT116 cells after incubation for 1 h and 6 h at 1% O_2_, and found that the HIF-1α pool is not static (Figure 5a), accumulating ∼3-fold over this period (Figure S10a). This continued accumulation is consistent with reports that HIF-1α transcription is upregulated in hypoxia^25,26^ while translation rates are maintained at normoxic rates.^27^ To assess whether degradation by **75** is functional at 1% O_2_ when HIF-1α levels are lower, we measured its effect in cells after 1 h and 6 h of incubation at 1% O_2_ and only observed degradation of HIF-1α in the 1 h samples (Figure 5b), demonstrating that the warhead engages HIF-1α and recruits the degradation machinery at 1% O_2_ in these samples. To assess the contribution of HIF-1α replenishment to the inactivity observed after 6 h, we used cycloheximide to block protein synthesis in cells after incubation at 1% O_2_ for 6 h. Cycloheximide alone reduced HIF-1α levels, consistent with ongoing turnover, and addition of **75** reduced HIF-1α further (Figure 5c), demonstrating functional degradation of HIF-1α by **75**. Together, these data show that **75** engages HIF-1α and is degradation-competent at 1% O_2_, and that the loss of net degradation reflects a kinetic competition in which resynthesis exceeds the clearance flux achievable as the HIF-1α pool accumulates.

**Figure 5:**
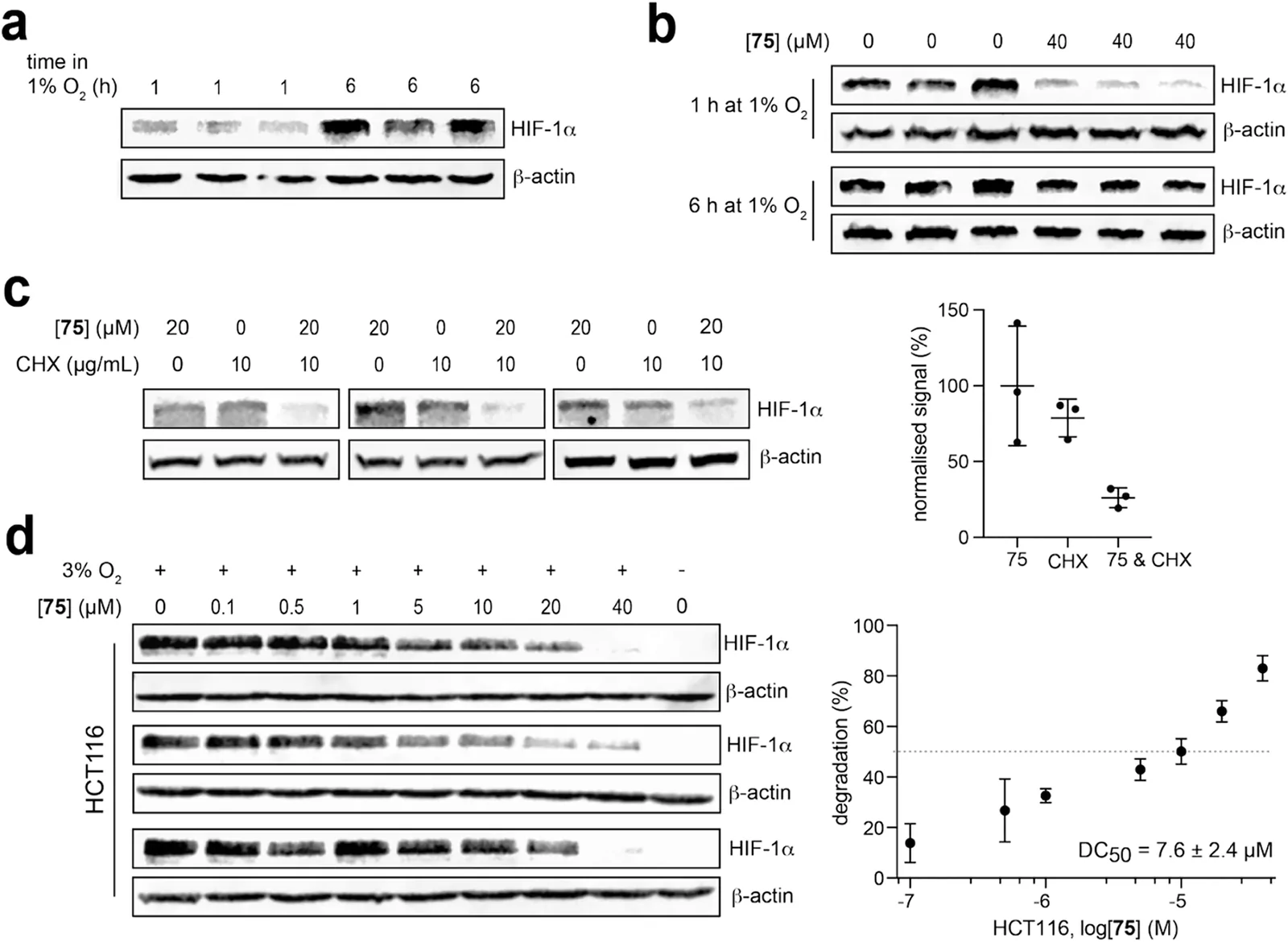
Mechanism of the oxygen-dependence of CP-PROTAC activity. (a) HIF-1α levels in HCT116 cells after 1 h and 6 h incubation at 1% O_2_, quantified by Western blot. (b) Effect of CP-PROTAC **75** (40 μM) on HIF-1α levels in HCT116 cells after 1 h and 6 h at 1% O_2_; for quantification of bands, see Figure S10b. (c) Effect of CP-PROTAC **75** (20 μM) on HIF-1α levels in HCT116 cells at 1% O_2_ (6 h) in the presence and absence of cycloheximide (10 μg/mL for 30 min); lanes show DMSO, DMSO + cycloheximide, and CP-PROTAC **75** + cycloheximide. (d) Western blot for HIF-1α in hypoxic HCT116 cells treated with varying doses of **75** (triplicate), and degradation curve of HIF-1α levels and calculated DC_50_. HIF-1α levels were quantified by densitometry, normalized to the β-actin loading control, and expressed as a percentage where 100% corresponds to HIF-1α in untreated (DMSO) cells under hypoxia and 0% corresponds to HIF-1α in normoxic cells; cells were treated with CP-PROTAC and incubated in normoxia for 1 h, followed by incubation in hypoxia for 1 or 6 h; data are mean (*n* = 3) ± SD.

To identify a level of hypoxia in which **75** is functional, we compared HIF-1α levels in HCT116 cells cultured in 1, 2, 3, and 4% oxygen, alongside DFX treatment (10 μM) (Figure S11a). Quantification showed ∼7-fold higher HIF-1α at 1% oxygen compared to hypoxia induced with 10 μM DFX, with ∼3-fold, ∼2.5 fold, and ∼2-fold increases at 2, 3, and 4% oxygen, respectively. At 3% oxygen, **75** (20 μM) reduced HIF-1α levels by 75%, whereas no degradation was observed at 2% oxygen (Figure S11b). **75** caused dose-dependent HIF-1α degradation in hypoxic HCT116 (3% O_2_) with a DC_50_ of 7.6 ± 2.4 μM (Figure 5d).

We assessed **75** activity using a U2OS reporter cell line expressing HRE-driven luciferase, in which HIF-1α depletion or HIF-1 PPI inhibition reduces the luciferase signal. **75** produced a dose-dependent reduction in luciferase activity with an IC_50_ of ∼5 μM (Figure 6a). We also tested **67** and observed a dose-dependent decrease in luciferase activity with an IC_50_ of between 5 and 10 μM, consistent with earlier HCT116 data (Figure 6b). Both CP-PROTACs showed superior activity at the same dose compared to the parent Tat-tagged cyclic peptide **47** (Figure 6c), suggesting a synergistic effect from both degradation and PPI inhibition. Quantitative PCR (qPCR) confirmed dose-dependent suppression of the HIF-1 target gene Carbonic Anhydrase 9 (CAIX) by **67** and **75** in hypoxic HCT116 cells (3% O_2_), with 20 μM of **75** reducing CAIX expression in hypoxic HCT116 cells toward normoxic levels (Figure 6d). Again, we observed significantly better CAIX inhibition with **67** and **75** versus the parent cyclic peptide **47**, which was Tat-tagged, to eliminate any difference to the CP-PROTACs due to potential differences in permeability (Figure 6d). We also assessed the effect of **75** (20 μM) on HIF-2α by Western blot and observed no change in HIF-2α levels (Figure S11c), indicating that **75** retains the HIF-1 isoform-specificity of its parent cyclic peptide.^18^

**Figure 6:**
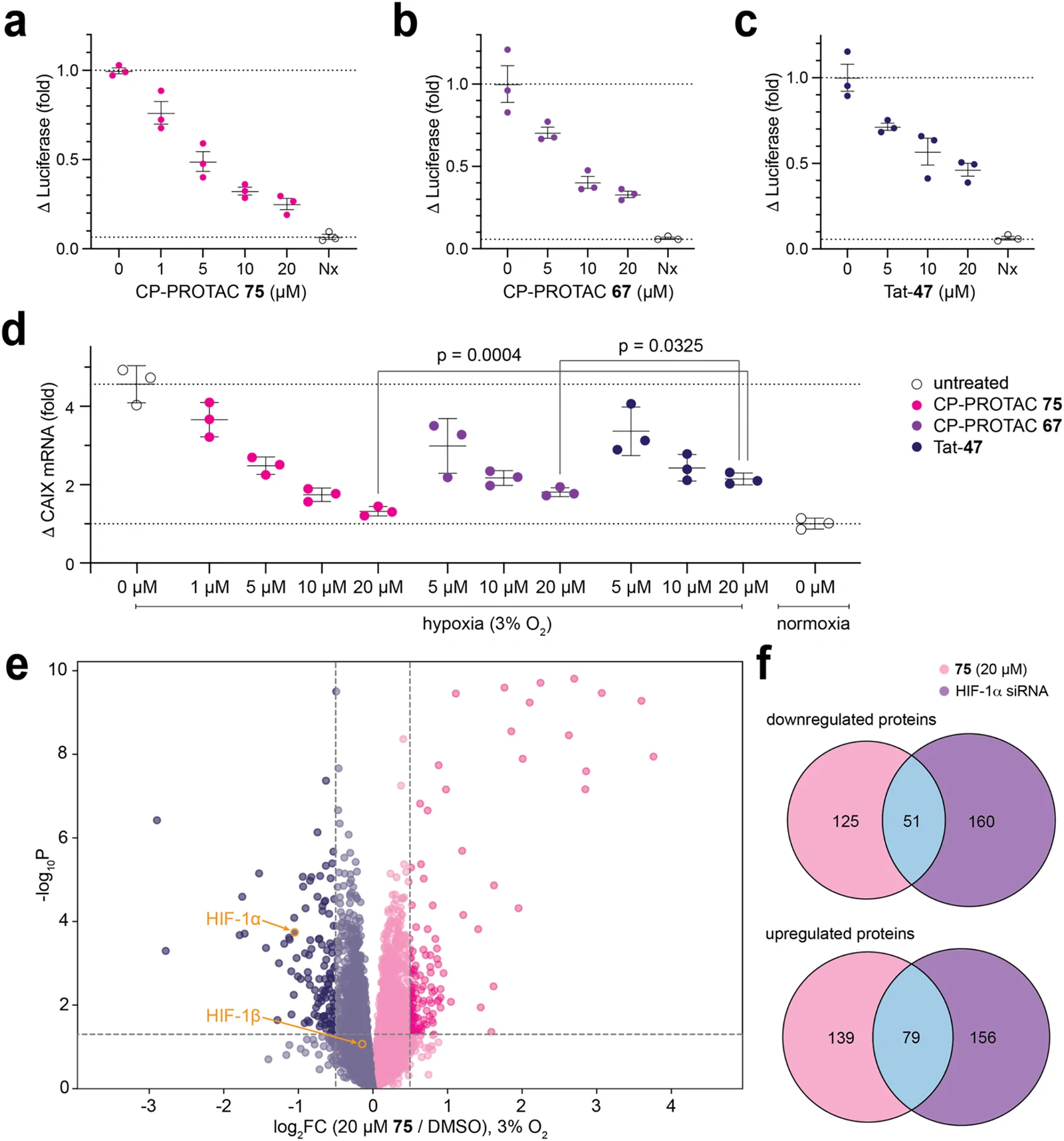
Assessing the activity of CP-PROTACs in cells incubated in hypoxia (3% O_2_). (a) The effect of **75** in U2OS-HRE-Luc cells incubated in hypoxia. Data is normalized to luciferase signal in untreated hypoxic cells (1.0) relative to those in normoxia (0.0). (b) The effect of **67** in U2OS-HRE-Luc cells incubated in hypoxia. Data is normalized to luciferase signal in untreated hypoxic cells (1.0) relative to those in normoxia (0.0). (c) The effect of **Tat-47** in U2OS-HRE-Luc cells incubated in hypoxia. Data is normalized to luciferase signal in untreated hypoxic cells (1.0) relative to those in normoxia (0.0). (d) The effect of **75**, **67**, **Tat-47**, on the HIF-1-mediated expression of the CAIX gene in hypoxic HCT116 cells by qPCR. CAIX mRNA levels are normalized to 18S and HPRT1 housekeeping genes, and the data are expressed as fold change relative to untreated hypoxic cells vs normoxic cells (1). Statistical comparison by one-way ANOVA with Dunnett’s multiple comparisons test against the Tat-47 control; *p*-values as indicated. (e) Whole-cell proteome analysis of HCT116 cells in 3% O_2_ treated with **75** (20 μM) vs vehicle treatment; HIF-1α and HIF-1β are highlighted (upregulation log2FC > 0.5, downregulation log2FC < −0.5, *p*-values <0.05 in each case). (f) Overlap of proteins modulated by **75** and HIF-1α siRNA. Cells were treated with CP-PROTAC and incubated in normoxia for 1 h, followed by incubation in hypoxia for 6 h; data are mean (*n* = 3) ± SD.

We next profiled proteome-wide changes induced by **75** under conditions where degradation is observed (HCT116, 3% O_2_) by using quantitative mass spectrometry. Relative to normoxia, cells cultured in 3% O_2_ showed altered abundance of 217 proteins (115 increased; 102 decreased; threshold |log2FC| > 0.5, *p* < 0.05) (Figure S12a). Notably, the proteins modulated at 3% O_2_ showed limited overlap with those observed at 1% O_2_,^28^ indicating distinct transcriptional programs engaged by mild versus severe hypoxia.

Treatment with **75** (20 μM) in 3% O_2_ produced broad proteome remodeling consistent with suppression of hypoxia signaling (176 proteins decreased and 206 increased relative to 3% O_2_ vehicle); **75** caused the loss of HIF-1α protein but not HIF-1β protein (Figure 6e). To benchmark these changes against genetic depletion of the target, we quantified proteome changes following HIF-1α siRNA treatment of HCT116 cells in 3% O_2_ (Figure S12b). A subset of proteins was regulated by both **75** and HIF-1α siRNA, supporting pathway-directed consequences of HIF-1α loss (Figure 6f). Plotting the fold-changes induced by HIF-1α siRNA against those induced by **75** revealed a positive correlation across the quantified proteome (Pearson *r* = 0.68) (*n* = 7975 proteins agreeing to within 0.5 log_2_FC); *r* = 0.53 across all 8278 proteins quantified in both datasets (Figure S13), indicating that the two perturbations remodel the proteome in a concordant direction. Exemplar canonical HIF-1 target proteins were reduced by both perturbations, although the magnitude of these changes was modest and largely below our 0.5 log_2_FC threshold. This is not unexpected, as such targets are defined predominantly from studies conducted at 1% O_2_, and as noted above, the hypoxic program engaged at 3% O_2_ overlaps only partially with that at 1% O_2_. Their limited movement is also consistent with the long half-lives of these abundant metabolic enzymes and with protein-level responses lagging transcriptional suppression over the treatment window. Because global proteomics primarily captures downstream regulation, these data cannot by themselves distinguish direct degradation from indirect effects; however, the convergence with genetic depletion supports on-pathway activity, and future time-resolved and ubiquitination-focused profiling can further refine selectivity.

Beyond confirming on-pathway activity, these data speak to the physiological character of the CP-PROTAC action. Treatment with **75** shifted the hypoxic proteome toward the normoxic state, attenuating rather than abolishing the hypoxia-response program. Such graded normalization may be therapeutically preferable to complete pathway ablation as HIF-1 supports physiological as well as pathological oxygen adaptation, and an agent that returns pathological hypoxia signaling toward near-normoxic levels (rather than eliminating it) may retain efficacy while preserving normal oxygen-sensing function. The catalytic, substoichiometric nature of degradation is well suited to this tunable mode of action, and the partial suppression of the HIF-1 target gene CAIX toward normoxic levels (Figure 6d) is consistent with it. We note that the incomplete overlap of modulated proteins between cells treated with **75** versus HIF-1a siRNA (Figure 6f) is expected from the distinct kinetics of the two perturbations (transient protein degradation versus sustained transcript knockdown), each carrying its own secondary effects, such that convergence, rather than absolute overlap, is the appropriate expectation for on-pathway activity.

## Conclusion

Cyclic peptides are an established modality in drug discovery with multiple clinical successes. Here, we report a practical route from cyclic peptides to functional degraders. Starting from a SICLOPPS-derived HIF-1α inhibitor, we rationally improved affinity and functional disruption of the HIF-1α/HIF-1β–DNA complex and then converted the optimized cyclic peptide into a set of cyclic-peptide PROTACs (CP-PROTACs) by appending a VHL ligand through SPPS-compatible bifunctional amino acid building blocks. In cells, Tat–disulfide delivery enabled CP-PROTAC entry and revealed a clear VHL-ligand- and proteasome-dependent reduction of HIF-1α. Together with rescue by proteasome inhibition and loss of activity for a control lacking the VHL ligand, these data support a recruitment-driven degrader mechanism rather than simple antagonism.

A central finding is that degrader activity is oxygen-state dependent: robust depletion is observed under moderate hypoxia (∼3% O_2_) but is lost under more severe hypoxia (∼1% O_2_). Our data establishes kinetic competition between hypoxia-driven HIF-1α accumulation (via stabilization and feedback-regulated synthesis),^25,26^ and the maximum degradation flux achievable through recruited ubiquitin–proteasome machinery as the cause. More broadly, effective degradation requires that degrader-driven clearance outpaces state-driven target accumulation; under severe hypoxia, replenishment and stabilization may exceed the achievable clearance ceiling. These results have implications for PROTAC-mediated depletion of rapidly replenished stress–response proteins. Because tumor hypoxia is heterogeneous, typically ranging from 1 to 5% in patient samples,^29–31^ HIF-1α degradation may be most tractable in modestly hypoxic tumors or tumor regions, while the most severely hypoxic cores, which are frequently the most therapy-resistant, may remain refractory to degrader-based approaches. This constraint is unlikely to be specific to the present compounds, as any degrader acting on a rapidly synthesized, stress-stabilized target will face the same kinetic ceiling, and extending efficacy into severely hypoxic microenvironments may require strategies that raise catalytic clearance capacity or that combine degradation with suppression of target synthesis.

The chemistry described here expands the accessible design space for degraders. Cyclic peptides engage targets that are challenging for small molecules, such asprotein–protein interactions , and the modular bifunctional amino acids used in this work allow systematic variation of attachment point and linker length without abandoning standard SPPS workflows. We anticipate that this strategy will be transferable to other cyclic-peptide ligands discovered by in-cell or *in vitro* selection approaches, enabling rapid “hit-to-degrader” iteration. Several considerations will shape the generality of this strategy. Our data show that both attachment position and linker length materially affect degradation efficiency (Figure 4), consistent with a requirement for productive ternary-complex geometry rather than mere target engagement;^32,33^ the bifunctional building blocks are designed to allow this parameter space to be scanned systematically within a standard SPPS workflow. Cell permeability remains the principal constraint; the unmodified CP-PROTACs did not reduce HIF-1α levels, and cellular activity required Tat-mediated delivery, a limitation shared broadly across intracellular cyclic-peptide and peptide-degrader modalities. Conjugation of an E3 ligand and linker further increases molecular size and polarity, which is likely to compound this challenge for larger or more polar warheads, and we therefore regard delivery and permeability as the foremost targets for future development. We have not assessed anti-proliferative activity here; HIF-1 principally drives adaptive survival under hypoxic conditions *in vivo*, and a full assessment of anti-tumor phenotype is an important next step beyond the scope of the present study, which establishes the degrader chemistry and its mechanism. Finally, although we employed the VHL-recruiting ligand VH032 (chosen because it recruits VHL independently of the prolyl hydroxylation that is suppressed in hypoxia, and is thus well matched to a hypoxic target), the building-block format itself is agnostic to the identity of the E3 ligand. Alternative recruiters compatible with the same SPPS-based chemistry, such as cereblon-directed ligands, could, in principle, be incorporated by the same route, extending the approach to targets and tissues where a different ligase is preferable. Further optimization of delivery and permeability should decouple degrader potency from uptake and release constraints. Overall, the work here provides a general framework for converting cyclic peptides into CP-PROTACs, extending targeted degradation to proteins that are difficult to drug with small molecules.

## Supplementary Material



## Data Availability

The data supporting the findings of this study are available within the paper and its Supporting Information. The raw data underpinning this study are openly available from the University of Southampton data repository at https://doi.org/10.5258/SOTON/D4001.

## Notes

Binding curves for *cyclo*-XLLFVY (Figure S1); binding curves for *cyclo*-CLLXVY (Figure S2); binding curves for *cyclo*-CLL­(*p*-CF_3_)­FVX (Figure S3); binding curves for *cyclo*-CLL­(*p*-CF_3_)­FX­(*p*-Br)­F (Figure S4); binding curves for *cyclo*-CLX­(*p*-CF_3_)­FI­(*p*-Br)F (Figure S5); cell activity of cyclic peptide 57 (Figure S6); linker and CP-PROTAC synthesis (Figure S7); MST of 75 binding to HIF-1α (Figure S8); Western blots of the effect of CP-PROTACs on HIF-1α in HCT116 cells (Figure S9); control experiments CP-PROTAC 75 (Figure S10); effect of CP-PROTAC 75 in hypoxic HCT116 cells (Figure S11); volcano plots for proteomics experiment (Figure S12); scatter plot of 75 treatment vs HIF-1α siRNA (Figure S13); material and methods; HPLC and MS data for all cyclic peptides and CP-PROTRACs (PDF)
