# Supplementary material for: Cyclic Peptide PROTACs Restore VHL-Mediated HIF-1α Degradation in Hypoxia
Source: J Am Chem Soc. 2026 Sep 14;148(37):39580–90. doi: 10.1021/jacs.6c08218 (PMC13614306; doi:10.1021/jacs.6c08218)
Supplement: Supplementary file 1 [file ja6c08218_si_001.pdf]

Supplementary information

Cyclic peptide PROTACs restore VHL-mediated HIF-1α degradation in hypoxia

Alexander McDermott,[1,#] Reece Gardner,[1,#] Monika Papayova,[1] Agnieszka B. Wisniewska,[1] Cyrielle Doigneaux,[1] Soran Mohammed,[1] Ali Tavassoli[1,*]

1. School of Chemistry and Chemical Engineering, University of Southampton, Southampton, U.K.
* e-mail: a.tavassoli@soton.ac.uk

# authors contributed equally to this work

Supplementary Figures

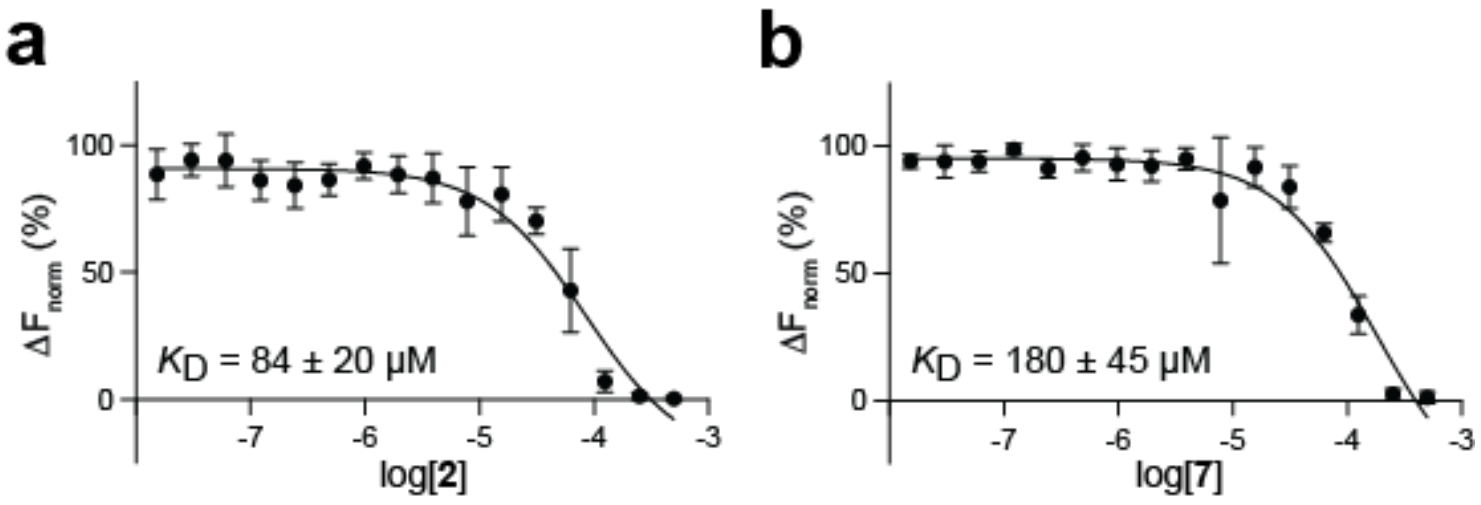

Figure S1. Binding curves and affinities for *cyclo*-XLLFVY derivatives to HIF-1α by MST. a) X = dC. **b)** X = hC. All data shown as mean (n = 3) ± SD.

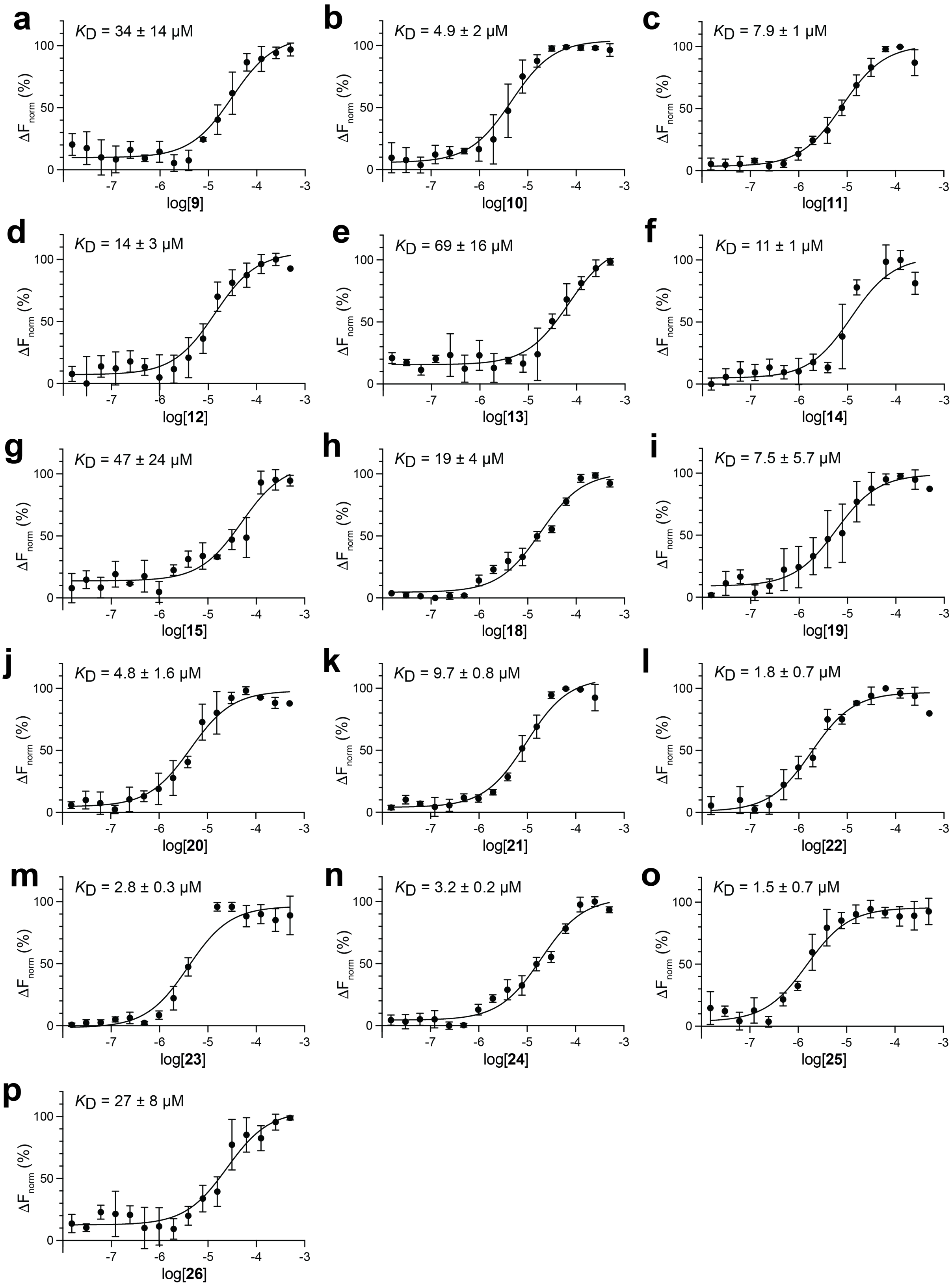

Figure S2. Binding curves and affinities for *cyclo*-CLLXVY derivatives to HIF-1α by MST. a) X = hF. **b)** X = dF. **c)** X = Nal. **d)** X = Cha. **e)** X= Phg. **f)** X = hCou. **g)** X = Pal. **h)** X = (*p*-F)F. **i)** X = (*p*-Cl)F. **j)** X = (*p*-Br)F. **k)** X = (*p*-I)F. **l)** X = (*p*-CF_3)F. **m)** X = (*p*-CF_3)dF. **n)** X = (*p*-Ph)F. **o)** X = (*p*-tBu)F. **p)** X = (*p*-NO_2)F. All data is mean (n = 3) ± SD.

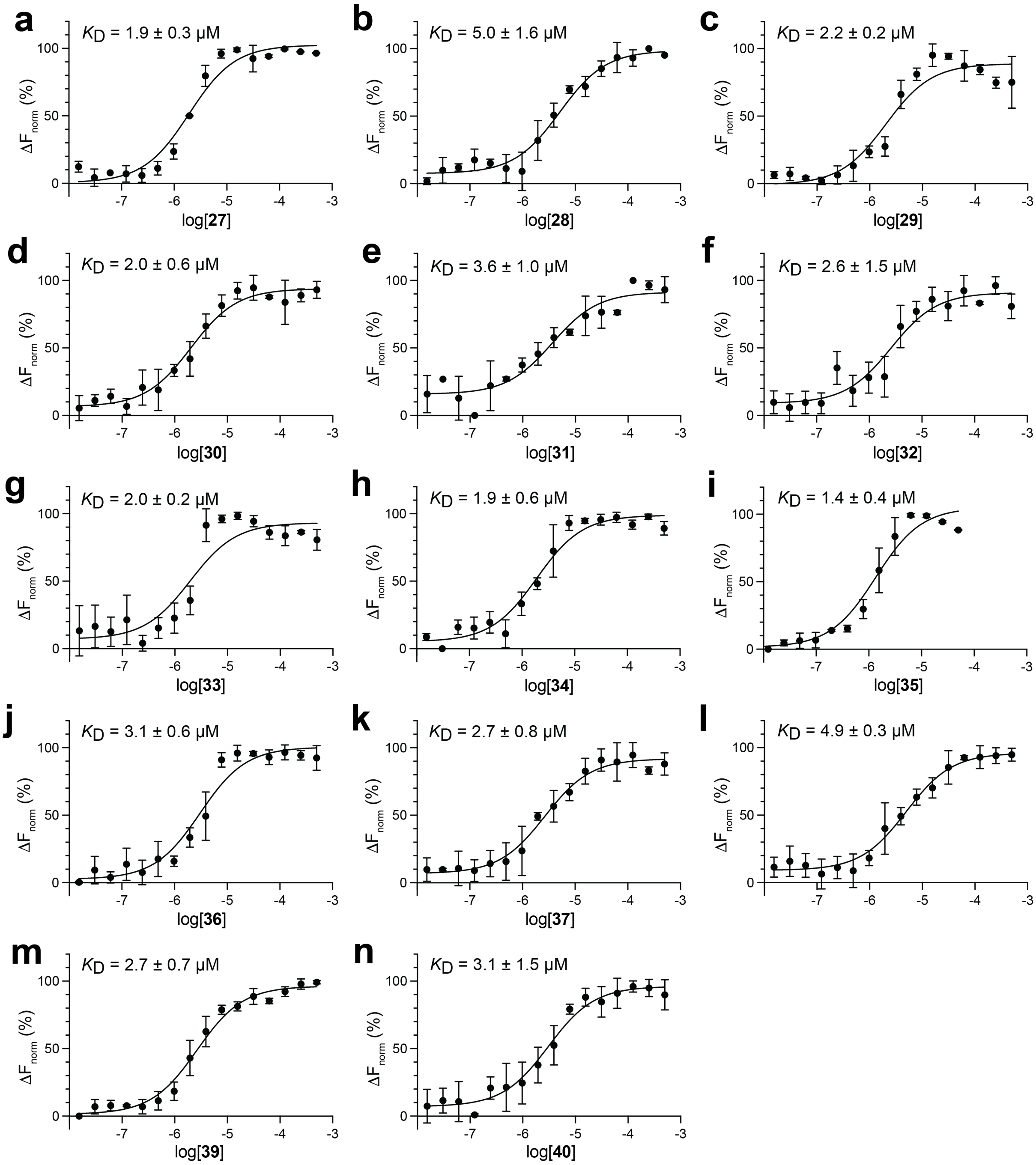

Figure S3. Binding curves and affinities for *cyclo*-CLL(*p*-CF_3)FVX derivatives to HIF-1α by MST. a) X = Nal. **b)** X = Cha. **c)** X= Phg. **d)** X = F. **e)** X = Pal. **f)** X = (*p*-OMe)F. **g)** X = (*p*-F)F. **h)** X = (*p*-Cl)F. **i)** X = (*p*-Br)F. **j)** X = (*p*-I)F. **k)** X = (*p*-CF_3)F. **l)** X = (*p*-Ph)F. **m)** X = (*p*-tBu)F. **n)** X = (*p*-NO_2)F. All data is mean (n = 3) ± SD.

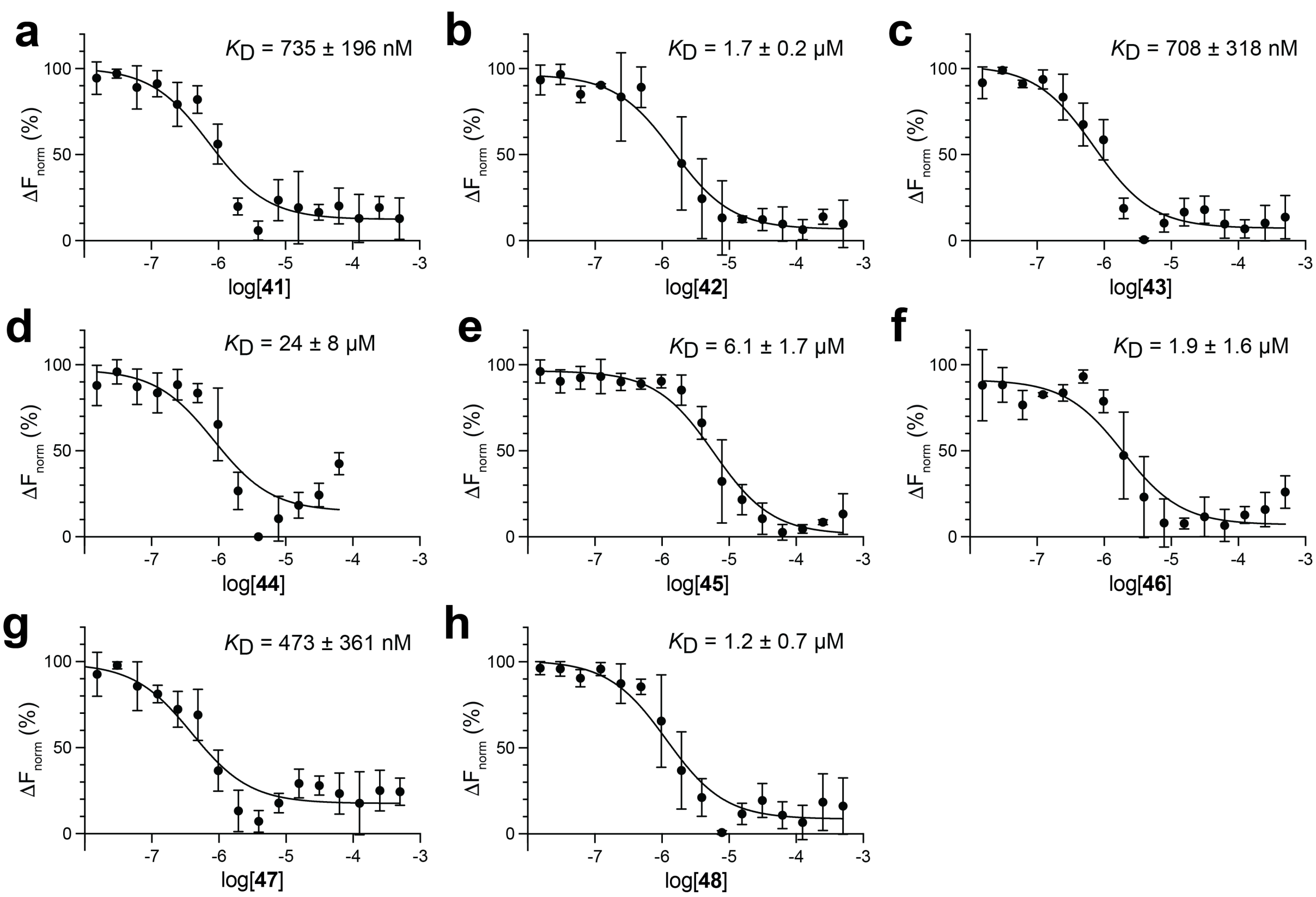

Figure S4. Binding curves and affinities for *cyclo*-CLL(*p*-CF_3)FX(*p*-Br)F derivatives to HIF-1α by MST. a) X = hL. **b)** X = Nle. **c)** X= L. **d)** X = Nva. **e)** X = Abu. **f)** X = Aib. **g)** X = I. **h)** X = Prop. All data is mean (n = 3) ± SD.

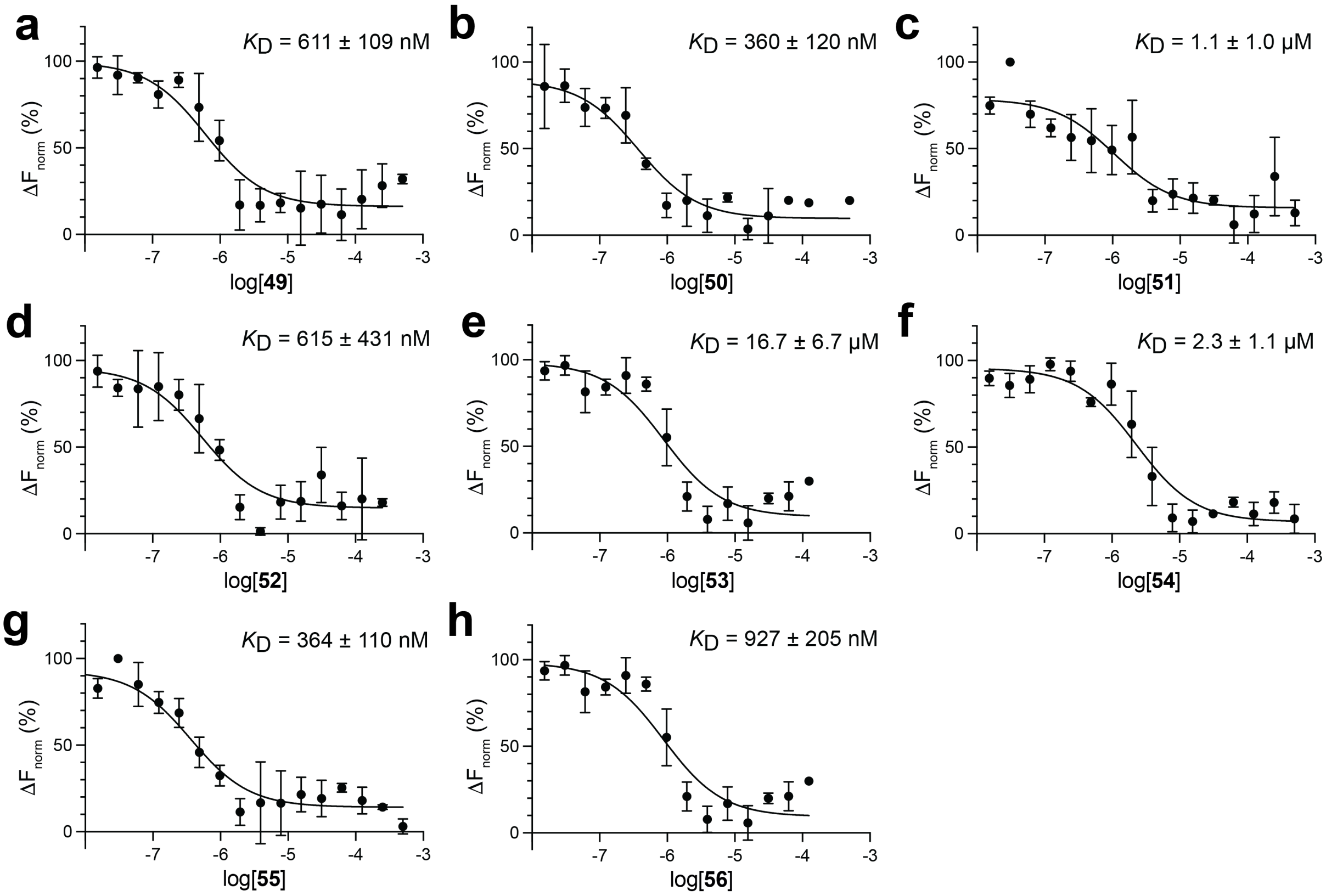

Figure S5. Binding curves and affinities for *cyclo*-CLX(*p*-CF_3)FI(*p*-Br)F derivatives to HIF-1α by MST. a) X = hL. **b)** X = Nle. **c)** X= V. **d)** X = Nva. **e)** X = Abu. **f)** X = Aib. **g)** X = I. **h)** X = Prop. All data is mean (n = 3) ± SD.

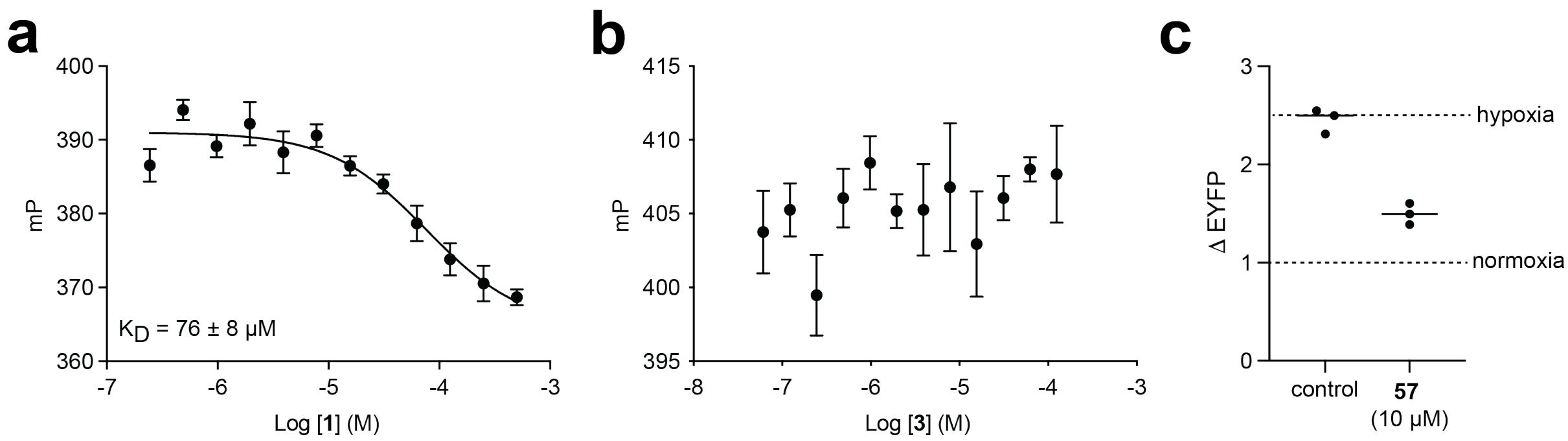

Figure S6. FP control experiments and cell activity of cyclic peptide 57. a) *cyclo*-CLLFVY (**1**) disrupts the HIF-1/HRE complex by FP with an IC_{50} of 76 ± 8 µM. **b)** Negative control *cyclo*-ALLFVY (**3**) does not disrupt the HIF-1/HRE complex by FP. **c)** 10 µM *cyclo*-CKNle(*p*-CF_3)FI(*p*-Br)F (**57**) reduced the hypoxia-induced yellow fluorescent protein (EYFP) by ~75% in a hypoxic Panc-1 fluorescent-reporter cell assay. All data is mean (n = 3) ± SD.

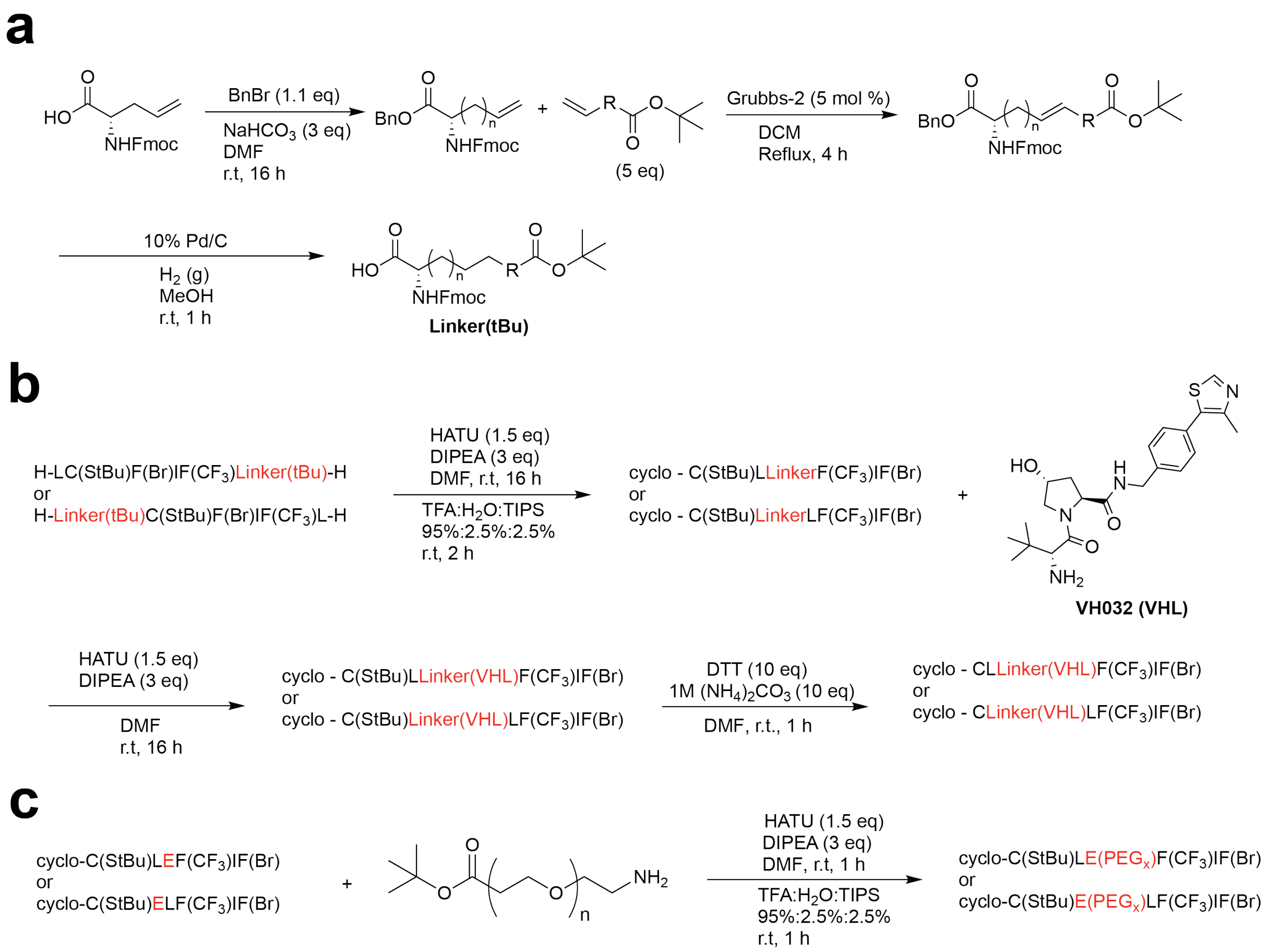

Figure S7. Linker and CP PROTAC synthesis. a) Synthetic pathway for the amino acid linkers. **b)** Synthesis of CP-PROTACs incorporating VH032. **c)** Synthesis of PEG-linked CP PROTACs.

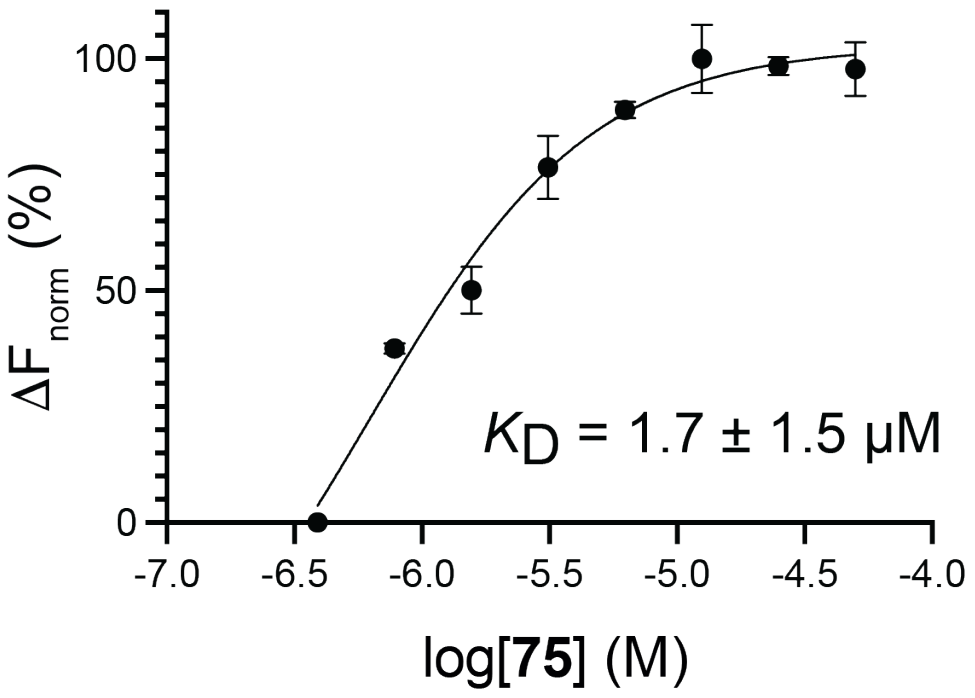

Figure S8. MST of CP-PROTAC 75 binding to the PASB domain of HIF-1α. Assay was conducted at the same time and under the same conditions as the data in Figure 3f, but in the absence of VHL-Elongin B/C, and shows the absence of a hook effect. Data is mean (n = 3) ± SD

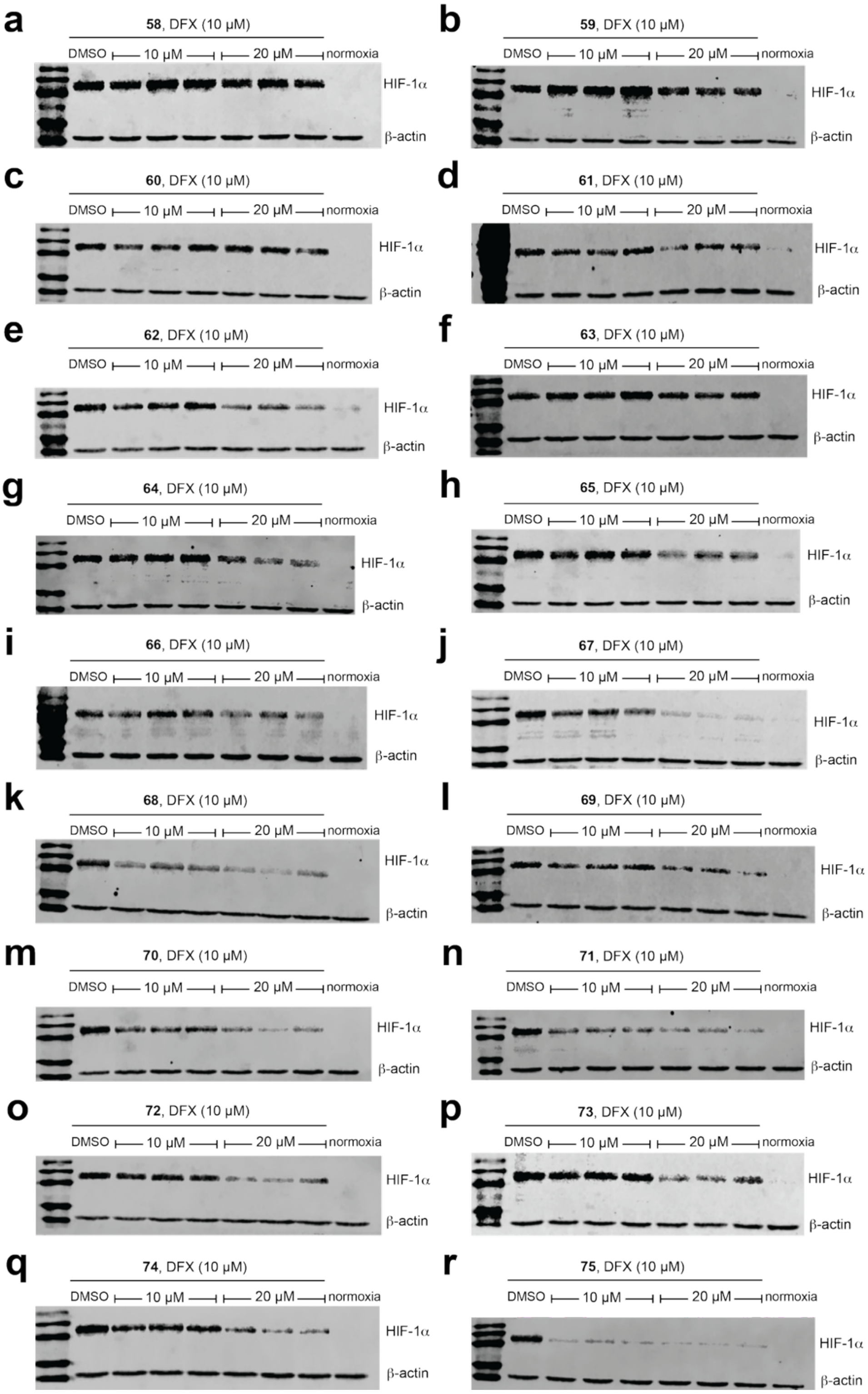

Figure S9. Western blots of the effect of CP-PROTACs on HIF-1α in HCT116 cells. Each blow shows: lane 1: Ladder, lane 2: DMSO Hypoxia control, lane 3-5: CP-PROTAC 10 µM, lane 6-8: CP-PROTAC 20 µM, lane 9: DMSO Normoxia control. **a)** CP-PROTAC **58**. **b)** CP-PROTAC **59**. **c)** CP-PROTAC **60**. **d)** CP-PROTAC **61**. **e)** CP-PROTAC **62**. **f)** CP-PROTAC **63**. **g)** CP-PROTAC **64**. **h)** CP-PROTAC **65**. **i)** CP-PROTAC **66**. **j)** CP-PROTAC **67**. **k)** CP-PROTAC **68**. **l)** CP-PROTAC **69**. **m)** CP-PROTAC **70**. **n)** CP-PROTAC **71**. **o)** CP-PROTAC **72**. **p)** CP-PROTAC **73**. **q)** CP-PROTAC **74**. **r)** CP-PROTAC **75**. Quantificatin of this data is in Figure 4a-d.

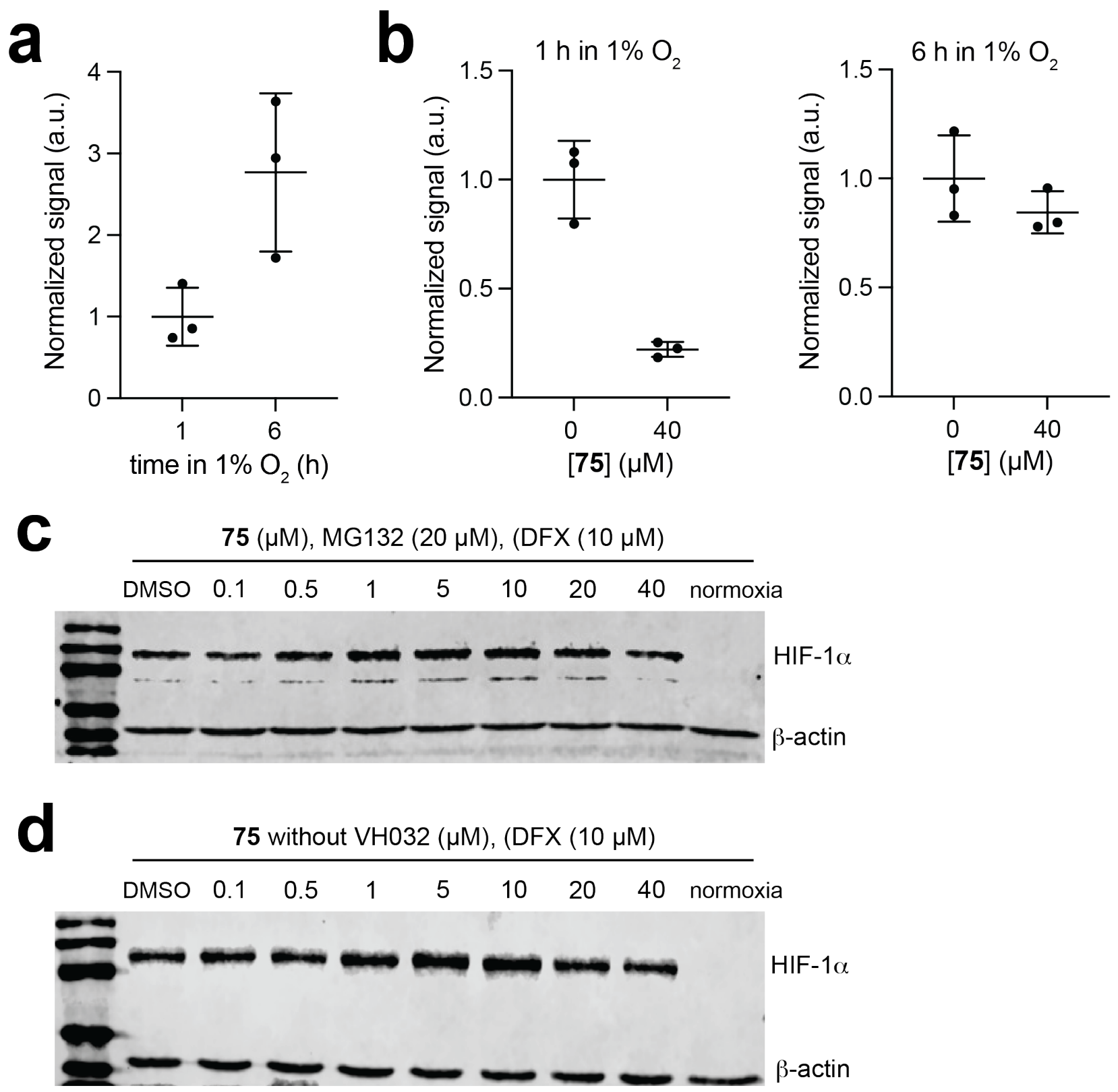

Figure S10. Western blots of HIF-1α in HCT116 cells. a) quantification of the western blot in Figure 5a. **b)** Quantification of the Western blot in Figure 5b. **c)** Cells treated with CP-PROTAC **75** (0.1-40 μM), MG132 (20 μM), and DFX (10 μM). **d)** cells treated with a negative control variant of **75** without VH032 (0.1-40 μM) and DFX (10 μM). HIF-1α levels were quantified by densitometry, normalized to the β-actin loading control; cells were treated with CP-PROTAC and incubated in normoxia for 1 h, followed by incubation in hypoxia for 6 h; All data are mean (n = 3) ± SD.

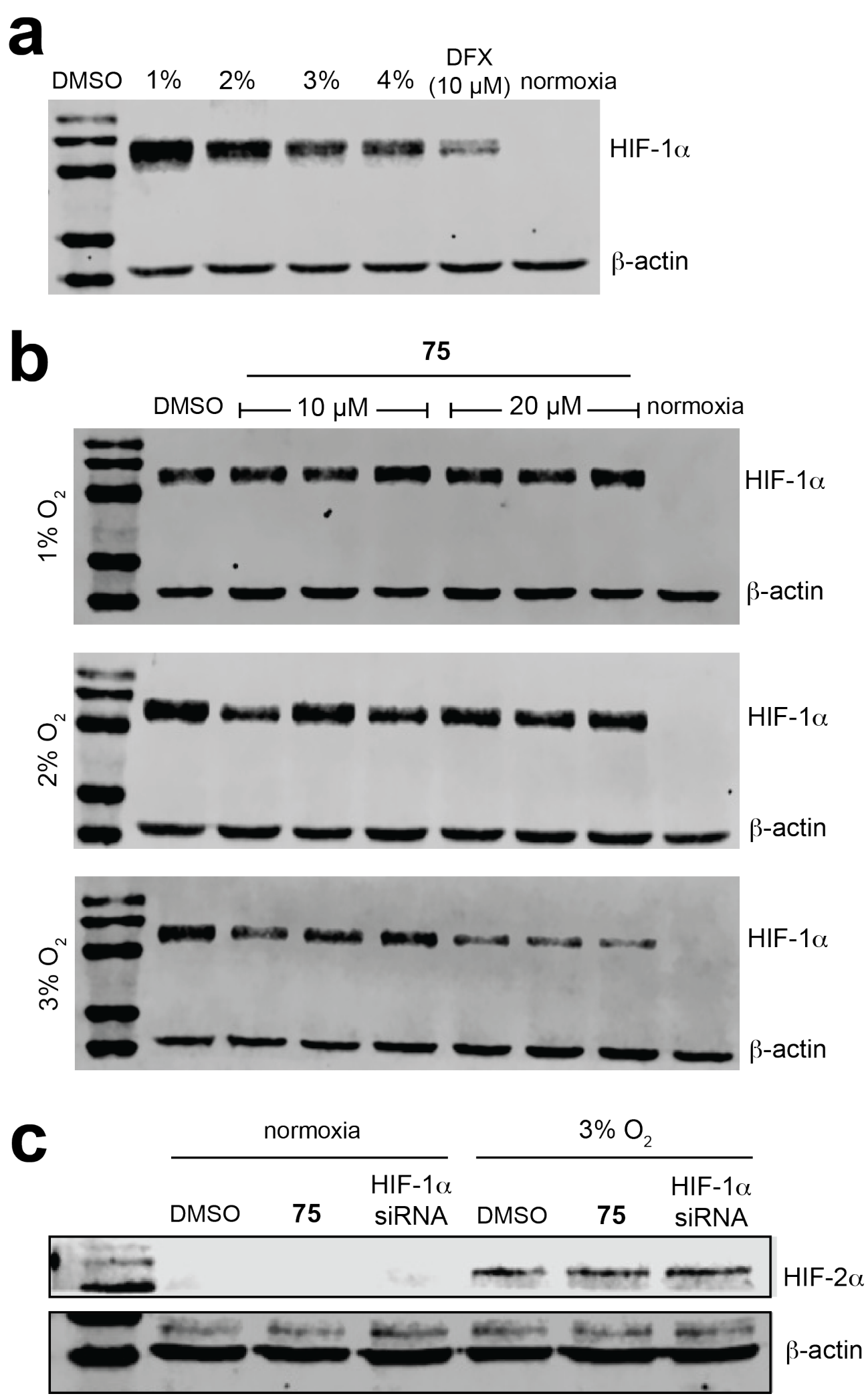

Figure S11. The effect of 75 on HCT116 cells in hypoxia. a) Western blot of HIF-1α at O2 levels from 1% to 4%, DFX (10 μM), and normoxia. **b)** The effect of **75** on HIF-1α at 1%, 2% and 3% O_2. **c)** The effect of **75** and siRNA to HIF-1α on HIF-2α at 3% O_2.

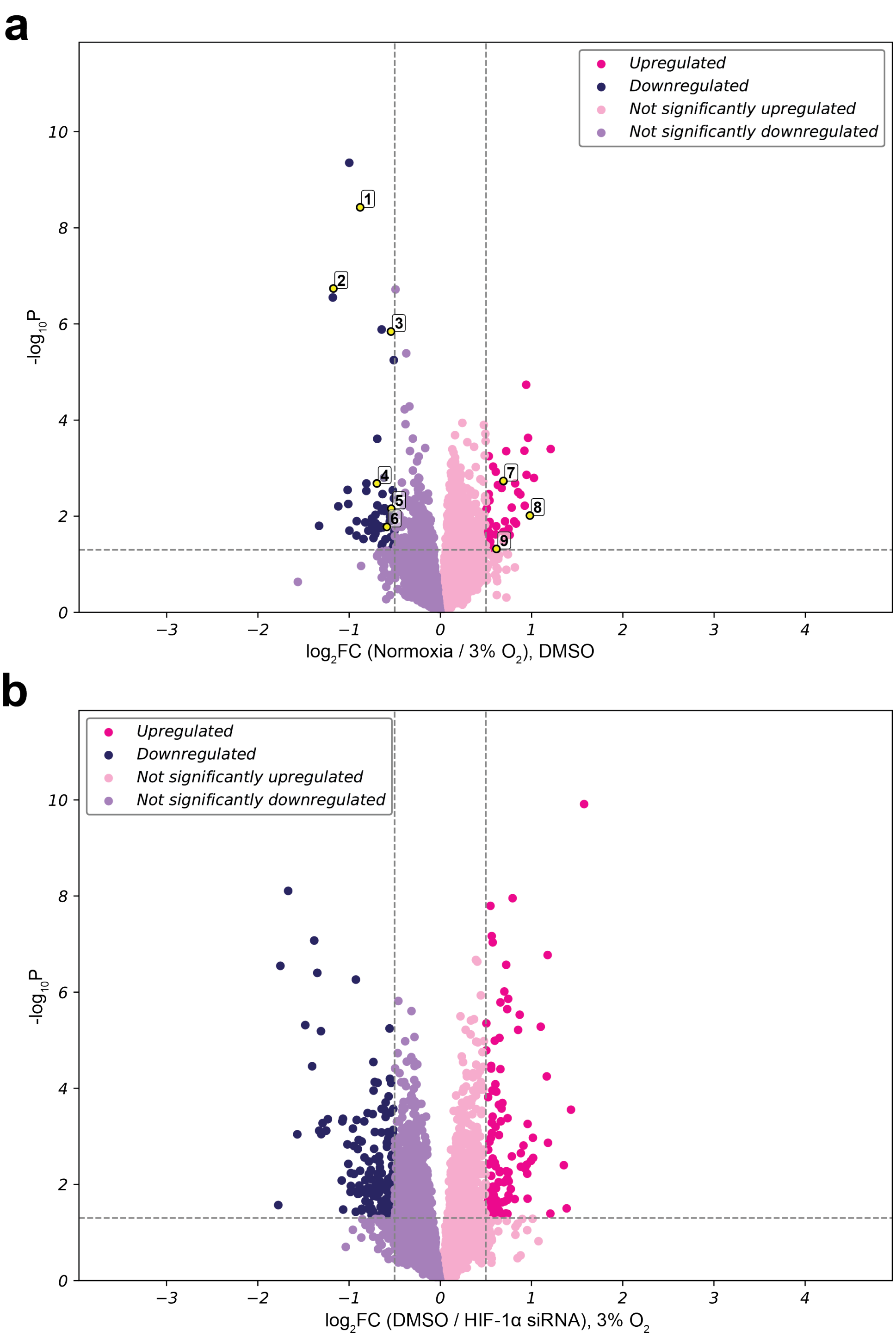

Figure S12. Whole cell proteomic analysis. a) HCT116 cells in normoxia vs. 3% O_2; proteins also present in a published dataset for HCT116 cells in 1% O_2 are highlighted in yellow: 1. Ferritin heavy chain (P02794), 2. Ferritin light chain (P02792), 3. Lysine-specific demethylase 4B (O94953), 4. Tripartite motif-containing protein 59 (Q8IWR1), 5. Glutaredoxin-1 (P35754), 6. Cyclin-dependent kinase 2-associated protein 1 (O14519), 7. Splicing regulator SDE2 (Q6IQ49), 8. Mitochondrial inner membrane scaffold 2 (Q96KR6), 9. CCHC-type zinc finger nucleic acid binding protein (P62633). **b)** HCT116 cells in 3% O_2 vs. cells treated with siRNA to HIF-1α. For all data upregulation log2FC>0.5, downregulation log2FC <-0.5, p values <0.05 in each case)

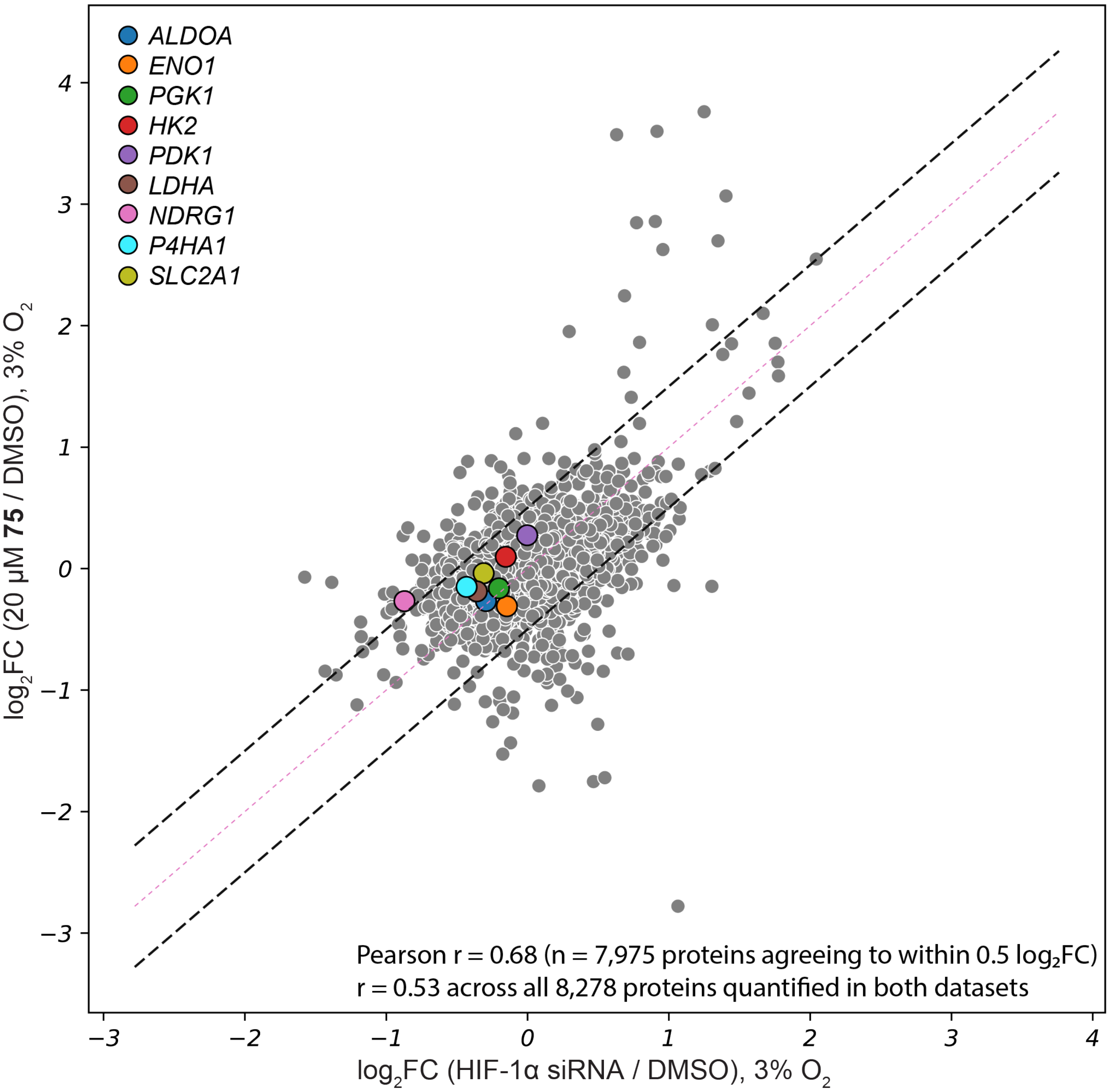

Figure S13. Correlation of proteome-wide changes induced by CP-PROTAC 75 and HIF-1α siRNA. Log_2 fold-changes in protein abundance induced by 20 µM 75 (y-axis) plotted against those induced by HIF-1α siRNA (x-axis), both relative to DMSO-treated cells, at 3% O_2. Grey points are all proteins quantified in both datasets; coloured points are exemplar canonical HIF-1 target proteins (at 1% O_2) as indicated. The black dashed lines indicate agreement between the two datasets to within 0.5 log_2FC, the quality threshold applied throughout this study. Pearson r = 0.68 (n = 7,975 proteins agreeing to within 0.5 log_2FC); across all 8,278 proteins quantified in both datasets, r = 0.53.

<u>Material and Methods</u>

All reagents were purchased from Fisher Scientific or Sigma Aldrich UK unless specified otherwise. Restriction enzymes and microbiology reagents were purchased from New England Biolabs Inc., FisherScientific (UK) or Promega (UK) and were used as directed by the manufacturer. Primers and oligonucleotides were synthesised by Integrated DNA Technologies (UK). DNA sequencing was carried out by Eurofins MWG Operon (Germany).

All the cells used in this study were cultures in DMEM medium with 10% FBS and incubated in humidified atmosphere with 5% CO2 unless stated otherwise. For studies in hypoxia, cells were incubated in a Don Whitley DW35 hypoxia incubator; isolation of mRNA and protein for these experiments was carried out in a hypoxic environment.

Synthetic Chemistry

<u>2-Chlorotrityl Chloride Resin Loading</u>

Peptides were synthesised by Fmoc solid-phase peptide synthesis using 2-Chlorotrityl chloride (2-CTC) resin. The first amino acid was loaded onto the 2-CTC (1 eq) by swelling the resin in DCM in a sinter funnel followed by the addition of Fmoc amino acid (3 eq) dissolved in DCM and DIPEA (3 eq). The resin was agitated via argon flow for 2 hours. MeOH (5 mL) was then added to the funnel to cap any unreacted resin and agitated for an additional 15 minutes. The reaction mixture was then removed under reduced pressure and the resin washed with DMF, DCM and Et_2O. The resin was then air dried and 10 mg was accurately weighed into a glass vial and the Fmoc protection group removed via with 20% Piperidine in DMF. A 100 mL aliquot of the reaction mixture was diluted 1:10 in DMF and the UV absorbance of dibenzofulvene-piperidine adduct was measured at a wavelength of 301 nm using a NanoDrop ND-1000 Spectrophotometer (NanoDrop Technologies, USA). The absorbance value was used in the following equation to determine the loading of the resin in mmol/gram.

L = (A301 x V x d)/(Ec x w x M)

L = Resin loading (mmols/gram)
A301 = Absorbance at 301 nm
V = Volume of the cleavage solution (1 mL)
d = dilution factor (10)
Ec = Extinction coefficient (= 7800mL/mmol.cm)
w = width of the NanoDrop (= 0.1 cm)
M = Weight of the resin sample (10 mg)

<u>Solid Phase Peptide Synthesis on 2-CTC resin</u>

Coupling and deprotection steps were performed at room temperature in 6 mL syringes with a porous a 0.2 mm Frit inside. Reaction mixtures within syringes were placed on a rocker which was slowly agitated from side to side. Coupling solutions were prepared using Fmoc-protected amino acid (3 eq.) and Oxyma Pure (3 eq.) dissolved in DMF, to which DIC (3 eq.) was added. The mixture was stirred for 3 minutes, after which the solution was added to the resin and agitated for 1 h. The resin was washed three times with DMF, then three times with DCM and finally three times with Et_2O. Successful coupling reactions were checked using the Kasier test prior to Fmoc deprotection. Fmoc deprotection was carried out by agitating the resin with 20% piperidine in DMF for 20 mins. The resin was washed as before, and successful deprotections were checked using the Kaiser test prior to moving on to the next coupling. After deprotection of the final residue, the dry resin was transferred

to a 12 mL vial and stirred with 20% HFIP in DCM (10 mL per mmol peptide) for 2 h to cleave the peptide from the resin. The mixture was filtered through a cotton filter, and the filtrate concentrated *in vacuo*. Peptide was precipitated from the remaining residue with cold Et_2O. The Et_2O was removed, and precipitate triturated a further two times with cold Et_2O, after which the solid was dried on a rotary evaporator. Depending on purity (determined by LC-MS), the peptide was either used for subsequent reactions without further purification, or was dissolved in a H_2O:MeCN mixture (1:1) prior to purification by reverse-phase chromatography.

<u>Solid Phase Peptide Synthesis on Wang resin</u>

Peptide synthesis on Wang resin was identical to the 2-CTC resin procedure with the exception of the resin cleavage stage which was done using TFA/TIPS/H_2O (95:2.5:2.5% v/v). The resin was stirred with this mixture for 2 h, followed by the same filtering process for 2-CTC resin and concentrated *in vacuo*. The linear peptide was triturated as described before and purity determined by LC-MS.

<u>Peptide Cyclisation</u>

The linear peptide was dissolved in DMF (1 mL DMF per mg peptide) followed by the addition of DIPEA (3 eq) and HATU (1.5 eq) and the reaction was left to stir at room temperature overnight. After reaction completion, the mixture was concentrated on a rotary evaporator and the remaining residue purified by reverse-phase chromatography. The fractions were checked via LC MS and pooled together and concentrated *in vacuo*.

<u>TFA Deprotection</u>

Acid labile protecting groups on cyclic peptides were dissolved in a TFA cleavage cocktail TFA/TIPS/H_2O (95:2.5:2.5% v/v) and stirred at ambient temperature for 1 hour. Excess TFA was then removed under reduced pressure and purified by reverse phase chromatography.

<u>Cys(S^tBu) deprotection</u>

Cys(S^tBu) containing peptide sequences were deprotected by dissolving the cyclic peptide in 1 mL DMF followed by the addition of DTT (30 eq) and $(NH_4)_2CO_3$ (30 eq) the mixture was then stirred for 1 h at ambient temperature. The mixture was then filtered through a 0.22 uM PTFE syringe filter and purified via reverse-phase HPLC.

<u>Biotage Chromatography</u>

Purification was performed on a Biotage-Isolera Spektra Four Purification system using a SFAR 30g D DUO Column for reverse phase chromatography. A binary solvent elution system of 0.1% TFA/H_2O (Solution A) and 0.1%TFA/MeCN (Solution B) was used to equilibrate the column at 90% Solution A and 10% Solution B for 3 column volumes at 25 $mLmin^{-1}$. Compounds were dissolved in either 2 mL of DMF or MeCN and injected directly onto the column. A series of gradients were set according to the results of the LC/MS of the crude material. Fractions were collected when UV 220nm exceeded a 40 mAu threshold. Fractions were analysed via LC/MS, desired fractions were then pooled, concentrated *in vacuo,* and lyophilised to yield the desired product.

<u>Preparative HPLCs</u>

Samples were injected as solutions of either DMF or MeCN via a Waters Flex inject system into a RP-HPLC system consisting of a Waters 1525 binary pump coupled to a Waters 2998 Chapter 8 133

photodiode array detector. Cyclic peptides were then purified using a Waters Atlantis T3 column (5.0 µm particle size, 19 × 100 mm). A binary solvent system consisting of 0.1 % TFA / waterH_2O, (Solution A) 0.1 % TFA / MeCN (Solution B) was ran at gradients according to their retention time determined by analysis via LC/MS of the crude. Fractions with an absorbance of 220 nM absorbance were collected, analysed via LC/MS, desired fractions pooled, concentrated *in vacuo,* and lyophilised to yield the desired product.

Nuclear magnetic resonance

Nuclear magnetic resonance (NMR) spectra were obtained from Brucker AV-400 spectrometers. Chemical shifts (d) for ^{1}H NMR spectra are recorded in parts per million from tetramethylsilane with the deuterated solvent resonance as the internal standard ($CDCl_3$: 7.26 ppm, MeOD: 3.31 ppm, DMSO: 2.50 ppm). Data were reported as follows: chemical shift (ppm) [multiplicity (s = singlet, d = doublet, t = triplet, m = multiplet and br = broad), coupling constant (Hz), integration and assignment]. ^{13}C NMR spectra were recorded with complete proton decoupling. Chemical shifts are reported in parts per millions from tetramethylsilane with the solvent resonance as the internal standard ($^{13}CDCl_3$: 77.0 ppm, MeOD: ppm, DMSO: ppm). *J* values are reported in Hz. Chemical shifts are reported in parts per million from tetramethylsilane. Assignments were made using a range of NMR experiments when required.

Electrospray LC/MS Analysis

Samples were analysed using a Waters (Manchester, UK) TQD mass spectrometer equipped with a triple quadrupole analyser. Samples were introduced to the mass spectrometer via an Acquity HClass quaternary solvent manager (with TUV detector at 254nm, sample and column manager). Ultra-performance liquid chromatography was undertaken via a Waters BEH C18 column (50 mm x 2.1mm 1.7µm). Gradient 20% acetonitrile (0.2% formic acid) to 100% acetonitrile (0.2% formic acid) in five minutes at a flow rate of 0.6 mL/min. Low-resolution mass spectra were recorded using positive ion electrospray ionisation.

High Resolution Mass Spectrometry

Samples were analysed using a MaXis (Bruker Daltonics, Bremen, Germany) mass spectrometer equipped with a Time of Flight (TOF) analyser. Samples were introduced to the mass spectrometer via a Dionex Ultimate 3000 autosampler and uHPLC pump. Gradient 20% acetonitrile (0.2% formic acid) to 100% acetonitrile (0.2% formic acid) in five minutes at 0.6 mL/ min. Column, Acquity UPLC BEH C18 (Waters) 1.7-micron 50 x 2.1 mm. High resolution mass spectra were recorded using positive ion electrospray ionisation.

Analytical HPLC

Analytical HPLCs were carried out on an Agilent 1260 Infinity II HPLC system running a binary solvent system consisting of 0.1 % TFA / water, (Solution A) 0.1 % TFA / MeCN (Solution B). Cyclic peptide PROTACs were injected (50 mL) onto a Poroshell 120 EC-C18 column (2.7 µm particle size, 3.0 x 100mm) and the gradient shown below ran at a flow rate of 0.625 mL/ min. UV absorbance was recorded at both 220 and 280 nm. Peaks detected at 220 UV absorbance were integrated to determine compound purity.

***in vitro* and cell-based assays**

HIF-1α PAS-B domain expression, purification and labelling for MST

The HIF-1α PAS-B domain was expressed and purified as previously described *(*Ball *et al.,* J. Am. Chem. Soc., 2024, 146(13); 8877-8886).

HIF-1α PAS-B MST

The binding of molecules to HIF-1α PAS-B domain was assessed by MST as previously described *(*Ball *et al.,* J. Am. Chem. Soc., 2024, 146(13); 8877-8886).

CP PROTAC-HIF-1α-VCB ternary complex MST

The formation of a ternary complex between CP PROTAC, HIF-1α and VHL complex was measured by MST. Recombinant HIF-1α protein was fluorescently labelled according to the manufacturer's protocol and diluted to a final concentration of 20 nM in MST HIF-1α buffer. Labelled HIF-1α was pre-incubated with 50 nM human VHL–Elongin B/C complex (VCB, Abcam, ab271497) for 30 min at room temperature. CP PROTAC was solubilised in DMSO, and all dilutions were prepared such that the final DMSO concentration in each MST sample was 2%. A 12-point serial dilution of the PROTAC was generated over a concentration range of 17 nM to 100 µM. Each dilution was mixed 1:1 with the pre-incubated HIF-1α/VCB complex to maintain a constant final concentration of labelled protein across all samples. Samples were incubated for 15 min at room temperature to allow equilibrium binding before loading into premium-coated MST capillaries (NanoTemper Technologies).
MST measurements were performed on a Monolith NT.115 instrument at 25 °C using 40% LED power and medium MST power. Thermophoresis traces were analysed using MO. Affinity Analysis software, and normalised fluorescence (Fnorm) values were exported to GraphPad Prism for curve fitting. All measurements were performed in triplicates.

Enzyme-Linked Immunosorbent Assay

To each well of a clear Pierce nickel coated 96-well plate (Thermo Scientific), purified His_6-HIF-1α PAS-B protein in HIF buffer was added (25 µL, 0.1 µM), the plate was then incubated for 1 hr at RT with rocking. The wells were then washed 2 x with HIF buffer (150 µL) and 1 x with PBS Tween (0.05 % Tween 20, 150 µL). 2 % milk in PBS Tween (150 µL) was then added to each well and incubated for 1 hr at RT with rocking as a blocking step. The wells were then washed 2 x PBS Tween (150 µL) and 1 x with HIF buffer (150 µL). A serial dilution of HIF-1β PAS-B ranging from 250 µM to 11 nM in HIF buffer (100 µL) was then added to measure IC_{50} of dimerization. A serial dilution of *cyclo*-CLLFVY (15 nM to 500 µM) in 100 % DMSO (10 µL) was added immediately before HIF-1β PAS-B (10 µM, 90 µL) for a 10 % final concentration of DMSO. The plate was then incubated at RT for 1 hr with rocking. The wells were then washed 2 x with HIF buffer (150 µL) and 1 x with PBS Tween (150 µL). A 1:1000 dilution of mouse anti-flag antibody in 2 % milk PBS Tween (50 µL) was then added to each well and the plate incubated for a further hour at RT with rocking. The wells were washed with 3 x PBS Tween (150 µL) with 5 minutes incubation per wash. A 1:6000 dilution of sheep anti mouse linked to horse radish peroxidase in 2 % milk PBS Tween (50 µL) was then added and a further incubation of 1 hr at RT with rocking carried out. The plate was then washed as before and 1-Step TMB Ultra Solution (Thermo Scientific) (100 µL) was added to each well and incubated for 15 minutes. H2SO4 (2M, 100 µL) was then added to each well and the absorbance at 450 nm determined on a BMG Clariostar plate reader.

HIF-1α immunofluorescence experiments

5.0 x 104 HeLa cells were plated on glass coverslips in 35 mm dishes in a final volume of 1.5 mL of DMEM + 10% FBS per well. The cells were then incubated overnight at 37 oC. The media was then removed and the cells were rinsed with cold PBS (1 mL). 1.5 mL of fresh warm media containing CLL-hCou-VY or CLLF(CF3)V-hCou each at 50 µM (1% final DMSO concentration) or media containing 1% DMSO only (as control) was added to the cells. When a treatment with DFX was carried out, DFX was added to the cell media at a final concentration of 100 µM. The cells were subsequently incubated overnight at 37 oC. Following incubation, the media was removed and the cells washed with cold PBS (three times 1mL). The cells were then fixed by incubating them in 1 mL of PBS containing 4% of PFA for 10 minutes at room temperature. The solution was then removed and the cells were washed with PBS (3 x 1 mL) and were subsequently permeabilized using 1 mL of 0.5 % TX-100 in PBS (room temperature for 15 minutes). The permeabilization solution was then removed and the cells rinsed with PBS (3 x 1 mL). 1 mL of blocking buffer (1 % BSA and glycine (22.52 mg/mL) in PBST (PBS + 0.1 % Tween-20)) was then added to each well and the plate was incubated at room temperature for 1 hour. The blocking solution was then removed and 1.5 mL of anti-HIF-1α antibody (NB100-449, Novus biologicals) diluted 1:100 in 1% BSA/PBST was added. The plate was then incubated overnight at 4 oC in a humidified chamber. The primary antibody solution was then removed and the cells washed with PBS (3 x 1 mL with 5 minute incubation on a rocker). 1 mL of a solution of secondary antibody anti-rabbit coupled to Alexa 568 (A11011, Thermofisher) at a 1:1000 dilution in 0.2 % BSA/PBST was then added to each well and the plate was incubated for 1 hour at room temperature. The secondary antibody solution was then removed and the cells washed with PBS (3 x 1mL with 5 minute incubation on a rocker). The slides were then dried in the dark and the coverslips mounted on slides with DAPI-free mounting medium. The slides were sealed with nail polish and stored at -20 oC. Fixed cells were imaged using a Nikon plan apochromat 63x oil-immersion objective on a Leica SP8 inverted scanning confocal microscope. A 561 nm solid-state laser line and PMT detection at 580-700 nm was used for visualisation of the Alexa568 probe, and a wavelength of 405 nm with PMT detection was utilised for visualisation of coumarin.

CP-PROTAC dosing of cells

HCT116 cells were grown in 3cm dishes until they were >70% confluent, using RPMI + 10% FBS media. Cells were treated with CP-PROTAC (final concentration, in 100% DMSO) or with vehicle control (1% DMSO). Cells were incubated in normoxia for 1hour. After this, cells were placed in hypoxia in a Don Whitley Scientific Hypoxia Workstation for 6 hours. After this, cells were lysed and prepared for Western blot in hypoxia as previously detailed *(*Ball *et al.,* J. Am. Chem. Soc., 2024, 146(13); 8877-8886).

HIF Luciferase Reporter Assays

Assays were conducted in previously reported U2OS-HRE-Luciferase (U2OS-HRE-Luc) cells lines (Miranda *et al*., J. Am. Chem. Soc., 2013, 135(28); 10418-10425.). Upon reaching 80 – 90% confluency, U2OS-HRE-Luc were plated (10 000 cells/well) in 96-well plates (Perkin-Elmer) and incubated in cell incubator for 16 h. The cells were treated with cyclic peptide at stipulated concentrations and left to incubate for 2 h in normoxia before being transferred to the Hypoxia Workstation and incubated for 6 h or 16 h. After the allotted incubation time, plates were removed from the Hypoxia Workstation and left to equilibrate to room temperature over 30 minutes. Luciferase activity was determined using the Bright-Glo assay kit (Promega) following the

manufacturer's protocol. Luminescence was measured using a ClarioStar plate reader. The luciferase signal of cells treated with peptide were normalised with untreated cells incubated in hypoxia and normoxia.

qPCR

HCT116 cells were plated on 35 x 10mm culture plates and grown in an incubator till 80-90% confluency. Fresh RPMI 1640 medium (10% FBS) media was applied to cells before treatment with peptide at specified concentrations and incubated in a cell incubator for 2 h. Subsequently, cells were transferred to a hypoxic workstation for 16 h. Total RNA was isolated using the SV Total RNA Isolation System kit (Promega) following the manufacturer's protocol and quantified using a Nanodrop ND-1000 spectrophotometer. Complementary DNA was synthesised using the GoScript Reverse Transcriptase kit (Promega) following the manufacturer's protocol. Quantitative real-time PCRs (qPCR) was performed in triplicate using iTaq Universal SYBR Green Supermix and primers for CA9 (NM_001216.3), 18S (NCBI ID: 106631781) and HPRT1 (NM 012583). Gene expression values were normalised to 18S and HPRT1. Data was plotted and IC_{50} calculated using Prism software with a four-parameter logistic model ([Inhibitor] vs normalised response).

Table 1. Primer sequences used for qPCR experiments

**Gene Name**	**Sequence**
Carbonic Anhydrase 9 (CA9)	Fw: 5'- CAACACGGGAAACCTCACCC -3'<br>Rv: 5'- CCGGGCCCTCCTCCAGAAAG -3'
18S	Fw: 5'- CAACACGGGAAACCTCACCC – 3'<br>Rv: 5' - CTCCACCAACTAAGAACGGCC – 3'
Hypoxanthine Phophoribosyltransferase 1 (HPRT1)	Fw: 5' - CTCATGGACTGATTATGGACAGGAC – 3'<br>Rv: 5' - GCAGGTCAGCAAAGAACTTATAGCC – 3'

Western Blot sample preparation in normoxia

HCT116 cells were plated onto Corning Falcon Easy-Grip Tissue Culture dishes (2.5 mL to 3 mL Volume working) to a volume of 2 mL of RPMI 1640 medium (10% FBS). The cells were incubated in a 5% CO_2 incubator at 37°C until a 80-90% confluency on the plates were reached. The media was removed and 1470 mL of RPMI 1640 medium (10% FBS). was added to the cells. Compounds were dissolved in DMSO to achieve a 10 mM stock solution which was diluted appropriately to give the stock concentration that would achieve the desired concentration for cell treatment following a 1:100 dilution in the RPMI 1640 medium (10% FBS) mixture. 15 mL of DFX (1mM (10 mM) dissolved in water) was added to the plates and incubated for 1 hr at 37 °C. The plates were then supplemented with 15 mL of compound and the plates transferred back to the incubator and incubated for the stipulated time frame. The media was removed, and the plates washed with DPBS thrice to remove excess RPMI 1640 medium (10% FBS) and dead cells. The plates were then treated with 100 mL ice cold lysis buffer and left to incubate on ice for 10 minutes. The plates were then scrapped using a cell scrapper and the lysate collected in 2 mL Eppendorf tubes. The cell lysate was sonicated six times for 20 seconds with 30 seconds break between each sonication on ice. The lysate was then centrifuged for 20 minutes at 10,000 rpm and the supernatant transferred to 0.2 mL PCR tubes. 50 mL of each sample was transferred to a fresh 0.2 mL PCR tube with 50 mL of 2x Laemmli buffer which were transferred to a PCR light cycler and the protein lysate was denatured at 95 °C for 10 minutes. The denatured cell lysates were stored at -20 °C until required.

Western Blot sample preparation in hypoxic chamber

HCT116 cells were plated onto Corning Falcon Easy-Grip Tissue Culture dishes (2.5 mL to 3 mL Volume working) to a volume of 2 mL of RPMI 1640 medium (10% FBS). The cells were incubated in a 5% CO2 incubator at 37 °C until a 80-90% confluency on the plates were reached. The media was removed and 1485 mL of DMEM (10% FBS) was added to the cells. Compounds were dissolved in DMSO to achieve a 10 mM stock solution which was diluted appropriately to give the stock concentration that would achieve the desired concentration for cell treatment following a 1:100 dilution in the DMEM mixture. The plates were then incubated with 15 mL of the compounds and left in an incubator at 37 °C for 1 h. Following incubation, the plates were transferred to a hypoxia station unit and incubated for the stipulated time frames. In the hypoxia station unit, the media was removed, and the plates washed with DPBS thrice to remove excess RPMI 1640 medium (10% FBS) and dead cells. The plates were then treated with ice cold lysis buffer (100 mL) and left to incubate on ice for 10 minutes. The plates were then scrapped using a cell scrapper and the lysate collected in 2 mL Eppendorf tubes. The cell lysate was sonicated six times for 20 seconds with 30 seconds break between each sonication on ice. The lysate was then centrifuged for 20 minutes at 10,000 rpm and the supernatant transferred to 0.2 mL PCR tubes. 50 mL of each sample was transferred to a fresh 0.2 mL PCR tube with 50 mL of 2x Laemmli buffer which were transferred to a PCR light cycler and the protein lysate was denatured at 95 °C for 10 minutes. The denatured cell lysates were stored at -20 °C until required.

Preparation of samples for proteomics

Whole cell lysates were prepared for proteomics analysis and sent to the University of Edinburgh for analysis. $8x10^5$ HCT116 cells were plated per 35mm dish, in RPMI + 10% FBS and left to settle overnight. 4 repeats of each condition were plated: Normoxia, Hypoxia, Normoxia + siRNA, Hypoxia + siRNA, Normoxia + PROTAC, Hypoxia + PROTAC. Assay Id s6539 Silencer Select Validated siRNA, 1 nmol_ [933294340_55984126]. siRNA was transfected into target cells at a final concentration of 5nM, using Lipofectamine RNAiMAX Transfection Reagent (ThermoFisher, 13778150), following manufacturer instructions. HCT116 cells were treated with PROTAC as described in “Western Blot sample preparation in hypoxic chamber”, at a final concentration of 20µM. Cell incubation was carried out at 3% hypoxia, or normoxia as appropriate. Following the 6 hour incubation of samples, PBS was used wash cells twice. Then, cells were scraped off plates using PBS and transferred into an Eppendorf tube. Cells were then spun at 10,000 rpm for 5 minutes to collect them and the PBS supernatant was removed. Cells were snap frozen on dry ice and stored at -80°C before being sent to the Mass Spectrometry Facility at the University of Edinburgh for further analysis.
Protein lysate concentration was quantified by Pierce BCA Protein assay kit (Thermofisher) following manufacturer’s instructions.

Proteomics data acquisition and analysis

Mass spectrometry acquisition and processing was conducted by the University of Edinburgh proteomics facility. Four biological replicates were analysed for each of six conditions: normoxia and 3% O_2, each treated with vehicle (DMSO), CP-PROTAC **75** (20 µM) or HIF-1α siRNA. A total of 10,018 protein groups were quantified across the dataset. Protein groups sharing peptide evidence are reported as semicolon-delimited accession lists and were treated as a single entry throughout. Entries corresponding to common laboratory contaminants (cRAP) were excluded from all analyses.

Differential abundance was assessed using DEqMS, which applies a variance model that accounts for the number of peptides quantified per protein. Proteins were considered differentially abundant between two conditions if they satisfied both of two criteria; a fold-change criterion: $|\log_2$ fold change$|$ > 0.5 with $p < 0.05$ by DEqMS and a presence/absence criterion. This applied to proteins that could not be assigned a fold change because they were undetected in one condition: proteins detected in zero of four replicates of one condition and in at least two of four replicates of the comparator were counted as decreased or increased in the first condition respectively. Proteins meeting either criterion in a given direction were combined to give the totals reported, and the two criteria contributed disjoint sets of proteins.

Western Blot analysis

30 mg of denatured protein lysate was loaded onto 8% or 10% SDS-PAGE gels and ran at 80 V for 80 mins. Separated protein was then transferred to a nitrocellulose membrane using a Trans-Blot Turbo Transfer system (Bio-Rad) according to the manufacturer's instructions. The nitrocellulose membrane was blocked by incubation with 5% powdered milk in PBS and 0.1% Tween for 1 h whilst agitating on a see-saw rocker. Primary antibody solutions were made up according to table and placed in sterile 50 mL falcon tubes. Nitrocellulose membranes were then transferred to the primary antibody mixture; Anti-HIF-1α antibody (1:1000 - HIF-1α (D1S7W) XP® Rabbit mAb #36169, Cell signalling) and anti-beta actin (1:10 000 β-Actin (8H10D10) Mouse mAb #3700, Cell signalling) in 5% BSA/PBS 0.1% Tween-20 buffer and incubated overnight at 4 °C on rollers. Following incubation, the membranes were transferred to clean petri dishes and washed with PBS 0.1% Tween-20 buffer thrice with each wash lasting 5 minutes. The membranes were then incubated with a secondary antibody mixture IRDye 800CW Goat anti-Rabbit IgG (H + L) (1:20 000, LICOR) and IRDye 680RD Goat anti-mouse IgG (H + L) (1:20 000, LICOR) in 5% milk/PBS 0.1 % Tween-20 buffer. The membranes were then washed with PBS 0.1% Tween-20 thrice with each wash lasting 5 minutes. The protein bands were visualised on an Odyssey XF imaging system (LI-COR). The band intensity was analysed using Empiria Studio 3.0 and quantified by normalizing to β-actin. Fold change in HIF-1α levels were plotted on Prism with DC_{50} calculated using linear regression.

Cycloheximide chase

HCT116 cells were grown in 3cm dishes until they were >70% confluent, using RPMI + 10% FBS media. At the start of the experiment, cells were treated with PROTAC (in 100% DMSO) or with vehicle control (1% DMSO). Cells were then left to incubate in normoxia for 1hour. After 1 hour, cells were placed in hypoxia, 1%, for 1 or 6 hours. Cycloheximide, CHX (Merck Chemicals) was weighed out and freshly dissolved in an appropriate amount of sterile water prior to dosing cells. Cells were then treated with 10ug/ml CHX solution for 30 minutes. After the 30 minute incubation, cells were lysed in a hypoxia workstation on ice, sonicated and centrifuged as previously described. Lysates were quantified and prepared for Western Blot as previously described, with 40ug protein loaded in each lane. Mini-PROTEAN TGX (#4561043, BioRad) or SurePAGE™, Bis-Tris, 10x8 (#M00656, GenScript) were used according to manufacturer instructions.

Synthesis and Analytical data for compounds

General protocol for cyclic peptide synthesis

The general protocol above was followed for the synthesis of the cyclic peptides below.

cyclo-CLLFVY (**1**)

The linear resin bound peptide Resin-LCYVFL was synthesised using Wang resin SPPS. The linear peptide was then cleaved from the resin as described in section 8.6.2. The linear peptide was then cyclised using EDC and Oxyma Pure (section 8.6.7), before being purified via reverse-phase flash chromatography (section 8.6.14), to yield the StBu protected cyclic peptide C(StBu)LLFVY. The StBu group was then removed as described in section 8.6.9 and the peptide purified via reverse-phase HPLC (section 8.6.12), like fractions were combined and concentrated under reduced pressure, then lyophilised to yield a white powder (11.8 mg, 8.0 % overall yield).

Analytical HPLC: R_t = 5.290 min (93 % purity).

m/z (ESI^+): 739.8 $[M+H]^+$ (100 %), 1479.2 $[2M+H]^+$ (50 %).

HRMS (ESI^+) found: 739.3833, $[M+H]^+$ calculated: 739.3847.

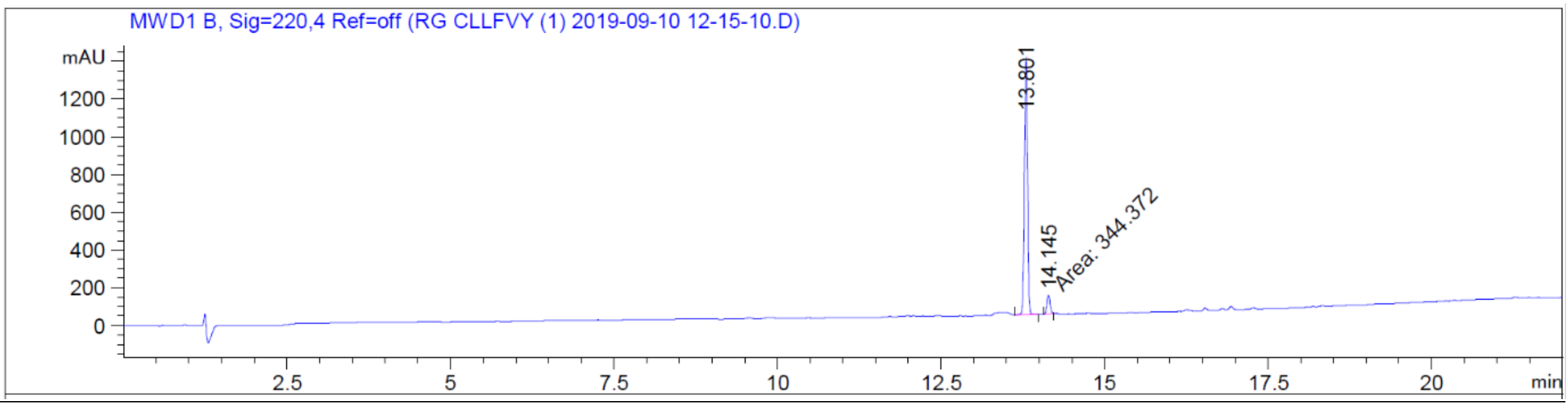

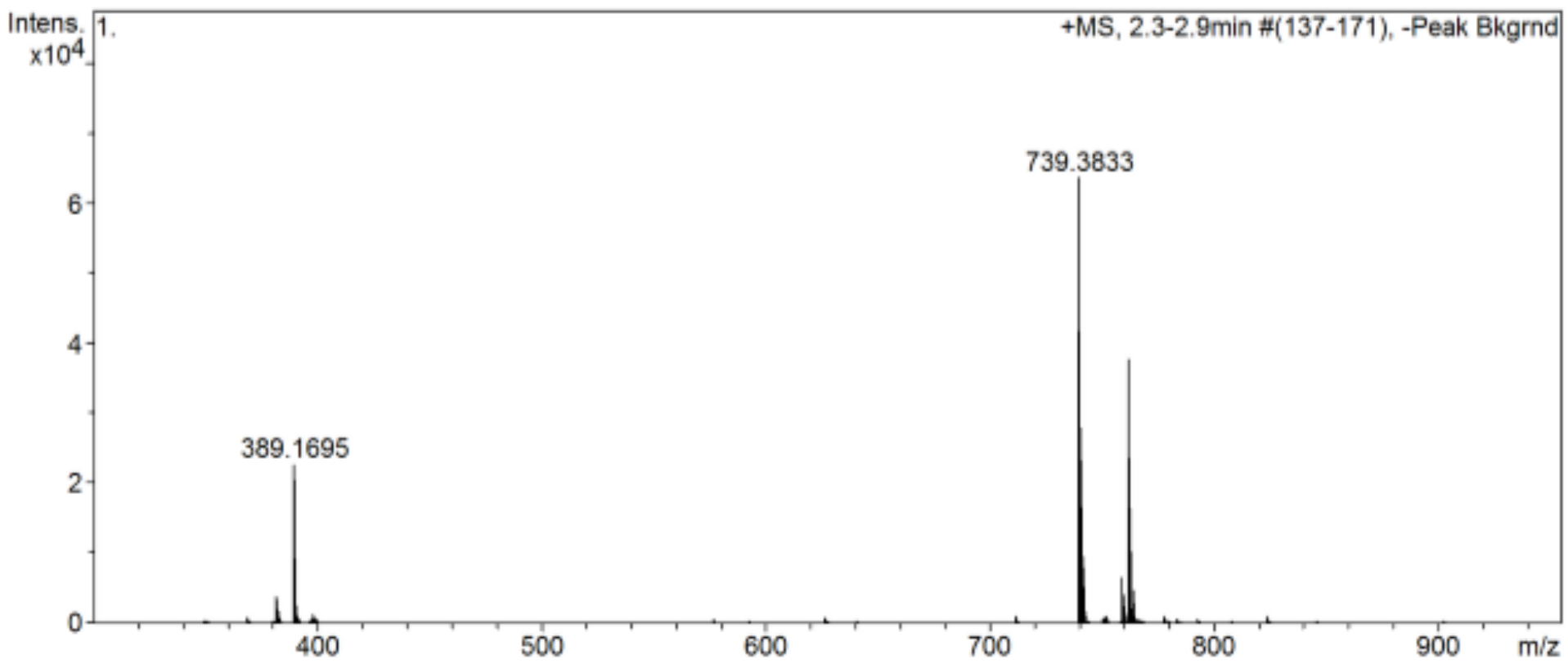

<u>*cyclo*-dCLLFVY (**2**)</u>

cyclo-dCLLFVY was obtained as a white solid (26.4 mg 14.9 % overall yield).

Analytical HPLC: R_t = 14.850 min (97% purity).

m/z (ESI^+): 739.60 $[M+H]^+$(100 %), 1478.91 $[2M+H]^+$ (50 %).

HRMS (ESI^+) found: 738.3773, $[M+H]^+$ calculated: 738.3775.

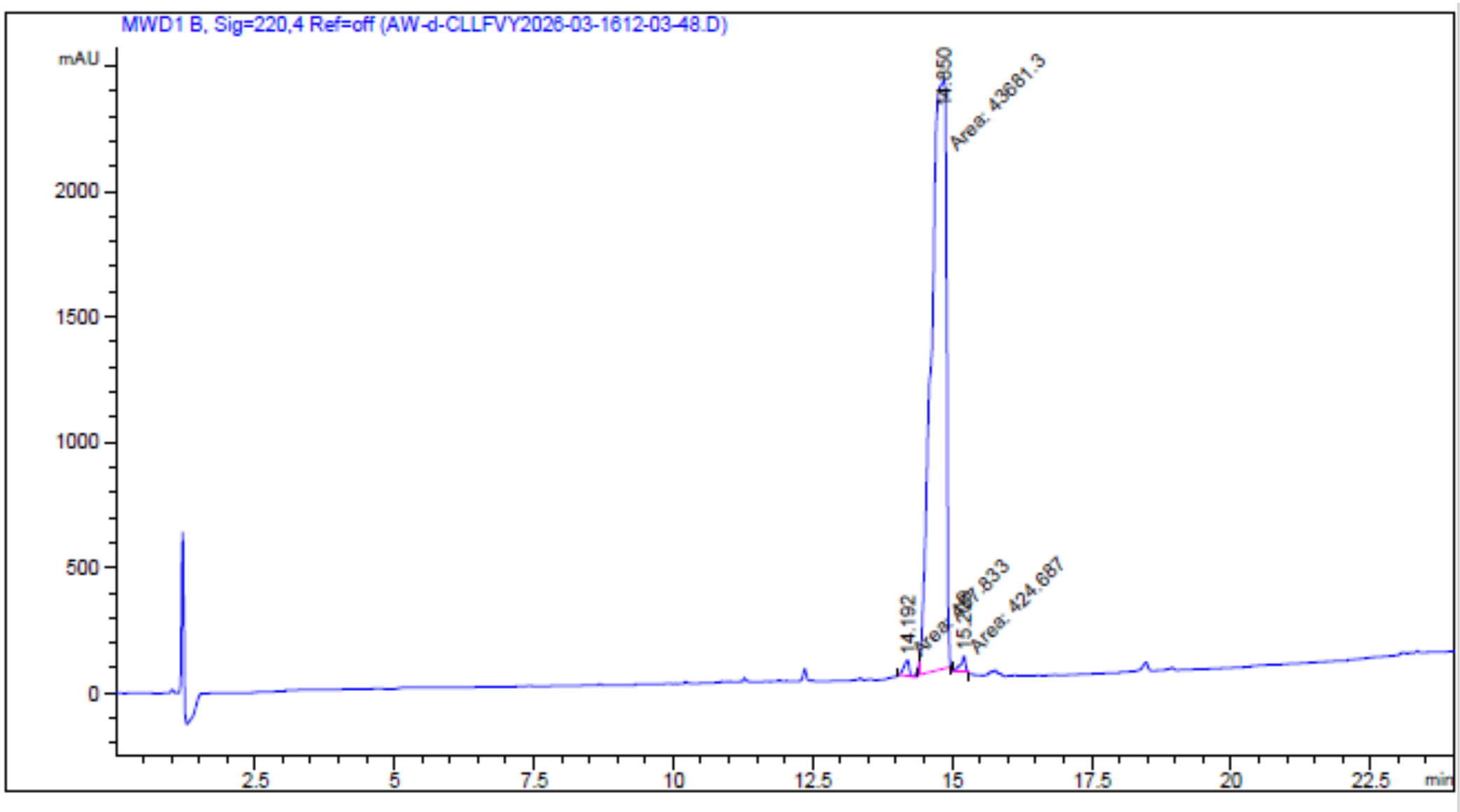

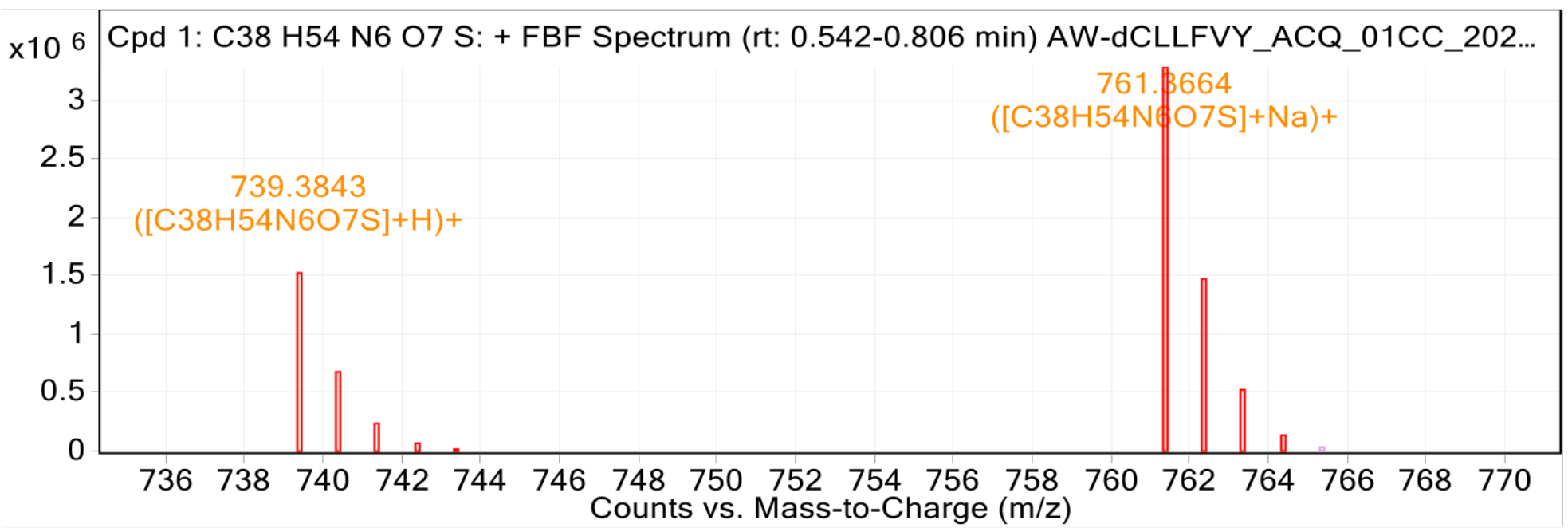

Compound Label	RT (min)	Observed mass (m/z)	Neutral observed mass (Da)	Theoretical mass (Da)	Mass error (ppm)	Isotope match score (%)
Cpd 1: C38 H54 N6 O7 S	0.65	739.3843	738.3773	738.3775	-0.28	98.54

Mass errors of between -5.00 and 5.00 ppm with isotope match scores above 60% are considered confirmation of molecular formulae

cyclo-ALLFVY (**3**)

cyclo-ALLFVY was obtained as a white solid (13.7 mg 13.3 % overall yield).

Analytical HPLC: R_t = 13.177 min (94 % purity).

m/z (ESI^+): 707.7 $[M+H]^+$(100 %), 1437.2 $[2M+H]^+$ (50 %).

HRMS (ESI^+) found: 707.4132, $[M+H]^+$ calculated: 707.4127.

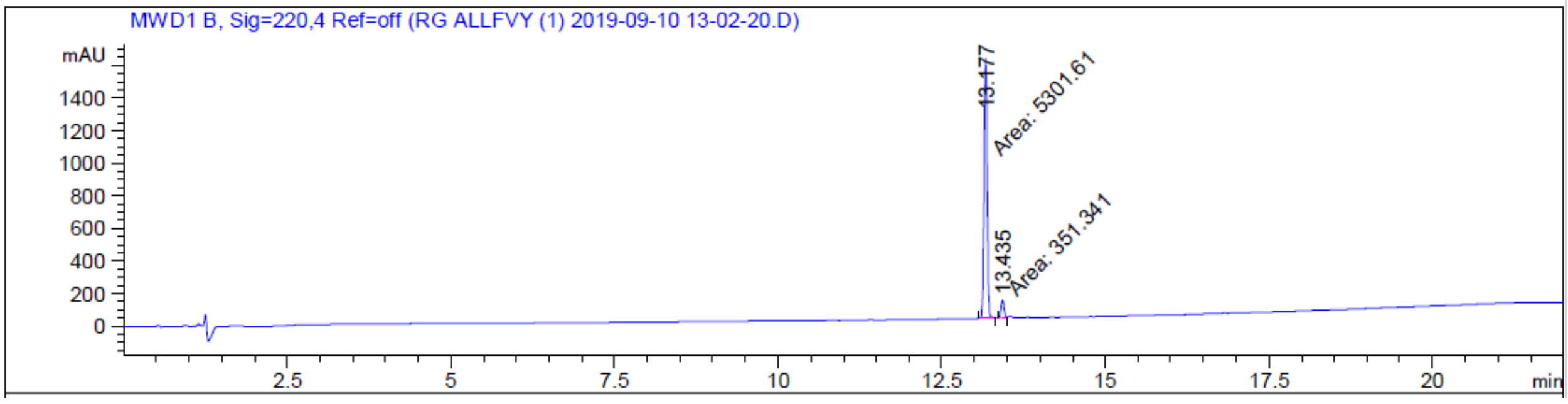

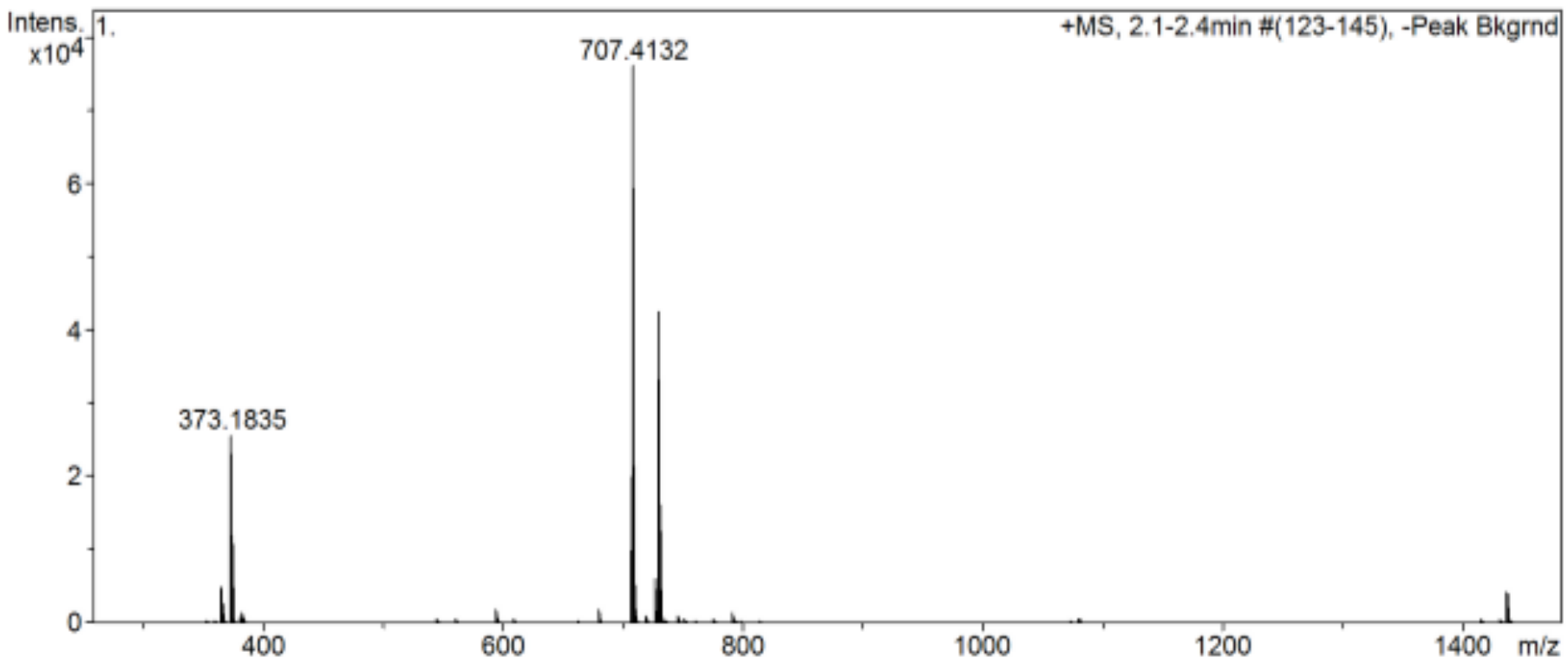

cyclo-MLLFVY (**4**)

cyclo-MLLFVY was obtained as a white solid (20.0 mg 14.0 % overall yield).

Analytical HPLC: R_t = 15.464 min (97 % purity).

m/z (ESI$^+$): 767.63 $[M+H]^+$(100 %).

HRMS (ESI$^+$) found: 766.4085, $[M+H]^+$ calculated: 766.4088.

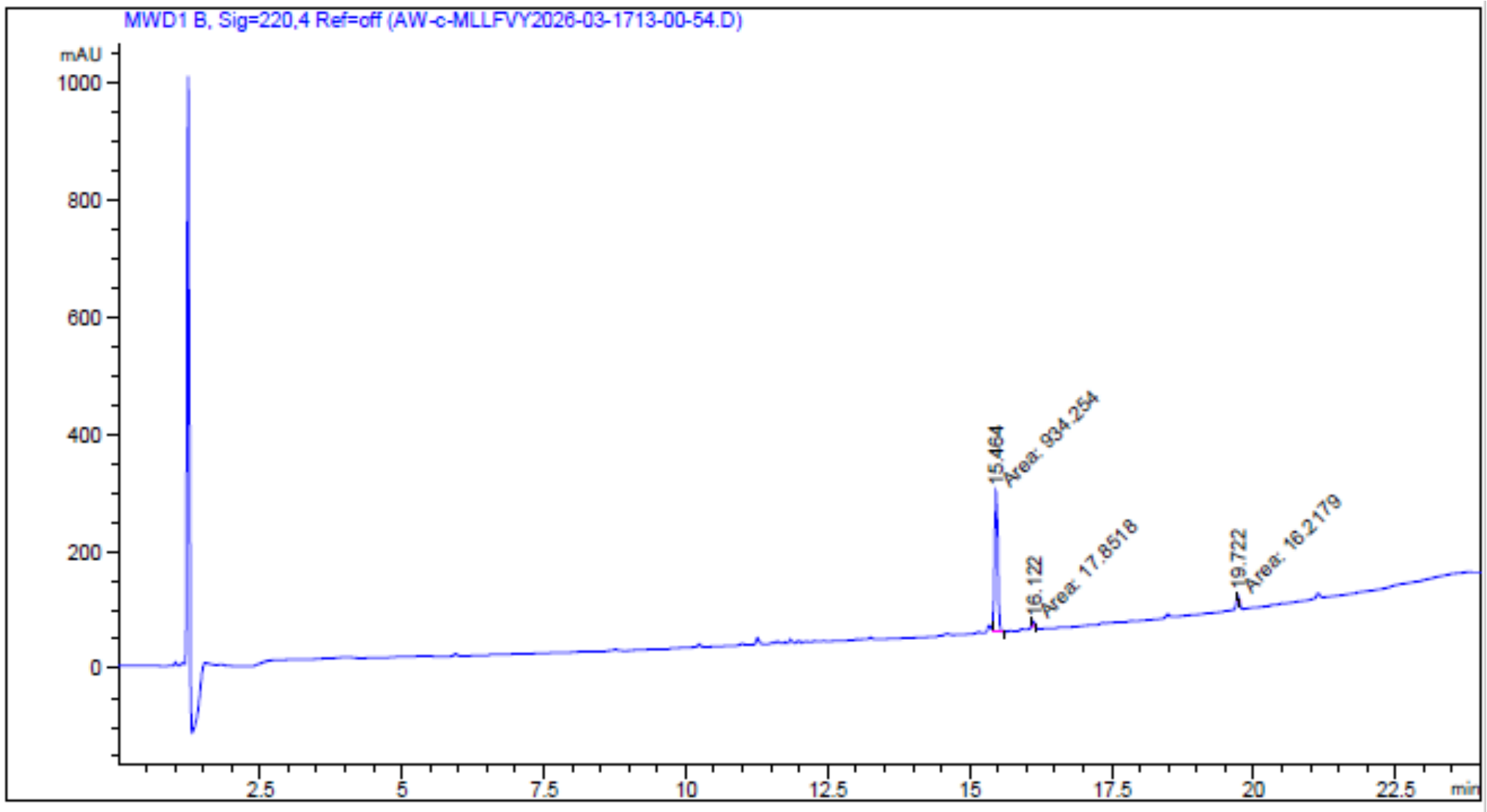

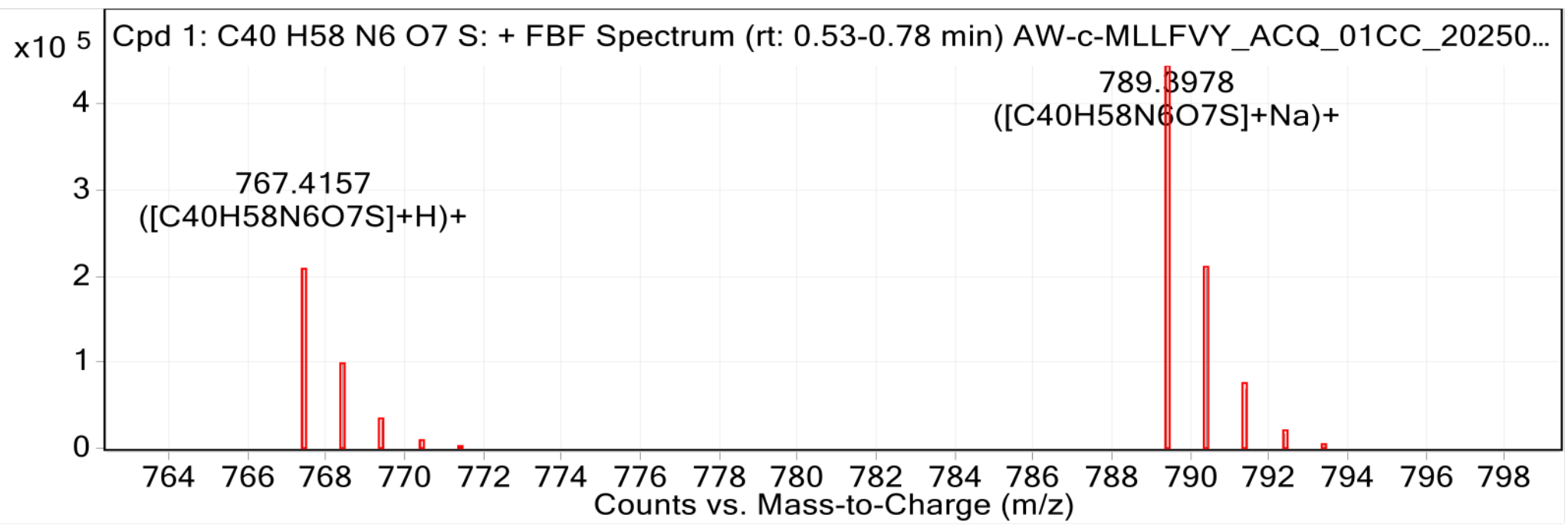

Compound Label	RT (min)	Observed mass (m/z)	Neutral observed mass (Da)	Theoretical mass (Da)	Mass error (ppm)	Isotope match score (%)
Cpd 1: C40 H58 N6 O7 S	0.64	789.3978	766.4085	766.4088	-0.31	99.45

Mass errors of between -5.00 and 5.00 ppm with isotope match scores above 60% are considered confirmation of molecular formulae

<u>*cyclo*-TpLLFVY (**5**)</u>

cyclo-TpLLFVY was obtained as a white solid (39.2 mg 26.2 % overall yield).

Analytical HPLC: R_t = 16.050 min (99 % purity).
m/z (ESI^+): 751.56 $[M+H]^+$(100 %).
HRMS (ESI^+) found: 750.3773, $[M+H]^+$ calculated: 750.3775.

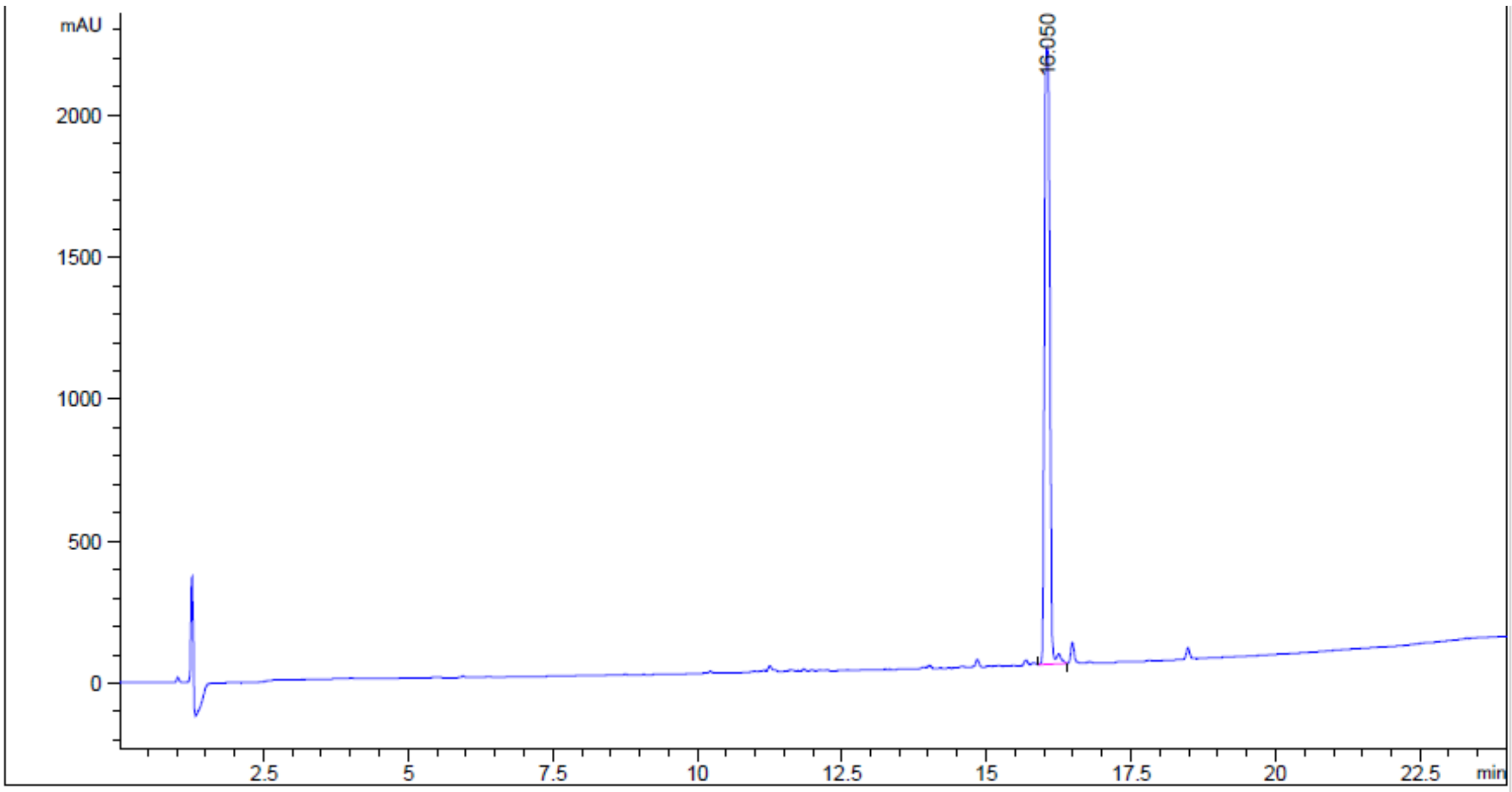

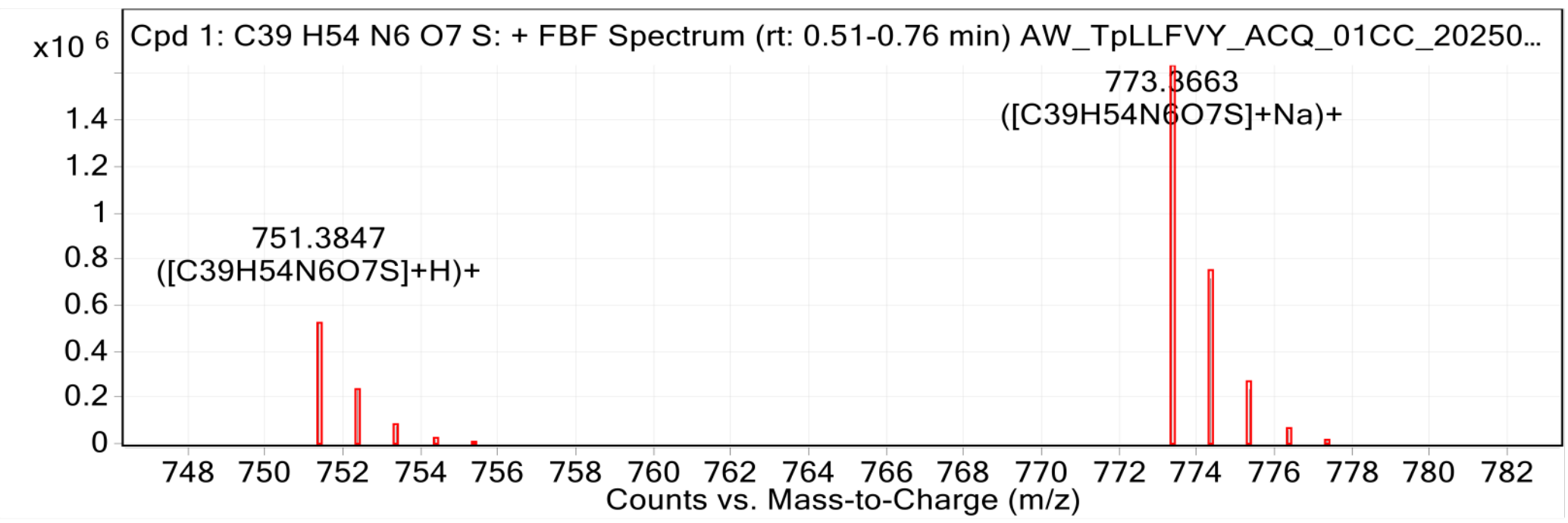

Compound Label	RT (min)	Observed mass (m/z)	Neutral observed mass (Da)	Theoretical mass (Da)	Mass error (ppm)	Isotope match score (%)
Cpd 1: C39 H54 N6 O7 S	0.62	751.3847	750.3773	750.3775	-0.27	99.47

Mass errors of between -5.00 and 5.00 ppm with isotope match scores above 60% are considered confirmation of molecular formulae

cyclo-hCLLFVY (**7**)

cyclo-hCLLFVY was obtained as a white solid (9.9 mg 5.1 % overall yield).

Analytical HPLC: R_t = 15.206 min (91 % purity).

m/z (ESI$^+$): 753.58 [M+H]$^+$(100 %).

HRMS (ESI$^+$) found: 752.3927, [M+H]$^+$ calculated: 752.3931.

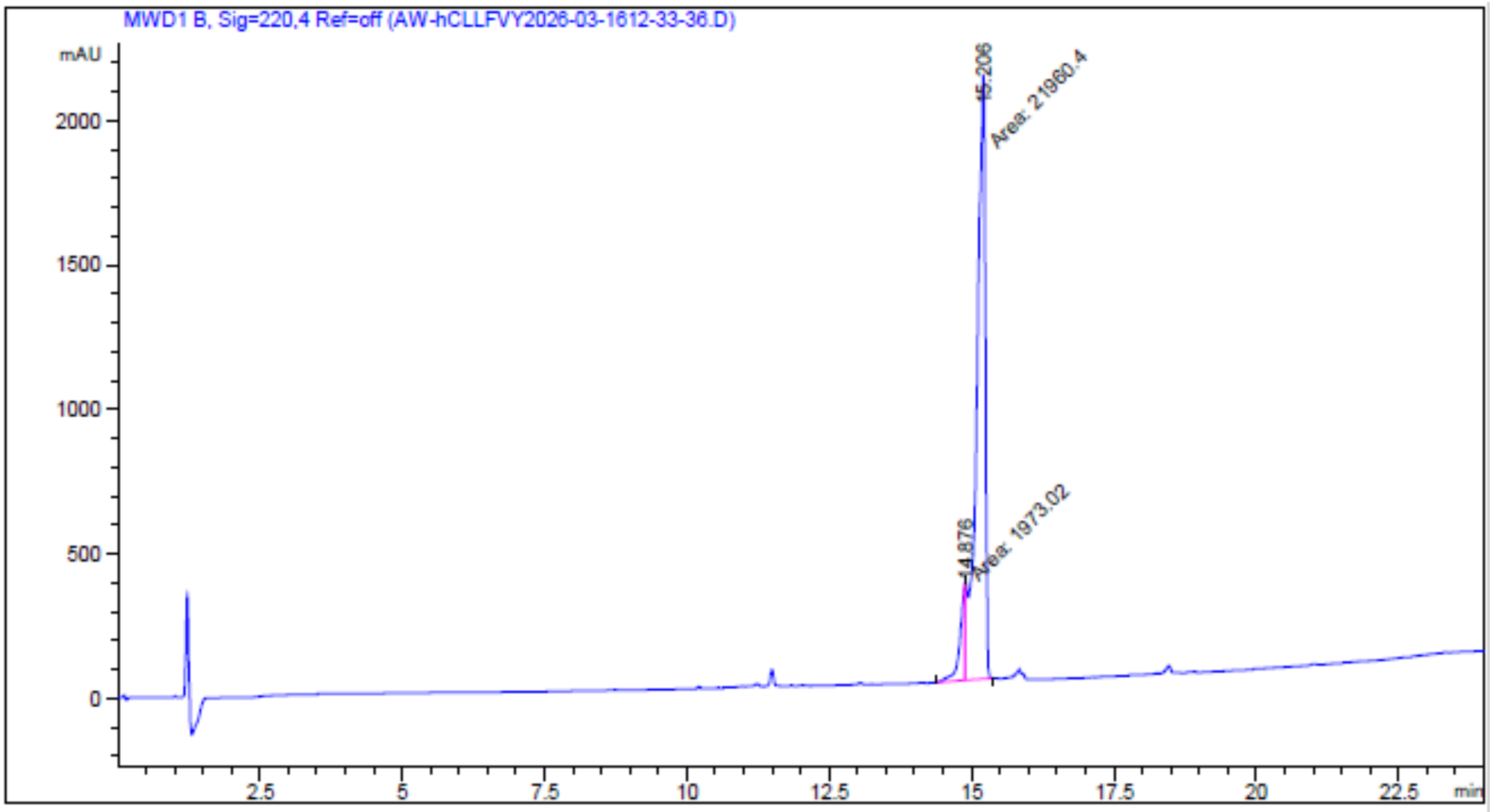

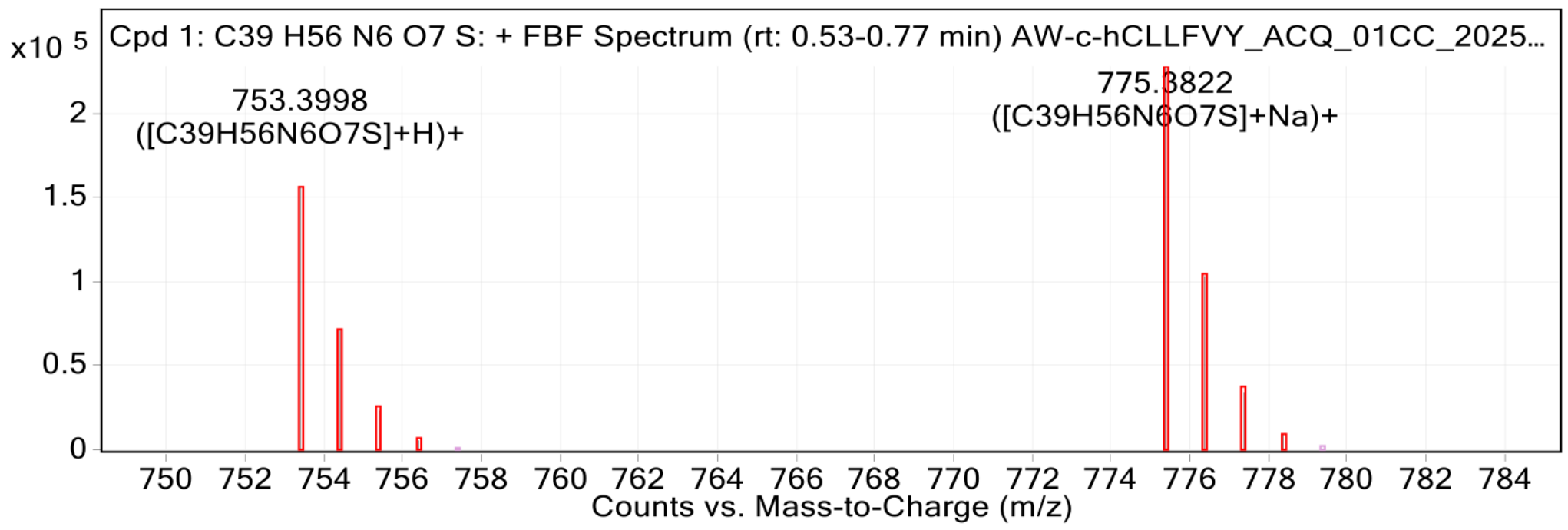

Compound Label	RT (min)	Observed mass (m/z)	Neutral observed mass (Da)	Theoretical mass (Da)	Mass error (ppm)	Isotope match score (%)
Cpd 1: C39 H56 N6 O7 S	0.63	753.3998	752.3927	752.3931	-0.57	99.23

Mass errors of between -5.00 and 5.00 ppm with isotope match scores above 60% are considered confirmation of molecular formulae

cyclo-SLLFVY (**8**)

cyclo-SLLFVY was obtained as a white solid (21.7 mg 16.9 % overall yield).

Analytical HPLC: R_t = 13.261 min (96 % purity).

m/z (ESI^+): 723.60 $[M+H]^+$(100 %), 1446.98 $[2M+H]^+$ (50 %).

HRMS (ESI^+) found: 722.3999, $[M+H]^+$ calculated: 722.4003.

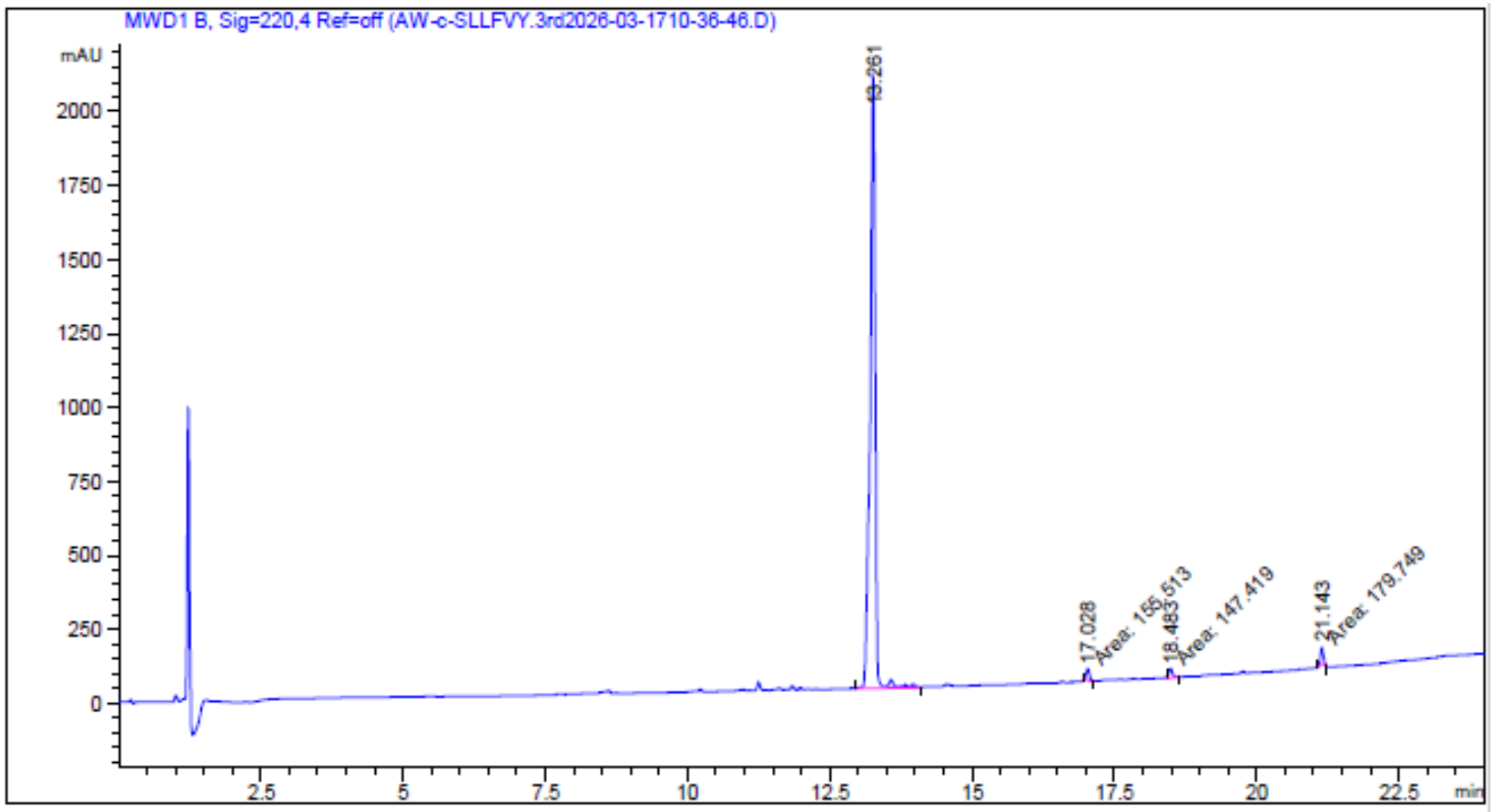

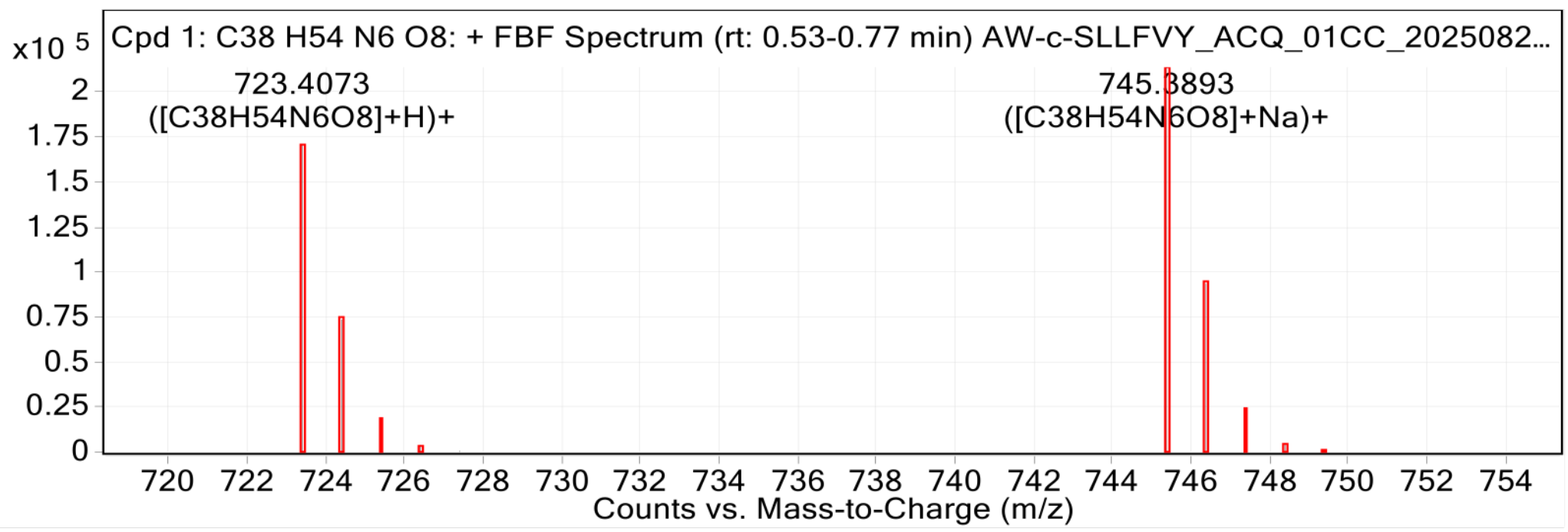

Compound Label	RT (min)	Observed mass (m/z)	Neutral observed mass (Da)	Theoretical mass (Da)	Mass error (ppm)	Isotope match score (%)
Cpd 1: C38 H54 N6 O8	0.63	723.4073	722.3999	722.4003	-0.52	99.69

Mass errors of between -5.00 and 5.00 ppm with isotope match scores above 60% are considered confirmation of molecular formulae

General protocol for *cyclo*-CLLFVY Phenylalanine derivatives

The linear resin bound sequence resin-LLCYV-NH_2 was synthesised using Wang resin SPPS. The resin was then split equally into 10 syringes and swelled in a small amount of DMF. A solution of unnatural amino acid (1 eq.), Oxyma pure (3 eq.) and DIC (3 eq.) was added and the syringes placed on a rocker for 1 hour. The reaction mixtures were then removed via vacuum filtration and the resins washed with DMF x 2, DCM x 2 and Et_2O x 1. Fmoc protected amino acids were then deprotected via the addition of piperidine 20 % in DMF and rocking for 30 minutes. The resins were then washed as before. The linear sequences were then cleaved from the resin and cyclised using HATU, HOAt and DIPEA. The StBu group was then removed and the peptide purified via reverse-phase HPLC. Fractions were combined and concentrated under reduced pressure, then lyophilised to yield the desired cyclic peptides.

cyclo-CLL-hF-VY (**9**)

cyclo-CLL-hF-VY was obtained as a white solid (29.5 mg, 19.3 % overall yield).

Analytical HPLC: R_t = 14.175 min (87 % purity).

m/z (ESI$^+$): 753.7 $[M+H]^+$ (100 %), 1149.6 $[2M+H]^+$ (45 %).

HRMS (ESI$^+$) found: 753.3991, $[M+H]^+$ calculated: 753.4004.

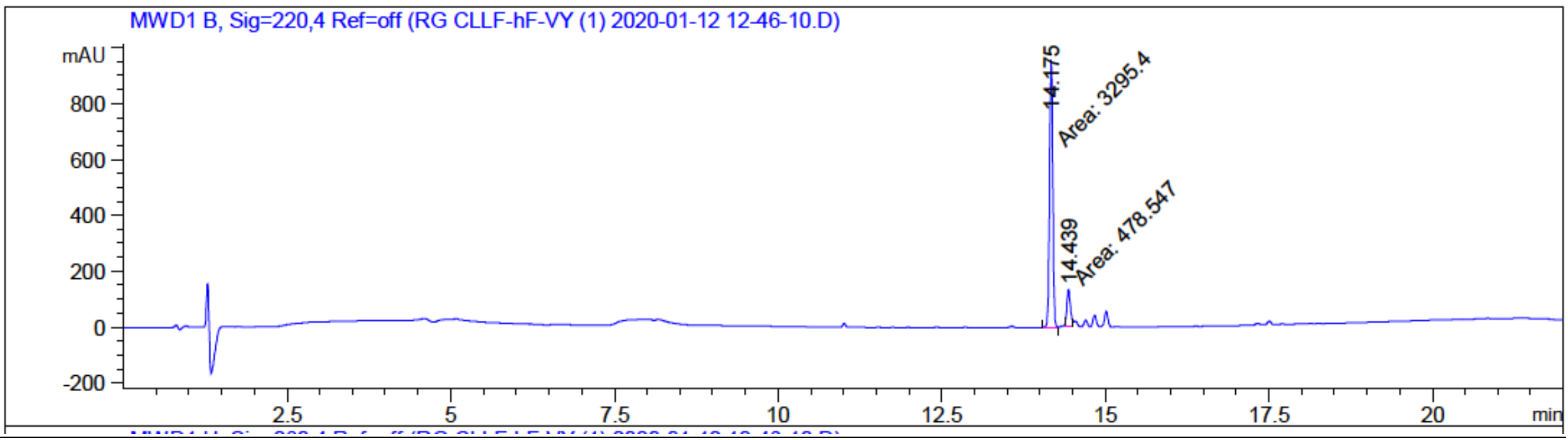

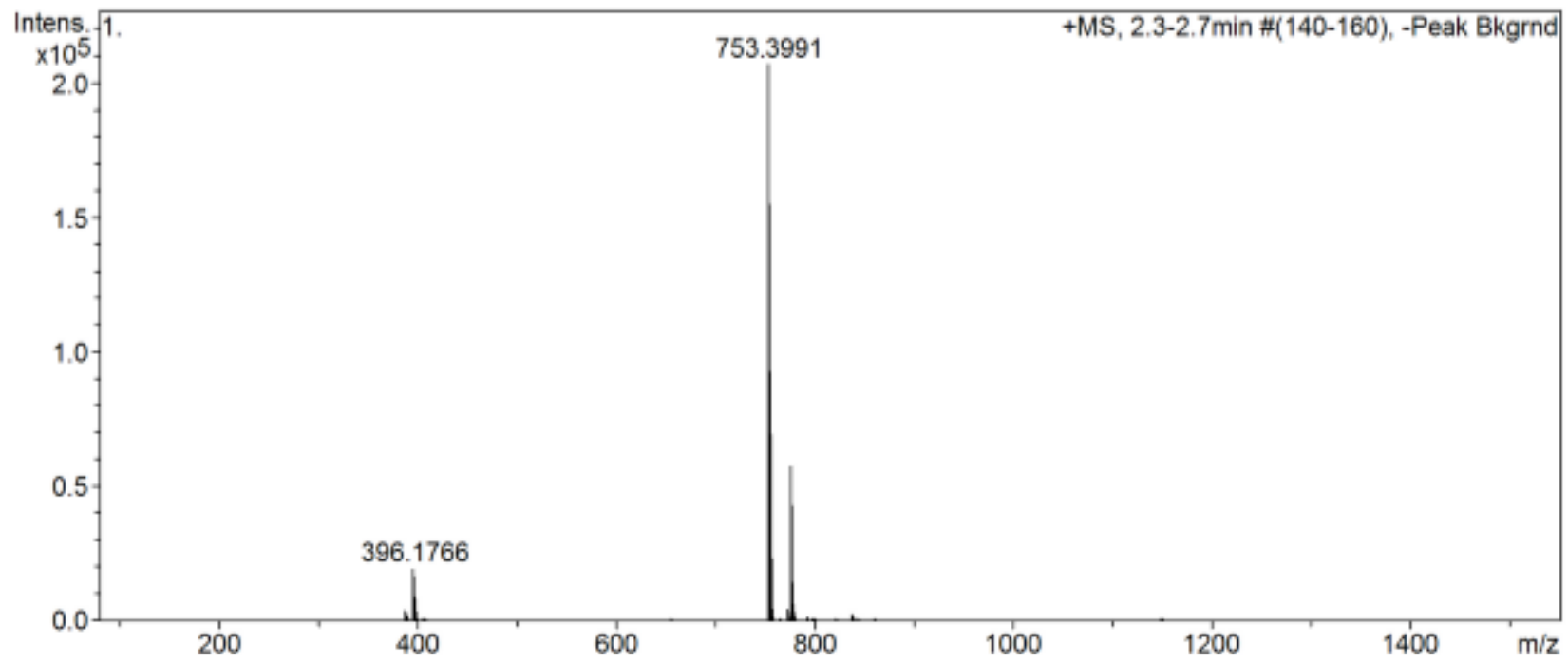

cyclo-CLL-dF-VY (**10**)

cyclo-CLL-dF-VY was obtained as a white solid (17.9 mg, 14.8 % overall yield).

Analytical HPLC: R_t = 13.746 min (94 % purity).

m/z (ESI^+): 739.8 $[M+H]^+$(100 %).

HRMS (ESI^+) found: 739.3846, $[M+H]^+$ calculated: 739.3847.

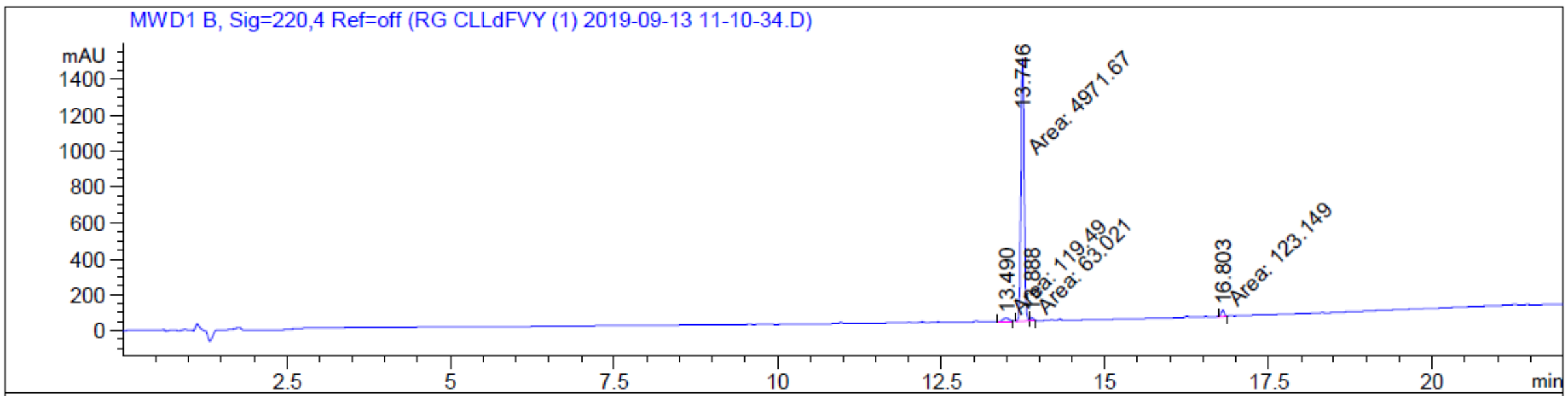

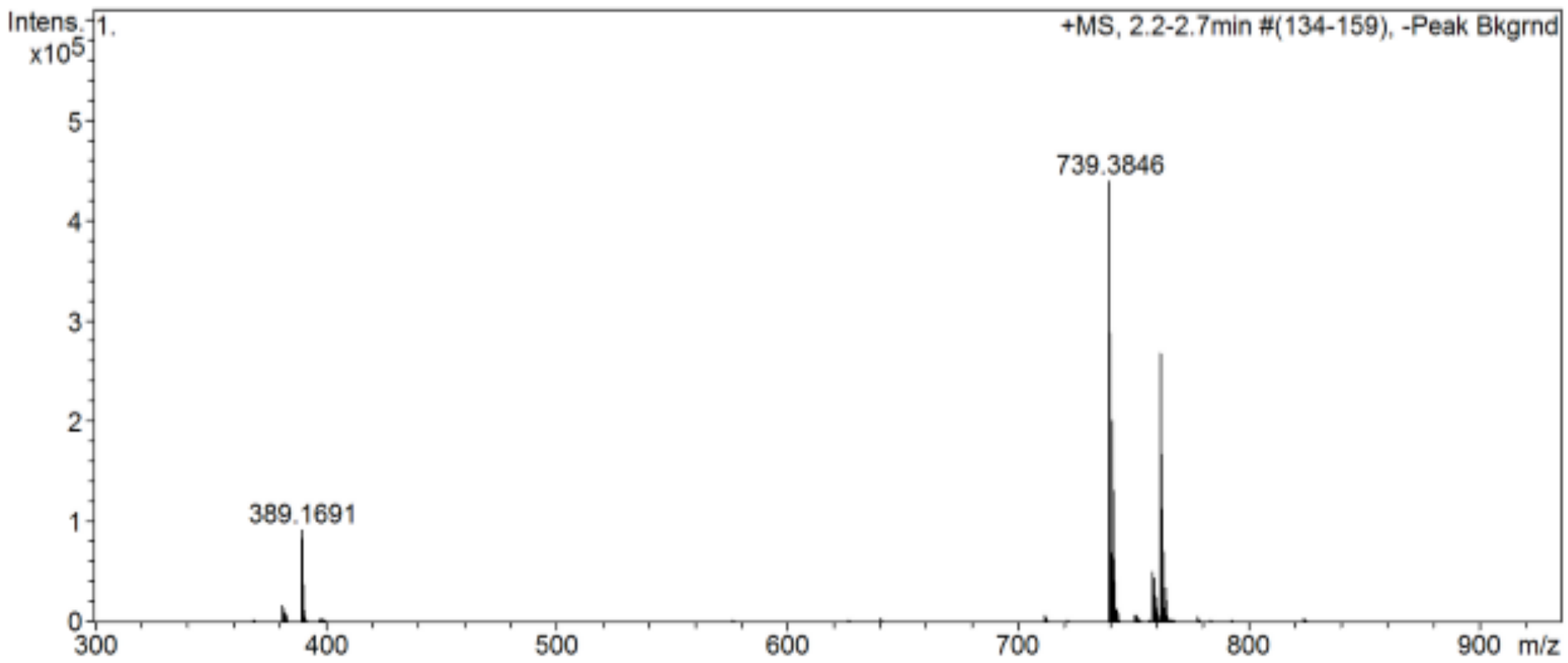

<u>*cyclo*-CLL-Nal-VY (**11**)</u>
cyclo-CLL-Nal-VY yield was obtained as a white solid (14.0 mg, 8.9 % overall yield).

Analytical HPLC: R_t = 15.317 min (92 % purity).
m/z (ESI$^+$): 789.7 [M+H]$^+$ (100 %), 811.6 [M+Na]$^+$ (35 %).
HRMS (ESI$^+$) found: 789.3997, [M+H]$^+$ calculated: 789.4004.

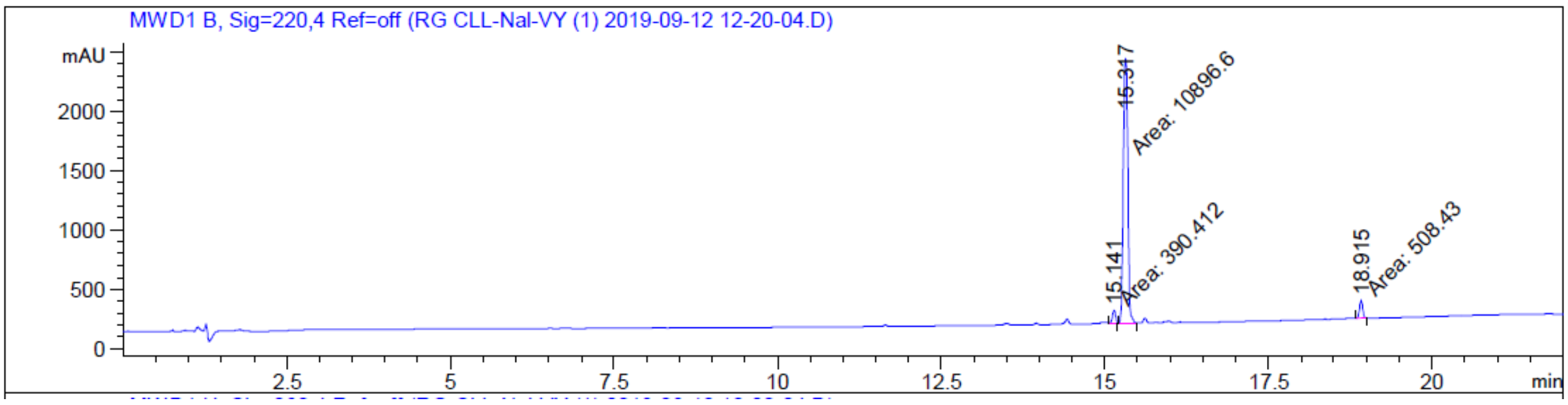

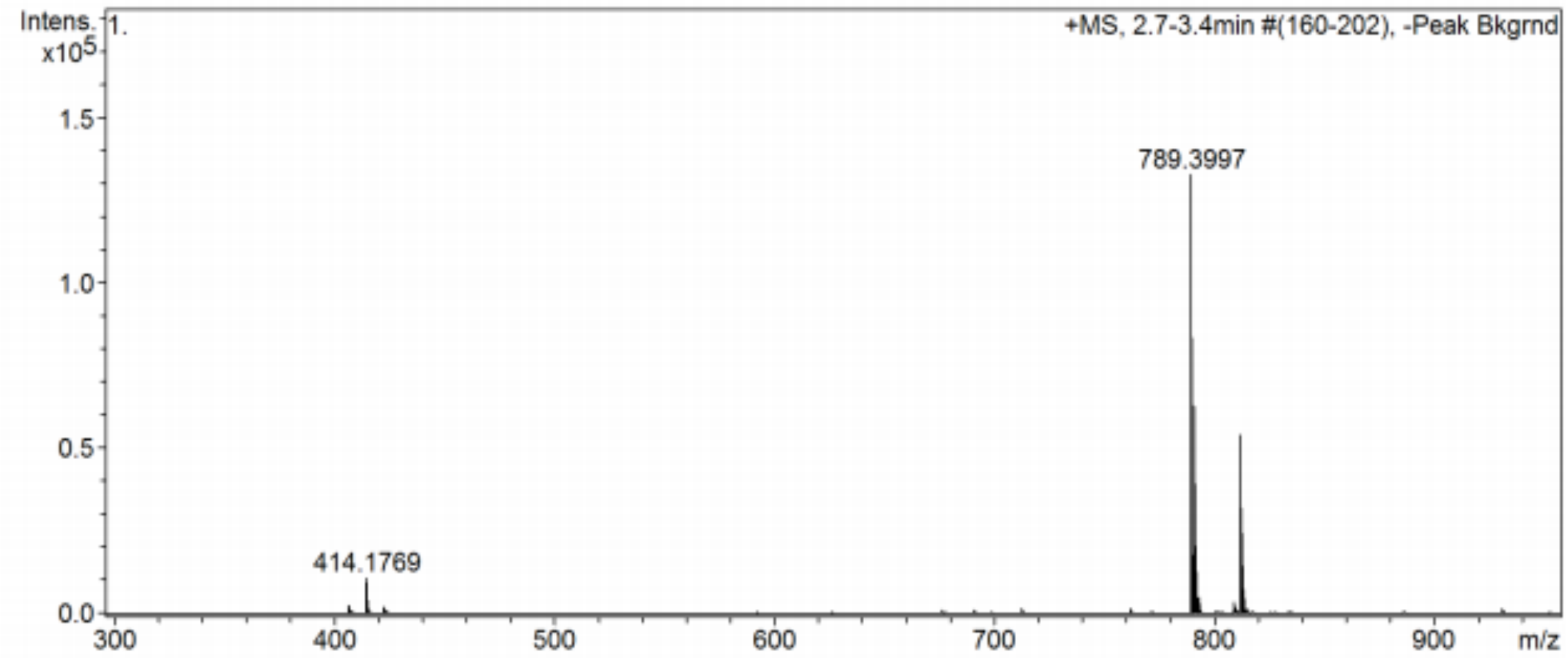

<u>*cyclo*-CLL-Cha-VY (**12**)</u>

cyclo-CLL-Cha-VY was obtained as a white solid (3.0 mg, 2.9 % overall yield).

Analytical HPLC: R_t = 15.188 min (99 % purity).
m/z (ESI$^+$): 745.8 $[M+H]^+$ (100 %), 1491.6 $[2M+H]^+$ (90 %).
HRMS (ESI$^+$) found: 745.4309, $[M+H]^+$ calculated: 745.4317.

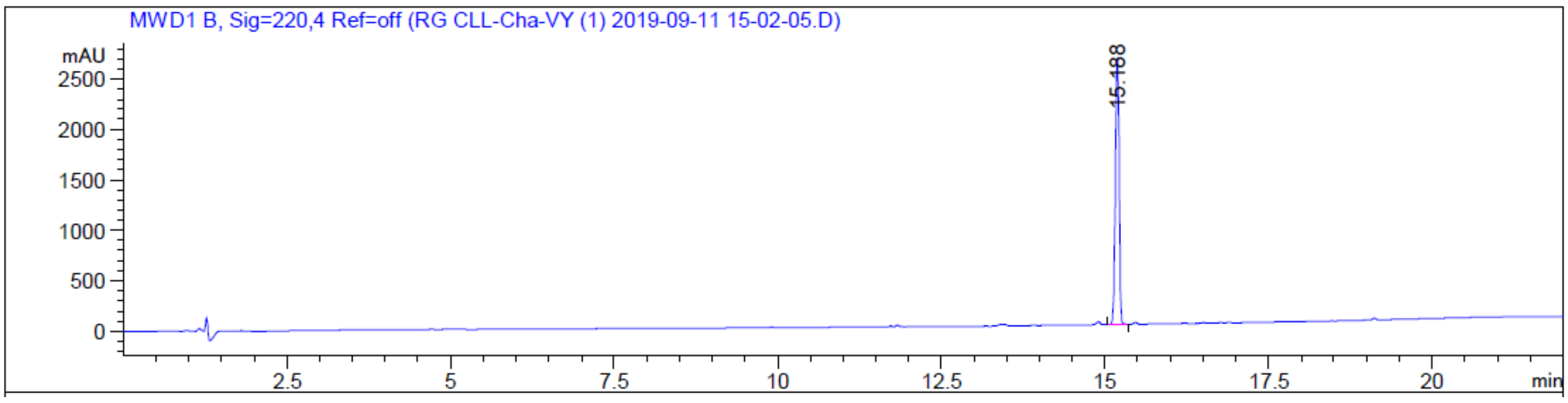

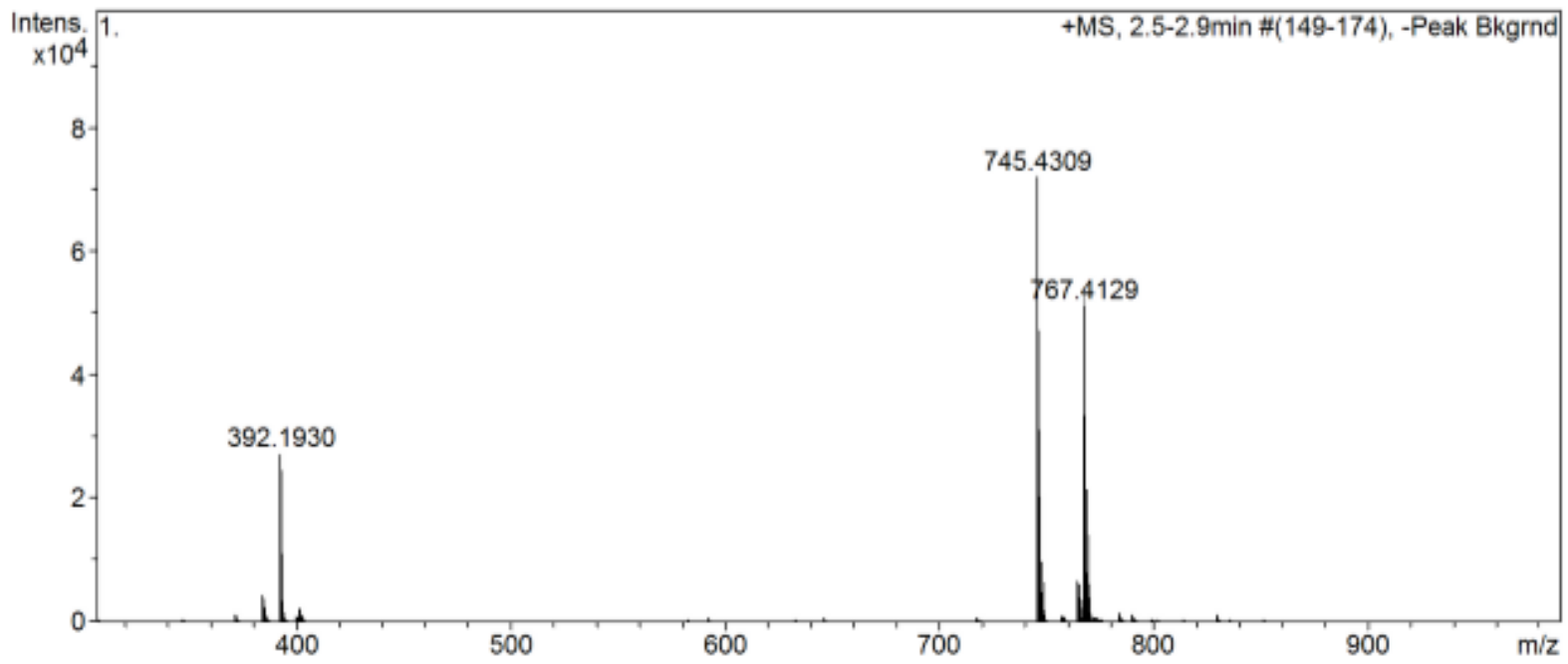

cyclo-CLL-Phg-VY (**13**)

cyclo-CLL-Phg-VY was obtained as a white solid (22.3 mg, 15.4 % overall yield).

Analytical HPLC: R_t = 12.903 min (87 % purity).

m/z (ESI^+): 725.7 $[M+H]^+$ (100 %), 1450.1 $[2M+H]^+$ (45 %).

HRMS (ESI^+) found: 725.3687, $[M+H]^+$ calculated: 725.3691.

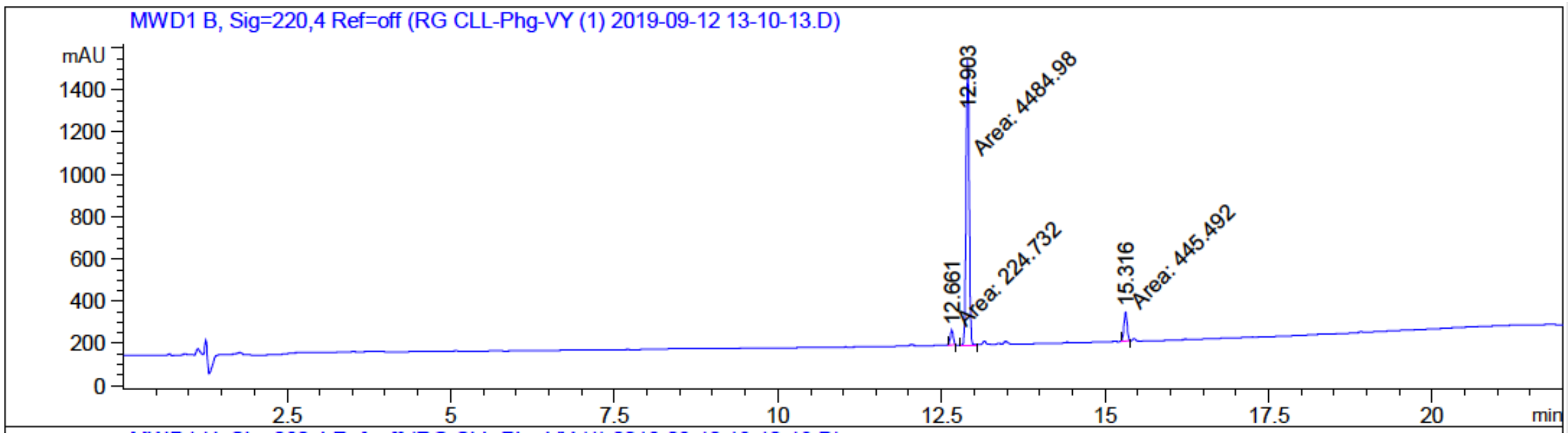

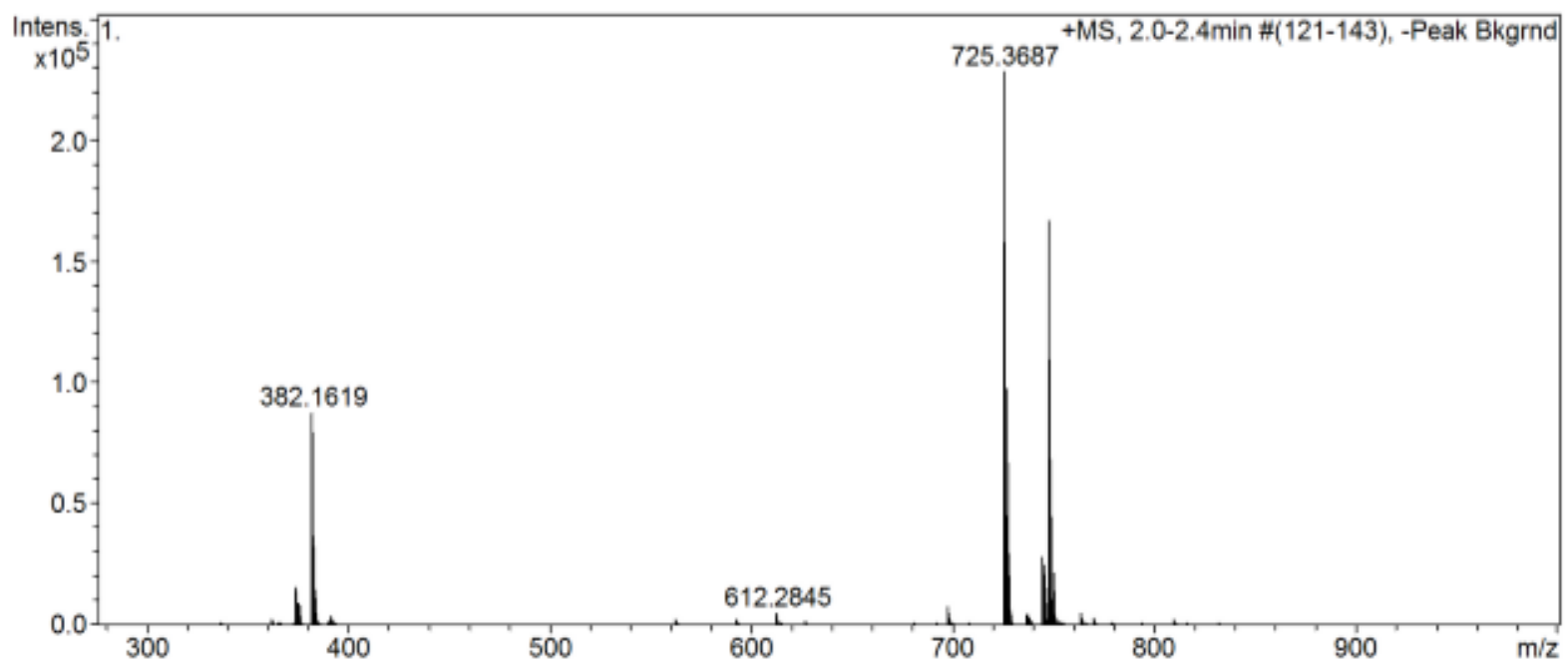

<u>*cyclo*-CLL-hCou-VY (**14**)</u>

cyclo-CLL-hCou-VY was obtained as a white solid (9.4 mg, 11.2 % overall yield).

Analytical HPLC: R_t = 10.990 min (91 % purity).
m/z (ESI$^+$): 837.5 $[M+H]^+$ (100 %), 859.5 $[M+Na]^+$ (100 %).
HRMS (ESI$^+$) found: 836.3769, $[M+H]^+$ calculated: 836.3779.

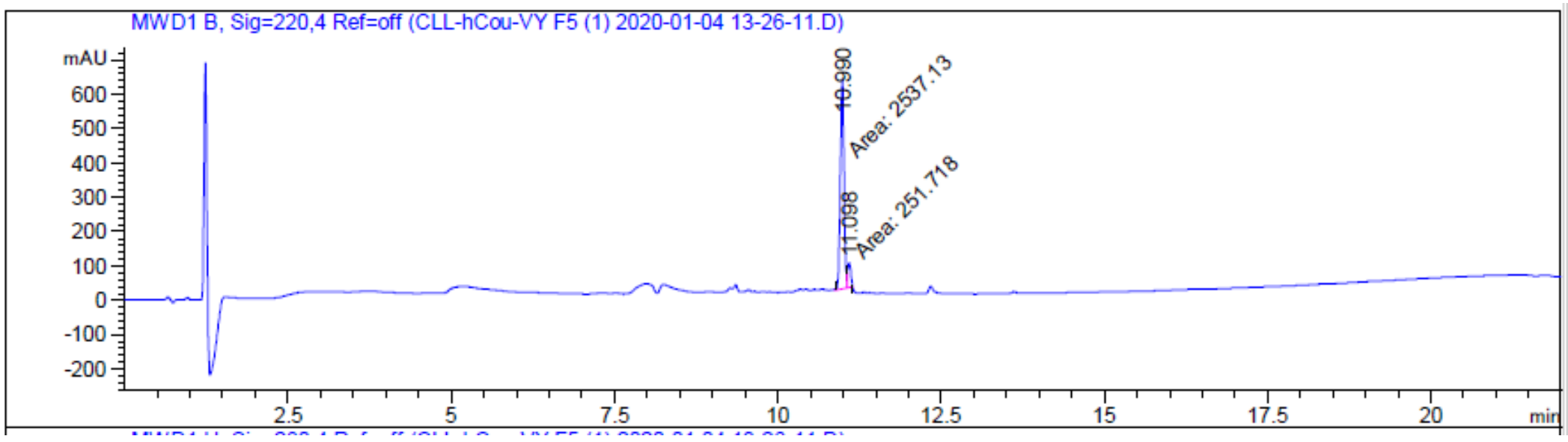

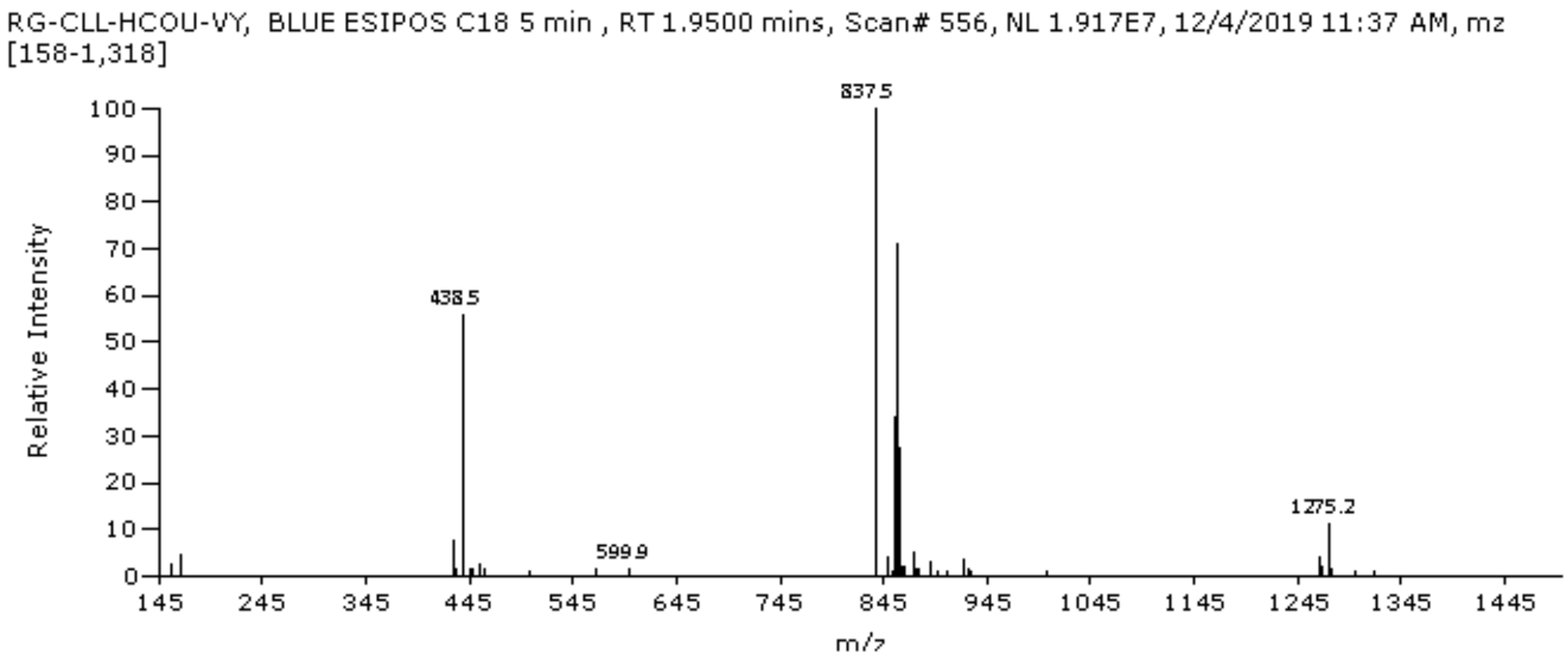

cyclo-CLL-Pal-VY (**15**)

cyclo-CLL-Pal-VY was obtained as white solid (16.2 mg, 15.1 % overall yield).

Analytical HPLC: R_t = 9.721 min (89 % purity).

m/z (ESI^+): 740.8 $[M+H]^+$ (100 %).

HRMS (ESI^+) found: 740.3817, $[M+H]^+$ calculated: 740.3800.

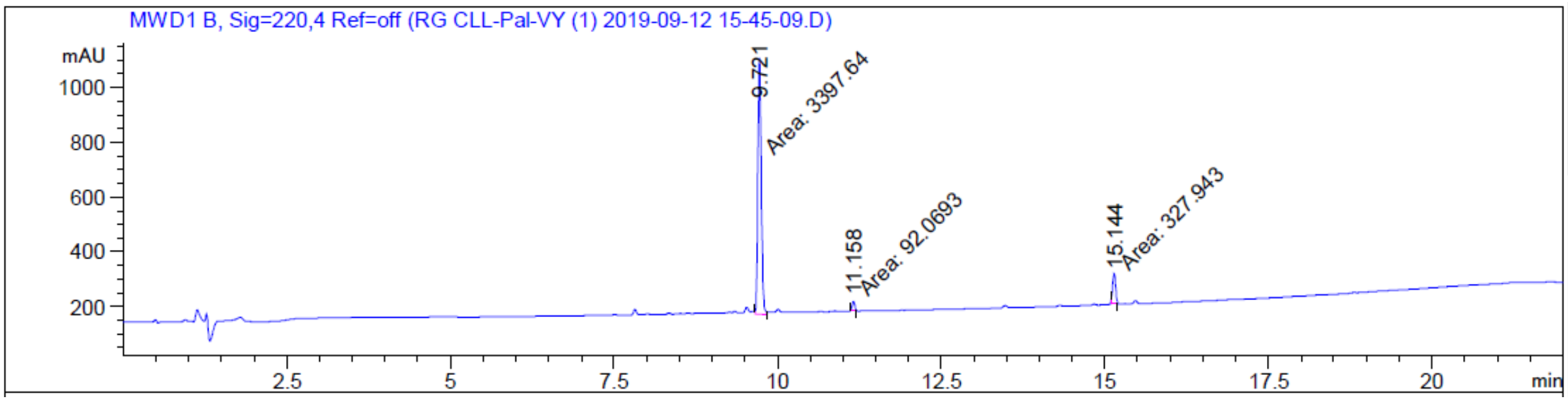

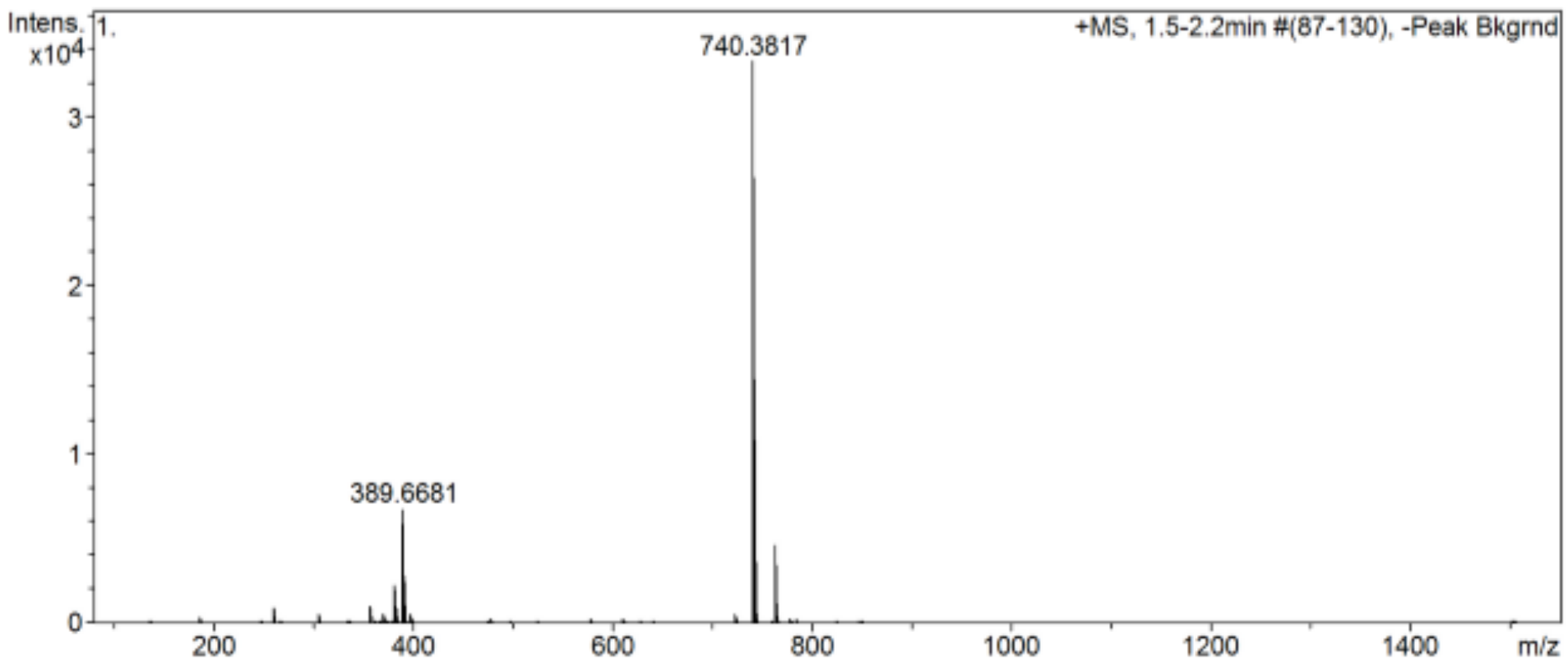

cyclo-CLLYVY (**16**)

cyclo-CLLYVY was obtained as white solid (25.1 mg, 22.2 % overall yield).

Analytical HPLC: R_t = 11.830 min (99 % purity).

m/z (ESI^+): 755.7 $[M+H]^+$ (100 %).

HRMS (ESI^+) found: 755.3791, $[M+H]^+$ calculated: 755.3797.

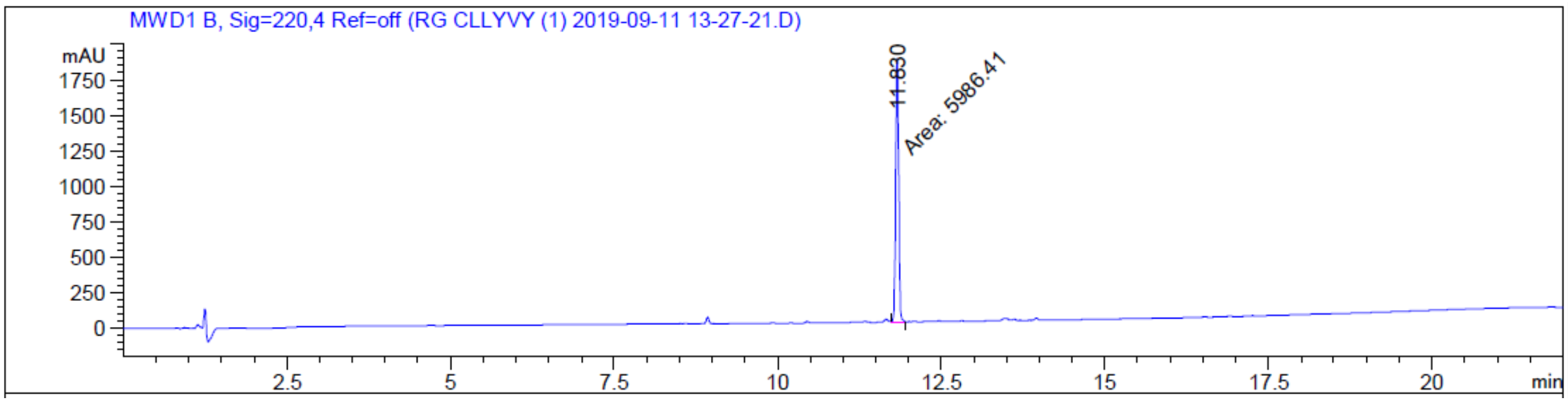

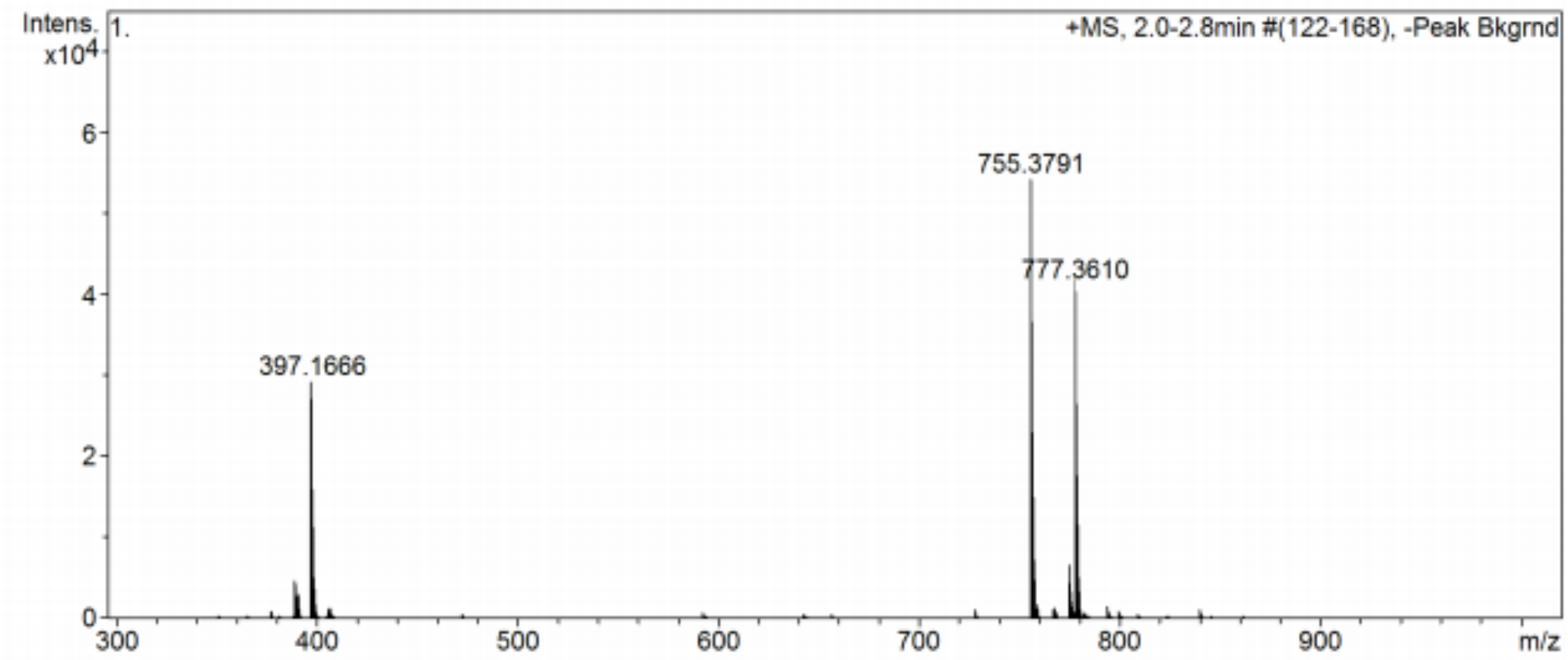

<u>*cyclo*-CLLY(Me)VY (**17**)</u>

cyclo-CLLY(Me)VY was obtained as a white solid yield (3.7 mg, 3.3 % overall yield).
Analytical HPLC: R_t = 13.419 min (90 % purity).
m/z (ESI^+): 769.8 $[M+H]^+$ (100 %), 791.8 $[M+Na]^+$ (25 %).
HRMS (ESI^+) found: 769.3944, $[M+H]^+$ calculated: 769.3953.

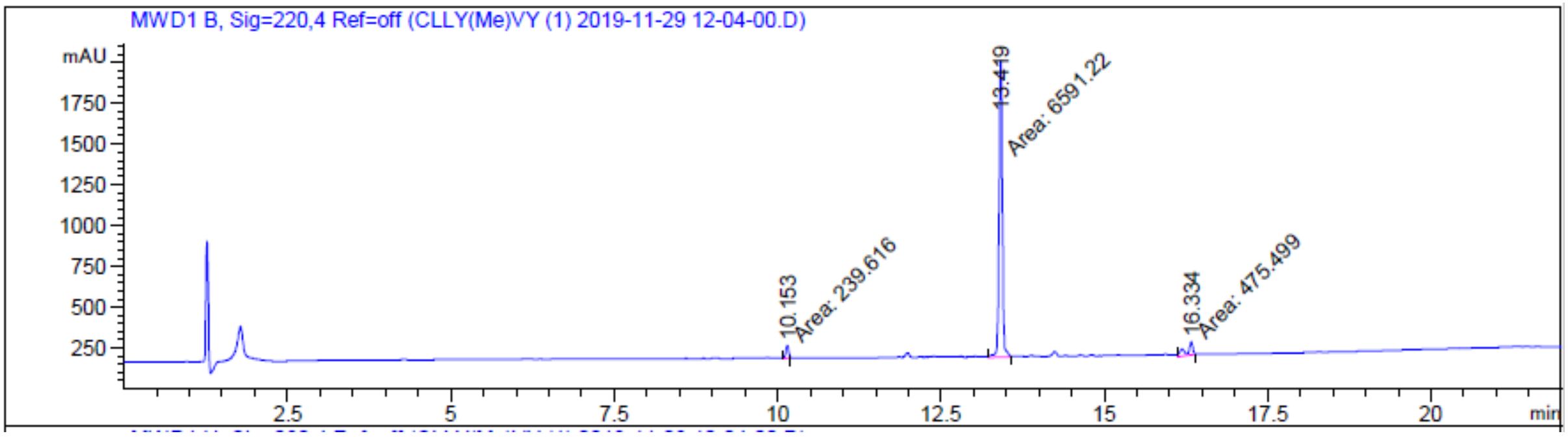

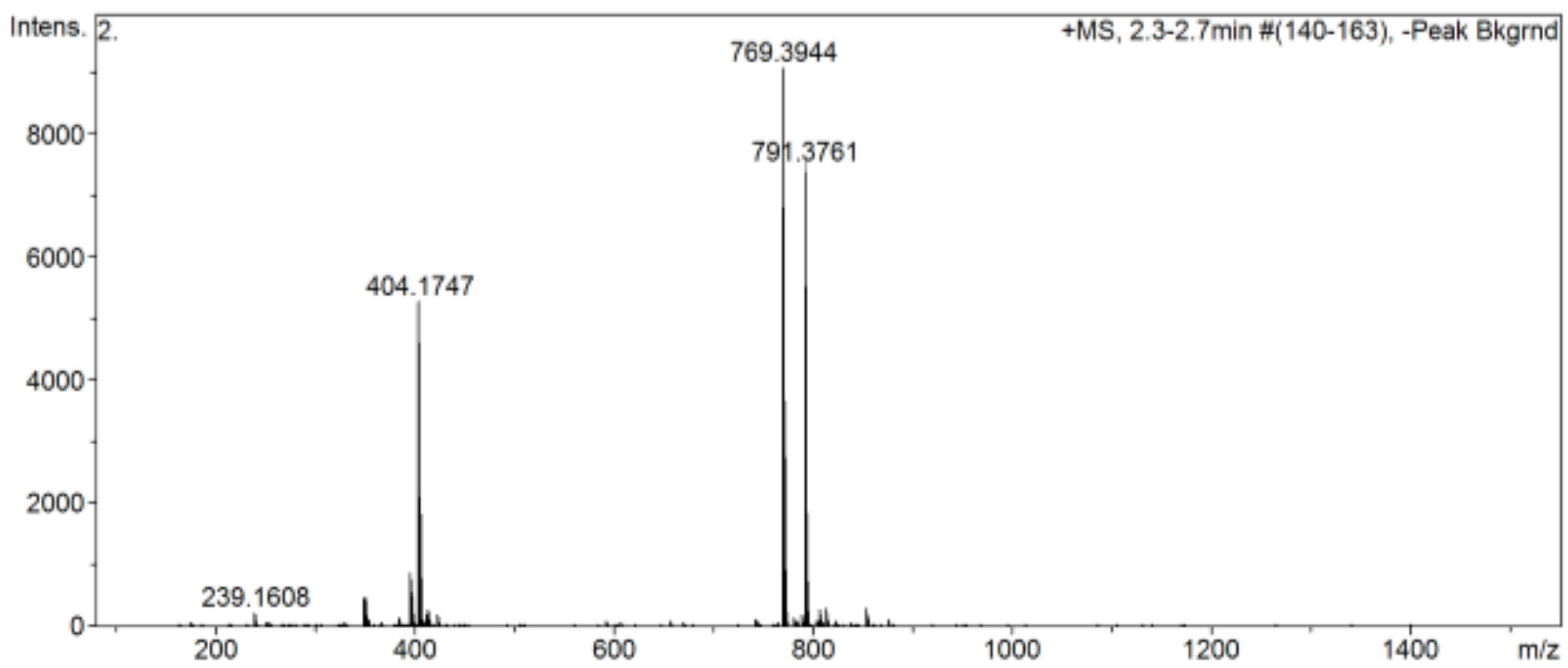

cyclo-CLL(p-F)FVY (**18**)

cyclo-CLL(*p*-F)FVY was obtained as a white solid (11.9 mg, 10.9 % overall yield).

Analytical HPLC: R_t = 13.905 min (92 % purity).

m/z (ESI$^+$): 757.8 $[M+H]^+$ (100 %).

HRMS (ESI$^+$) found: 757.3751, $[M+H]^+$ calculated: 757.3753.

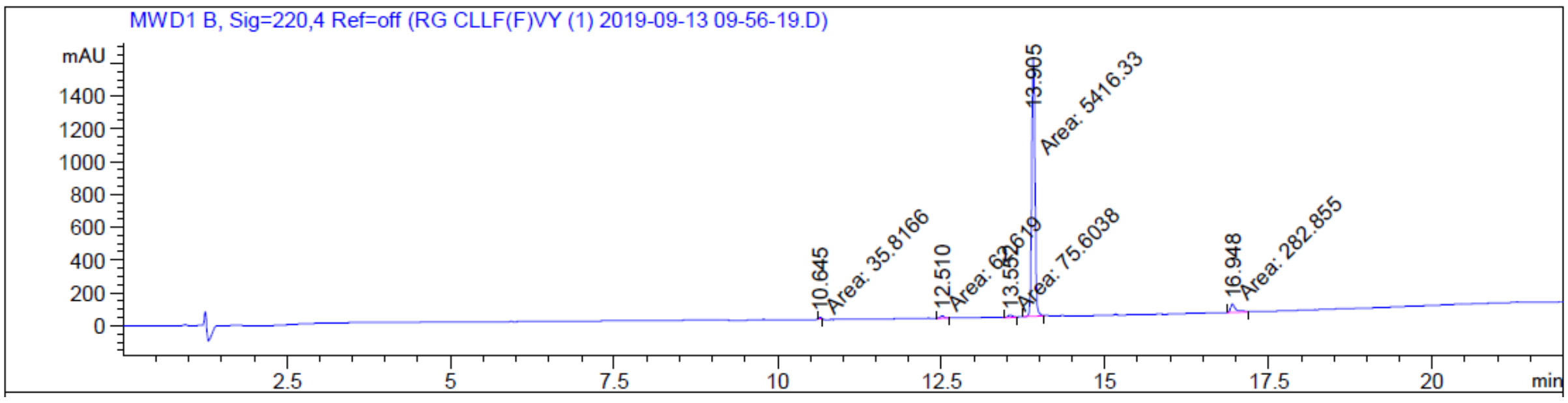

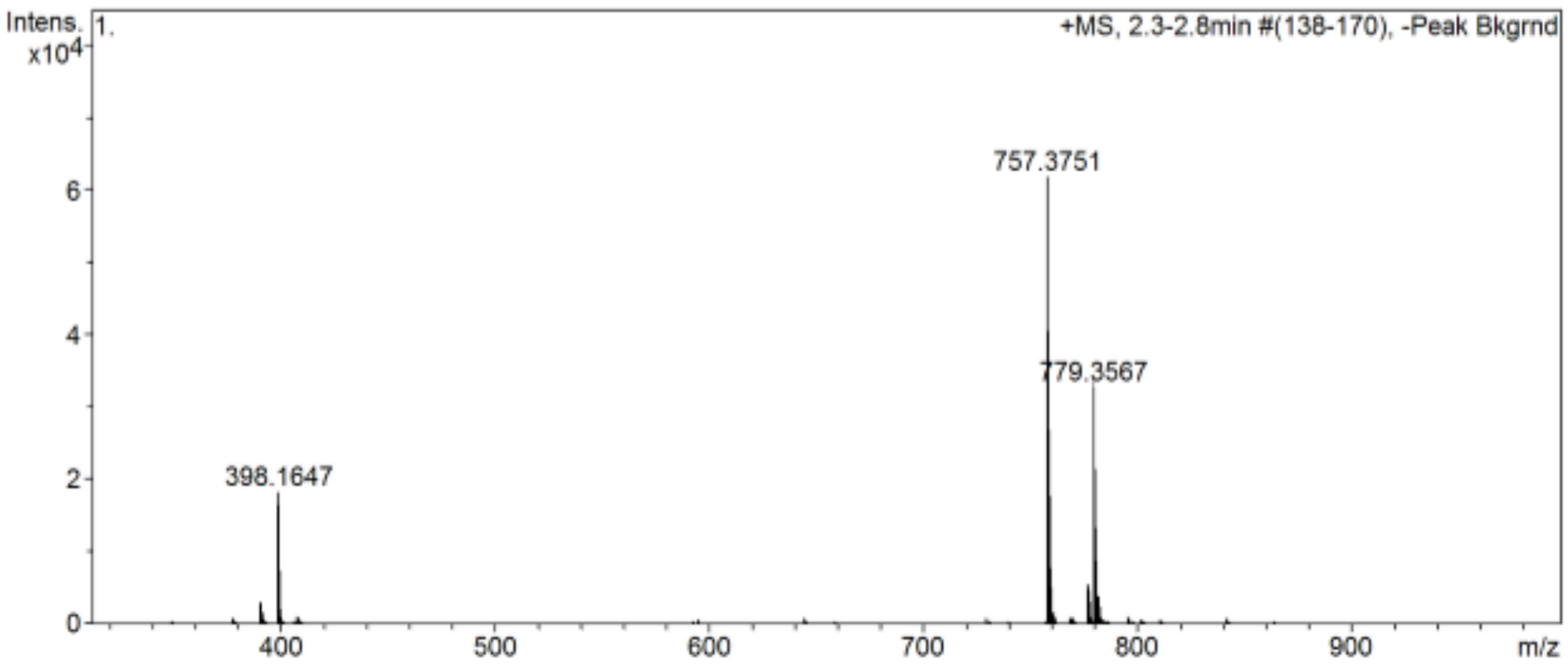

<u>*cyclo*-CLL(*p*-Cl)FVY (**19**)</u>

Cyclo-CLL(*p*-Cl)FVY was obtained as a white solid (8.5 mg, 7.1 % overall yield).

Analytical HPLC: R_t = 14.519 min (94 % purity).
m/z (ESI$^+$): 773.7 $[M+H]^+$ (100 %), 795.7 $[M+Na]^+$ (30 %).
HRMS (ESI$^+$) found: 773.3452, $[M+H]^+$ calculated: 773.3458.

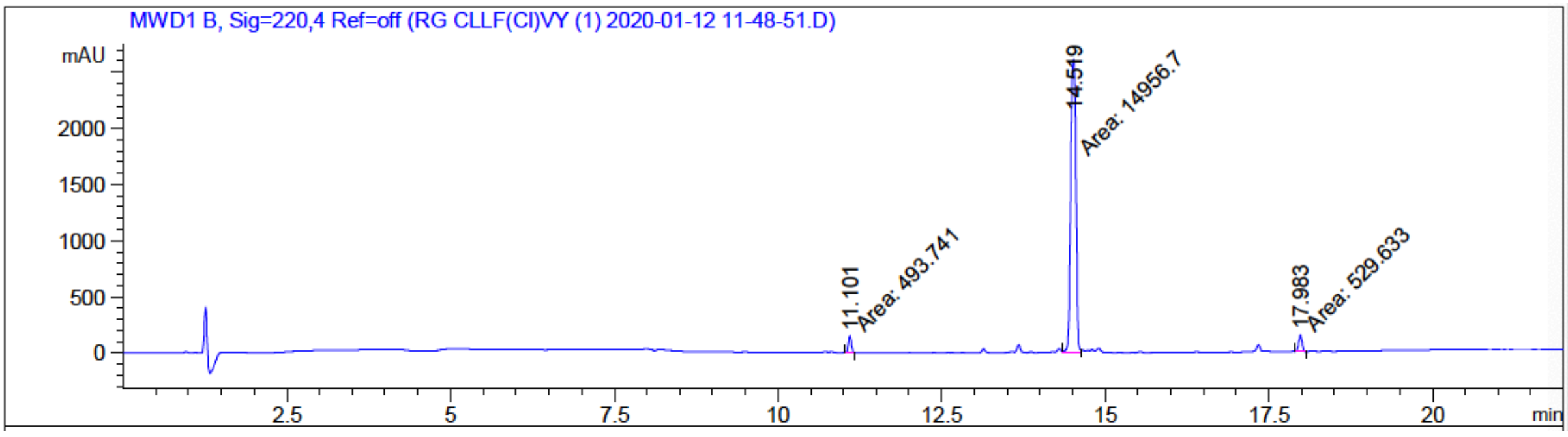

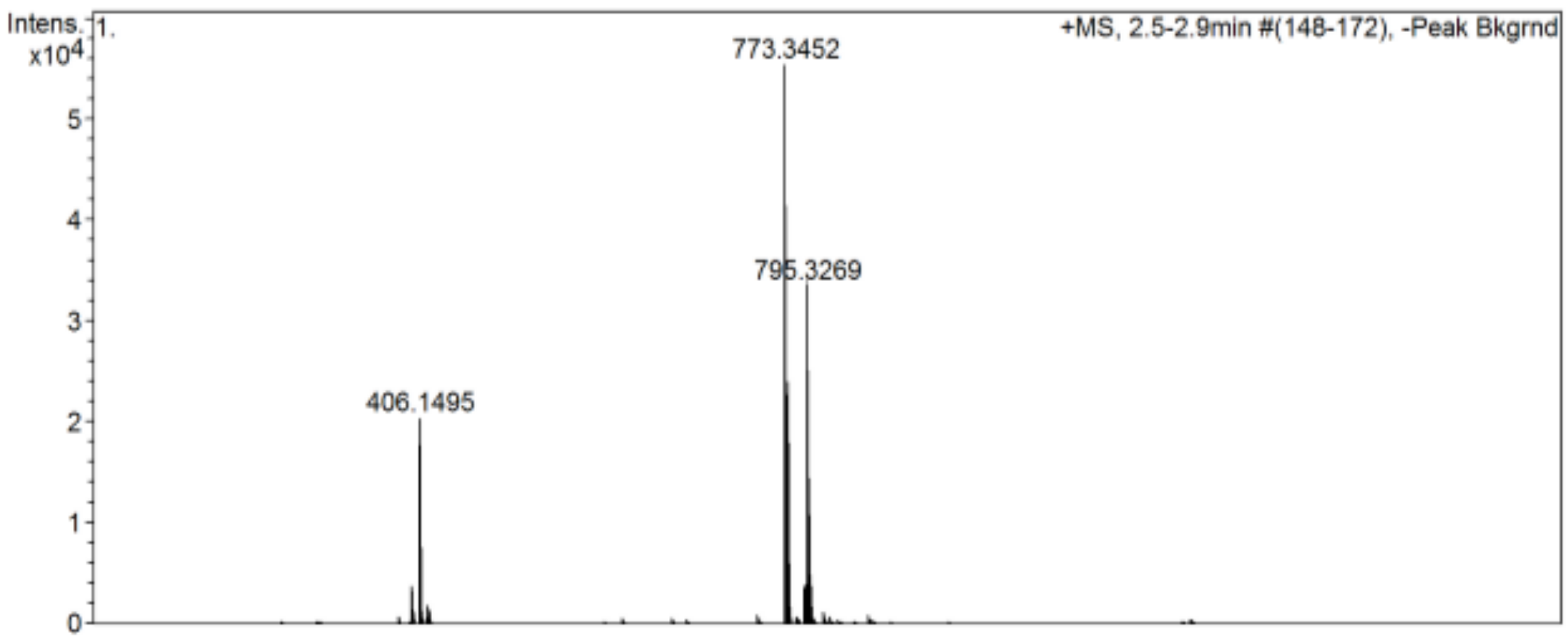

<u>*cyclo*-CLL(*p*-Br)FVY (**20**)</u>
cyclo-CLLF(Br) VY was obtained as a white solid (12.1 mg, 7.4 % overall yield).

Analytical HPLC: R_t = 14.657 min (90 % purity).
m/z (ESI$^+$): 819.5 $[M+H]^+$ (100 %).
HRMS (ESI$^+$) found: 817.2926, $[M+H]^+$ calculated: 817.2953.

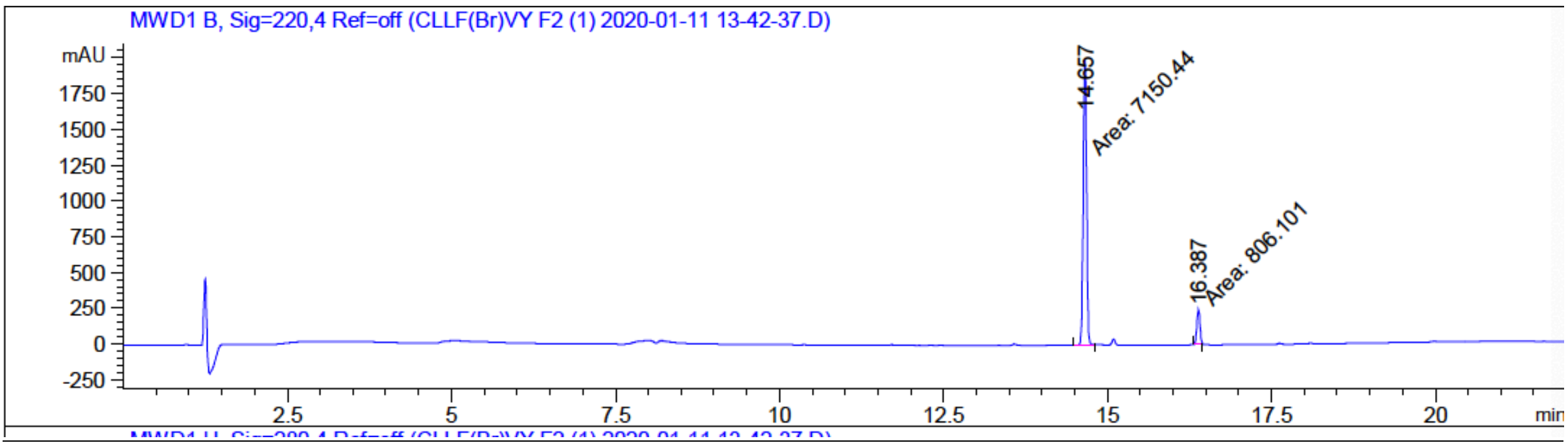

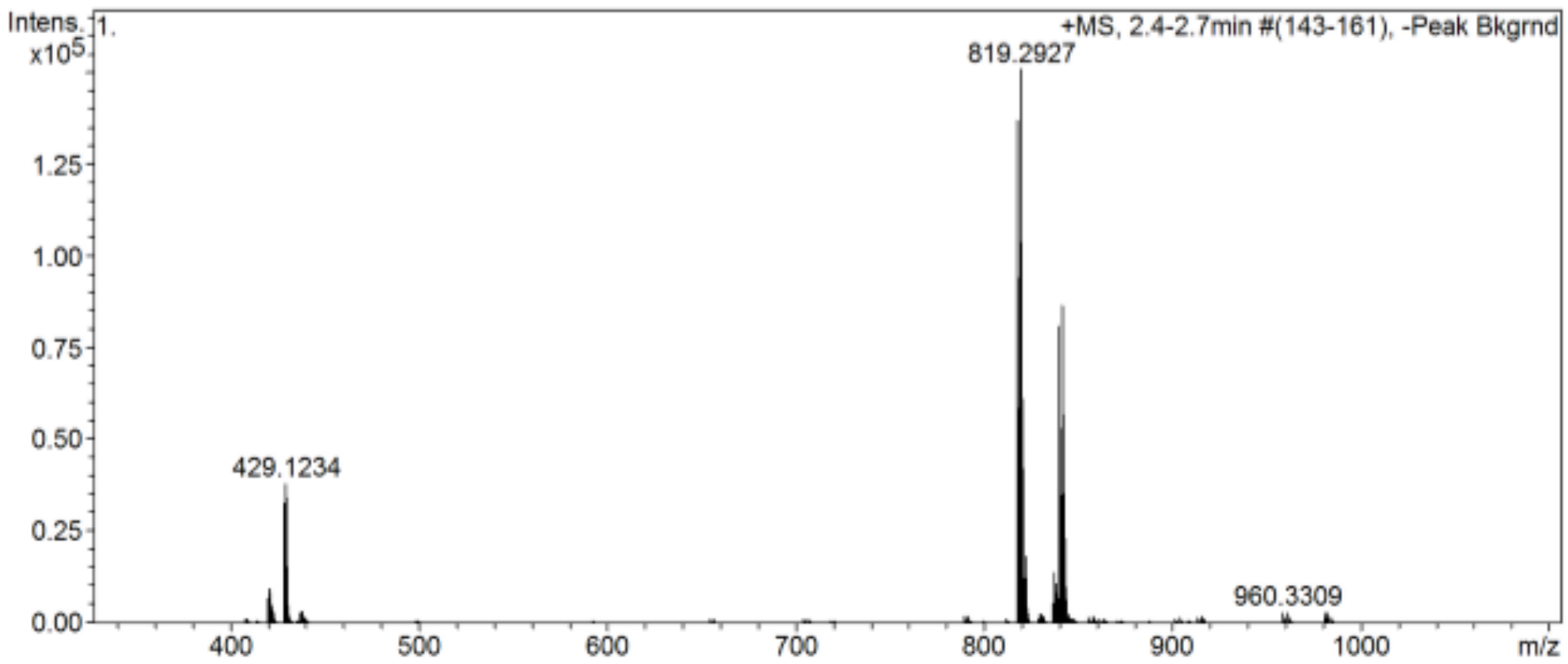

cyclo-CLL(p-I)FVY (**21**)

cyclo-CLL(*p*-I)FVY was obtained as a white solid (15.8 mg, 9.1 % overall yield).

Analytical HPLC: R_t = 15.144 min (90 % purity).
m/z (ESI$^+$): 865.7 $[M+H]^+$ (100 %).
HRMS (ESI$^+$) found: 865.2803, $[M+H]^+$ calculated: 865.2814.

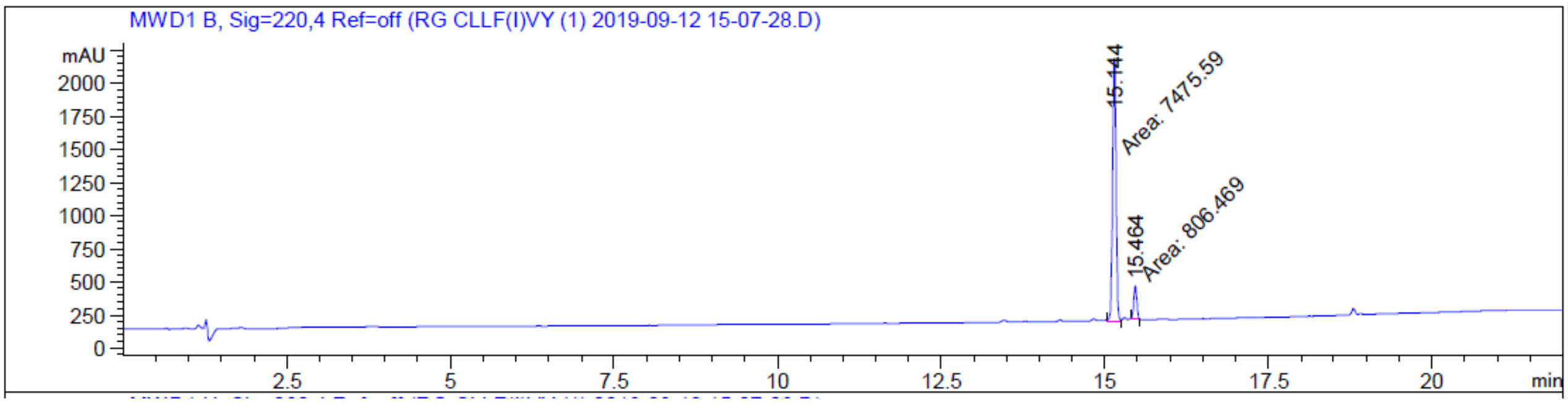

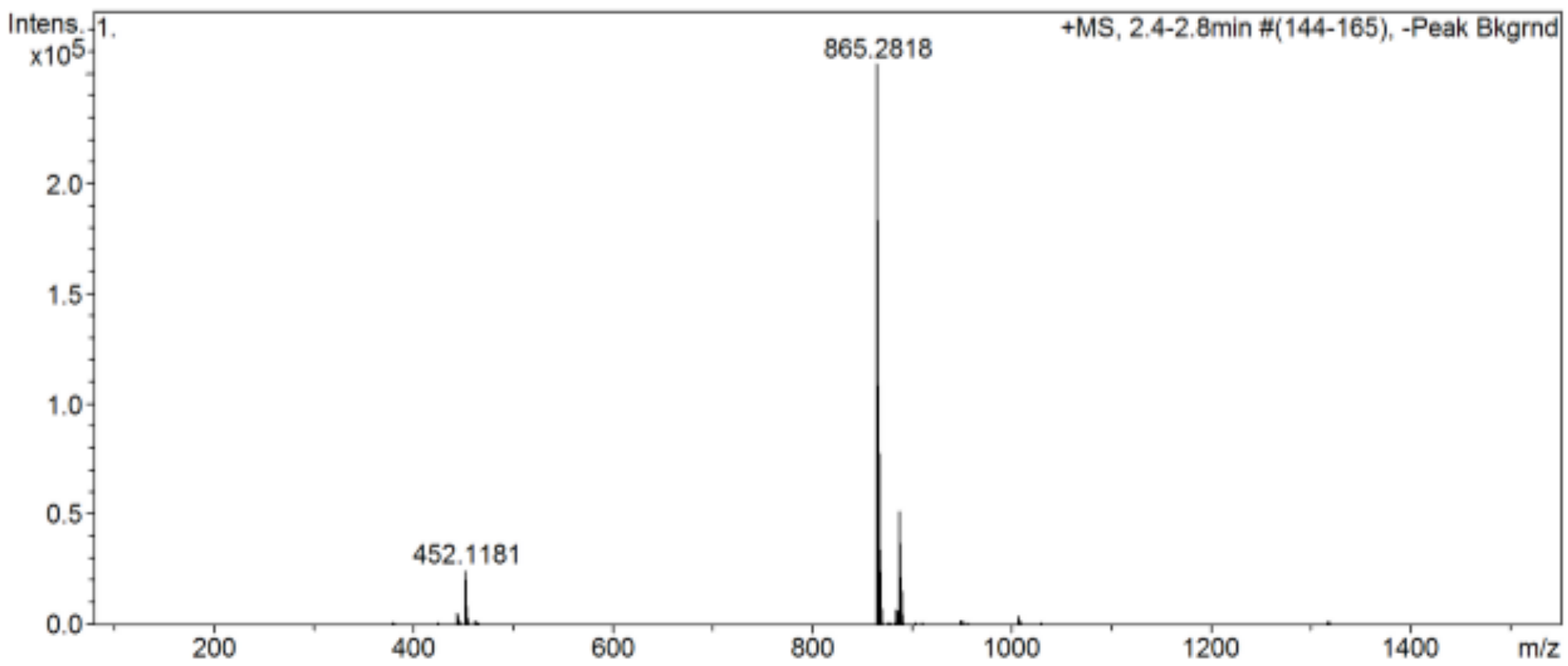

cyclo-CLL(p-CF_3)FVY (**22**)

cyclo-CLL(*p*-CF_3)FVY was obtained as a white solid (29.4 mg, 18.2 % overall yield).

Analytical HPLC: R_t = 14.840 min (90 % purity).

m/z (ESI^+): 807.7 $[M+H]^+$ (100 %), 829.7 $[M+Na]^+$ (60 %).

HRMS (ESI^+) found: 807.3720, $[M+H]^+$ calculated: 807.3721.

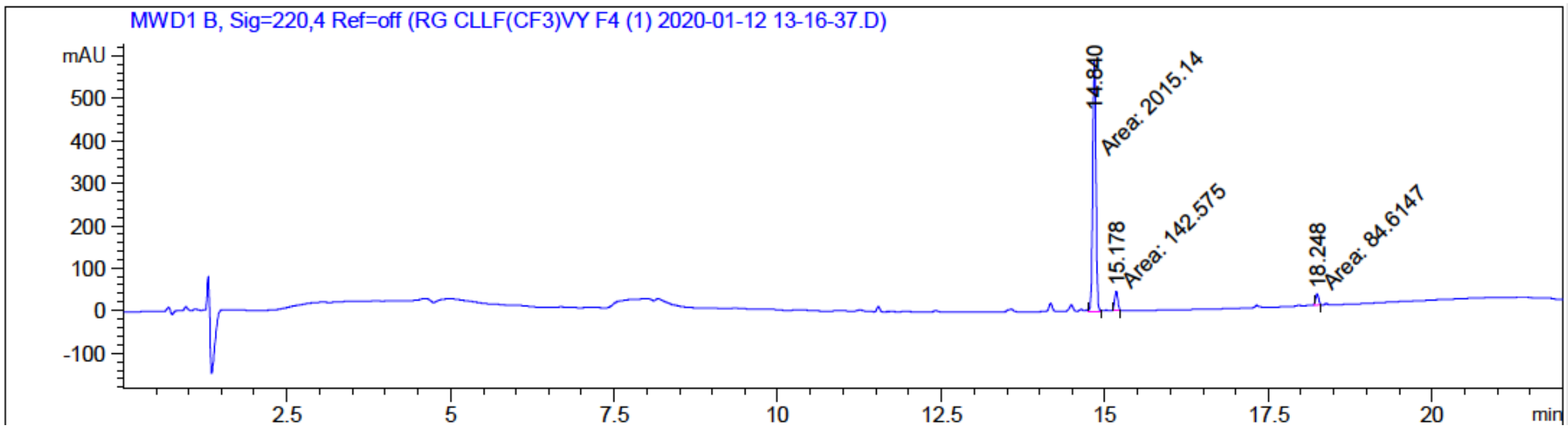

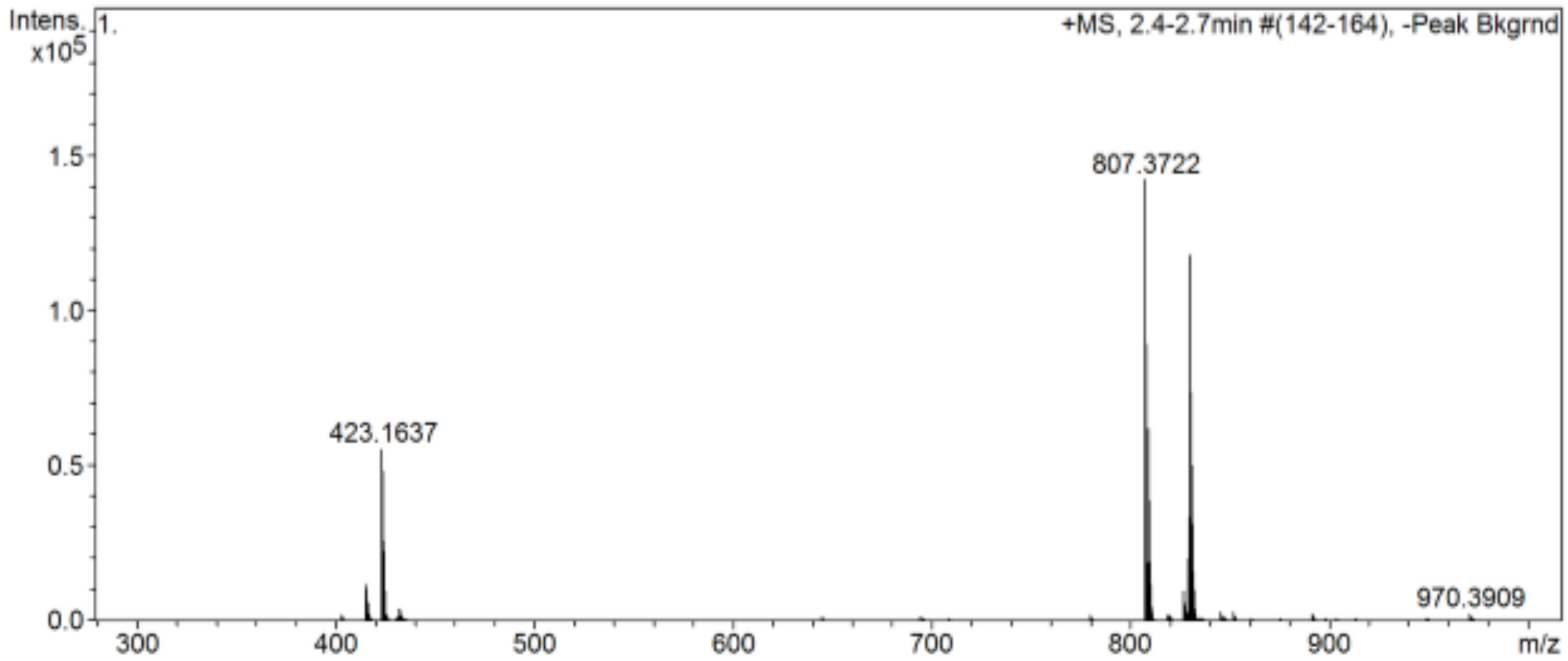

cyclo-CLL(*p*-CF_3)dFVY (**23**)

cyclo-CLL(*p*-CF_3)dFVY was obtained as a white solid (40.9 mg, 25.4 % overall yield).

Analytical HPLC: R_t = 16.163 min (93 % purity).
m/z (ESI^+): 807.57 $[M+H]^+$ (100 %), 829.51 $[M+Na]^+$ (20 %).
HRMS (ESI^+) found: 807.3714, $[M+H]^+$ calculated: 807.3721.

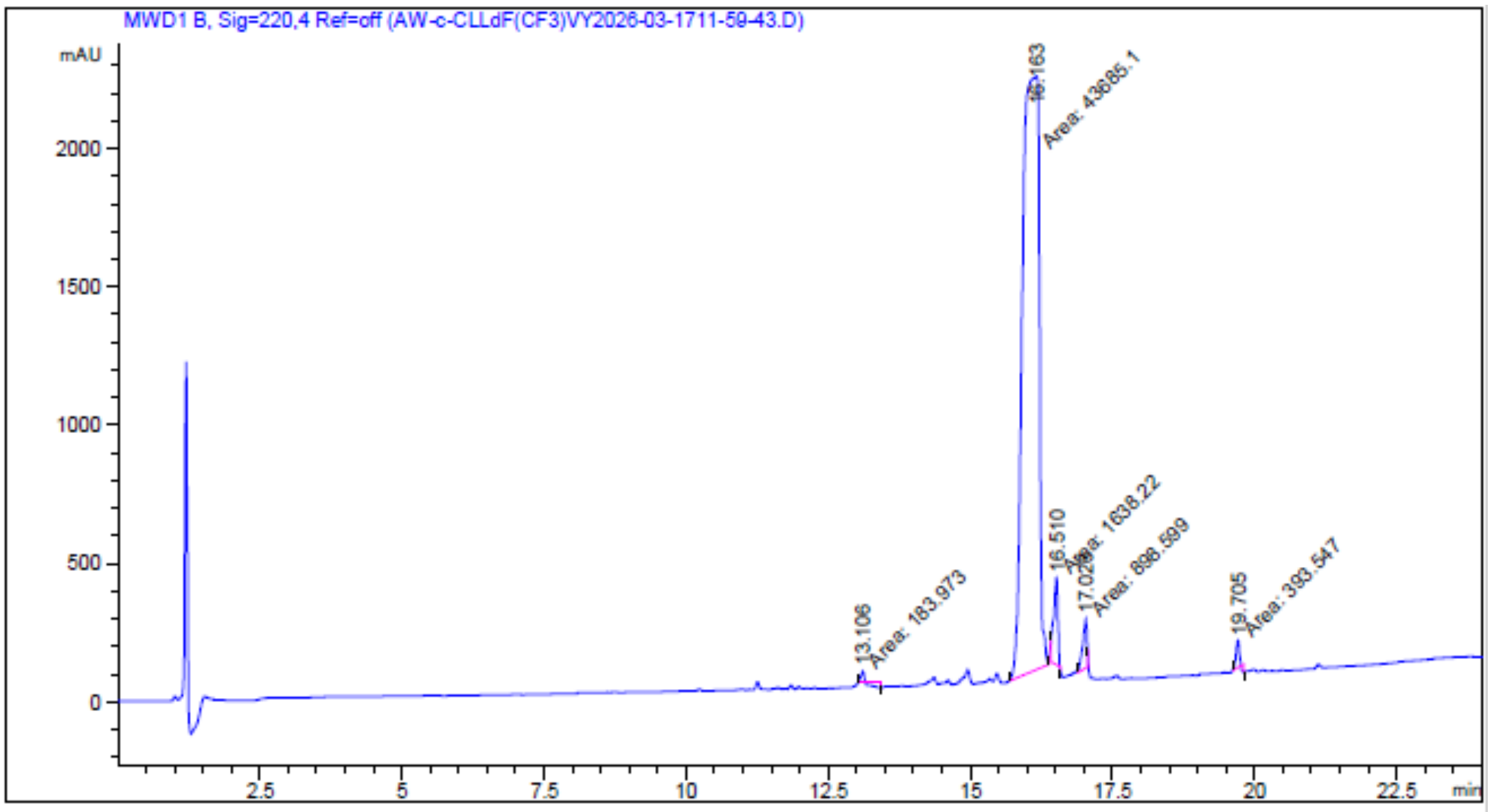

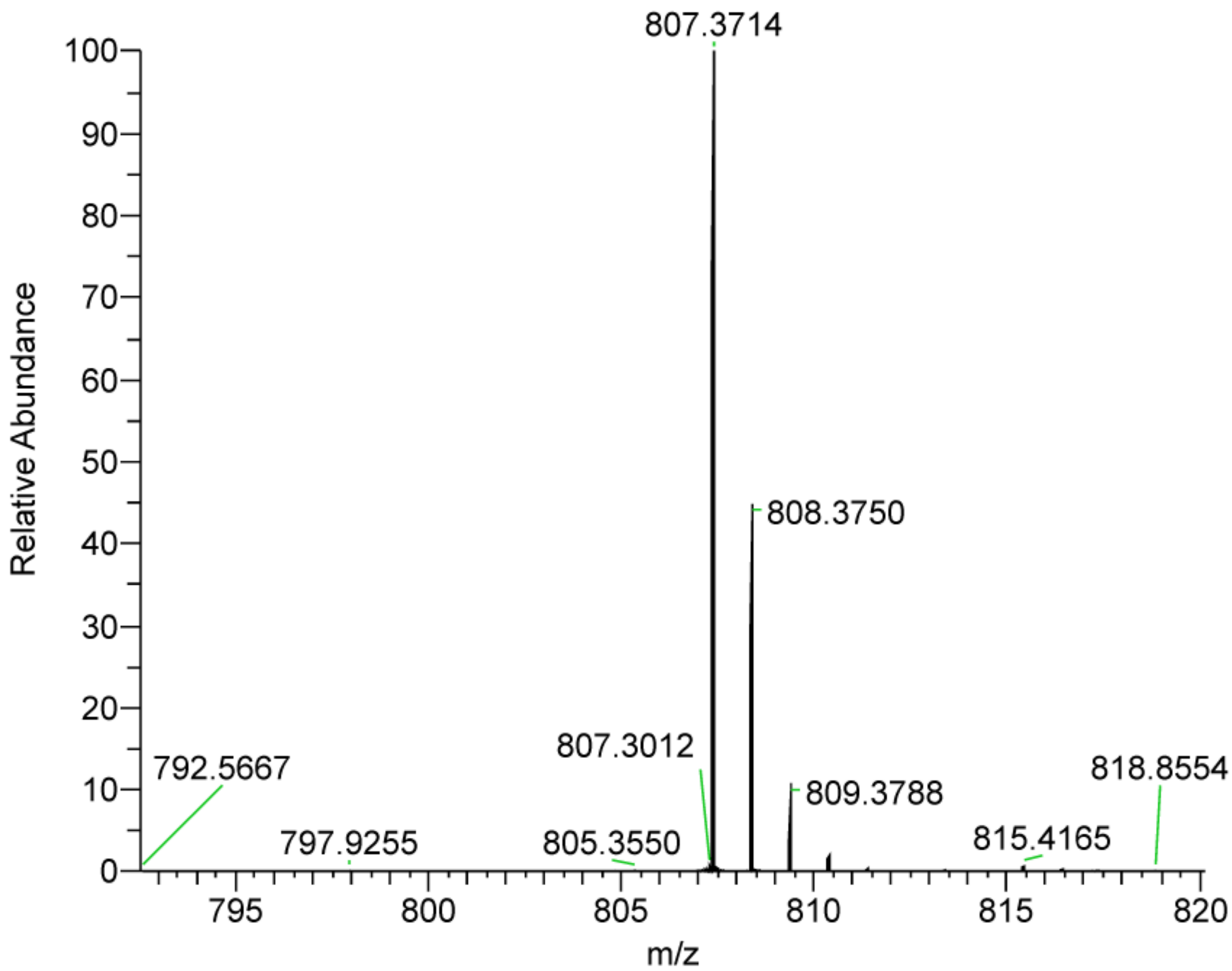

cyclo-CLL(*p*-Ph)FVY (**24**)

cyclo-CLL(*p*-Ph)FVY was obtained as a white solid (22.3 mg, 13.7 % overall yield).

Analytical HPLC: R_t = 17.023 min (94 % purity).

m/z (ESI^+): 815.6 $[M+H]^+$ (100 %), 837.5 $[M+Na]^+$ (20 %).

HRMS (ESI^+) found: 814.4084, $[M+H]^+$ calculated: 814.4088.

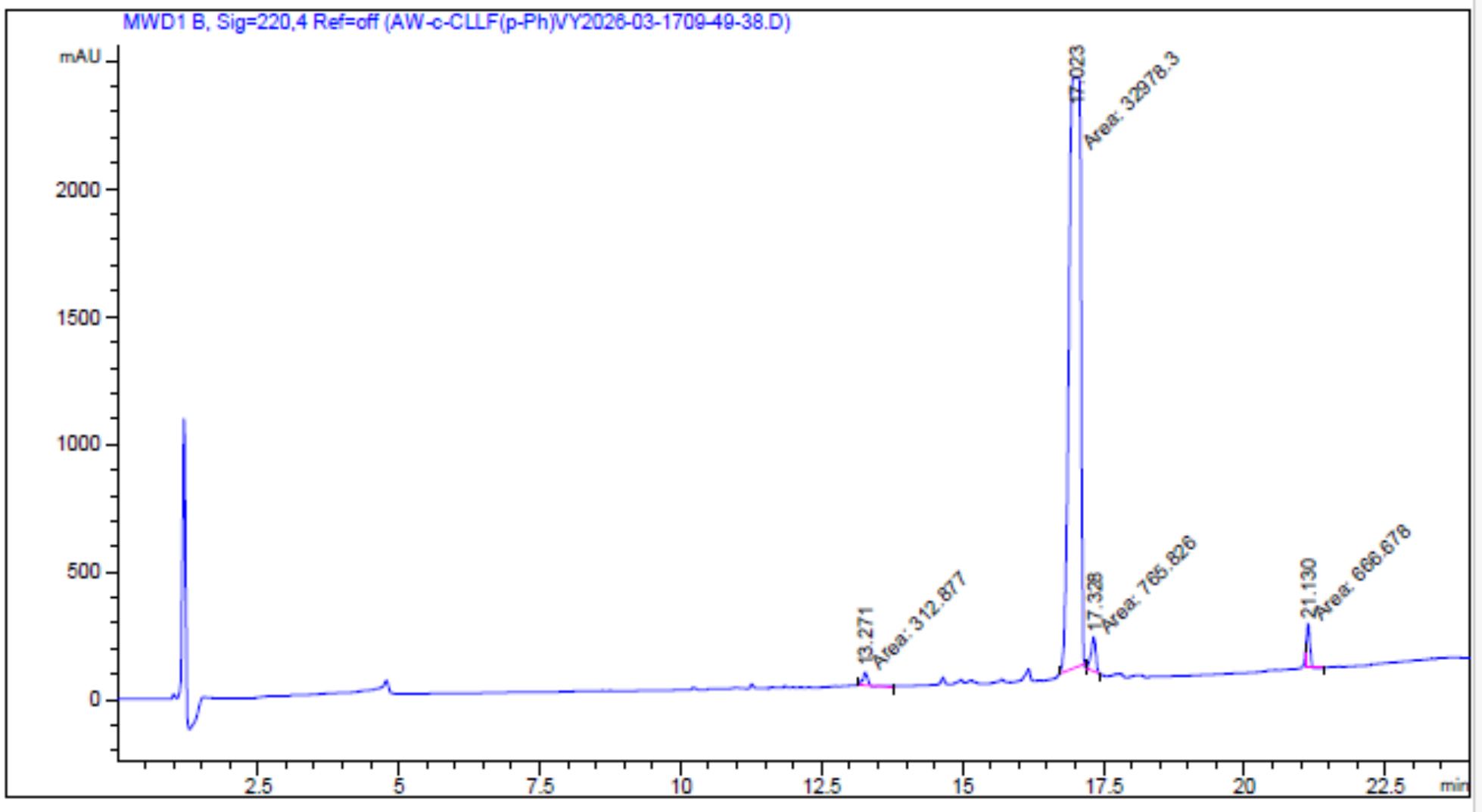

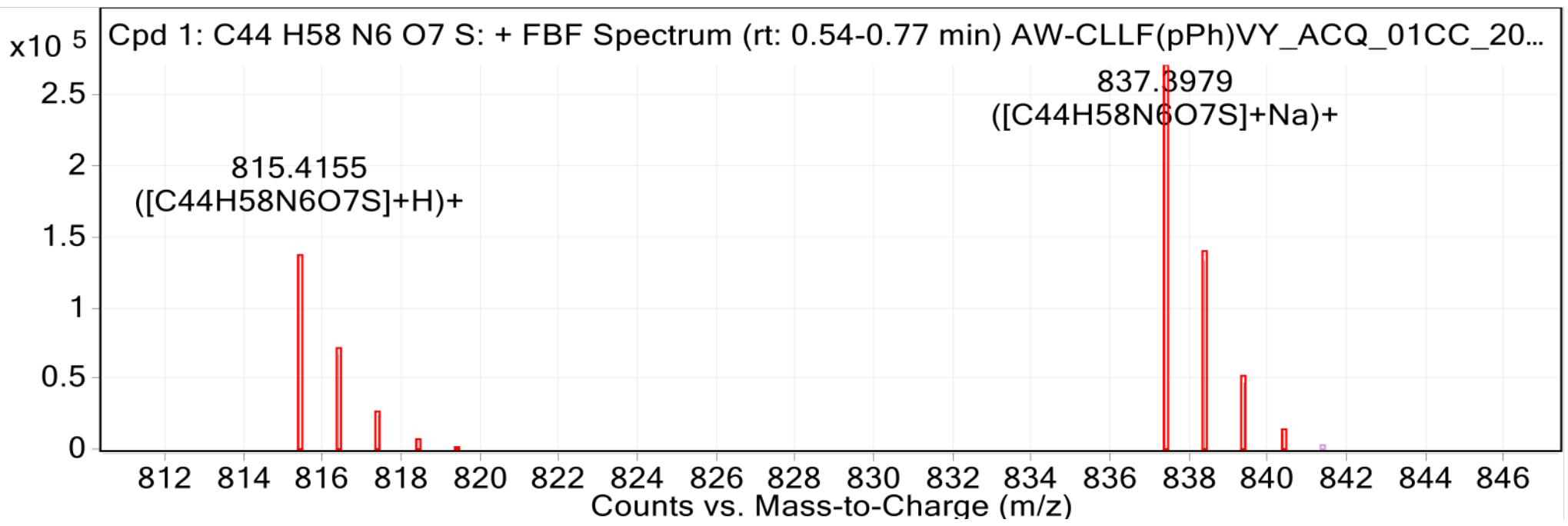

Compound Label	RT (min)	Observed mass (m/z)	Neutral observed mass (Da)	Theoretical mass (Da)	Mass error (ppm)	Isotope match score (%)
Cpd 1: C44 H58 N6 O7 S	0.64	837.3979	814.4084	814.4088	-0.41	99.07

Mass errors of between -5.00 and 5.00 ppm with isotope match scores above 60% are considered confirmation of molecular formulae

cyclo-CLL(*p*-tBu)FVY (**25**)

cyclo-CLL(*p*-tBu)FVY was obtained as a white solid (16.6 mg, 10.4 % overall yield).

Analytical HPLC: R_t = 16.384 min (93 % purity).
m/z (ESI$^+$): 795.87 $[M+H]^+$ (100 %).
HRMS (ESI$^+$) found: 795.4449, $[M+H]^+$ calculated: 795.4473.

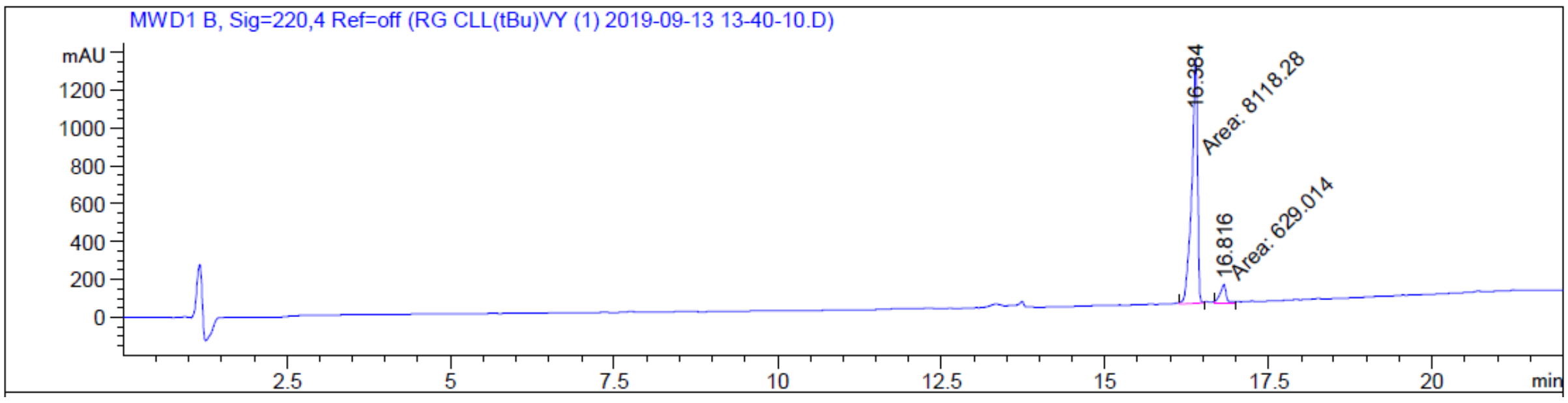

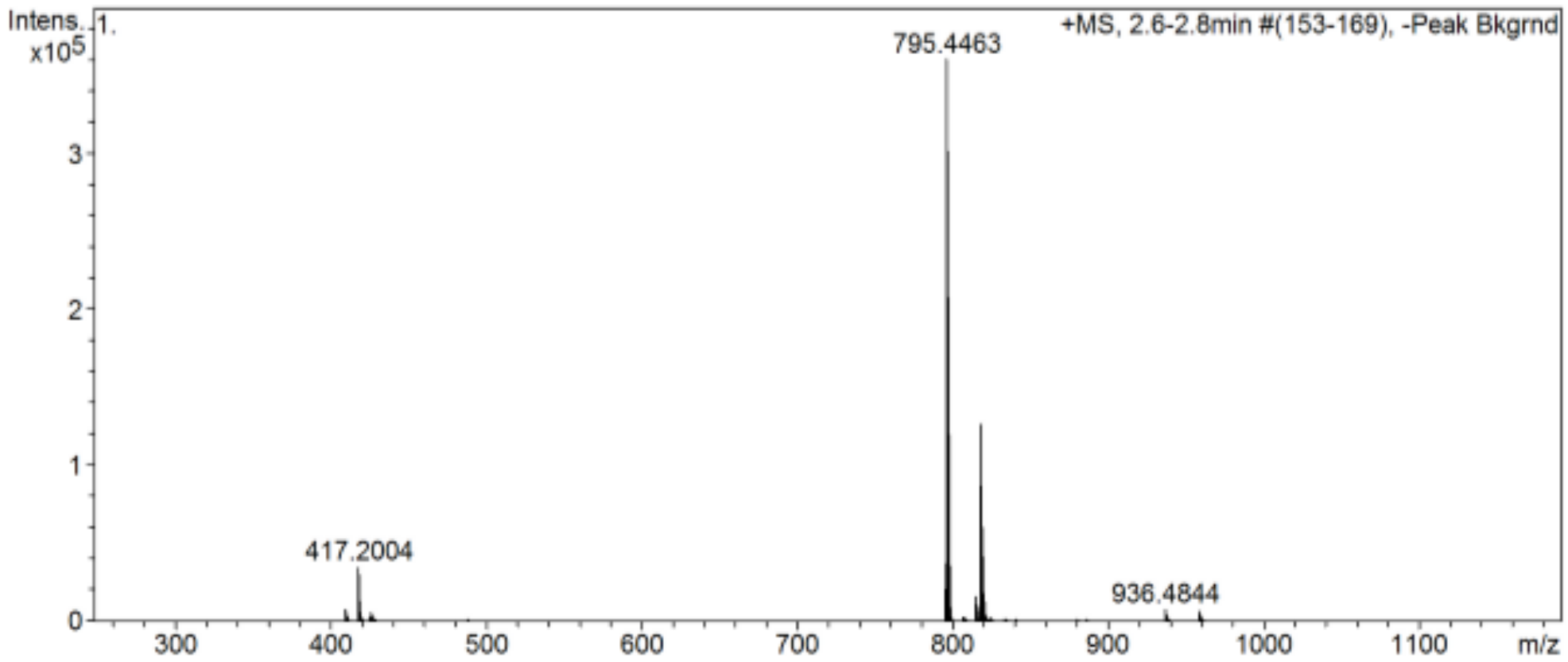

cyclo-CLLF(4-NO_2)VY (**26**)

cyclo-CLLF(4-NO_2)VY was obtained as a white solid (11.5 mg, 10.1 % overall yield).

Analytical HPLC: R_t = 13.580 min (93 % purity).

m/z (ESI^+): 784.8 $[M+H]^+$ (100 %).

HRMS (ESI^+) found: 784.3695, $[M+H]^+$ calculated: 784.3698.

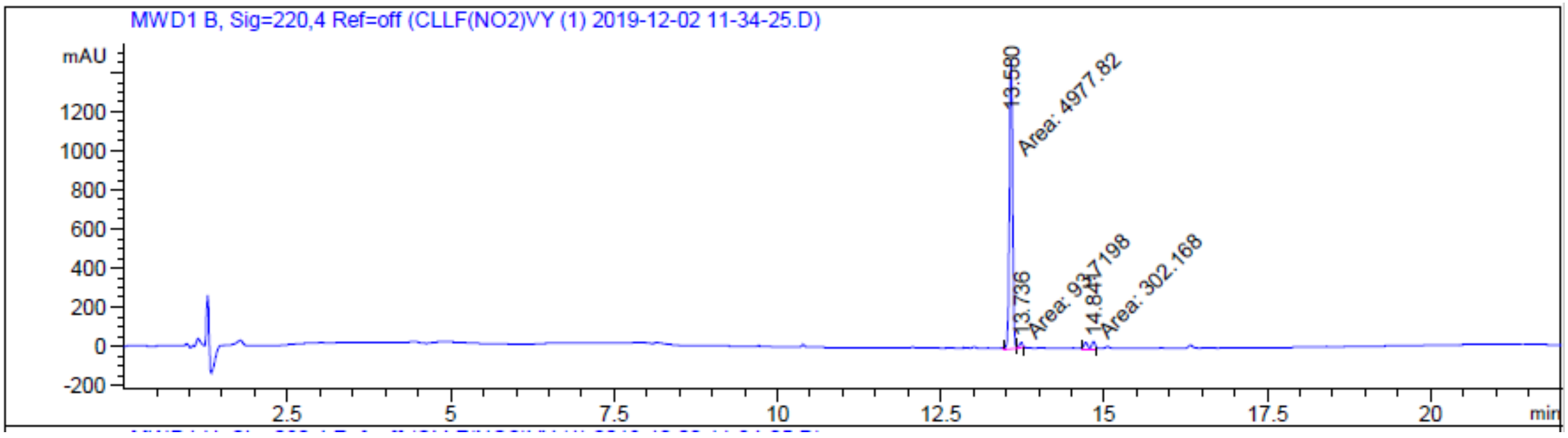

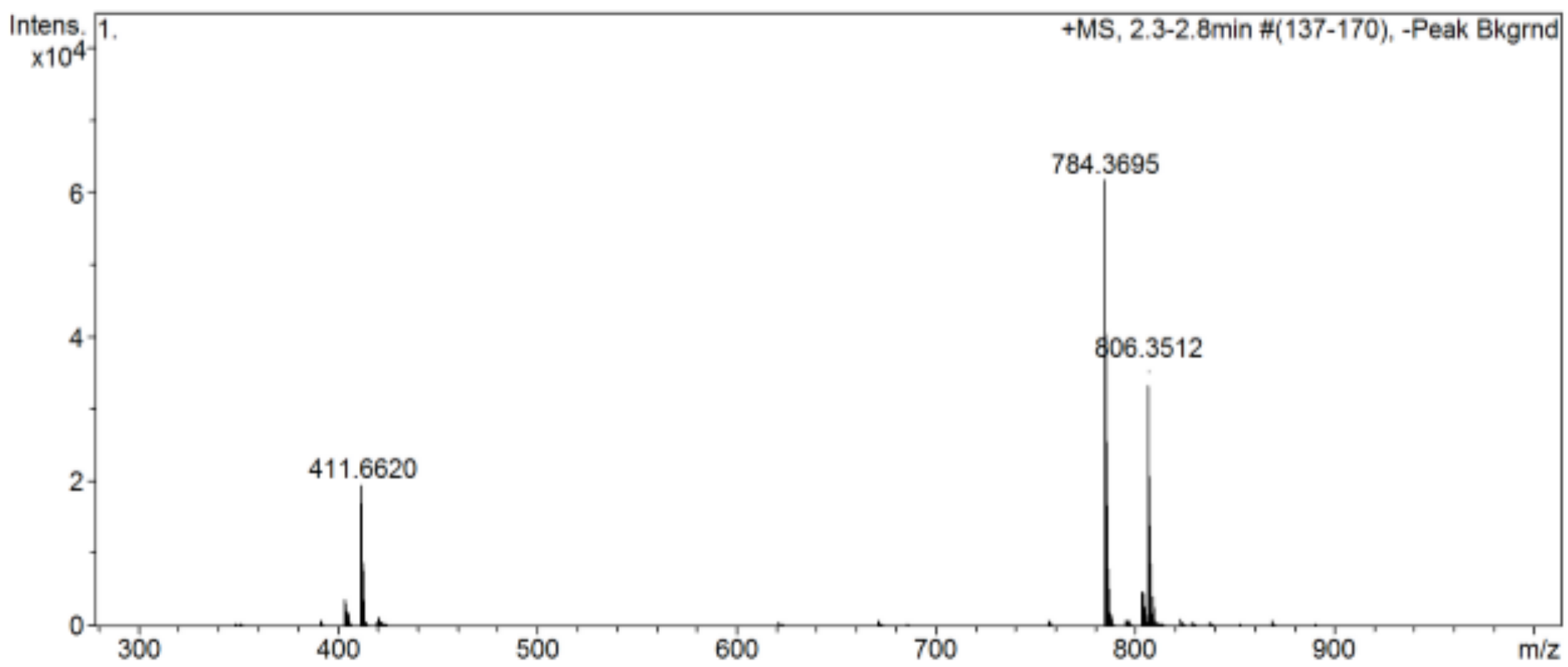

General protocol for *cyclo*-CLLF(CF_3)VY Tyrosine Derivatives

The linear resin bound sequence resin-LC-NH_2 was synthesised using Wang resin SPPS (section 8.6.1). The resin was then split equally into 10 syringes and swelled in a small amount of DMF. Standard SPPS protocols were then followed to yield the resin bound linear sequence resin-LCXVF(CF_3)L where X is the unnatural amino acid. The linear sequences were then cleaved from the resin, and cyclised using HATU, HOAt and DIPEA. The StBu group was then removed and the peptide purified via reverse-phase HPLC. Fractions were combined and concentrated under reduced pressure, then lyophilised to yield the desired cyclic peptides.

cyclo-CLLF(CF_3)VNal (**27**)

cyclo-CLLF(CF_3)VNal was obtained as white solid (31.9 mg, 19.0 % overall yield).

Analytical HPLC: R_t = 17.959 min (99 % purity).

m/z (ESI^+): 841.6 $[M+H]^+$ (100 %).

HRMS (ESI^+) found: 841.3919, $[M+H]^+$ calculated: 841.3929.

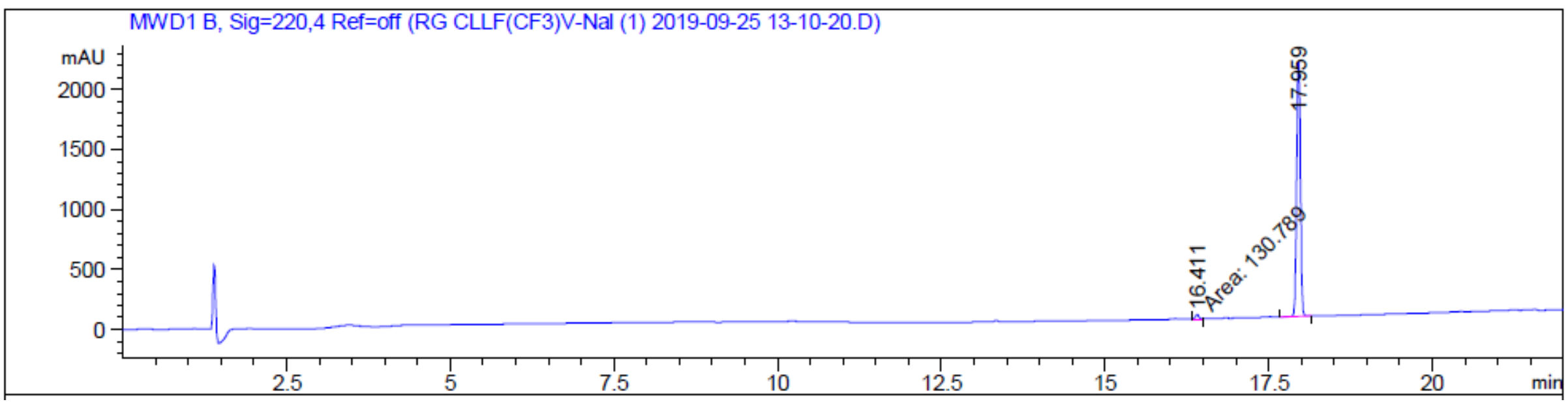

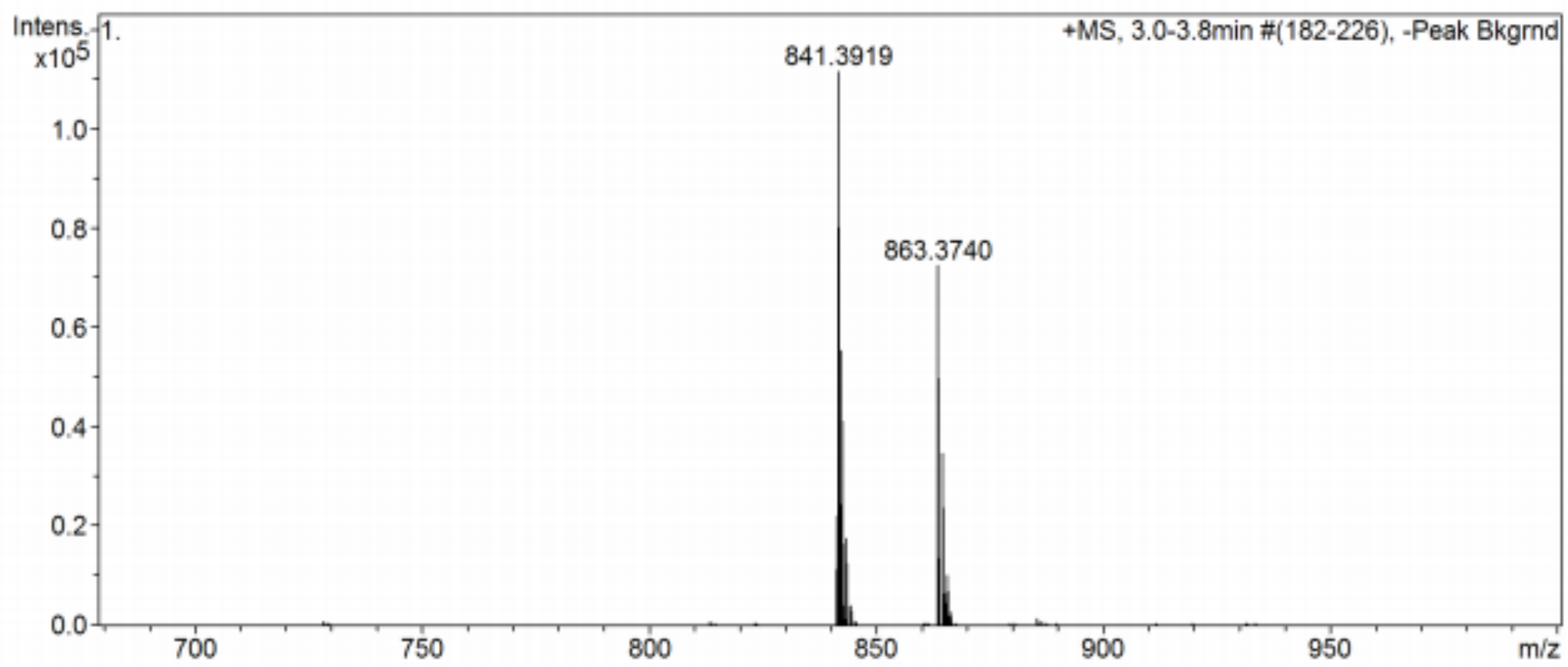

cyclo-CLLF(CF_3)VCha (**28**)

cyclo-CLLF(CF_3)VCha was obtained as a white solid (16.2 mg, 10.2 % overall yield).

Analytical HPLC: R_t = 18.111 min (97 % purity).
m/z (ESI^+): 797.7 $[M+H]^+$ (100 %).
HRMS (ESI^+) found: 797.4242, $[M+H]^+$ calculated: 797.4242.

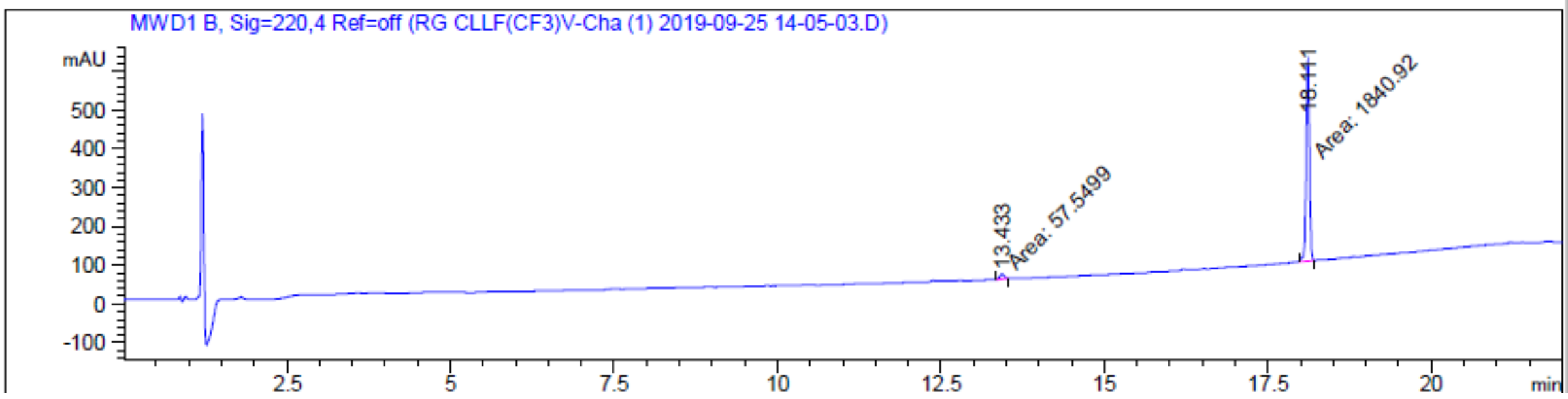

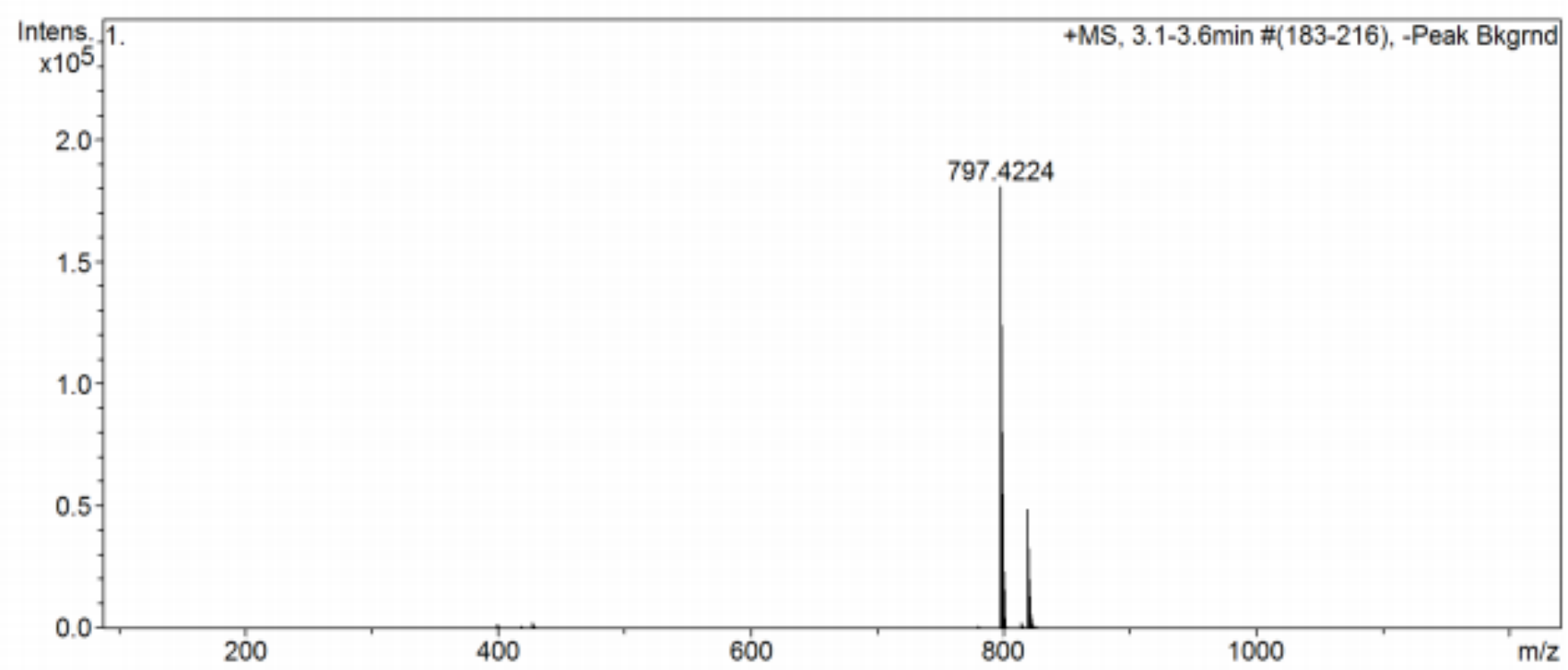

cyclo-CLLF(CF_3)VPhg (**29**)

cyclo-CLLF(CF_3)VPhg was obtained as a white solid (16.1 mg, 10.4 % overall yield).

Analytical HPLC: R_t = 16.544 min (92 % purity).
m/z (ESI^+): 777.7 $[M+H]^+$ (100 %).
HRMS (ESI^+) found: 777.3627, $[M+H]^+$ calculated: 777.3616.

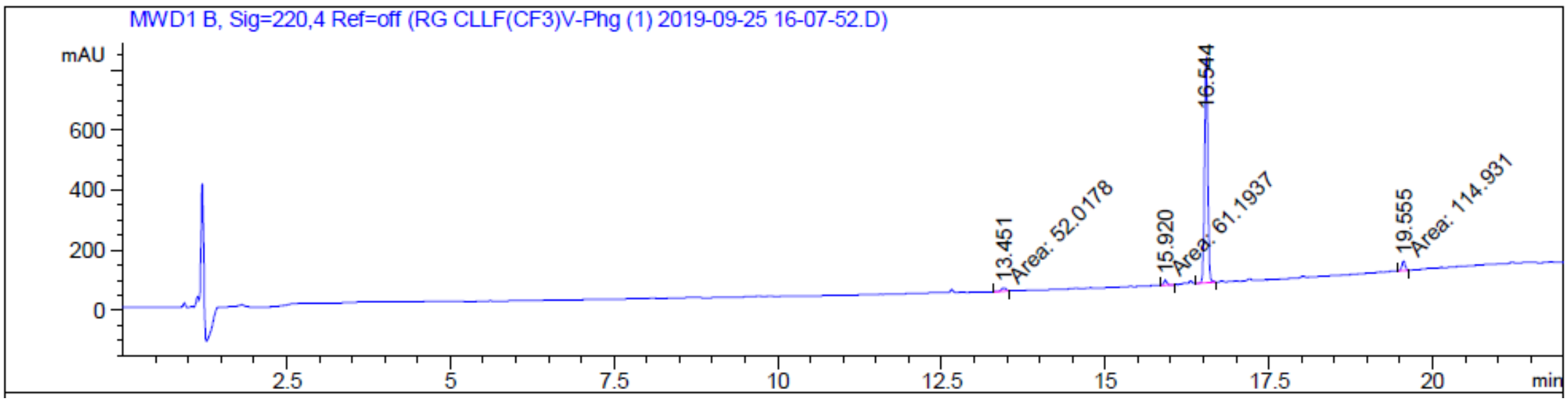

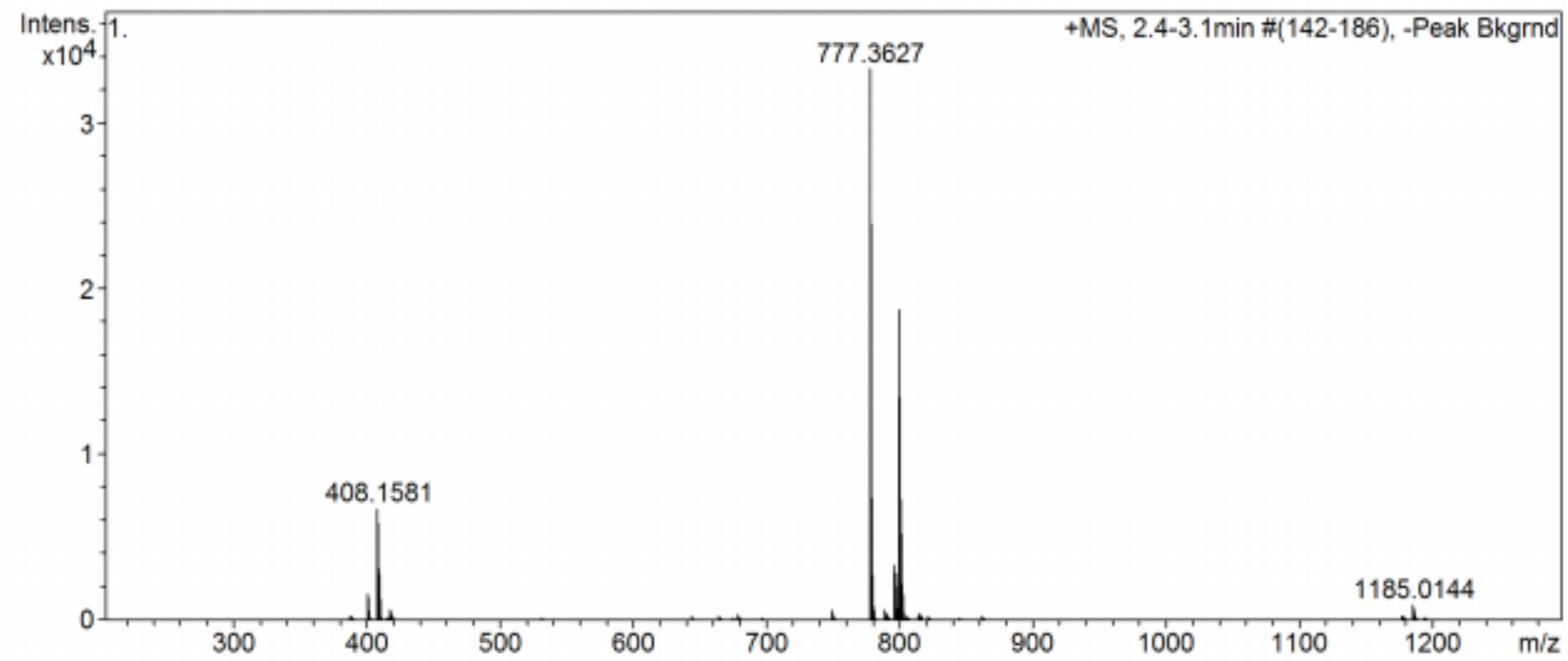

cyclo-CLLF(CF_3)VF (**30**)

cyclo-CLLF(CF_3)VF was obtained as a white solid (62.7 mg, 39.7 % overall yield).

Analytical HPLC: R_t = 16.769 min (95 % purity).
m/z (ESI^+): 791.6 $[M+H]^+$ (100 %), 813.6 $[M+Na]^+$ (20 %).
HRMS (ESI^+) found: 791.3779, $[M+H]^+$ calculated: 791.3772.

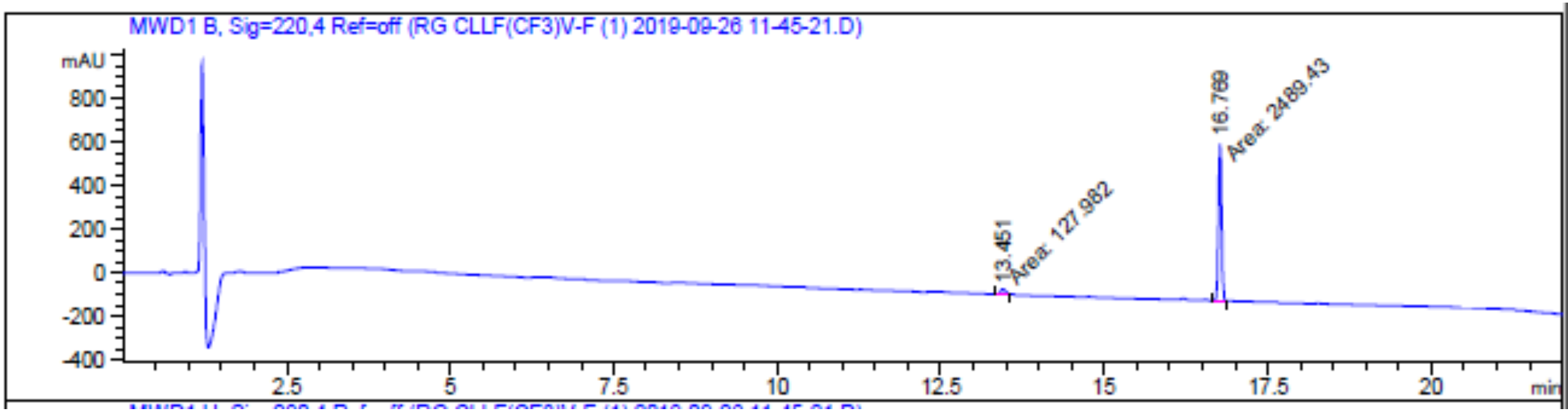

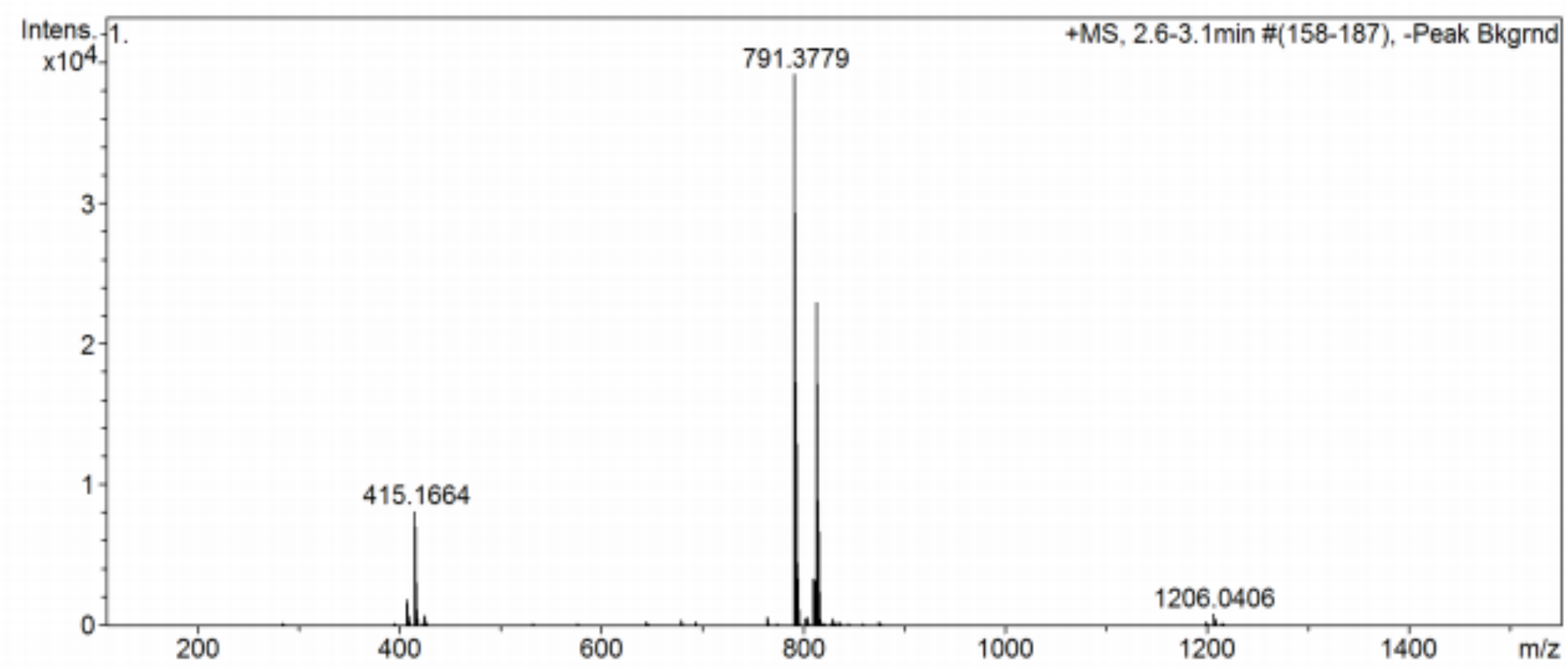

cyclo-CLLF(CF_3)V-Pal (**31**)

cyclo-CLLF(CF_3)V-Pal was obtained as a white solid (24.7 mg, 15.6 % overall yield).

Analytical HPLC: R_t = 12.770 min (97 % purity).

m/z (ESI^+): 792.6 $[M+H]^+$ (100 %).

HRMS (ESI^+) found: 792.3716, $[M+H]^+$ calculated: 792.3725.

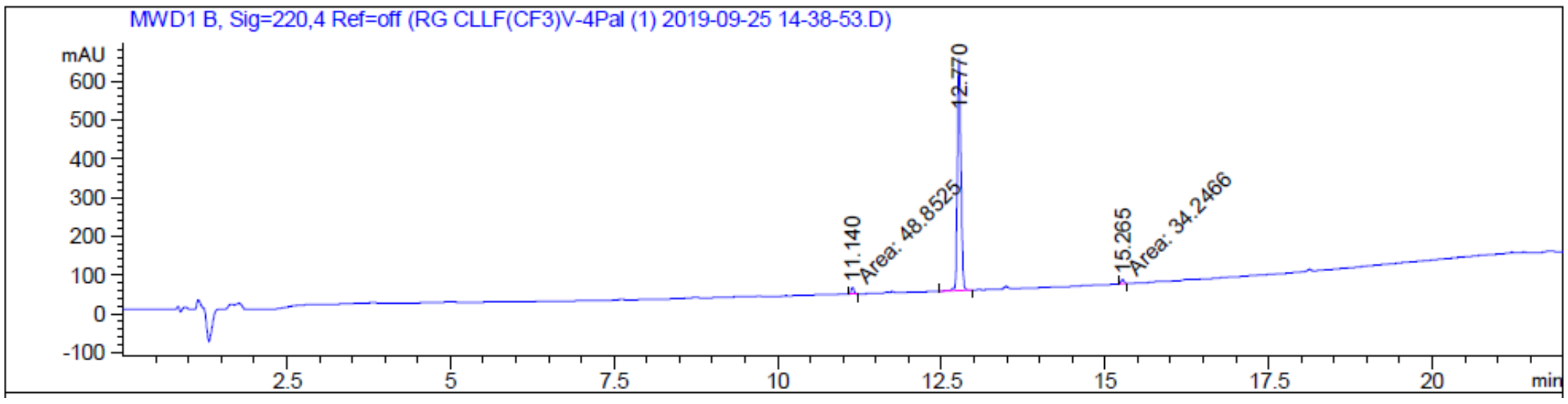

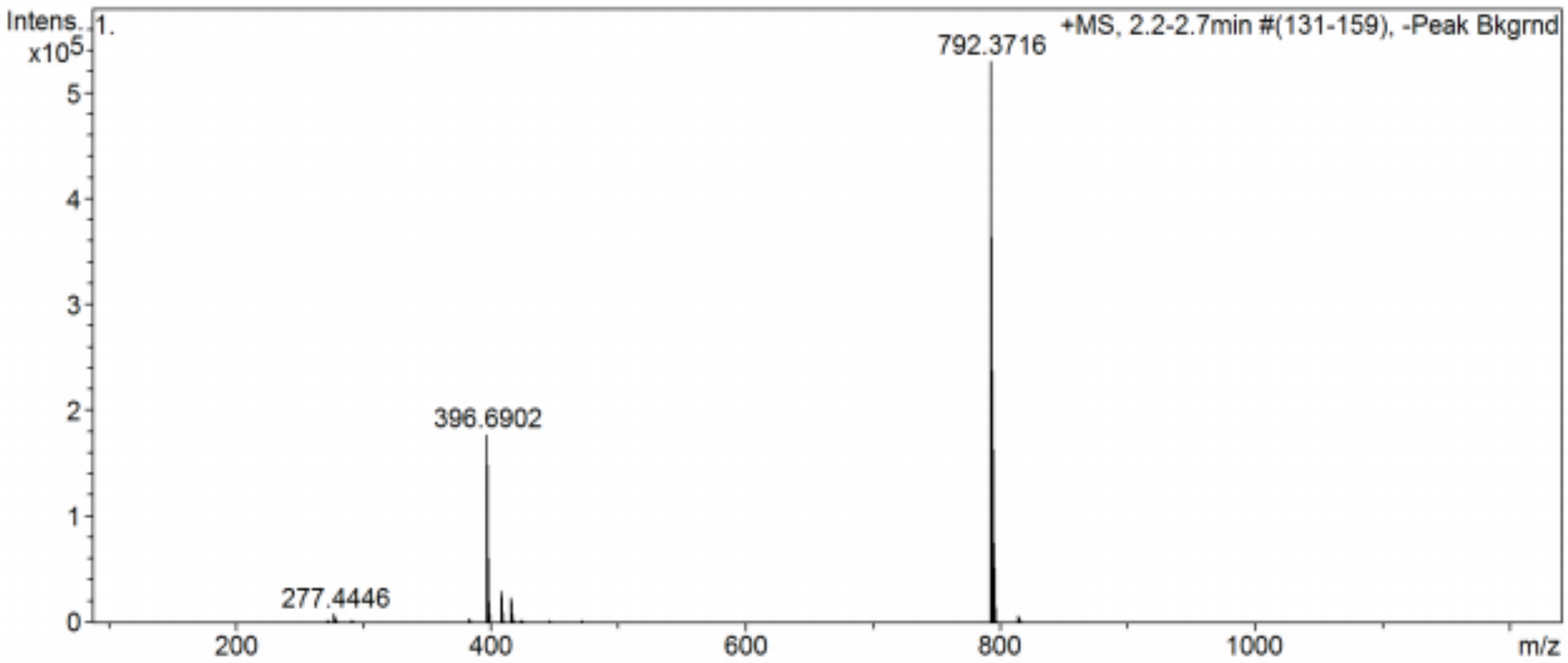

<u>*cyclo*-CLLF(CF_3)V(*p*-OMe)F(**32**)</u>

cyclo-CLLF(CF_3)V(*p*-OMe)F was obtained as a white solid (9.2 mg, 5.6 % overall yield).

Analytical HPLC: R_t = 16.557 min (99 % purity).
m/z (ESI^+): 821.7 $[M+H]^+$ (100 %).
HRMS (ESI^+) found: 821.3866, $[M+H]^+$ calculated: 821.3878.

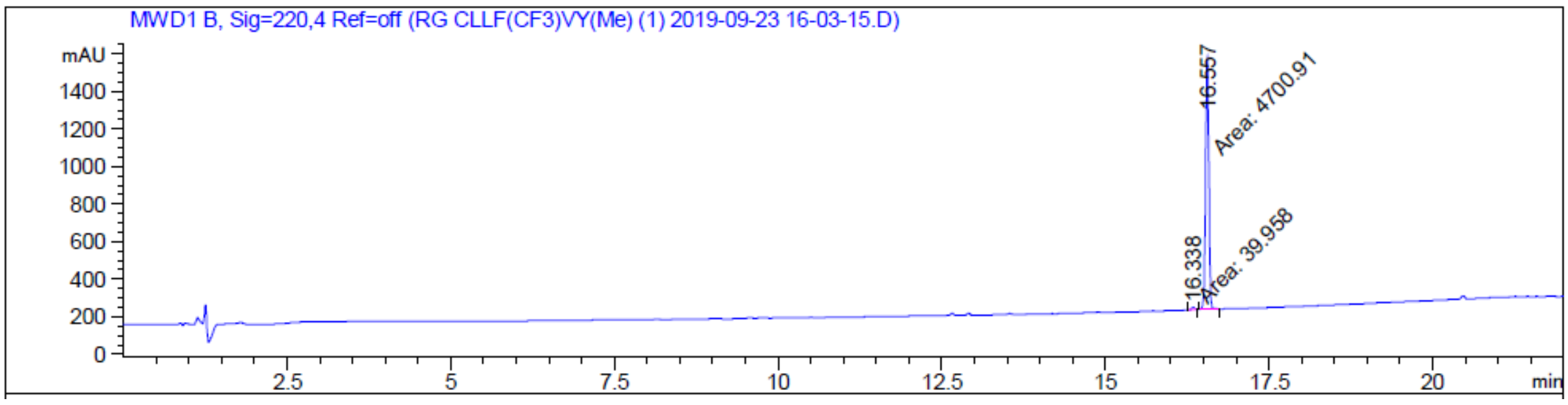

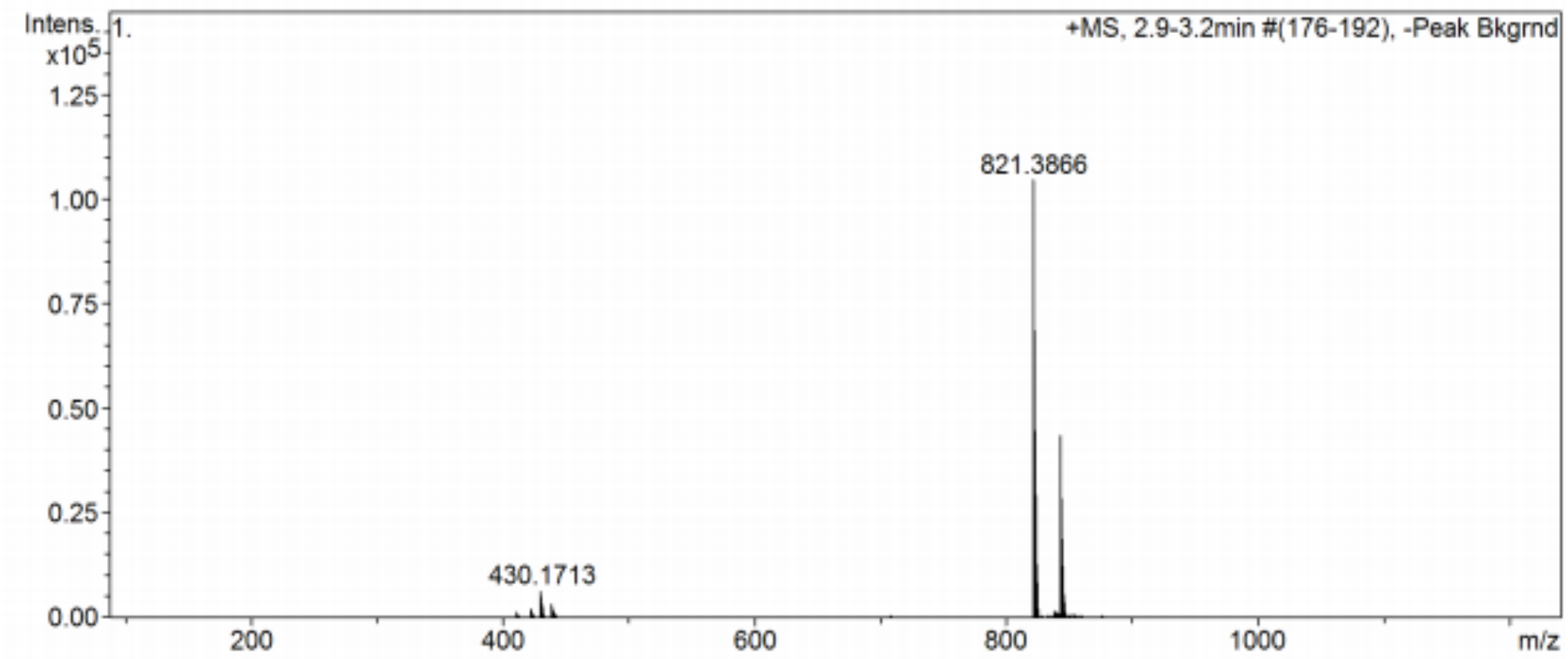

cyclo-CLLF(CF_3)V(*p*-F)F (**33**)

cyclo-CLLF(CF_3)V(*p*-F)F was obtained as a white solid (28.4 mg, 17.6 % overall yield).

Analytical HPLC: R_t = 16.665 min (95 % purity).
m/z (ESI^+): 809.7 $[M+H]^+$ (100 %).
HRMS (ESI^+) found: 809.3672, $[M+H]^+$ calculated: 809.3678.

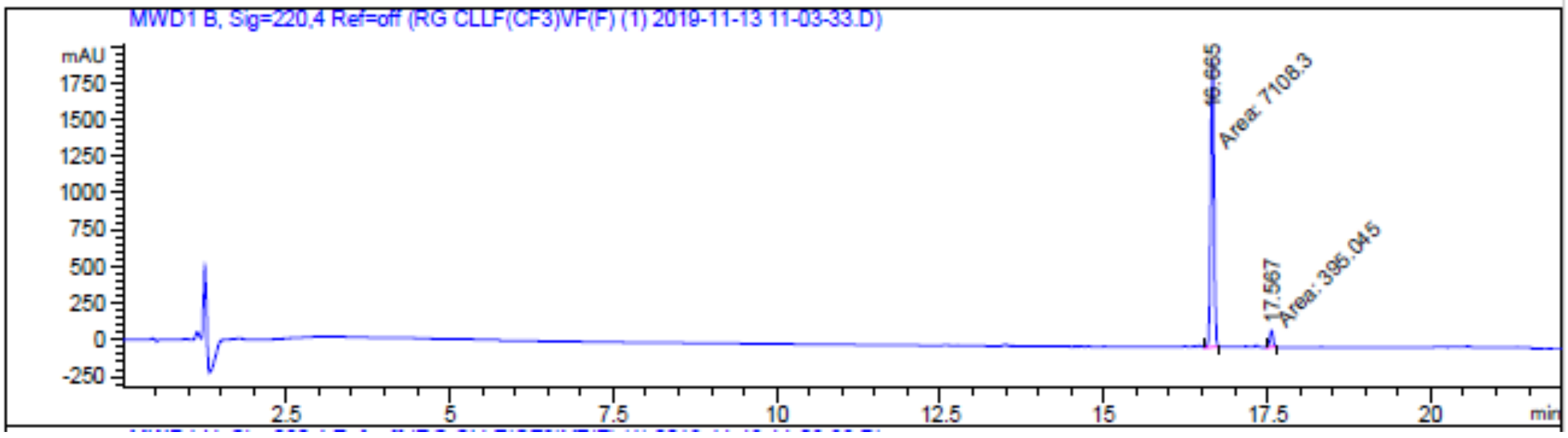

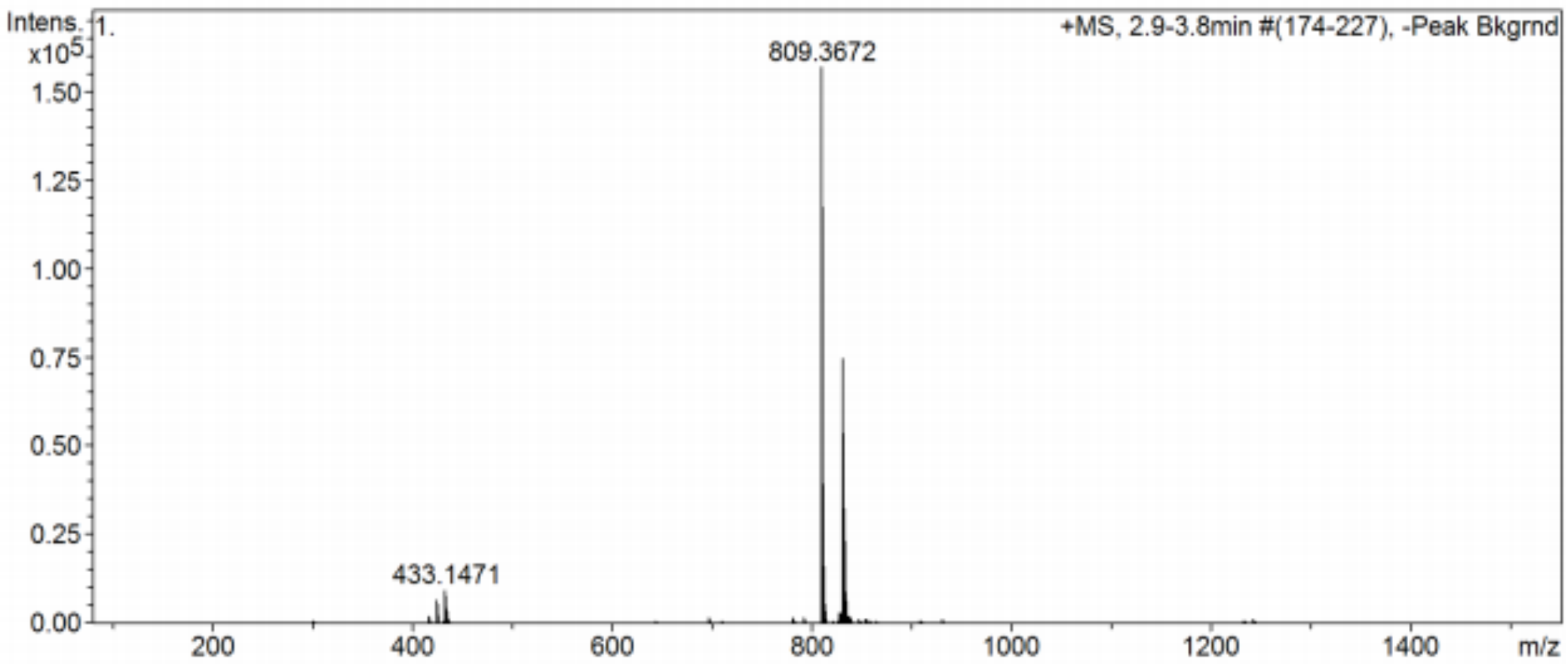

<u>*cyclo*-CLLF(CF_3)V(*p*-Cl)F (**34**)</u>

cyclo-CLLF(CF_3)V(*p*-Cl)F was obtained as a white solid (28.4 mg, 17.2 % overall yield).

Analytical HPLC: R_t = 17.376 min (99 % purity).
m/z (ESI^+): 825.6 $[M+H]^+$ (100 %).
HRMS (ESI^+) found: 825.3382, $[M+H]^+$ calculated: 825.3382.

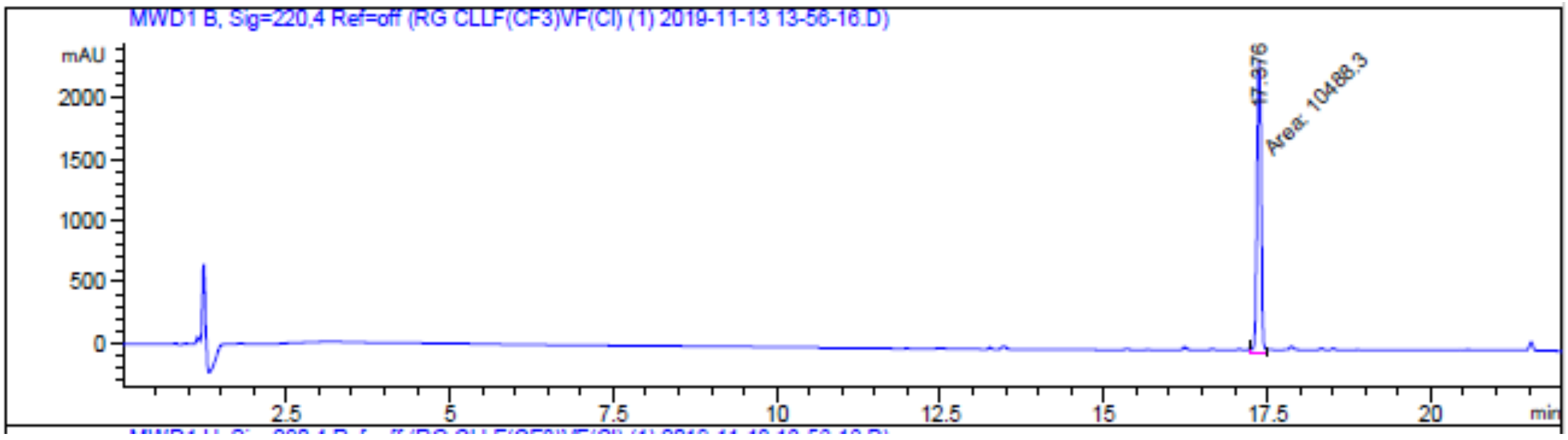

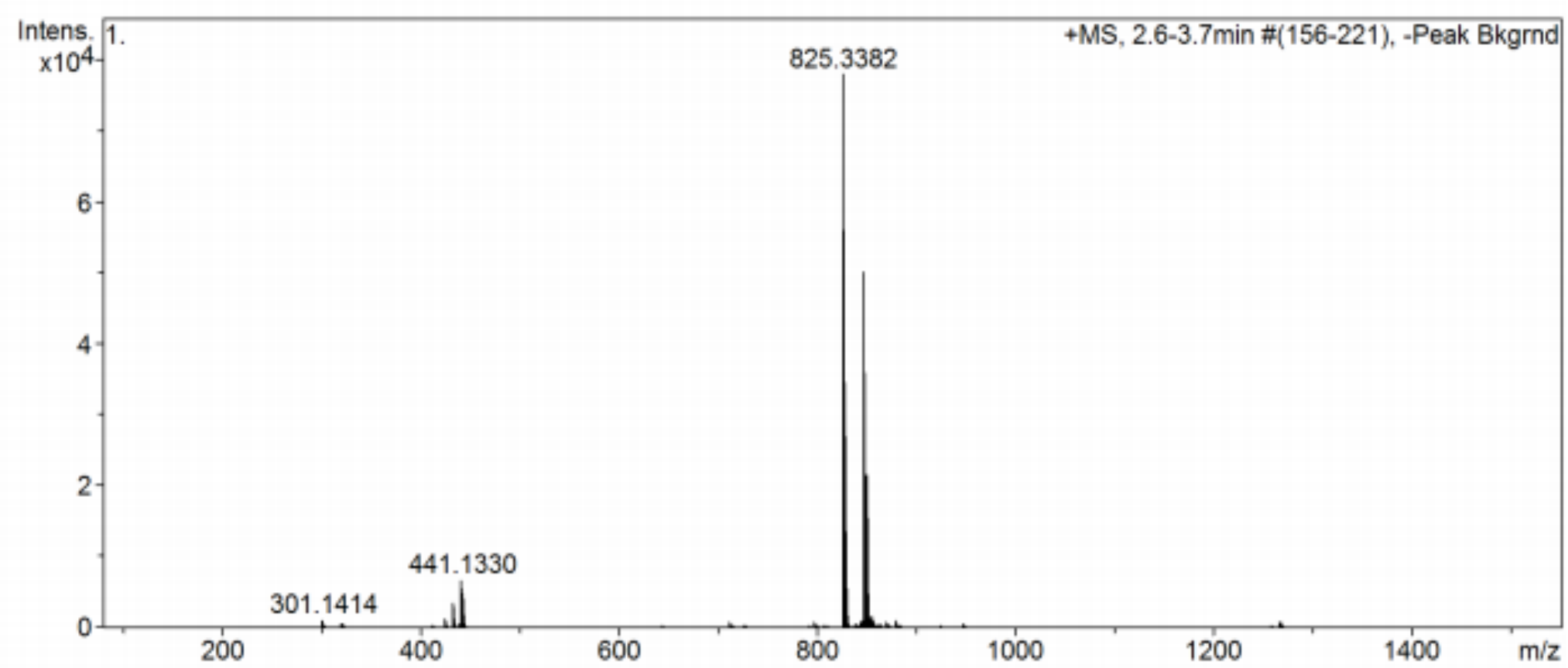

<u>*cyclo*-CLLF(CF_3)V(*p*-Br)F (**35**)</u>

cyclo-CLLF(CF_3)V(*p*-Br)F was obtained as a white solid (8.3 mg, 4.8 % overall yield).

Analytical HPLC: R_t = 17.580 min (99 % purity).
m/z (ESI^+): 871.6 $[M+H]^+$ (100 %).
HRMS (ESI^+) found: 869.2877, $[M+H]^+$ calculated: 869.2881.

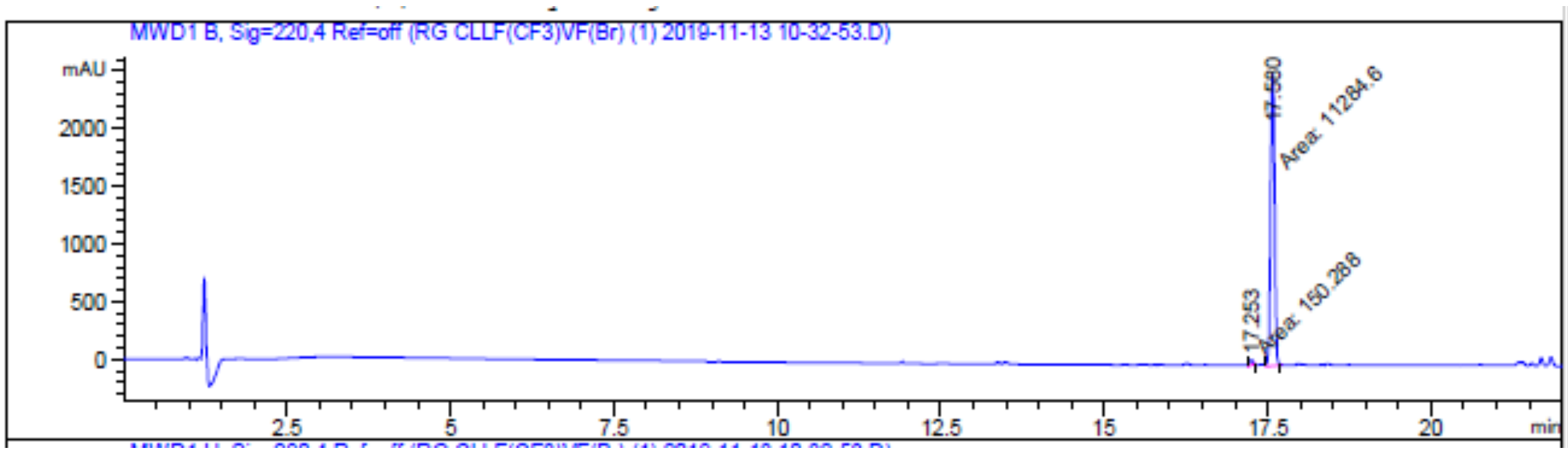

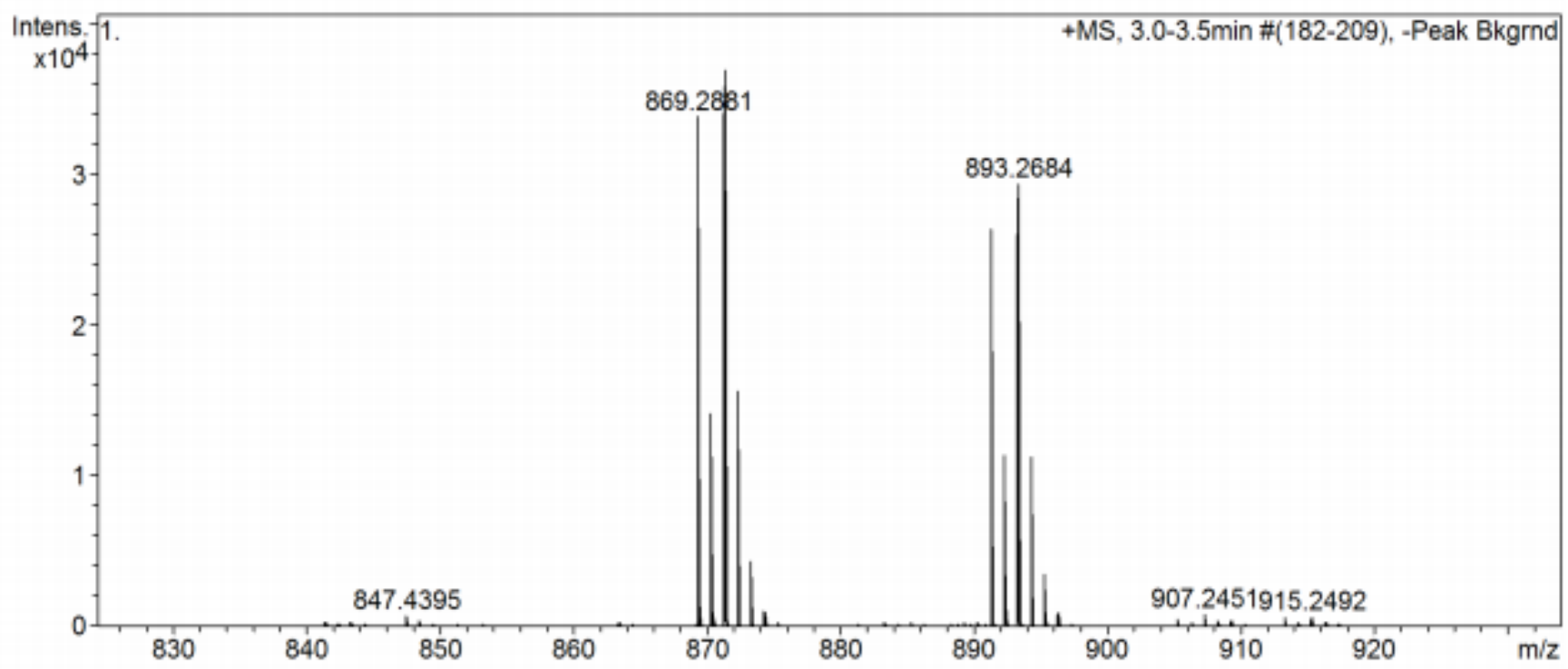

cyclo-CLLF(CF_3)V(*p*-I)F (**36**)

cyclo-CLLF(CF_3)V(*p*-I)F was obtained as a white solid (19.8 mg, 10.8 % overall yield).

Analytical HPLC: R_t = 17.893 min (91 % purity).
m/z (ESI^+): 917.7 $[M+H]^+$ (100 %).
HRMS (ESI^+) found: 917.2728, $[M+H]^+$ calculated: 917.2739.

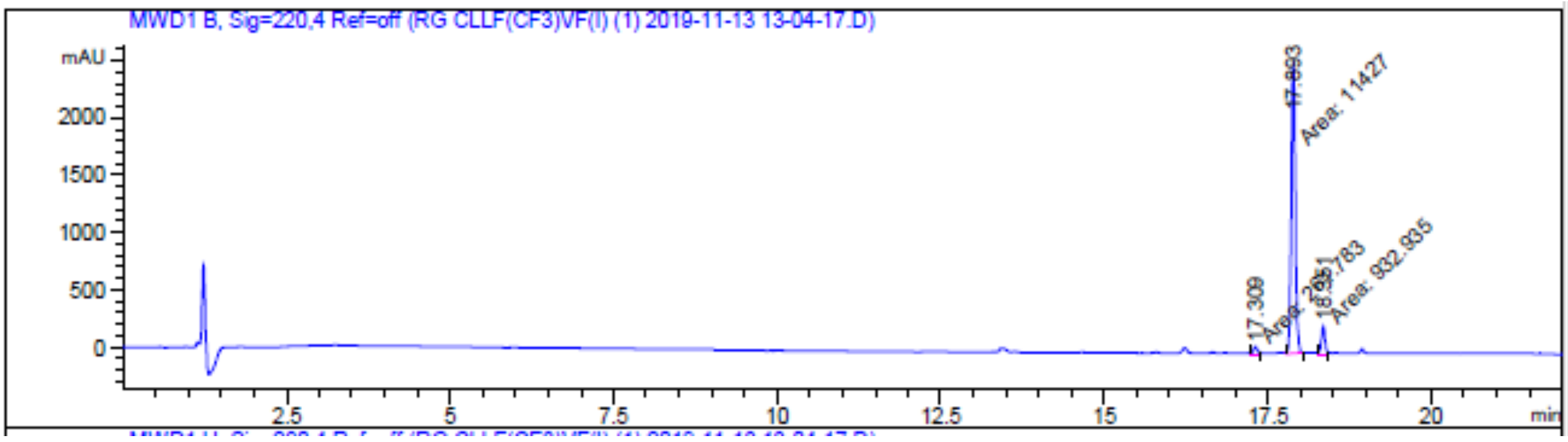

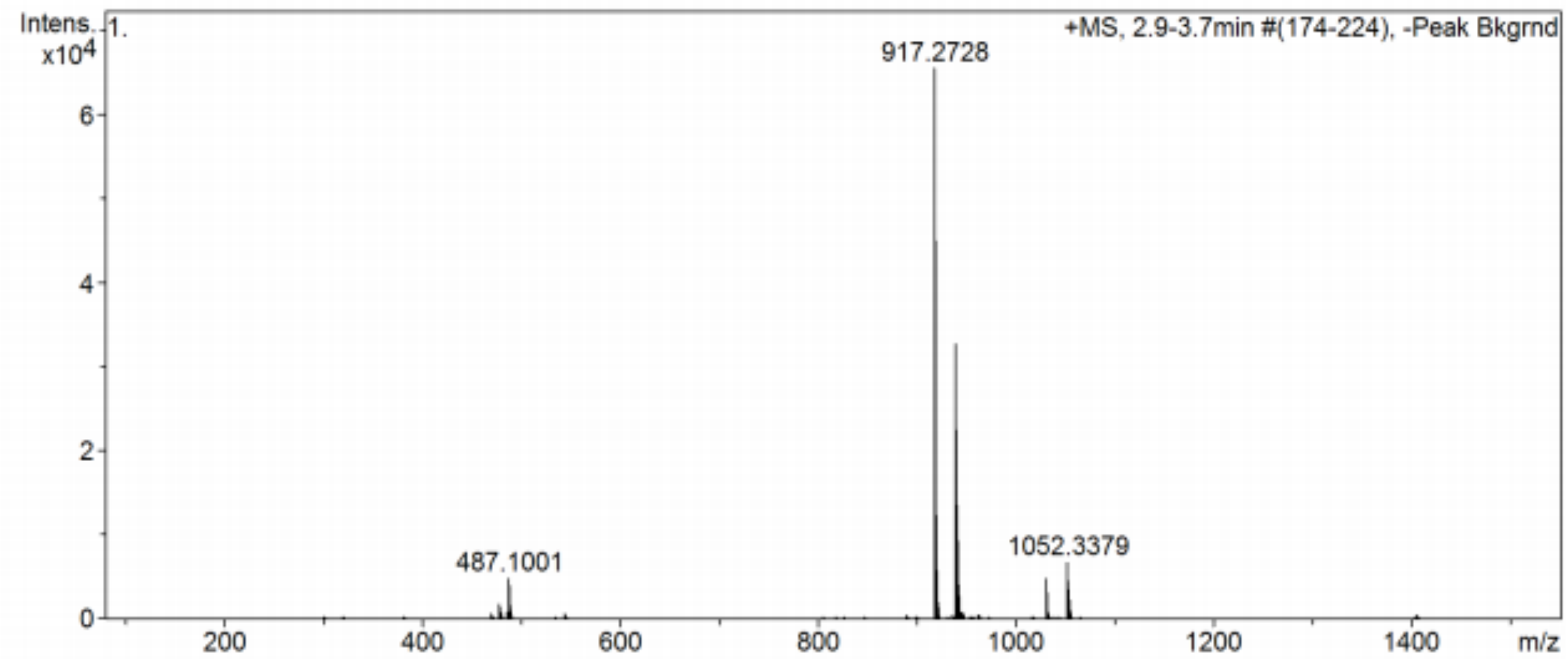

<u>*cyclo*-CLLF(CF_3)V(*p*-CF_3)F **(37)**</u>

cyclo-CLLF(CF_3)V(*p*-CF_3)F was obtained as a white solid (31.6 mg, 18.4 %).

Analytical HPLC: R_t = 16.284 min (94% purity).

m/z (ESI^+): 859.6 $[M+H]^+$.

HRMS (ESI^+) found: 859.3659, $[M+H]^+$ calculated: 859.3646.

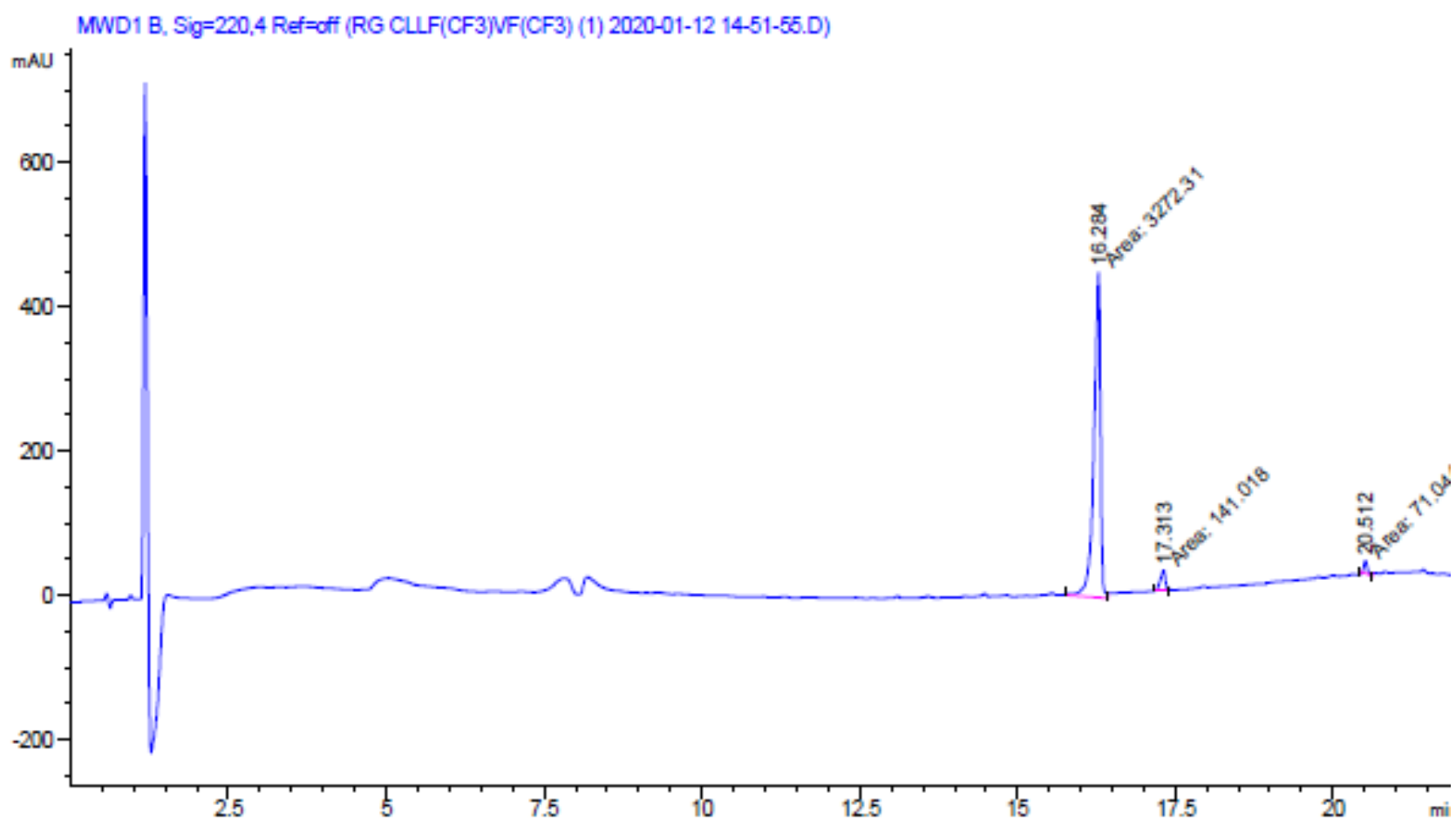

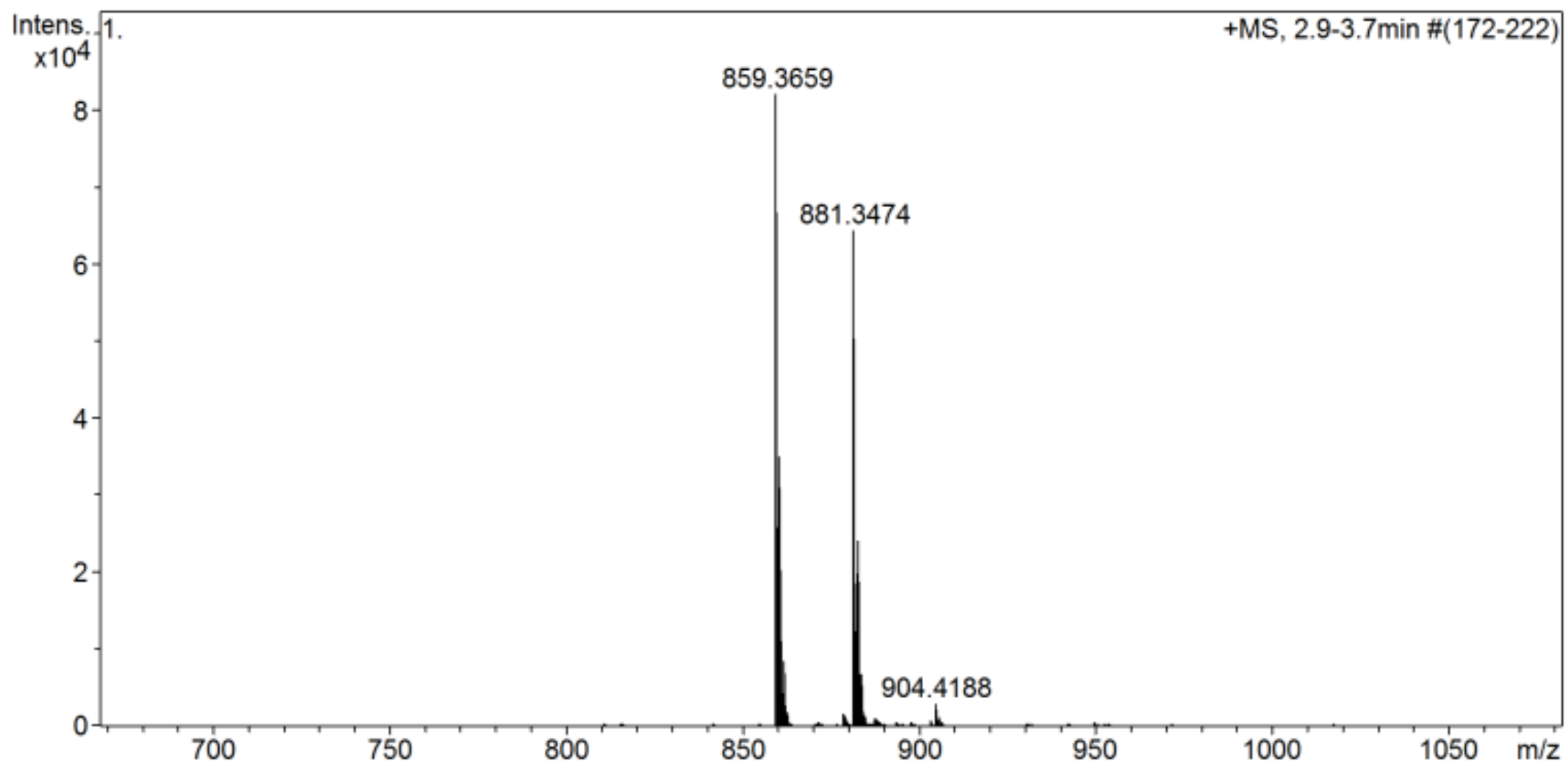

cyclo-CLLF(CF_3)V(*p*-Ph)F (**38**)

cyclo-CLLF(CF_3)V(*p*-Ph)F was obtained as a white solid (29.1 mg, 16.8 %).

Analytical HPLC: R_t = 18.351 min (98 % purity).

m/z (ESI^+): 867.7 $[M+H]^+$ (100 %), 889.7 $[M+Na]^+$ (30 %).

HRMS (ESI^+) found: 867.4078, $[M+H]^+$ calculated: 867.4085.

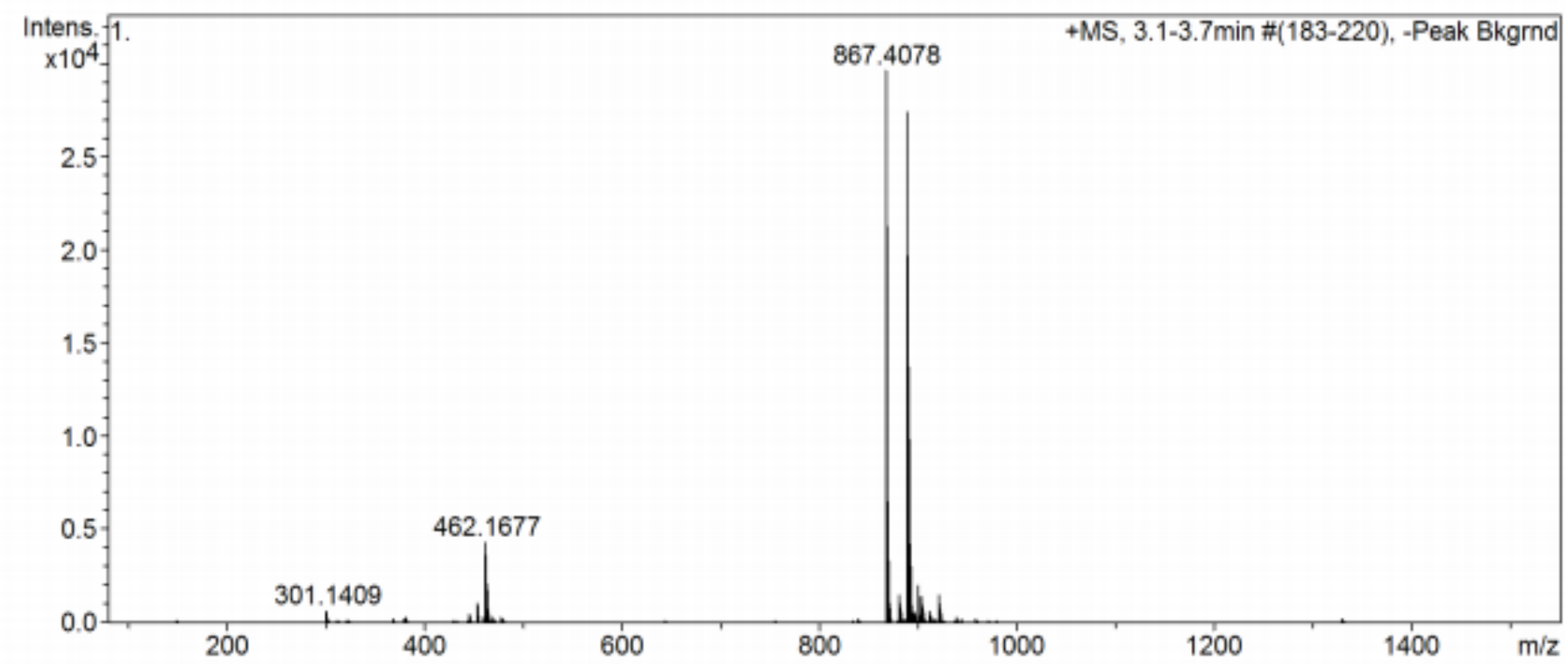

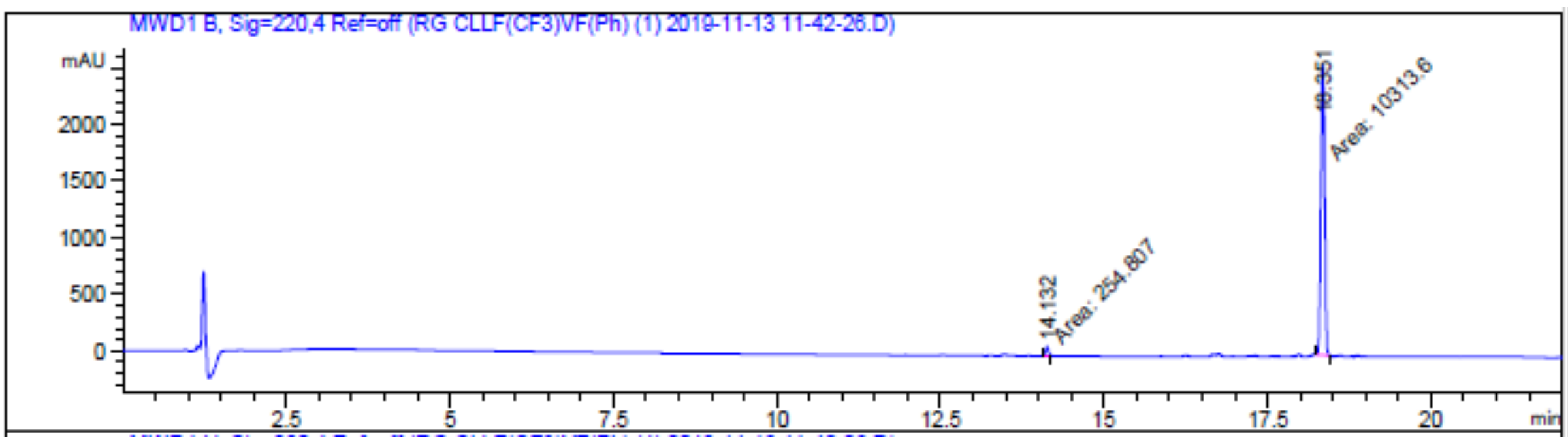

<u>*cyclo*-CLLF(CF_3)VF(*p*-tBu)F (**39**)</u>

cyclo-CLLF(CF_3)V(*p*-tBu)F was obtained as a white solid (24.1 mg, 14.2 %).

Analytical HPLC: R_t = 17.333 min 99 % purity).

m/z (ESI^+): 847.7 $[M+H]^+$ (100 %), 869.7 $[M+Na]^+$ (60 %).

HRMS (ESI^+) found: 847.4391, $[M+H]^+$ calculated: 847.4398.

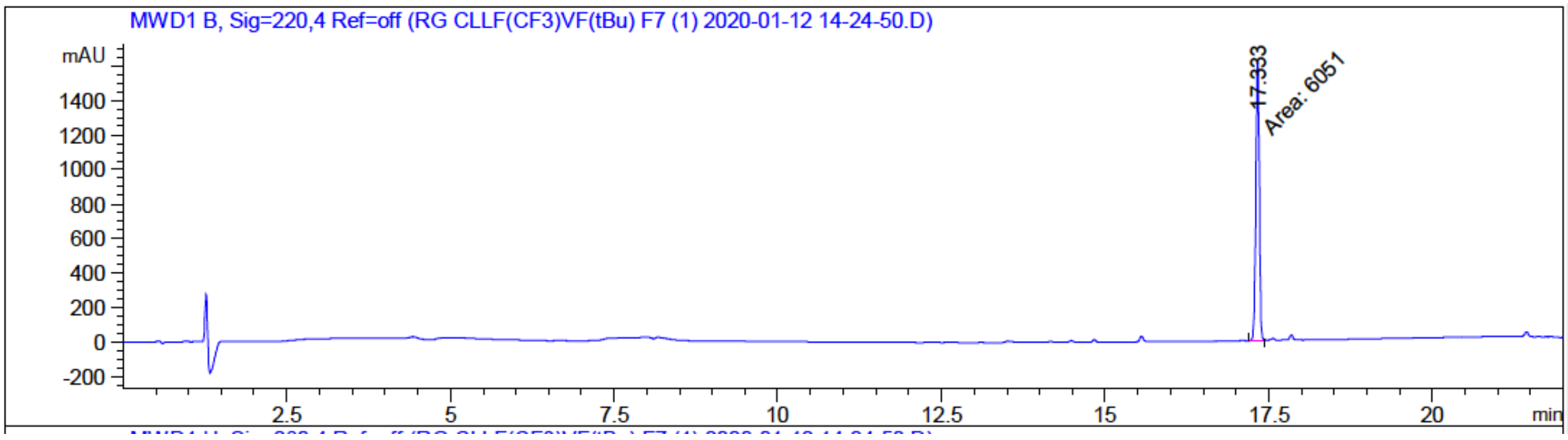

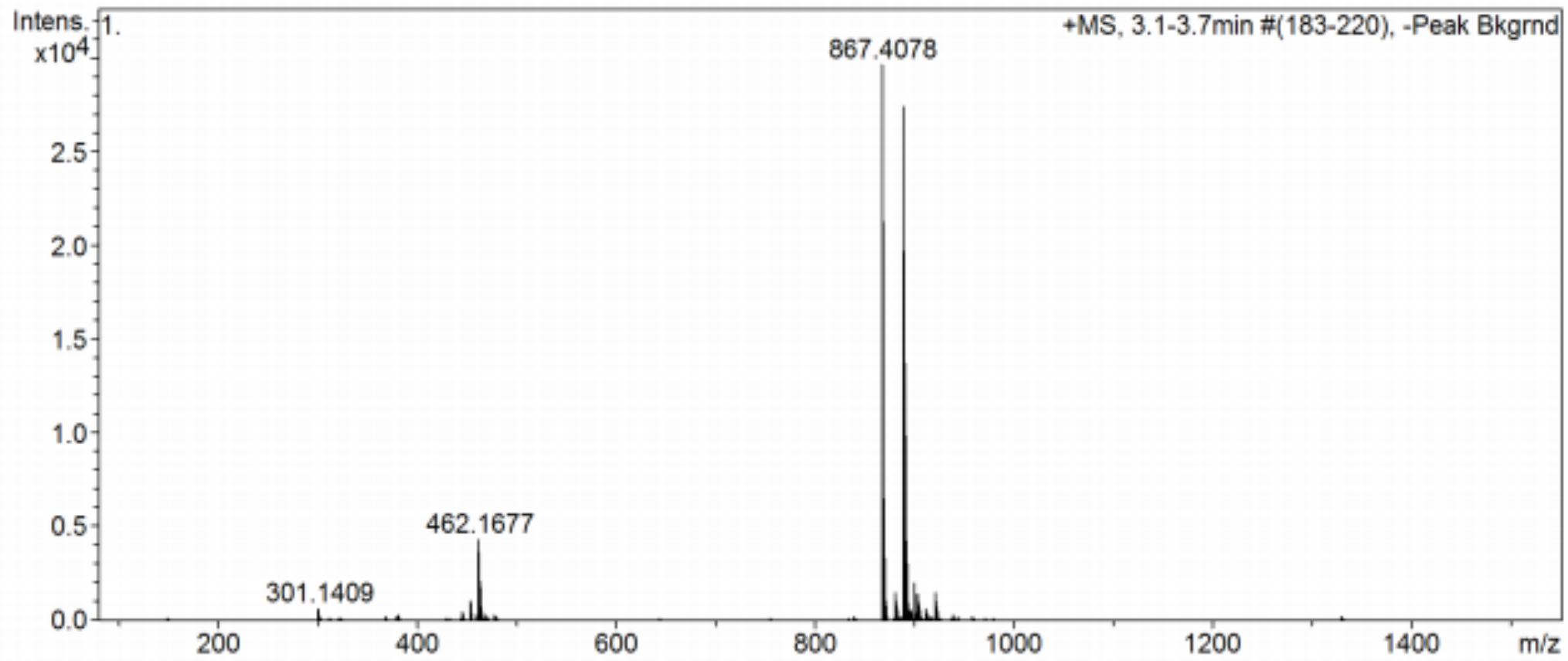

cyclo-CLLF(CF_3)VF(NO_2) (**40**)

Cyclo-CLLF(CF_3)VF(NO_2) was obtained as a white solid (20.0 mg, 12.0 %).

Analytical HPLC: R_t = 12.877 min (99 % purity).

m/z (ESI^+): 836.6 $[M+H]^+$ (100 %).

HRMS (ESI^+) found: 836.3616, $[M+H]^+$ calculated: 836.3623.

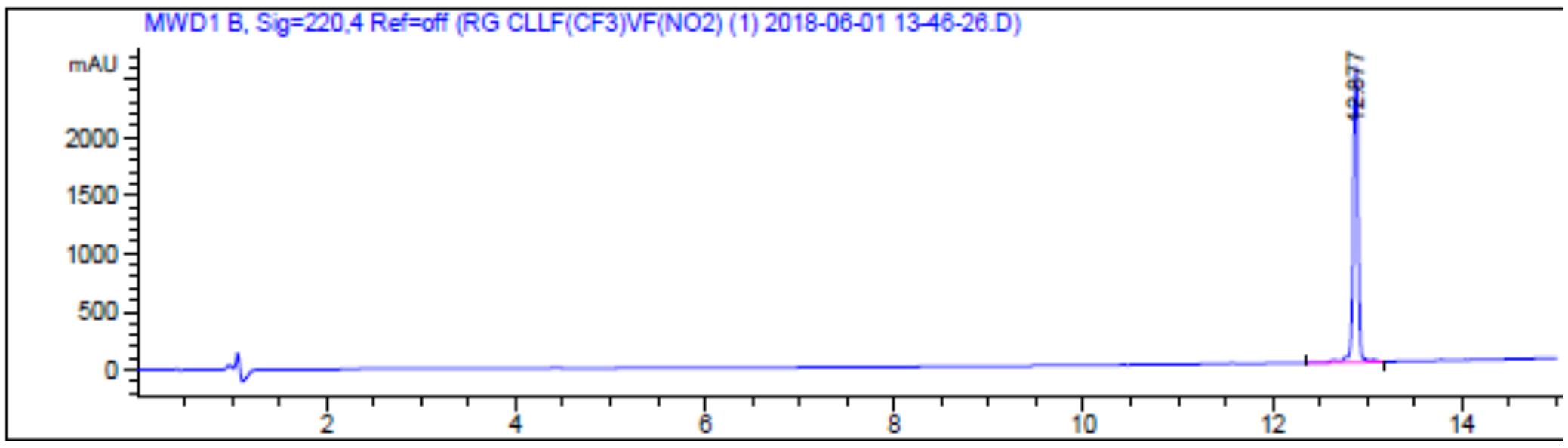

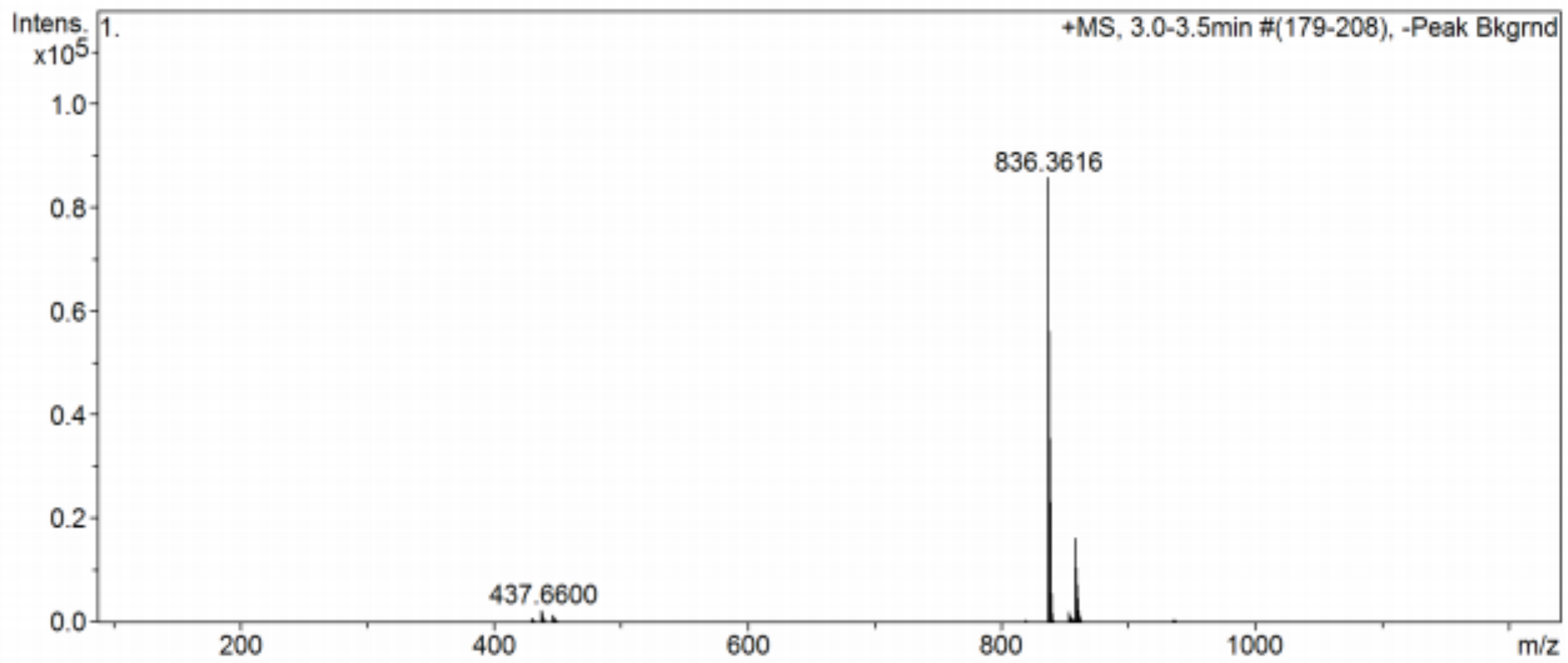

cyclo-CLLF(CF_3)VF(Br) Valine Derivatives

Fmoc-Leu-Wang resin (0.1mmol) was added to 8 syringes and the Fmoc group removed via exposure to 20 % piperidine in DMF. A solution of amino acid (1 eq.), Oxyma pure (3 eq.) and DIC (3 eq.) were added and the syringes placed on a rocker for 1 hour. The reaction mixtures were then removed via vacuum filtration and the resins washed with DMF x 2, DCM x 2 and Et_2O x 1. Fmoc protected amino acids were then deprotected via the addition of piperidine 20 % in DMF and being placed on a rocker for 30 minutes. The resins were then washed as before. These steps were repeated until the desired linear sequence was synthesised. The linear sequences were then cleaved from the resin using a TFA cleavage cocktail and cyclised using HATU and DIPEA. excess DMF was removed under reduced pressure and the crude cyclic peptide used without further purification. The StBu group was then removed and the peptide purified via reverse-phase HPLC, fractions were combined and concentrated under reduced pressure, then lyophilised to yield the desired cyclic peptides.

cyclo-CLLF(*p*-CF_3)FhL(*p*-Br)F (**41**)

cyclo-CLLF(*p*-CF_3)FhL(*p*-Br)F was obtained as a white solid (33.3 mg, 18.6 %).

Analytical HPLC: R_t = 18.577 min (99 % purity).
m/z (ESI$^+$): 899.7 $[M+H]^+$ (100 %).
HRMS (ESI$^+$) found: 897.3166, $[M+H]^+$ calculated: 897.3190.

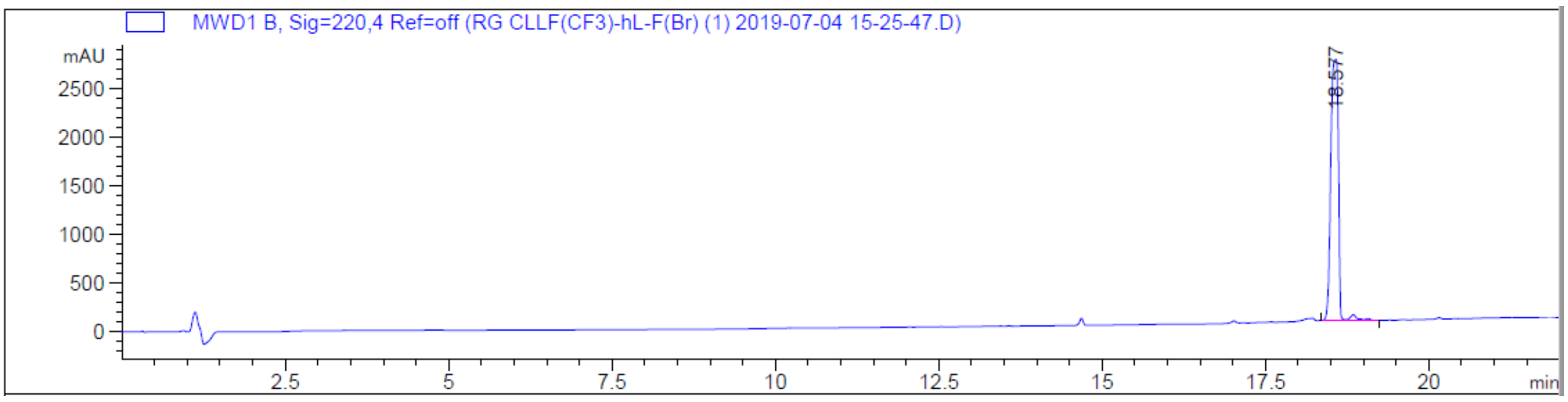

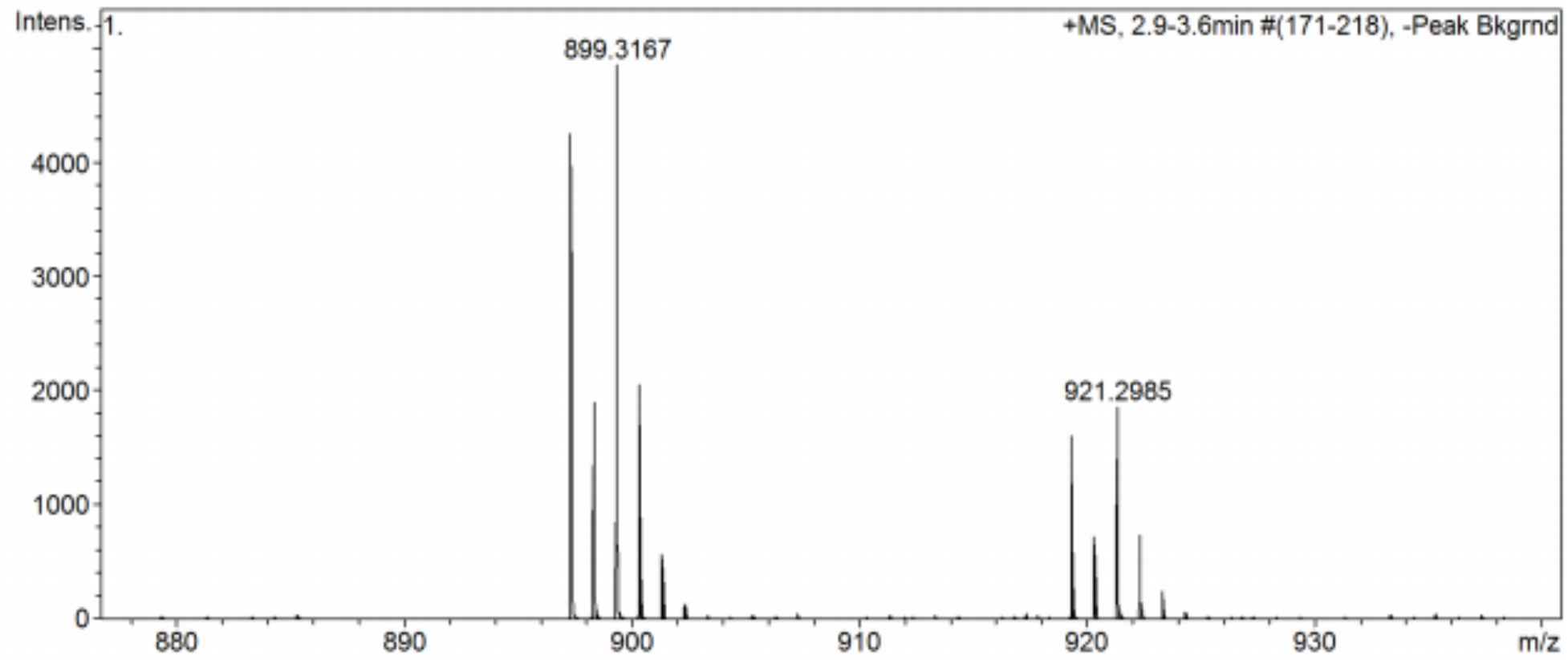

<u>*cyclo*-CLLF(*p*-CF_3)FNle(*p*-Br)F (**42**)</u>

cyclo-CLLF(*p*-CF_3)FNle(*p*-Br)F was obtained as a white solid (15.2 mg, 17.2 %).

Analytical HPLC: R_t = 18.107 min (95 % purity).
m/z (ESI$^+$): 885.5 $[M+H]^+$ (100 %), 905.4 $[M+Na]^+$ (60 %).
HRMS (ESI$^+$) found: 883.3034, $[M+H]^+$calculated: 883.3034.

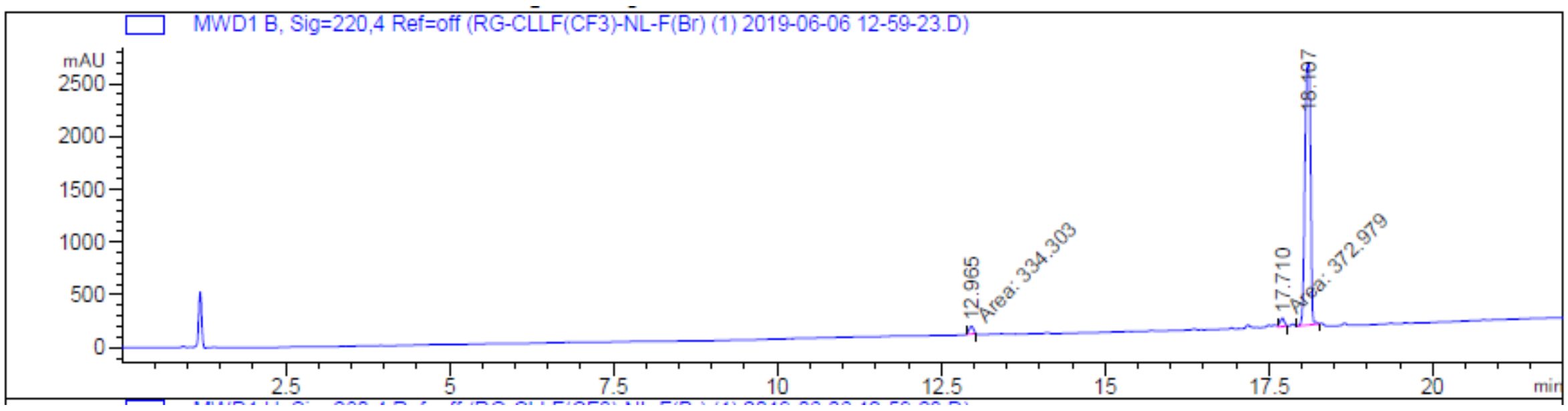

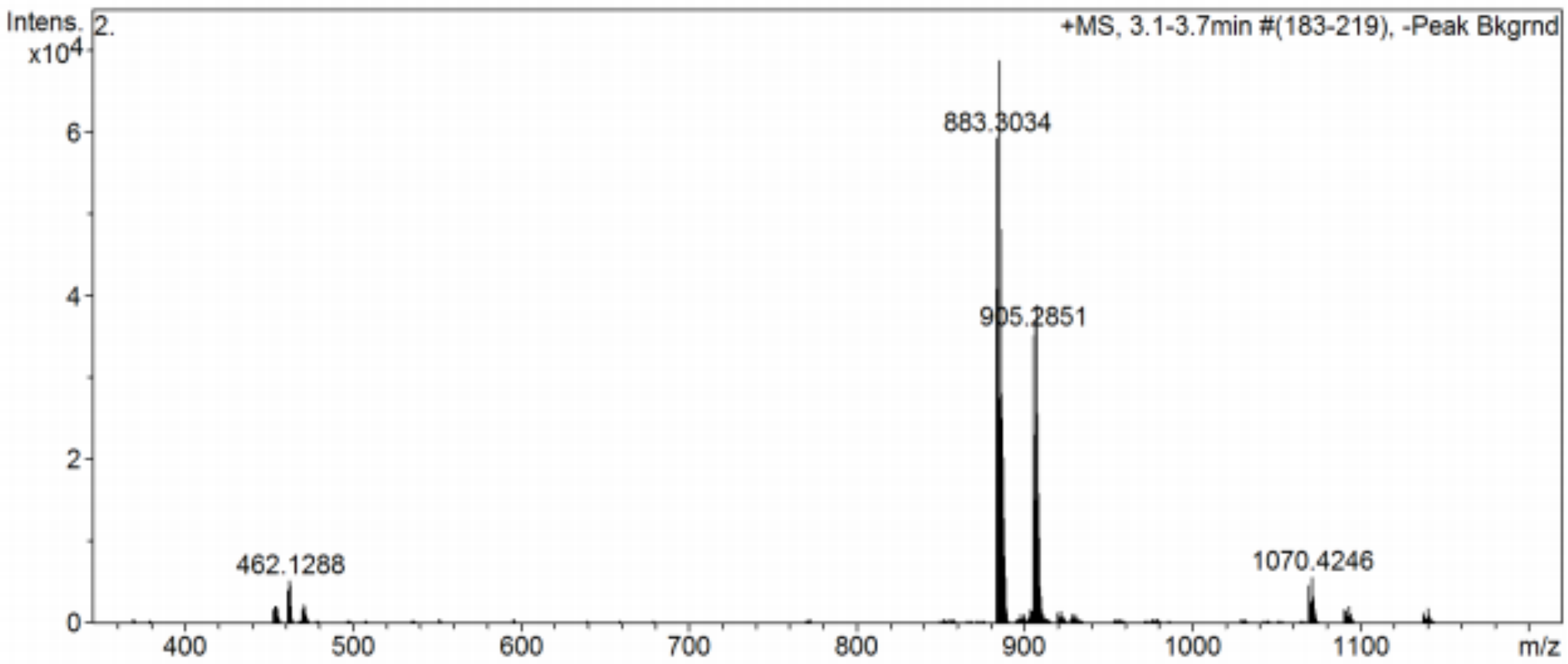

cyclo-CLLF(*p*-CF_3)FL(*p*-Br)F (**43**)

cyclo-CLLF(*p*-CF_3)FL(*p*-Br)F was obtained as a white solid (25.9 mg, 29.4 %).

Analytical HPLC: R_t = 17.873 min (99 % purity).
m/z (ESI$^+$): 885.5 $[M+H]^+$ (100 %).
HRMS (ESI$^+$) found: 905.2863, $[M+Na]^+$ calculated: 905.2859.

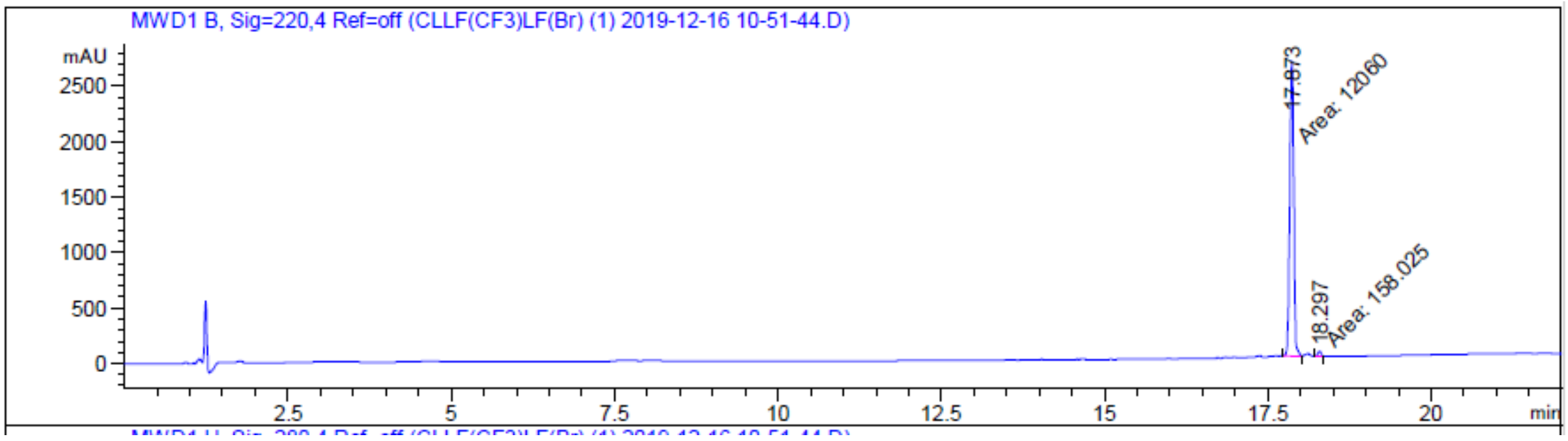

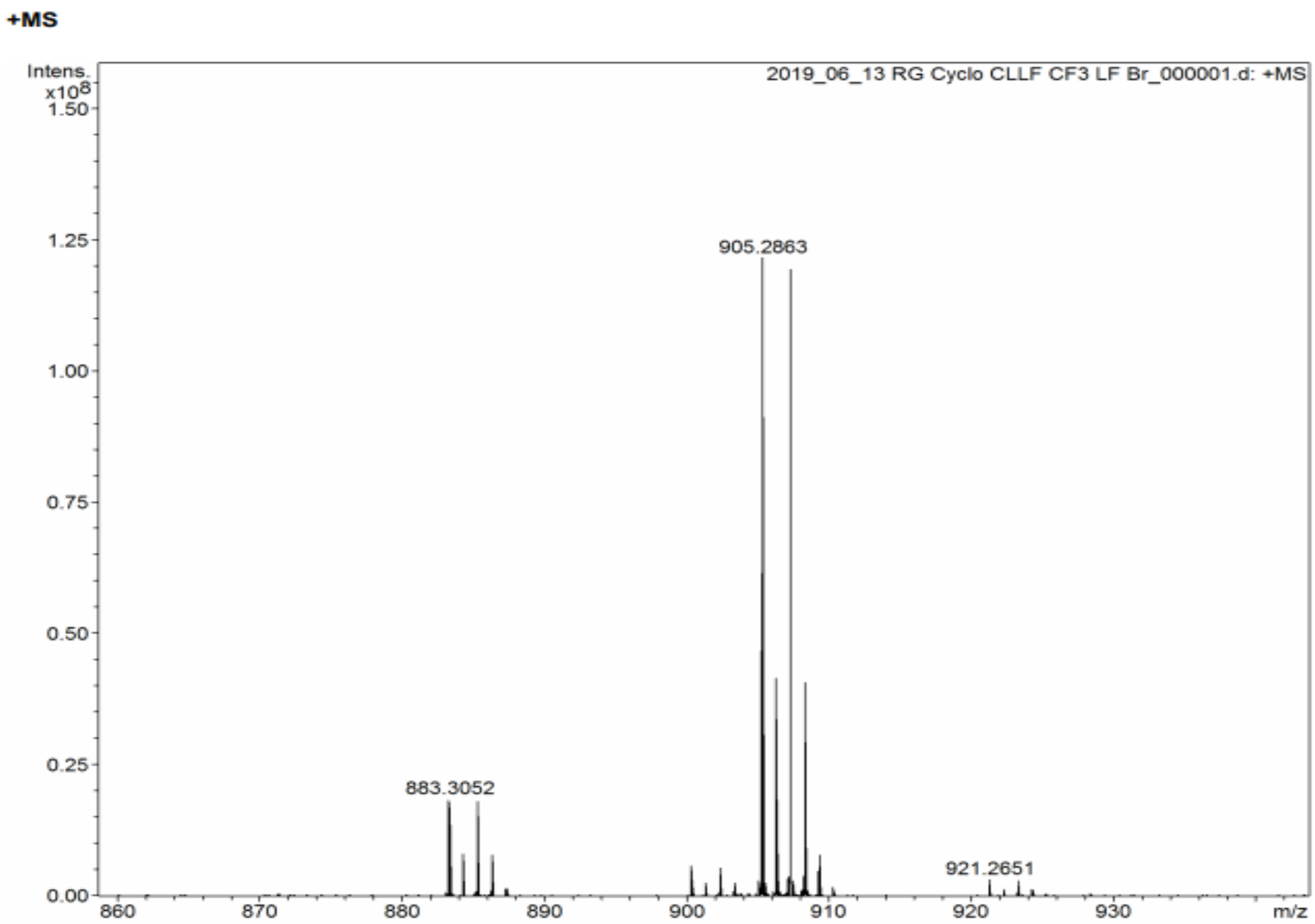

cyclo-CLLF(*p*-CF_3)FNva(*p*-Br)F (**44**)

cyclo-CLLF(*p*-CF_3)FNva(*p*-Br)F was obtained as a white solid (23.8 mg, 27.4 %).

Analytical HPLC: R_t = 17.340 min (98 % purity).

m/z (ESI^+): 893.5 $[M+Na]^+$ (100 %), 869.5 $[M+H]^+$ (90 %).

HRMS (ESI^+) found: 869.2873, $[M+H]^+$ calculated: 869.2877.

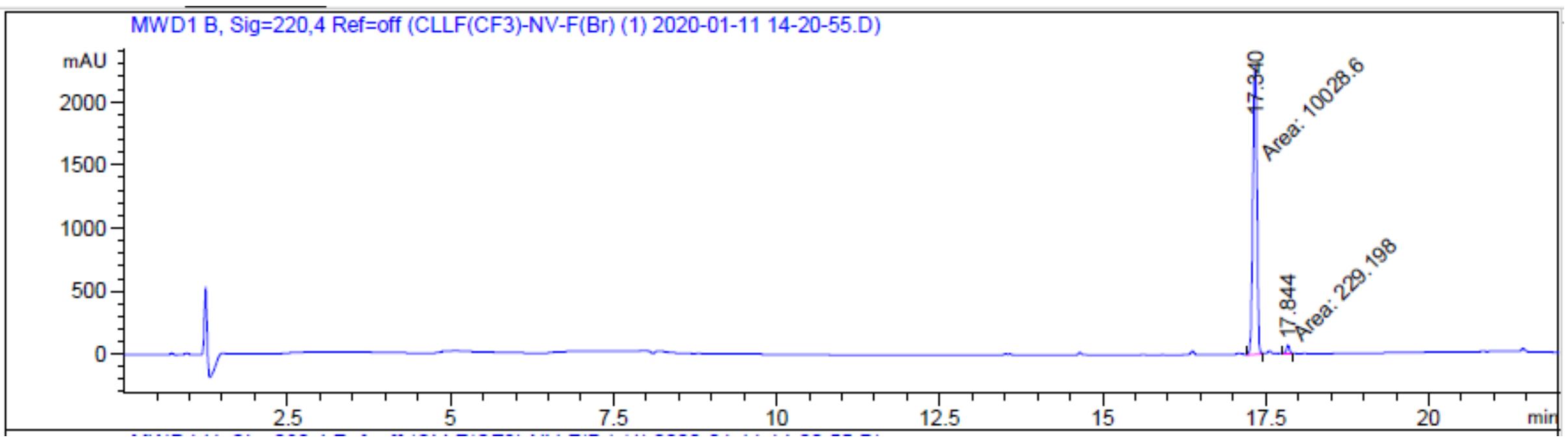

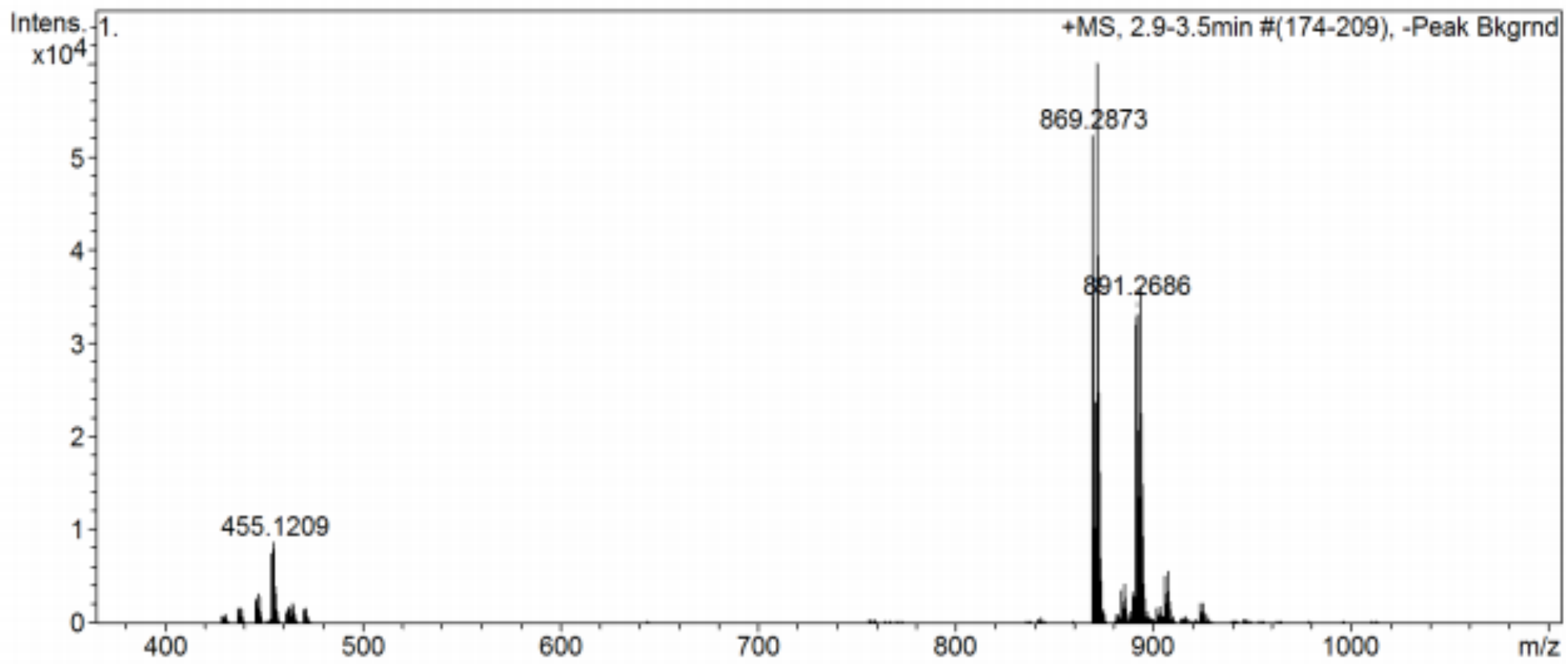

<u>*cyclo*-CLLF(*p*-CF_3)FAbu(*p*-Br)F (**45**)</u>

cyclo-CLLF(*p*-CF_3)FAbu(*p*-Br)F was obtained as a white solid (32.9 mg, 38.5 %).

Analytical HPLC: R_t = 16.609 min (99 % purity).
m/z (ESI^+): 857.4 $[M+H]^+$ (100 %), 879.5 $[M+Na]^+$ (40 %)
HRMS (ESI^+) found: 877.2537, $[M+H]^+$ calculated: 877.2546.

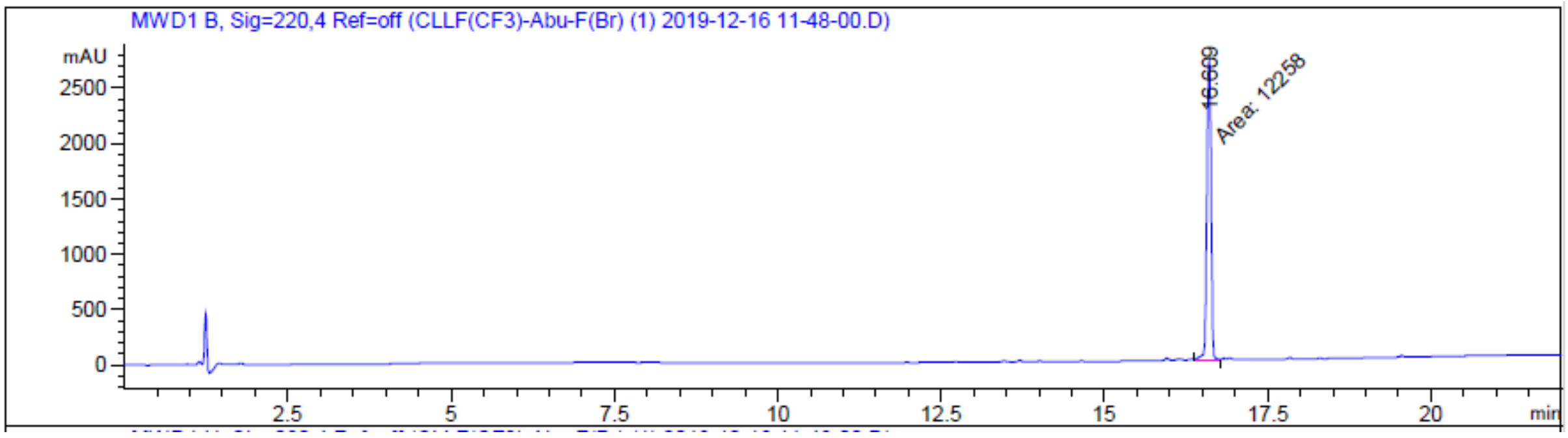

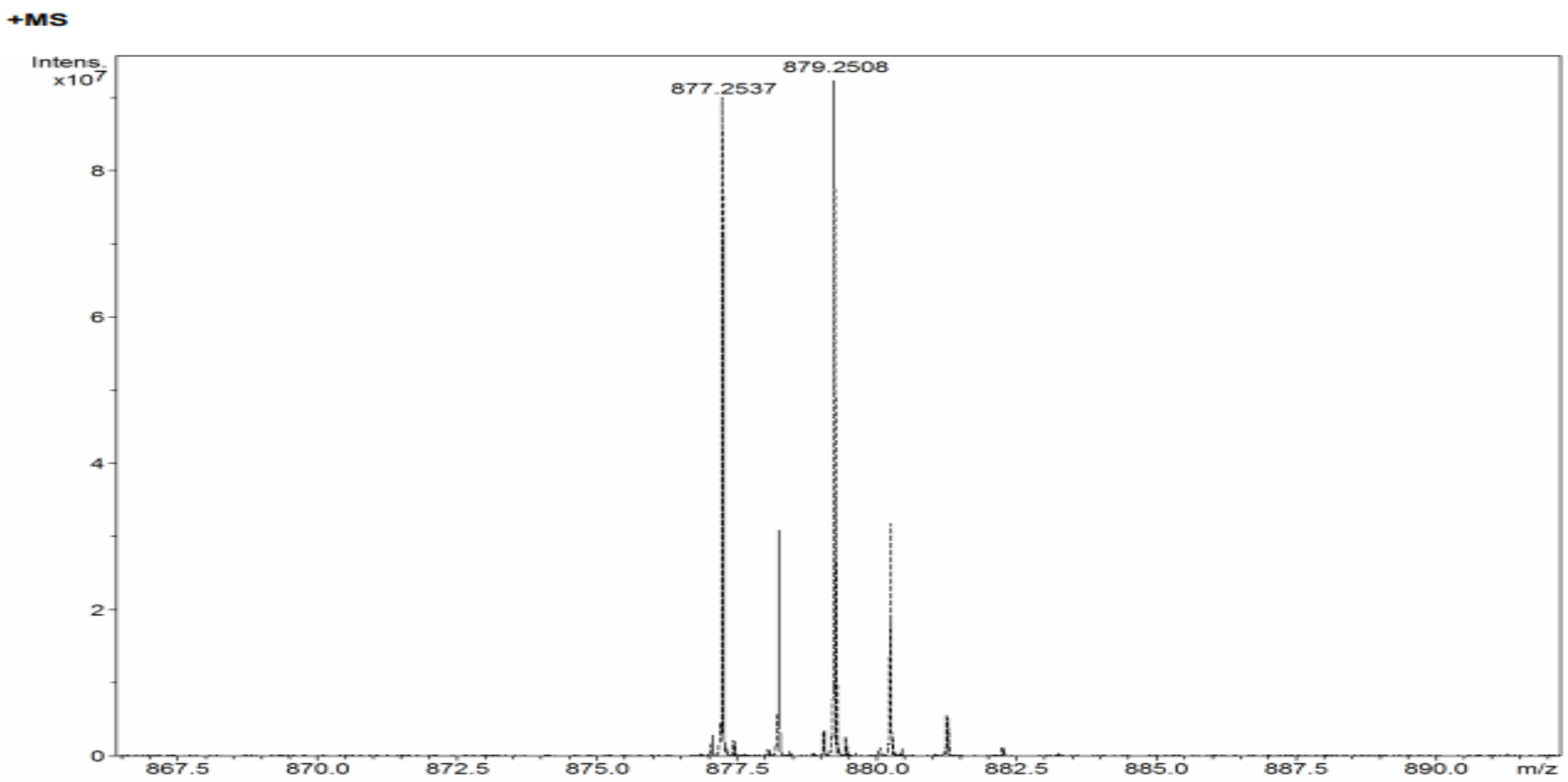

<u>*cyclo*-CLLF(*p*-CF_3)FAib(*p*-Br)F (**46**)</u>

cyclo-CLLF(*p*-CF_3)FAib(*p*-Br)F was obtained as a white solid (3.3 mg, 3.86 %).

Analytical HPLC: R_t = 16.835 min (89 % purity).
m/z (ESI^+): 857.4 $[M+H]^+$ (100 %).
HRMS (ESI^+) found: 877.2460, $[M+Na]^+$calculated: 877.2546.

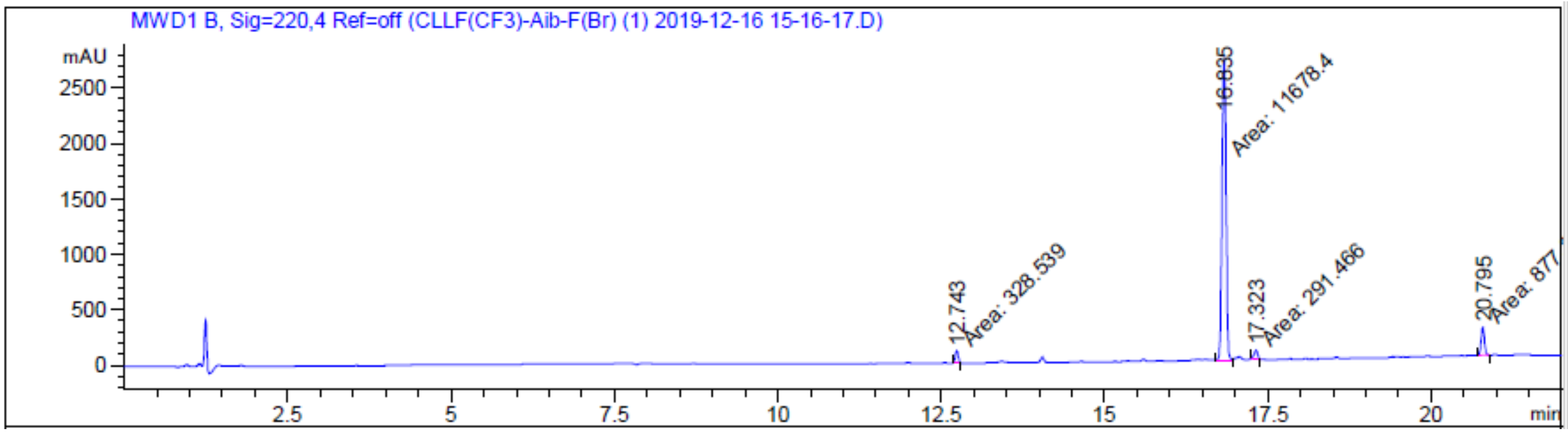

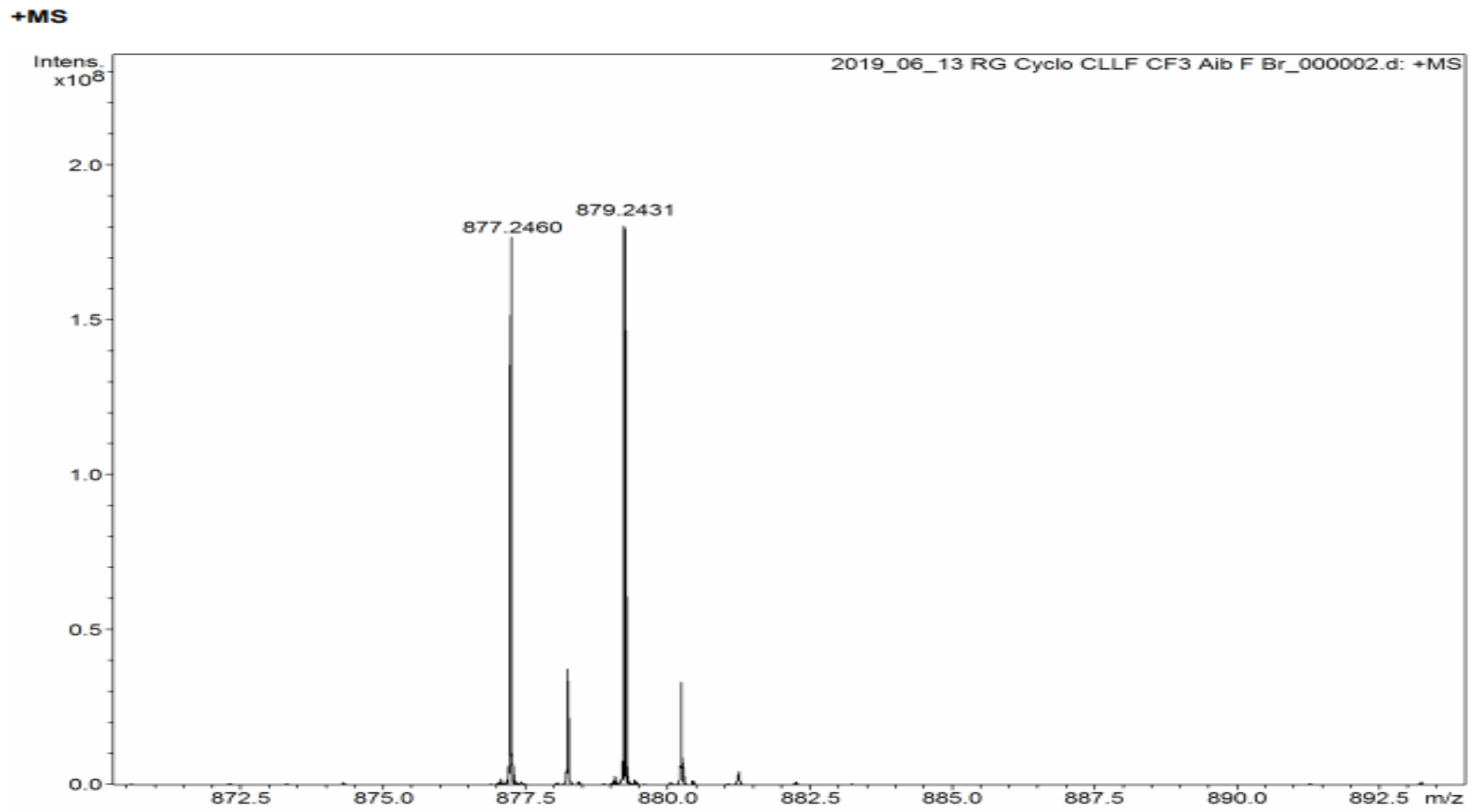

cyclo-CLLF(p-CF$_3$)FI(p-Br)F (**47**)

cyclo-CLLF(*p*-CF$_3$)FI(*p*-Br)F_was obtained as a white solid (14.2 mg, 8.0 %).

Analytical HPLC: R_t = 18.149 min (99 % purity).
m/z (ESI$^+$): 885.6 [M+H]$^+$ (100 %), 907.6 [M+Na]$^+$ (40 %).
HRMS (ESI$^+$) found: 883.3020, [M+H]$^+$ calculated: 883.3034.

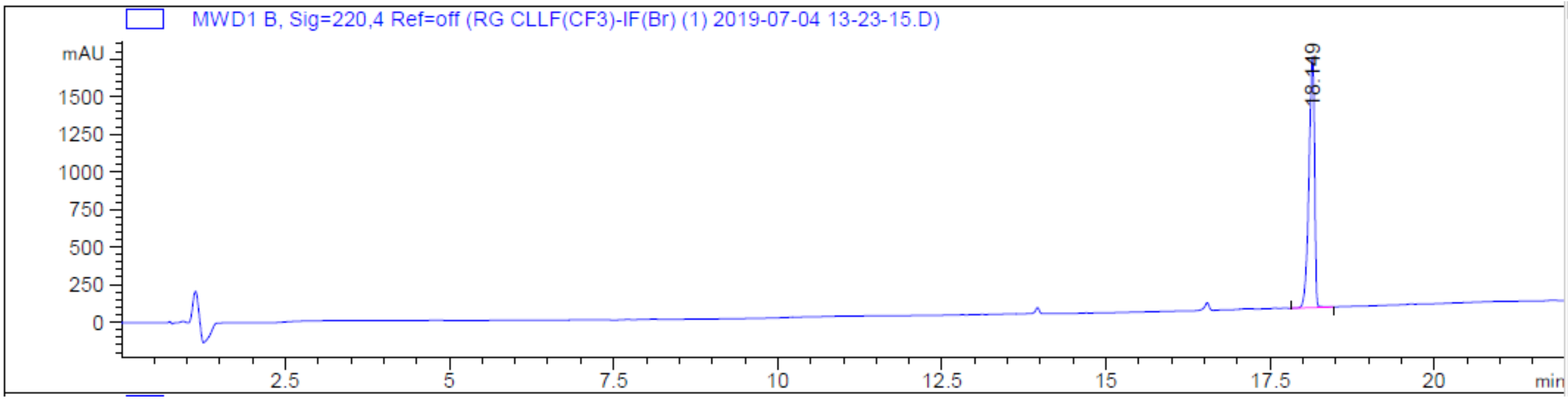

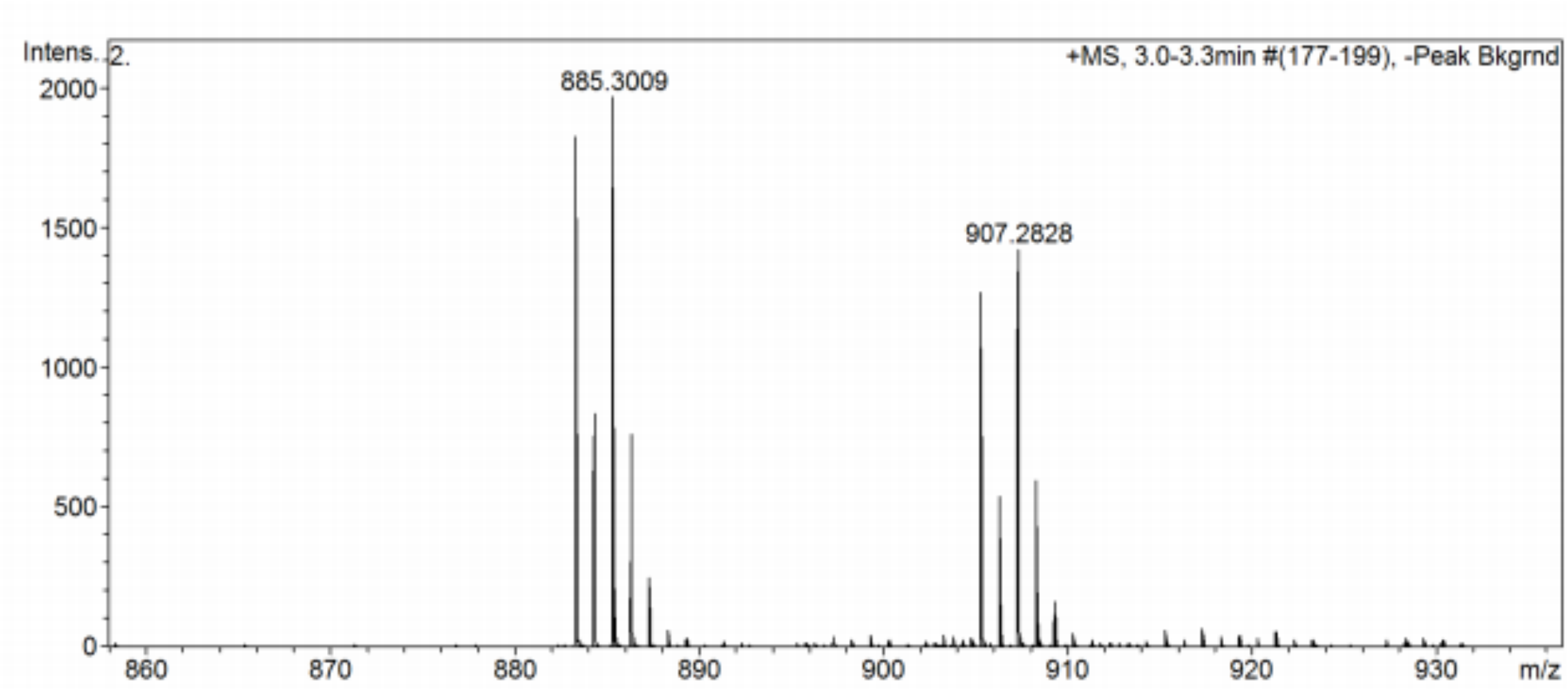

cyclo-CLLF(p-CF_3)FProp(p-Br)F (**48**)

cyclo-CLLF(*p*-CF_3)FProp(*p*-Br)F was obtained as a white solid (14.2 mg, 8.0 %).

Analytical HPLC: R_t = 16.822 min (98 % purity).

m/z (ESI$^+$): 873.5 [M+H]$^+$ (100 %), 893.4 [M+Na]$^+$ (99 %).

HRMS (ESI$^+$) found: 871.2660, [M+H]$^+$ calculated: 871.2670.

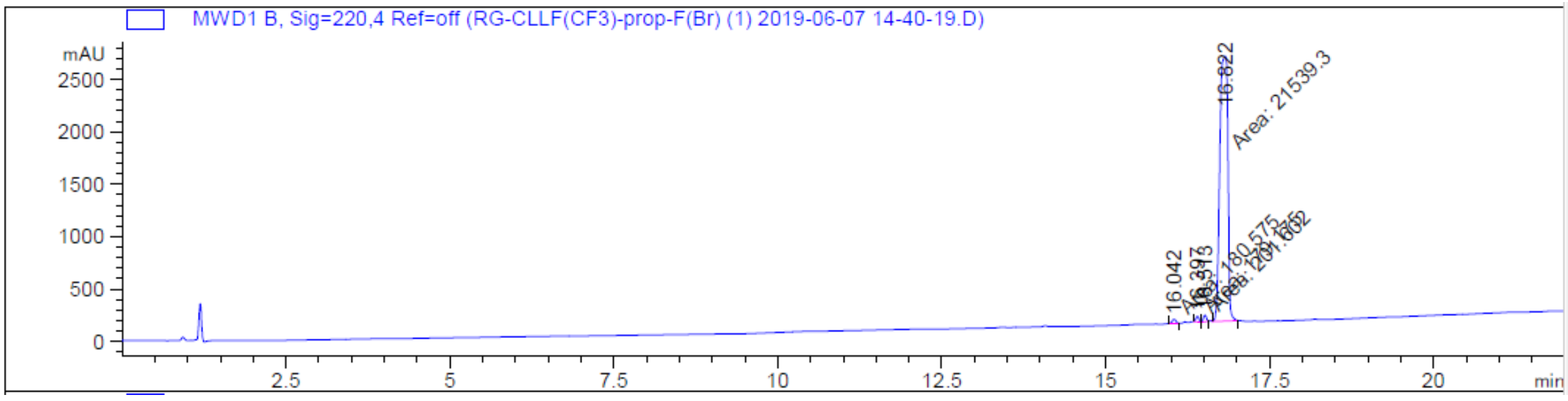

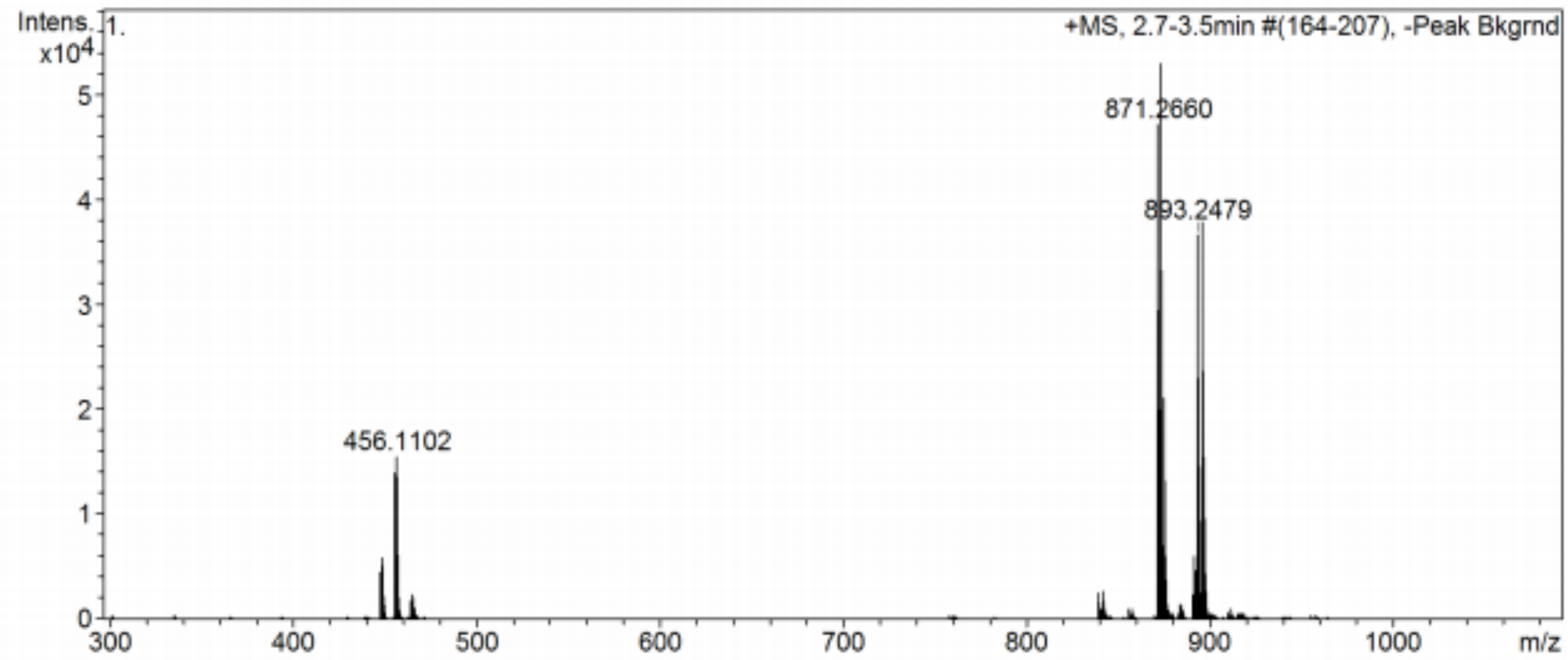

cyclo-CLhLF(*p*-CF_3)FI(*p*-Br)F (**49**)

cyclo-CLhLF(*p*-CF_3)FI(*p*-Br)F_was obtained as a white solid (16.2 mg, 18.1 %).

Analytical HPLC: R_t = 18.838 min (99 % purity).

m/z (ESI^+): 899.7 $[M+H]^+$ (100 %).

HRMS (ESI^+) found: 897.3166, $[M+H]^+$ calculated: 897.3190.

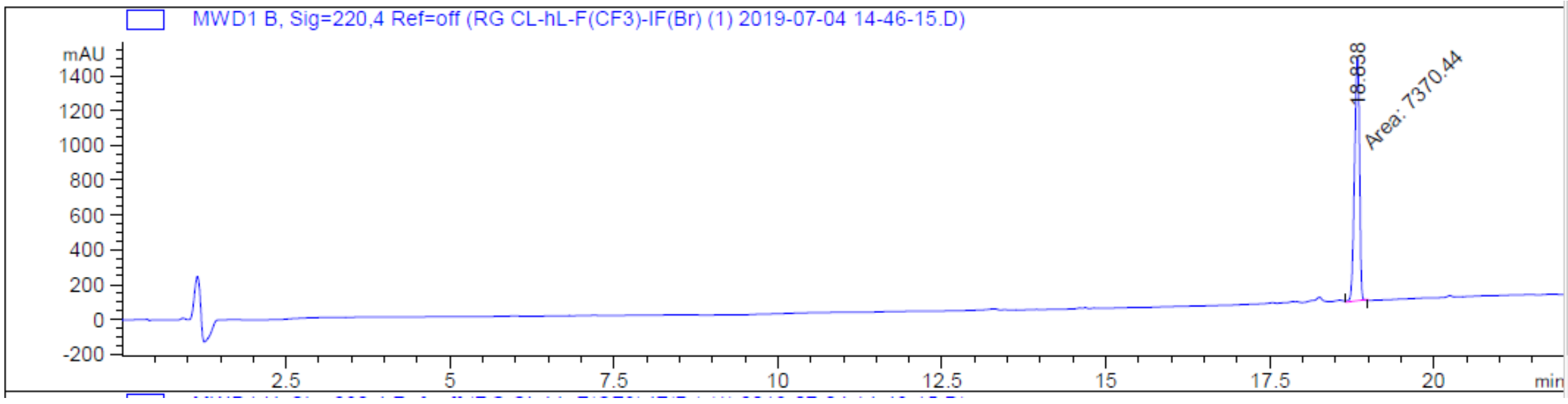

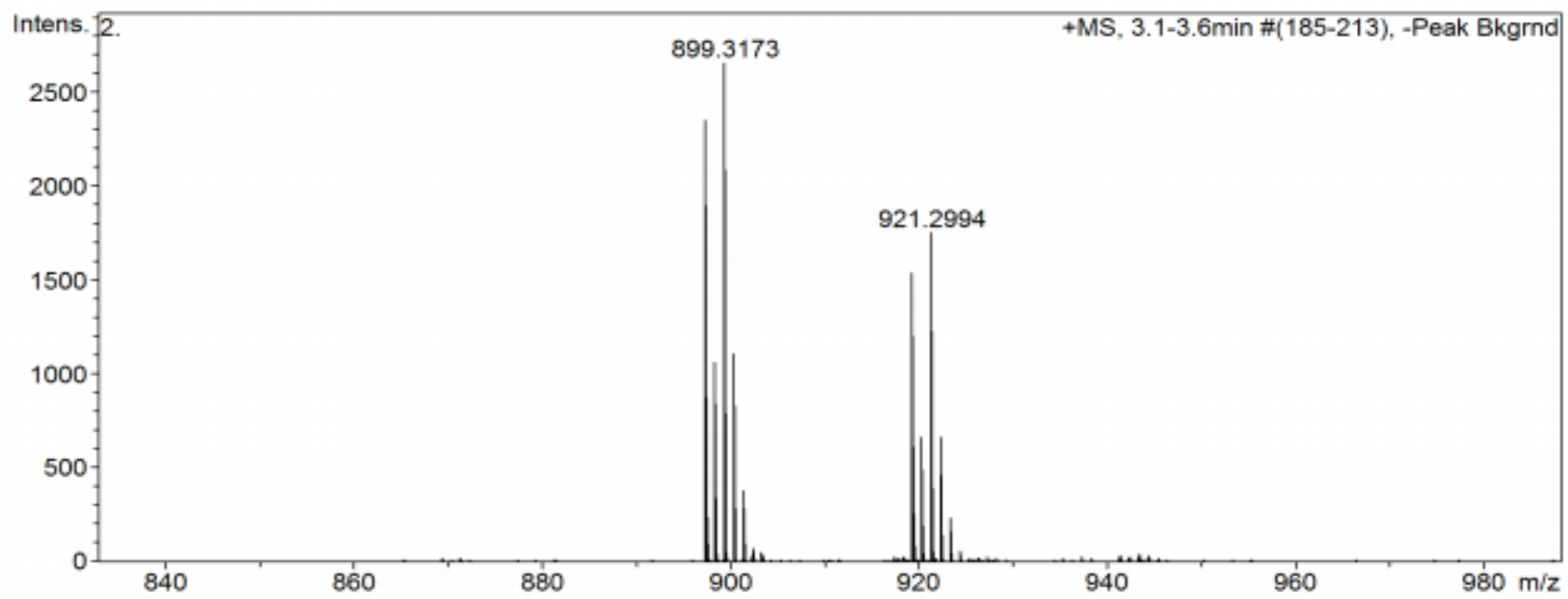

<u>*cyclo*-CLNleF(*p*-CF_3)FI(*p*-Br)F (**50**)</u>

cyclo-CLNleF(*p*-CF_3)FI(*p*-Br)F was obtained as a white solid (12.5 mg, 14.2 %).

Analytical HPLC: R_t = 18.310 min (99 % purity).
m/z (ESI$^+$): 885.6 $[M+H]^+$ (100 %).
HRMS (ESI$^+$) found: 883.3021, $[M+H]^+$ calculated: 883.3034.

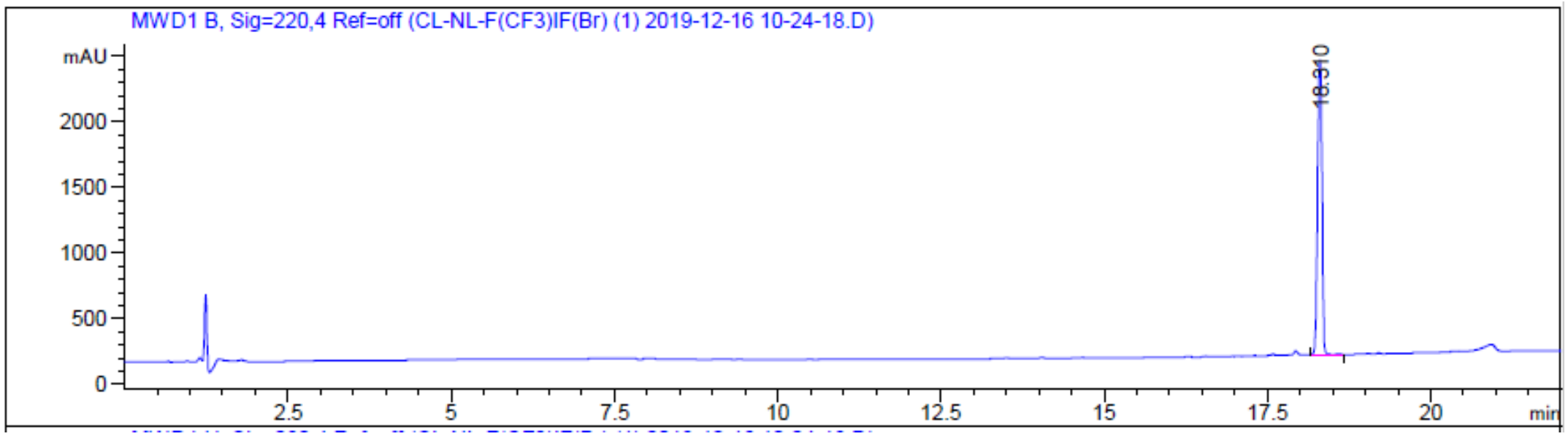

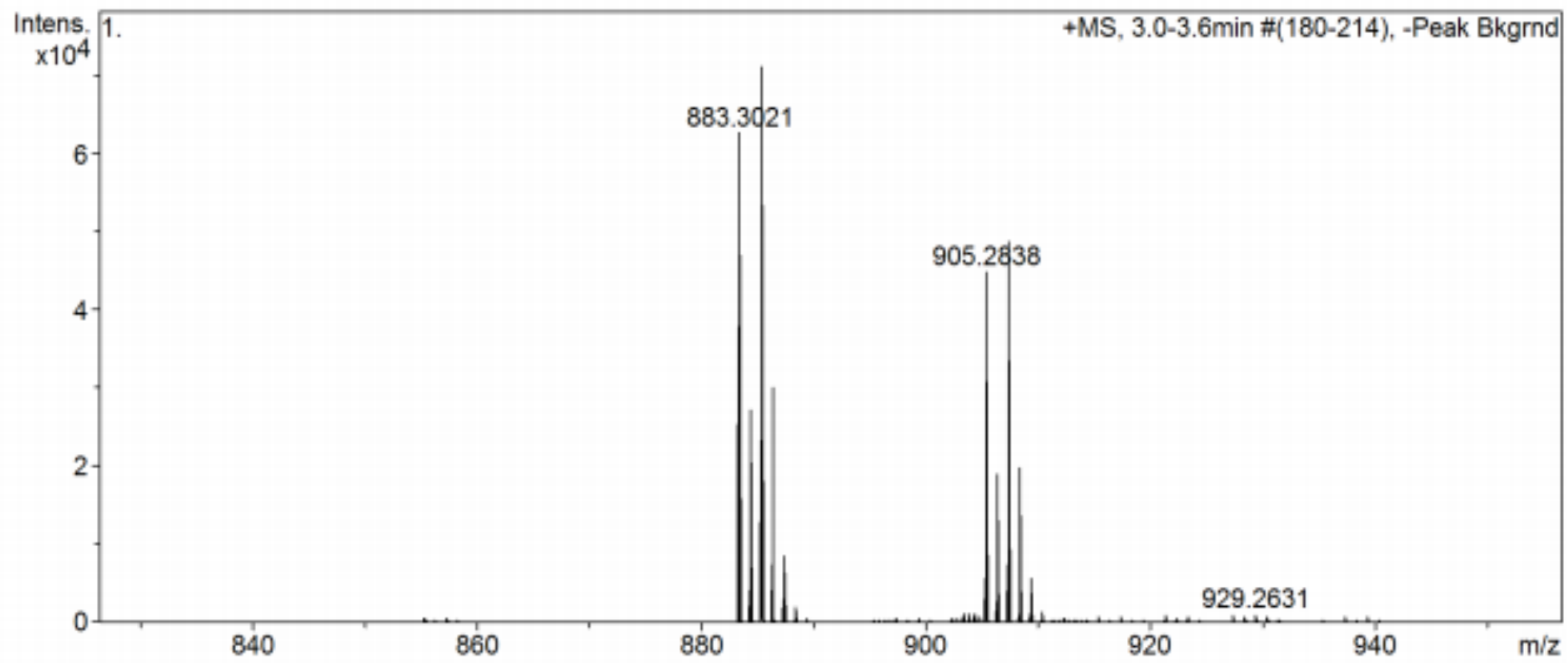

cyclo-CLVF(p-CF_3)FI(p-Br)F (**51**)

cyclo-CLVF(*p*-CF_3)FI(*p*-Br)F was obtained as a white solid (0.4 mg, 0.5 %).

Analytical HPLC: R_t = 18.116 min (97 % purity).
m/z (ESI$^+$): 871.6 $[M+H]^+$ (100%), 890.6 [M+Na] (45 %).
HRMS (ESI$^+$) found: 869.2850, $[M+H]^+$ calculated: 869.2877.

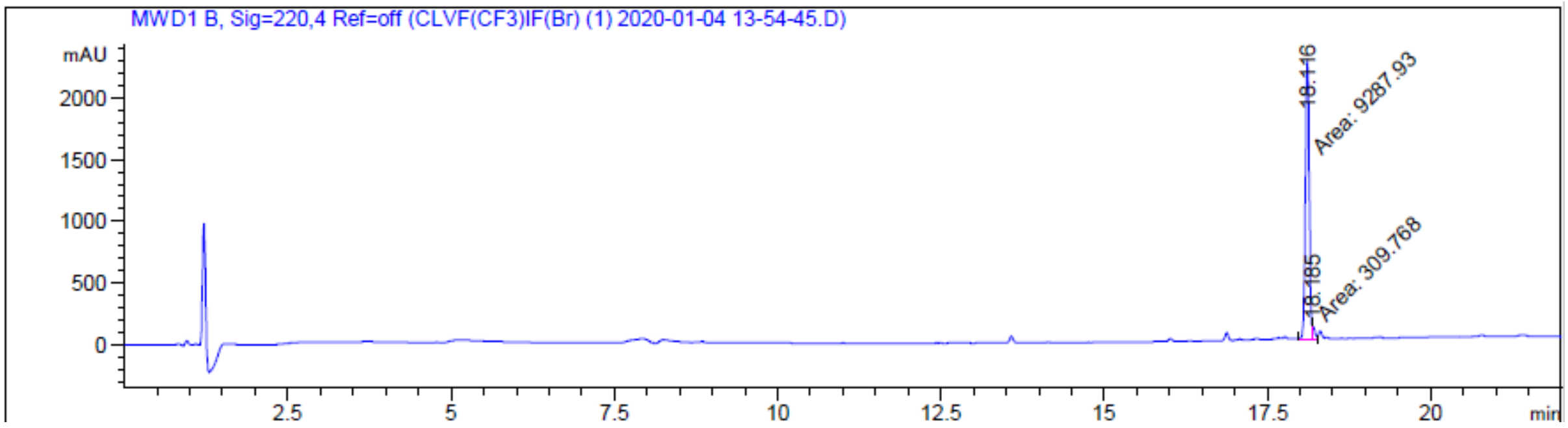

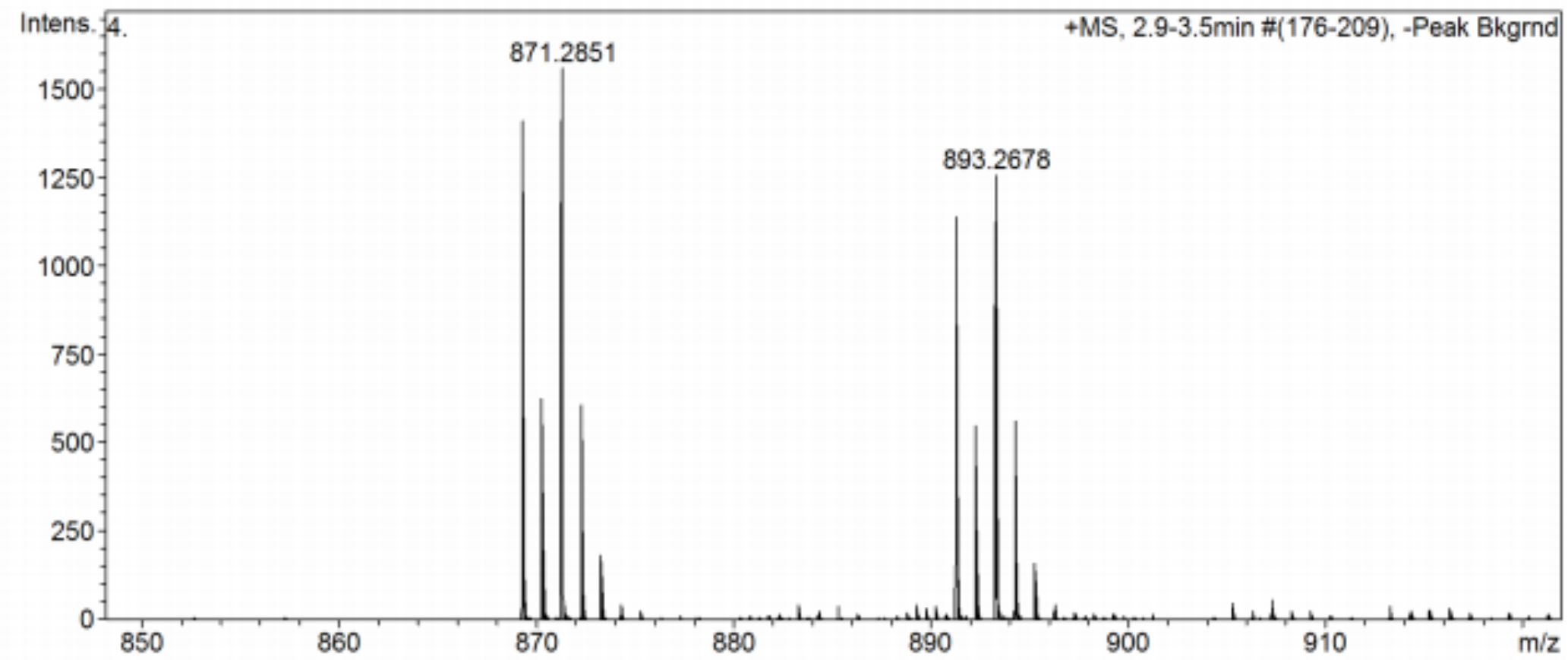

cyclo-CLNvaF(p-CF_3)FI(p-Br)F (**52**)

cyclo-CLNvaF(*p*-CF_3)FI(*p*-Br)F was obtained as a white solid (6.7 mg, 7.7 %).

Analytical HPLC: R_t = 17.729 min (99 % purity).
m/z (ESI^+): 871.6 $[M+H]^+$ (100 %).
HRMS (ESI^+) found: 869.2853, $[M+H]^+$ calculated: 869.2877.

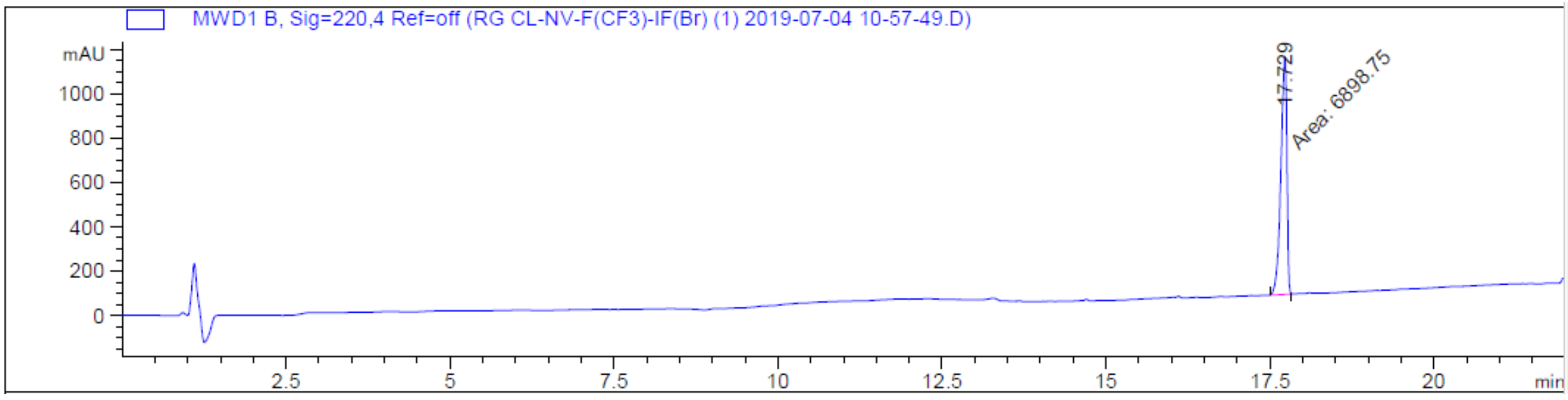

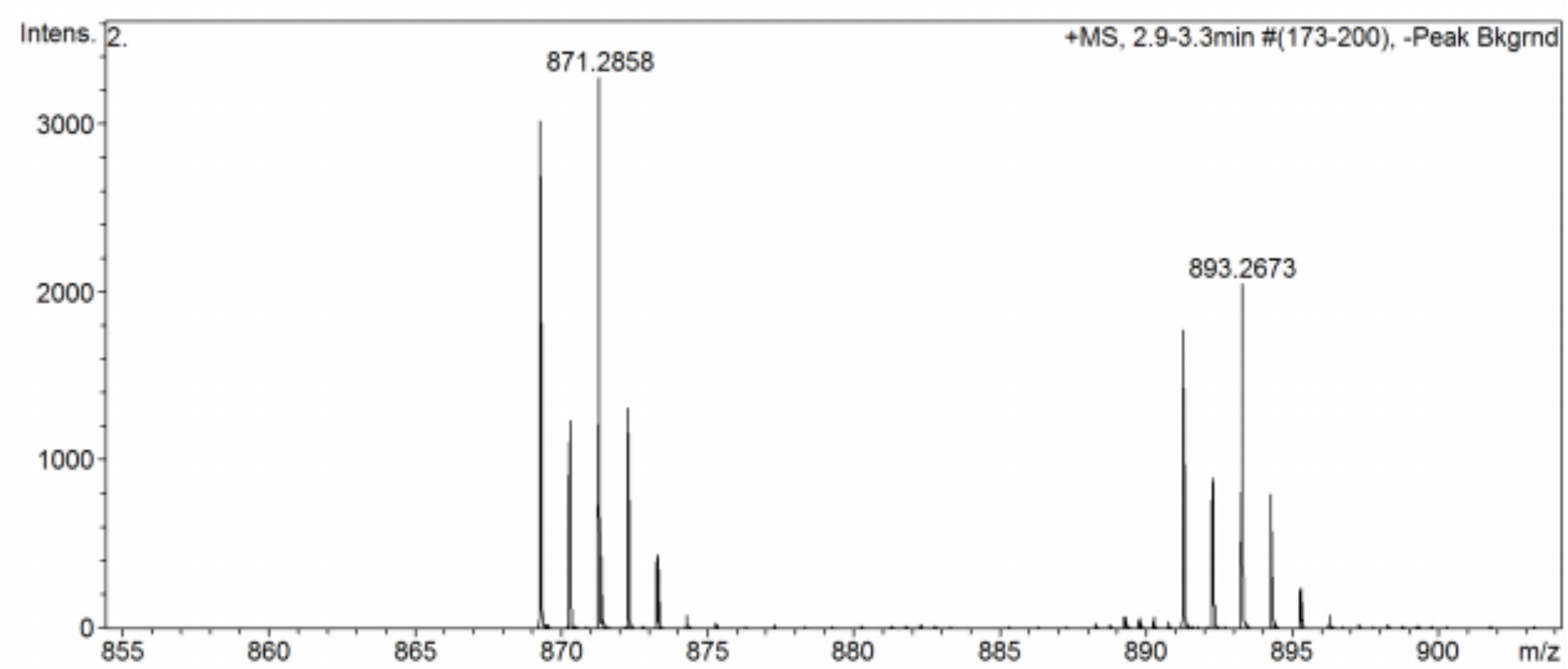

cyclo-CLAbuF(*p*-CF_3)FI(*p*-Br)F (**53**)

cyclo-CLAbuF(*p*-CF_3)FI(*p*-Br)F was obtained as a white solid (18.5 mg, 21.6 %).

Analytical HPLC: R_t = 16.316 min (91 % purity).

m/z (ESI^+): 857.6 $[M+H]^+$ (100 %).

HRMS (ESI^+) found: 855.2721, $[M+H]^+$ calculated: 855.2710.

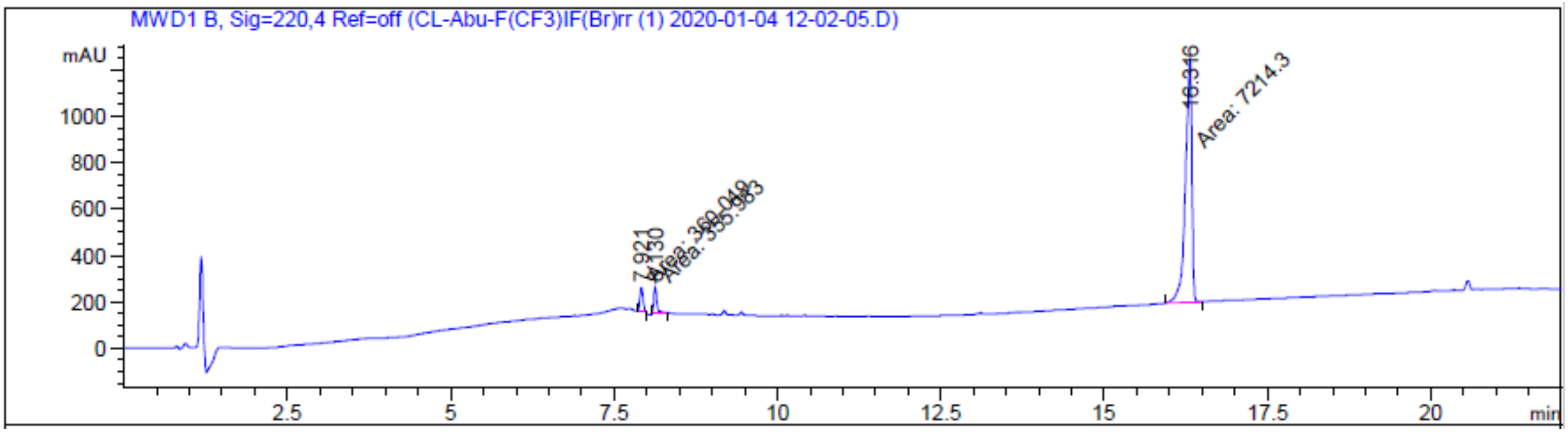

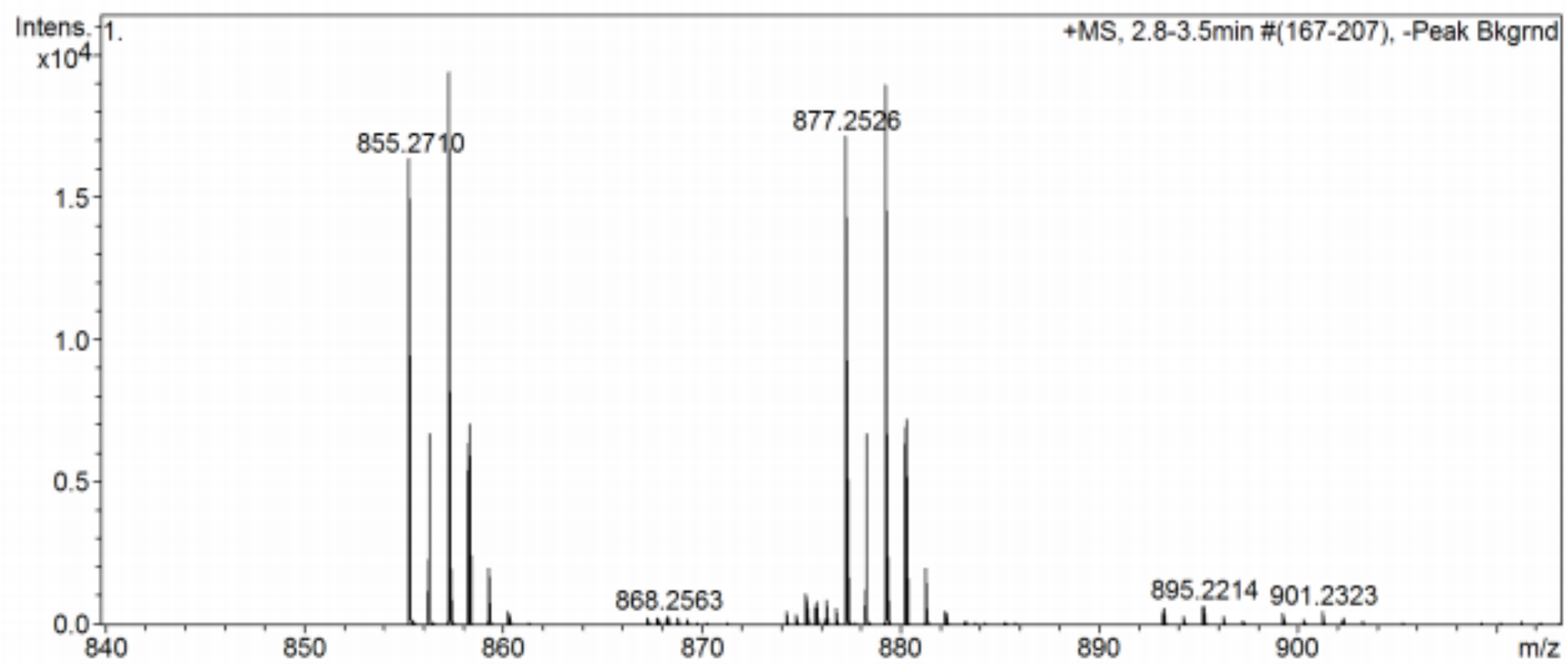

cyclo-CLAibF(p-CF_3)FI(p-Br)F (**54**)

cyclo-CLAibF(*p*-CF_3)FI(*p*-Br)F_was obtained as a white solid (24.7 mg, 28.9 %).

Analytical HPLC: R_t = 17.737 min (96 % purity).

m/z (ESI$^+$): 857.6 $[M+H]^+$ (100 %).

HRMS (ESI$^+$) found: 855.2721, $[M+H]^+$ calculated: 855.2721.

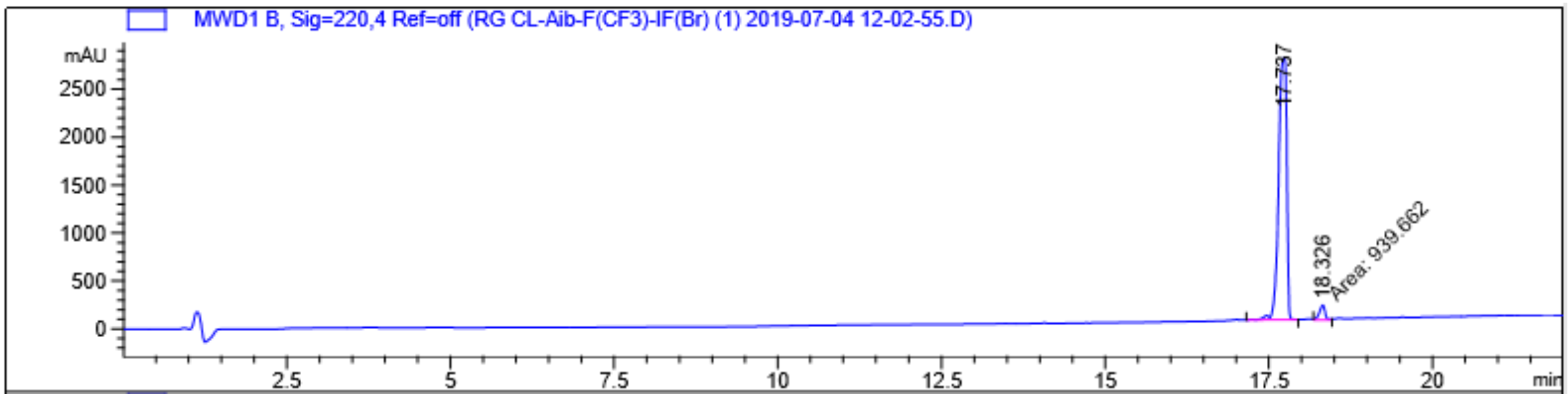

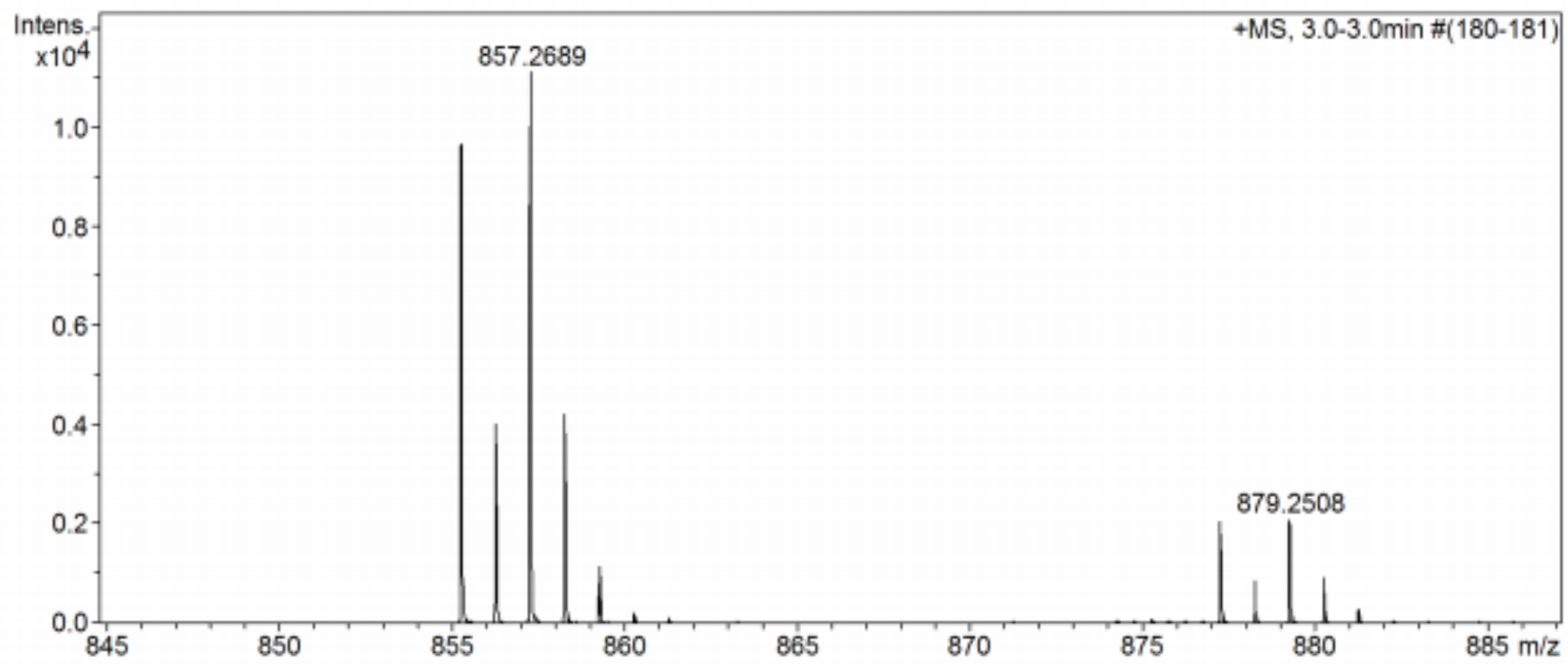

cyclo-CLIF(*p*-CF_3)FI(*p*-Br)F (**55**)
cyclo-CLIF(*p*-CF_3)FI(*p*-Br)F was obtained as a white solid (4.5 mg, 5.1 %).

Analytical HPLC: R_t = 18.566 min (96 % purity).
m/z (ESI$^+$): 885.6 $[M+H]^+$ (100 %).
HRMS (ESI$^+$) found: 883.3020, $[M+H]^+$ calculated: 883.3034.

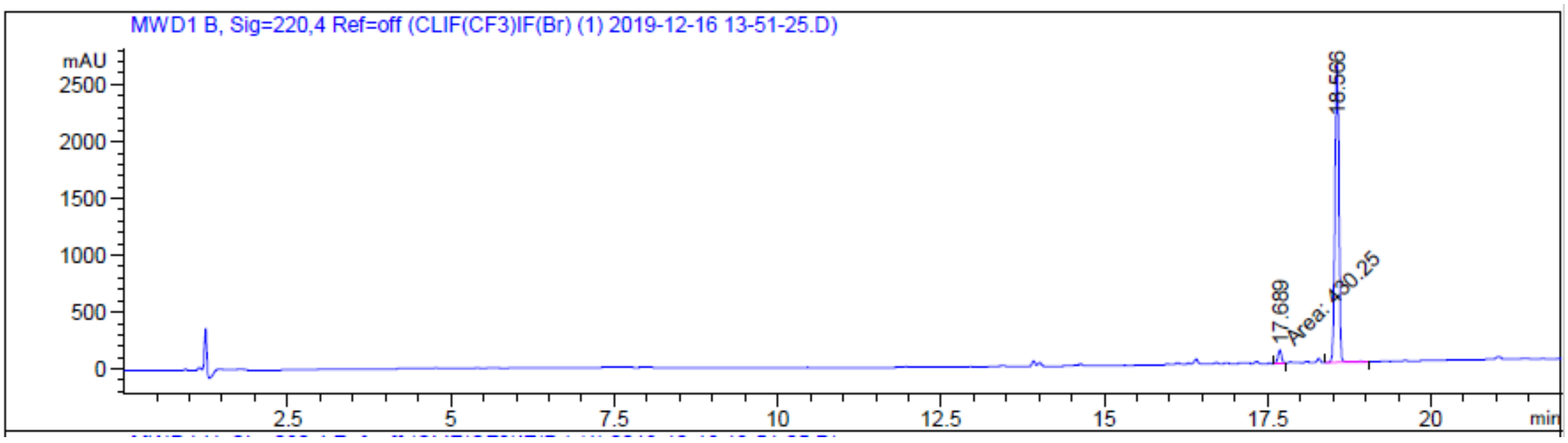

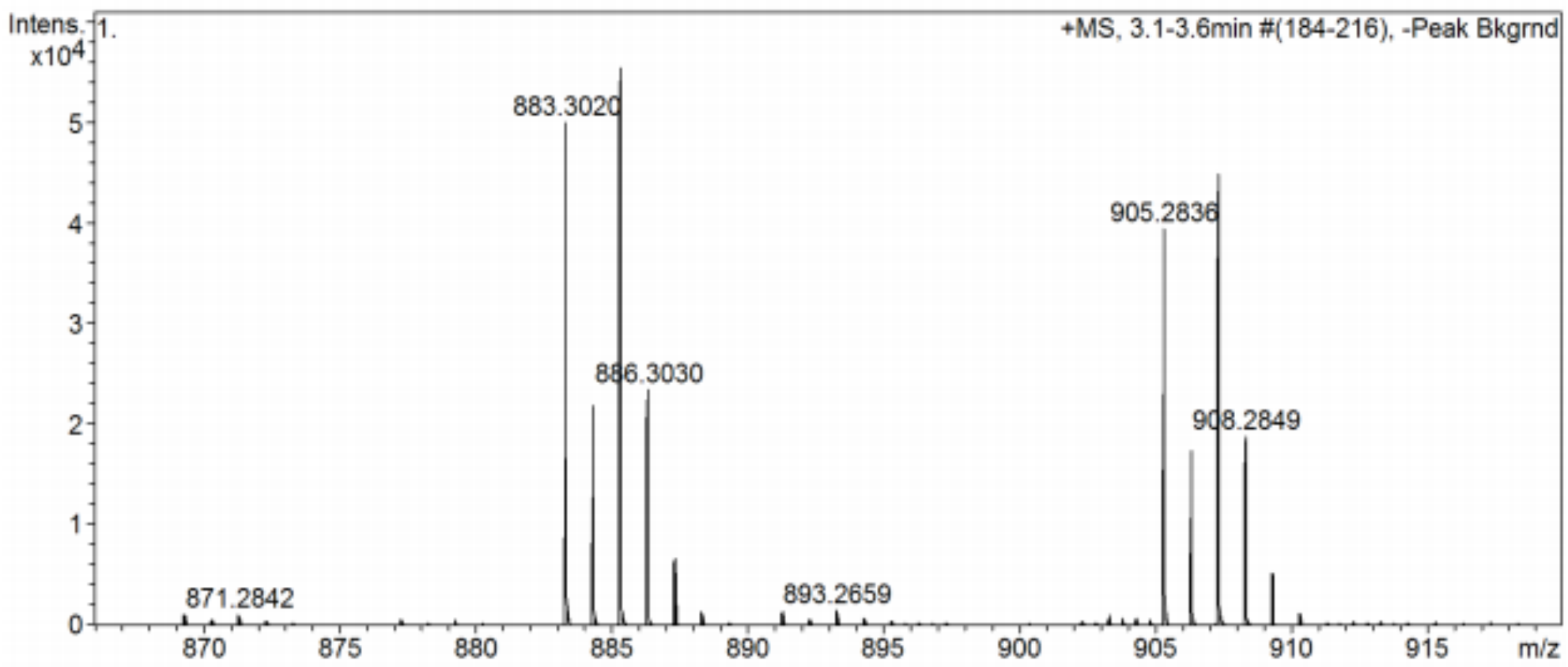

cyclo-CLPropF(p-CF_3)FI(p-Br)F (**56**)

cyclo-CLPropF(*p*-CF_3)FI(*p*-Br)F was obtained as white solid (6.1 mg, 7.0 %).

Analytical HPLC: R_t = 17.001 min (95 % purity).
m/z (ESI^+): 873.5 $[M+H]^+$ (100 %).
HRMS (ESI^+) found: 871.2670, $[M+H]^+$ calculated: 871.2670.

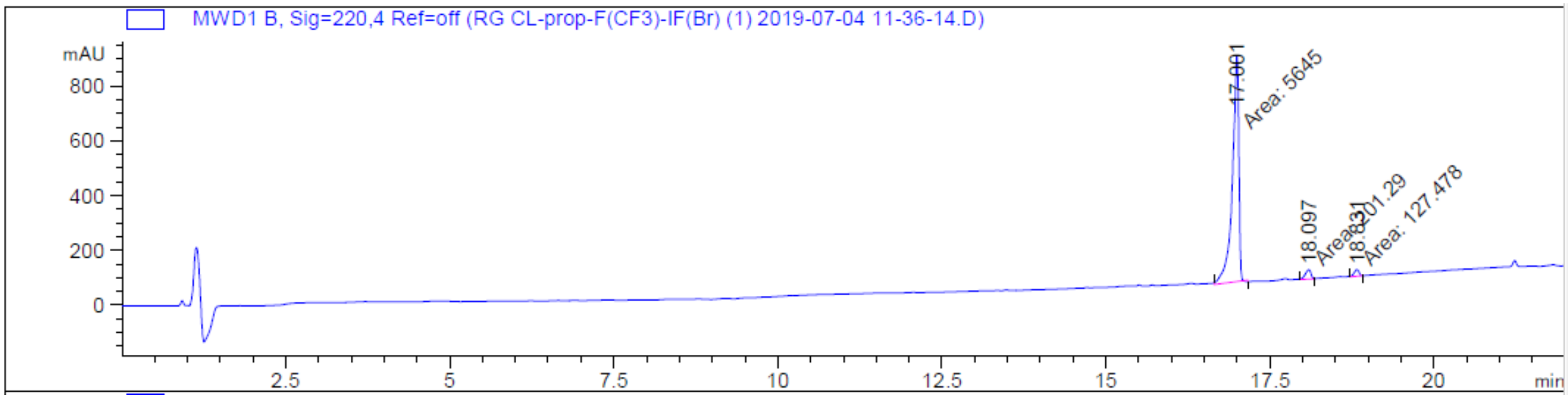

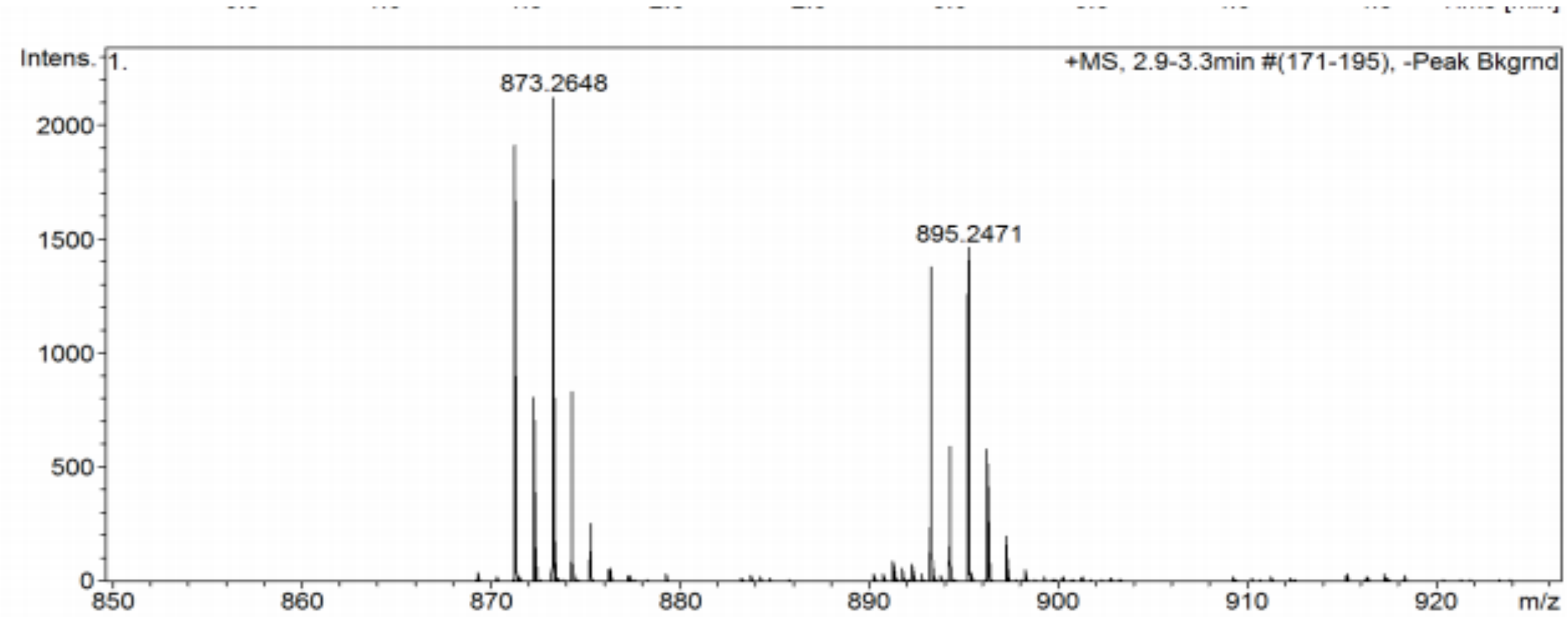

cyclo-CKNle(*p*-CF_3)FI(*p*-Br)F (**57**)

cyclo-CKNleF(*p*-CF_3)FI(*p*-Br)F_was obtained as white solid (51.2 mg, 28.5 %).

Analytical HPLC: R_t = 14.309 min (90 % purity).

m/z (ESI^+): 900.5 $[M+H]^+$ (100 %).

HRMS (ESI^+) found: 898.3061, $[M+H]^+$ calculated: 898.3143

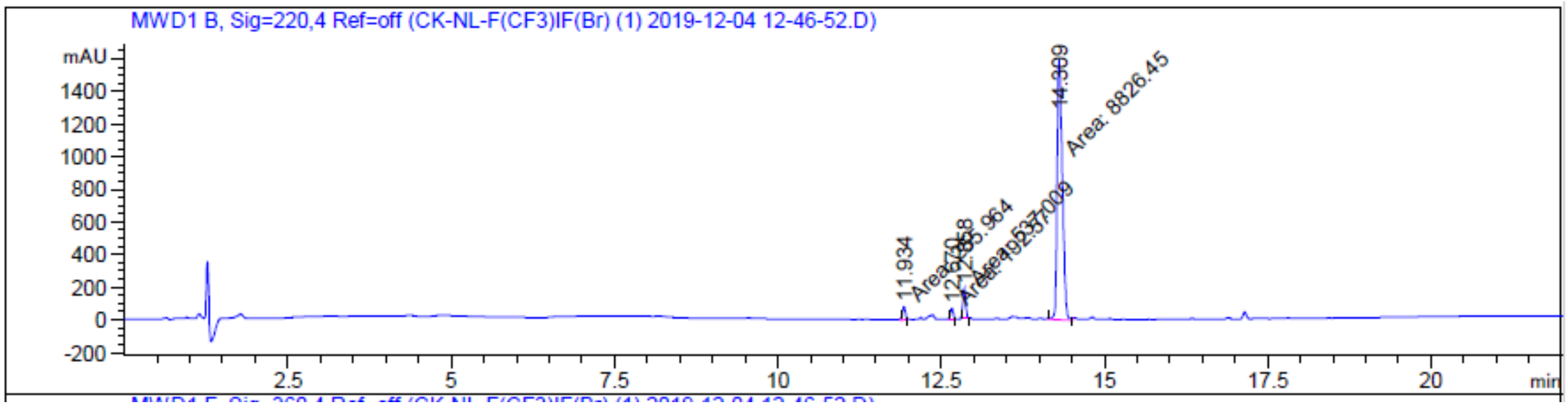

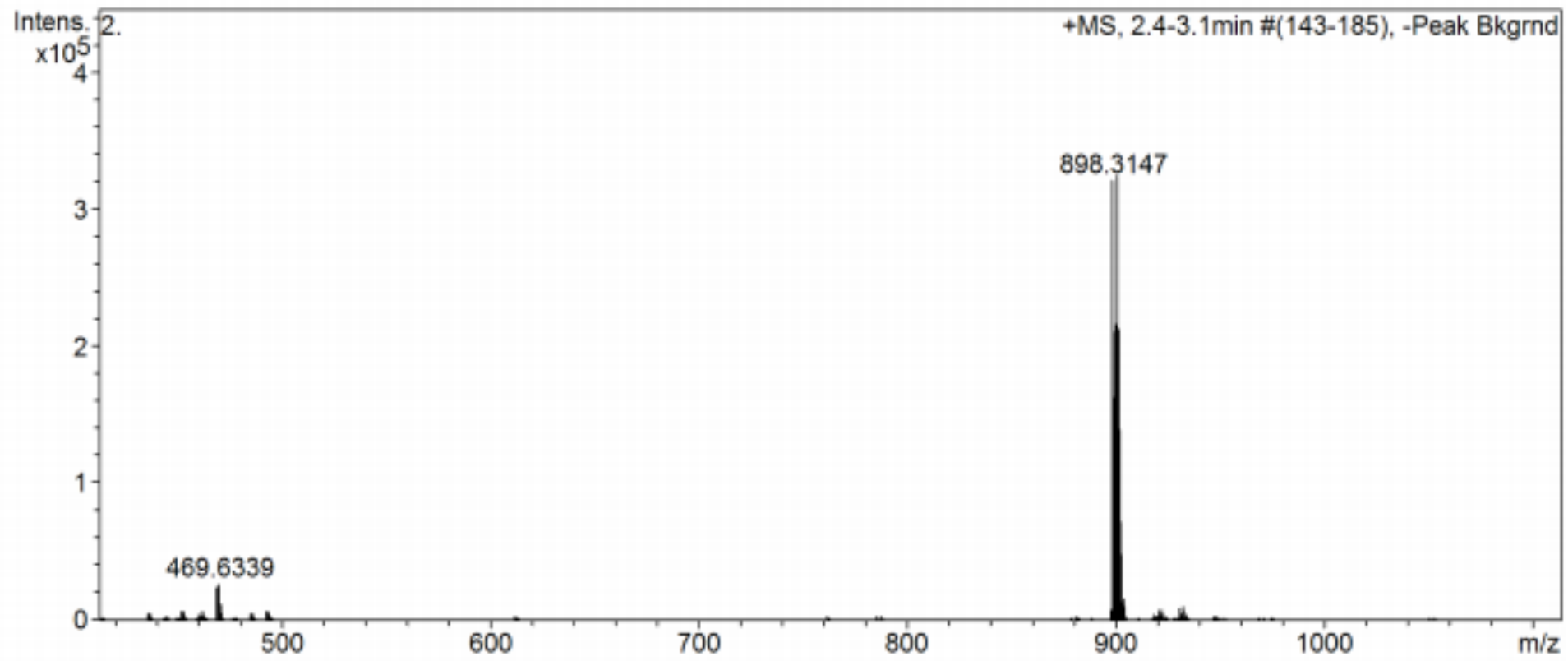

Synthesis of CTat

CGRKKRRQRRRPPQ linear peptide (CTat) was synthesised using 2-CTC Solid Phase peptide synthesis. The peptide was cleaved from the resin utilising a cleavage cocktail containing TFA/TIPS/H2O (95:2.5:2.5%) for 2 h at RT. The resin was removed via filtration and the peptide was concentrated *in vacuo*. The peptide was triturated with cold Et2O (-80 °C) which was removed followed by two additional washes of ice cold Et2O (-80 C). The resulting solid was further dried via rotary evaporation.

LR MS (ESI+): m/z $[M+H]^+$ cald for C73H136N36O17S, 1822.2, found; 911.6 $[M+2H]^{2+}$, 608.3 $[M+3H]^{3+}$, 456.4 $[M+4H]^{4+}$, 365.4 $[M+5H]^{5+}$.

Synthesis of 2-Mercaptopyridine (Spy) protected CTat

CGRKKRRQRRRPPQ (834 mg, 0.46 mmol, 1 eq) was dissolved in DMF (100 mL) to which was added Aldrithiol-2 (1 g, 4.6 mmol, 10 eq) and was left to stir at ambient temperature overnight. The mixture was concentrated *in vacuo*, and the product C(Spy)GRKKRRQRRRPPQ was precipitated from the reaction mixture by drop-wise addition of the oily mixture into ice cold Et2O (-80 °C). The Et2O was removed, and the precipitate washed two additional times followed by drying via rotary evaporation. The crude was then purified using a Biotage chromatography system. Fractions were analysed via LC/MS, desired fractions were then pooled, concentrated *in vacuo*, and lyophilised to yield C(Spy)GRKKRRQRRRPPQ as a white solid (400 mg, 0.21 mmol, 45%).

LR MS (ESI+): m/z $[M+H]^+$ cald for C78H139N37O17S2, 1930.1, found; 966.2$[M+2H]^{2+}$, 644.4 $[M+3H]^{3+}$, 483.7 $[M+4H]^{4+}$, 387.2 $[M+5H]^{5+}$.

cyclo-C(Tat)LL(*p*-CF_3)FI(*p*-Br)F (**Tat-47**)

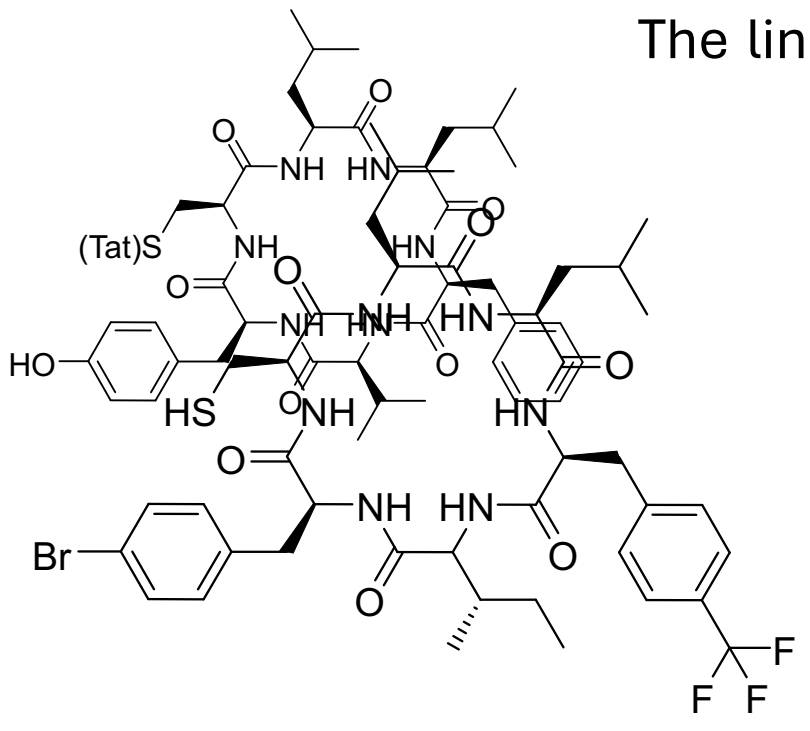

The linear peptide LCF(Br)IF(CF3)L was synthesised using Wang resin SPPS at a 0.2 mmol scale. The linear peptide was then cyclised and purified via reverse-phase flash chromatography to yield the S^{t}Bu protected cyclic peptide C(StBu)LLF(CF3)IF(Br). The S^{t}Bu was then removed and purified via reversed-phase HPLC. Fractions were analysed via LC/MS, desired fractions were then pooled, concentrated *in vacuo,* and lyophilised to yield the desired product as an off-white powder (20 mg, 0.022 mmol, 11% overall). *Cyclo*-CLLF(CF3)IF(Br) (5 mg, 0.0079 mmol, 1 eq) was added to a solution of C(Spy)GRKKRRQRRRPPQ (30 mg, 0.016 mmol, 2 eq) dissolved in DMF (2 mL) and stirred in an inert atmosphere overnight at ambient temperature. The mixture was then purified by reverse-phase HPLC. Fractions analysed via LC/MS, desired fractions were then pooled, concentrated *in vacuo*, and lyophilised to yield *Cyclo*-C(Tat)LLF(CF3)IF(Br) as a white solid (8 mg, 0.0058 mmol, 74%).

LR MS (ESI$^+$): m/z $[M+H]^+$ cald for $C_{113}H_{188}BrF_3N_{42}O_{23}S_2$, 2701.4, found; 542.1 $[M+5H]^{5+}$, 451.8 $[M+6H]^{6+}$.

HRMS (ESI$^+$): shows expected model isotope pattern.

Analytical HPLC: Rt: (220 nm) 12.654 min (98.4%)

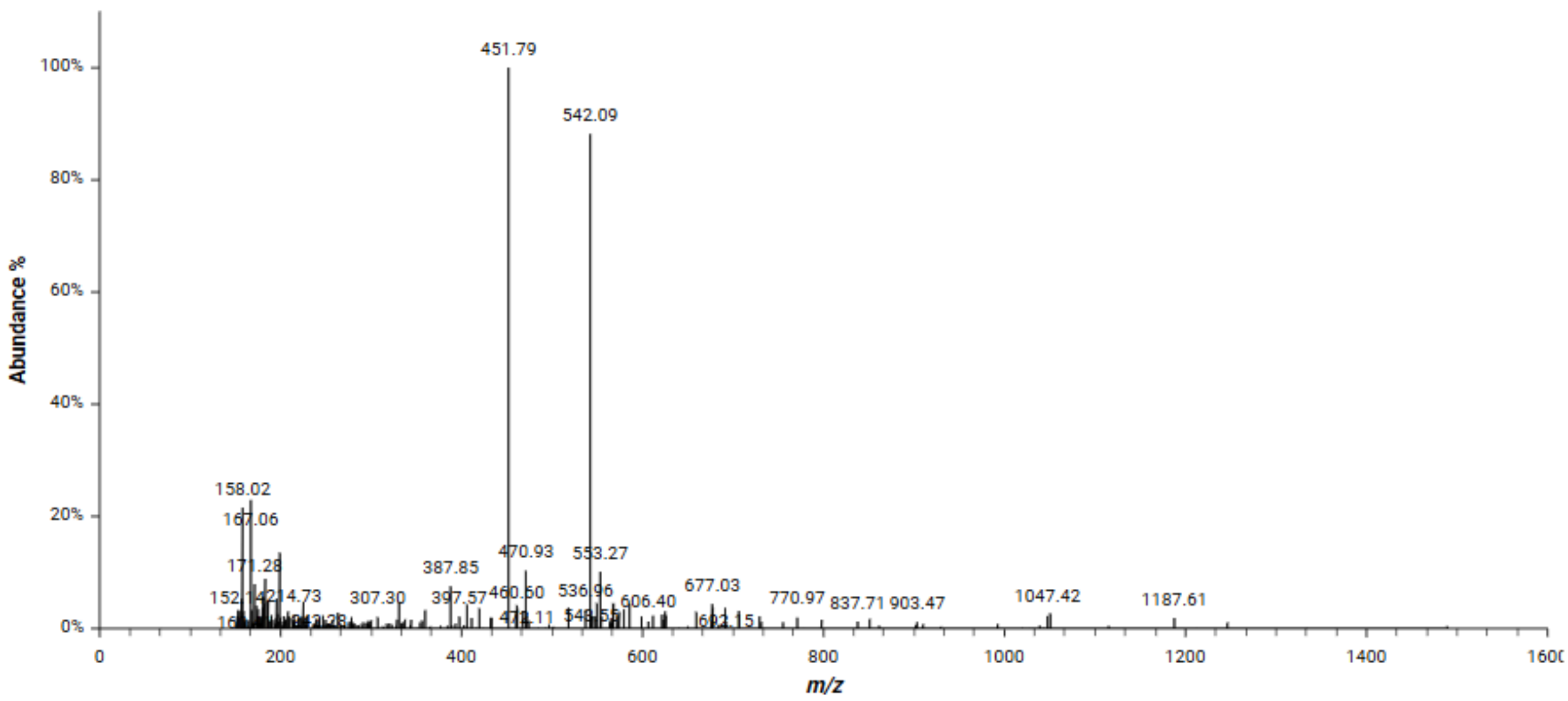

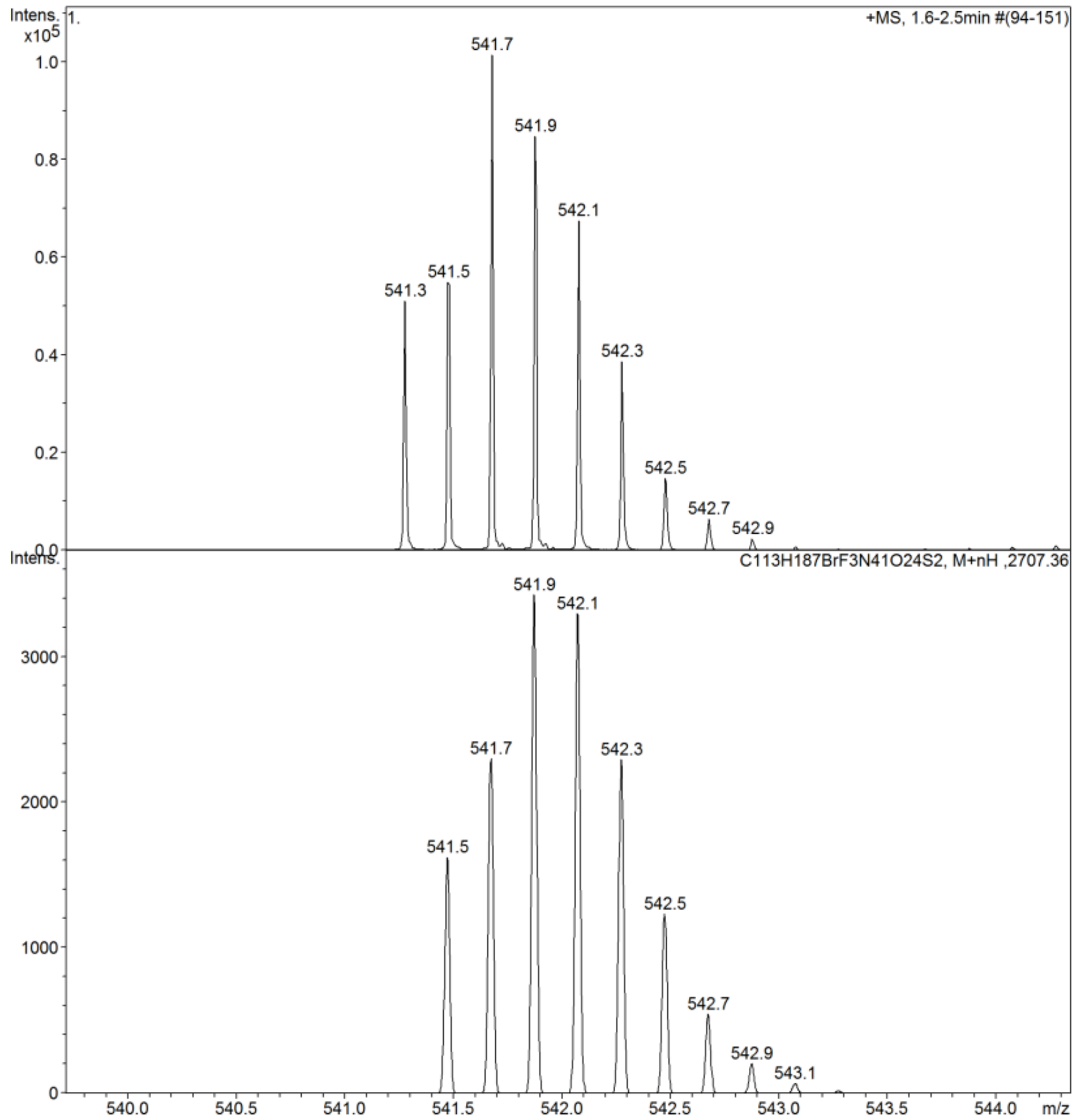
Intens.
x10^5
+MS, 1.6-2.5min #(94-151)
541.3
541.5
541.7
541.9
542.1
542.3
542.5
542.7
542.9
C113H187BrF3N41O24S2, M+nH ,2707.36
543.1
m/z

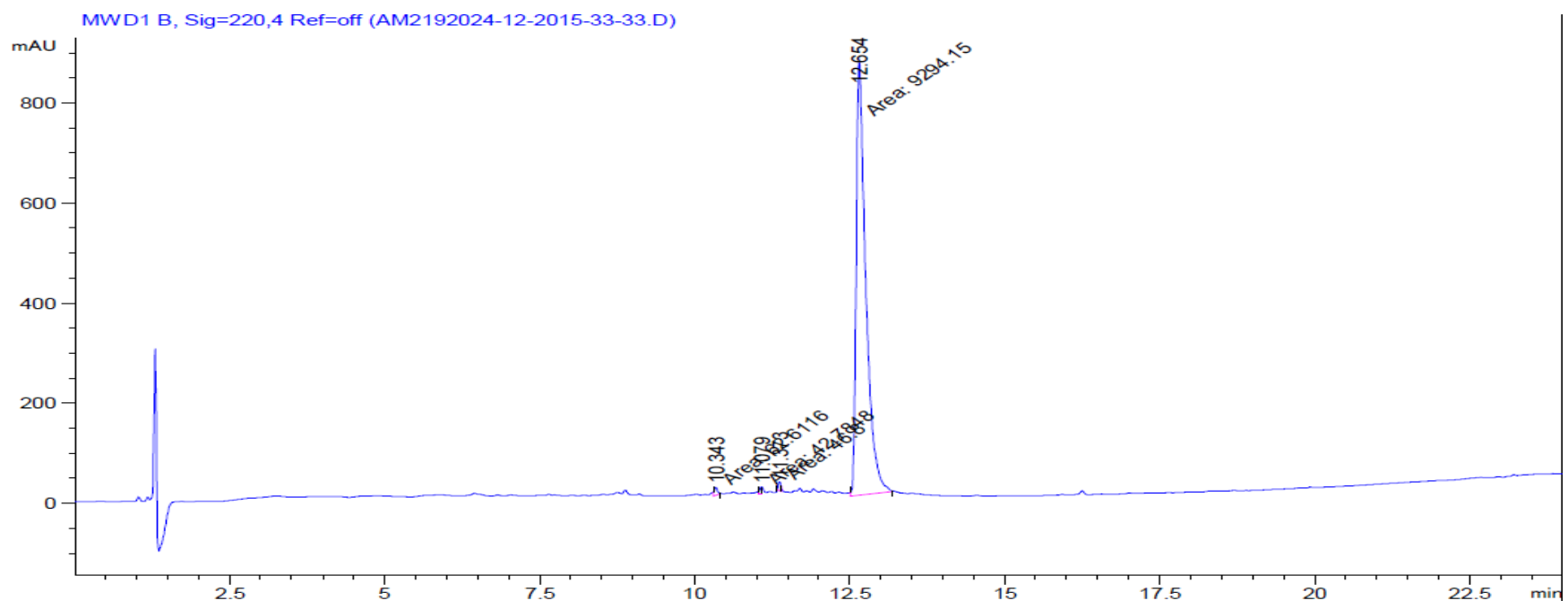
MWD1 B, Sig=220,4 Ref=off (AM2192024-12-2015-33-33.D)
mAU
12.654
Area: 9294.15
10.343
min

VH032

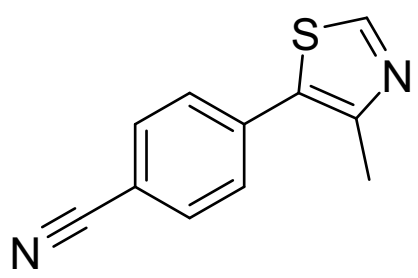

The following procedures used to synthesise VH032 were adapted from Buckley et al (Buckley DL, *et al.* Targeting the von Hippel-Lindau E3 ubiquitin ligase using small molecules to disrupt the VHL/HIF-1α interaction. *J Am Chem Soc*. 2012;134(10):4465-4468), apart from 4-(4-methylthiazol-5-yl)benzonitrile.

A solution of dry DMAc (6 mL) and 4-methylthiazole (2 mL, 22.12 mmol, 2 eq) to an oven dried 3-neck round bottom flask with a stirrer bar. The solution was purged with argon for 30 minutes whilst stirring. 4-bromobenzonitrile (2 g, 11.06 mmol, 1 eq), potassium acetate (2.17g, 22.12 mmol, 2 eq) and palladium acetate (2.4 mg, 0.0182% mmol) were subsequently added, and the mixture was heated to 150 °C and stirred for 16 h under argon. The mixture was cooled down to room temperature and was diluted with EtOAc (20 mL) which was washed with water (3 x 20mL). The combined organic extracts were then dried ($MgSO_4$), filtered and concentrated *in vacuo*. Purification *via* flash chromatography eluted the product at 1:1 EtOAc:PE to afford 4-(4-methylthiazol-5-yl)benzonitrile as a yellow solid (1.82 g, 9.1mmol, 82%).

^{1}H NMR (400 MHz, CHLOROFORM-*d*) δ ppm 8.90 (s, 1 H) 7.74 (d, *J*=8.44 Hz, 2 H) 7.57 (d, *J*=8.44 Hz, 2 H) 2.59 (s, 3 H).

LR MS (ESI$^+$) m/z [M+H]$^+$ cald for $C_{11}H_8N_2S_1$, 201.1, found; 201.1.

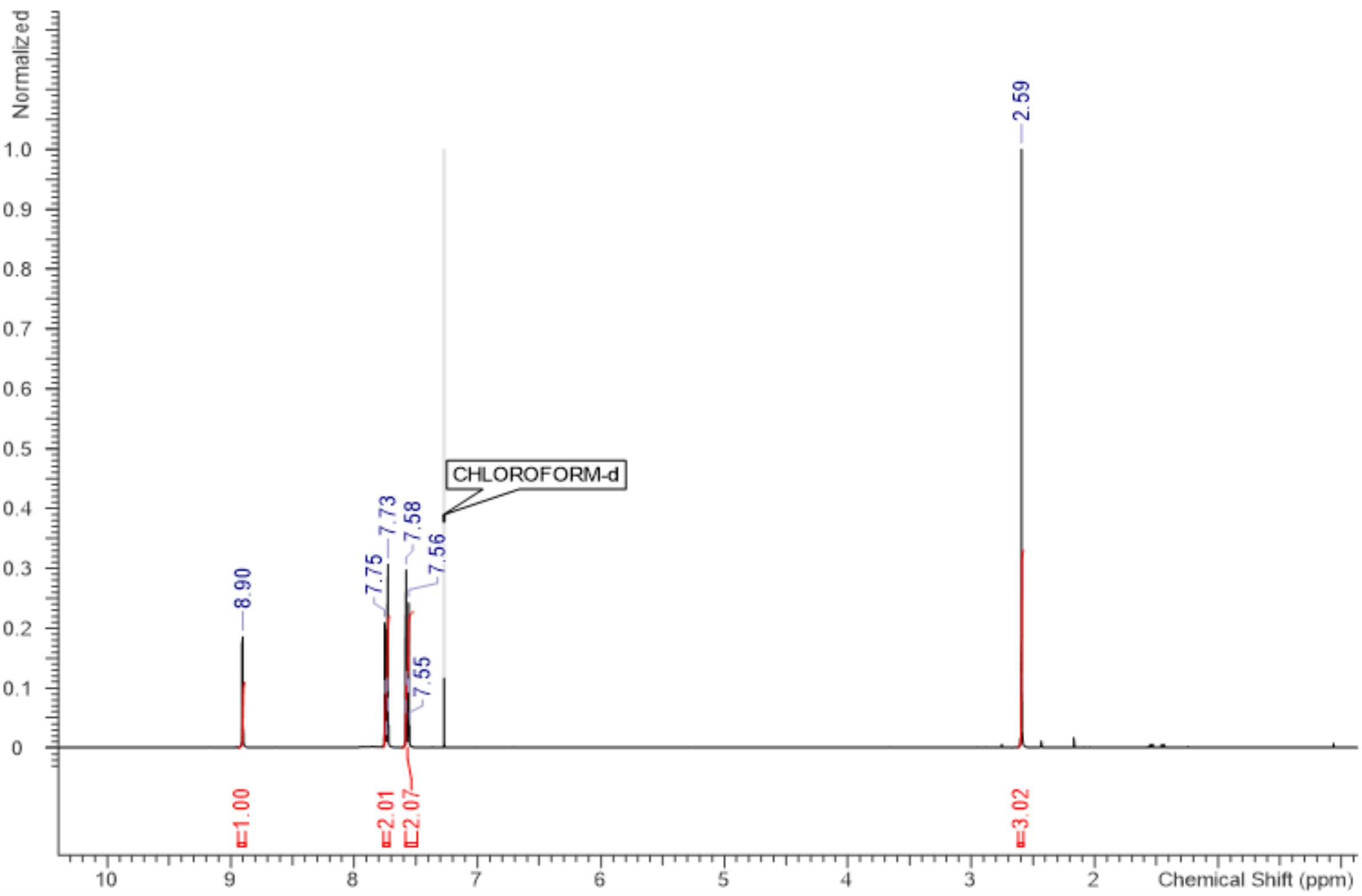

(4-(4-methylthiazol-5-yl)phenyl)methanamine

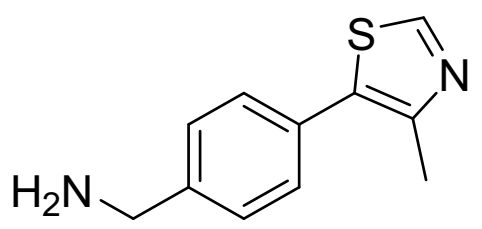

4-(4-methylthiazol-5-yl)benzonitrile (1.82 g, 9 mmol) was dissolved in 5 mL of THF with a stirrer bar and the solution was cooled to 0 °C. Whilst stirring 1.0 M BH3 in THF (9 mL, 9 mmol, 3 eq) was added to the solution drop wise and allowed to stir under argon overnight at r.t.

A fieser workup methodology was utilised where the solution was diluted with 10 mL of diethyl ether and cooled to 0ºC. Whilst stirring, 10 mL of water was added slowly to the solution (Caution: Flammable gas evolved). Once bubbling subsided, 9 mL of 1 M NaOH was added. The organic phase was washed with water (3 x 10 mL) and the combined organic extracts dried ($MgSO_4$), filtered and concentrated *in vacuo*. Purification via flash chromatography eluted the product at 10% MeOH/DCM with 1 M NH_3 in methanol to afford (4-(4-methylthiazol-5-yl)phenyl)methanamine as a yellow oil (584 mg, 2.9 mmol, 31% yield).

^{1}H NMR (400 MHz, METHANOL-d_4) δ ppm 8.84 (s, 1 H) 7.57 (s, 4 H) 3.80 (s, 2 H) 2.43 (s, 3 H).

LR MS (ESI$^+$): m/z $[M+H]^+$ cald for $C_{11}H_{12}N_2S_1$, 205.1, found; 205.3

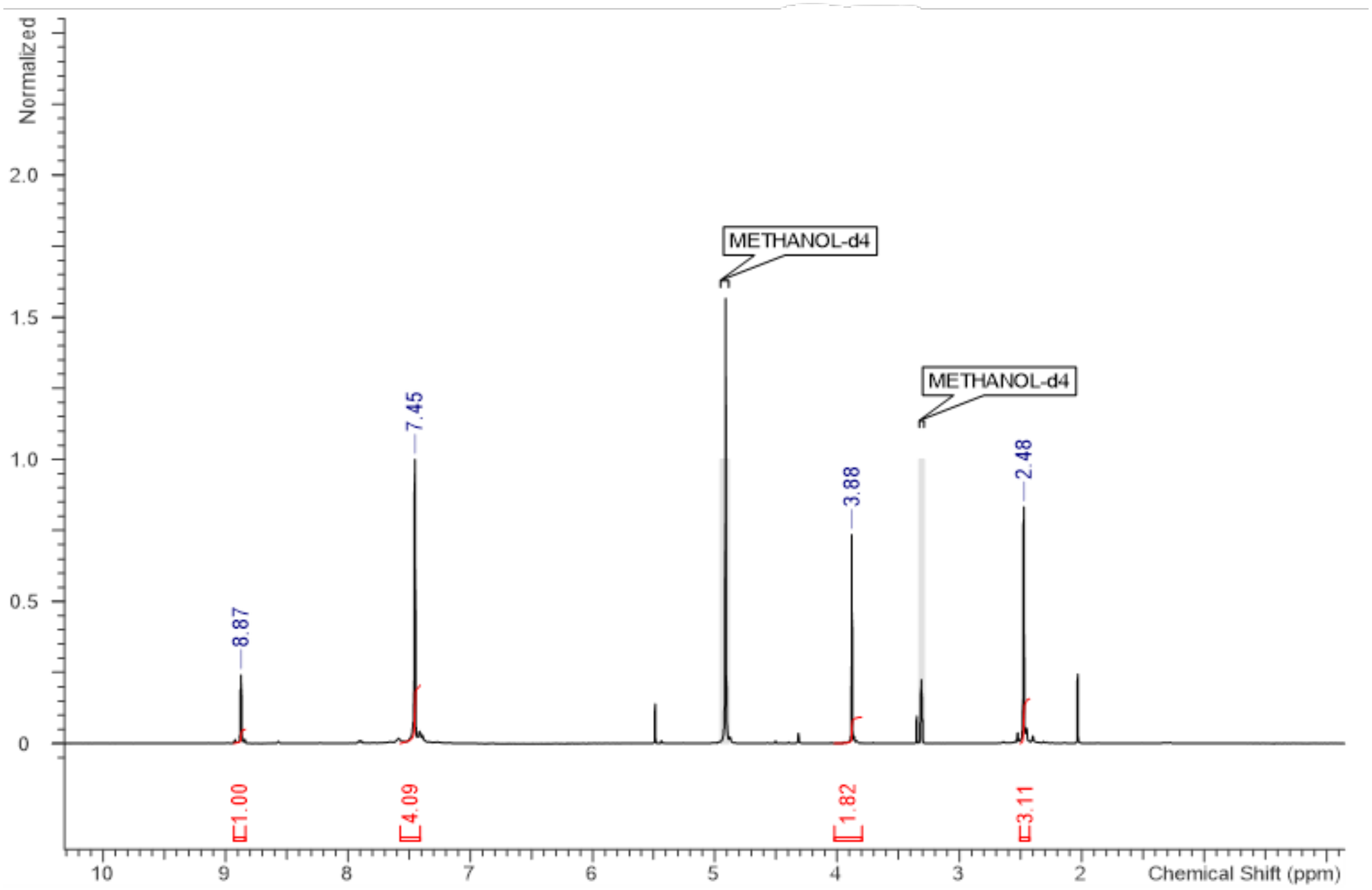

tert-butyl (2S,4R)-4-hydroxy-2-((4-(4-methylthiazol-5-yl)benzyl)carbamoyl)pyrrolidine-1-carboxylate

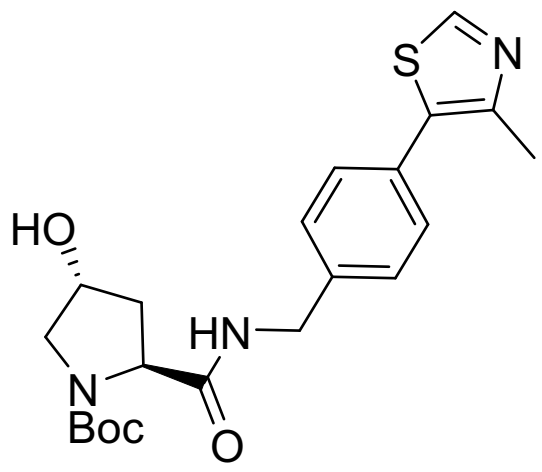

(2S,4R)-1-(tert-butoxycarbonyl)-4-hydroxypyrrolidine-2-carboxylic acid (793 mg, 3.43 mmol, 1.2 eq), HATU (1.6 g, 4.3 mmol, 1.5 eq) and DIPEA (1.96 mL, 11.44 mmol, 3 eq) was added to a solution of DMF (10 mL). A solution of (4-(4-methylthiazol-5-yl)phenyl)methanamine (584 mg, 2.86 mmol) dissolved in DMF (10 mL) was subsequently added. The solution was stirred overnight at ambient temperature and was quenched with water. The aqueous phase was extracted with EtOAc (x3) and the combined organic extracts washed in brine. The combined organic extracts were dried (MgSO4), concentrated *in vacuo* and purified by Reverse phase chromatography and was eluted at 60% MeCN/TFA(0.1%) /40% Water. The desired fractions were then pooled, concentrated *in vacuo* and lyophilised. Tert-butyl (2S,4R)-4-hydroxy-2-((4-(4-methylthiazol-5-yl)benzyl)carbamoyl)pyrrolidine-1-carboxylate (**3c**) was afforded as an off-white solid (625 mg, 1.97 mmol, 69%).

^{1}H NMR (400 MHz, DMSO-d_6) δ 8.97 (s, 1 H) 8.43 - 8.50 (t, J = 5.99 Hz, 1 H) 7.32 - 7.45 (m, 4 H) 5.74 (s, 1 H) 4.98 - 5.01 (d, J = 3.42 Hz, 1 H) 4.32 - 4.40 (m, 1 H) 4.16 - 4.26 (m, 1 H) 4.14 - 4.27 (m, 3 H) 3.38 - 3.47 (m, 1 H) 3.31 (s, 7 H) 2.43 (m, 3 H) 2.09 - 2.15 (m, 1 H) 1.78 - 1.91 (m, 1 H) 1.40 and 1.25 (s, 9 H).
LR MS (ESI$^+$): m/z $[M+H]^+$ cald for $C_{21}H_{27}N_3O_4S_1$, 418.2, found; 418.3.

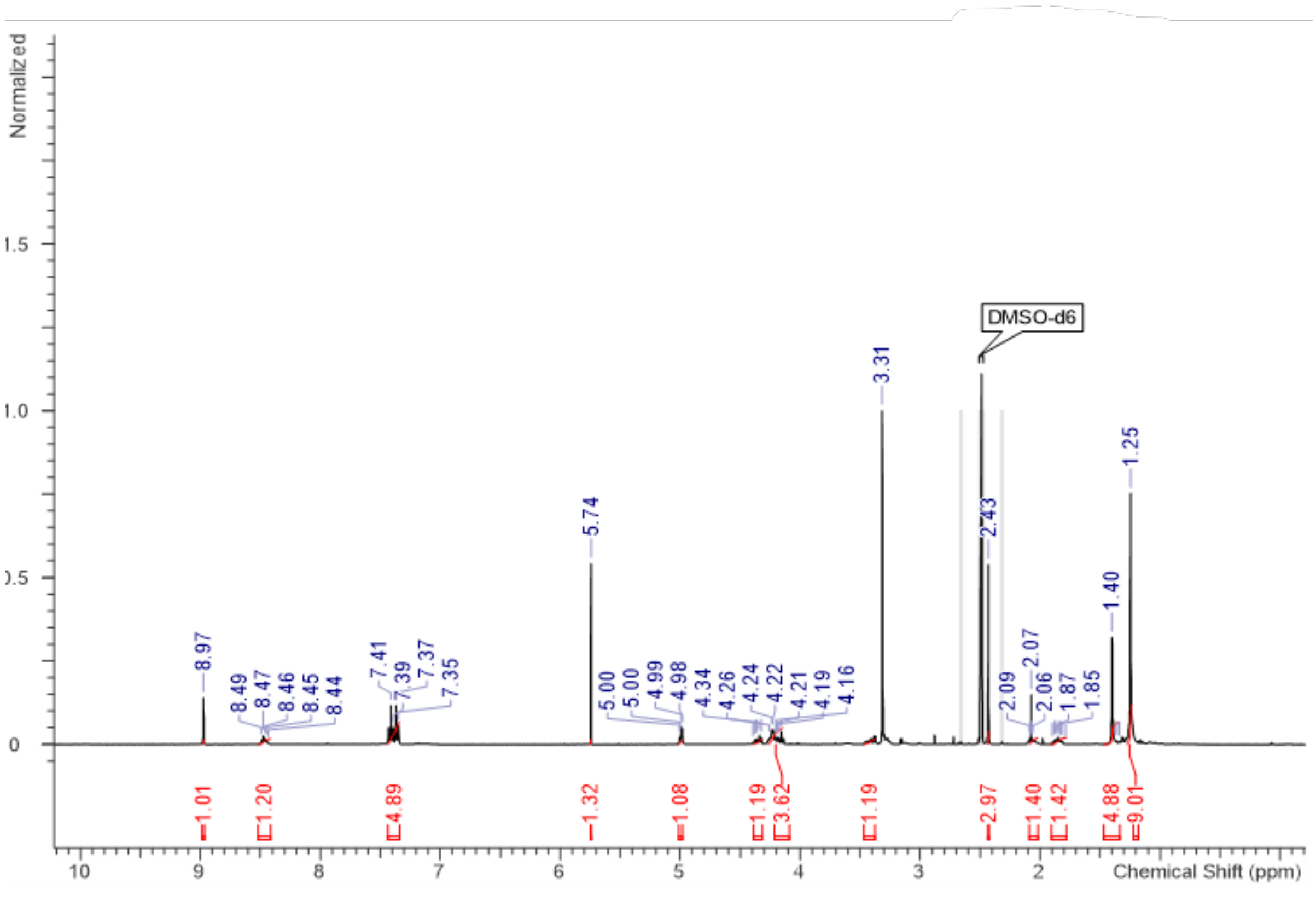

(2S,4R)-4-hydroxy-N-(4-(4-methylthiazol-5-yl)benzyl)pyrrolidine-2-carboxamide TFA salt

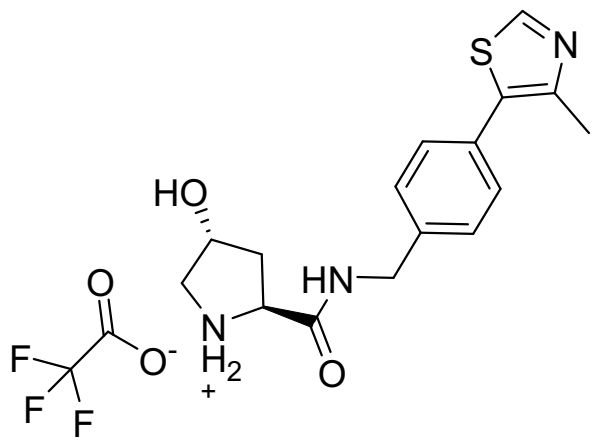

(2S,4R)-4-hydroxy-2-((4-(4-methylthiazol-5-yl)benzyl)carbamoyl)pyrrolidine-1-carboxylate (**3c**) was dissolved in a 1:1 TFA:DCM solution (10 mL) and was stirred at ambient temperature. The deprotection reaction was monitored by TLC until completion. Volatiles were removed by concentrating *in vacuo* to afford the (2S,4R)-4-hydroxy-N-(4-(4-methylthiazol-5-yl)benzyl)pyrrolidine-2-carboxamide TFA salt as a yellow oil in quantitative yield.

^{1}H NMR (400 MHz, METHANOL-d_4) δ ppm 9.35 (m, 1 H) 7.51 (q, *J*=8.42 Hz, 4 H) 4.61 (m, 1 H) 4.45 - 4.56 (m, 3 H) 3.48 – 3.34 (m, 2H) 2.53 (s, 3 H) 2.45 - 2.51 (m, 1 H) 1.97 - 2.16 – 2.00 (m, 1 H).
LR MS (ESI^{+}): m/z [M+H]$^{+}$ cald for $C_{16}H_{19}N_3O_2S_1$, 318.1, found; 318.3.

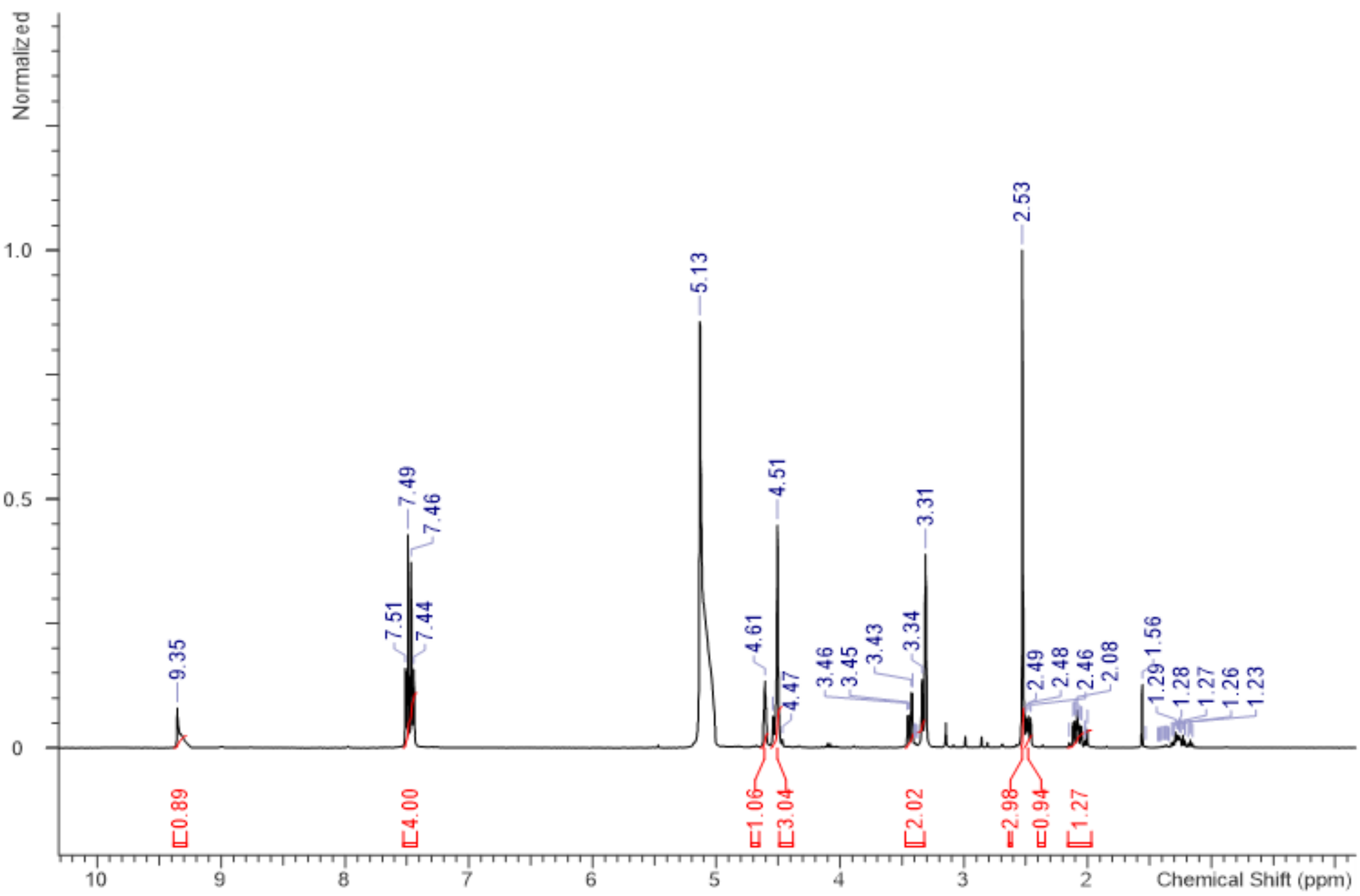

tert-butyl ((S)-1-((2S,4R)-4-hydroxy-2-((4-(4-methylthiazol-5-yl)benzyl)carbamoyl)pyrrolidin-1-yl)-3,3-dimethyl-1-oxobutan-2-yl)carbamate

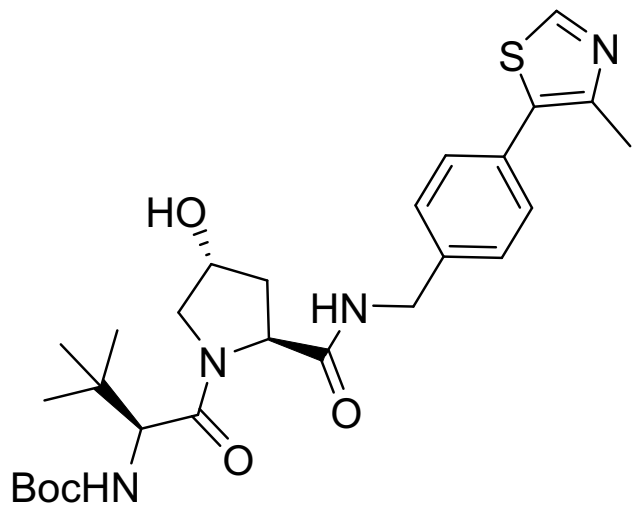

2S)-2-[[(tert-butoxy)carbonyl]amino]-3,3-dimethylbutanoic acid was dissolved in DMF (10 mL) followed by the addition of DIPEA (1.35 mL, 7.88 mmol, 3 eq) and HATU (1.12g, 2.96 mmol, 1.5 eq). (2S,4R)-4-hydroxy-N-(4-(4-methylthiazol-5-yl)benzyl)pyrrolidine-2-carboxamide TFA salt (1.97 mmol) was then dissolved in DMF and added to the reaction mixture. The solution was stirred over night at ambient temperature and was quenched with water. The aqueous phase was extracted with EtOAc (x3) and the combined organic extracts washed in brine. The combined organic extracts were dried (MgSO4), concentrated *in vacuo* and purified by Reverse phase chromatography and was eluted at 65% MeCN/TFA(0.1%) /35% H_2O/TFA (0.1%). The desired fractions were then pooled, concentrated *in vacuo* and lyophilised. tert-butyl ((S)-1-((2S,4R)-4-hydroxy-2-((4-(4-methylthiazol-5-yl)benzyl)carbamoyl)pyrrolidin-1-yl)-3,3-dimethyl-1-oxobutan-2-yl)carbamate was afforded as an off-white solid (491mg, 0.93 mmol, 47%).

^{1}H NMR (400 MHz, METHANOL-d_4) δ ppm 9.06 (m, 1 H) 7.49 (d, *J*=9.09 Hz, 4 H) 4.50 - 4.73 – 4.51 (m, 3 H) 4.38 - 4.29 (m, 1 H) 3.95 - 3.71 (m, 2 H) 2.49 (m, 3 H) 2.31 - 2.19 (m, 1 H) 2.16 - 2.03 (m, 1 H) 1.46 - 1.44 (m, 9 H) 1.01 (s, 9 H).
LR MS (ESI$^+$): m/z [M+H]$^+$ cald for $C_{27}H_{38}N_4O_5S_1$, 531.3 found; 531.5.

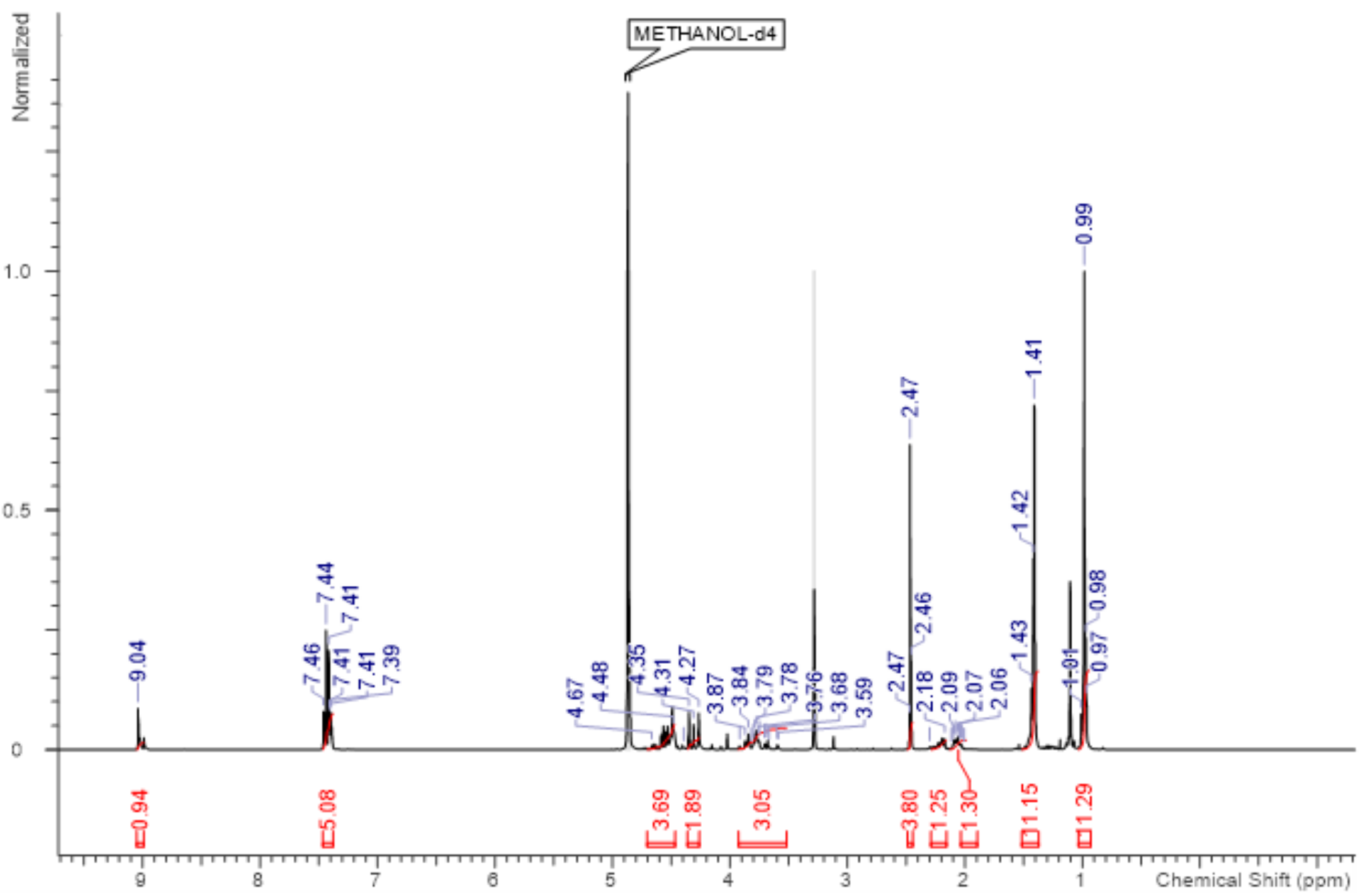

(2S,4R)-1-((S)-2-amino-3,3-dimethylbutanoyl)-4-hydroxy-N-(4-(4-methylthiazol-5-yl)benzyl)pyrrolidine-2-carboxamide

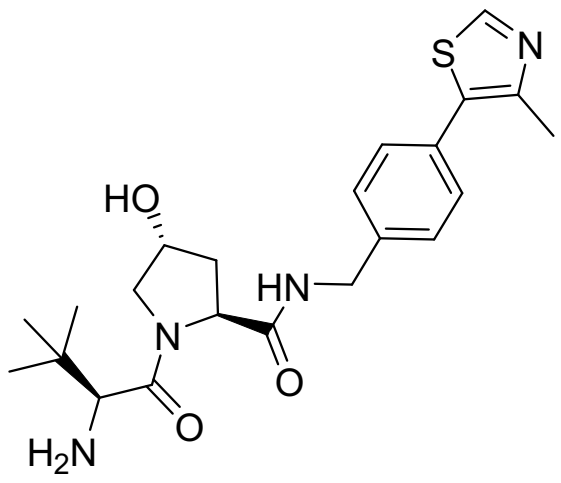

tert-butyl ((S)-1-((2S,4R)-4-hydroxy-2-((4-(4-methylthiazol-5-yl)benzyl)carbamoyl)pyrrolidin-1-yl)-3,3-dimethyl-1-oxobutan-2-yl)carbamate (491mg, 0.93 mmol) was dissolved in a 1:1 TFA:DCM (5 mL) solution and allowed to stir at ambient temperature. The deprotection reaction was monitored by TLC until completion. Volatiles were removed by concentrating *in vacuo* to afford the (2S,4R)-1-((S)-2-amino-3,3-dimethylbutanoyl)-4-hydroxy-N-(4-(4-methylthiazol-5-yl)benzyl)pyrrolidine-2-carboxamide TFA salt as a colourless oil. The compound was purified via Reverse phase chromatography and was eluted at 40% MeCN/TFA(0.1%) /60% H_2O/TFA 0.1%. affording (2S,4R)-1-((S)-2-amino-3,3-dimethylbutanoyl)-4-hydroxy-N-(4-(4-methylthiazol-5-yl)benzyl)pyrrolidine-2-carboxamide as a white crystalline solid (245 mg, 0.57mmol, 61%).

^{1}H NMR (400 MHz, METHANOL-d_4) δ ppm 9.28 (s, 1 H) 7.52 - 7.43 (m, 4 H) 4.68 (m, 1, H) 4.47 - 4.61 (m, 2 H) 4.35 (d, J=15.65 Hz, 1 H) 4.03 - 4.08 (m, 1 H) 3.80 - 3.88 (m, 1 H) 3.66 - 3.77 (m, 1 H) 2.51 (s, 3 H) 2.33 - 2.28 (m, 1 H) 2.13 - 2.06 (m, 1 H) 1.13 (s, 9H).
LR MS (ESI$^+$): m/z $[M+H]^+$ cald for $C_{22}H_{30}N_4O_3S_1$, 431.2, found; 431.2.
Characterisation of VHL ligand data matches with the reported literature by Buckley et al.

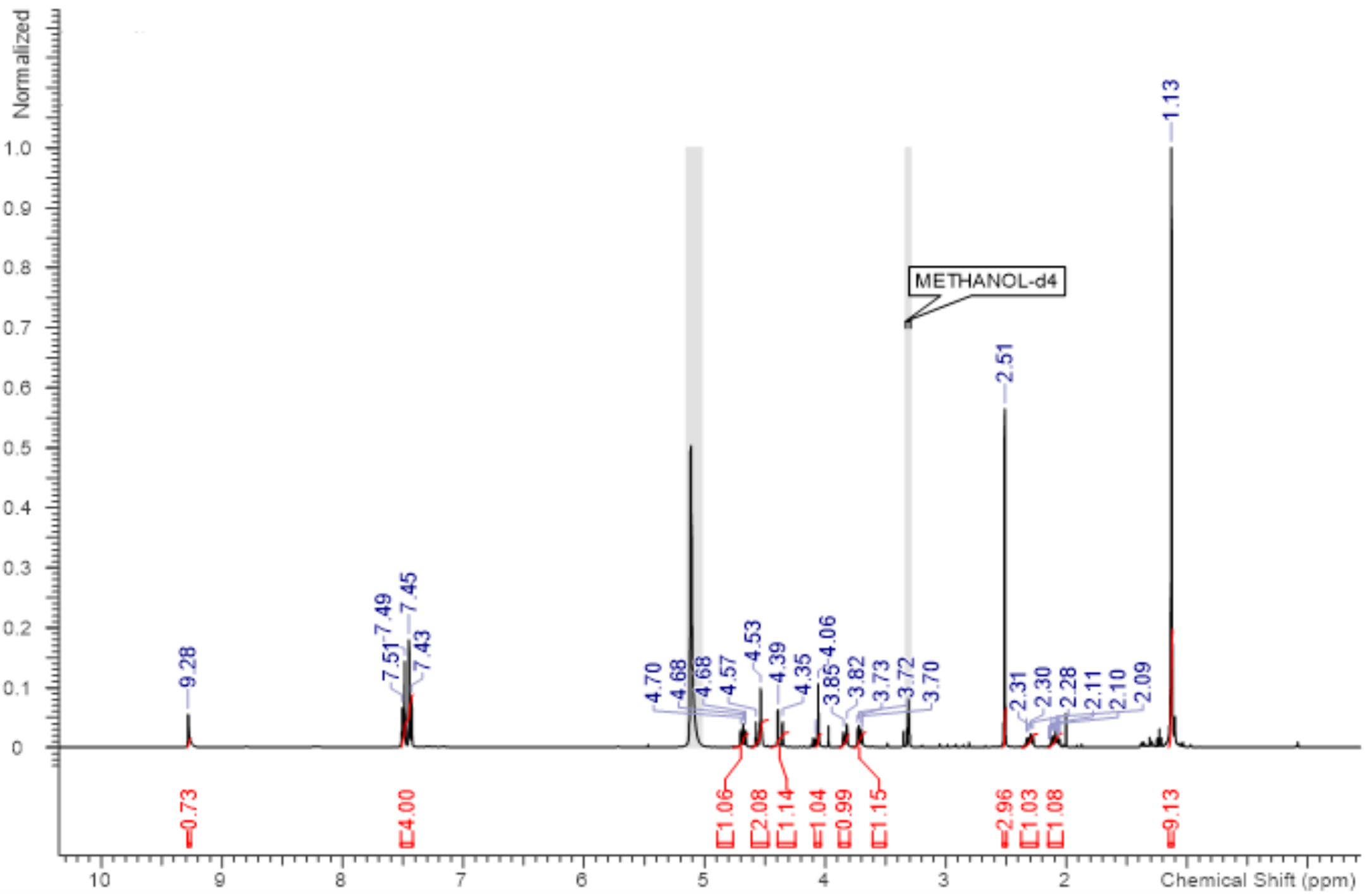

Benzyl (S)-2-((((9H-fluoren-9-yl)methoxy)carbonyl)amino)pent-4-enoate

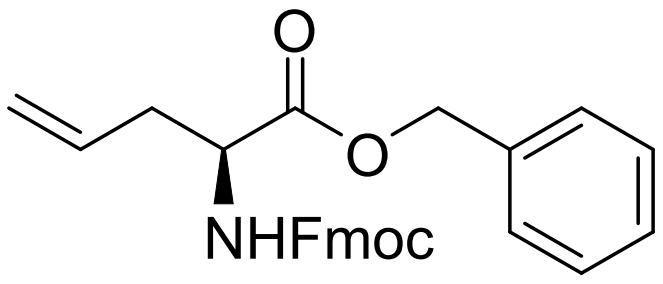

To a solution of Fmoc-allyl glycine (2 g, 5.93 mmol) dissolved in 20 mL of dry DMF was added $NaHCO_3$ (1.5 g, 17.8 mmol, 3 eq) whilst stirring. Benzyl bromide (0.78 mL, 6.5 mmol, 1.1 eq) was subsequently added drop wise to the mixture which was left to stir for 16 hrs at ambient temperature. The reaction was diluted with water (40 mL) followed by extraction with EtOAc (3 x 50 mL). The combined organic extracts were washed in brine (40 mL), dried (MgSO4), concentrated *in vacuo* and purified by flash chromatography (20% EtOAc/PE) affording (S)-2-(((9H-fluoren-9-yl)methoxy)carbonyl)amino)pent-4-enoic acid (2.0 g, 4.7 mmol, 79%) as a white solid.

^{1}H NMR (400 MHz, CHLOROFORM-*d*) δ ppm 7.77 (d, *J*=7.46 Hz, 1 H) 7.60 (br d, *J*=7.34 Hz, 2 H) 7.31 - 7.44 (m, 9 H) 5.63 - 5.73 (m, 1 H) 5.37 - 5.39 (m, 1 H) 5.10 - 5.25 (m, 4 H) 4.52-4.57 (m, 1 H) 4.40 (d, J=8.44 Hz, 2 H) 4.23 (t, J= 7.09 Hz, 1 H) 2.51 - 2.67 (m, 2 H).
LR MS (ESI$^+$): m/z $[M+H]^+$ cald for $C_{27}H_{25}NO_4$, 428.2, found; 428.4.

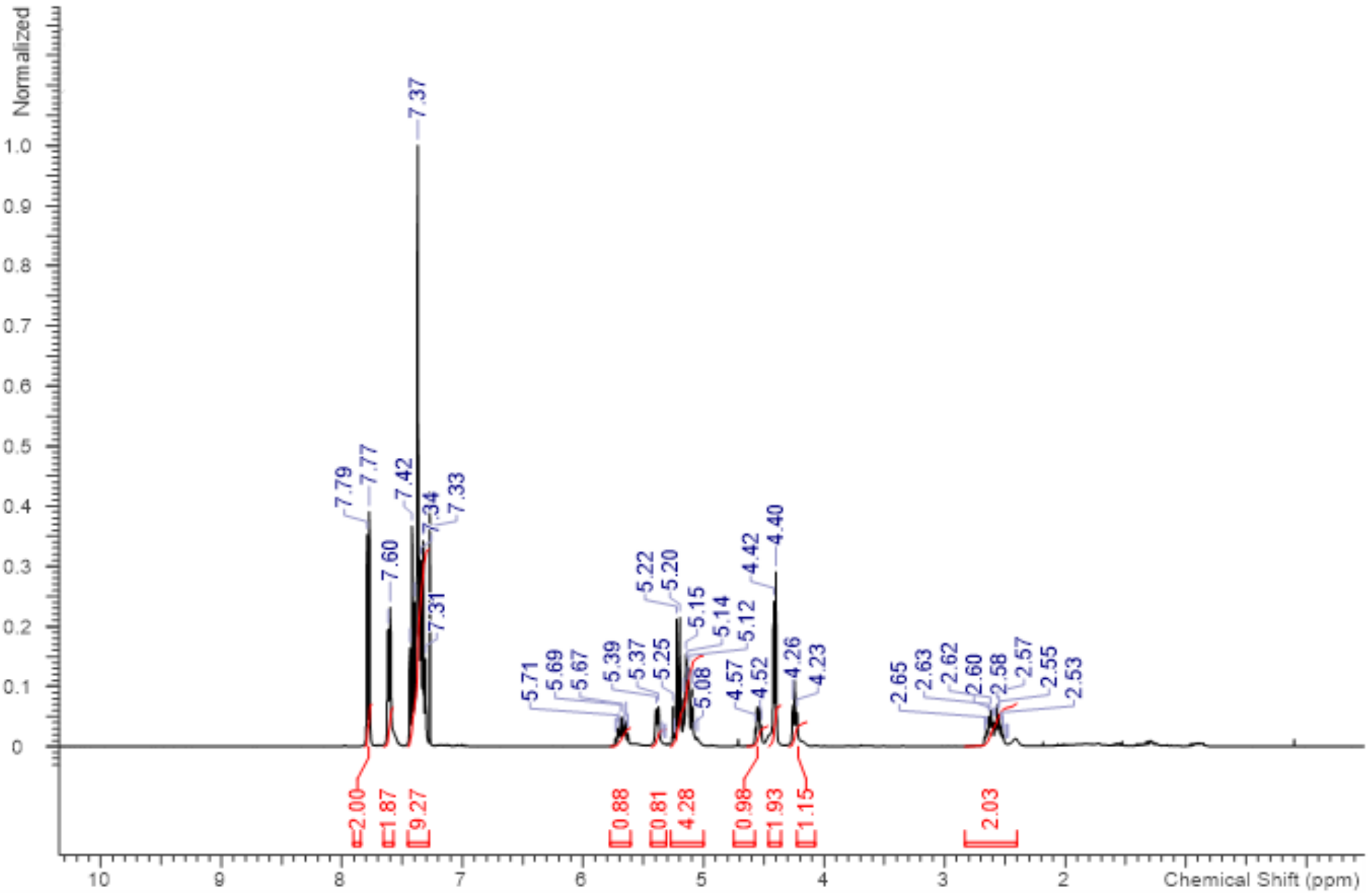

<u>6-benzyl 1-(tert-butyl) (S,E)-5-((((9H-fluoren-9-yl)methoxy)carbonyl)amino)hex-2-enedioate</u>

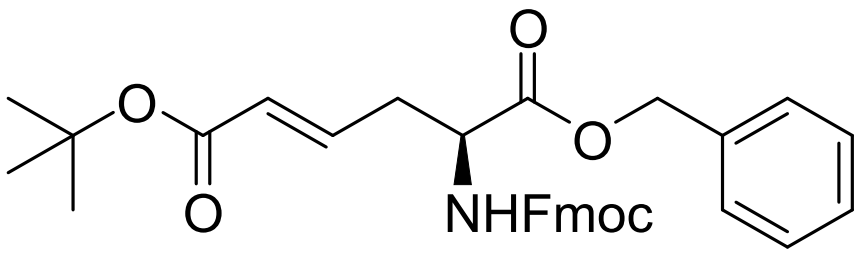

benzyl (S)-2-((((9H-fluoren-9-yl)methoxy)carbonyl)amino)pent-4-enoate (2 g, 4.7 mmol) was added dry DCM (20 mL) and was stirred until all solids were dissolved. The flask was purged with argon followed by the addition of Second-Generation Grubbs catalyst (236 mg, 0.235 mmol, 5%) and tert-butyl acrylate (6.8 mL, 47 mmol, 10 eq). The reaction was heated to reflux and stirred for 4 hrs under argon. The reaction was monitored by TLC for indication of completion. Reaction mixture was directly added to flash column for purification via flash chromatography and was eluted at 20% EtOAc/PE affording 6-benzyl 1-(tert-butyl) (S,E)-5-((((9H-fluoren-9-yl)methoxy)carbonyl)amino)hex-2-enedioate as a colourless oil (2 g mgs, 3.8 mmol, 81%).

^{1}H NMR (400 MHz, CHLOROFORM-*d*) δ p pm 7.77 (d, *J*=7.58 Hz, 2 H), 7.59 (br dd, *J*=7.46, 6.48 Hz, 2 H), 7.30 - 7.43 (m, 9 H), 6.67 – 6.75 (m, 1H), 5.77 (d, J= 15.28 Hz, 1 H), 5.44 (br d, *J*=7.46 Hz, 1 H) 5.17 - 5.24 (m, 2 H) 5.06 - 5.28 (m, 1 H) 4.54 - 4.61 (m, 1 H) 4.35 - 4.49 (m, 2 H) 4.21 (t, J=6.36 Hz, 1 H) 2.63 – 2.79 (m, 2 H), 1.48 (s, 9 H)
LR MS (ESI$^+$): m/z $[M+Na]^+$ cald for $C_{27}H_{25}NO_4$, 550.2, found; 550.3.

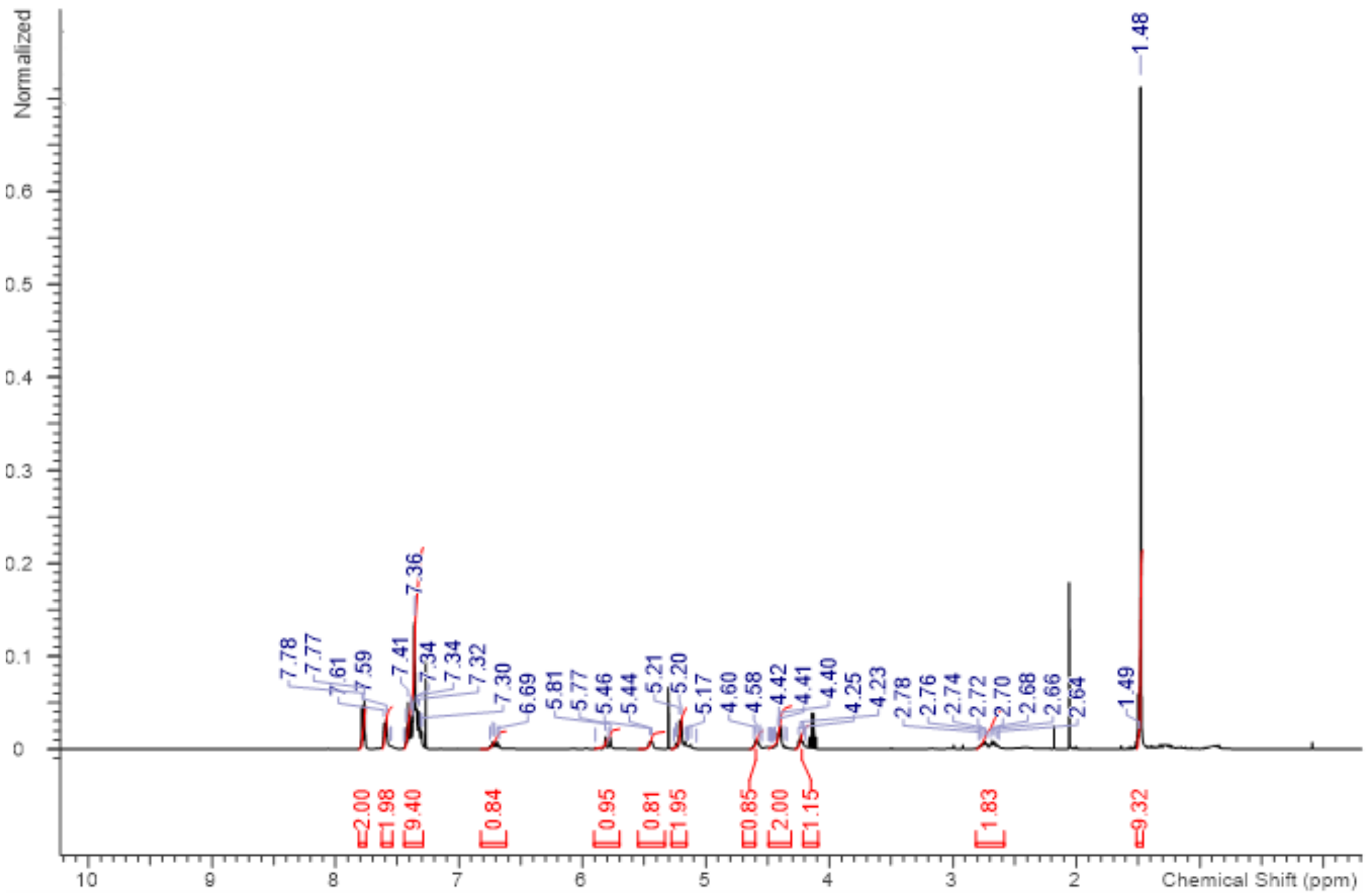

(S)-2-((((9H-fluoren-9-yl)methoxy)carbonyl)amino)-6-(tert-butoxy)-6-oxohexanoic acid

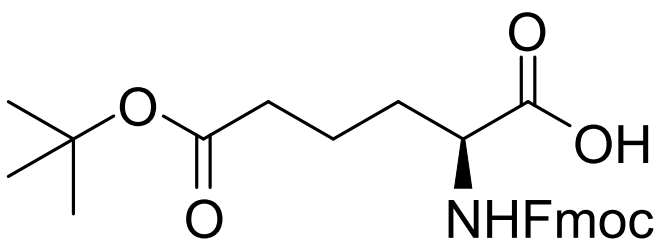

(S,E)-5-((((9H-fluoren-9-yl)methoxy)carbonyl)amino)-6-(benzyloxy)-6-oxohex-2-enoic acid (1 g, 1.9 mmol) was dissolved in MeOH (30 mL) and 10% Pd/C (100 mg) was added and stirred under a hydrogen atmosphere. The reaction was monitored by TLC till completion (2 h) which was then filtered through celite and concentrated in vacuo. The crude residue was purified by reverse phase chromatography eluting the product at 60% MeCN/TFA 0.1% and 40% H_2O/TFA 0.1%. The desired fractions were pooled, giving (S)-2-((((9H-fluoren-9-yl)methoxy)carbonyl)amino)-6-(tert-butoxy)-6-oxohexanoic acid (688 mgs, 1.56 mmol, 82%) as a white solid.

^{1}H NMR (400 MHz, DMSO-d_6) δ = 7.88 (d, *J* = 7.5 Hz, 2H), 7.72 (br d, *J* = 7.5 Hz, 2H), 7.65 (br d, *J* = 8.1 Hz, 1H), 7.43 - 7.40 (t, J= , 2H), 7.34 - 7.31 (m, J= , 2H), 4.34 - 4.16 (m, 3H), 4.03 - 3.86 (m, 1H), 2.25 - 2.11 (m, 2H), 1.80 - 1.49 (m, 4H), 1.39 (s, 8H), 1.43 - 1.34 (m, 1H).
LR MS (ESI$^+$): m/z [M+H]$^+$ cald for $C_{25}H_{29}NO_6$, 440.2, found; 440.3.

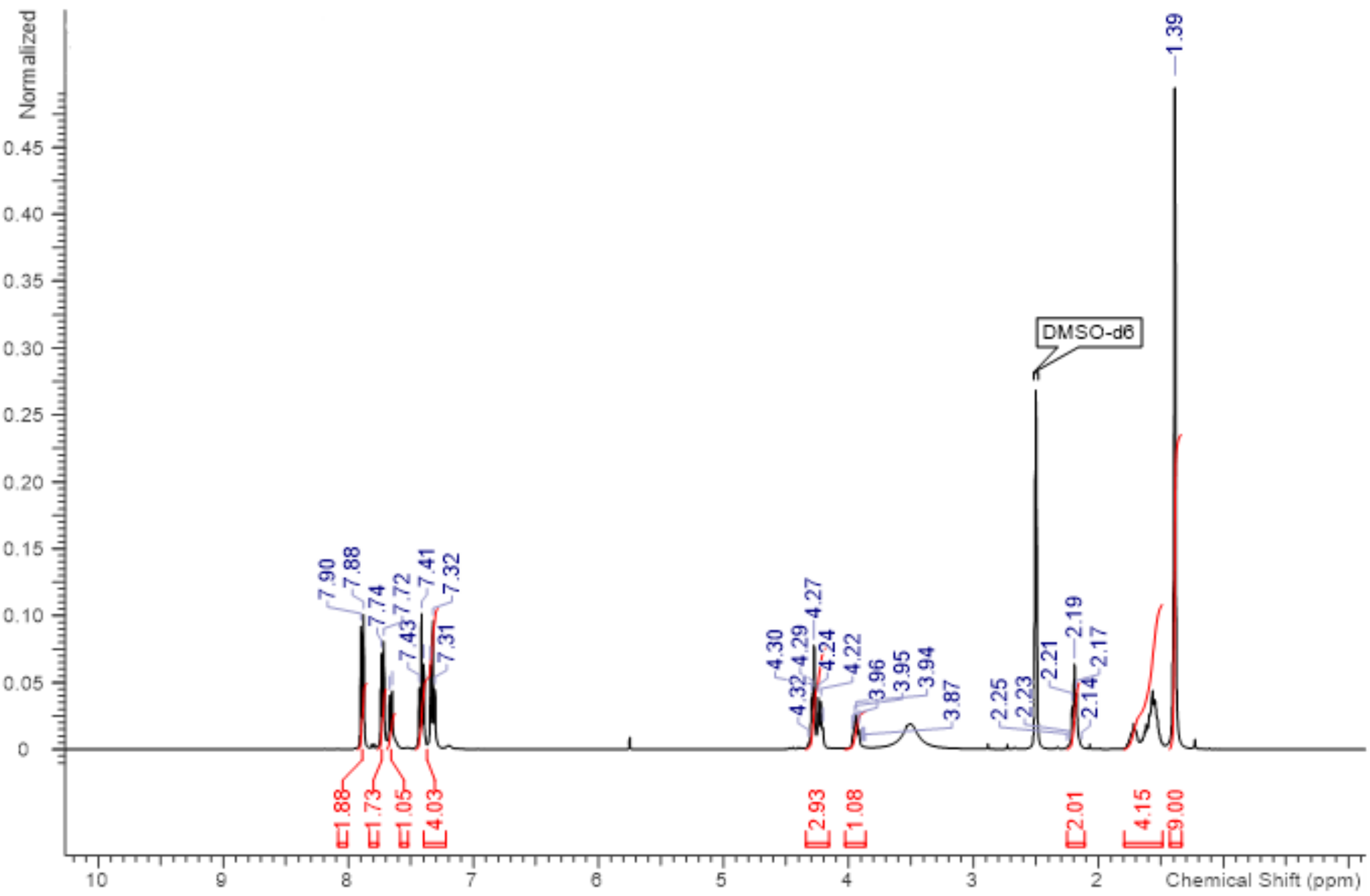

7-benzyl 1-(tert-butyl) (S,E)-6-((((9H-fluoren-9-yl)methoxy)carbonyl)amino)hept-3-enedioate

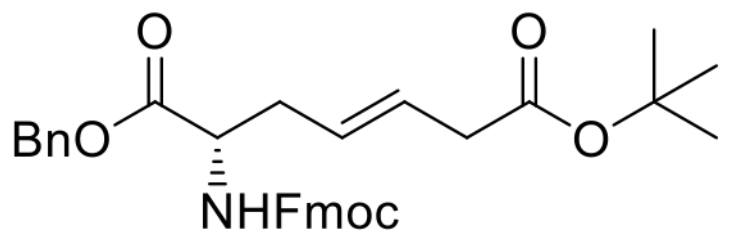

Procedure adapted from Voigtritter K et al.
Benzyl (S)-2-((((9H-fluoren-9-yl)methoxy)carbonyl)amino)pent-4-enoate (1 g, 2.3 mmol) was added to dry Et_2O (10 mL), heated to 40 °C and stirred until all solids were dissolved. Second-Generation Grubbs catalyst (44 mg, 0.07 mmol, 2 mol %), CuI (20 mg, 0.1 mmol, 3 mol %) and tert-butyl but-3-enoate (5 g, 35 mmol, 10 eq) were then added to the flask under argon and left to stir at reflux for 16 hrs. The reaction mixture was concentrated *in vacuo*, adsorbed onto silica gel, and loaded onto a manual chromatography column. The product was purified at 50% EtOAc/PET to afford 7-benzyl 1-(tert-butyl) (S,E)-6-((((9H-fluoren-9-yl)methoxy)carbonyl)amino)hept-3-enedioate **(1** as a colourless oil (872 mg, 1.6 mmol, 70%).

^{1}H NMR (400 MHz, CHLOROFORM-d) δ = 7.76 (d, *J* = 7.5 Hz, 2H), 7.61 (br d, *J* = 7.7 Hz, 2H), 7.43 - 7.29 (m, 9H), 5.82 - 5.33 (m, 3H), 5.24 - 5.16 (m, 2H), 4.53 - 4.49 (m, 1H), 4.43 - 4.35 (m, 2H), 4.25 - 4.21 (m, 1H), 2.98 – 2.96* (d, J = 7.7 Hz, H = 1), 2.91 (d, J = 6.60 H= 1), 2.67 - 2.53 (m, 2H), 1.47 - 1.43 (m, 9H). * cis/trans isomers
LR MS (ESI$^+$): m/z [M+H]$^+$ cald for $C_{33}H_{35}NO_6$: 542.3, found; 542.5

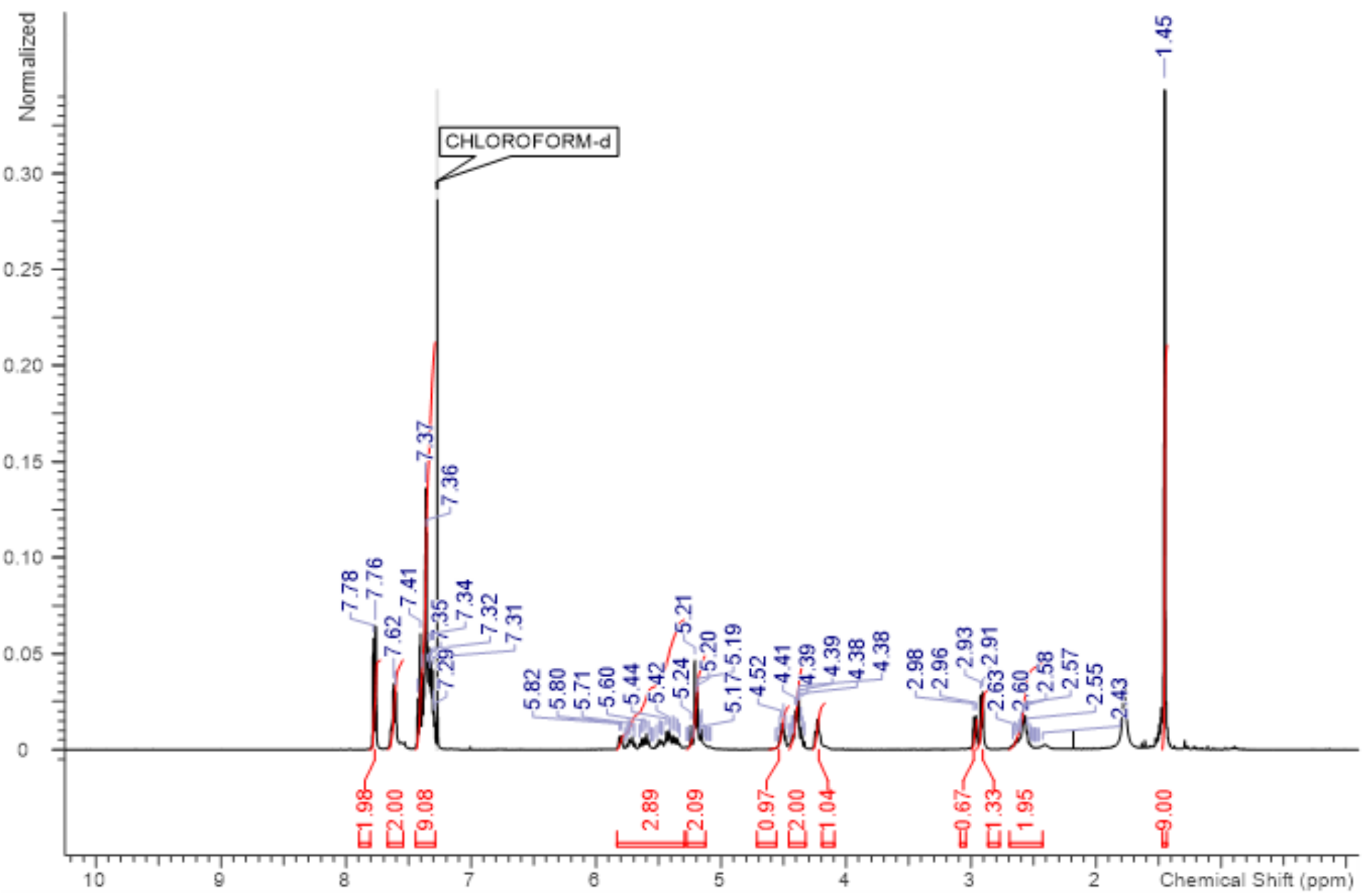

(S)-2-((((9H-fluoren-9-yl)methoxy)carbonyl)amino)-7-(tert-butoxy)-7-oxoheptanoic acid

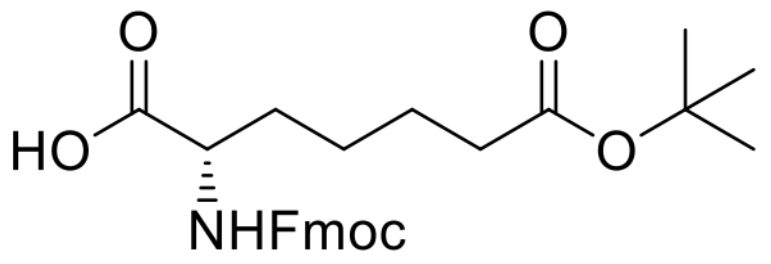

(7-benzyl 1-(tert-butyl) (S,E)-6-((((9H-fluoren-9-yl)methoxy)carbonyl)amino)hept-3-enedioate (872 mg, 1.6 mmol) was dissolved in MeOH (10 mL) and 10% Pd/C (80 mg) was added and stirred under a hydrogen atmosphere. The reaction was monitored by TLC till completion (2 h) which was then filtered through celite and concentrated *in vacuo*. The crude residue was purified by reverse phase chromatography eluting the product at 60% MeCN/TFA 0.1% and 40% H_2O/TFA 0.1%. The desired fractions were pooled and lyophilised to give (S)-2-((((9H-fluoren-9-yl)methoxy)carbonyl)amino)-7-(tert-butoxy)-7-oxoheptanoic acid (646 mg, 1.42 mmol, 89%) as a white solid.

^{1}H NMR (400 MHz, CHLOROFORM-d) δ = 8.02 (brs, 1H), 7.76 (d, *J* = 7.6 Hz, 2H), 7.61 - 7.53 (m, 2H), 7.42 - 7.40 (m, 1H), 7.38 (t, *J* = 7.5, Hz, 2H), 7.30 (t, *J* = 7.5, 1H), 5.42 (d, *J* = 8.2 Hz, 1H), 4.46 (m, 1H), 4.46 - 4.38 (m, 2H), 4.21 (t, *J* = 6.8 Hz, 1H), 2.22 (t, *J* = 7.6 Hz, 2H), 1.91 - 1.89 (m, 1H), 1.78 - 1.52 (m, 3H), 1.45 (s, 9H), 1.41 - 1.29 (m, 2H)
^{13}C NMR (101 MHz, CHLOROFORM-d) δ = 176.68, 173.28, 156.29, 143.83, 143.68, 141.32, 127.74, 127.10, 125.09, 120.00, 80.54, 67.15, 53.64, 47.14, 35.21, 31.94, 28.09, 24.69, 24.56
LR MS (ESI$^+$): m/z [M+H]$^+$ cald for $C_{26}H_{31}NO_6$, 454.2, found; 454.3

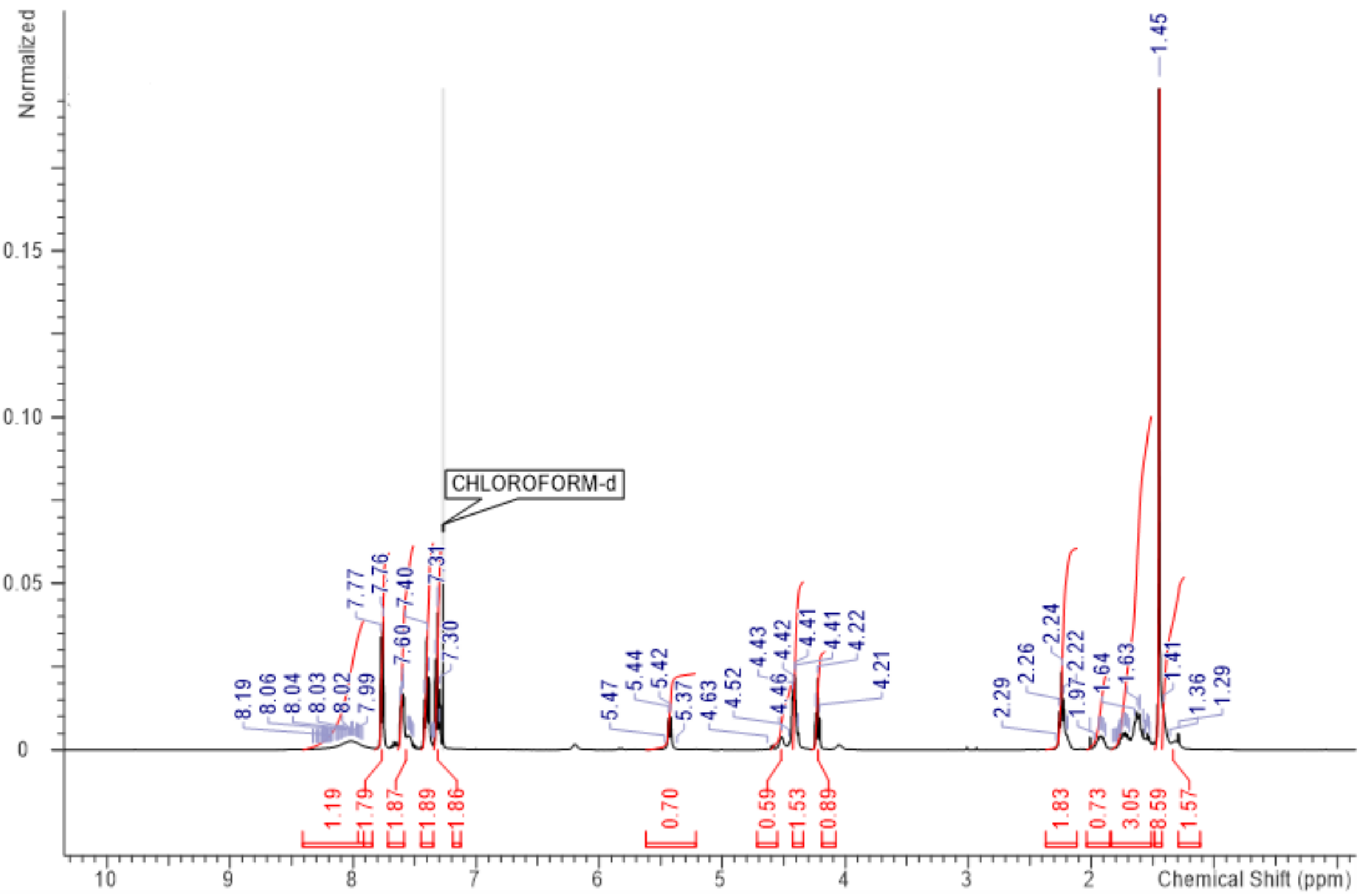

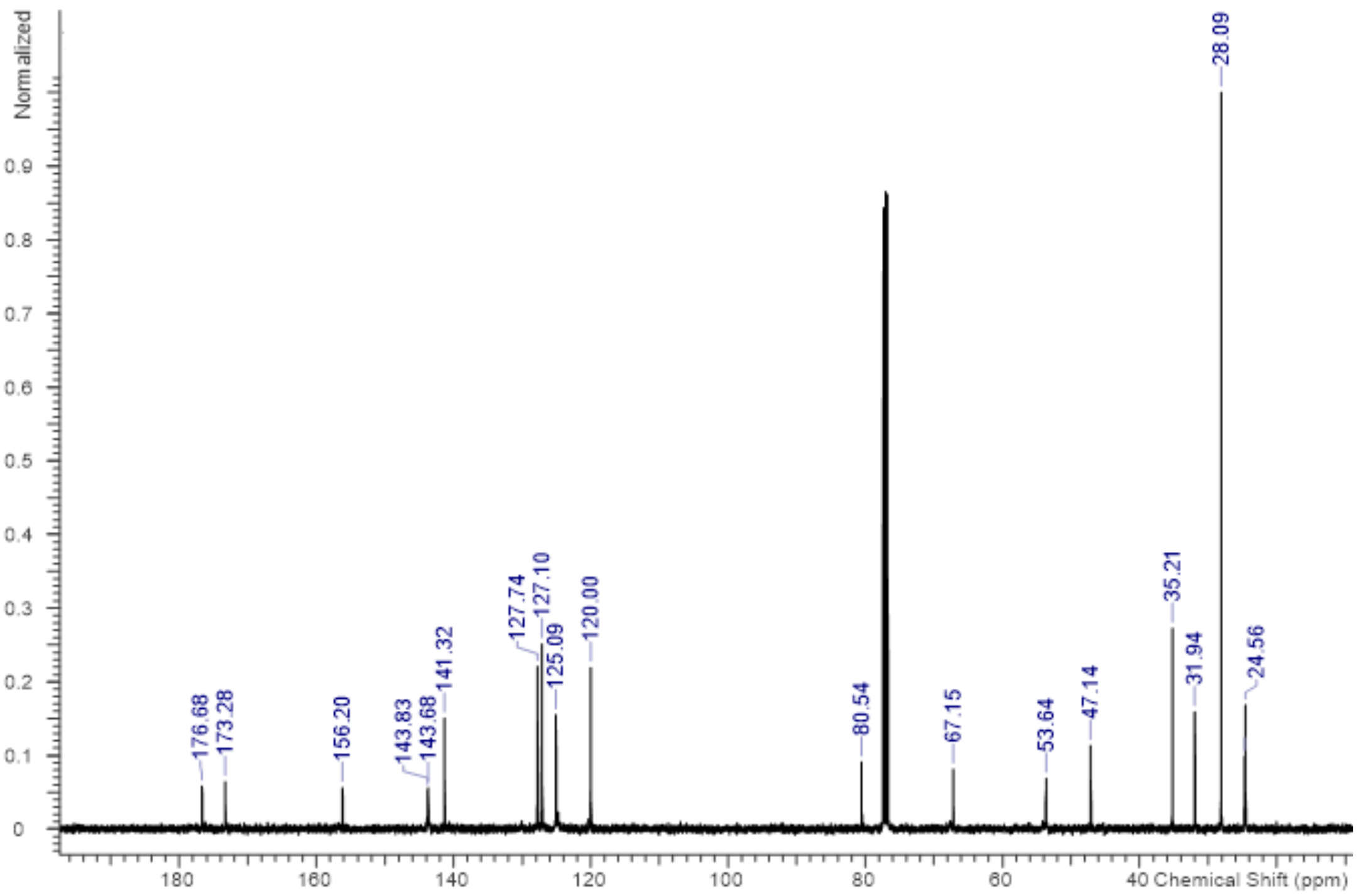

Normalized
0.9
0.8
0.7
0.6
0.5
0.4
0.3
0.2
0.1
0
176.68
173.28
156.20
143.83
143.68
141.32
127.74
127.10
125.09
120.00
80.54
67.15
53.64
47.14
35.21
31.94
28.09
24.56
180
160
140
120
100
80
60
40 Chemical Shift (ppm)

1-benzyl 8-(*tert*-butyl) (S,E)-2-((((9H-fluoren-9-yl)methoxy)carbonyl)amino)oct-4-enedioate

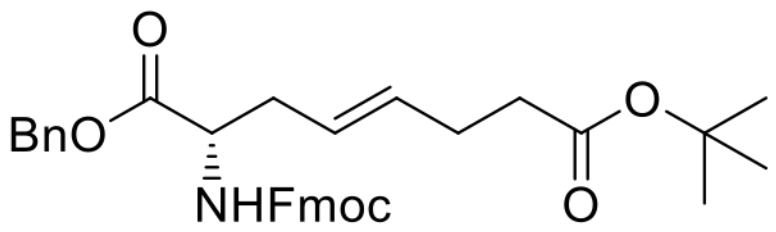

Benzyl (S)-2-((((9H-fluoren-9-yl)methoxy)carbonyl)amino)pent-4-enoate (1 g, 2.3 mmol) was added to dry Et_2O (10 mL), heated to 40 °C and stirred until all solids were dissolved. Second-Generation Grubbs catalyst (44 mg, 0.07 mmol, 2 mol %), CuI (20 mg, 0.1 mmol, 3 mol %) and tert-butyl pent-3-enoate (5 g, 35 mmol, 10 eq) were then added to the flask under argon and left to stir at reflux for 16 hrs. The reaction mixture was concentrated *in vacuo*, adsorbed onto silica gel, and loaded onto a manual chromatography column. The product was purified at 50% EtOAc/PET to afford 1-benzyl 8-*tert*-butyl) (S,E)-2-((((9H-fluoren-9-yl)methoxy)carbonyl)amino)oct-4-enedioate as a colourless oil (876 mg, 1.52 mmol, 66%).

^{1}H NMR (400 MHz, CHLOROFORM-d) 7.7 (d, *J* = 7.5 Hz, 2H), 7.60 (br d, *J* = 7.3 Hz, 2H), 7.29 (m, 9H), 5.47 (d, J = 9.3 Hz, 1H), 5.24 – 5.15 (m, 3H), 4.54 – 4.46 (m, 2H), 4.22 (t, J 7.2 Hz, 1H), 2.72 - 2.59 (m, 0.5H), 2.57 – 2.46 (m, 1.52H), 2.29 – 2.24 (m, 4H), 1.45 (s, 9H)*Rotamers
LR MS (ESI$^+$): m/z [M+H]$^+$ cald for $C_{34}H_{37}NO_6$, 556.3, found, 556.5 [M+H+].

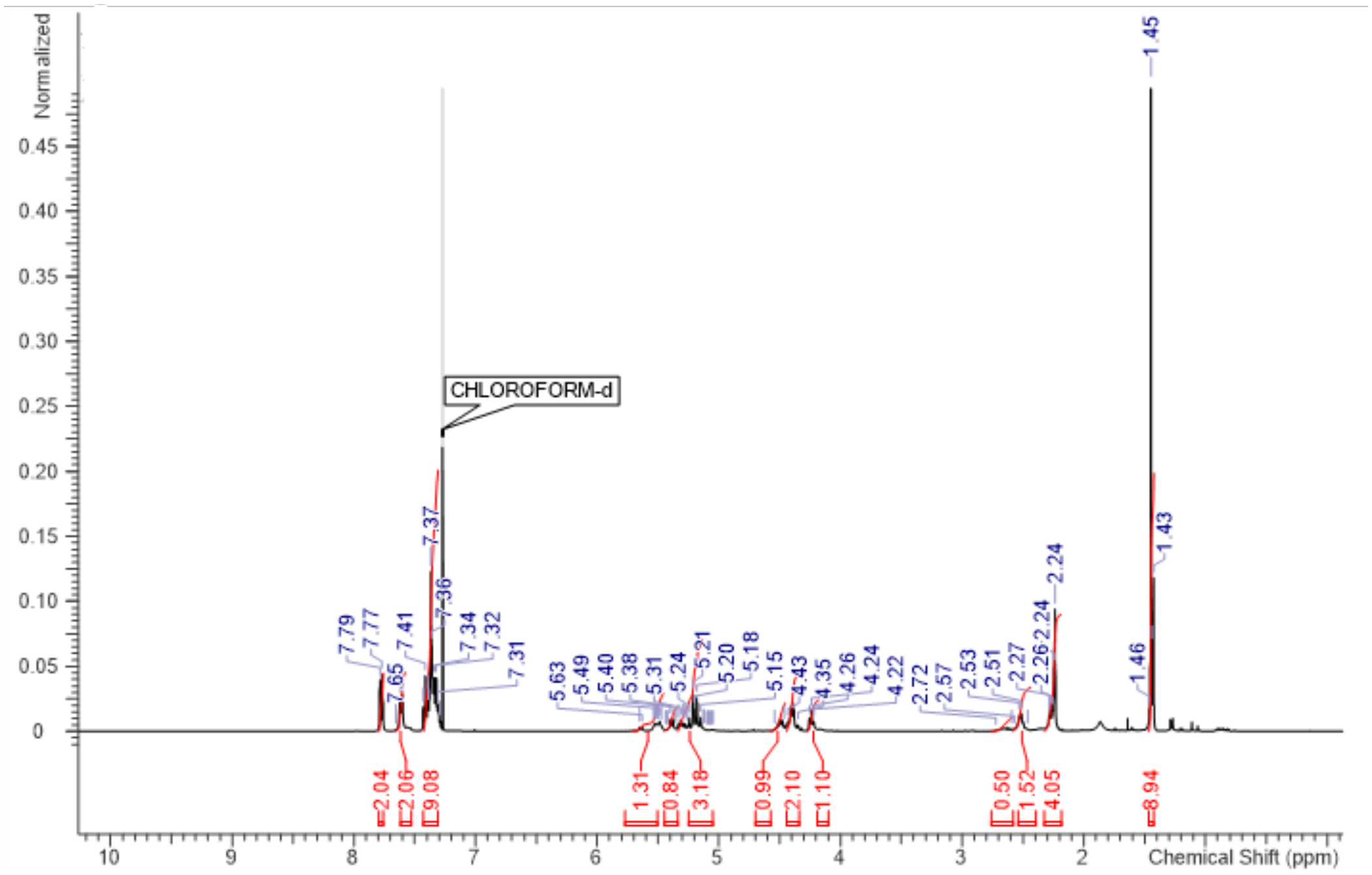

(S)-2-((((9H-fluoren-9-yl)methoxy)carbonyl)amino)-8-(tert-butoxy)-8-oxooctanoic acid

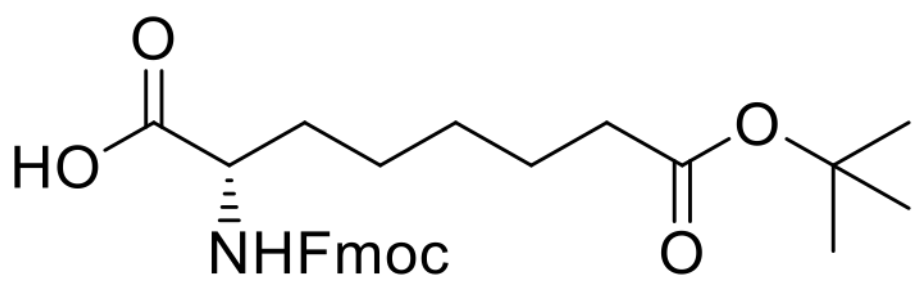

1-benzyl 8-(tert-butyl) (S,E)-2-((((9H-fluoren-9-yl)methoxy)carbonyl)amino)oct-4-enedioate (876 mg, 1.52 mmol) was dissolved in MeOH (10 mL) and 10% Pd/C (80 mg) was added and stirred under a hydrogen atmosphere. The reaction was monitored by TLC till completion (2 h) which was then filtered through celite and concentrated *in vacuo*. The crude residue was purified by reverse phase chromatography eluting the product at 60% MeCN/TFA 0.1% and 40% H_2O/TFA 0.1%. The desired fractions were pooled and lyophilised to give (S)-2-((((9H-fluoren-9-yl)methoxy)carbonyl)amino)-8-(tert-butoxy)-8-oxooctanoic acid (585 mg, 1.25 mmol, 82%) as a white solid.

^{1}H NMR (400 MHz, CHLOROFORM-d) δ = 8.54 (m, 1H), 7.67 (d, *J* = 7.5 Hz, 2H), 7.53 - 7.44 (m, 2H), 7.29 (t, *J* = 7.3 Hz, 2H), 7.21 (t, *J* = 7.5 Hz, 2H), 5.32 (d, *J* = 8.3 Hz, 1H), 4.46- 4.37 (m, 1H), 4.37 - 4.29 (m, 2H), 4.12 (t, *J* = 6.9 Hz, 1H), 2.12 (t, *J* = 7.5 Hz, 2H), 1.85 - 1.78 (m, 1H), 1.67 - 1.60 (m, 1H), 1.56 - 1.45 (m, 2H), 1.36 (s, 9H), 1.34 - 1.20 (m, 4H)
^{13}C NMR (101 MHz, CHLOROFORM-d) δ = 176.88, 173.54, 156.20, 143.84, 143.68, 141.32, 127.74, 127.10, 125.08, 120.00, 80.42, 67.14, 53.72, 47.15, 35.40, 32.11, 28.55, 28.11, 24.93, 24.75.
LR MS (ESI$^+$): m/z $[M+H]^+$ cald for $C_{27}H_{33}NO_6$, 468.2, found; 468.4

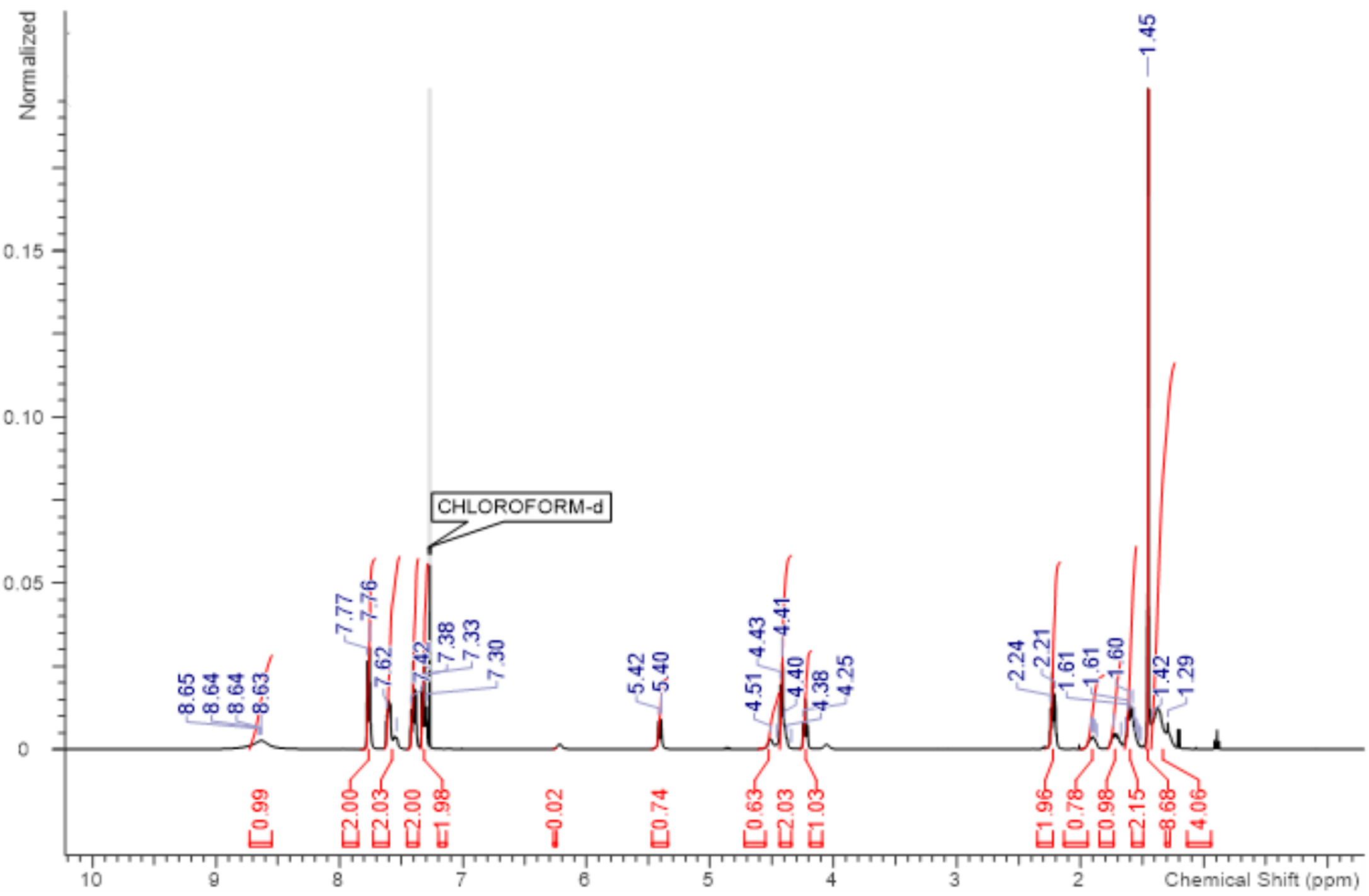

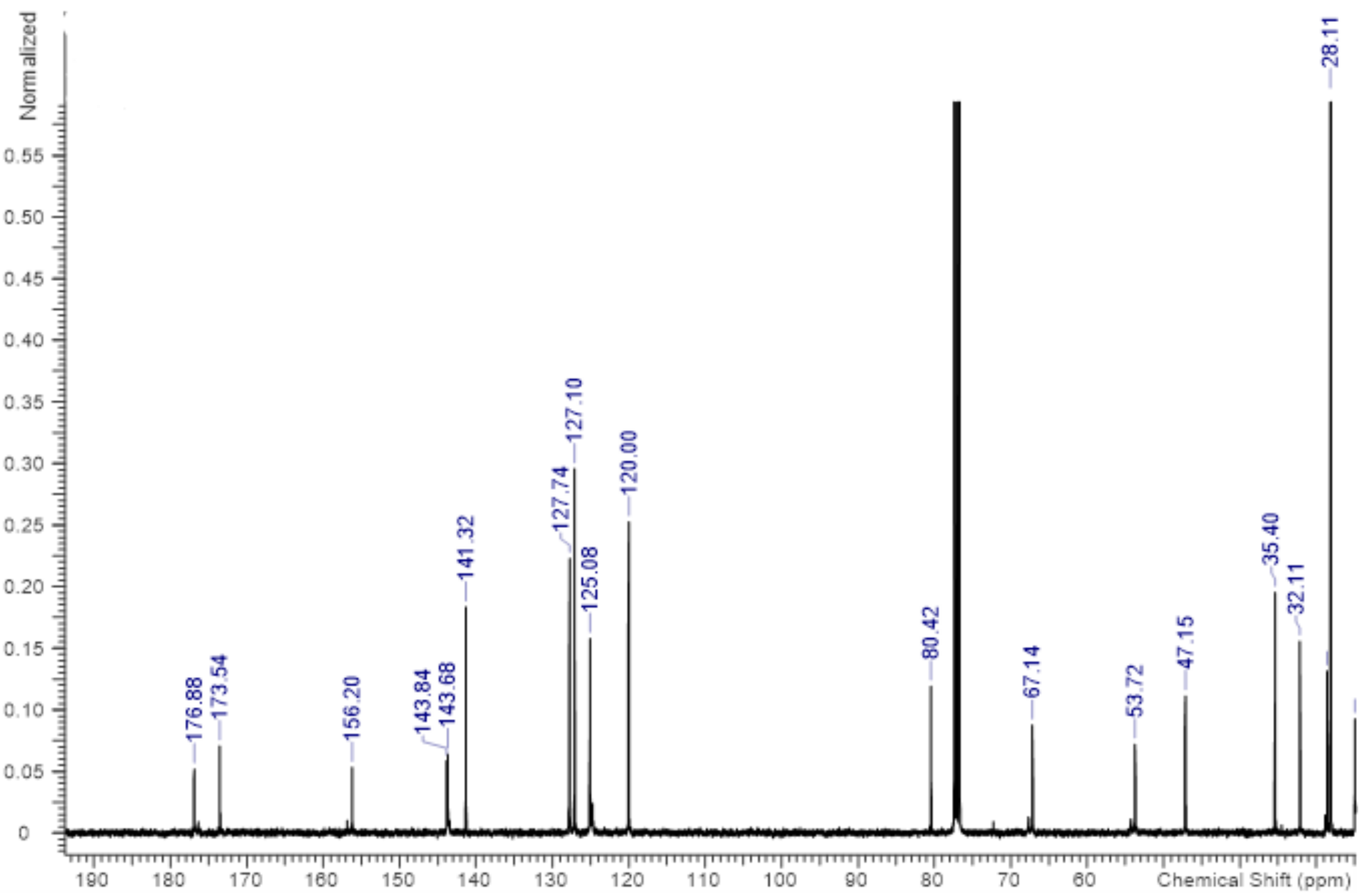

Normalized
0.55
0.50
0.45
0.40
0.35
0.30
0.25
0.20
0.15
0.10
0.05
0
176.88
173.54
156.20
143.84
143.68
141.32
127.74
127.10
125.08
120.00
80.42
67.14
53.72
47.15
35.40
32.11
28.11
190
180
170
160
150
140
130
120
110
100
90
80
70
60
Chemical Shift (ppm)

<u>1-benzyl 9-(tert-butyl) (E)-2-((((9H-fluoren-9-yl)methoxy)carbonyl)amino)non-4-enedioate</u>

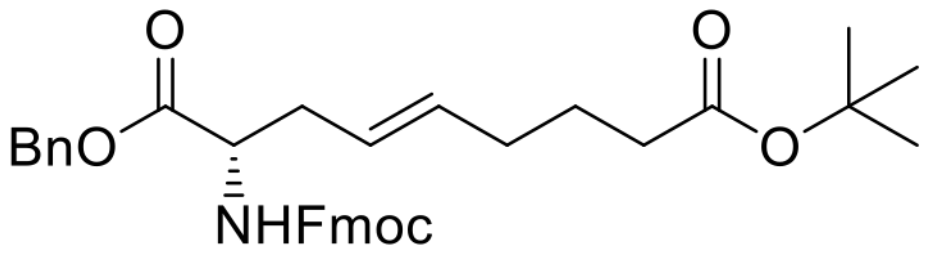

Benzyl (S)-2-((((9H-fluoren-9-yl)methoxy)carbonyl)amino)pent-4-enoate (1 g, 2.3 mmol) was added to dry Et_2O (10 mL), heated to 40 °C and stirred until all solids were dissolved. Second-Generation Grubbs catalyst (44 mg, 0.07 mmol, 2 mol %), CuI (20 mg, 0.1 mmol, 3 mol %) and tert-butyl hexan-3-enoate (5 g, 35 mmol, 10 eq) were then added to the flask under argon and left to stir at reflux for 16 hrs. The reaction mixture was concentrated *in vacuo*, adsorbed onto silica gel, and loaded onto a manual chromatography column. The product was purified at 50% EtOAc/PET to afford 1-benzyl 9-(tert-butyl) (E)-2-((((9H-fluoren-9-yl)methoxy)carbonyl)amino)non-4-enedioate as a colourless oil (845 mg, 1.48 mmol, 64%).

^{1}H NMR (400 MHz, CHLOROFORM-d) δ = 7.78 (d, *J* = 7.6 Hz, 2H), 7.62 (br d, *J* = 7.1 Hz, 2H), 7.43 - 7.30 (m, 9H), 5.58 - 5.39 (m, 2H), 5.33 - 5.24 (m, 1H), 5.21 - 5.15 (m, 2H), 4.55 - 4.46 (m, 1H), 4.41 - 4.34 (m, 2H), 4.25 – 4.22 (m, 1H), 2.67 - 2.42 (m, 2H), 2.21 - 2.17 (m, 2H), 2.04 - 1.97 (m, 2H), 2.10 - 1.82 (m, 2H), 1.67 - 1.55 (m, 2H), 1.45 (s, 9H)
LR MS (ESI$^+$): m/z $[M+H]^+$ cald for $C_{35}H_{39}NO_6$, 570.3, found; 570.5.

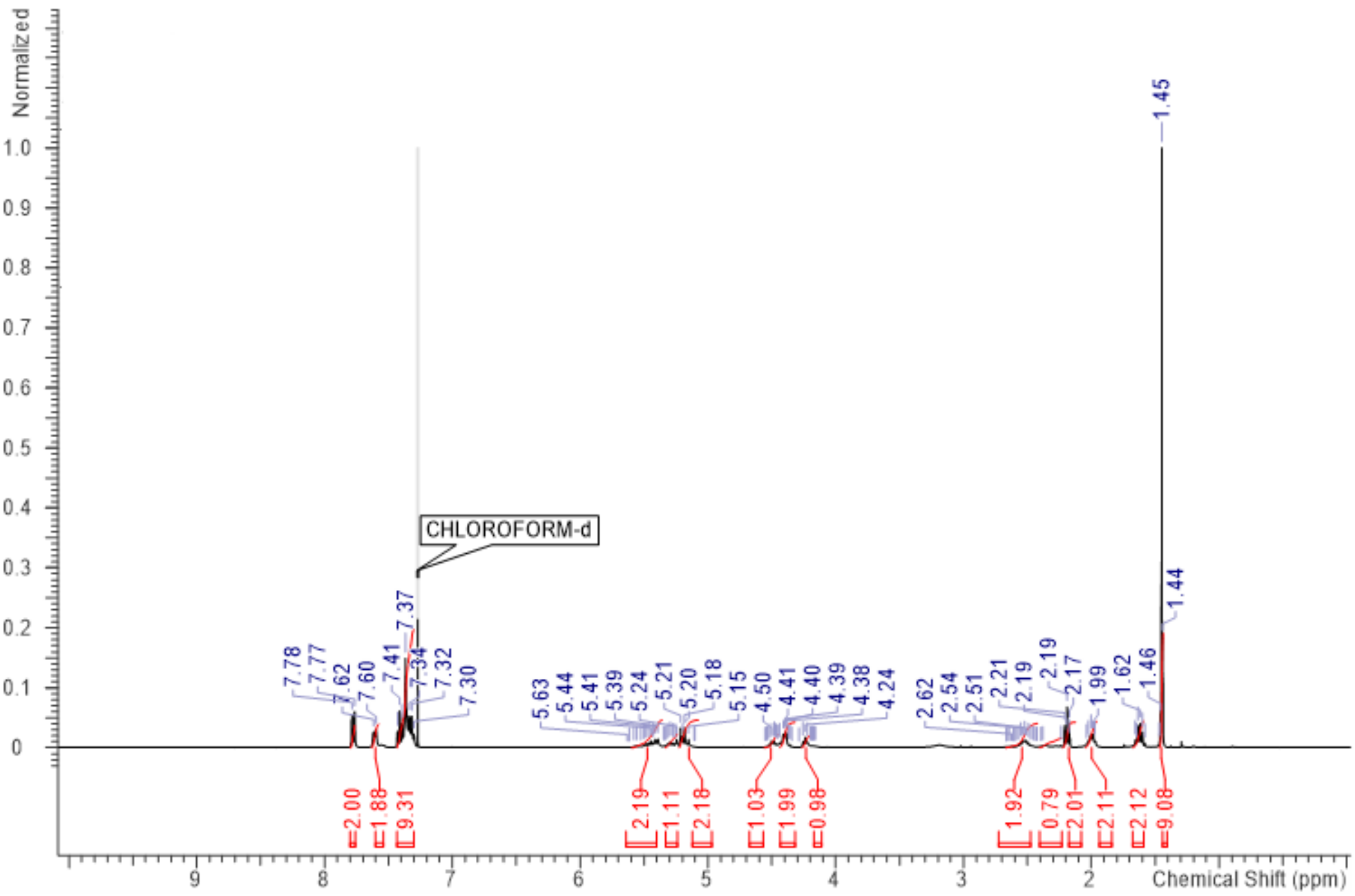

2-((((9H-fluoren-9-yl)methoxy)carbonyl)amino)-9-(tert-butoxy)-9-oxononanoic acid

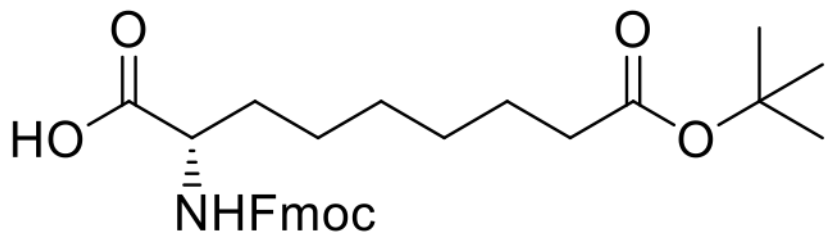

1-benzyl 9-(*tert*-butyl) (E)-2-((((9H-fluoren-9-yl)methoxy)carbonyl)amino)non-4-enedioate (845 mg, 1.48 mmol) was dissolved in MeOH (10 mL) and 10% Pd/C (80 mg) was added and stirred under a hydrogen atmosphere. The reaction was monitored by TLC till completion (2 h) which was then filtered through celite and concentrated *in vacuo*. The crude residue was purified by reverse phase chromatography eluting the product at 60% MeCN/TFA 0.1% and 40% H_2O/TFA 0.1%. The desired fractions were pooled and lyophilised to give 2-((((9H-fluoren-9-yl)methoxy)carbonyl)amino)-9-(tert-butoxy)-9-oxononanoic acid (613 mg, 1.27 mmol, 86%) as a white solid.

^{1}H NMR (400 MHz, CHLOROFORM-d) δ = 7.76 (d, *J* = 7.5 Hz, 2H), 7.61 - 7.54 (m, 2H), 7.39 (t, *J* = 7.6 Hz, 2H), 7.30 (t, *J* = 7.5 Hz, 2H), 5.32 (d, *J* = 8.1 Hz, 1H), 4.55 - 4.38 (m, 3H), 4.22 (t, *J* = 6.9 Hz, 1H), 2.20 (t, *J* = 7.5 Hz, 2H), 1.94 - 1.85 (m, 1H), 1.76 - 1.67 (m, 1H), 1.62 - 1.55 (m, 2H), 1.45 (s, 9H), 1.40 - 1.27 (m, 6H).
^{13}C NMR (101 MHz, CHLOROFORM-d) δ = 176.69, 173.59, 156.15, 143.82, 143.69, 141.32, 127.74, 127.09, 125.07, 120.01, 80.30, 67.14, 53.71, 47.15, 35.50, 32.18, 28.77, 28.74, 28.12, 24.99, 24.89.
LR MS (ESI$^+$): m/z [M+H]$^+$ cald for $C_{28}H_{35}NO_6$, 482.3, found, 482.4.

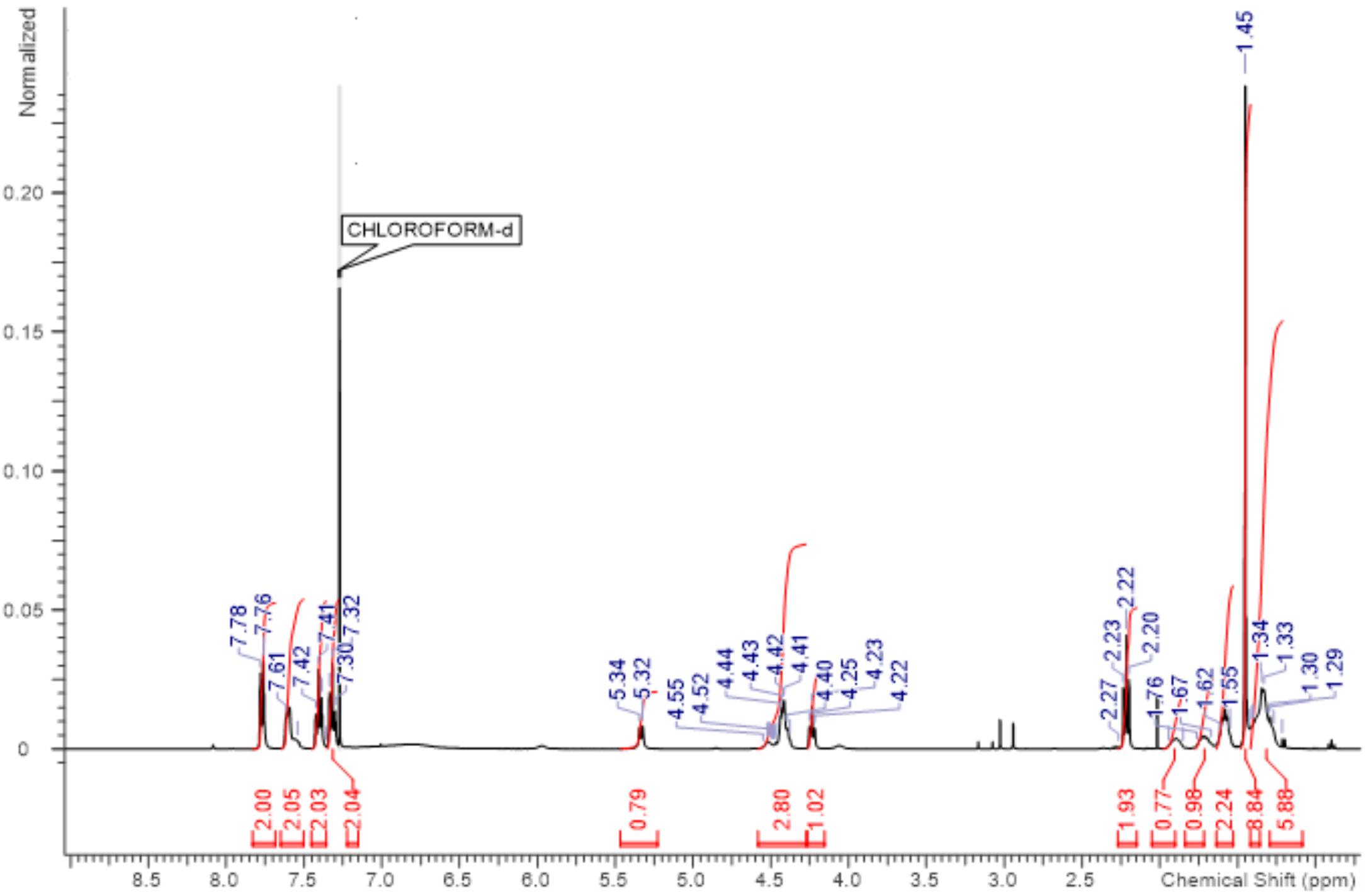

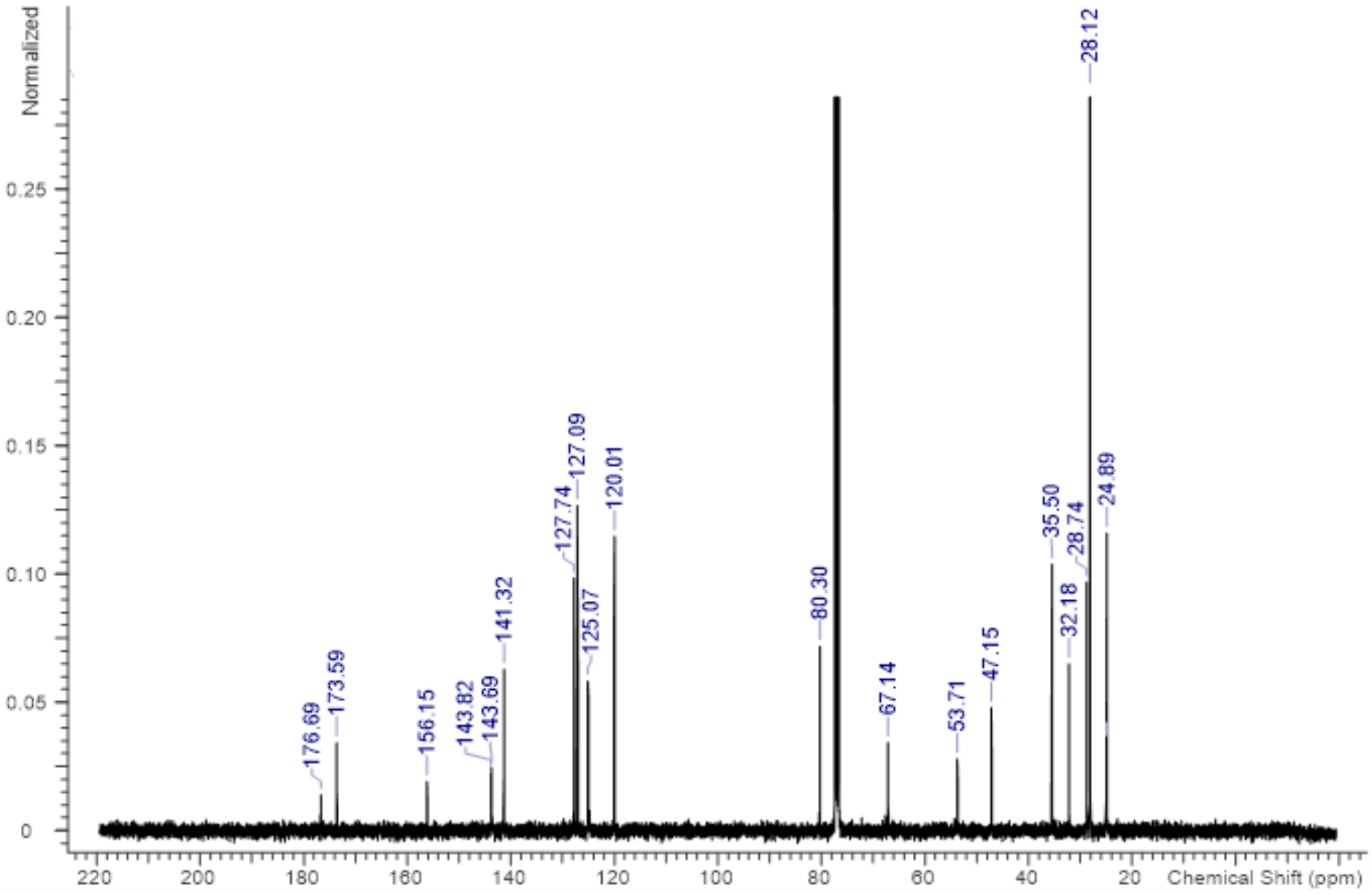

Normalized
0.25
0.20
0.15
0.10
0.05
0
176.69
173.59
156.15
143.82
143.69
141.32
127.74
127.09
125.07
120.01
80.30
67.14
53.71
47.15
35.50
32.18
28.74
28.12
24.89
220
200
180
160
140
120
100
80
60
40
20
Chemical Shift (ppm)

<u>*cyclo*-C(Tat)E(VHL)L(*p*-CF_3)FI(*p*-Br)F (**58**)</u>

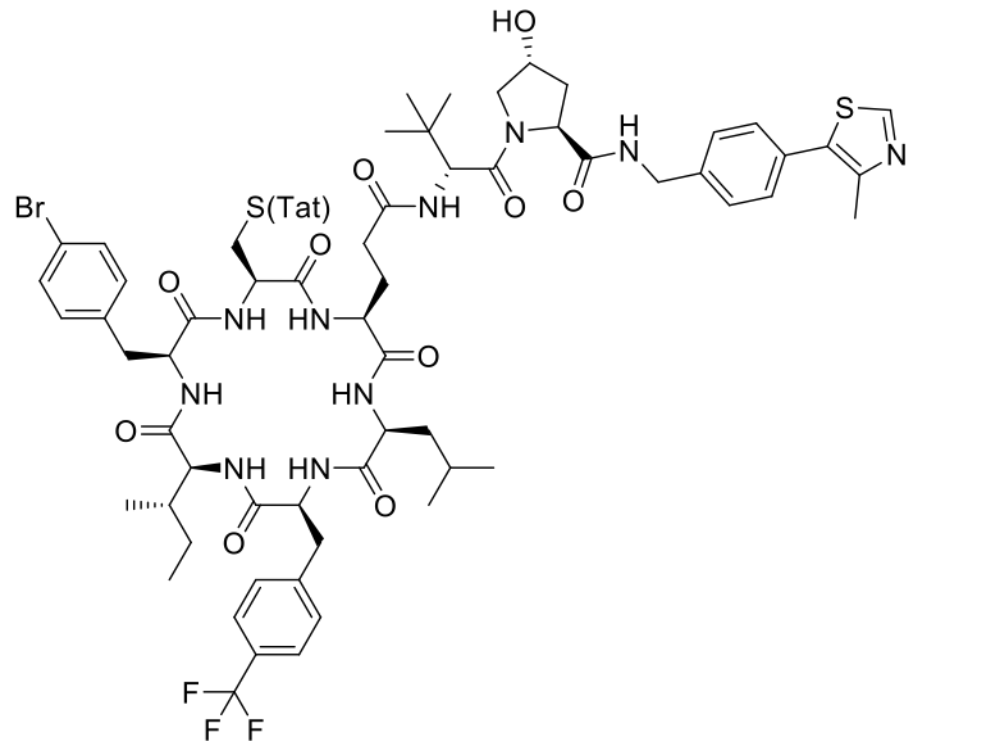

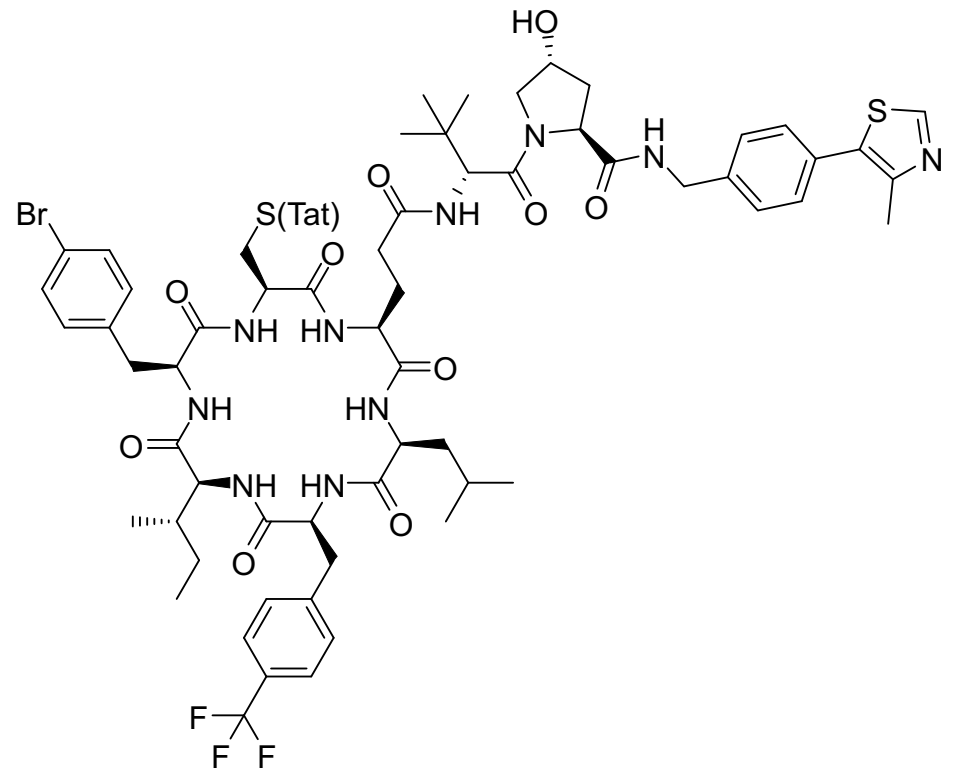

The Linear peptide LECF(Br)IF(CF_3) was synthesised using 2-CTC SPPS at a 0.2 mmol scale. The linear peptide was then cyclised and subjected to TFA deprotection. The crude was purified via reverse-phase flash chromatography to yield the StBu protected cyclic peptide C(StBu)ELF(CF_3)IF(Br) (31 mg, 0.03 mmol, 30%). The pure cyclic peptide (31 mg, 0.03 mmol) was dissolved in DMF (2 mL) to which was added DIPEA (16 µL, 0.09 mmol, 3 eq) and HATU (17 mg, 0.045 mmol, 1.5 eq) and left to stir for 10 minutes. VH032 (15 mg, 0.072 mmol, 1.2 eq) was added to the mixture and the reaction was left to stir overnight at ambient temperature and purified by reverse phase HPLC chromatography to give an impure StBu protected cyclic peptide PROTAC as a white solid (10.7 mg, 0.0076 mmol, 25%). The product was then StBu deprotected and purified via reverse-phase HPLC. Fractions were analysed via LC/MS, desired fractions were then pooled, concentration *in vacuo*, and lyophilised to yield the cyclic peptide PROTAC CE(VHL)LF(CF_3)IF(Br) as an off-yellow solid (5 mg, 0.0038 mmol, 51%). PROTAC CE(VHL)LF(CF_3)IF(Br) (4 mg, 0.0031mmol) was added to a solution of C(Spy)GRKKRRQRRRPPQ (9 mg, 0.0047 mmol, 1.5 eq) dissolved in DMF (2 mL) and stirred in an inert atmosphere overnight at ambient temperature. The mixture was then purified by reverse-phase HPLC. Fractions analysed via LC/MS, desired fractions were then pooled, concentrated *in vacuo*, and lyophilised to yield *Cyclo*- C(Tat)E(VHL)LF(CF_3)IF(Br) as a white solid (6.3 mg, 0.002, 65%).

LR MS (ESI^+): m/z $[M+H]^+$ cald for $C_{134}H_{212}BrF_3N_{46}O_{27}S_3$, 3129.5, found; 784.2 $[M+4H]^{4+}$, 627.83 $[M+5H]^{5+}$.
HRMS (ESI^+): shows expected model isotope pattern.
Analytical HPLC: Rt: (220 nm) 12.095 min (98% Purity).

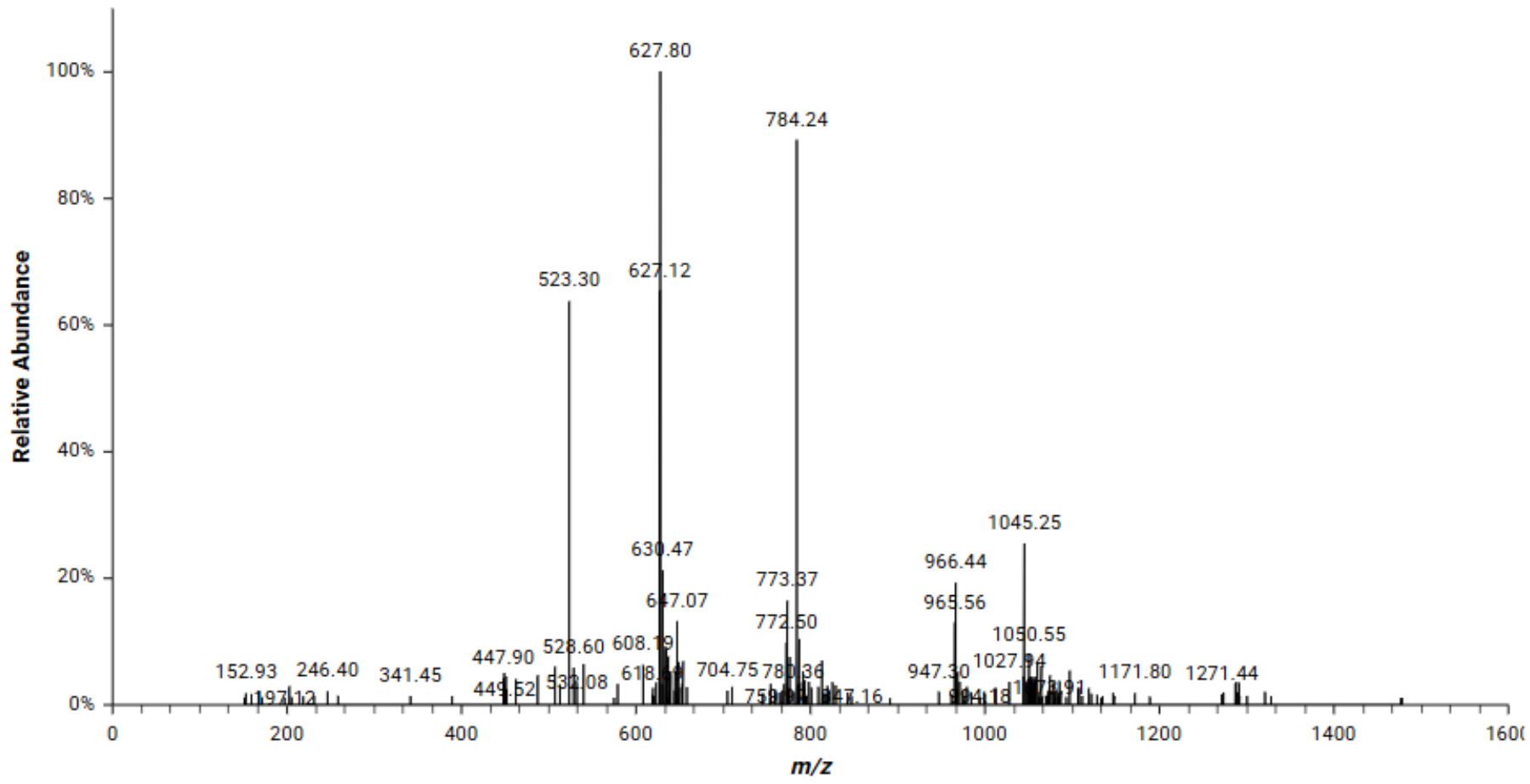

Relative Abundance
m/z
627.80
784.24
523.30
627.12
1045.25

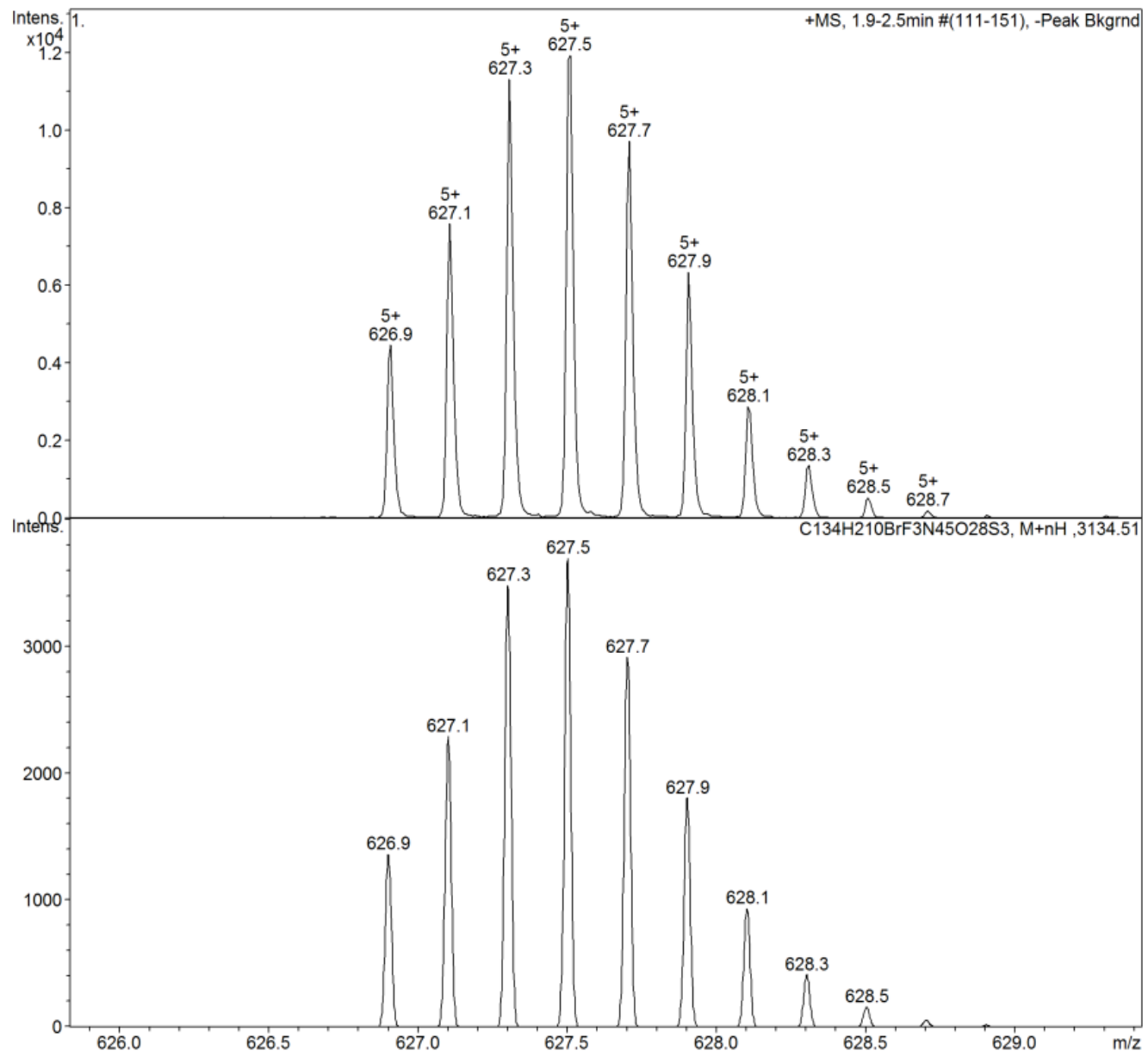

+MS, 1.9-2.5min #(111-151), -Peak Bkgrnd
C134H210BrF3N45O28S3, M+nH ,3134.51
m/z

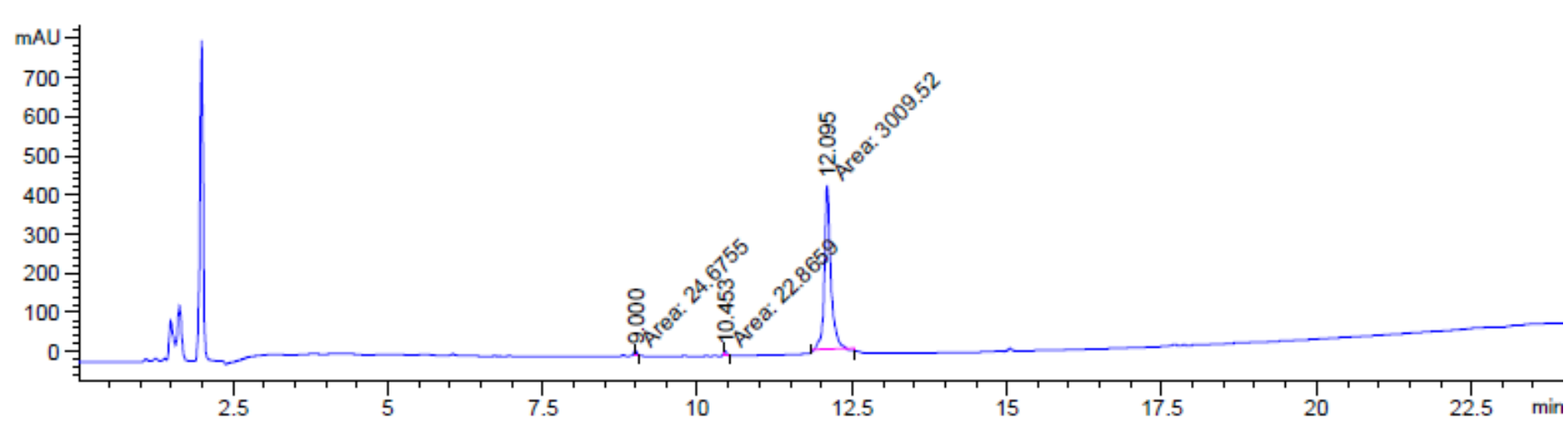

mAU
12.095
Area: 3009.52
min

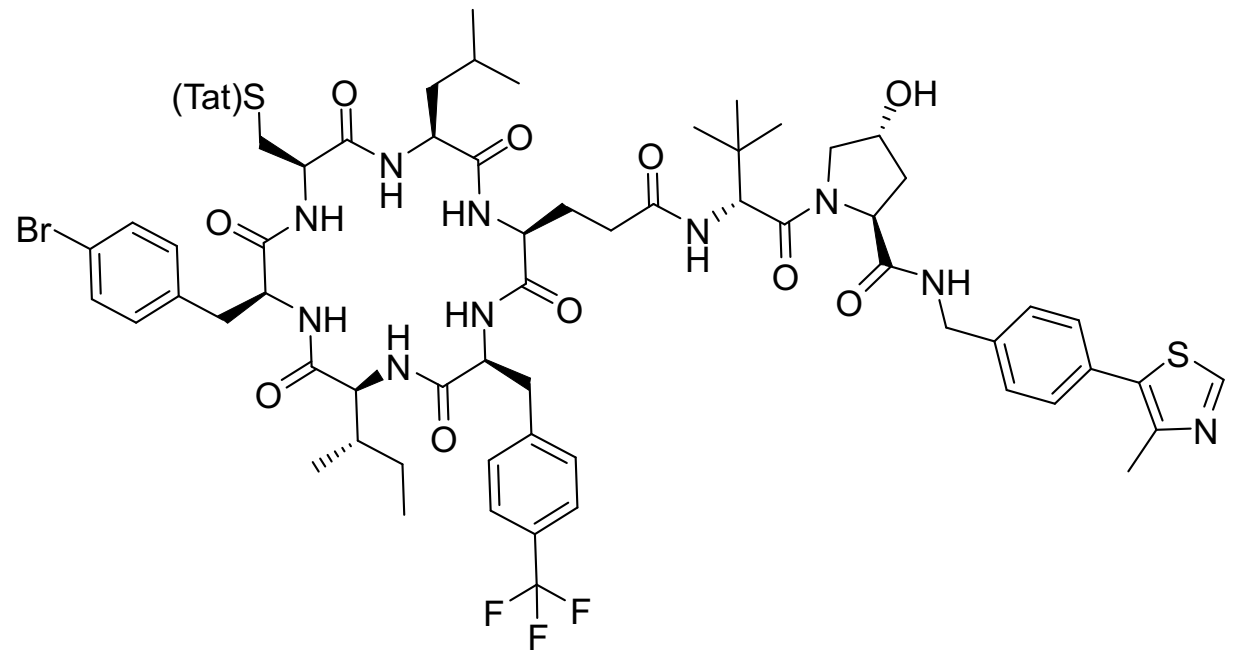

cyclo-C(Tat)(AA)(VHL)LF(CF_3)IF(Br) (**59**)

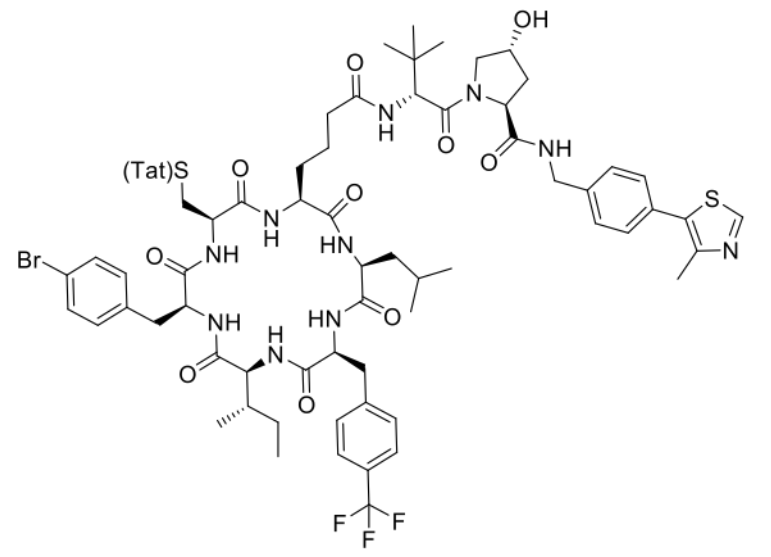

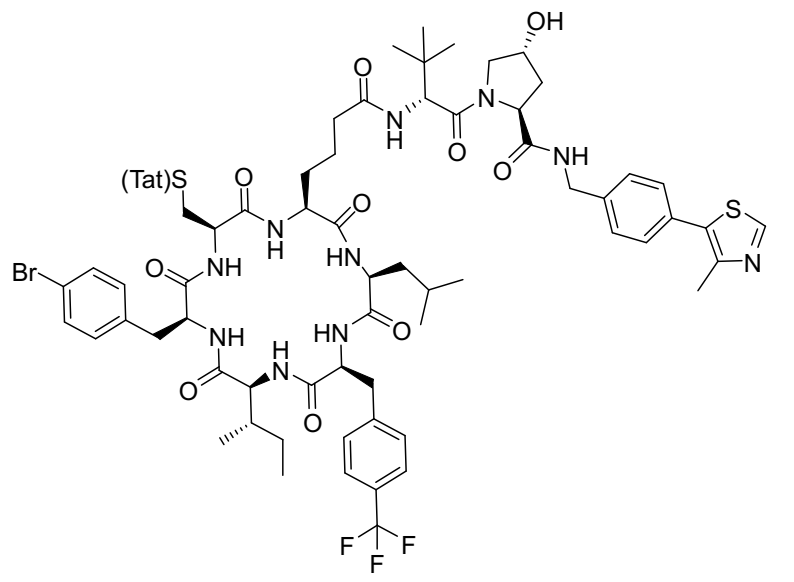

The Linear peptide L(AA)CF(Br)IF(CF3) was synthesised using 2-CTC SPPS a 0.1 mmol scale. The linear peptide was then cyclised and subjected to TFA deprotection. The crude was purified via reverse-phase flash chromatography to yield the StBu protected cyclic peptide C(StBu)(AA)LF(CF_3)IF(Br) (31 mg, 0.03 mmol, 30%). The pure cyclic peptide (31 mg, 0.03 mmol) was dissolved in DMF (2 mL) to which was added DIPEA (16 μL, 0.09 mmol, 3 eq) and HATU (18 mg, 0.045 mmol, 1.5 eq) and left to stir for 10 minutes. VH032 (15 mg, 0.041 mmol, 1.2 eq) was added to the mixture and the reaction was left to stir overnight at ambient temperature and purified by reverse phase HPLC chromatography to give an impure StBu protected cyclic peptide PROTAC as a white solid (25 mg, 0.018 mmol, 60%). The product was then StBu deprotected and purified via reverse-phase HPLC. Fractions were analysed via LC/MS, desired fractions were then pooled, concentrated *in vacuo*, and lyophilised to yield the cyclic peptide PROTAC C(AA)(VHL)LF(CF_3)IF(Br) as an off-yellow solid (8.2 mg, 0.0062 mmol, 36%). PROTAC C(AA)(VHL)LF(CF_3)IF(Br) (6 mg, 0.0045 mmol) was added to a solution of C(Spy)GRKKRRQRRRPPQ (13 mg, 0.0068 mmol, 1.5 eq) dissolved in DMF (2 mL) and stirred in an inert atmosphere overnight at ambient temperature. The mixture was then purified by reverse-phase HPLC. Fractions analysed via LC/MS, desired fractions were then pooled, concentrated *in vacuo*, and lyophilised to yield *Cyclo*- C(Tat)(AA)(VHL)LF(CF_3)IF(Br) as a white solid (10 mg, 0.0032 mmol, 71%).

LR MS (ESI$^+$): m/z $[M+H]^+$ cald for $C_{135}H_{214}BrF_3N_{46}O_{27}S_3$, 3142.5, found; 787.9 $[M+4H]^{4+}$, 630.3 $[M+5H]^{5+}$.
HRMS (ESI$^+$): shows expected model isotope pattern.
Analytical HPLC: Rt: (220 nm) 12.400 min (98% Purity).

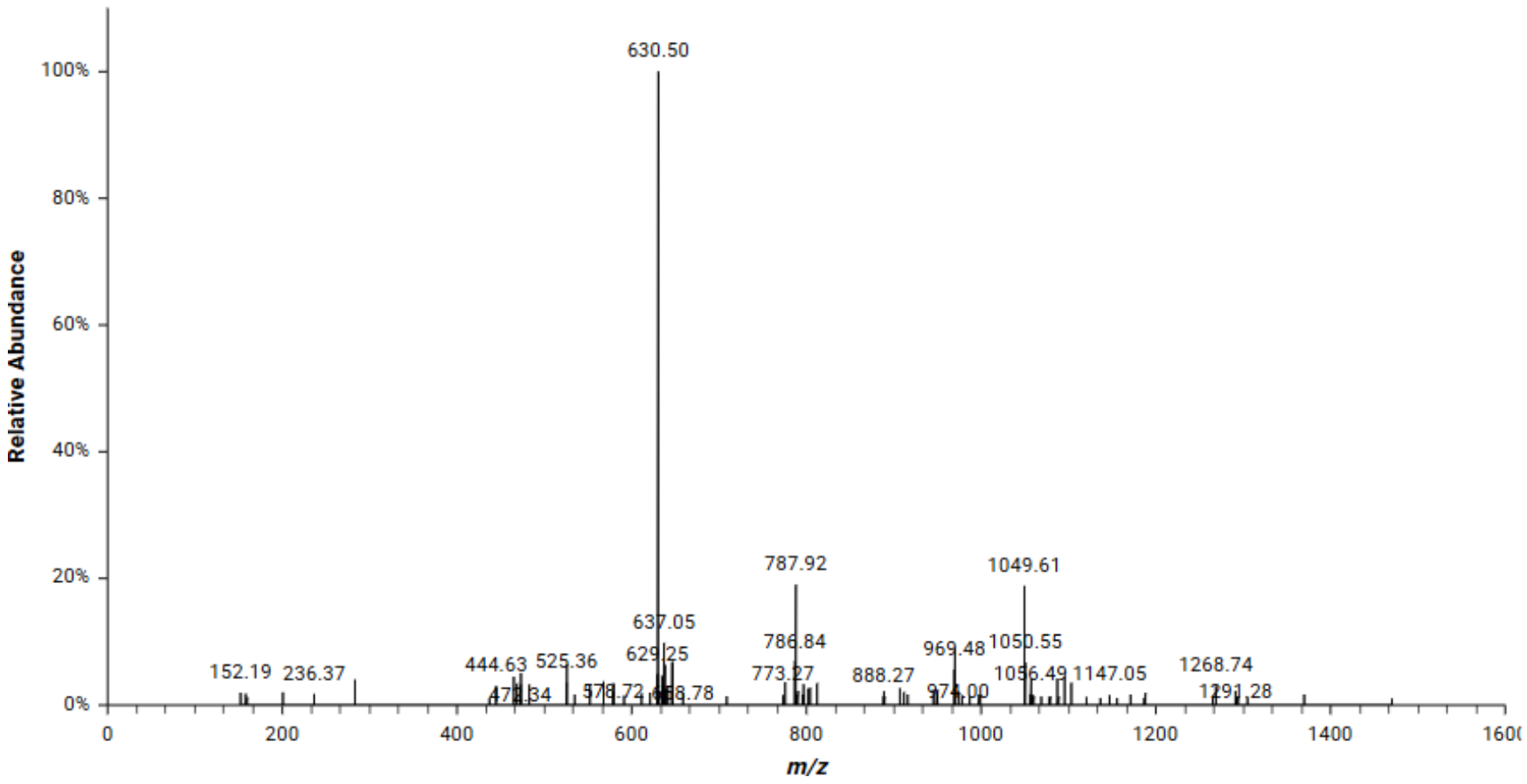
630.50
787.92
1049.61
637.05
629.25
786.84
969.48
1050.55
152.19
236.37
444.63
525.36
773.27
888.27
1056.49
1147.05
1268.74
1291.28
Relative Abundance
m/z

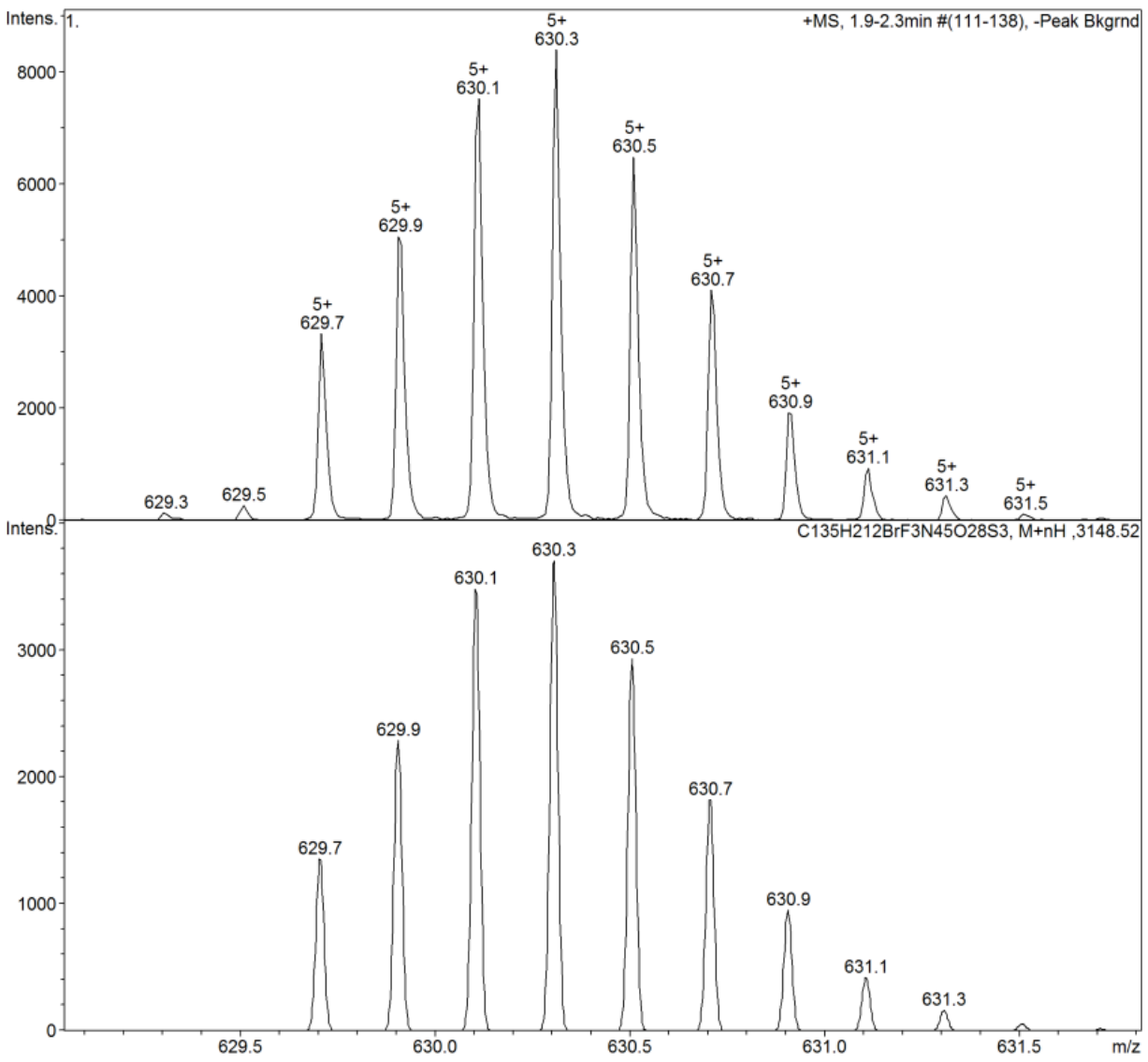
+MS, 1.9-2.3min #(111-138), -Peak Bkgrnd
C135H212BrF3N45O28S3, M+nH ,3148.52
Intens.
m/z

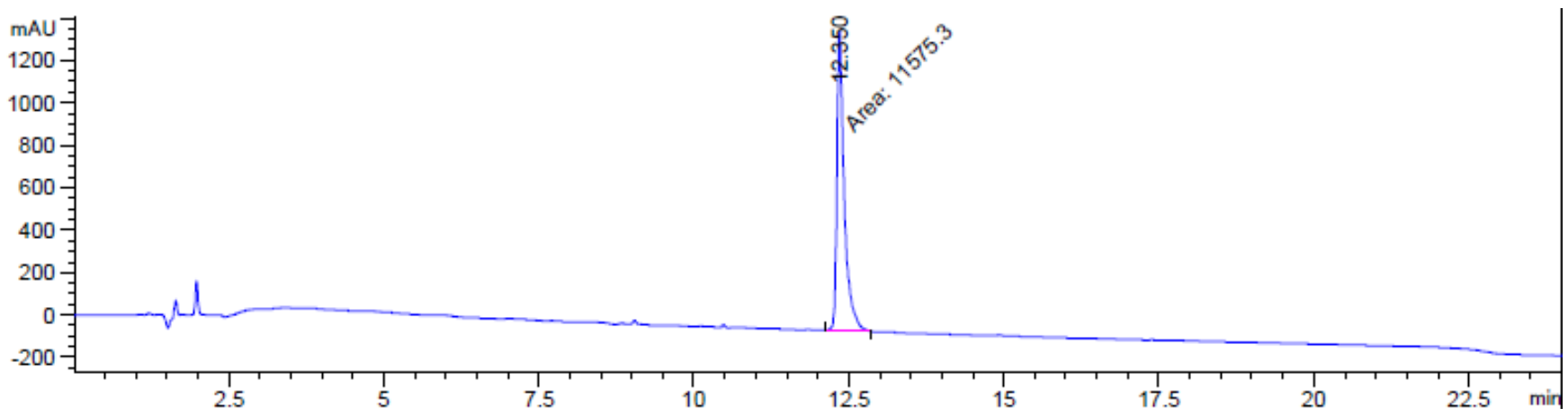
mAU
12.350
Area: 11575.3
min

cyclo-C(Tat)(P)(VHL)LF(CF_3)IF(Br) (**60**)

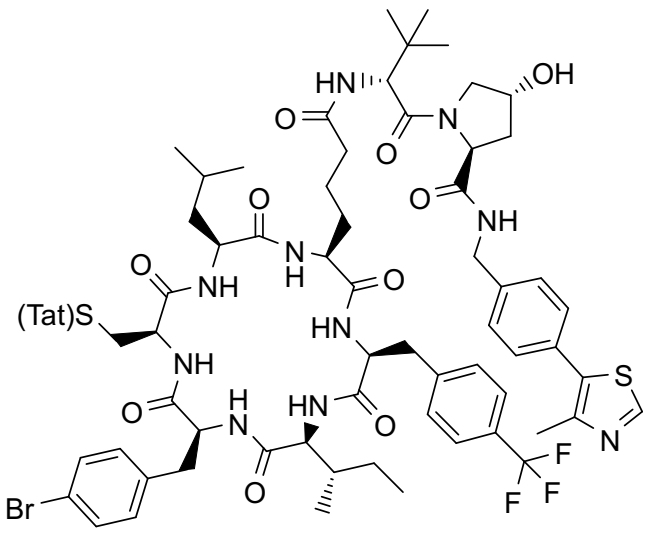

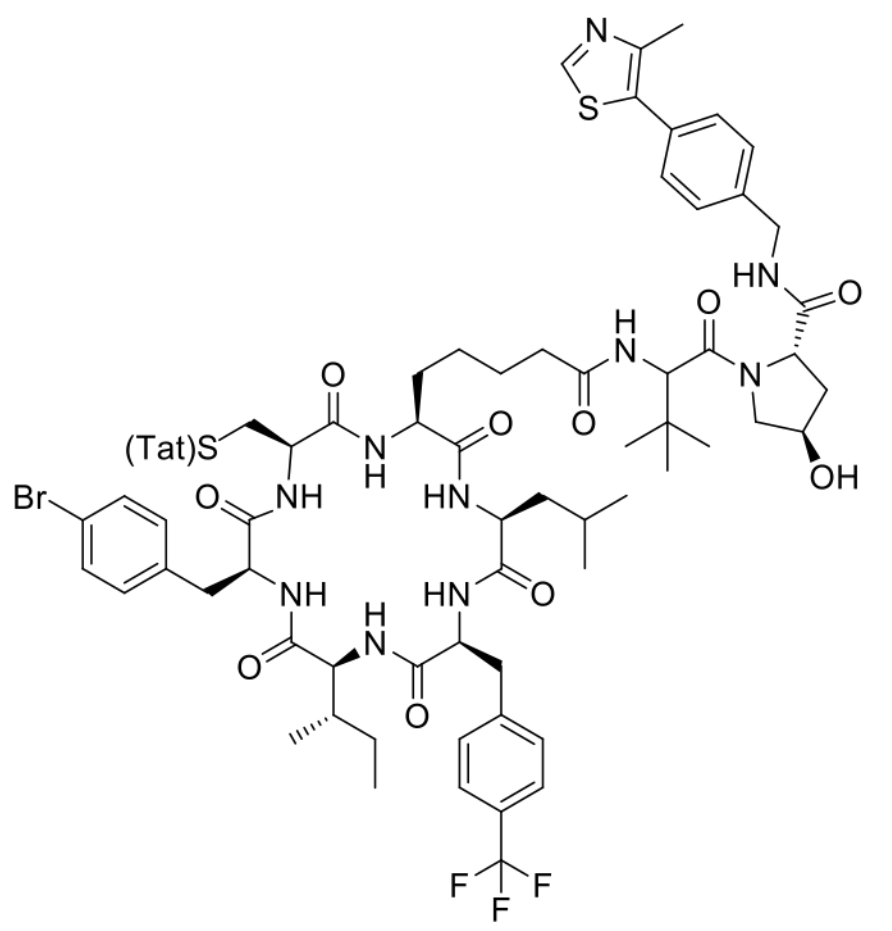

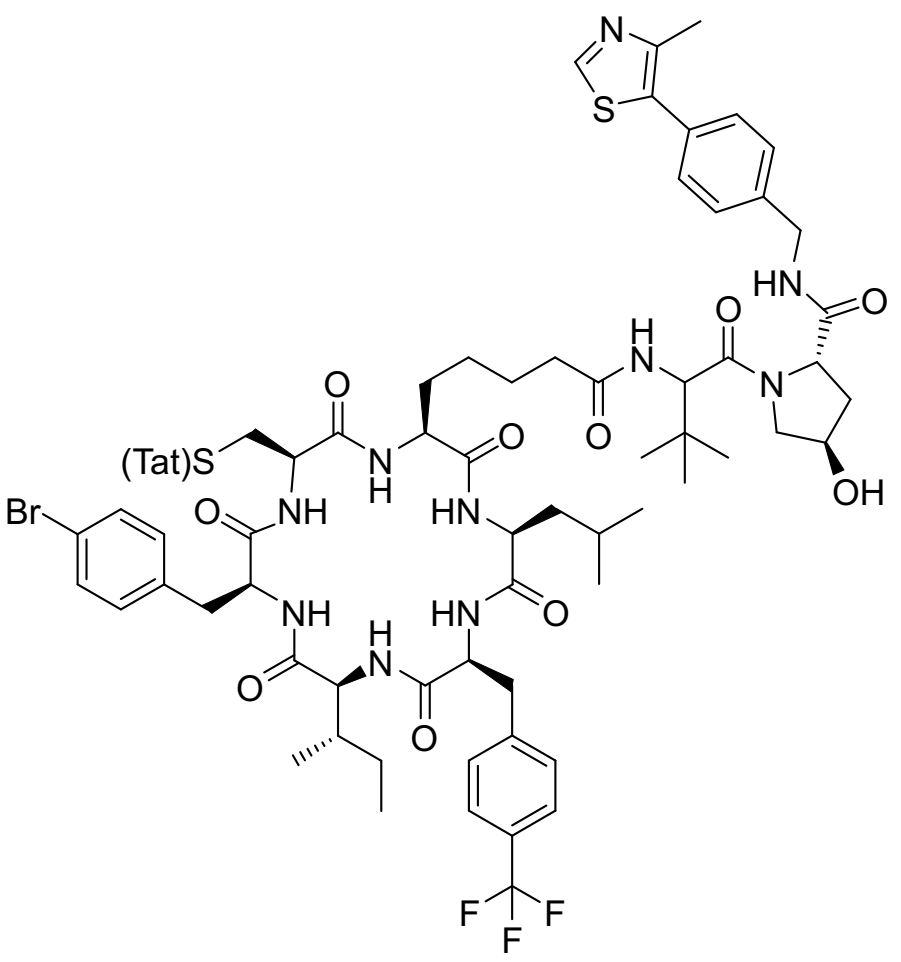

The linear peptide L(P)C(StBu)F(Br)IF(CF_3) (P= Pentanoic acid R group amino acid) was synthesised using 2-CTC resin SPPS at a 0.1 mmol scale. The linear peptide was then cyclised and subjected to TFA deprotection. The crude was subsequently purified via reverse-phase flash chromatography to yield C(StBu)(P)LF(CF_3)IF(Br) as a white solid (24 mg, 0.024 mmol, 24%). The cyclic peptide (24 mg, 0.024 mmol) was dissolved in DMF (1 mL) to which was added DIPEA (12 µl, 0.072 mmol, 3 eq), HATU (14 mg, 0.036 mmol, 1.5 eq) and VH032 ligand (11 mgs, 0.029 mmol, 1.2 eq). The reaction was then stirred at ambient temperature overnight and purified by reverse phase chromatography to give C(StBu)(P)(VHL)LF(CF_3)IF(Br) as a white solid (10.1 mg, 0.008 mmol, 31%). The StBu group was then deprotected and the crude purified via reverse-phase HPLC. Fractions were analysed via LC/MS, desired fractions were then pooled, concentrated *in vacuo,* and lyophilised to yield the cyclic peptide C(P)(VHL)LF(CF_3)IF(Br) as a white solid (5 mg, 0.0037 mmol, 47%). *cyclo-* C(P)(VHL)LF(CF_3)IF(Br) (4 mg, 0.003 mmol) was added to a solution of C(Spy)GRKKRRQRRRPPQ (11.6 mg, 0.006 mmol) dissolved in DMF (2 mL) and stirred in an inert atmosphere overnight at ambient temperature. The mixture was then purified by reverse-phase HPLC. Fractions analysed via LC/MS, desired fractions were then pooled, concentrated *in vacuo*, and lyophilised to yield *cyclo-* C(Tat)(P)(VHL)LF(CF_3)IF(Br) as a white solid (8 mg, 0.0025 mmol, 84%).

LR MS (ESI+): m/z $[M+H]^+$ cald for $C_{136}H_{216}BrF_3N_{46}O_{27}S_3$, 3157.5, found; 791.1 $[M+4H]^{4+}$, 633.1 $[M+5H]^{5+}$.
HRMS (ESI+): shows expected model isotope pattern.
Analytical HPLC: Rt: (220 nm) 12.684 min (100% Purity).

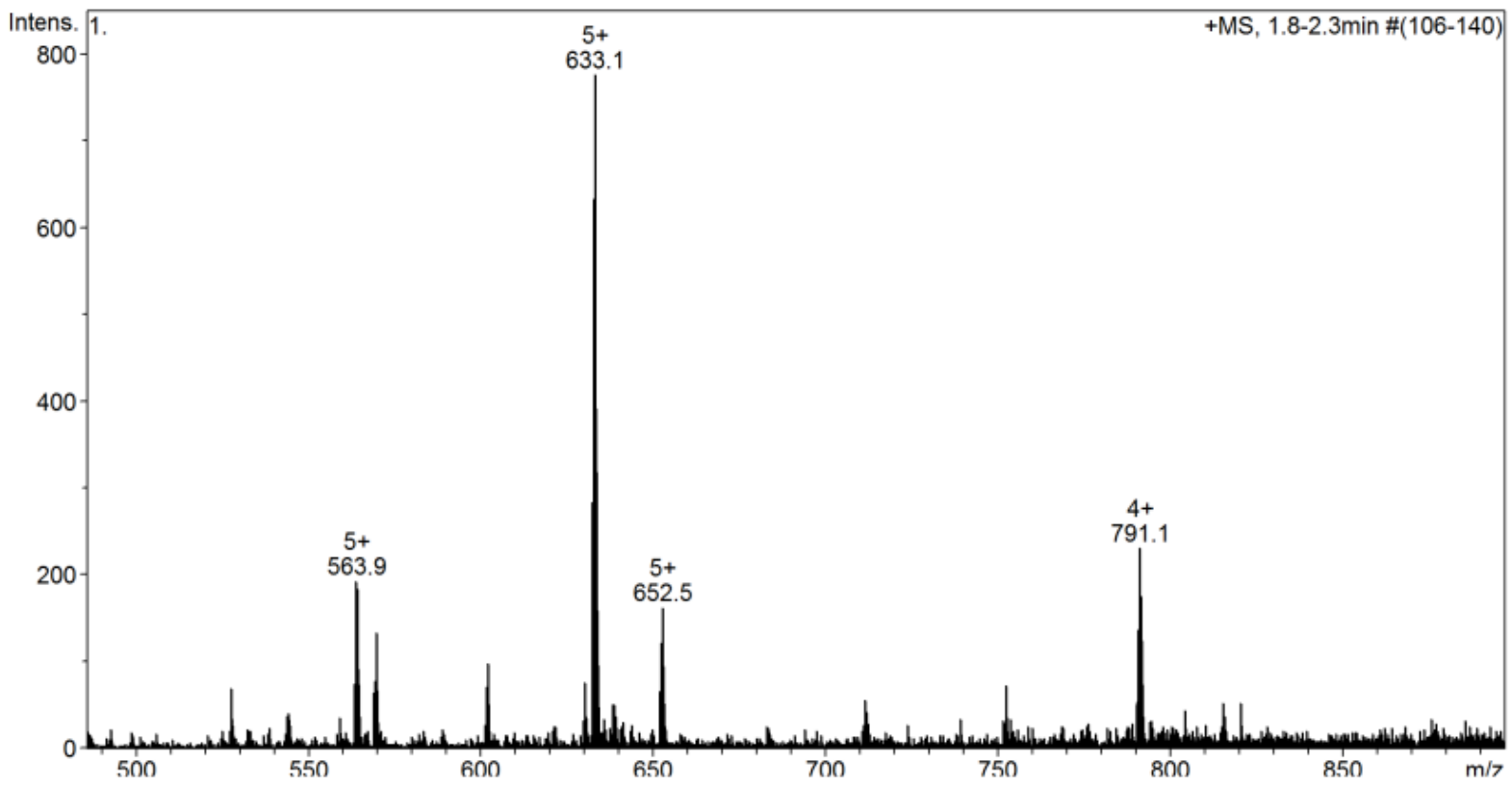

Intens.
+MS, 1.8-2.3min #(106-140)
5+
633.1
5+
563.9
5+
652.5
4+
791.1
m/z

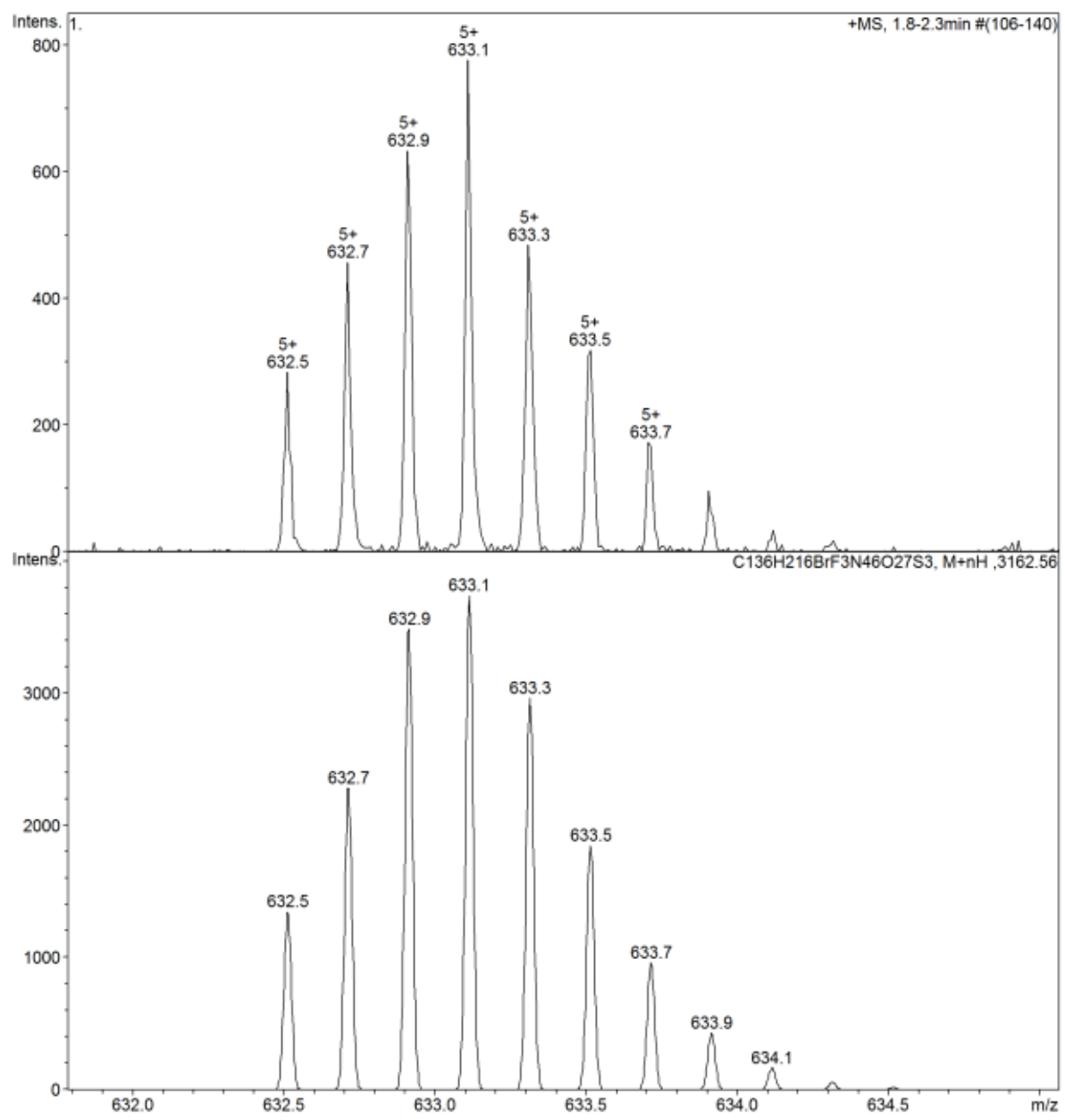

+MS, 1.8-2.3min #(106-140)
C136H216BrF3N46O27S3, M+nH ,3162.56
m/z

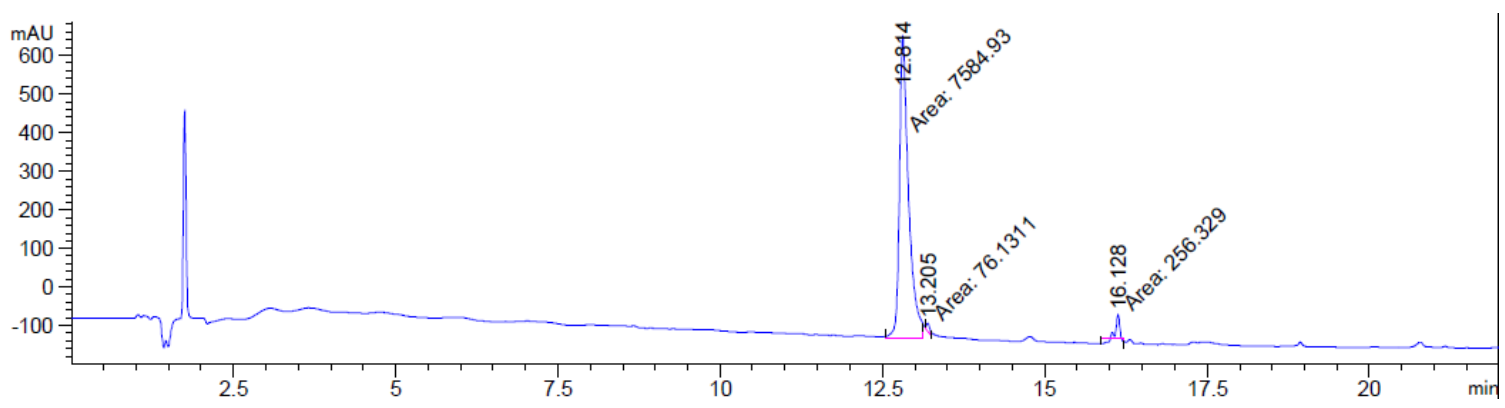

mAU
12.814
Area: 7584.93
13.205
Area: 76.1311
16.128
Area: 256.329
min

<u>*Cyclo*-C(Tat)(HX)(VHL)LF(CF_3)IF(Br) (**61**)</u>

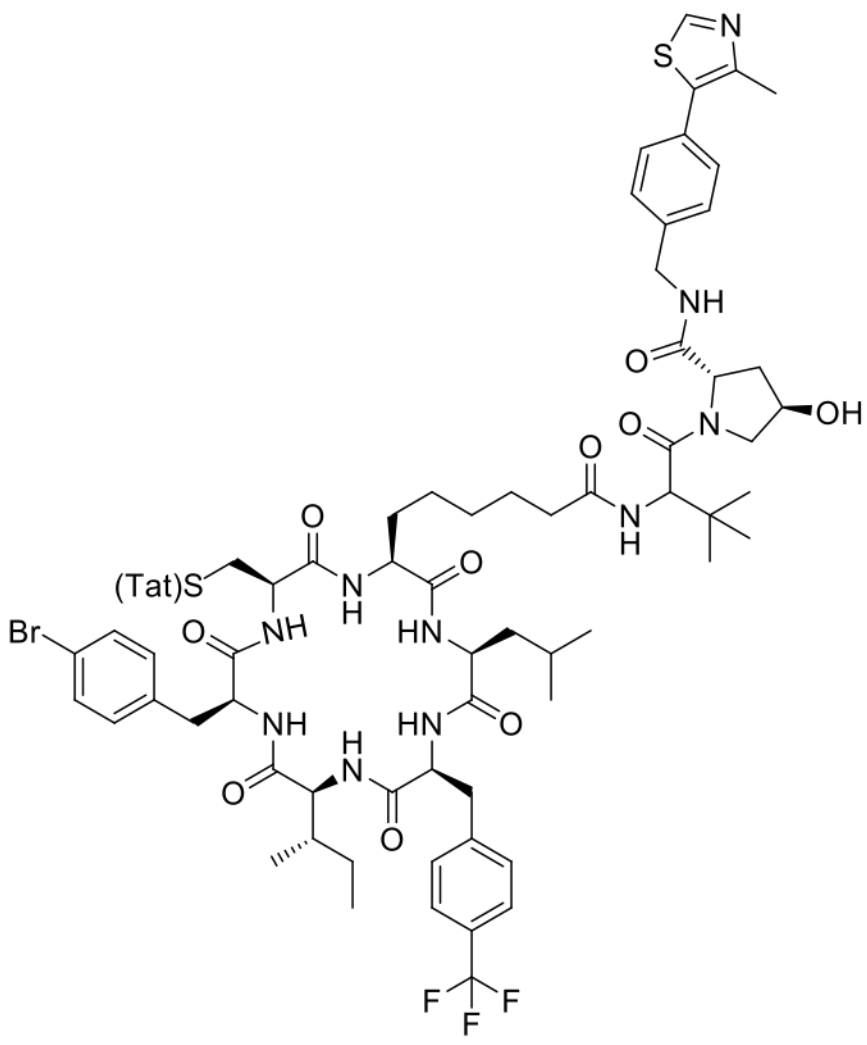

The linear peptide L(HX)C(StBu)F(Br)IF(CF_3) (HX= Hexanoic acid R group amino acid) was synthesised using 2-CTC resin SPPS at a 0.1 mmol scale. The linear peptide was then cyclised and subjected to TFA deprotection. The crude was subsequently purified via reverse-phase flash chromatography to yield C(StBu)(HX)LF(CF_3)IF(Br) as a white solid (21 mg, 0.02 mmol, 20%). The pure cyclic peptide (21 mg, 0.02 mmol) was dissolved in DMF (1 mL) to which was added DIPEA (10.2 µl, 0.6 mmol, 3 eq), HATU (11.4 mg, 0.03 mmol, 1.5 eq) and VH032 ligand (14 mgs, 0.025 mmol, 1.2 eq). The reaction was then stirred at ambient temperature overnight and purified by reverse phase chromatography to give C(StBu)(HX)(VHL)LF(CF_3)IF(Br) as a white solid (13.3 mg, 0.0092 mmol, 47%). The StBu group was then cleaved and the crude purified via reverse-phase HPLC. Fractions were analysed via LC/MS, desired fractions were then pooled, concentrated *in vacuo,* and lyophilised to yield the cyclic peptide C(HX)(VHL)LF(CF_3)IF(Br) as a white solid (6.7 mg, 0.0037 mmol, 40%). *cyclo*- C(HX)(VHL)LF(CF_3)IF(Br) (5 mg, 0.0037 mmol) was added to a solution of C(Spy)GRKKRRQRRRPPQ (14 mg, 0.0074 mmol, 2 eq) dissolved in DMF (2 mL) and stirred in an inert atmosphere overnight at ambient temperature. The mixture was then purified by reverse-phase HPLC. Fractions were analysed via LC/MS, desired fractions were then pooled, concentrated *in vacuo*, and lyophilised to yield *cyclo*-C(Tat)(HX)(VHL)LF(CF3)IF(Br) as a white solid (8 mg, 0.0025 mmol, 68%).

LR MS (ESI+): m/z $[M+H]^+$ cald for $C_{137}H_{218}BrF_3N_{46}O_{27}S_3$, 3171.54, found; 1059.2 $[M+3H]^{3+}$, 794.6 $[M+4H]^{4+}$, 635.9 $[M+5H]^{5+}$.
HRMS (ESI+): shows expected model isotope pattern.
Analytical HPLC: Rt: (220 nm) 12.811 min (100% Purity).

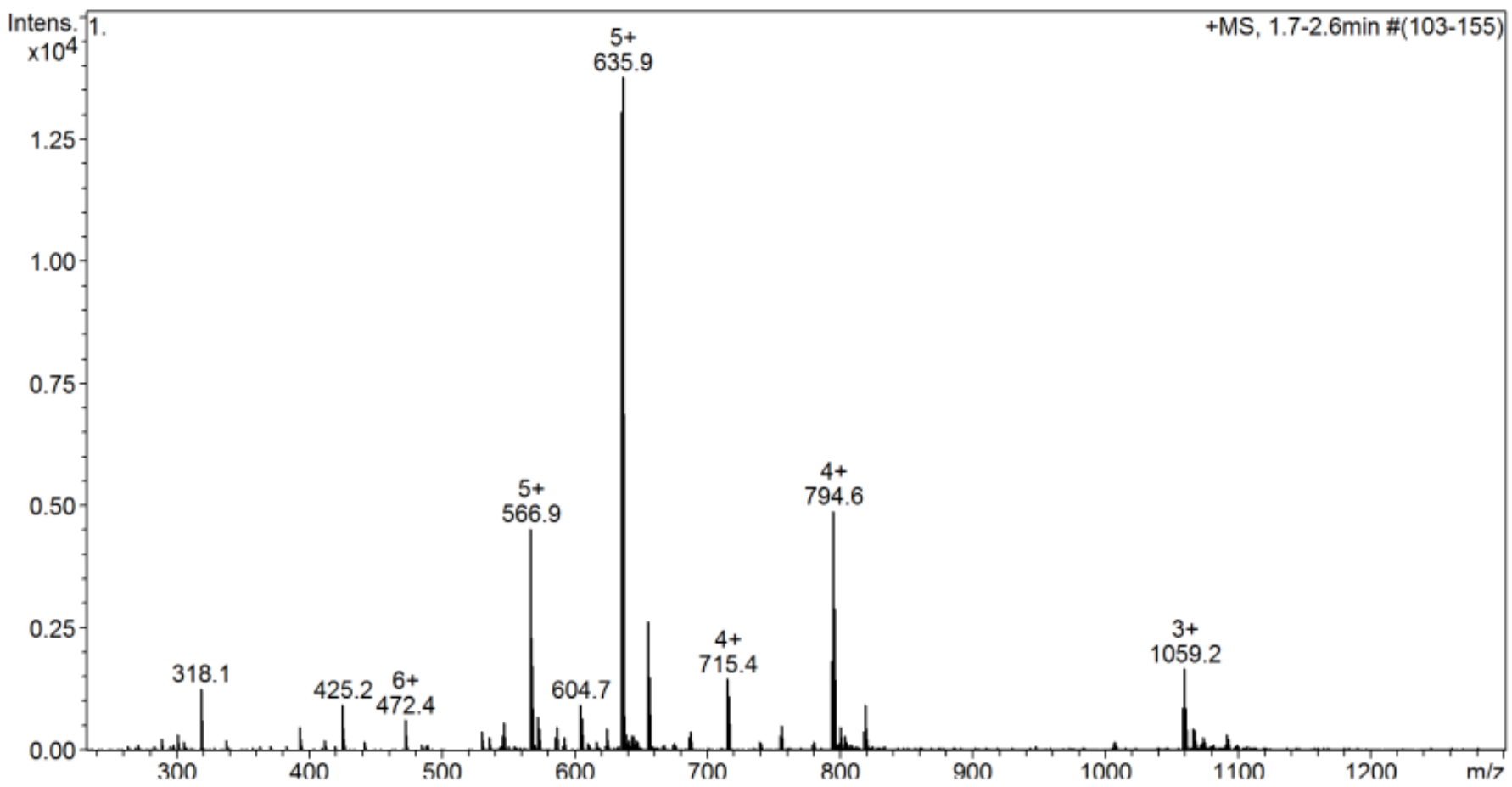
Intens.
x10^4
1.
+MS, 1.7-2.6min #(103-155)
5+
635.9
5+
566.9
4+
794.6
4+
715.4
3+
1059.2
318.1
425.2
6+
472.4
604.7
m/z

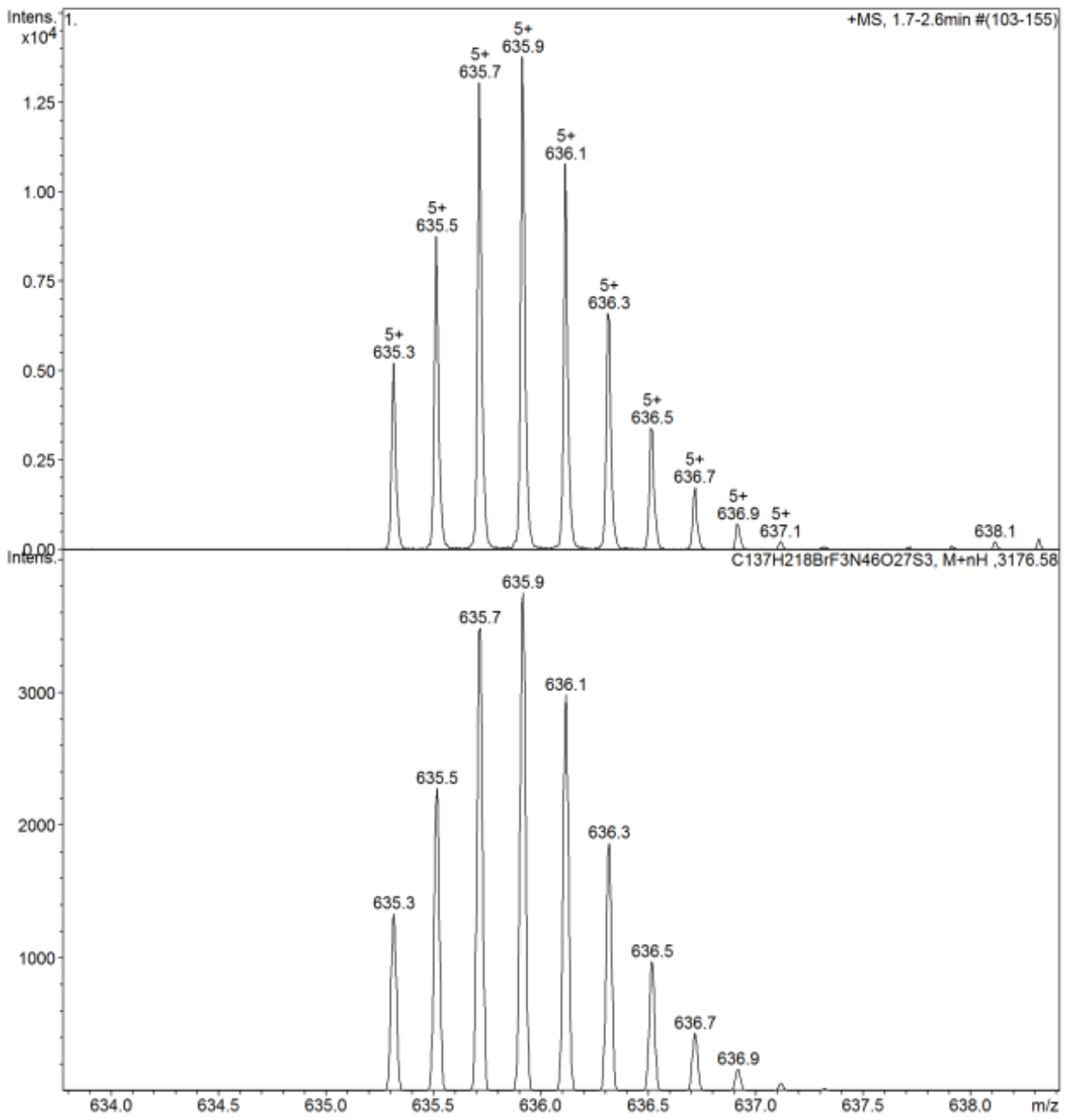
Intens.
x10^4
1.
+MS, 1.7-2.6min #(103-155)
C137H218BrF3N46O27S3, M+nH ,3176.58
m/z

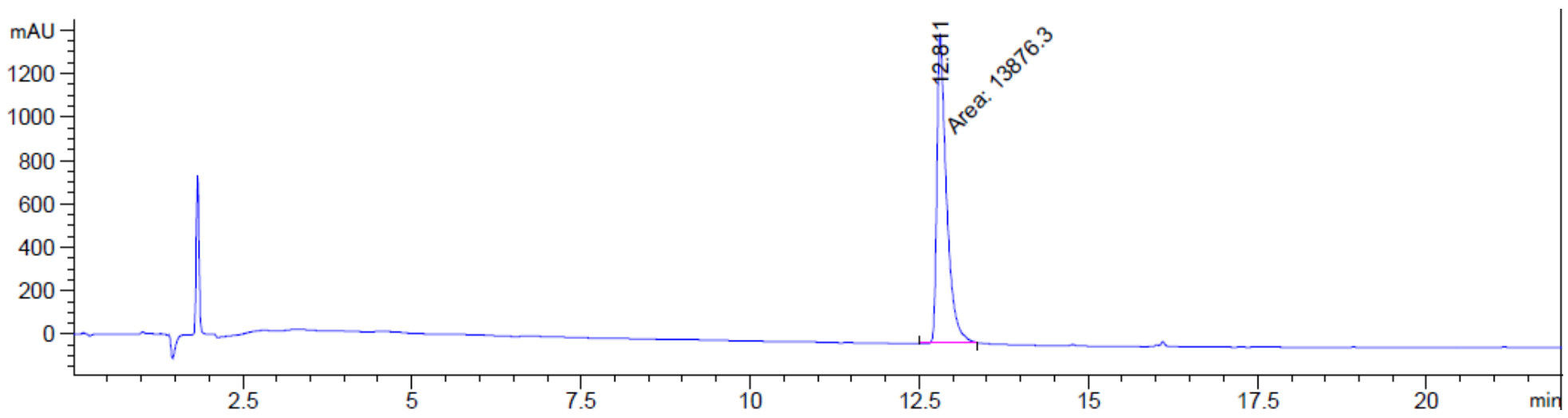
mAU
12.811
Area: 13876.3
min

<u>*cyclo*-C(Tat)(HP)(VHL)LF(CF_3)IF(Br) (**62**)</u>

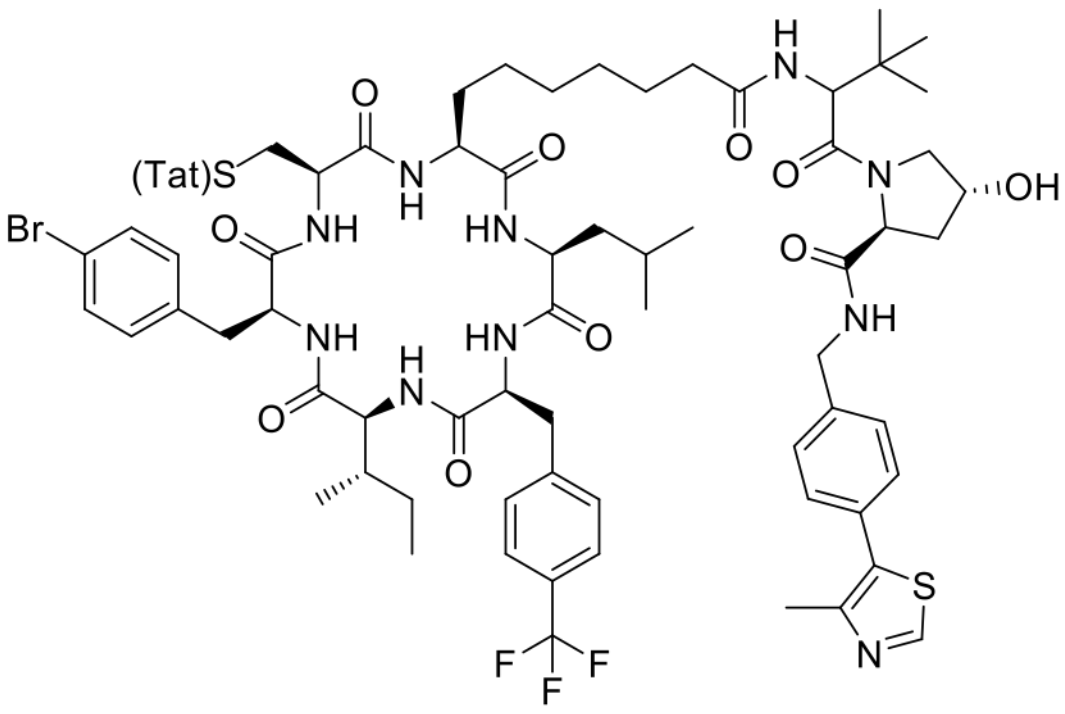

The linear peptide L(HP)C(StBu)F(Br)IF(CF_3) (HP = Heptanoic acid R group amino acid) was synthesised using 2-CTC resin SPPS at a 0.1 mmol scale. The linear peptide was then cyclised and subjected to TFA deprotection. The crude was subsequently purified via reverse-phase flash chromatography to yield *cyclo*-C(StBu)(HP)LF(CF_3)IF(Br) as a white solid (33 mg, 0.032 mmol, 32%). The pure cyclic peptide (33 mg, 0.032 mmol) was dissolved in DMF (1 mL) to which was added DIPEA (49 µL, 0.29 mmol, 3 eq), HATU (18 mg, 0.048 mmol, 1.5 eq) and VH032 ligand (14.7 mgs, 0.11 mmol, 1.1 eq). The reaction was then stirred at ambient temperature overnight and purified by reverse phase chromatography to give C(StBu)(HP)(VHL)LF(CF_3)IF(Br) as a white solid (27 mg, 0.019 mmol, 58%). The StBu group was then cleaved and the crude purified via reverse-phase HPLC. Fractions were analysed via LC/MS, desired fractions were then pooled, concentrated *in vacuo,* and lyophilised to yield the cyclic peptide C(HP)(VHL)LF(CF_3)IF(Br) as a white solid (9.7 mg, 0.0071 mmol, 37%). *cyclo*- C(HP)(VHL)LF(CF_3)IF(Br) (8.7 mg, 0.0064 mmol) was added to a solution of C(Spy)GRKKRRQRRRPPQ (24 mg, 0.013 mmol, 2 eq) dissolved in DMF (2 mL) and stirred in an inert atmosphere overnight at ambient temperature. The mixture was then purified by reverse-phase HPLC. Fractions analysed via LC/MS, desired fractions were then pooled, concentrated *in vacuo*, and lyophilised to yield *cyclo*- C(Tat)(HP)(VHL)LF(CF_3)IF(Br) as a white solid (16 mg, 0.005 mmol, 78%).

LR MS (ESI+): m/z $[M+H]^+$ cald for $C_{138}H_{220}BrF_3N_{46}O_{27}S_3$, 3185.56, found; 1063.9 $[M+3H]^{3+}$, 798.1 $[M+4H]^{4+}$, 638.7 $[M+5H]^{5+}$.
HRMS (ESI+): shows expected model isotope pattern.
Analytical HPLC: Rt: (220 nm) 12.975 min (91.5% Purity).

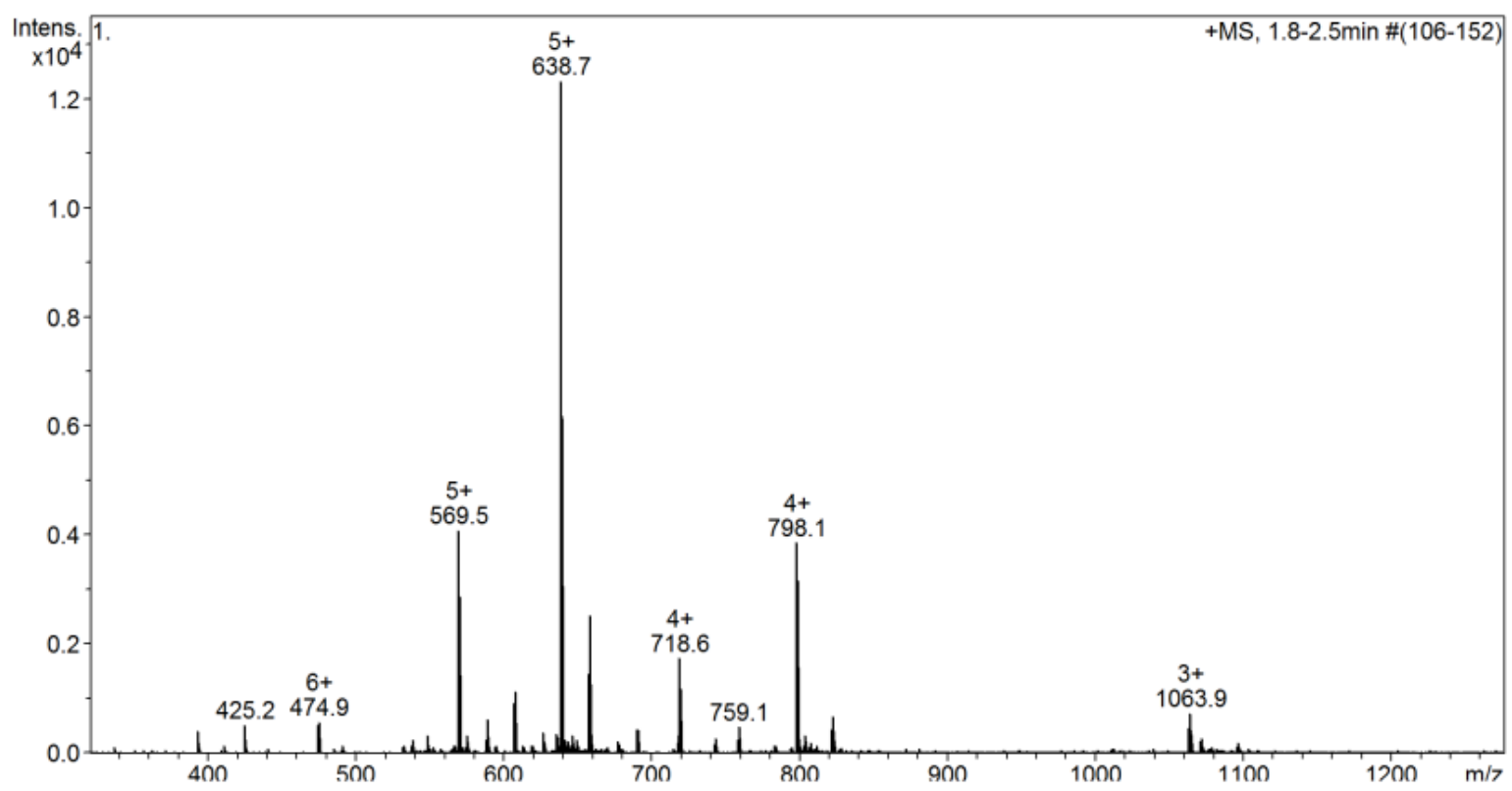

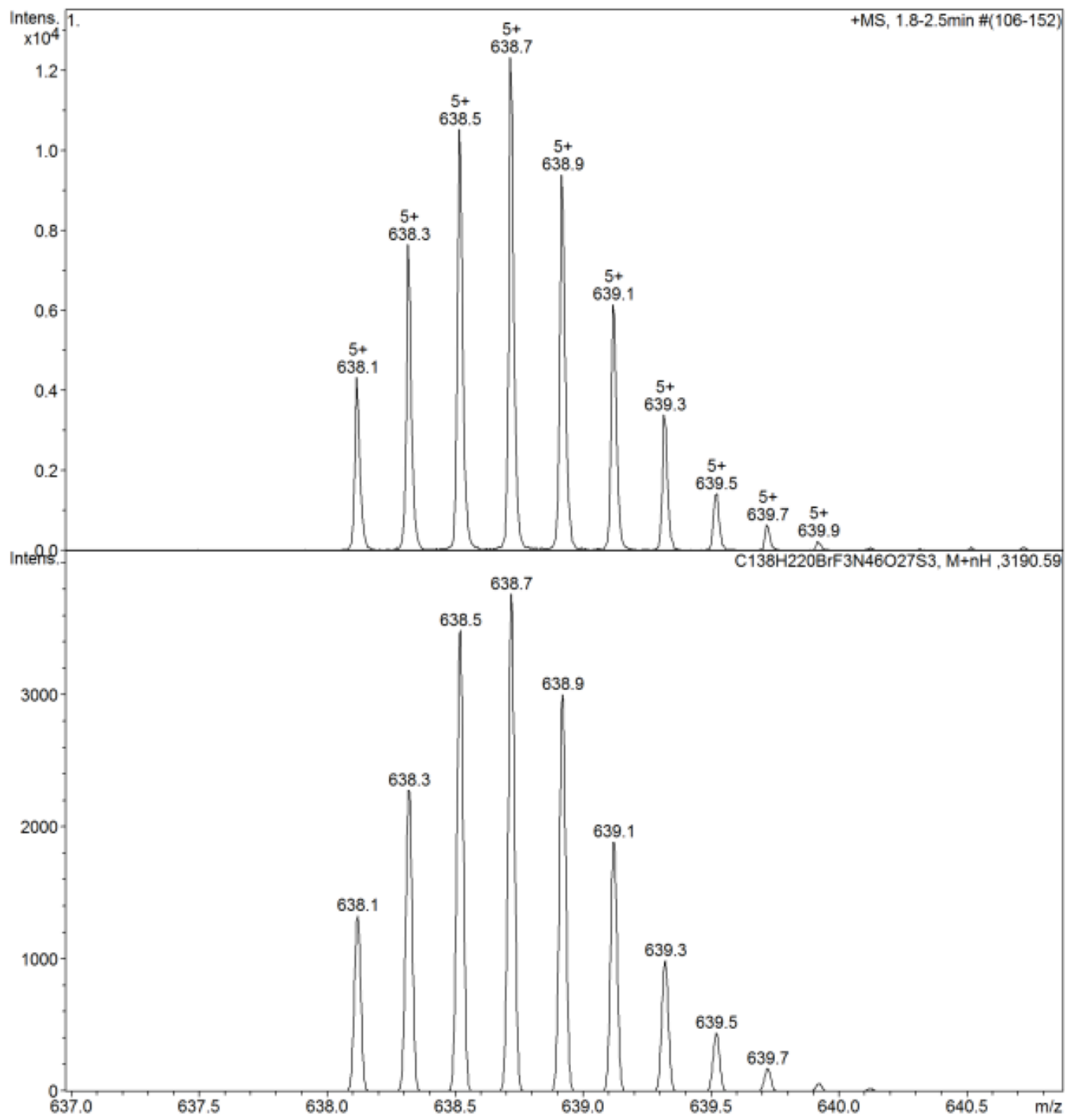
Intens.
x10^4
+MS, 1.8-2.5min #(106-152)
5+ 638.1
5+ 638.3
5+ 638.5
5+ 638.7
5+ 638.9
5+ 639.1
5+ 639.3
5+ 639.5
5+ 639.7
5+ 639.9
C138H220BrF3N46O27S3, M+nH ,3190.59
638.1
638.3
638.5
638.7
638.9
639.1
639.3
639.5
639.7
m/z

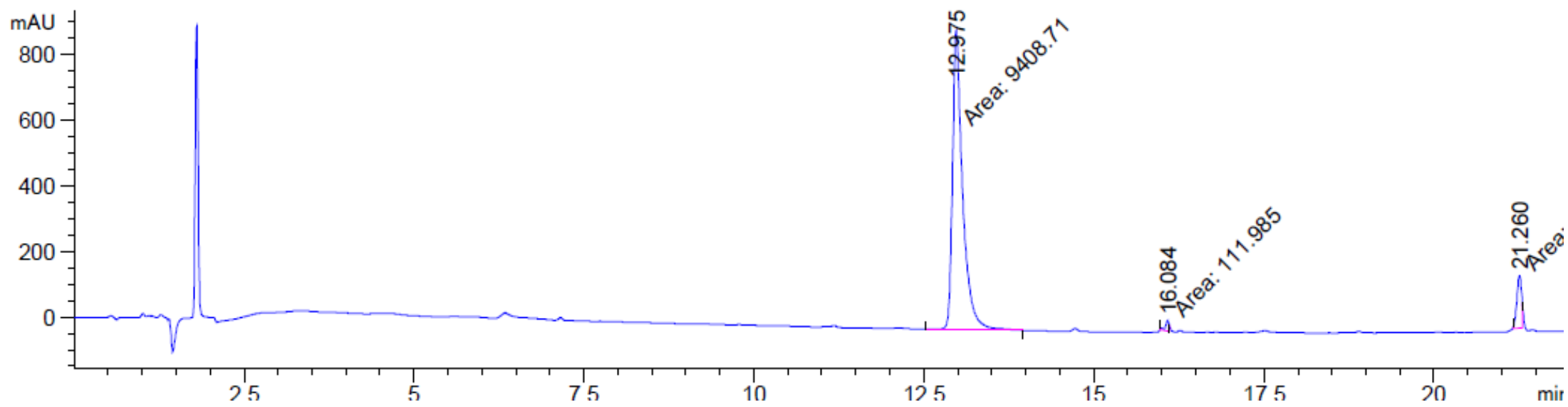
mAU
12.975
Area: 9408.71
16.084
Area: 111.985
21.260
min

<u>*cyclo*-C(Tat)LE(VHL)(*p*-CF_3)FI(*p*-Br)F (**63**)</u>

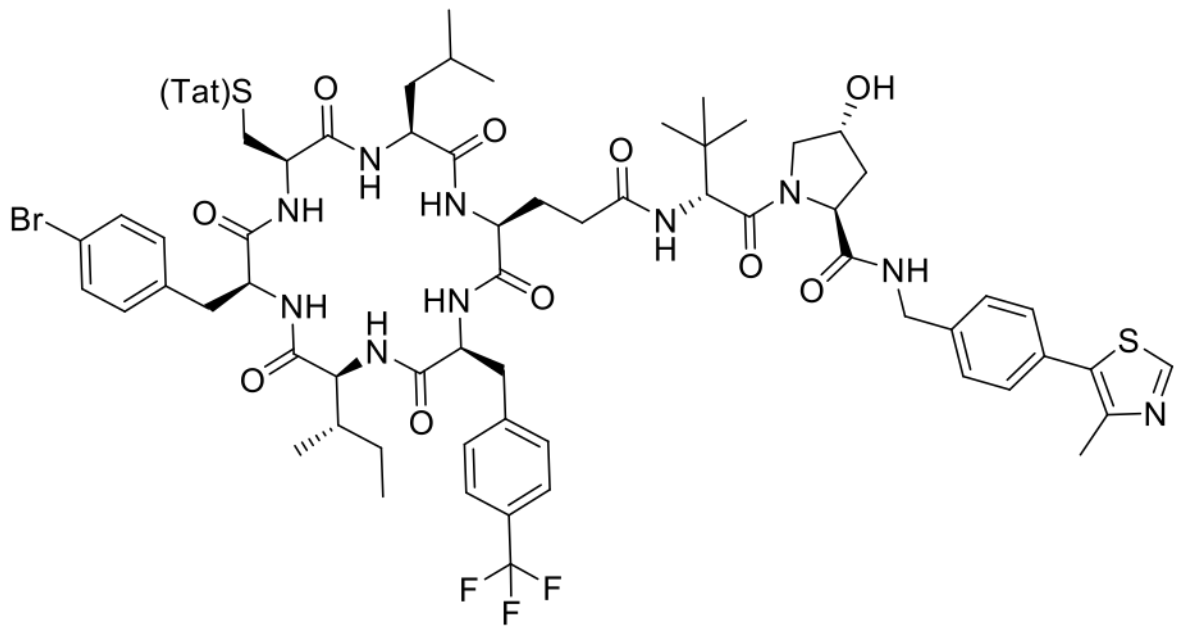

The Linear peptide LCF(Br)IF(CF_3)E was synthesised using 2-CTC SPPS at a 0.2 mmol scale. The linear peptide was then cyclised and subjected to TFA deprotection. The crude was purified via reverse-phase flash chromatography to yield the StBu protected cyclic peptide C(StBu)LEF(CF_3)IF(Br) (23 mg, 0.024 mmol, 24%). The pure cyclic peptide (23 mg, 0.024 mmol) was dissolved in DMF (2 mL) to which was added DIPEA (12 μL, 0.072 mmol, 3 eq) and HATU (14 mg, 0.036 mmol, 1.5 eq) and left to stir for 10 minutes. VH032 (12 mg, 0.029 mmol, 1.2 eq) was added to the mixture and the reaction was left to stir overnight at ambient temperature and purified by reverse phase HPLC chromatography to give an impure StBu protected cyclic peptide PROTAC as a white solid (18 mg, 0.0127 mmol, 53%). The product was then StBu deprotected and purified via reverse-phase HPLC. Fractions were analysed via LC/MS, desired fractions were then pooled, concentration *in vacuo*, and lyophilised to yield the cyclic peptide PROTAC CLE(VHL)F(CF_3)IF(Br) as an off-yellow solid (11 mg, 0.0084 mmol, 66%). PROTAC CLE(VHL)F(CF_3)IF(Br) (10 mg, 0.0076 mmol) was added to a solution of C(Spy)GRKKRRQRRRPPQ (21 mg, 0.011 mmol, 1.5 eq) dissolved in DMF (2 mL) and stirred in an inert atmosphere overnight at ambient temperature. The mixture was then purified by reverse-phase HPLC. Fractions were analysed via LC/MS, desired fractions were then pooled, concentrated *in vacuo*, and lyophilised to yield *Cyclo*- C(Tat)LE(VHL)F(CF_3)IF(Br) as a white solid (7.6 mg, 0.024, 32%).

LR MS (ESI$^+$): m/z $[M+H]^+$ cald for $C_{134}H_{212}BrF_3N_{46}O_{27}S_3$, 3128.5, found; 784.4 $[M+4H]^{4+}$, 627.5 $[M+5H]^{5+}$.
HRMS (ESI$^+$): shows expected model isotope pattern.
Analytical HPLC: Rt: (220 nm) 12.121 min (97% Purity).

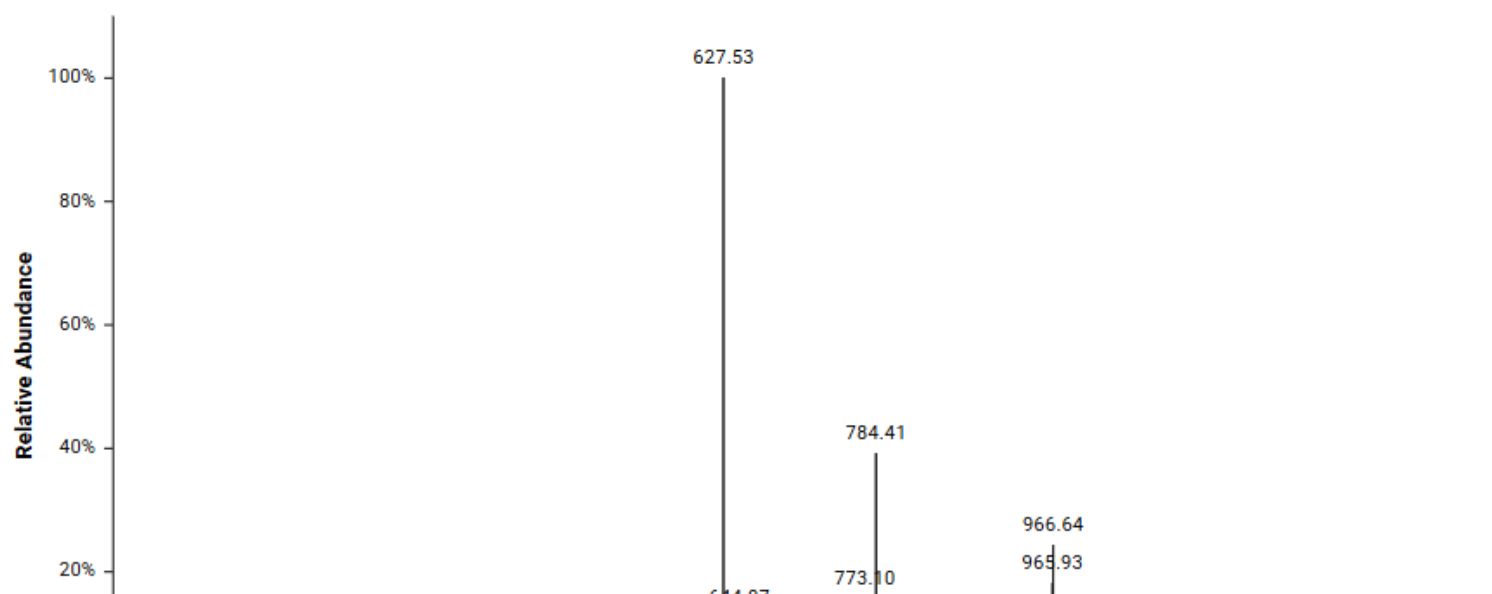

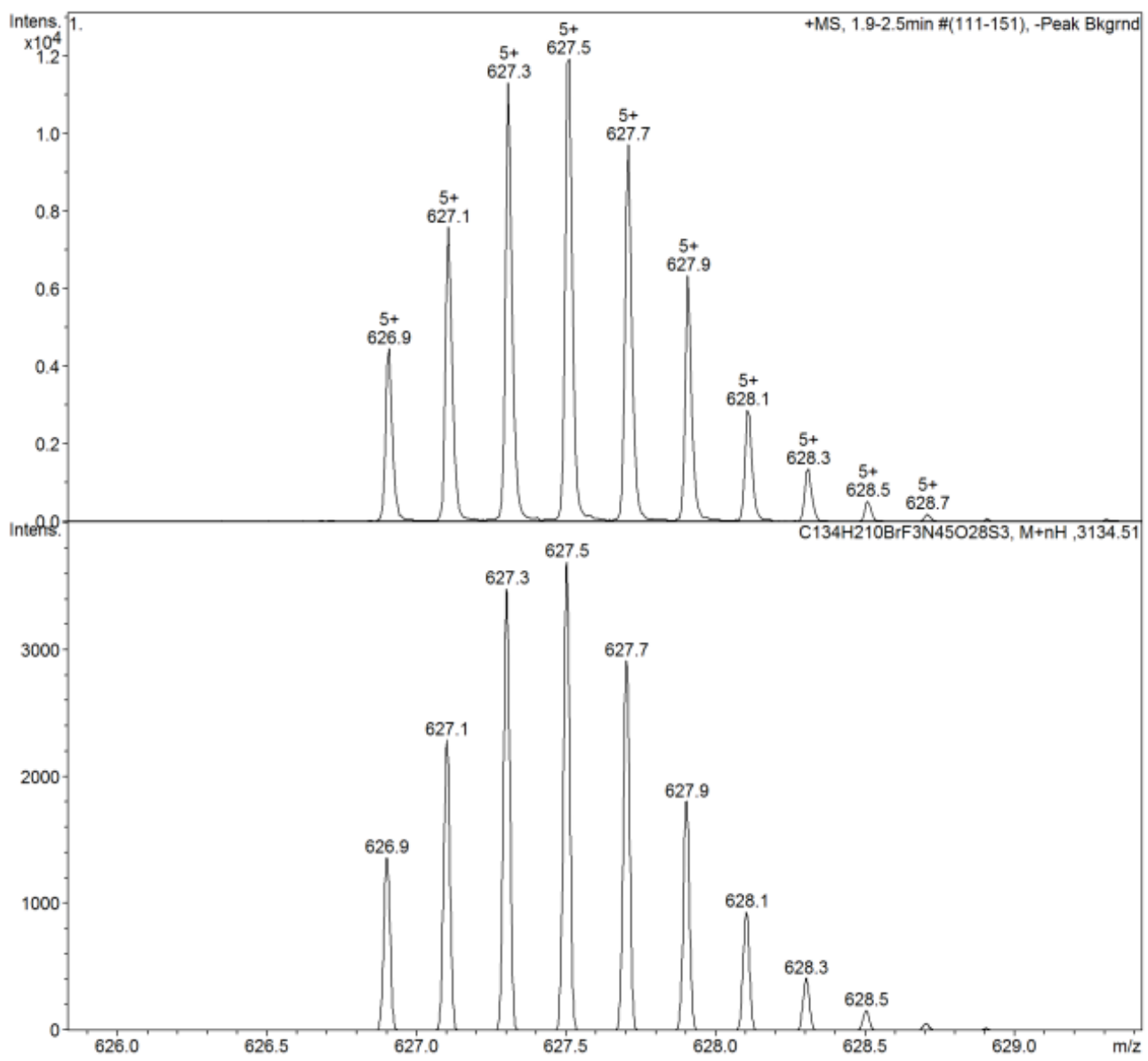
+MS, 1.9-2.5min #(111-151), -Peak Bkgrnd
C134H210BrF3N45O28S3, M+nH ,3134.51
m/z

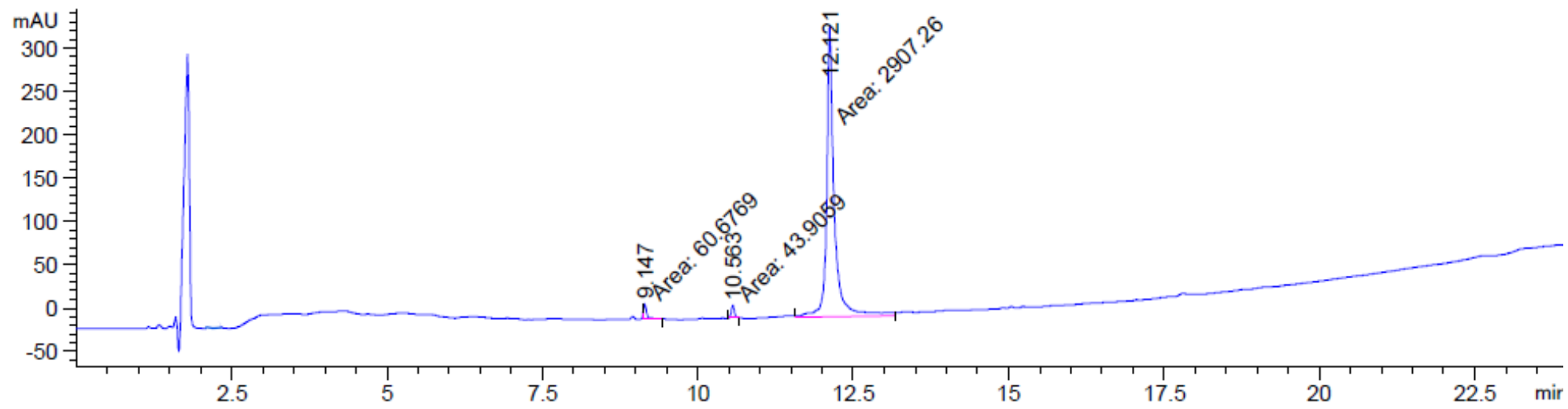
mAU
9.147
Area: 60.6769
10.563
Area: 43.9059
12.121
Area: 2907.26
min

cyclo-C(Tat)L(AA)(VHL)F(CF_3)IF(Br) (**64**)

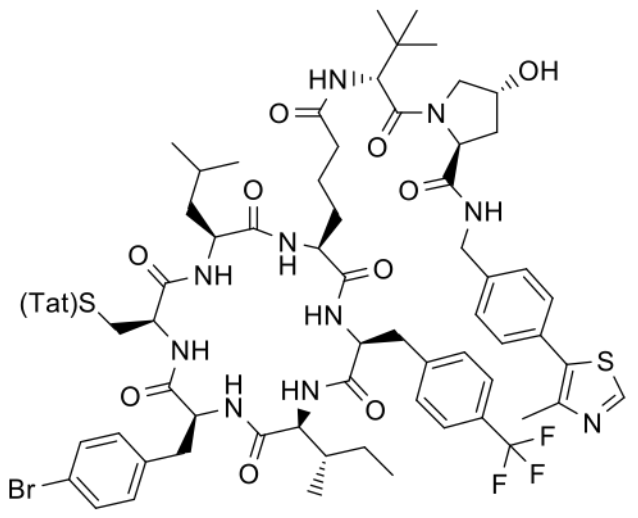

The Linear peptide LCF(Br)IF(CF3)(AA) (AA = aminoadipic acid) was synthesised using 2-CTC SPPS at a 0.1 mmol scale. The linear peptide was then cyclised and subjected to TFA deprotection. The crude was purified via reverse-phase flash chromatography to yield the StBu protected cyclic peptide C(StBu)L(AA)F(CF_3)IF(Br) (33 mg, 0.03 mmol, 30%). The pure cyclic peptide (33 mg, 0.034 mmol) was dissolved in DMF (2 mL) to which was added DIPEA (18 μL, 0.01 mmol, 3 eq) and HATU (19 mg, 0.05 mmol, 1.5 eq) and left to stir for 10 minutes. VH032 (17 mg, 0.041 mmol, 1.2 eq) was added to the mixture and the reaction was left to stir overnight at ambient temperature and purified by reverse phase HPLC chromatography to give an impure StBu protected cyclic peptide PROTAC as a white solid (24 mg, 0.017 mmol, 50%). The product was then StBu deprotected was deprotected and purified via reverse-phase HPLC. Fractions were analysed via LC/MS, desired fractions were then pooled, concentrated *in vacuo*, and lyophilised to yield the cyclic peptide PROTAC CL(AA)(VHL)F(CF_3)IF(Br) as an off-yellow solid (13.8 mg, 0.01 mmol, 61%). PROTAC CL(AA)(VHL)F(CF_3)IF(Br) (9 mg, 0.007 mmol) was added to a solution of C(Spy)GRKKRRQRRRPPQ (20 mg, 0.011 mmol, 1.5 eq) dissolved in DMF (2 mL) and stirred in an inert atmosphere overnight at ambient temperature. The mixture was then purified by reverse-phase HPLC. Fractions analysed via LC/MS, desired fractions were then pooled, concentrated *in vacuo*, and lyophilised to yield *Cyclo*- C(Tat)L(AA)(VHL)F(CF_3)IF(Br) as a white solid (15 mg, 0.0048 mmol, 69%).

LR MS (ESI$^+$): m/z $[M+H]^+$ cald for $C_{135}H_{214}BrF_3N_{46}O_{27}S_3$, 3143.5, found; 787.5 $[M+4H]^{4+}$, 630.3 $[M+5H]^{5+}$.
HRMS (ESI$^+$): shows expected model isotope pattern.
Analytical HPLC: Rt: (220 nm) 12.400 min (98% Purity).

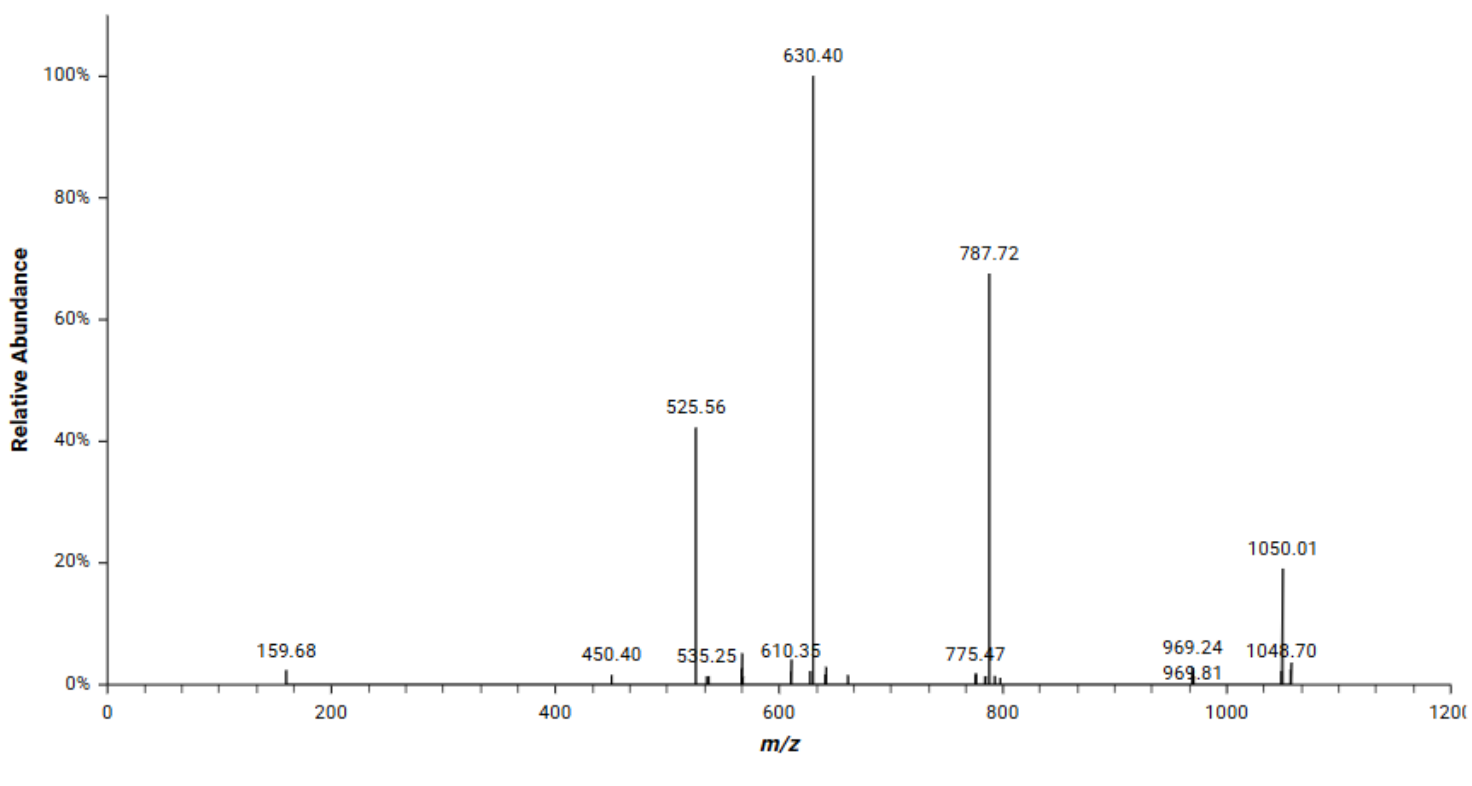

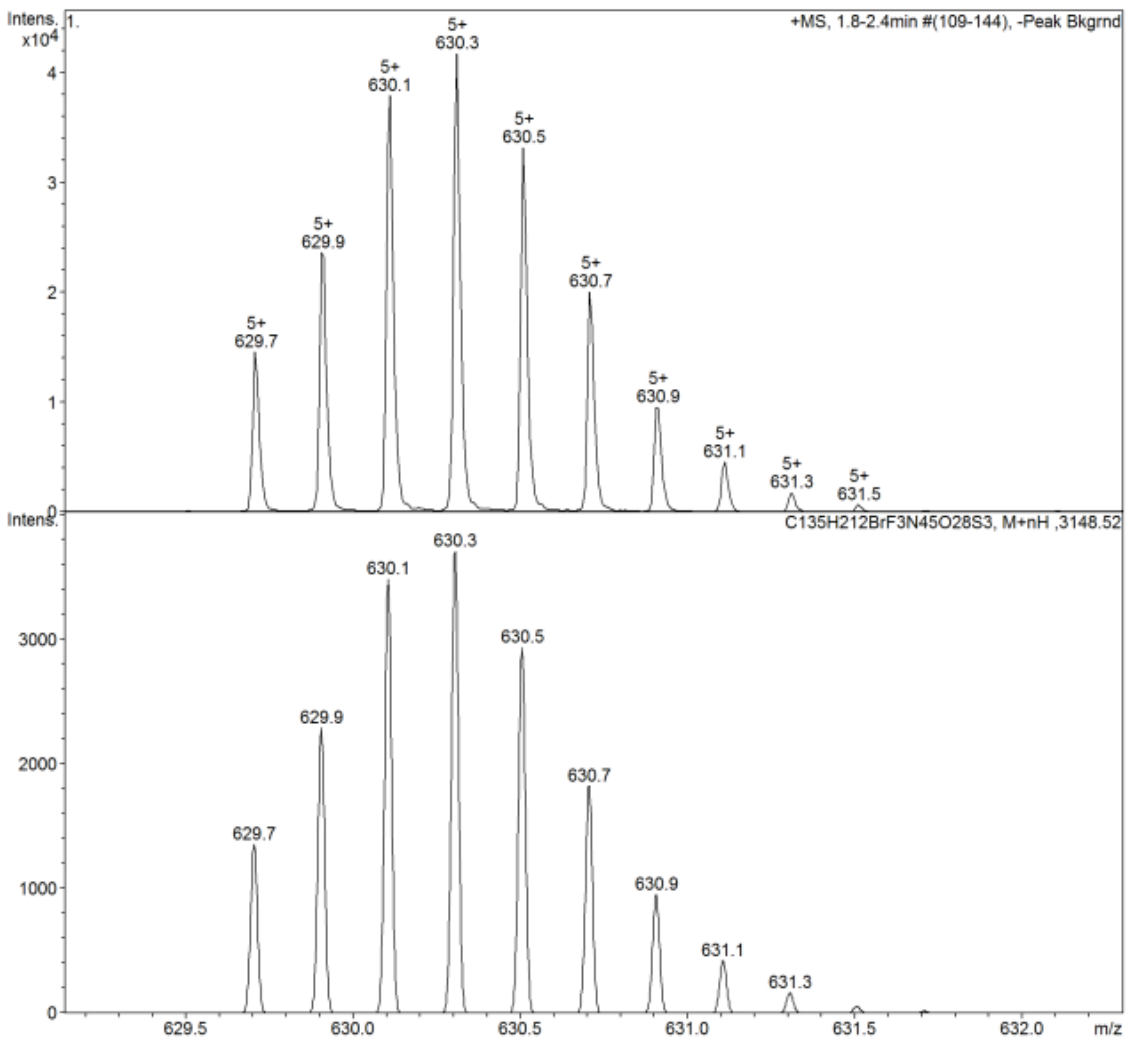

+MS, 1.8-2.4min #(109-144), -Peak Bkgrnd
C135H212BrF3N45O28S3, M+nH ,3148.52

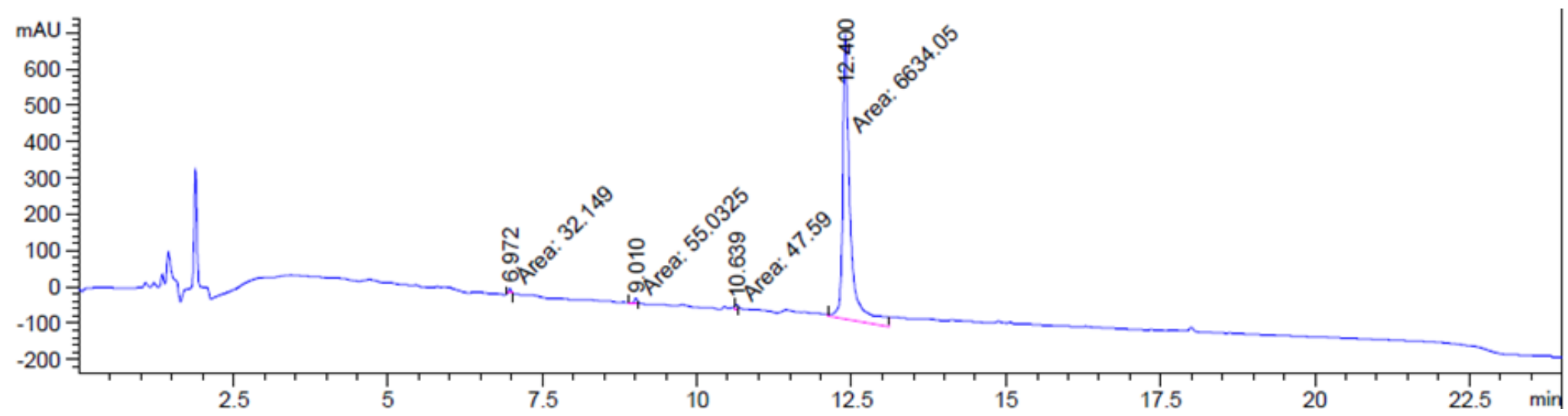

6.972
Area: 32.149
9.010
Area: 55.0325
10.639
Area: 47.59
12.400
Area: 6634.05

cyclo-C(Tat)L(P)(VHL)F(CF_3)IF(Br) (**65**)

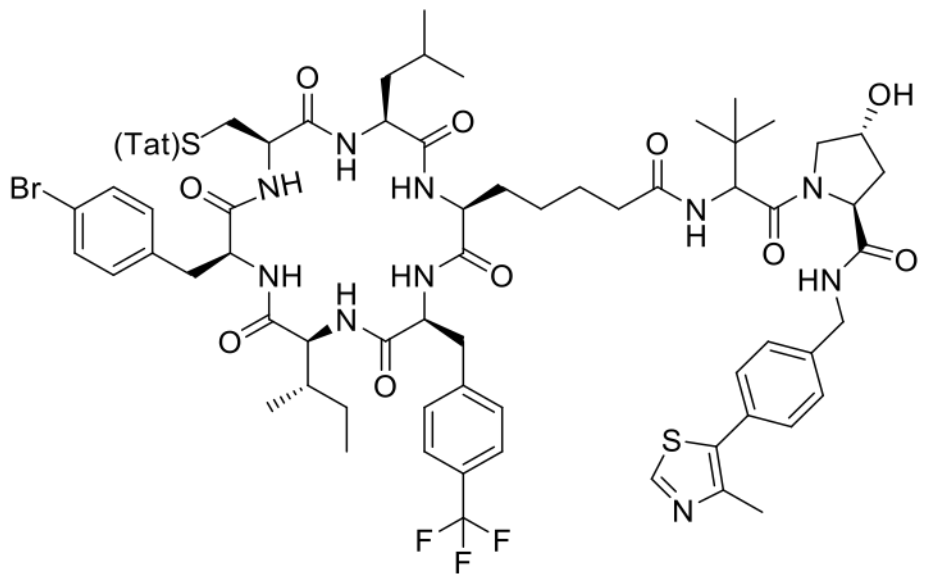

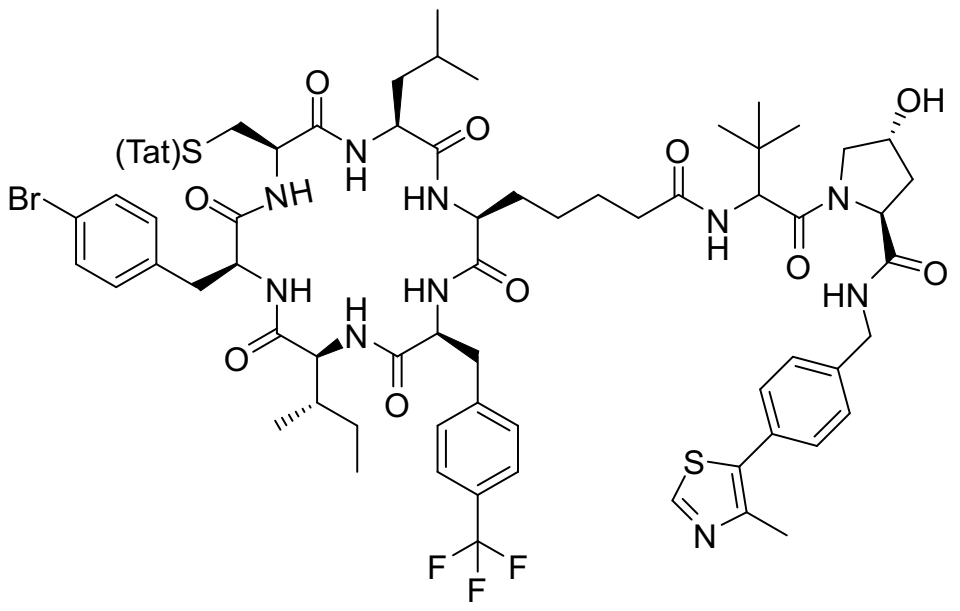

The linear peptide LC(StBu)F(Br)IF(CF_3)(P) (P= Pentanoic acid R group amino acid) was synthesised using 2-CTC resin SPPS at a 0.1 mmol scale. The linear peptide was then cyclised and subjected to TFA deprotection. The crude mixture was subsequently purified via reverse-phase flash chromatography to yield C(StBu)L(P)F(CF_3)IF(Br) as a white solid (22 mg, 0.022 mmol, 22%). The pure cyclic peptide (22 mg, 0.022 mmol) was dissolved in DMF (1 mL) to which was added DIPEA (11 µl, 0.66 mmol, 3 eq), HATU (11.4 mg, 0.033 mmol, 1.5 eq) and VH032 ligand (11 mgs, 0.026 mmol, 1.2 eq). The reaction was then stirred at ambient temperature overnight and purified by reverse phase chromatography to give the StBu protected cyclic peptide as a white solid (17.7 mg, 0.012 mmol, 54%). The StBu group was then cleaved as described in 5.1.5 and the crude purified via reverse-phase HPLC. Fractions were analysed via LC/MS, desired fractions were then pooled, concentrated *in vacuo,* and lyophilised to yield the cyclic peptide C(P)(VHL)LF(CF_3)IF(Br) as a white solid (12 mg, 0.009 mmol, 75%). *cyclo*- CL(P)(VHL)F(CF_3)IF(Br) (11 mg, 0.008 mmol) was added to a solution of C(Spy)GRKKRRQRRRPPQ (34 mg, 0.018 mmol) dissolved in DMF (2 mL) and stirred in an inert atmosphere overnight at ambient temperature. The mixture was then purified by reverse-phase HPLC. Fractions were analysed via LC/MS, desired fractions were then pooled, concentrated *in vacuo*, and lyophilised to yield *cyclo*- C(Tat)L(P)(VHL)F(CF_3)IF(Br) as a white solid (14 mg, 0.0044 mmol, 55%).

LR MS (ESI+): m/z $[M+H]^+$ cald for $C_{136}H_{216}BrF_3N_{46}O_{27}S_3$, 3157.5, found; 791.3 [M+4H]4+, 633.0 $[M+5H]^{5+}$, 528.5 $[M+6H]^{6+}$.
Analytical HPLC: Rt: (220 nm) 12.684 min (100% Purity).
HRMS (ESI+): shows expected model isotope pattern.

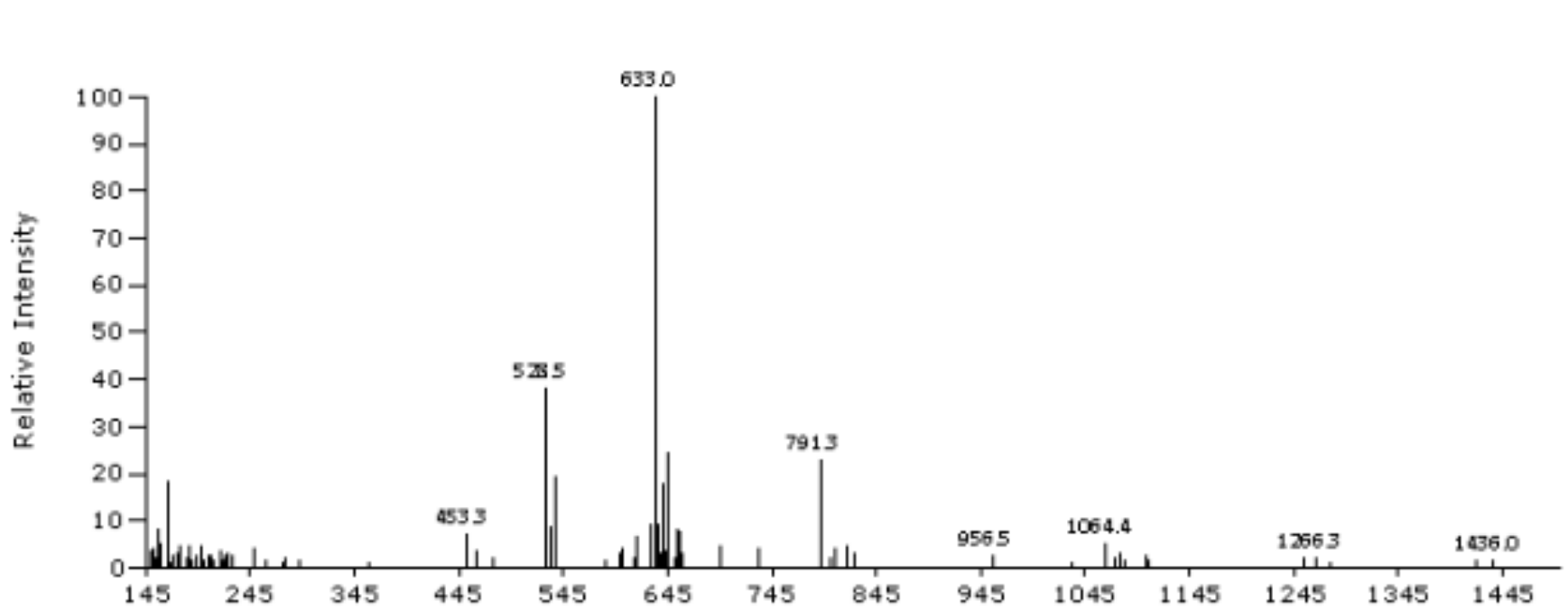

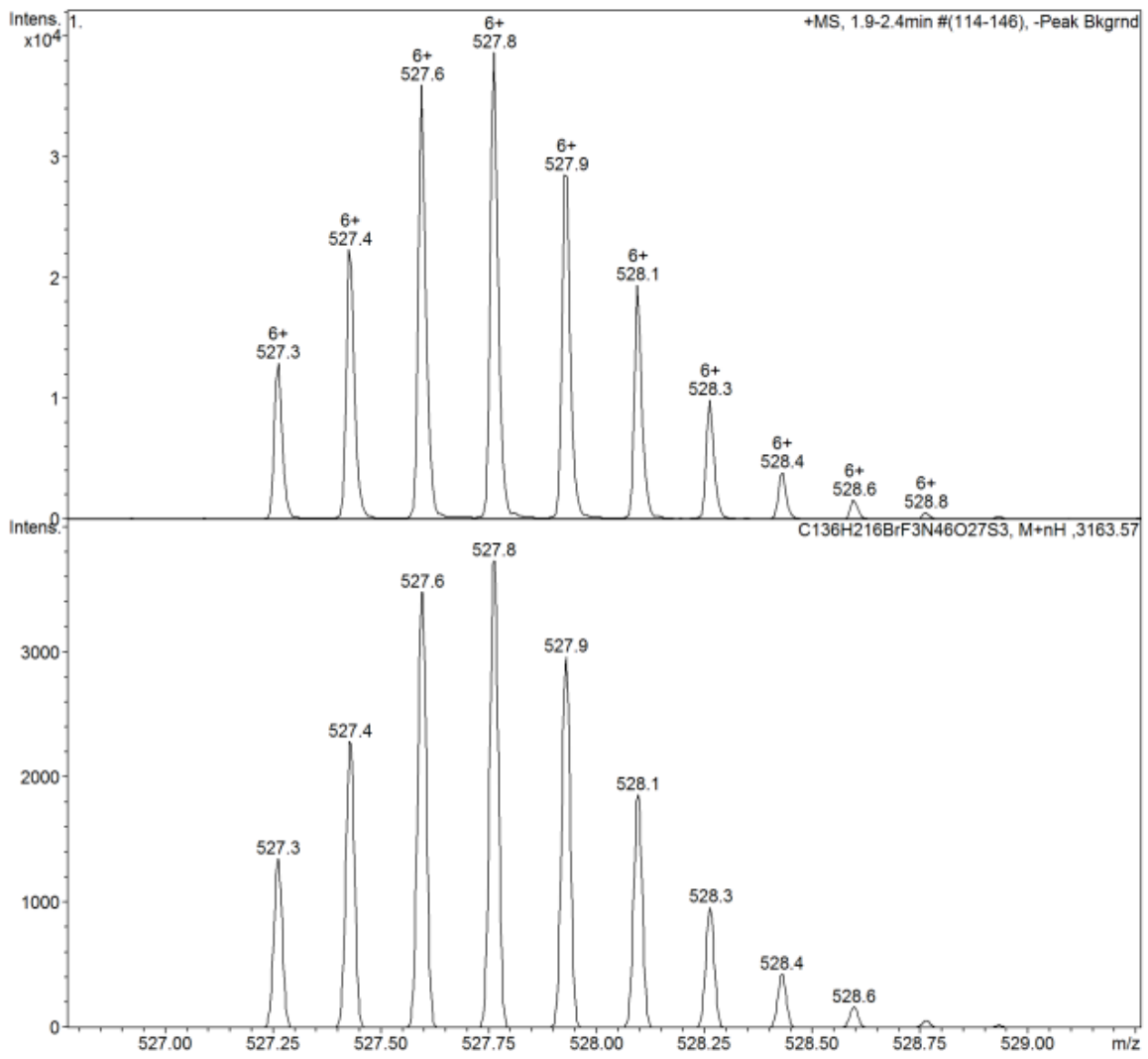

+MS, 1.9-2.4min #(114-146), -Peak Bkgrnd
C136H216BrF3N46O27S3, M+nH ,3163.57
Intens.
x10^4
6+ 527.3
6+ 527.4
6+ 527.6
6+ 527.8
6+ 527.9
6+ 528.1
6+ 528.3
6+ 528.4
6+ 528.6
6+ 528.8
m/z

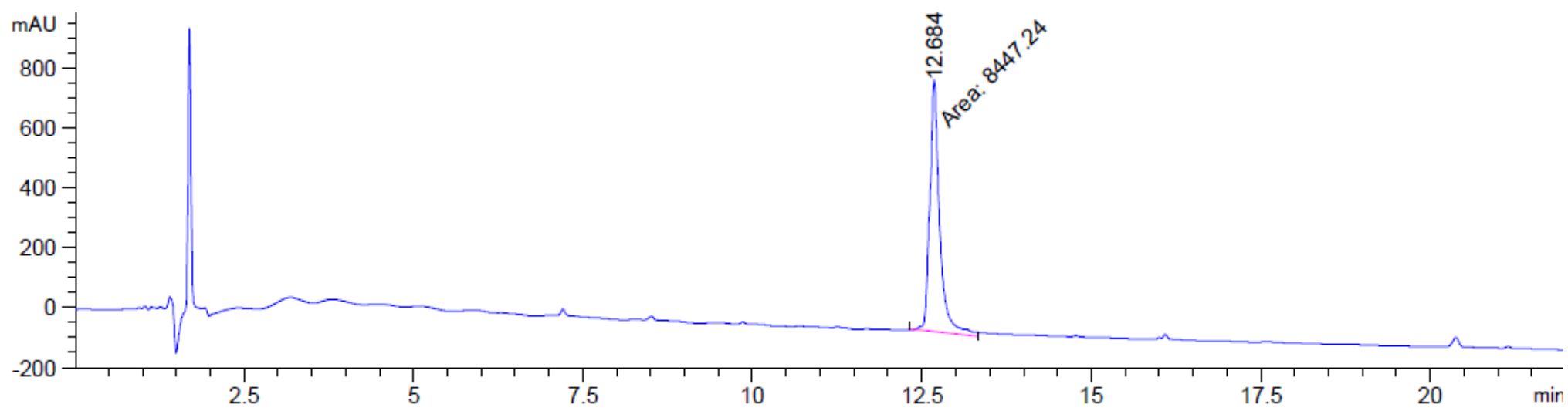

mAU
12.684
Area: 8447.24
min

cyclo-C(Tat)L(HX)(VHL)F(CF_3)IF(Br) (**66**)

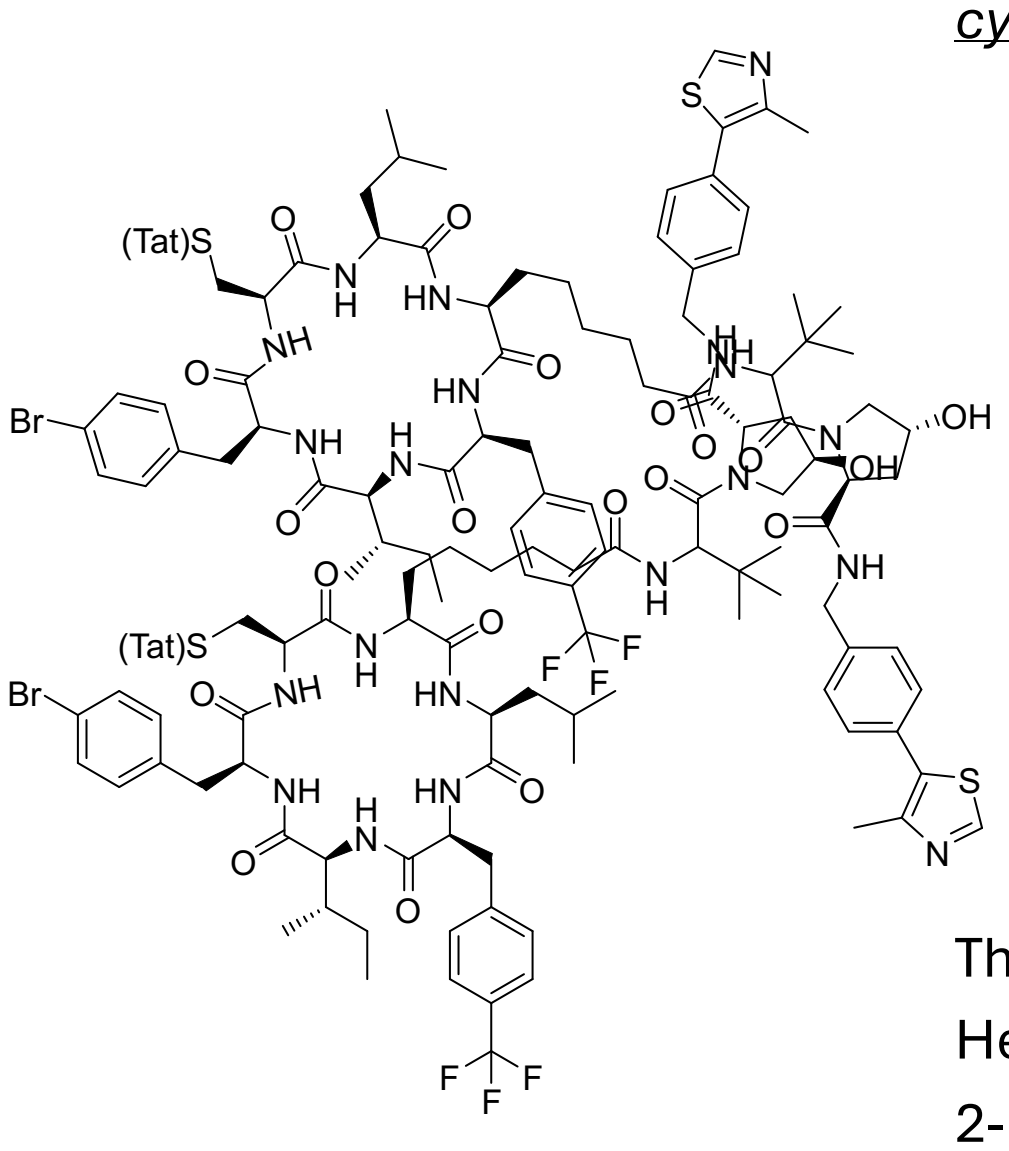

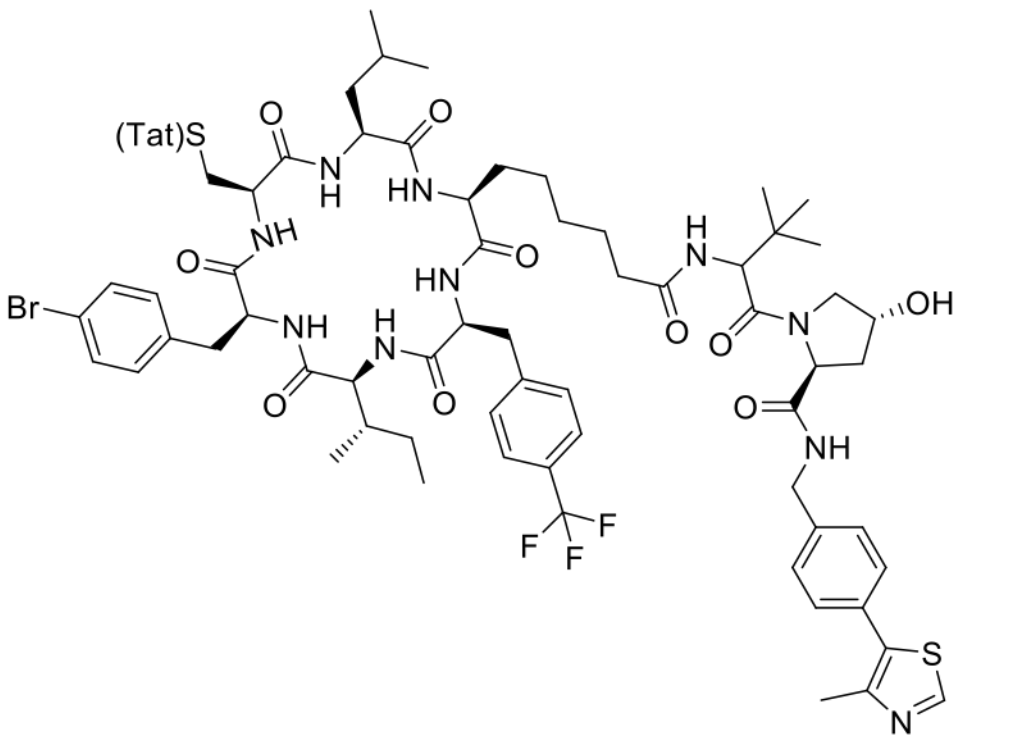

The linear peptide LC(StBu)F(Br)IF(CF_3)(HX) (HX= Hexanoic acid R group amino acid) was synthesised using 2-CTC resin SPPS at a 0.1 mmol scale. The linear peptide was then cyclised and subjected to TFA deprotection. The crude was subsequently purified via reverse-phase flash chromatography to yield C(StBu)L(HX)F(CF_3)IF(Br) as a white solid (21 mg, 0.02 mmol, 20%). The pure cyclic peptide (21 mg, 0.02 mmol) was dissolved in DMF (1 mL) to which was added DIPEA (10.2 µL, 0.06 mmol, 3 eq), HATU (11.4 mg, 0.03 mmol, 1.5 eq) and VH032 ligand (11.3 mgs, 0.024 mmol, 1.2 eq). The reaction was then stirred at ambient temperature overnight and purified by reverse phase chromatography to give CL(HX)(VHL)F(CF_3)IF(Br) as a white solid (17.3 mg, 0.012 mmol, 60%). The StBu group was then cleaved and the crude purified via reverse-phase HPLC. Fractions were analysed via LC/MS, desired fractions were then pooled, concentrated *in vacuo,* and lyophilised to yield the cyclic peptide CL(HX)(VHL)F(CF_3)IF(Br) as a white solid (6 mg, 0.0044 mmol, 38%). *cyclo*-CL(HX)(VHL)F(CF_3)IF(Br) (5 mg, 0.0037 mmol) was added to a solution of C(Spy)GRKKRRQRRRPPQ (11.4 mg, 0.006 mmol) dissolved in DMF (2 mL) and stirred in an inert atmosphere overnight at ambient temperature. The mixture was then purified by reverse-phase HPLC. Fractions analysed via LC/MS, desired fractions were then pooled, concentrated *in vacuo*, and lyophilised to yield *cyclo*- C(Tat)L(HX)(VHL)F(CF_3)IF(Br) as a white solid (10 mg, 0.0032 mmol, 86%).

LR MS (ESI+): m/z $[M+H]^+$ cald for $C_{137}H_{218}BrF_3N_{46}O_{27}S_3$, 3171.54, found; 794.8 [M+4H]4+, 636.0 $[M+5H]^{5+}$, 530.3 $[M+6H]^{6+}$.
HRMS (ESI+): shows expected model isotope pattern.
Analytical HPLC: Rt: (220 nm) 12.817 min (100% Purity).

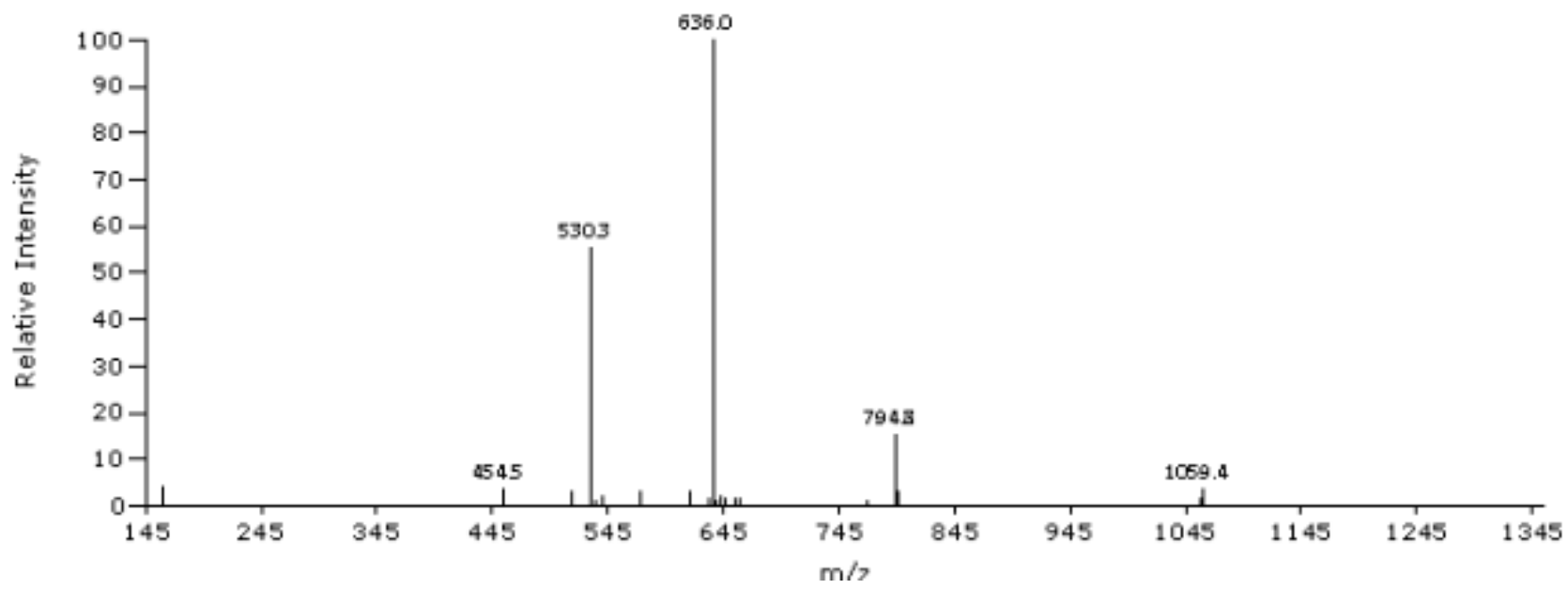

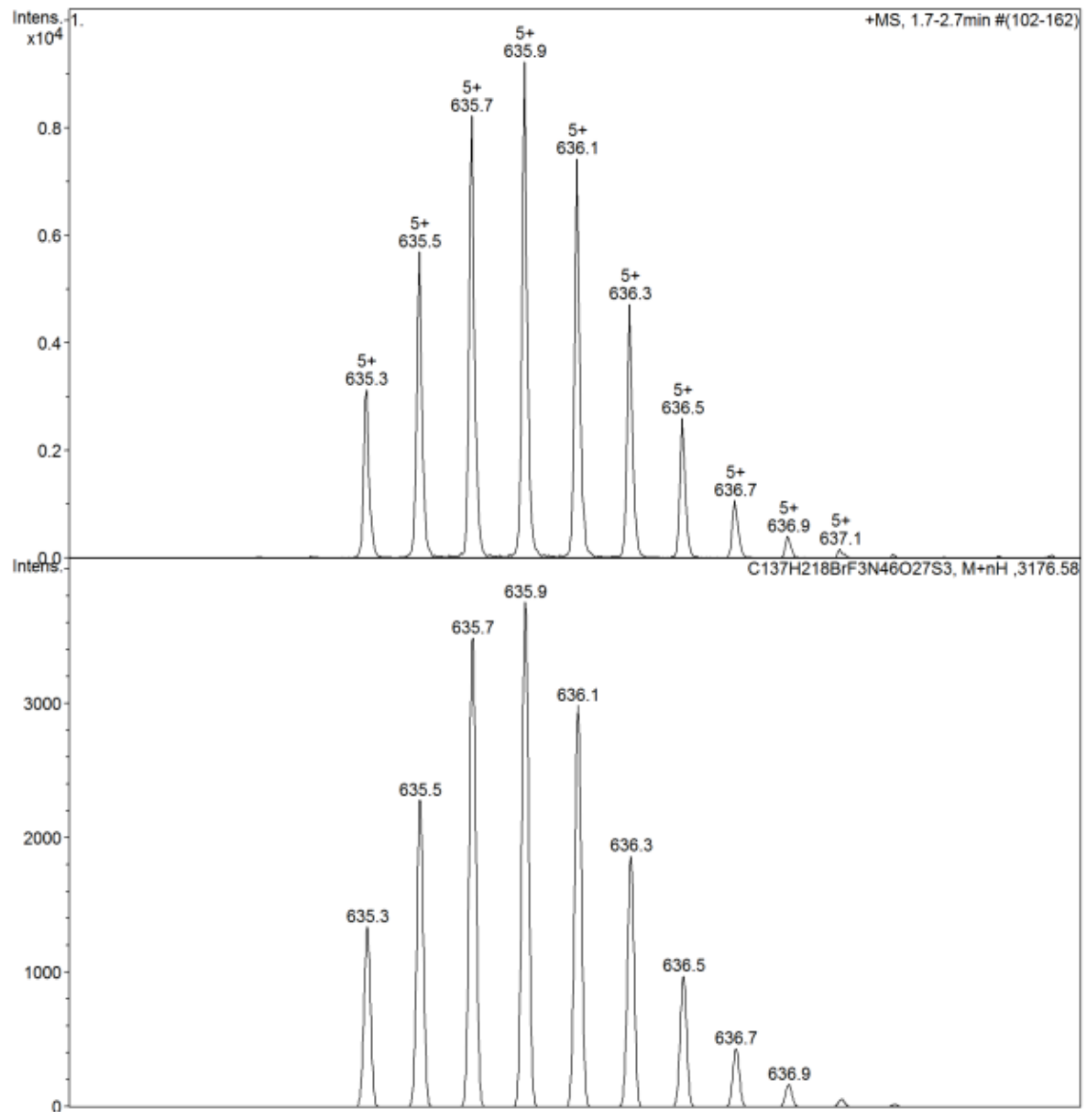
Intens.
x10^4
+MS, 1.7-2.7min #(102-162)
5+ 635.3
5+ 635.5
5+ 635.7
5+ 635.9
5+ 636.1
5+ 636.3
5+ 636.5
5+ 636.7
5+ 636.9
5+ 637.1
Intens.
C137H218BrF3N46O27S3, M+nH ,3176.58
635.3
635.5
635.7
635.9
636.1
636.3
636.5
636.7
636.9

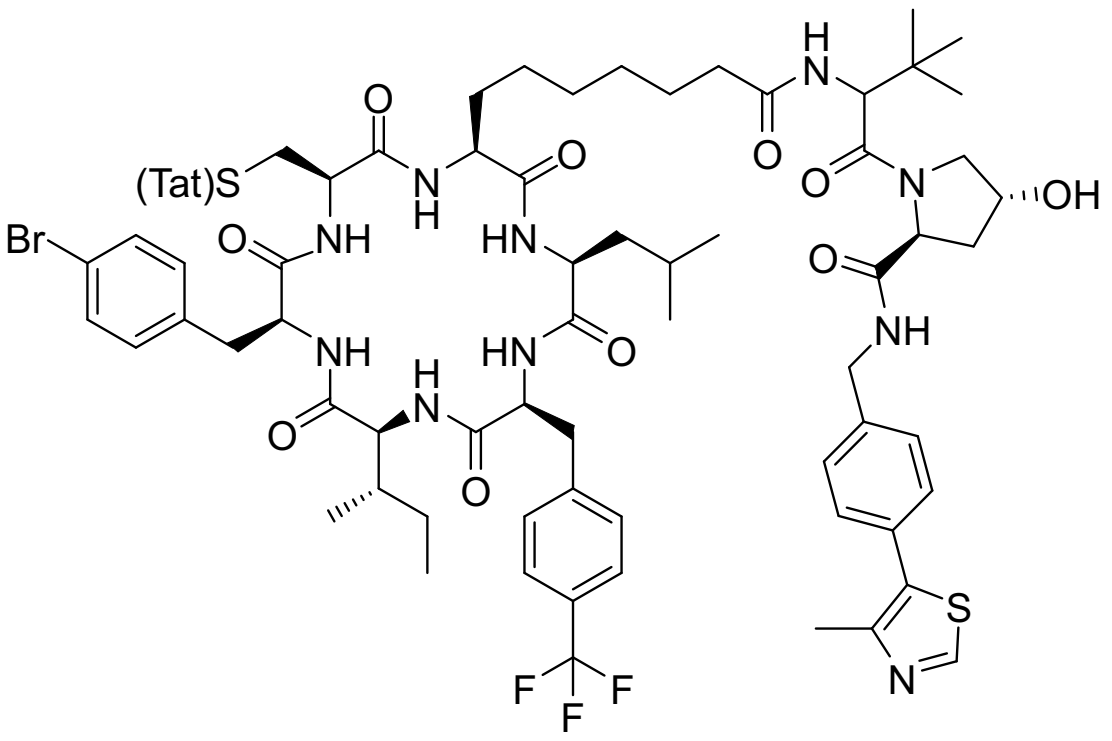
(Tat)S
Br
OH
F
F
F

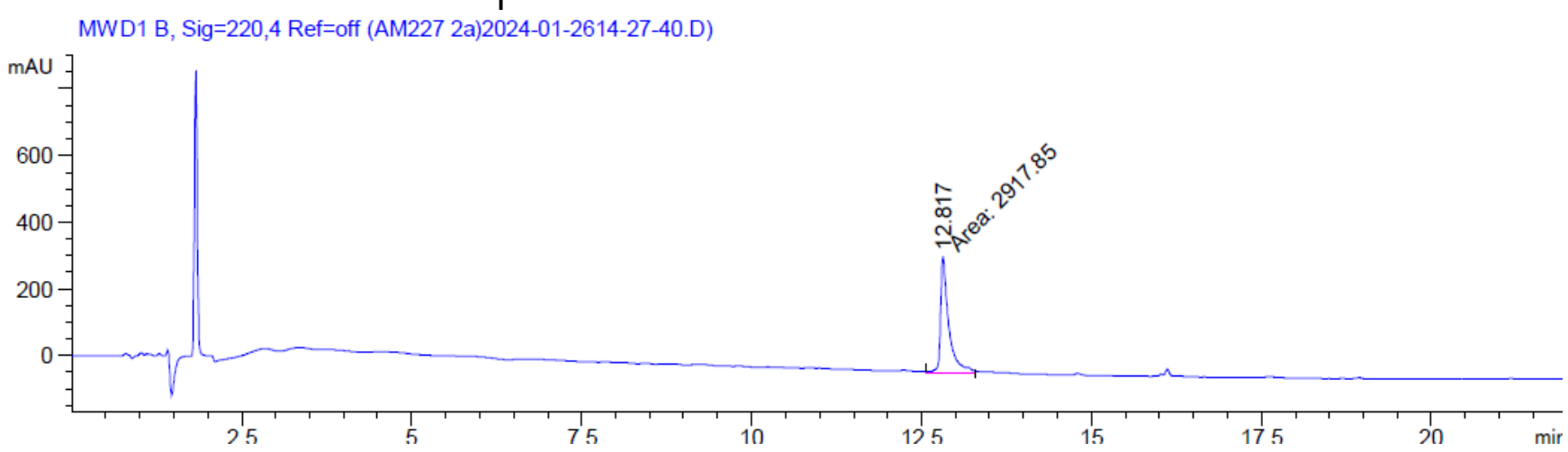
MWD1 B, Sig=220,4 Ref=off (AM227 2a)2024-01-2614-27-40.D)
mAU
12.817
Area: 2917.85
min

<u>*Cyclo*-C(Tat)L(HP)(VHL)F(CF_3)IF(Br) (**67**)</u>

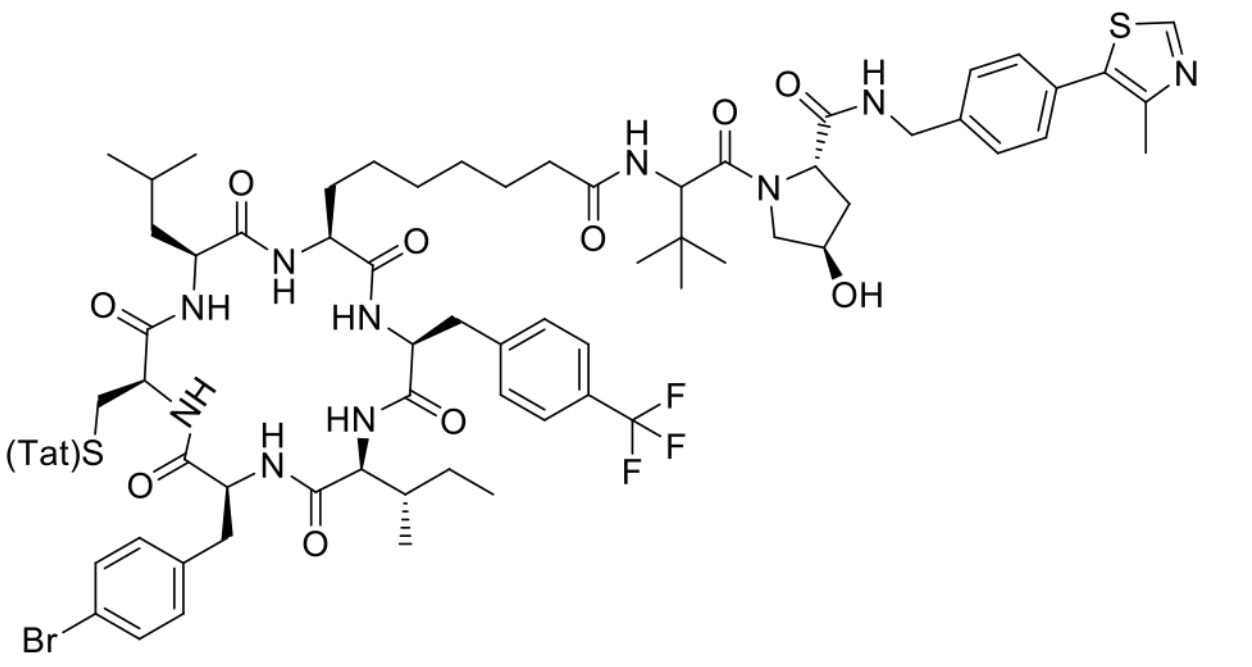

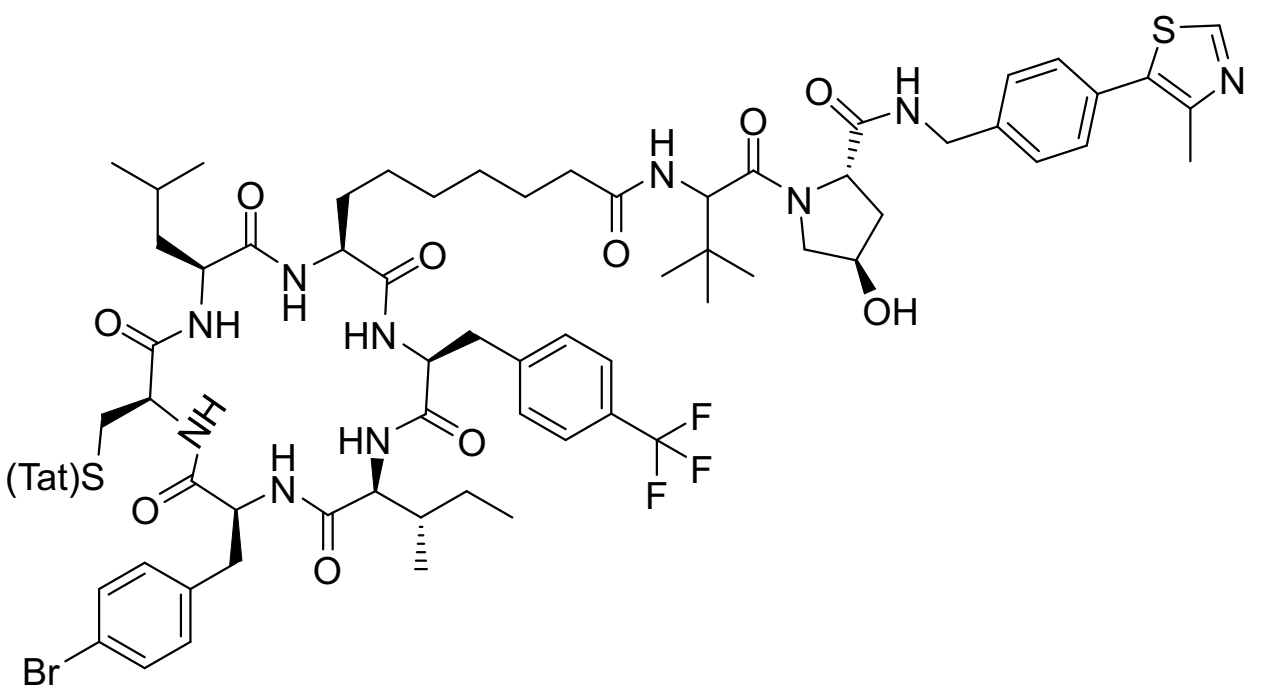

The linear peptide LC(StBu)F(Br)IF(CF_3)HP (HP = Heptanoic acid R group amino acid) was synthesised using 2-CTC resin SPPS at a 0.1 mmol scale. The linear peptide was then cyclised and subjected to TFA deprotection. The crude mixture was purified via reverse-phase flash chromatography to yield C(StBu)L(HP)F(CF_3)IF(Br) as a white solid (12.7 mg, 0.013 mmol, 13%). The pure cyclic peptide (12.7 mg, 0.013 mmol) was dissolved in DMF (1 mL) to which was added DIPEA (7 µl, 0.039 mmol, 3 eq), HATU (7.6 mg, 0.020 mmol, 1.5 eq) and VH032 ligand (6 mgs, 0.014 mmol, 1.1 eq). The reaction was then stirred at ambient temperature overnight and purified by reverse phase chromatography to give C(StBu)L(HP)(VHL)F(CF_3)IF(Br) as a white solid (7 mg, 0.004 mmol, 37%). The StBu group was then cleaved and the crude purified via reverse-phase HPLC. Fractions were analysed via LC/MS, desired fractions were then pooled, concentrated *in vacuo,* and lyophilised to yield the cyclic peptide CL(HP)(VHL)F(CF_3)IF(Br) as a white solid (2.2 mg, 0.0016 mmol, 40%). *cyclo*-CL(HP)(VHL)F(CF_3)IF(Br) (1.2 mg, 0.0009 mmol) was added to a solution of C(Spy)GRKKRRQRRRPPQ (3.5 mg, 0.0018 mmol) dissolved in DMF (2 mL) and stirred in an inert atmosphere overnight at ambient temperature. The mixture was then purified by reverse-phase HPLC. Fractions analysed via LC/MS, desired fractions were then pooled, concentrated *in vacuo*, and lyophilised to yield *cyclo*- C(Tat)L(HP)(VHL)F(CF_3)IF(Br) as a white solid (2 mg, 0.00063 mmol, 70%).

LR MS (ESI+): m/z $[M+H]^+$ cald for $C_{138}H_{220}BrF_3N_{46}O_{27}S_3$, 3185.56, found; 1063.9 $[M+3H]^{3+}$, 797.9 $[M+4H]^{4+}$, 638.8 $[M+5H]^{5+}$.
HRMS (ESI+): shows expected model isotope pattern.
Analytical HPLC: Rt: (220 nm) 12.897 min (100% Purity).

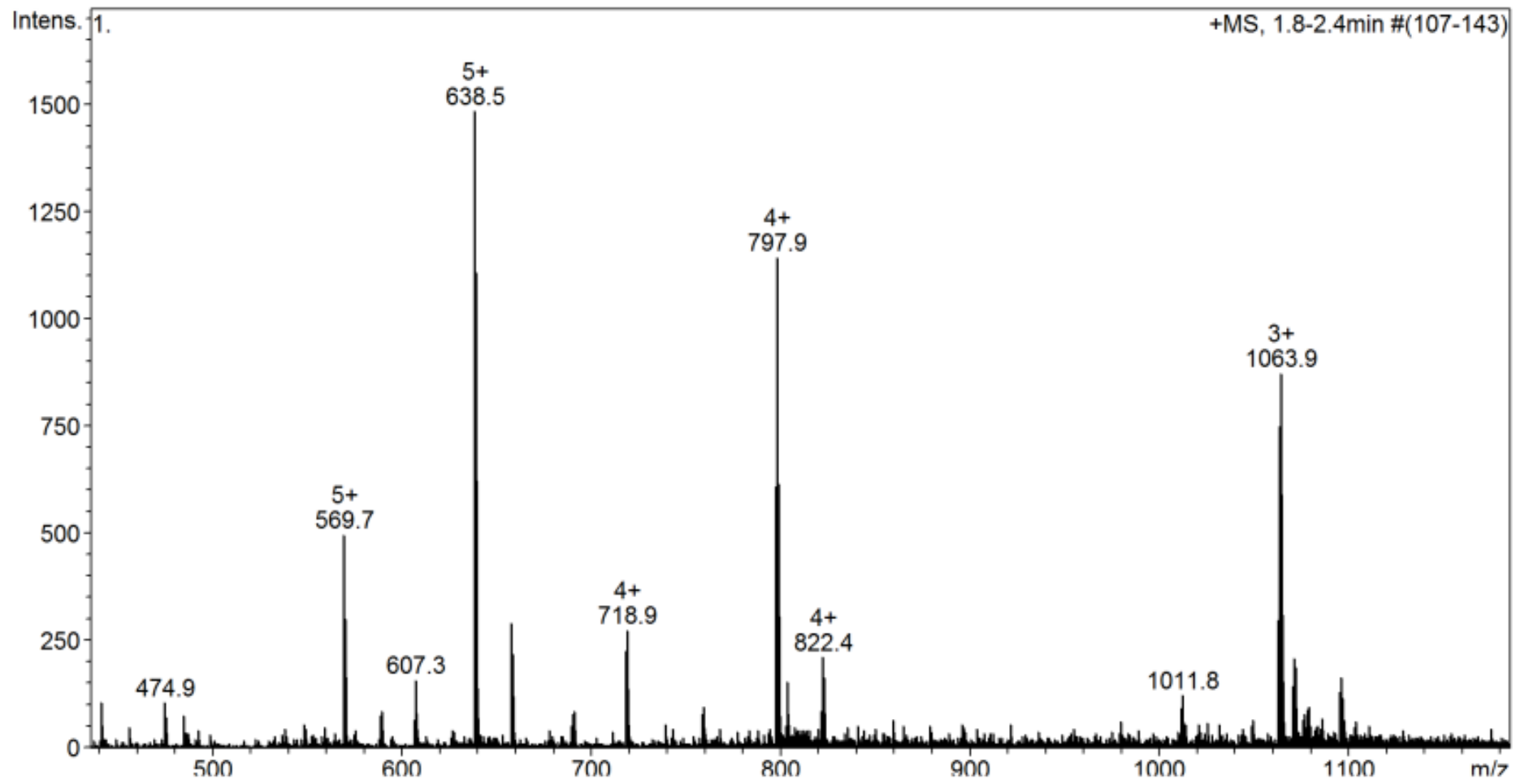
Intens.
1.
+MS, 1.8-2.4min #(107-143)
5+
638.5
4+
797.9
3+
1063.9
5+
569.7
4+
718.9
4+
822.4
474.9
607.3
1011.8
m/z

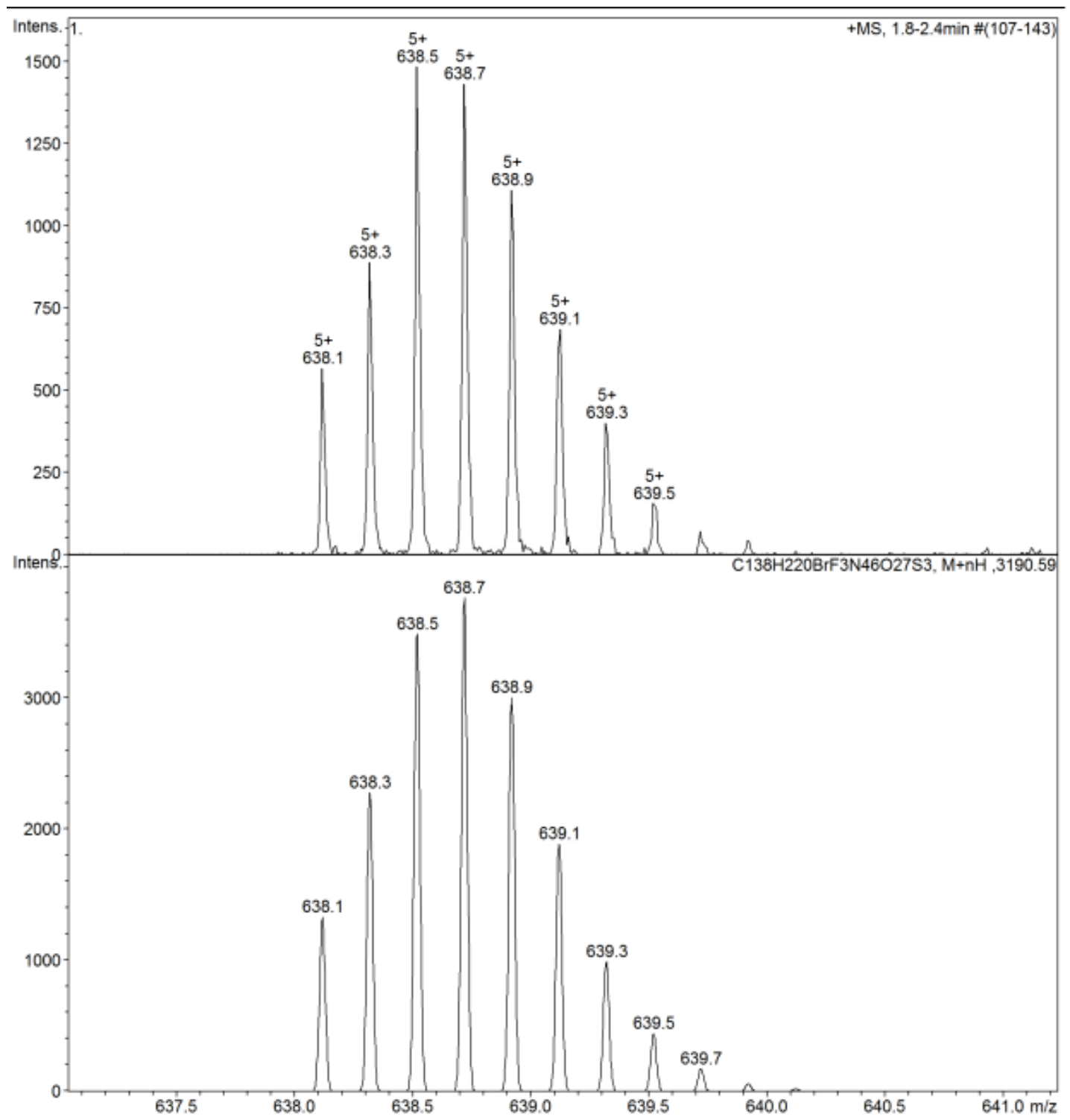
Intens.
1.
+MS, 1.8-2.4min #(107-143)
5+ 638.1
5+ 638.3
5+ 638.5
5+ 638.7
5+ 638.9
5+ 639.1
5+ 639.3
5+ 639.5
Intens.
C138H220BrF3N46O27S3, M+nH ,3190.59
638.1
638.3
638.5
638.7
638.9
639.1
639.3
639.5
639.7
m/z

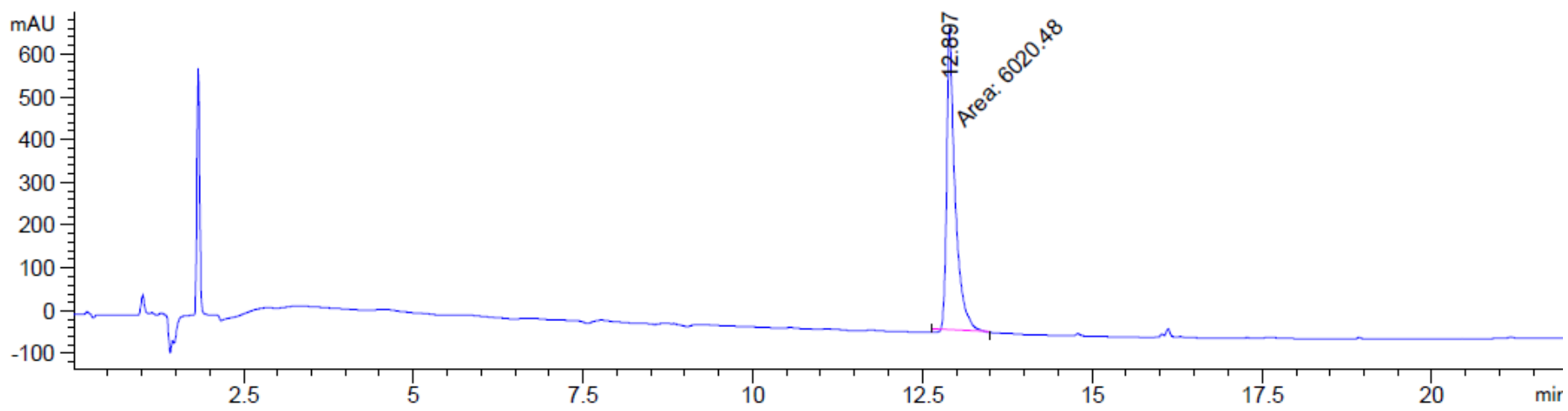
mAU
12.897
Area: 6020.48
min

cyclo-C(Tat)E(PEG_4)(VHL)LF(CF_3)IF(Br) (**68**)

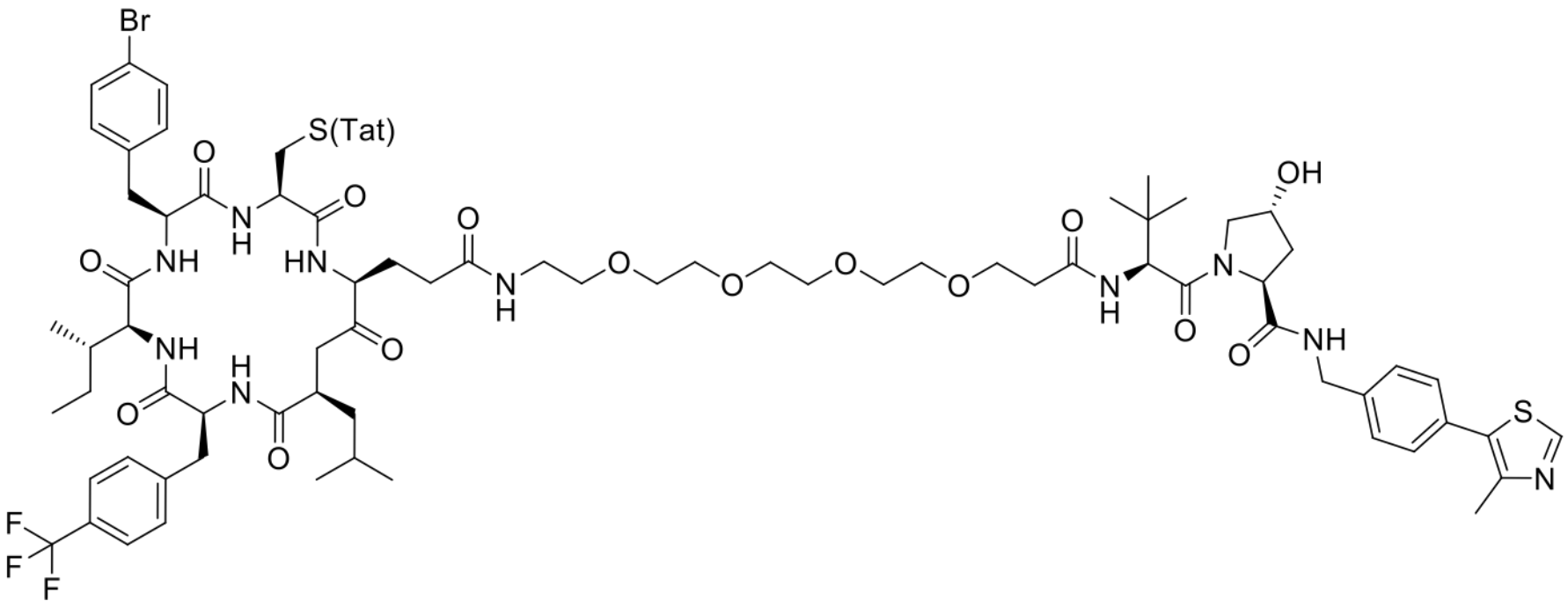

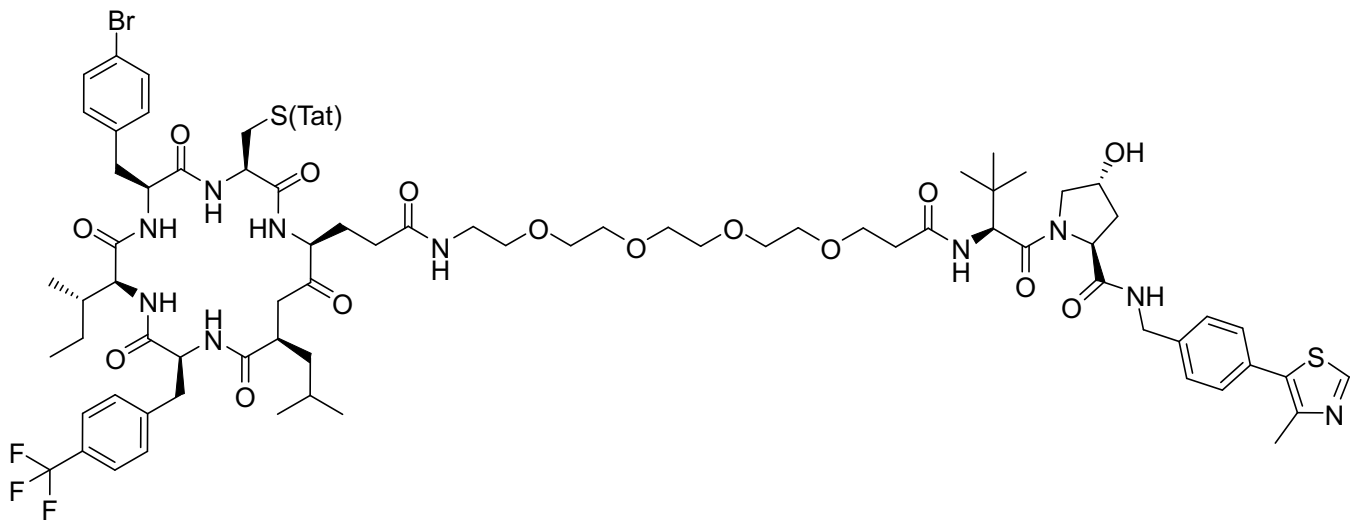

The linear peptide EC(StBu)F(Br)IF(CF_3)L was synthesised using 2-CTC resin SPPS at a 0.2 mmol scale. The linear peptide was then cyclised and subjected to TFA deprotection. The crude mixture was subsequently purified via reverse-phase flash chromatography to yield C(StBu)ELF(CF_3)IF(Br) as a white solid (70 mg, 0.071 mmol, 35%). The pure cyclic peptide (17.5 mg, 0.018 mmol) was dissolved in DMF (1 mL) to which was added DIPEA (18 µL, 0.054 mmol, 3 eq), HATU (8.3 mg, 0.022 mmol, 1.2 eq) and tert-butyl 1-amino-3,6,9,12-tetraoxapentadecan-15-oate (11.6 mgs, 0.036 mmol, 2 eq). The reaction was then stirred at ambient temperature and reaction progress was monitored by LC-MS. Once completed, the reaction crude was concentrated *in vacuo* and subjected to TFA deprotection. The TFA reaction mixture was then concentrated *in vacuo* and purified by reverse phase chromatography to give C(StBu)E(PEG_4)LF(CF_3)IF(Br) as a white solid (13 mg, 0.011 mmol, 59%).
C(StBu)E(PEG_4)LF(CF_3)IF(Br) (13 mg, 0.011 mmol) was dissolved in DMF (1 mL) to which was added DIPEA (5.3 µl, 0.033mmol, 3 eq), HATU (6 mg, 0.013 mmol, 1.2 eq) and VH032 ligand (5.4 mg, 0.013 mmol, 1.2 eq). The reaction was then stirred at ambient temperature and reaction progress monitored by LC-MS. Once completed, the reaction crude was purified by reverse phase chromatography to give C(StBu)E(PEG_4(VHL))LF(CF_3)IF(Br) as a white solid (7.4 mg, 0.0045 mmol, 41%). The StBu group was then cleaved and the crude purified via reverse-phase HPLC. Fractions were analysed via LC/MS, desired fractions were then pooled, concentrated *in vacuo,* and lyophilised to yield the cyclic peptide CE(PEG_4(VHL))LF(CF_3)IF(Br) as a white solid (4.1 mg, 0.0025 mmol, 55%). *cyclo*- CE(PEG_4(VHL))LF(CF_3)IF(Br) (4.1 mg, 0.0025 mmol) was added to a solution of C(Spy)GRKKRRQRRRPPQ (9 mg, 0.0047 mmol) dissolved in DMF (2 mL) and stirred in an inert atmosphere overnight at ambient temperature. The mixture was then purified by reverse-phase HPLC. Fractions analysed via LC/MS, desired fractions were then pooled, concentrated *in vacuo*, and lyophilised to yield *cyclo*- C(Tat)E(PEG_4(VHL))LF(CF_3)IF(Br) as a white solid (5.3 mg, 0.0016 mmol, 63%).

LR MS (ESI+): m/z $[M+H]^+$ cald for $C_{146}H_{234}BrF_3N_{46}O_{32}S_3$, 3376.64, found; 846.5 $[M+4H]^{4+}$, 676.9 $[M+5H]^{5+}$, 564.6 $[M+6H]^{6+}$.
HRMS (ESI+): shows expected model isotope pattern.

Analytical HPLC: Rt: (220 nm) 12.425 min (100% Purity).

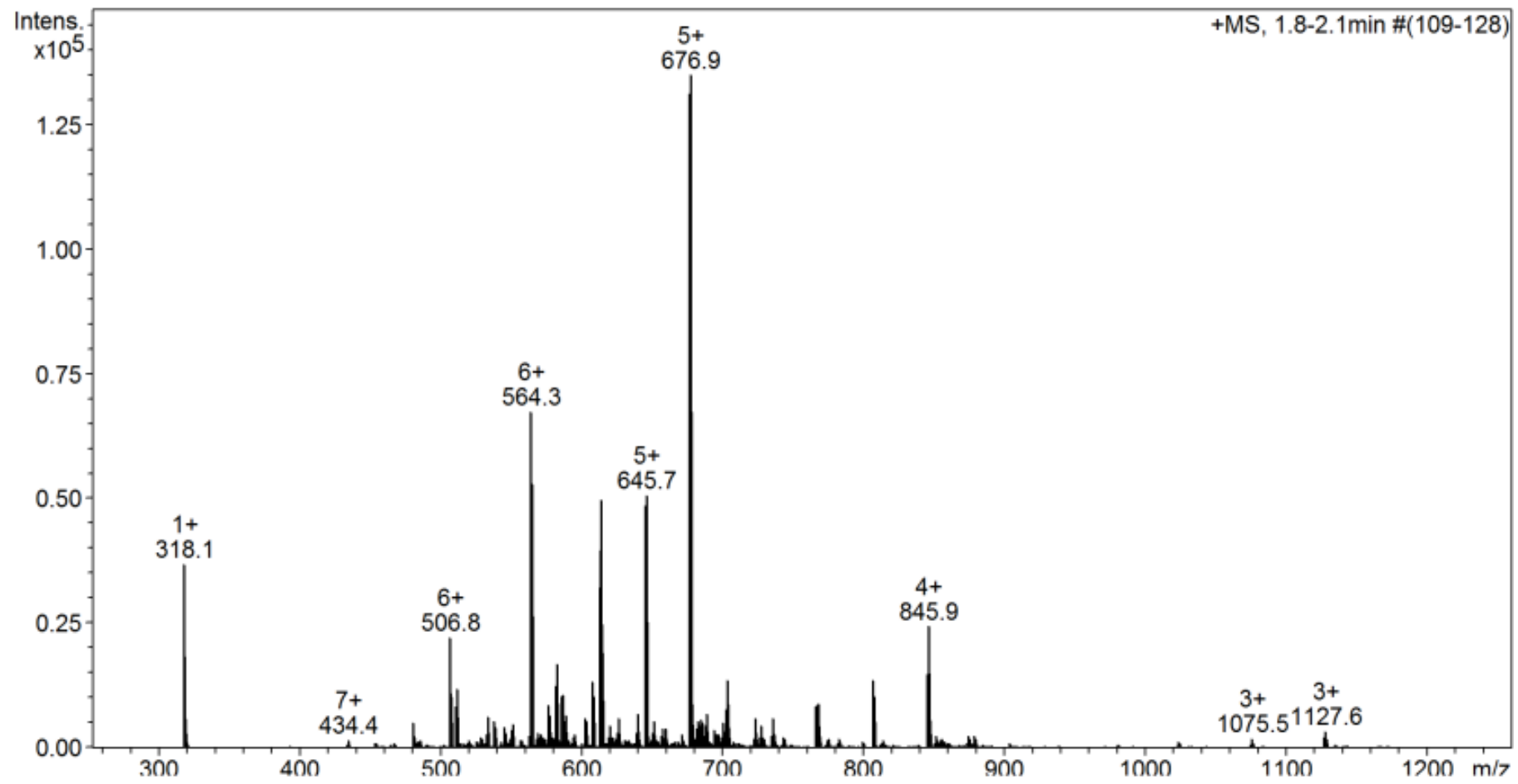

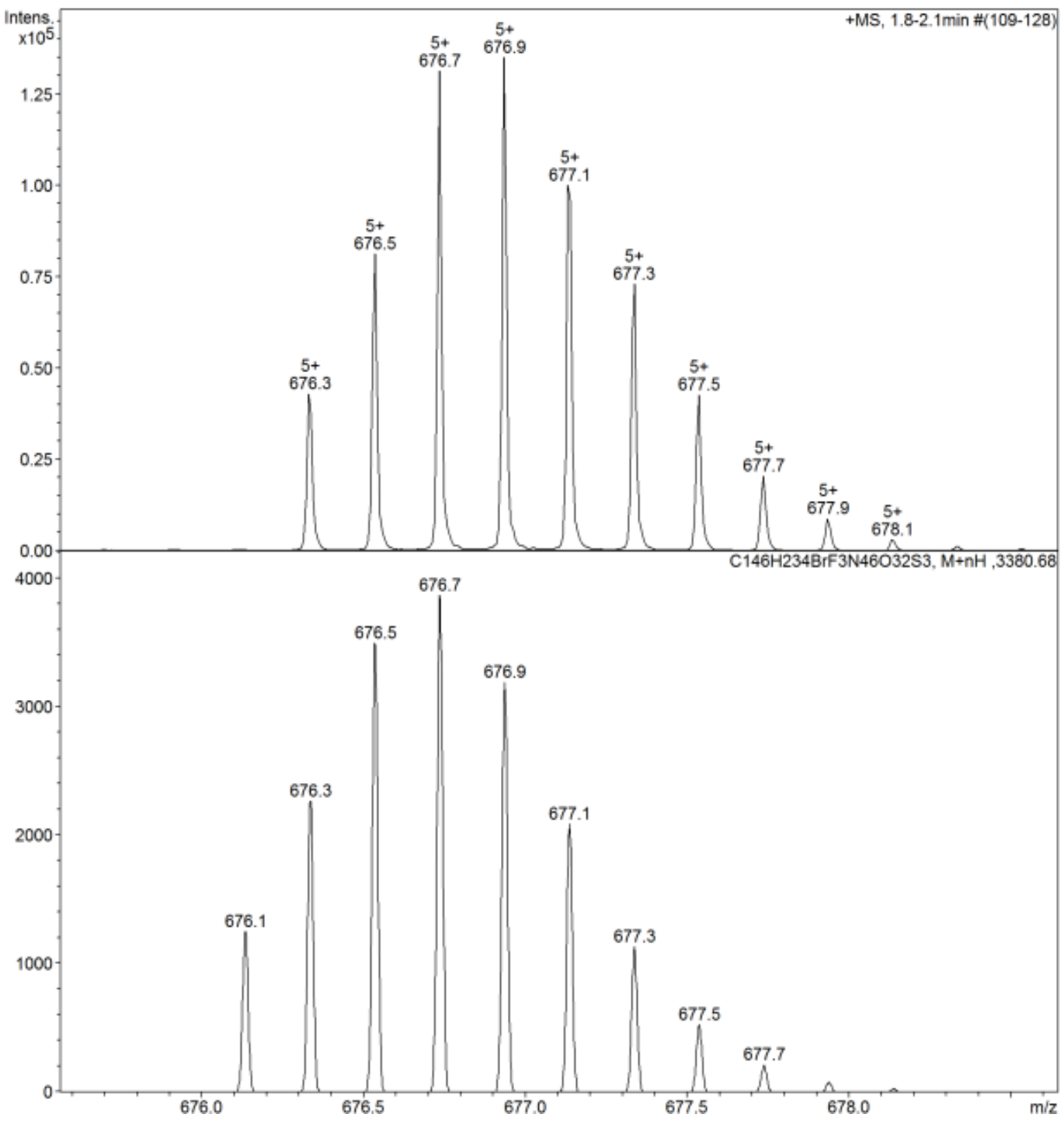

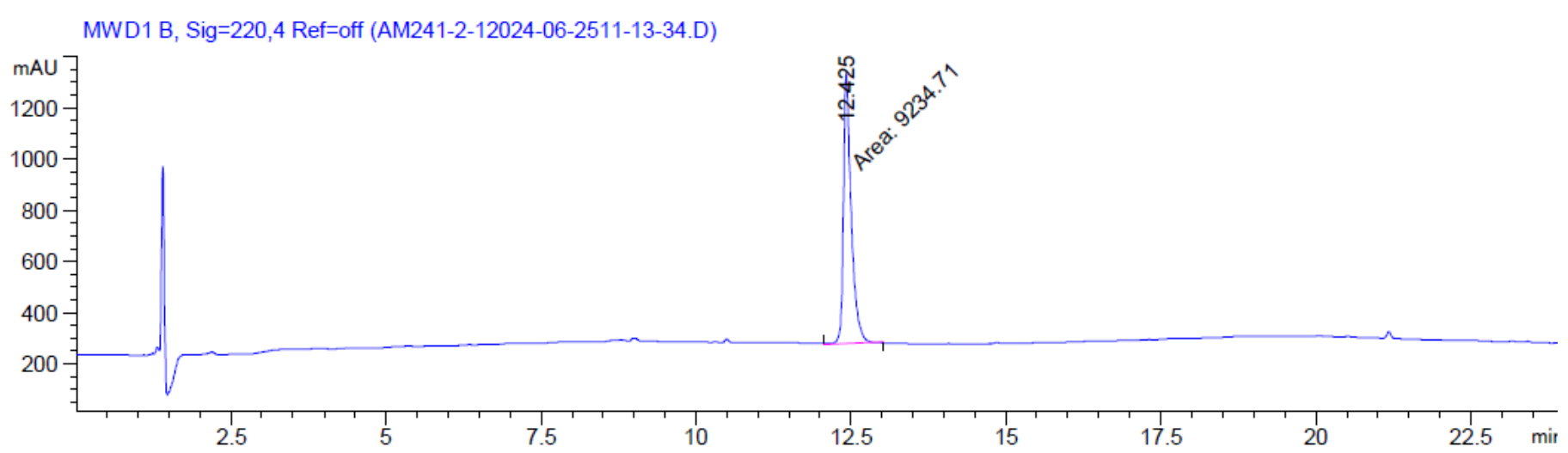

<u>*cyclo*-C(Tat)E(PEG$_5$)(VHL)LF(CF$_3$)IF(Br) (**69**)</u>

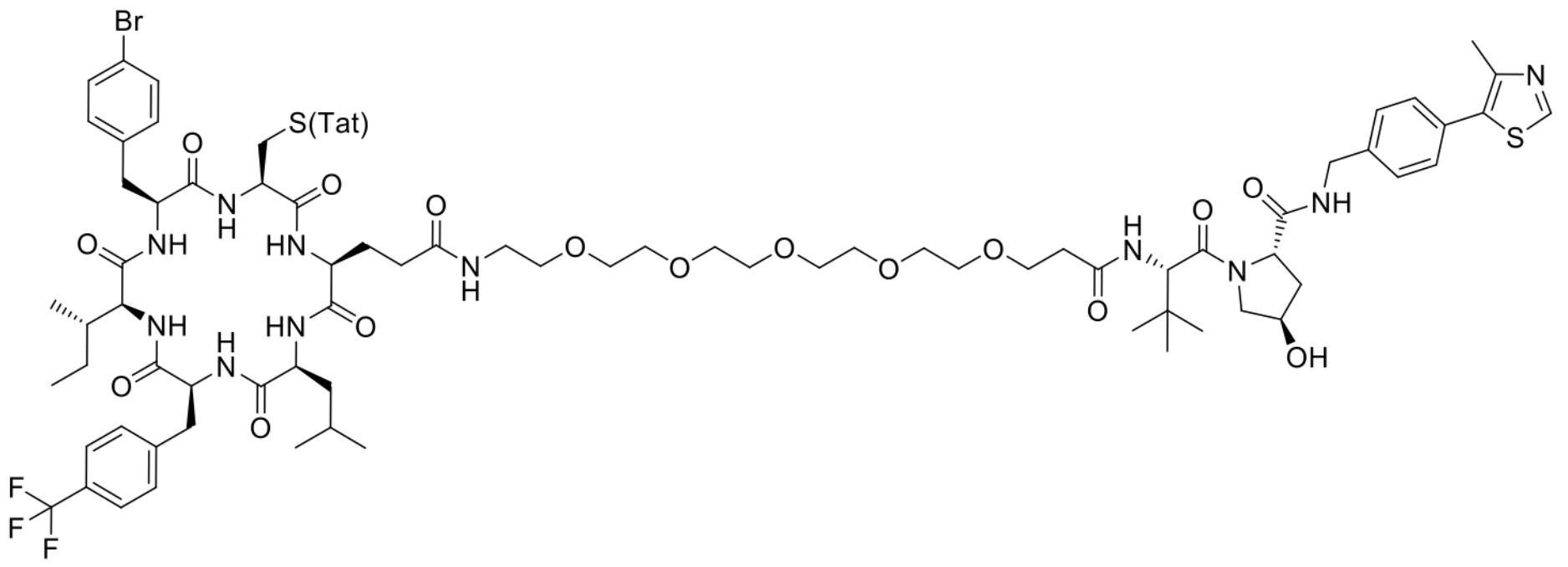

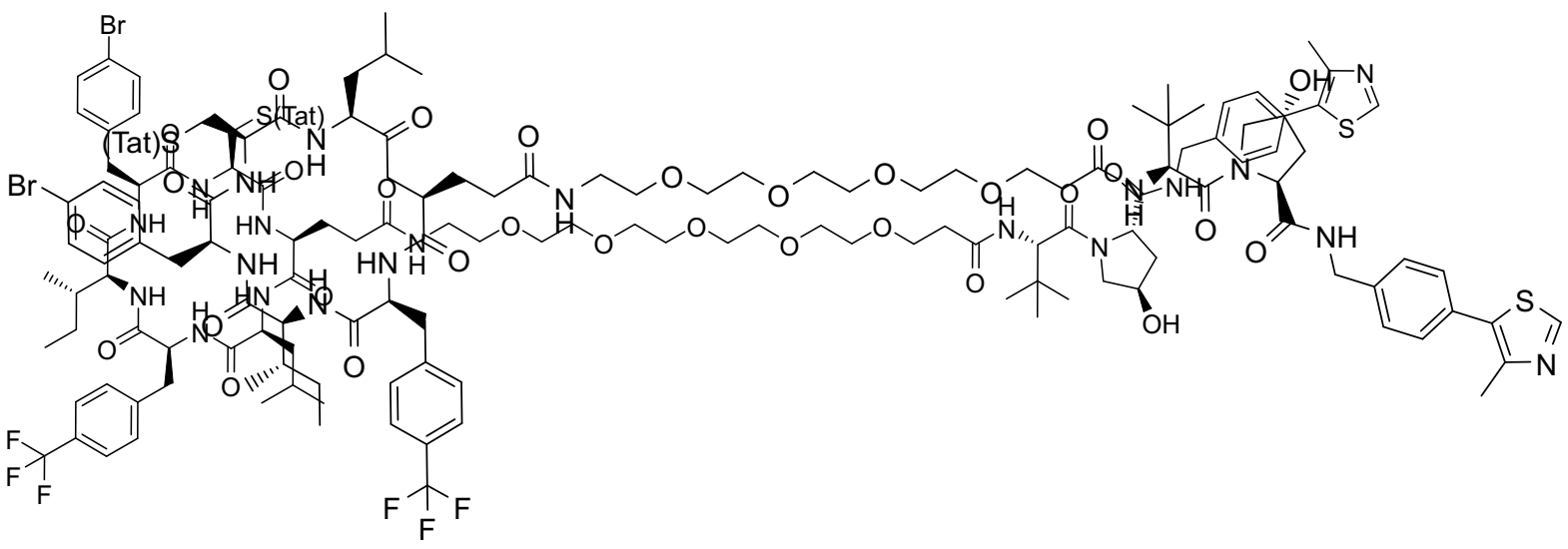

The linear peptide EC(StBu)F(Br)IF(CF$_3$)L was synthesised using 2-CTC resin SPPS as outlined in 5.1.2 at a 0.2 mmol scale. The linear peptide was then cyclised via HATU cyclisation conditions stated in 5.1.3. The cyclic peptide crude was then subjected to TFA deprotection as described in 5.1.4 and was subsequently purified via reverse-phase flash chromatography to yield C(StBu)ELF(CF$_3$)IF(Br) as a white solid (70 mg, 0.071 mmol, 35%). The pure cyclic peptide (17.5 mg, 0.018 mmol) was dissolved in DMF (1 mL) to which was added DIPEA (18 µl, 0.054 mmol, 3 eq), HATU (8.3 mg, 0.022 mmol, 1.2 eq) and *tert*-Butyl-1-amino-3,6,9,12,15-pentaoxaoctadecan-18-oate (13.1 mgs, 0.036 mmol, 2 eq). The reaction was then stirred at ambient temperature and reaction progress was monitored by LC-MS. Once completed, the reaction crude was concentrated *in vacuo* and subjected to TFA deprotection as detailed in 5.1.4. The TFA reaction mixture was then concentrated *in vacuo* and purified by reverse phase chromatography to give C(StBu)E(PEG$_5$)LF(CF$_3$)IF(Br) as a white solid (15 mg, 0.012 mmol, 67%). C(StBu)E(PEG$_5$)LF(CF$_3$)IF(Br) (15 mg, 0.012 mmol, 67%) was dissolved in DMF (1 mL) to which was added DIPEA (6 µl, 0.035 mmol, 3 eq), HATU (6.7 mg, 0.014 mmol, 1.2 eq) and VH032 ligand (6 mg, 0.014 mmol, 1.2 eq). The reaction was then stirred at ambient temperature and reaction progress monitored by LC-MS. Once completed, the reaction crude was purified by reverse phase chromatography to give C(StBu)E(PEG$_5$(VHL))LF(CF$_3$)IF(Br) as a white solid (9.5 mg, 0.0056 mmol, 47 %). The StBu group was then cleaved as described in 5.1.5 and the crude purified via reverse-phase HPLC. Fractions were analysed via LC/MS, desired fractions were then pooled, concentrated *in vacuo,* and lyophilised to yield the cyclic peptide CE(PEG$_5$(VHL))LF(CF$_3$)IF(Br) as a white solid (4.3 mg, 0.0025 mmol, 47%). *cyclo*-CE(PEG$_5$(VHL))LF(CF$_3$)IF(Br) (4.3 mg, 0.0025 mmol) was added to a solution of C(Spy)GRKKRRQRRRPPQ (9 mg, 0.005 mmol) dissolved in DMF (2 mL) and stirred in an inert atmosphere overnight at ambient temperature. The mixture was then purified by reverse-phase HPLC. Fractions analysed via LC/MS, desired fractions were then pooled, concentrated *in vacuo*, and lyophilised to yield *cyclo*- C(Tat)E(PEG$_5$(VHL))LF(CF$_3$)IF(Br) as a white solid (6 mg, 0.0018 mmol, 70%).

LR MS (ESI+): m/z $[M+H]^{+}$ cald for $C_{147}H_{237}BrF_3N_{47}O_{33}S_3$, 3421.7, found; 856.7 $[M+4H]^{4+}$, 686.2 $[M+5H]^{5+}$, 571.9 $[M+6H]^{6+}$.

HRMS (ESI+): shows expected model isotope pattern.

Analytical HPLC: Rt: (220 nm) 12.121 min (96.5% Purity).

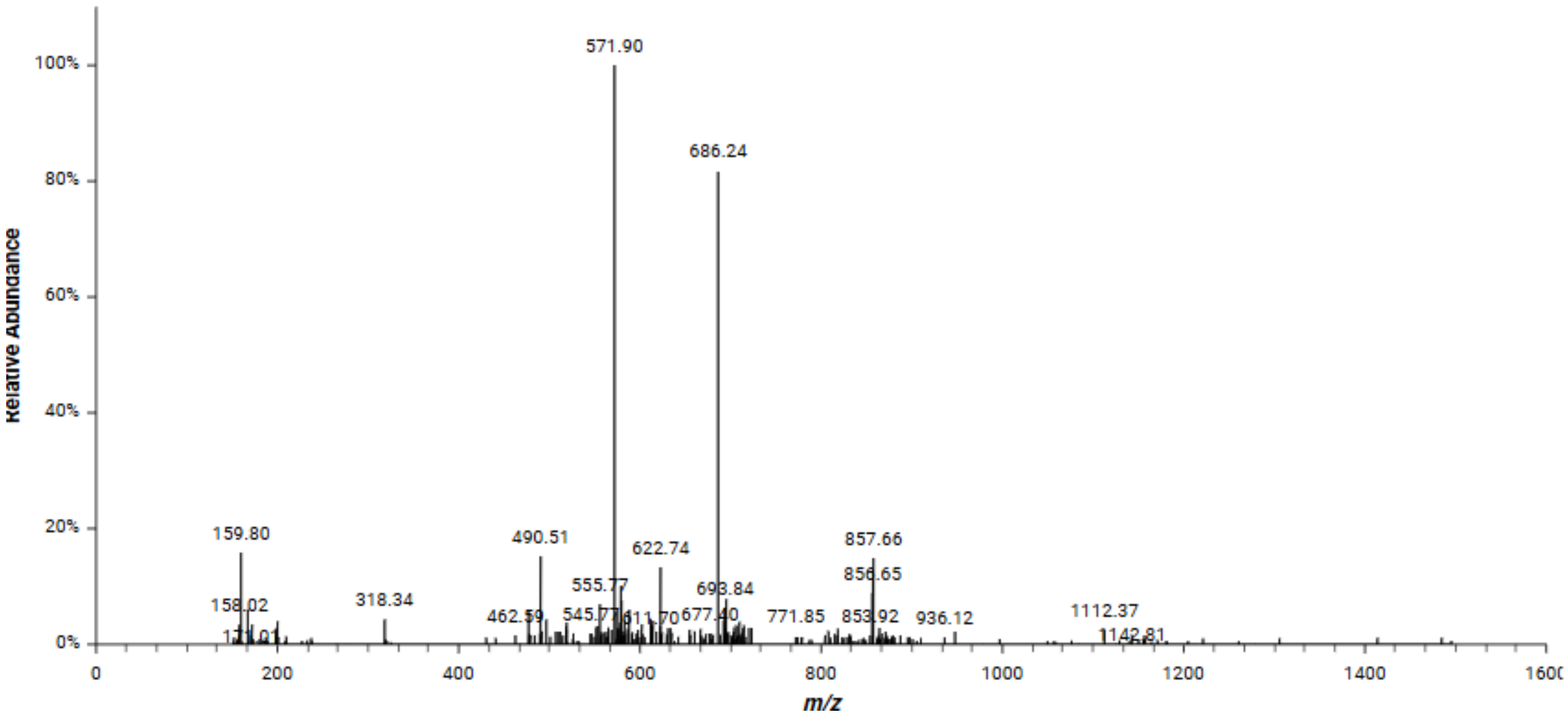

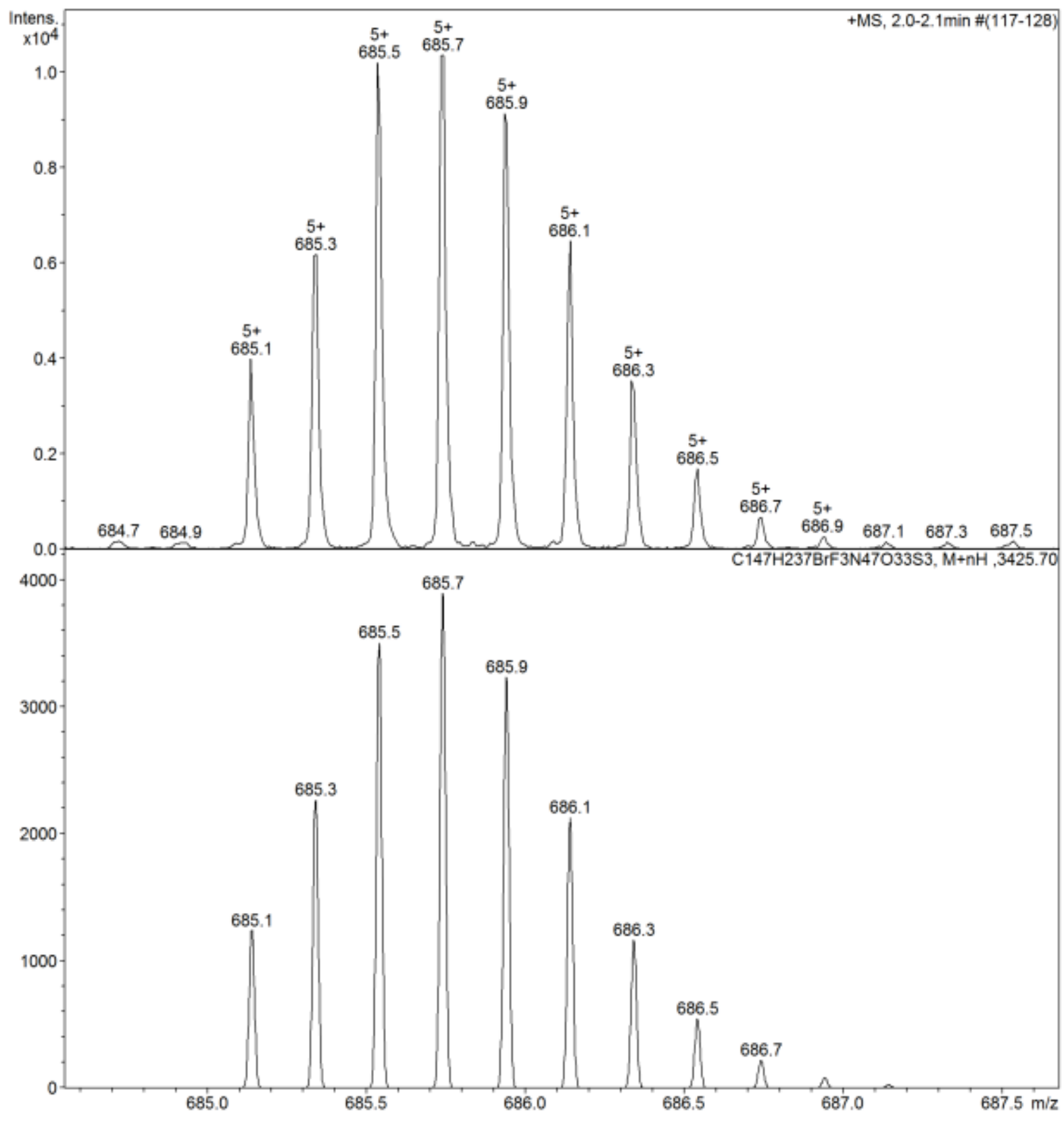

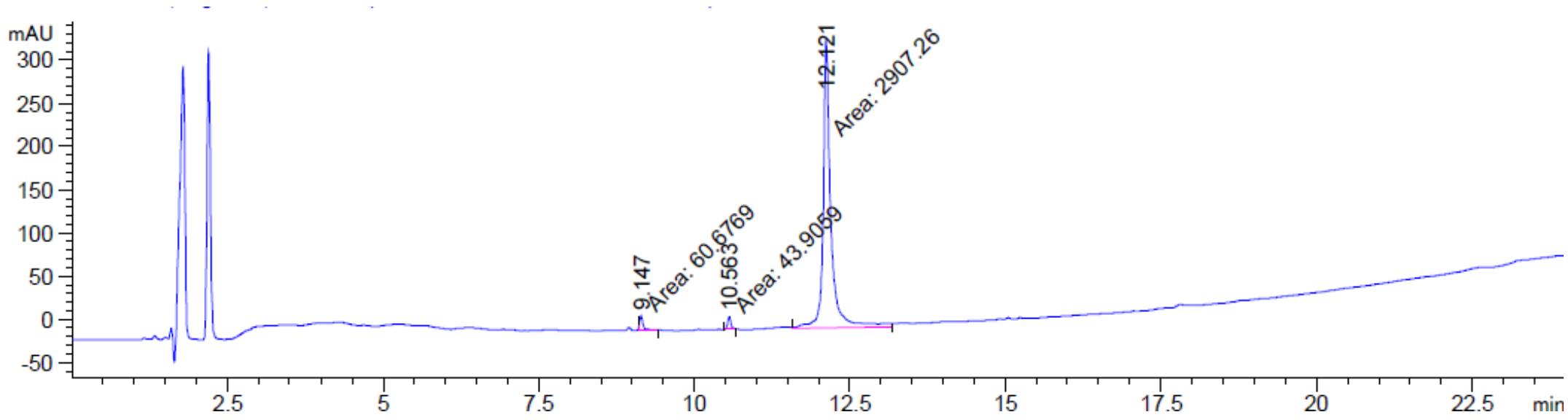

mAU
300
250
200
150
100
50
0
-50
2.5
5
7.5
10
12.5
15
17.5
20
22.5
min
9.147
Area: 60.6769
10.563
Area: 43.9059
12.121
Area: 2907.26

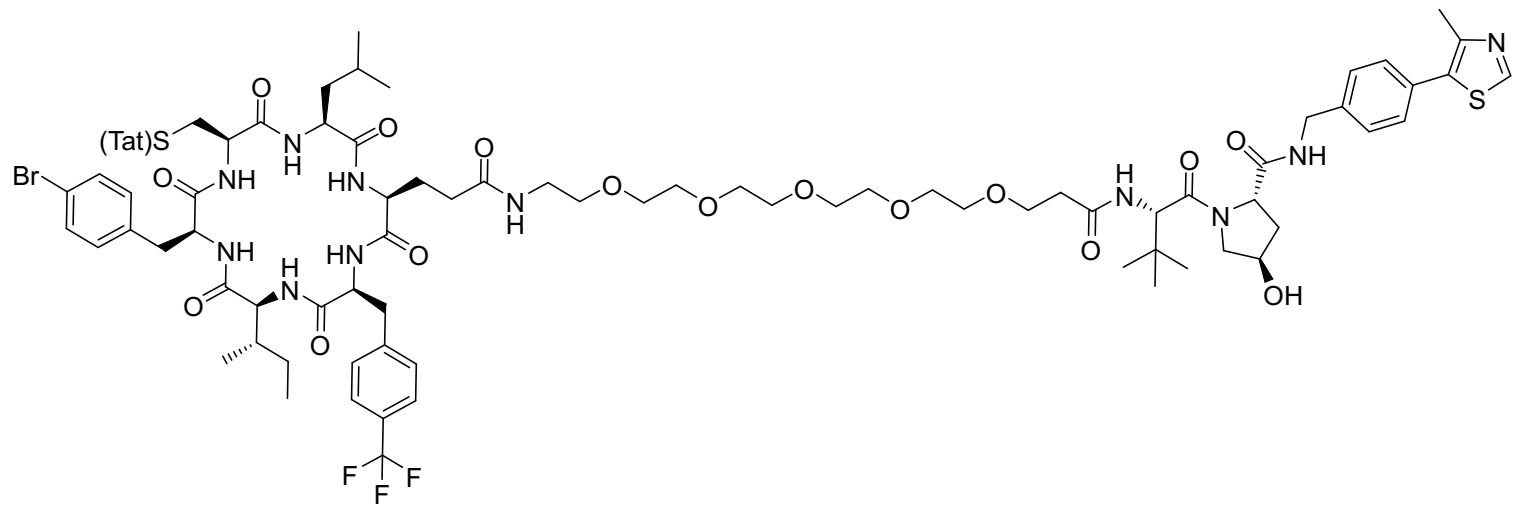

<u>*cyclo*-C(Tat)E(PEG$_6$)(VHL)LF(CF$_3$)IF(Br)</u> <u>(**70**)</u>

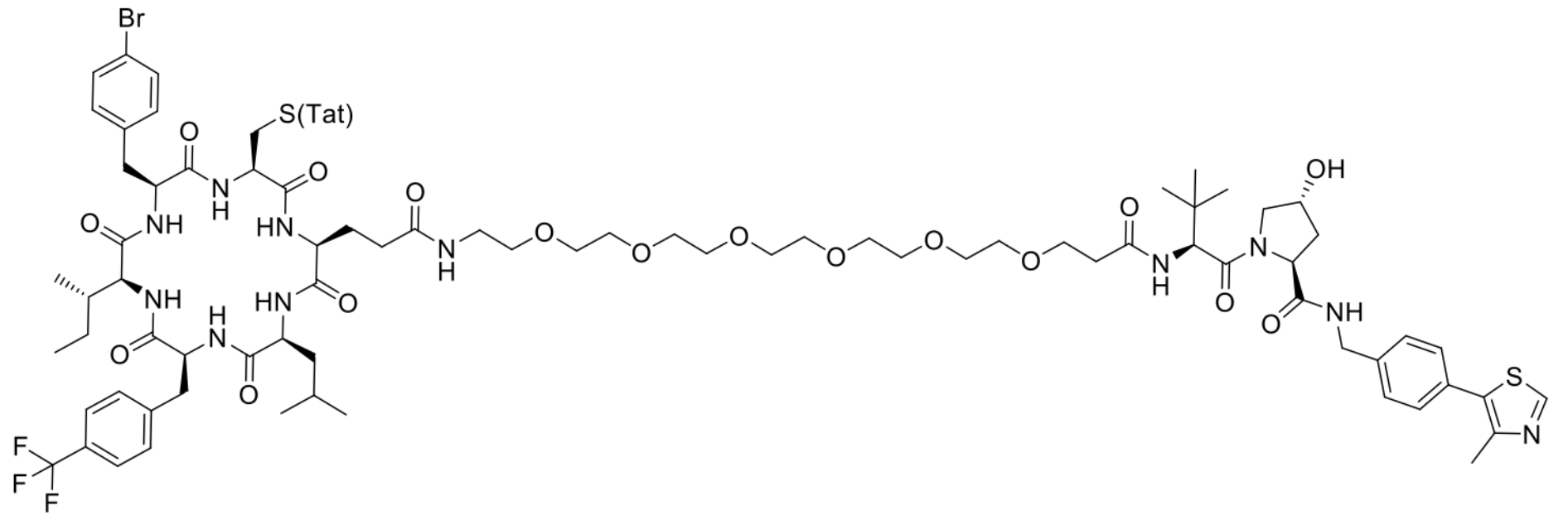

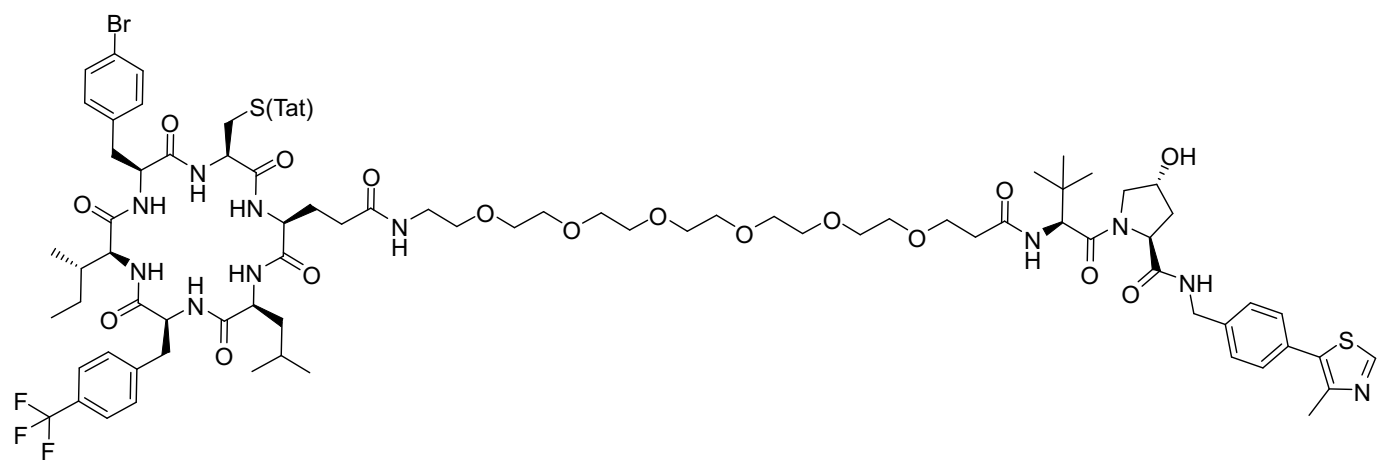

The linear peptide EC(StBu)F(Br)IF(CF$_3$)L was synthesised using 2-CTC resin SPPS at a 0.2 mmol scale. The linear peptide was then cyclised and subjected to TFA deprotection. The crude mixture was purified via reverse-phase flash chromatography to yield C(StBu)ELF(CF$_3$)IF(Br) as a white solid (70 mg, 0.071 mmol, 35%). The pure cyclic peptide (17.5 mg, 0.018 mmol) was dissolved in DMF (1 mL) to which was added DIPEA (18 µL, 0.054 mmol, 3 eq), HATU (8.3 mg, 0.022 mmol, 1.2 eq) and 4,7,10,13,16,19 Hexaoxaheneicosanoic acid, 21-amino-, 1,1-dimethylethyl ester (14.7 mgs, 0.036 mmol, 2 eq). The reaction was then stirred at ambient temperature and reaction progress was monitored by LC-MS. Once completed, the reaction crude was concentrated *in vacuo* and subjected to TFA deprotection. The TFA reaction mixture was then concentrated *in vacuo* and purified by reverse phase chromatography to give C(StBu)E(PEG$_6$)LF(CF$_3$)IF(Br) as a white solid (15 mg, 0.011 mmol, 61%). C(StBu)E(PEG$_6$)LF(CF$_3$)IF(Br) (15 mg, 0.011 mmol) was dissolved in DMF (1 mL) to which was added DIPEA (5.8 µL, 3 eq), HATU (6.4, 0.013 mmol, 1.2 eq) and VH032 ligand (6 mg, 0.014 mmol, 1.2 eq). The reaction was then stirred at ambient temperature and reaction progress monitored by LC-MS. Once completed, the reaction crude was purified by reverse phase chromatography to give C(StBu)E(PEG$_6$(VHL))LF(CF$_3$)IF(Br) as a white solid (8 mg, 0.0046 mmol, 42%). The StBu group was then removed and the crude purified via reverse-phase HPLC. Fractions were analysed via LC/MS, desired fractions were then pooled, concentrated *in vacuo,* and lyophilised to yield the cyclic peptide CE(PEG$_6$(VHL))LF(CF$_3$)IF(Br) as a white solid (3 mg, 0.0017 mmol, 37%). *Cyclo*-CE(PEG$_6$(VHL))LF(CF$_3$)IF(Br) (3 mg, 0.0017 mmol) was added to a solution of C(Spy)GRKKRRQRRRPPQ (7 mg, 0.0034 mmol, 2 eq) dissolved in DMF (2 mL) and stirred in an inert atmosphere overnight at ambient temperature. The mixture was then purified by reverse-phase HPLC. Fractions analysed via LC/MS, desired fractions were then pooled, concentrated *in vacuo*, and lyophilised to yield *cyclo*- C(Tat)E(PEG$_6$(VHL))LF(CF$_3$)IF(Br) as a white solid (5 mg, 0.0014 mmol, 82%).

LR MS (ESI+): m/z $[M+H]^+$ cald for $C_{149}H_{241}BrF_3N_{47}O_{34}S_3$, 3465.7, found; 868.2 $[M+4H]^{4+}$, 694.9 $[M+5H]^{5+}$, 579.3 $[M+6H]^{6+}$.

HRMS (ESI+): shows expected model isotope pattern.

Analytical HPLC: Rt: (220 nm) 12.306 min (92% Purity).

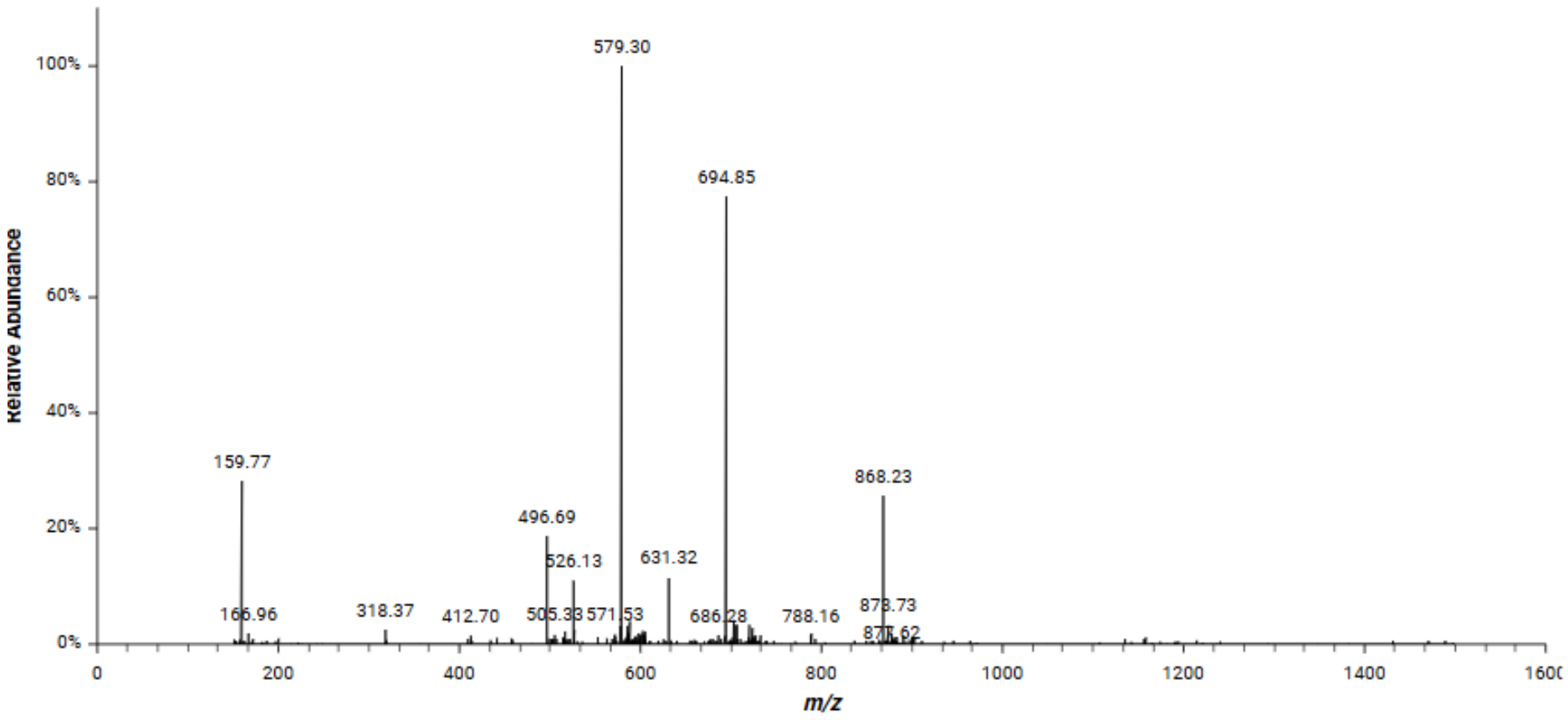

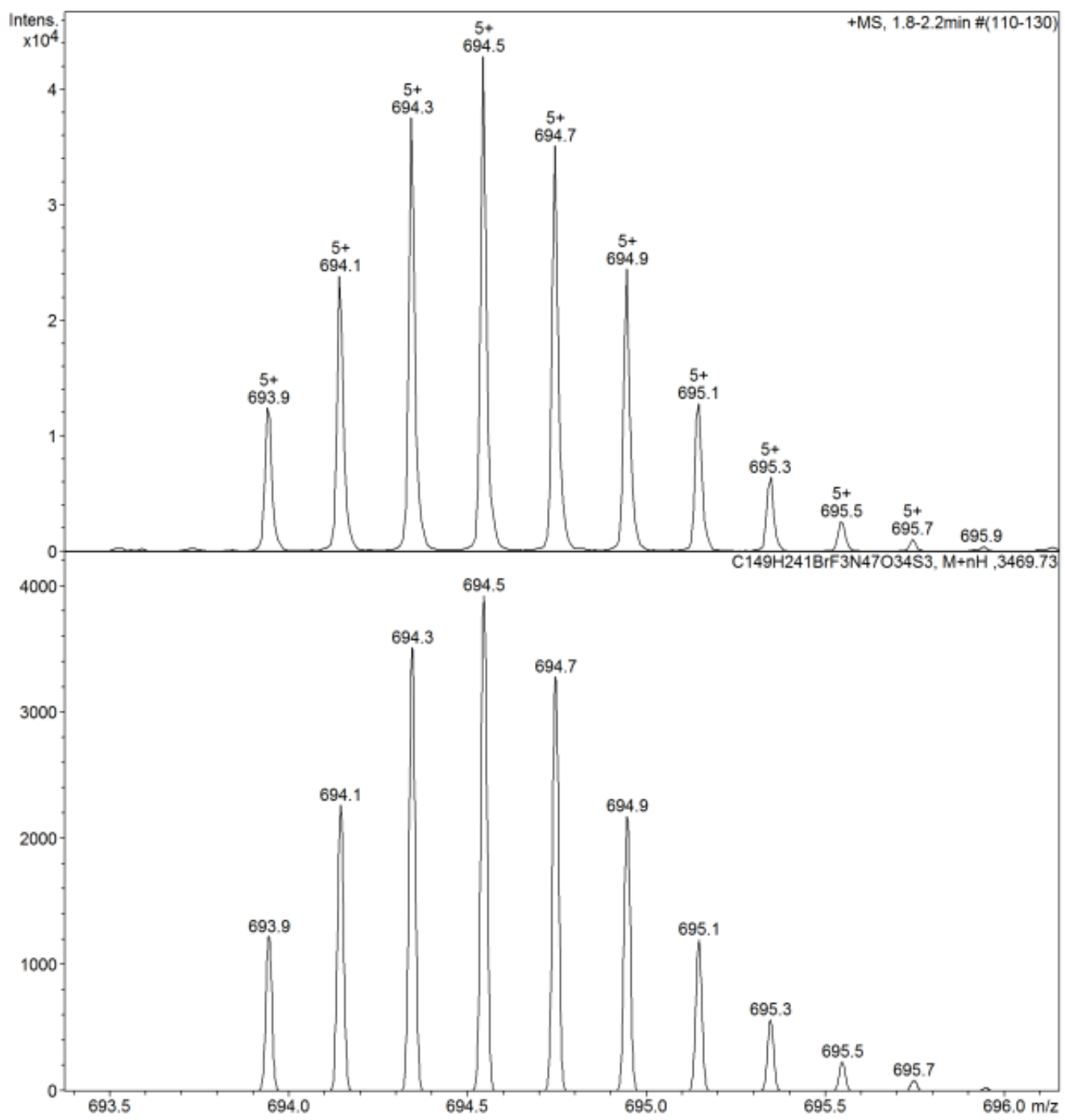

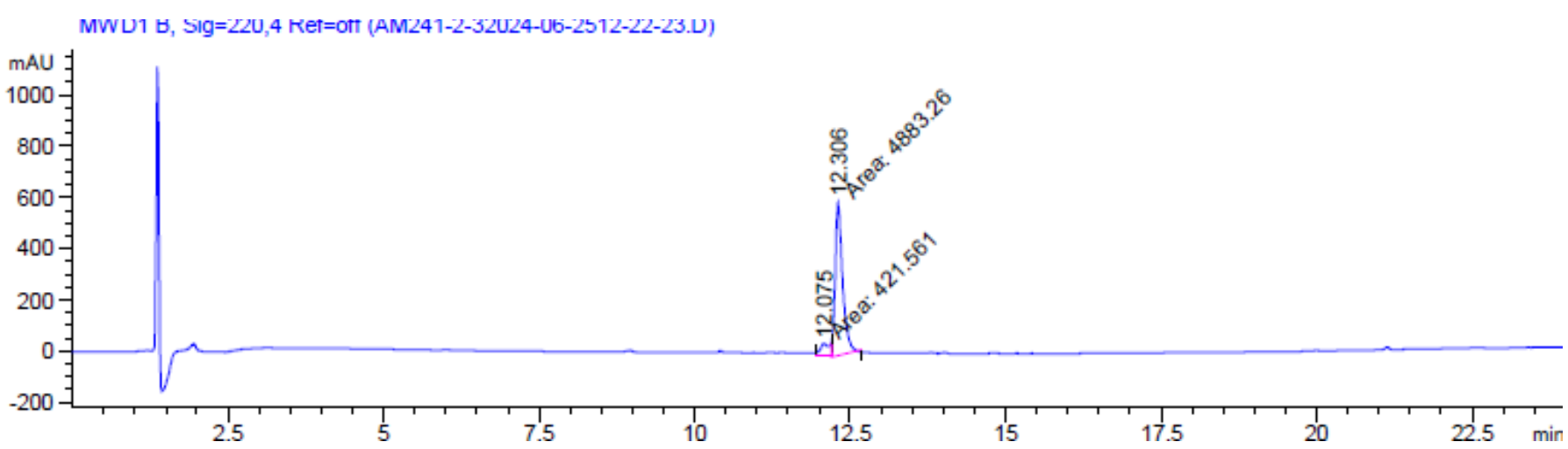

cyclo-C(Tat)E(PEG$_7$)(VHL)LF(CF$_3$)IF(Br) (**71**)

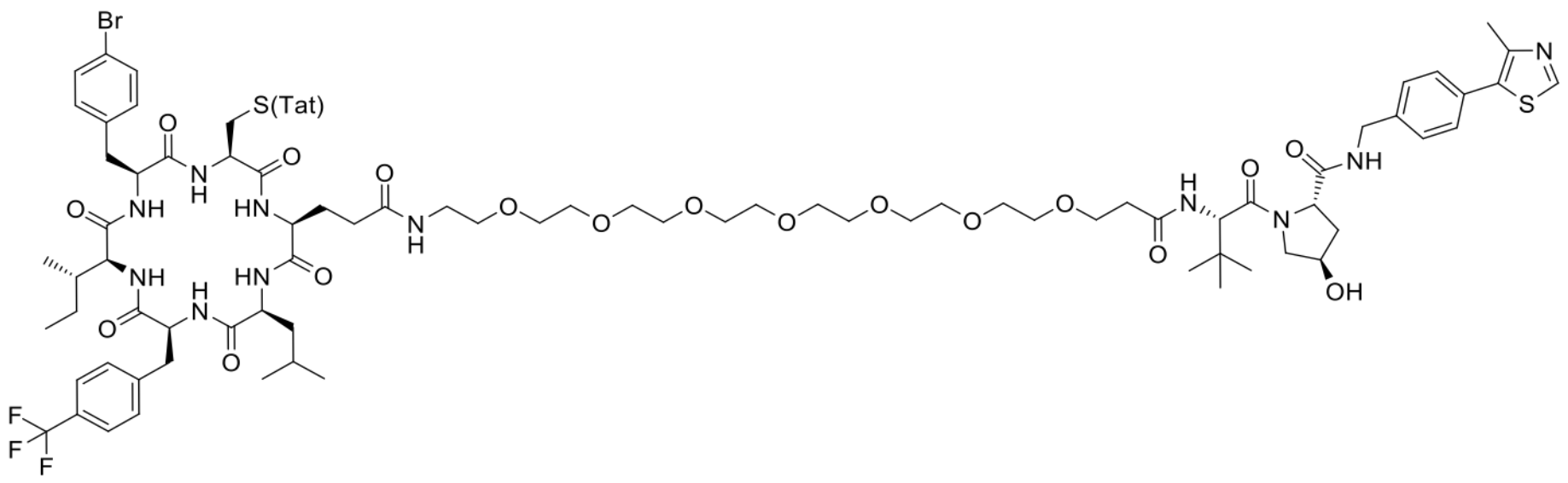

The linear peptide EC(StBu)F(CF$_3$)IF(Br)L was synthesised using 2-CTC resin SPPS at a 0.2 mmol scale. The linear peptide was then cyclised and subjected to TFA deprotection and concentrated *in vacuo*. The crude mixture was purified reverse-phase flash chromatography to yield C(StBu)ELF(CF$_3$)IF(Br) as a white solid (70 mg, 0.071 mmol, 35%). The pure cyclic peptide (17.5 mg, 0.018 mmol) was dissolved in DMF (1 mL) to which was added DIPEA (18 µL, 0.054 mmol, 3 eq), HATU (8.3 mg, 0.022 mmol, 1.2 eq) and tert-Butyl 1 amino-3,6,9,12,15,18,21 heptaoxatetracosan-24-oate (16.3 mgs, 0.036 mmol, 2 eq). The reaction was then stirred at ambient temperature and reaction progress was monitored by LC-MS. Once completed, the reaction crude was concentrated *in vacuo* and subjected to TFA deprotection. The TFA reaction mixture was then concentrated *in vacuo* and purified by reverse phase chromatography to give C(StBu)E(PEG$_7$)LF(CF$_3$)IF(Br) as a white solid (8 mg, 0.0067 mmol, 37%). C(StBu)E(PEG$_7$)LF(CF$_3$)IF(Br) (8 mg, 0.0067 mmol) was dissolved in DMF (1 mL) to which was added DIPEA (3 µL, 0.02 mmol, 3 eq), HATU (3 mg, 0.008 mmol, 1.2 eq) and VH032 ligand (3 mg, 0.008 mmol, 1.2 eq). The reaction was then stirred at ambient temperature and reaction progress monitored by LC-MS. Once completed, the reaction crude was purified by reverse phase chromatography to give C(StBu)E(PEG$_7$(VHL))LF(CF$_3$)IF(Br) as a white solid (4 mg, 0.0023 mmol, 34%). The StBu group was then removed and the crude purified via reverse-phase HPLC. Fractions were analysed via LC/MS, desired fractions were then pooled, concentrated *in vacuo,* and lyophilised to yield the cyclic peptide CE(PEG$_7$(VHL))LF(CF$_3$)IF(Br) as a white solid (2.5 mg, 0.0014 mmol, 61%). *cyclo*- CE(PEG$_7$(VHL))LF(CF$_3$)IF(Br) (2.5 mg, 0.0014 mmol) was added to a solution of C(Spy)GRKKRRQRRRPPQ (5.5 mg, 0.0028 mmol, 2 eq) dissolved in DMF (2 mL) and stirred in an inert atmosphere overnight at ambient temperature. The mixture was then purified by reverse-phase HPLC. Fractions analysed via LC/MS, desired fractions were then pooled, concentrated *in vacuo*, and lyophilised to yield *cyclo*- C(Tat)E(PEG$_7$(VHL))LF(CF$_3$)IF(Br) as a white solid (4.2 mg, 0.0012 mmol, 86%).

LR MS (ESI+): m/z $[M+H]^+$ cald for $C_{151}H_{245}BrF_3N_{47}O_{35}S_3$, 3509.71, found; 878.9 $[M+4H]^{4+}$, 703.7 $[M+5H]^{5+}$, 586.6 $[M+6H]^{6+}$.
HRMS (ESI+): shows expected model isotope pattern.
Analytical HPLC: Rt: (220 nm) 12.318 min (97% Purity).

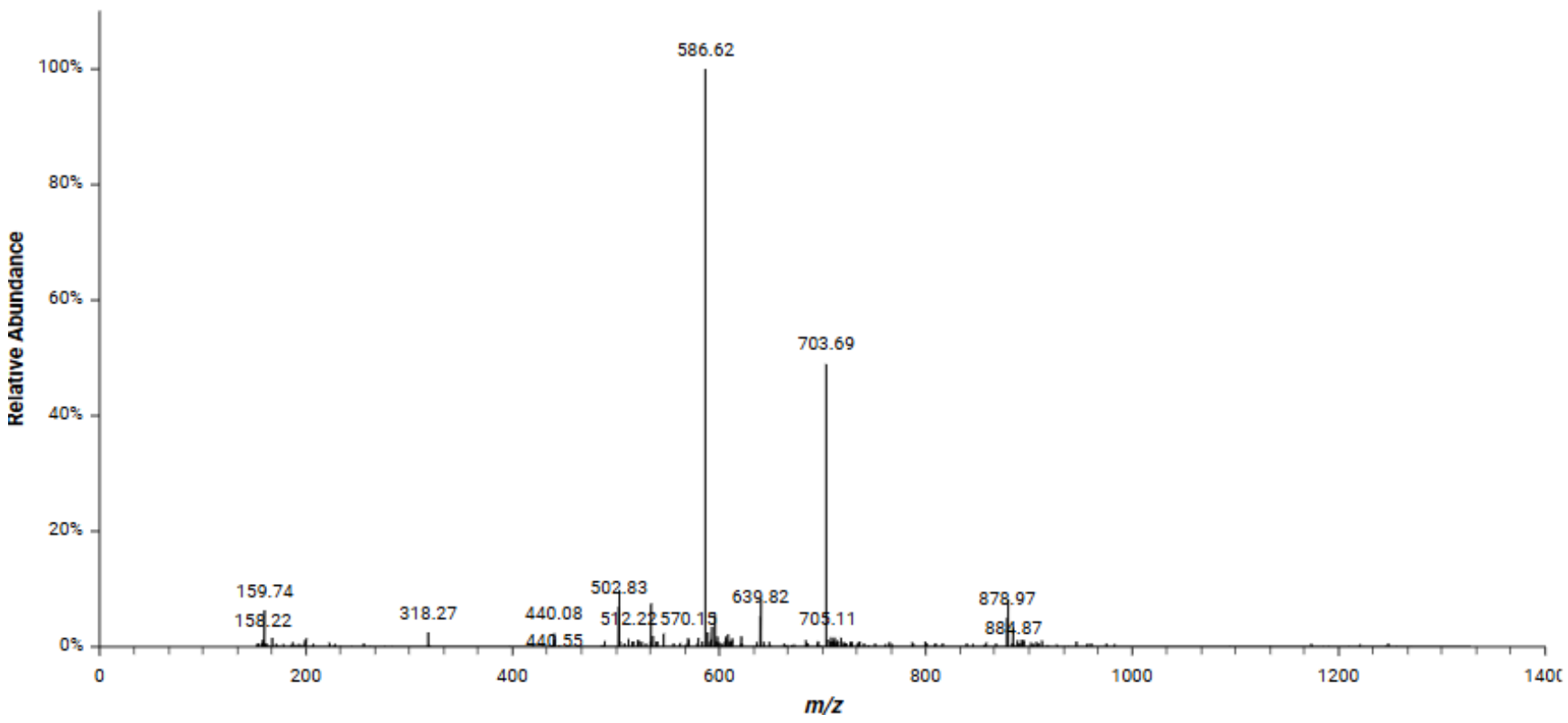
Relative Abundance
586.62
703.69
159.74
158.22
318.27
440.08
440.55
502.83
512.22
570.19
639.82
705.11
878.97
884.87
m/z

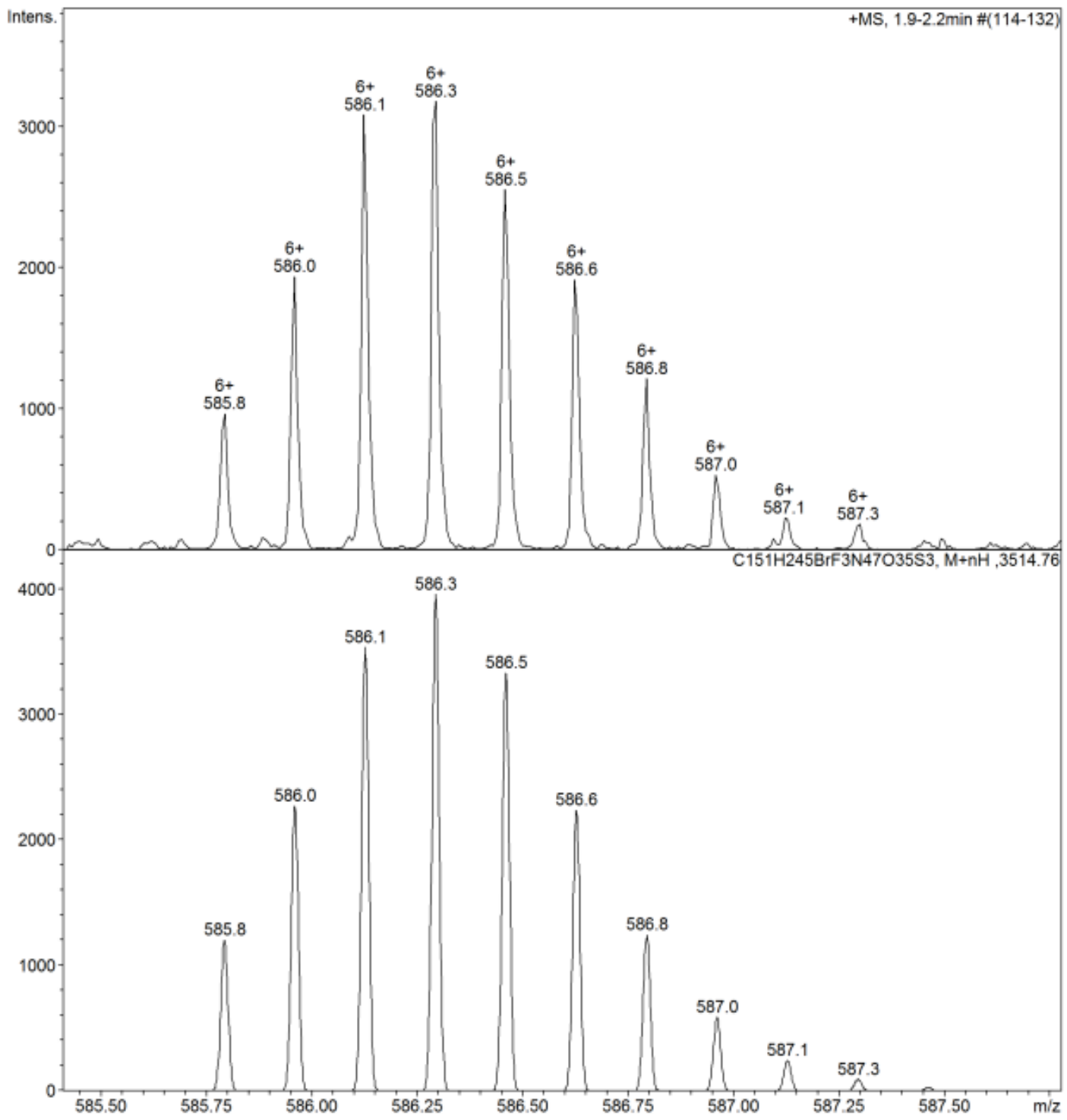
Intens.
+MS, 1.9-2.2min #(114-132)
6+ 585.8
6+ 586.0
6+ 586.1
6+ 586.3
6+ 586.5
6+ 586.6
6+ 586.8
6+ 587.0
6+ 587.1
6+ 587.3
C151H245BrF3N47O35S3, M+nH ,3514.76
585.8
586.0
586.1
586.3
586.5
586.6
586.8
587.0
587.1
587.3
m/z

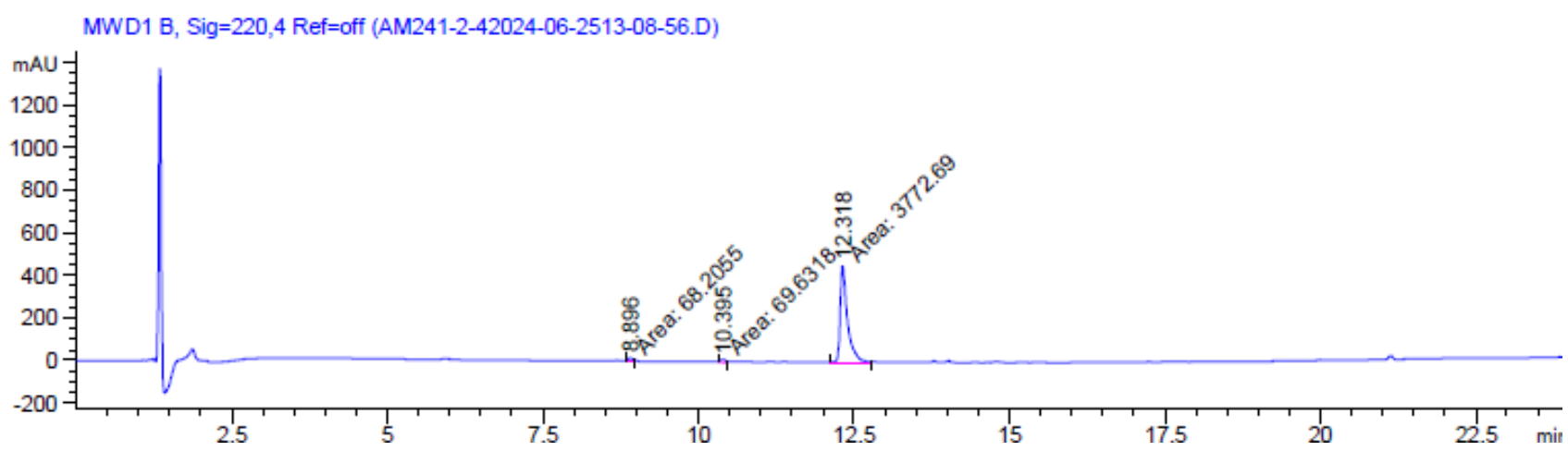
MWD1 B, Sig=220,4 Ref=off (AM241-2-42024-06-2513-08-56.D)
mAU
8.896
Area: 68.2055
10.395
Area: 69.6318
12.318
Area: 3772.69
min

cyclo-C(Tat)LE(PEG$_4$)(VHL)F(CF$_3$)IF(Br) **(72)**

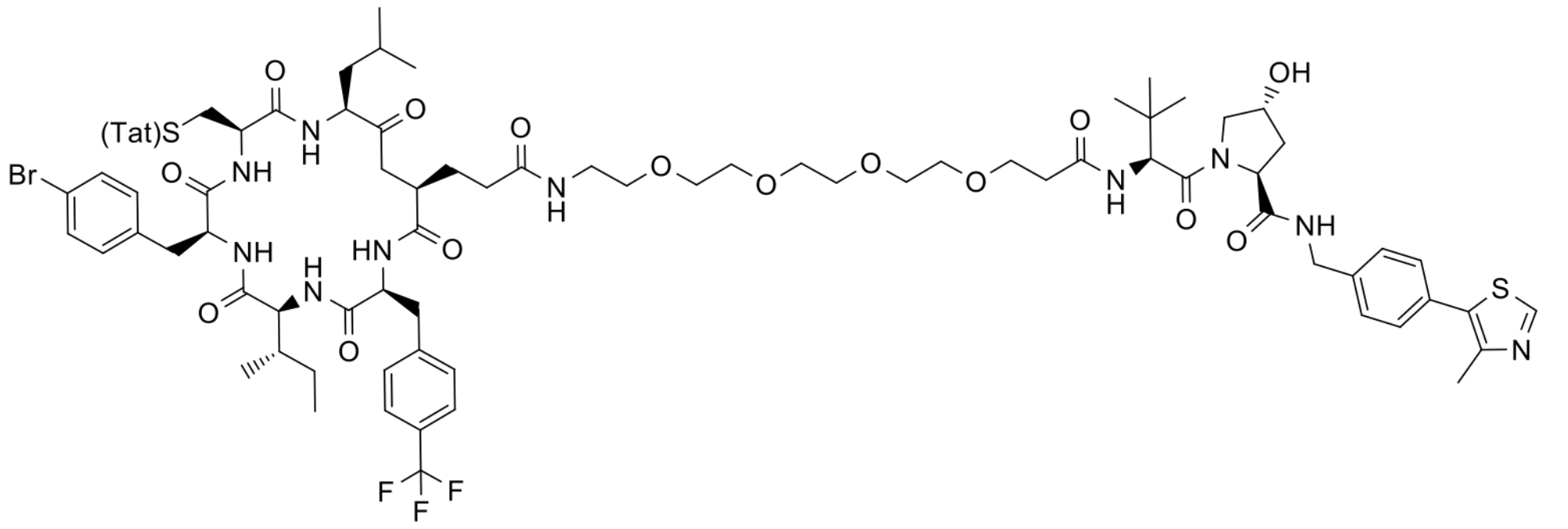

The linear peptide LC(StBu)F(Br)IF(CF$_3$)E was synthesised using 2-CTC resin SPPS at a 0.2 mmol scale. The linear peptide was then cyclised and subjected to TFA deprotection. The crude mixture was purified via reverse-phase flash chromatography to yield C(StBu)LEF(CF$_3$)IF(Br) as a white solid (53 mg, 0.053 mmol, 27%). The pure cyclic peptide (13.3 mg, 0.013 mmol) was dissolved in DMF (1 mL) to which was added DIPEA (6.6 μl, 0.04 mmol, 3 eq), HATU (6 mg, 0.016 mmol, 1.2 eq) and tert-butyl 1-amino-3,6,9,12-tetraoxapentadecan-15-oate (5 mgs, 0.016 mmol, 1.2 eq). The reaction was then stirred at ambient temperature and reaction progress was monitored by LC-MS. Once completed, the reaction crude was concentrated *in vacuo* and subjected to TFA deprotection. The TFA reaction mixture was then concentrated *in vacuo* and purified by reverse phase chromatography to give C(StBu)LE(PEG$_4$)F(CF$_3$)IF(Br) as a white solid (7.3 mg, 0.0059, 37%). C(StBu)LE(PEG$_4$)F(CF$_3$)IF(Br) (7.3 mg, 0.0059 mmol) was dissolved in DMF (1 mL) to which was added DIPEA (4 μl, 0.0177 mmol, 3 eq), HATU (2.7 mg, 0.007 mmol, 1.2 eq) and VH032 ligand (3 mg, 0.007 mmol, 1.2 eq). The reaction was then stirred at ambient temperature and reaction progress monitored by LC-MS. Once completed, the reaction crude was purified by reverse phase chromatography to give C(StBu)LE(PEG$_4$(VHL))F(CF$_3$)IF(Br) as a white solid (5.6 mg, 0.0034 mmol, 58%). The StBu group was then cleaved and the crude purified via reverse-phase HPLC. Fractions were analysed via LC/MS, desired fractions were then pooled, concentrated *in vacuo,* and lyophilised to yield the cyclic peptide CLE(PEG$_4$(VHL))F(CF$_3$)IF(Br) as a white solid. *cyclo*- CLE(PEG$_4$(VHL))F(CF$_3$)IF(Br) (2.5 mg, 0.0016 mmol, 47%) was added to a solution of C(Spy)GRKKRRQRRRPPQ (6.2 mg, 0.0032 mmol, 2 eq) dissolved in DMF (2 mL) and stirred in an inert atmosphere overnight at ambient temperature. The mixture was then purified by reverse-phase HPLC. Fractions analysed via LC/MS, desired fractions were then pooled, concentrated *in vacuo*, and lyophilised to yield *cyclo*-C(Tat)LE(PEG$_4$(VHL))F(CF$_3$)IF(Br) as a white solid (2 mg, 0.0006 mmol, 38%).

LR MS (ESI+): m/z $[M+H]^+$ cald for $C_{146}H_{234}BrF_3N_{46}O_{32}S_3$, 3376.64, found; 676.9 $[M+5H]^{5+}$, 564.5 $[M+6H]^{6+}$.
HRMS (ESI+): shows expected model isotope pattern.
Analytical HPLC: Rt: (220 nm) 12.290 min (97% Purity).

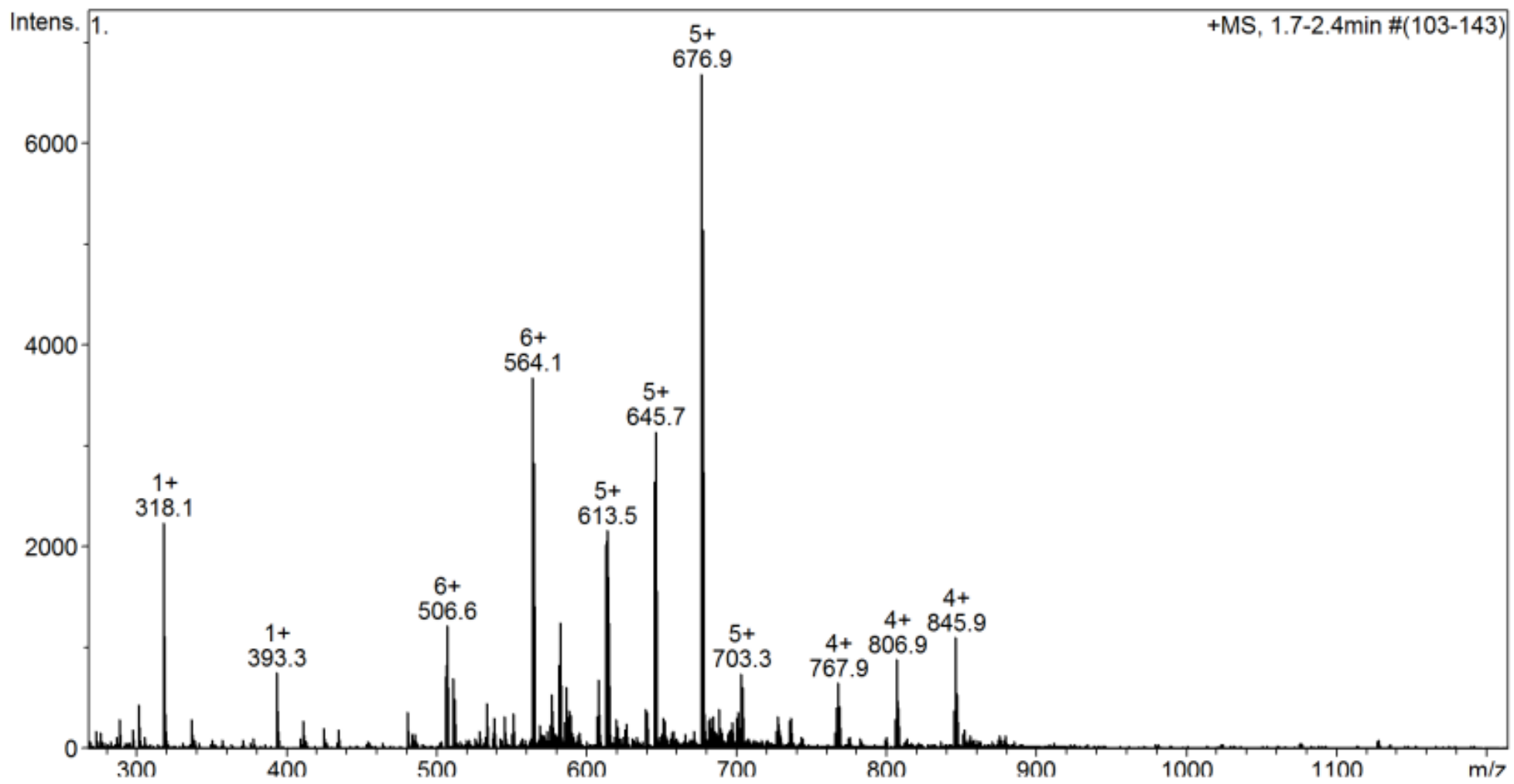
Intens.
1.
+MS, 1.7-2.4min #(103-143)
5+
676.9
6+
564.1
5+
645.7
5+
613.5
1+
318.1
1+
393.3
6+
506.6
5+
703.3
4+
767.9
4+
806.9
4+
845.9
m/z

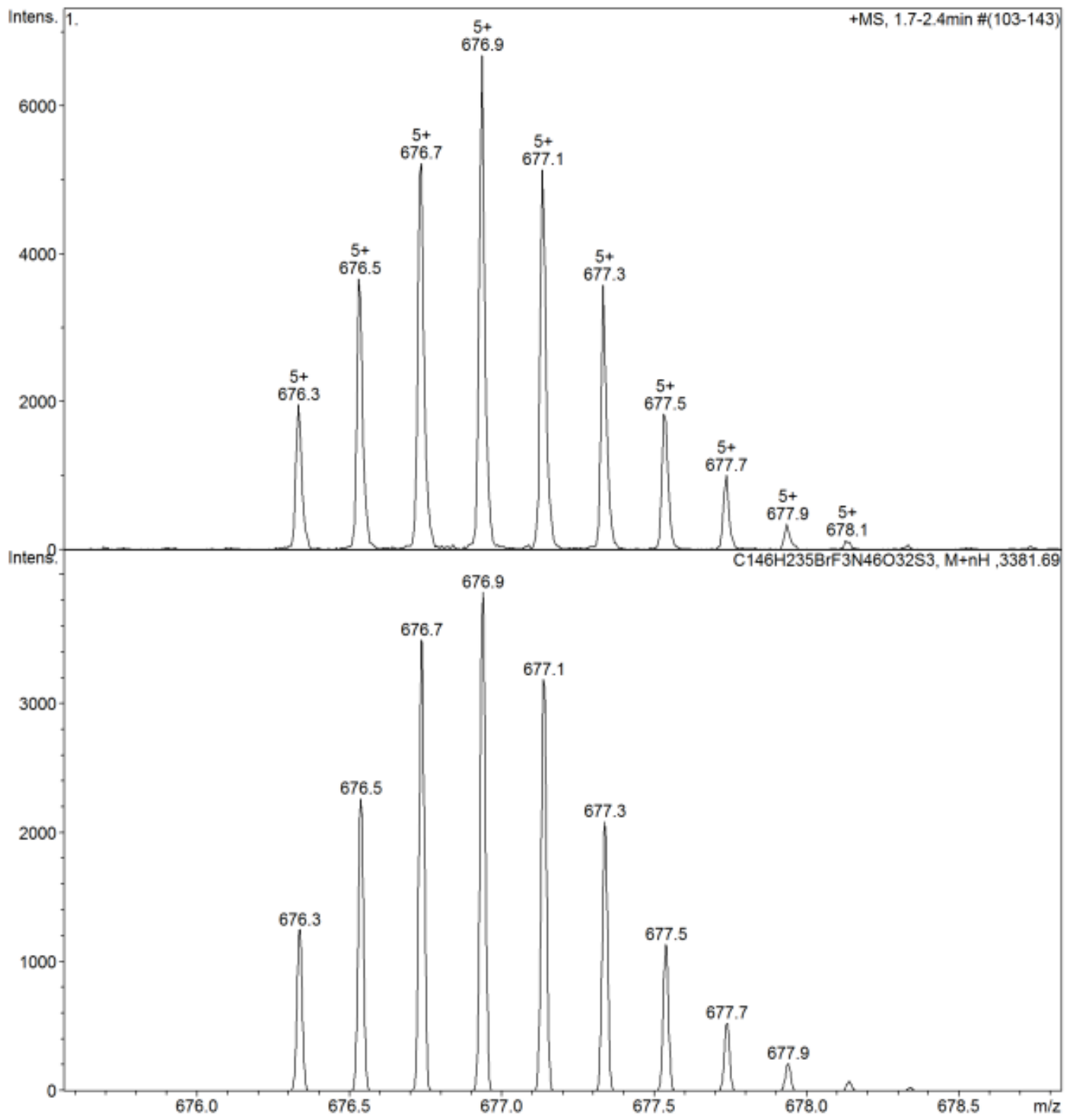
Intens.
1.
+MS, 1.7-2.4min #(103-143)
5+
676.3
5+
676.5
5+
676.7
5+
676.9
5+
677.1
5+
677.3
5+
677.5
5+
677.7
5+
677.9
5+
678.1
Intens.
C146H235BrF3N46O32S3, M+nH ,3381.69
676.3
676.5
676.7
676.9
677.1
677.3
677.5
677.7
677.9
m/z

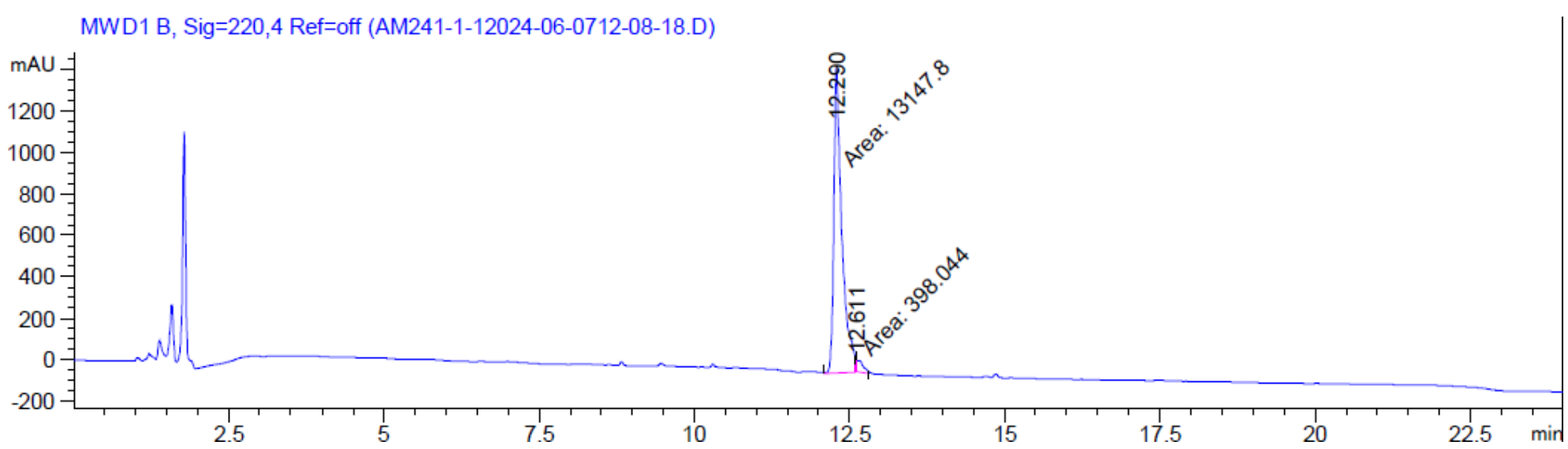
MWD1 B, Sig=220,4 Ref=off (AM241-1-12024-06-0712-08-18.D)
mAU
12.290
Area: 13147.8
12.611
Area: 398.044
min

cyclo-C(Tat)LE(PEG$_5$)(VHL)F(CF$_3$)IF(Br) (**73**)

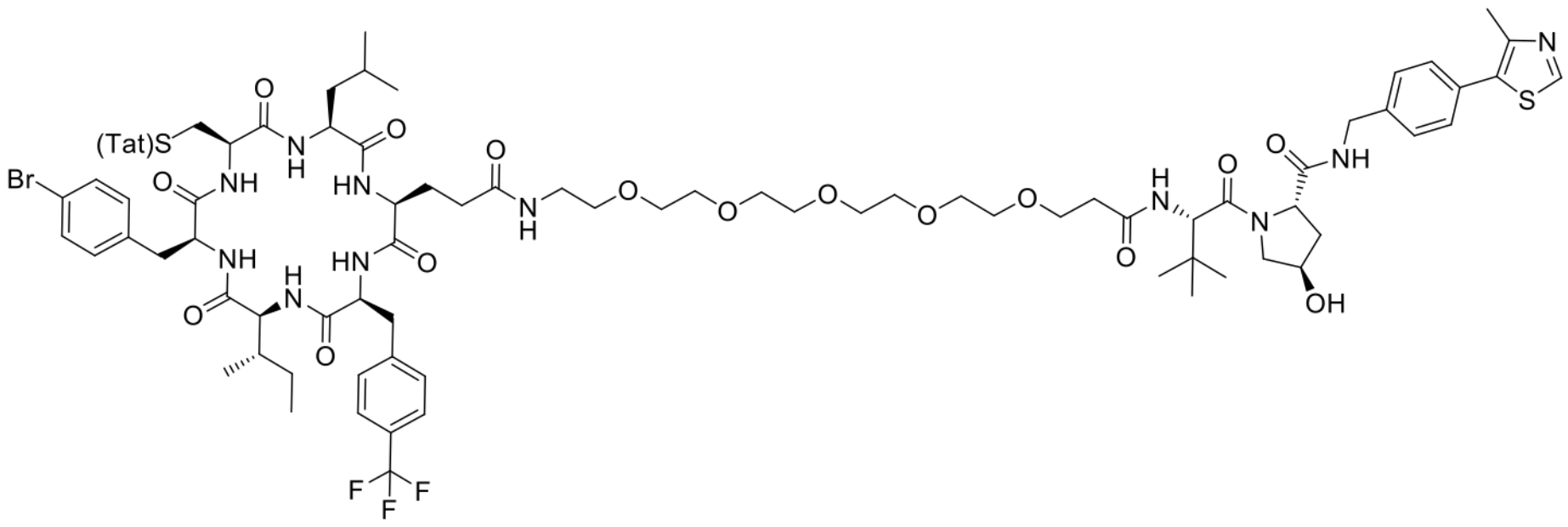

The linear peptide LC(StBu)F(Br)IF(CF$_3$)E was synthesised using 2-CTC resin SPPS at a 0.2 mmol scale. The linear peptide was then cyclised and subjected to TFA deprotection. The crude mixture was purified via reverse-phase flash chromatography to yield C(StBu)LEF(CF$_3$)IF(Br) as a white solid (53 mg, 0.053 mmol, 27%). The pure cyclic peptide (13.3 mg, 0.013 mmol) was dissolved in DMF (1 mL) to which was added DIPEA (6.6 µL, 0.04 mmol, 3 eq), HATU (6 mg, 0.015 mmol, 1.2 eq) and *tert*-Butyl-1-amino-3,6,9,12,15-pentaoxaoctadecan-18-oate (5.7 mg, 0.016 mmol, 1.2 eq). The reaction was then stirred at ambient temperature and reaction progress was monitored by LC-MS. Once completed, the reaction crude was concentrated *in vacuo* and subjected to TFA deprotection. The TFA reaction mixture was then concentrated *in vacuo* and purified by reverse phase chromatography to give C(StBu)LE(PEG$_5$)F(CF$_3$)IF(Br) as a white solid (4 mg, 0.003 mmol, 23%). C(StBu)LE(PEG$_5$)F(CF$_3$)IF(Br) (4 mg, 0.003 mmol) was dissolved in DMF (1 mL) to which was added DIPEA (2 µl, 0.009 mmol, 3 eq), HATU (1.4 mg, 0.004 mmol, 1.2 eq) and VH032 ligand (1.5 mg, 0.0036 mmol, 1.2 eq). The reaction was then stirred at ambient temperature and reaction progress monitored by LC-MS. Once completed, the reaction crude was purified by reverse phase chromatography to give C(StBu)LE(PEG5(VHL))F(CF$_3$)IF(Br) as a white solid (3.3 mg, 0.002 mmol, 67%). The StBu group was then and the crude purified via reverse-phase HPLC. Fractions were analysed via LC/MS, desired fractions were then pooled, concentrated *in vacuo,* and lyophilised to yield the cyclic peptide CLE(PEG$_5$(VHL))F(CF$_3$)IF(Br) as a white solid. *cyclo*- CLE(PEG$_5$(VHL))F(CF$_3$)IF(Br) (1.6 mg, 0.001 mmol, 50%) was added to a solution of C(Spy)GRKKRRQRRRPPQ (4 mg, 0.002 mmol) dissolved in DMF (2 mL) and stirred in an inert atmosphere overnight at ambient temperature. The mixture was then purified by reverse-phase HPLC. Fractions analysed via LC/MS, desired fractions were then pooled, concentrated *in vacuo*, and lyophilised to yield *cyclo*- C(Tat)LE(PEG$_5$(VHL))F(CF$_3$)IF(Br) as a white solid (2.4 mg, 0.0007 mmol, 70%).

LR MS (ESI+): m/z $[M+H]^+$ cald for $C_{147}H_{237}BrF_3N_{47}O_{33}S_3$, 3421.7, found; 685.7 $[M+5H]^{5+}$, 572.04 $[M+6H]^{6+}$.
HRMS (ESI+): shows expected model isotope pattern.
Analytical HPLC: Rt: (220 nm) 12.401 min (91% Purity).

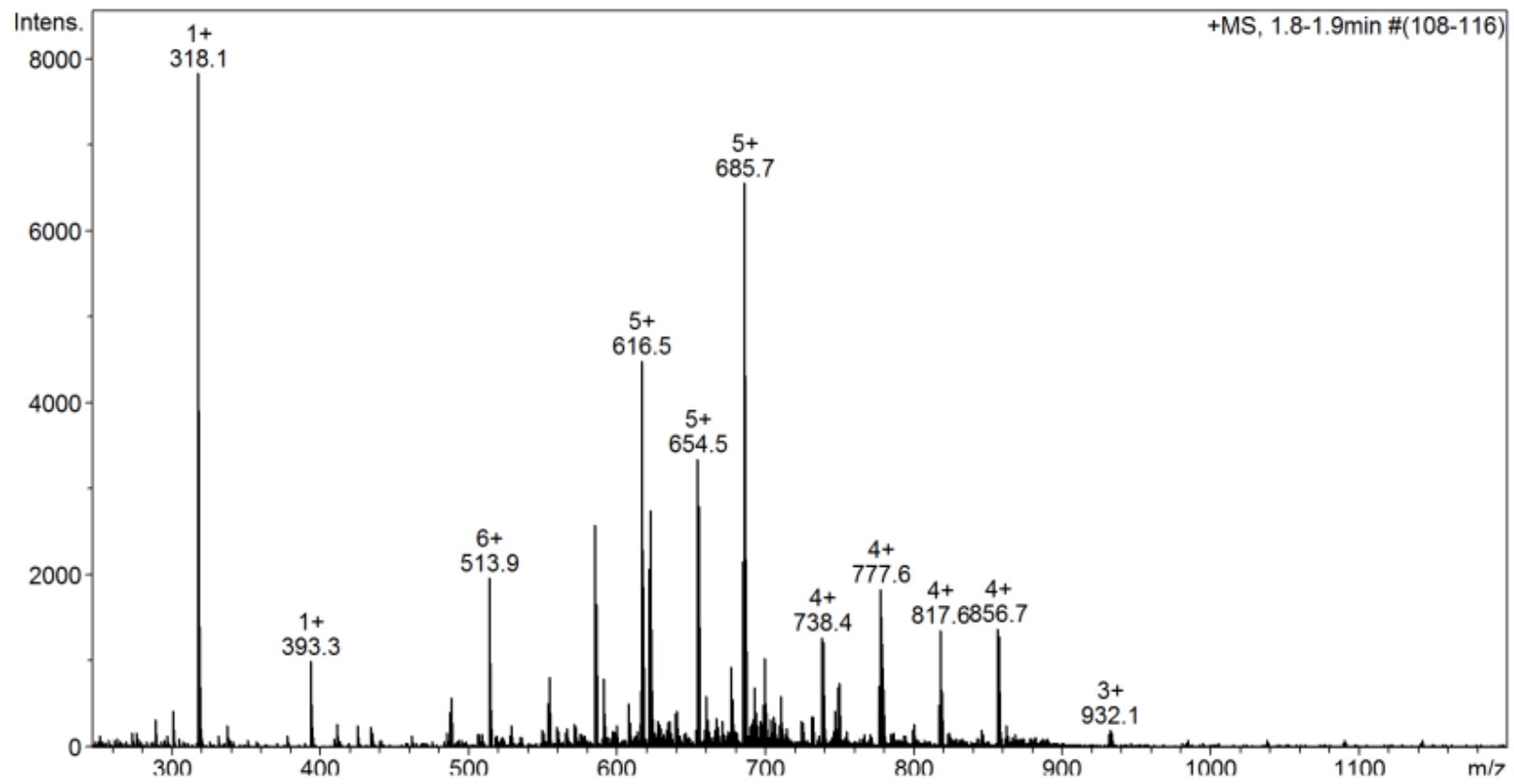

Intens.
+MS, 1.8-1.9min #(108-116)
1+ 318.1
5+ 685.7
5+ 616.5
5+ 654.5
6+ 513.9
1+ 393.3
4+ 777.6
4+ 738.4
4+ 817.6
4+ 856.7
3+ 932.1
m/z

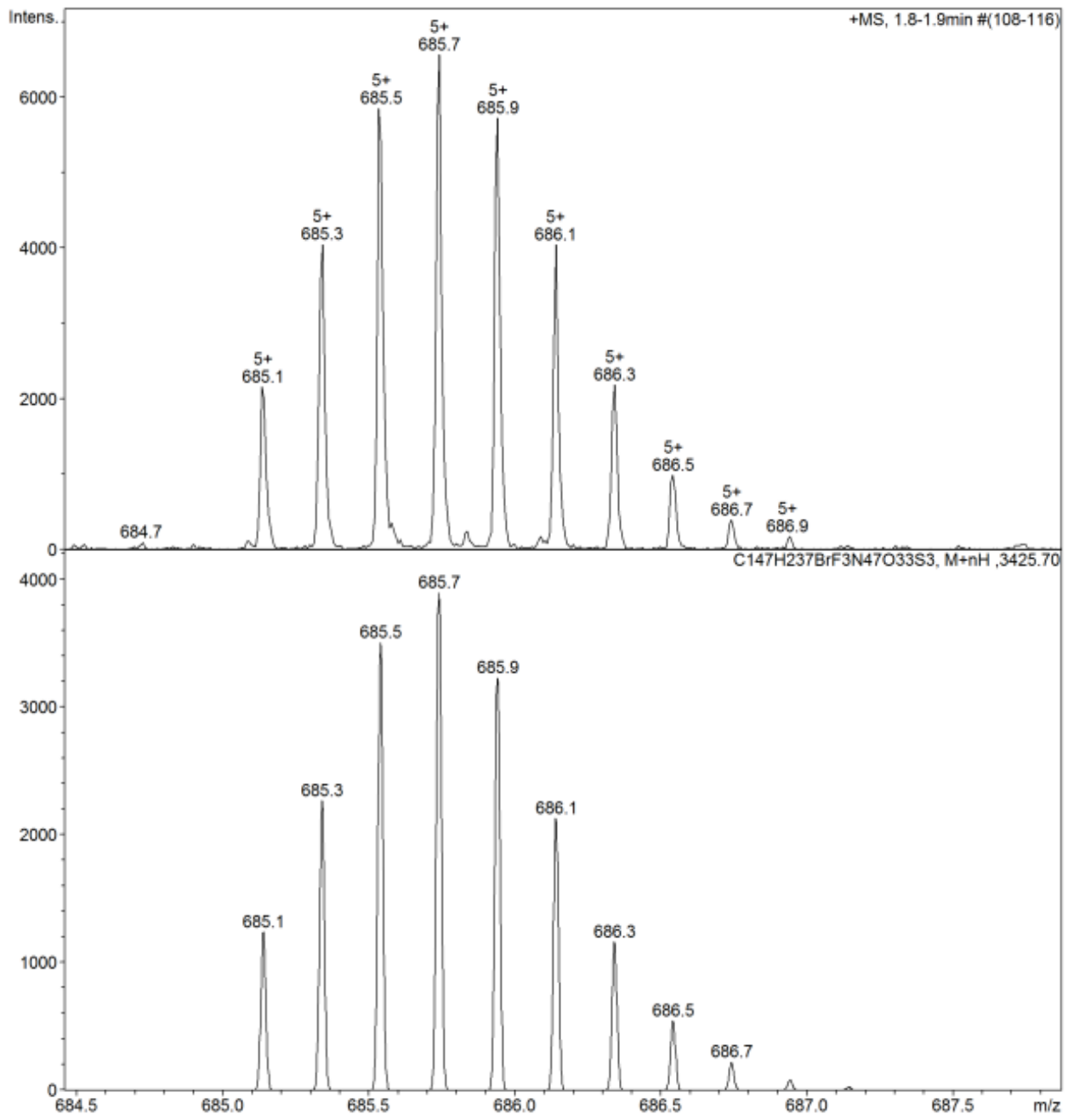

Intens.
+MS, 1.8-1.9min #(108-116)
C147H237BrF3N47O33S3, M+nH ,3425.70
m/z

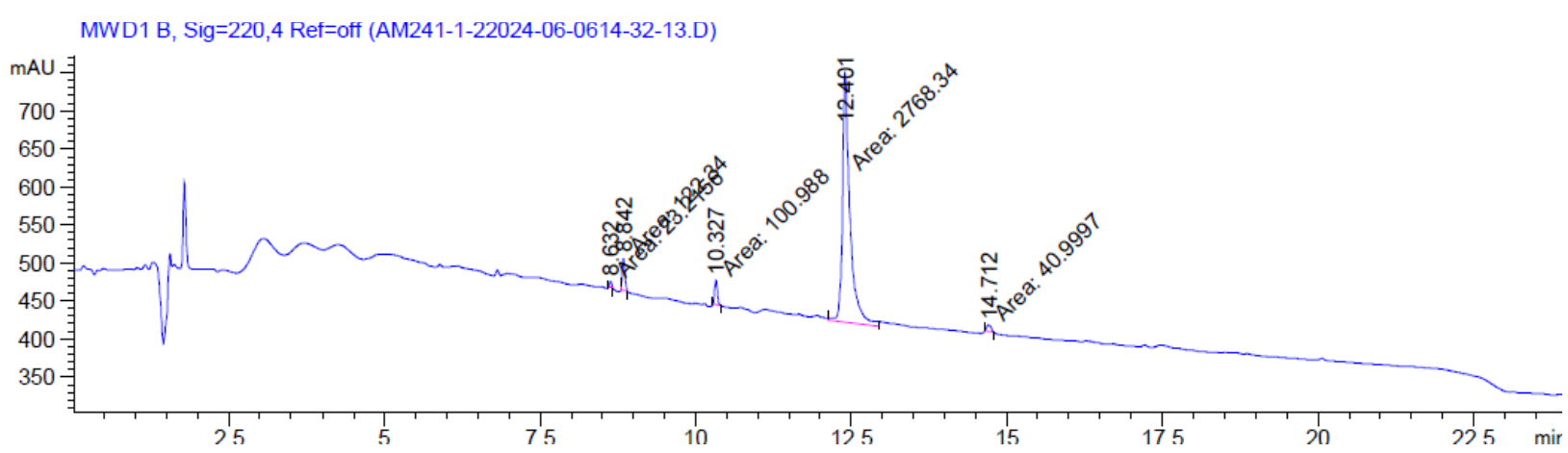

MWD1 B, Sig=220,4 Ref=off (AM241-1-22024-06-0614-32-13.D)
mAU
12.401
Area: 2768.34
10.327
Area: 100.988
14.712
Area: 40.9997
min

<u>*cyclo*-C(Tat)LE(PEG_6)(VHL)F(CF_3)IF(Br) (**74**)</u>

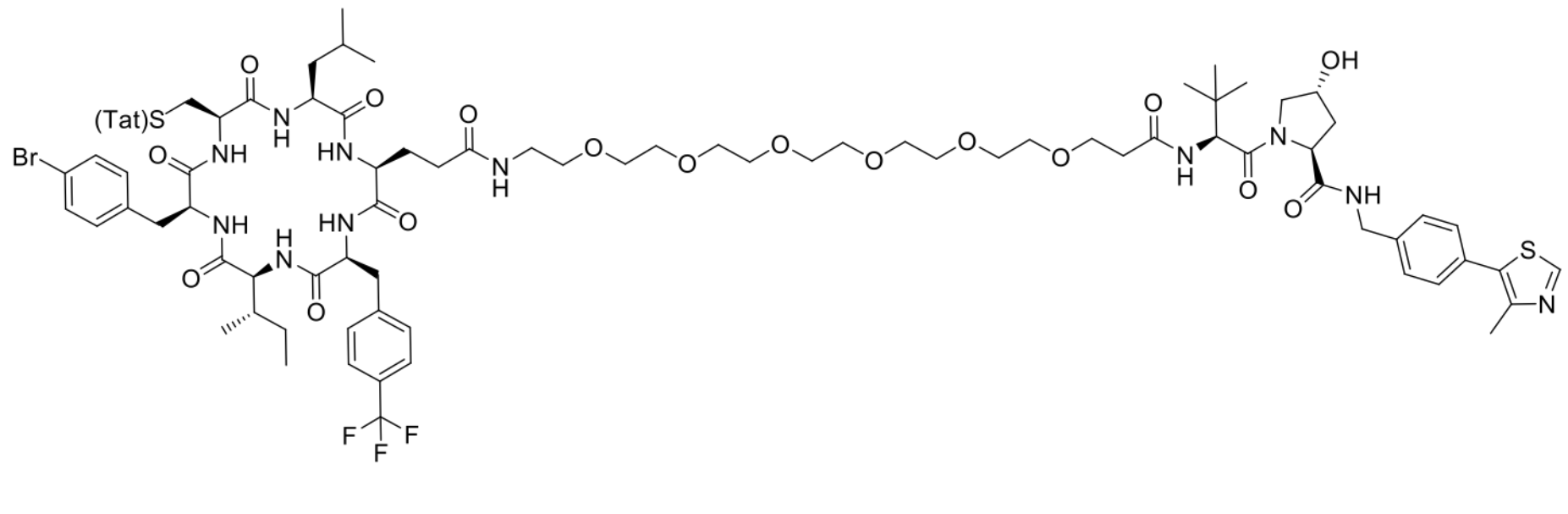

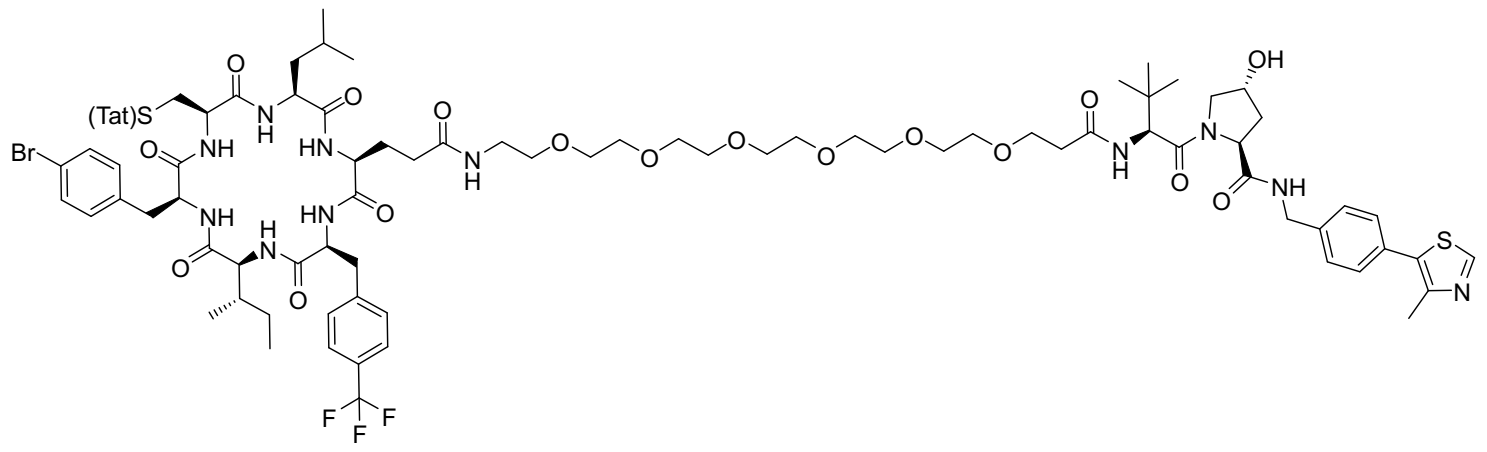

The linear peptide LC(StBu)F(Br)IF(CF_3)E was synthesised using 2-CTC resin SPPS at a 0.2 mmol scale. The linear peptide was then cyclised and subjected to TFA deprotection. The crude mixture was purified via reverse-phase flash chromatography to yield C(StBu)LEF(CF_3)IF(Br) as a white solid (53 mg, 0.053 mmol, 27%). The pure cyclic peptide (13.3 mg, 0.013 mmol) was dissolved in DMF (1 mL) to which was added DIPEA (6.6 µL, 0.039 mmol, 3 eq), HATU (6 mg, 0.016 mmol, 1.2 eq) and 4,7,10,13,16,19 Hexaoxaheneicosanoic acid, 21-amino-, 1,1-dimethylethyl ester (6.4 mgs, 0.016 mmol, 1.2 eq). The reaction was then stirred at ambient temperature and reaction progress was monitored by LC-MS. Once completed, the reaction crude was concentrated *in vacuo* and subjected to TFA deprotection. The TFA reaction mixture was then concentrated *in vacuo* and purified by reverse phase chromatography to give C(StBu)LE(PEG_6)F(CF_3)IF(Br) as a white solid (5.5 mg, 0.004 mmol, 31%). C(StBu)LE(PEG_6)F(CF_3)IF(Br) (5.5 mg, 0.004 mmol) was dissolved in DMF (1 mL) to which was added DIPEA (2.4 µL, 0.012 mmol, 3 eq), HATU (1.8 mg, 0.005 mmol, 1.2 eq) and VH032 ligand (2 mg, 0.005 mmol, 1.2 eq). The reaction was then stirred at ambient temperature and reaction progress monitored by LC-MS. Once completed, the reaction crude was purified by reverse phase chromatography to give C(StBu)LE(PEG_6(VHL))F(CF_3)IF(Br) as a white solid (5.6 mg, 0.0032 mmol, 80%). The StBu group was then removed and the crude purified via reverse-phase HPLC. Fractions were analysed via LC/MS, desired fractions were then pooled, concentrated *in vacuo,* and lyophilised to yield the cyclic peptide CLE(PEG_6(VHL))F(CF_3)IF(Br) (2.2 mg, 0.0013 mmol, 41%) as a white solid. *cyclo*- CLE(PEG_6(VHL))F(CF_3)IF(Br) (2.2 mg, 0.0013 mmol) was added to a solution of C(Spy)GRKKRRQRRRPPQ (5.2 mg, 0.0026 mmol) dissolved in DMF (2 mL) and stirred in an inert atmosphere overnight at ambient temperature. The mixture was then purified by reverse-phase HPLC. Fractions analysed via LC/MS, desired fractions were then pooled, concentrated *in vacuo*, and lyophilised to yield *cyclo*-C(Tat)LE(PEG_6(VHL))F(CF_3)IF(Br) as a white solid (2 mg, 0.0058 mmol, 44%).

LR MS (ESI+): m/z $[M+H]^+$ cald for $C_{149}H_{241}BrF_3N_{47}O_{34}S_3$, 3465.7, found; 694.5 $[M+5H]^{5+}$, 579.13 $[M+6H]^{6+}$.
HRMS (ESI+): shows expected model isotope pattern.
Analytical HPLC: Rt: (220 nm) 12.280 min (100% Purity).

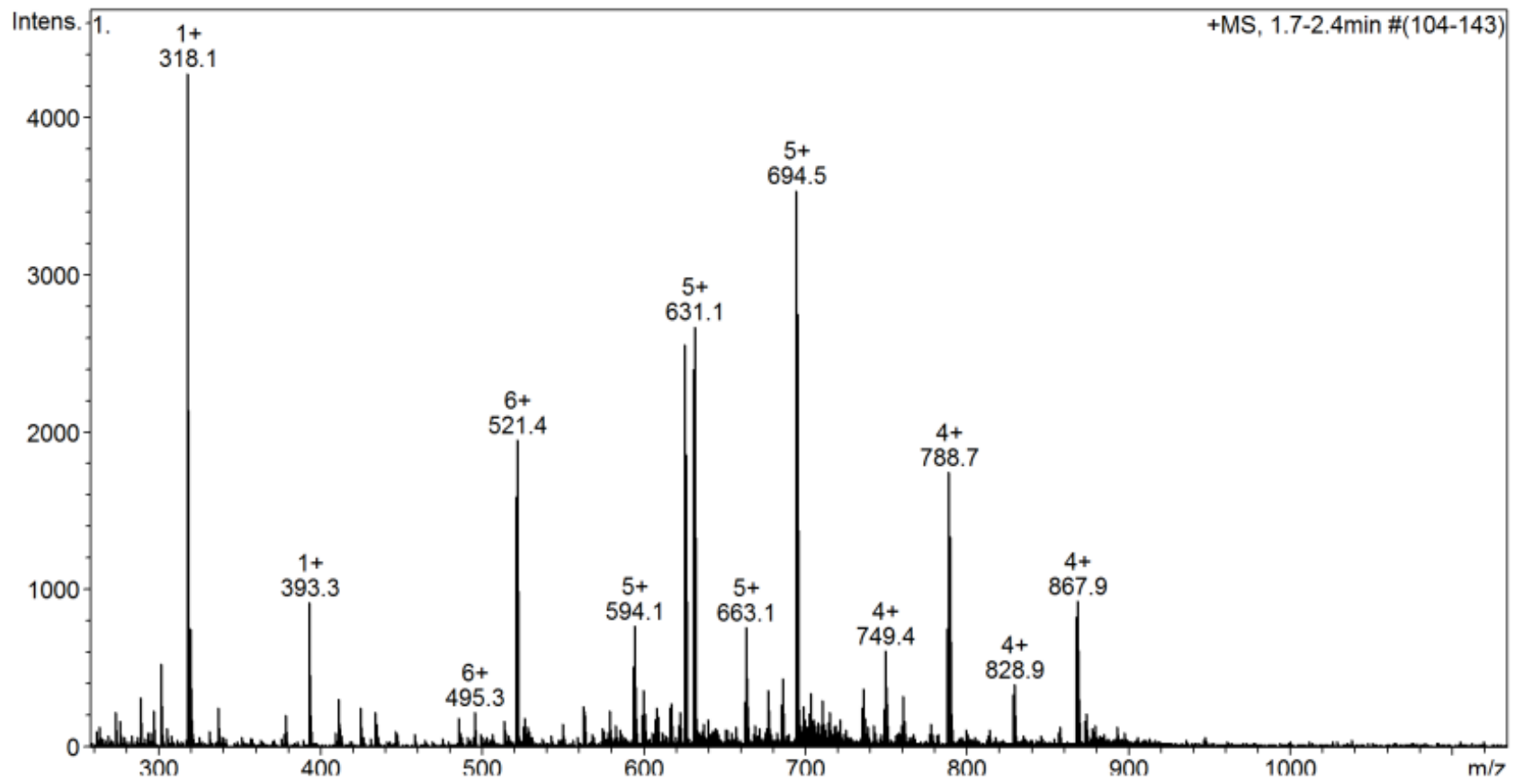

Intens.
+MS, 1.7-2.4min #(104-143)
1+ 318.1
1+ 393.3
6+ 495.3
6+ 521.4
5+ 594.1
5+ 631.1
5+ 663.1
5+ 694.5
4+ 749.4
4+ 788.7
4+ 828.9
4+ 867.9
m/z

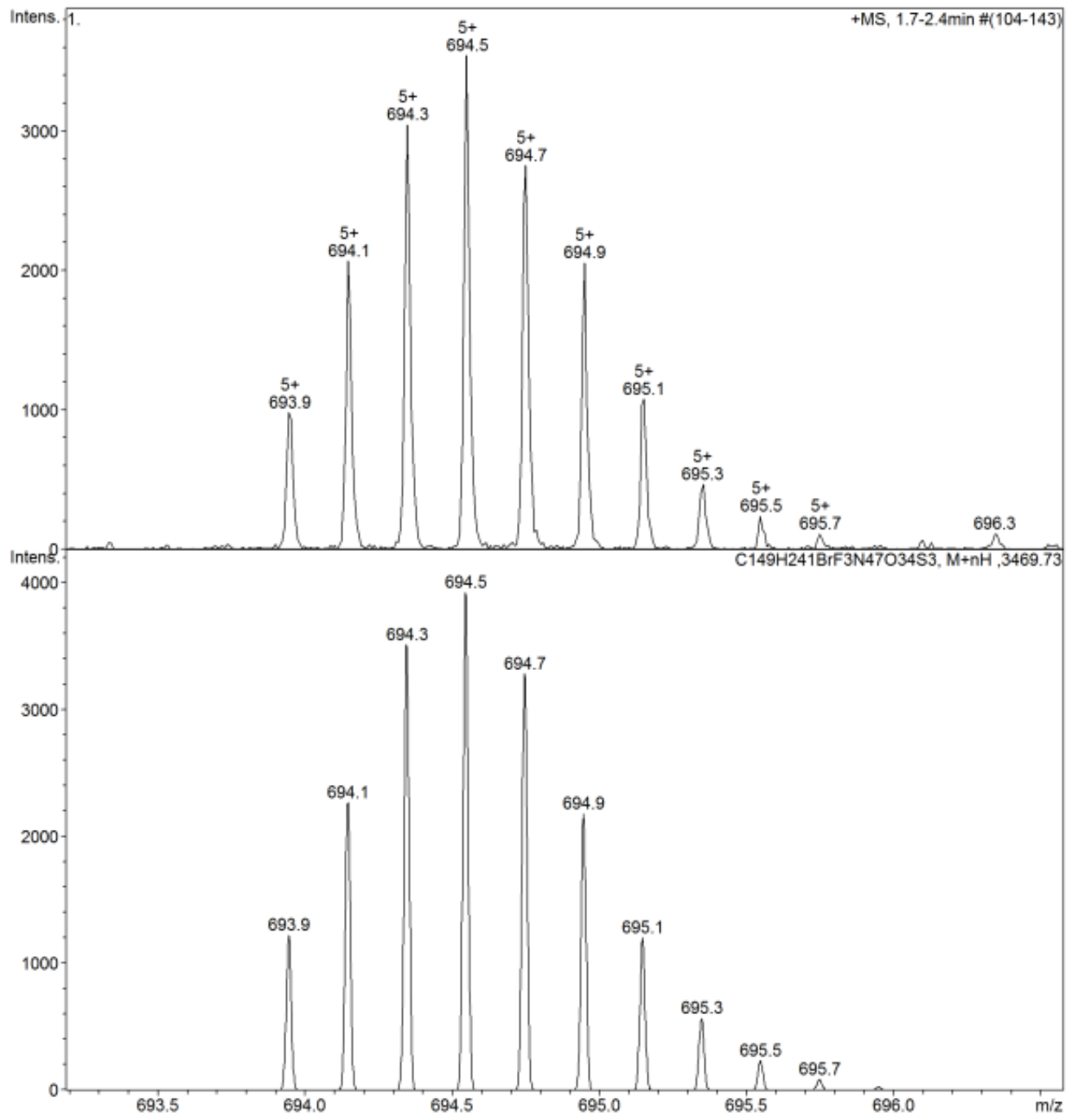

Intens.
+MS, 1.7-2.4min #(104-143)
5+ 693.9
5+ 694.1
5+ 694.3
5+ 694.5
5+ 694.7
5+ 694.9
5+ 695.1
5+ 695.3
5+ 695.5
5+ 695.7
696.3
C149H241BrF3N47O34S3, M+nH ,3469.73
m/z

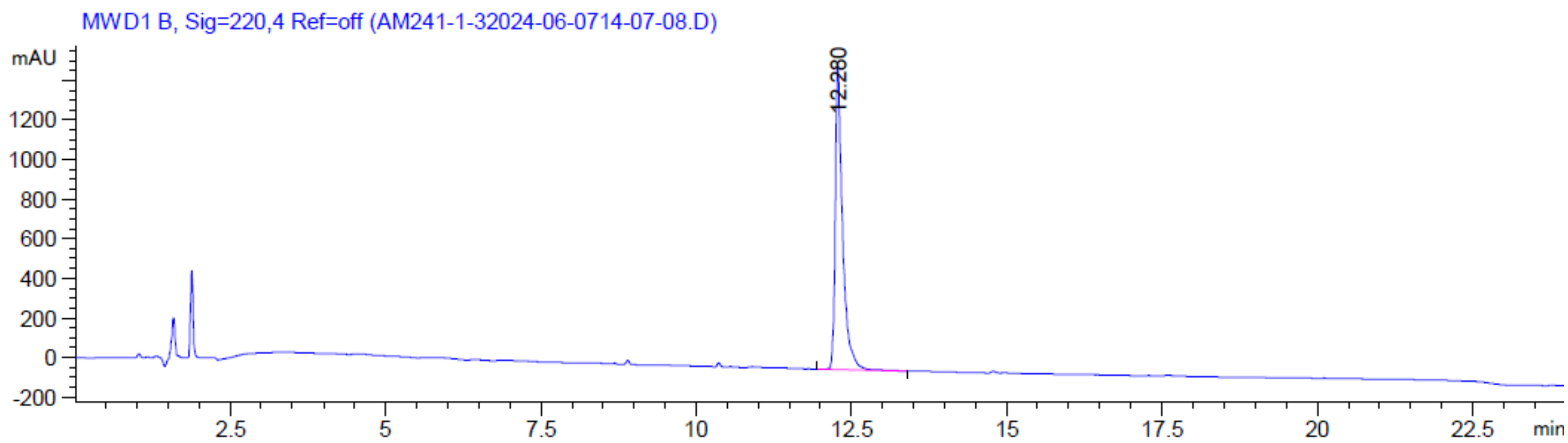

MWD1 B, Sig=220,4 Ref=off (AM241-1-32024-06-0714-07-08.D)
mAU
12.280
min

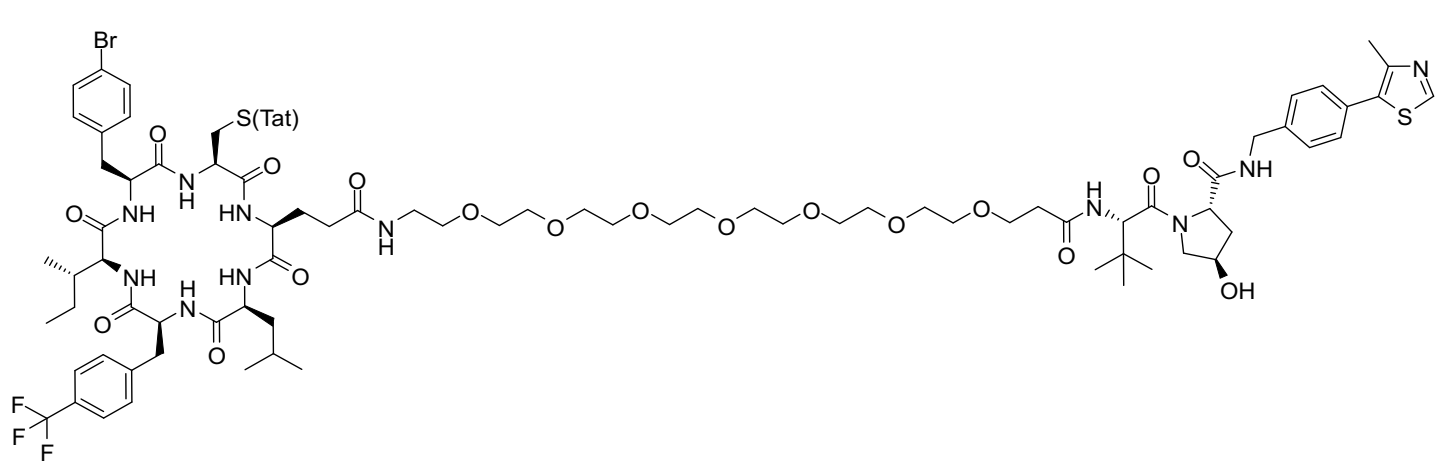

cyclo-C(Tat)LE(PEG_7)(VHL)F(CF_3)IF(Br) (**75**)

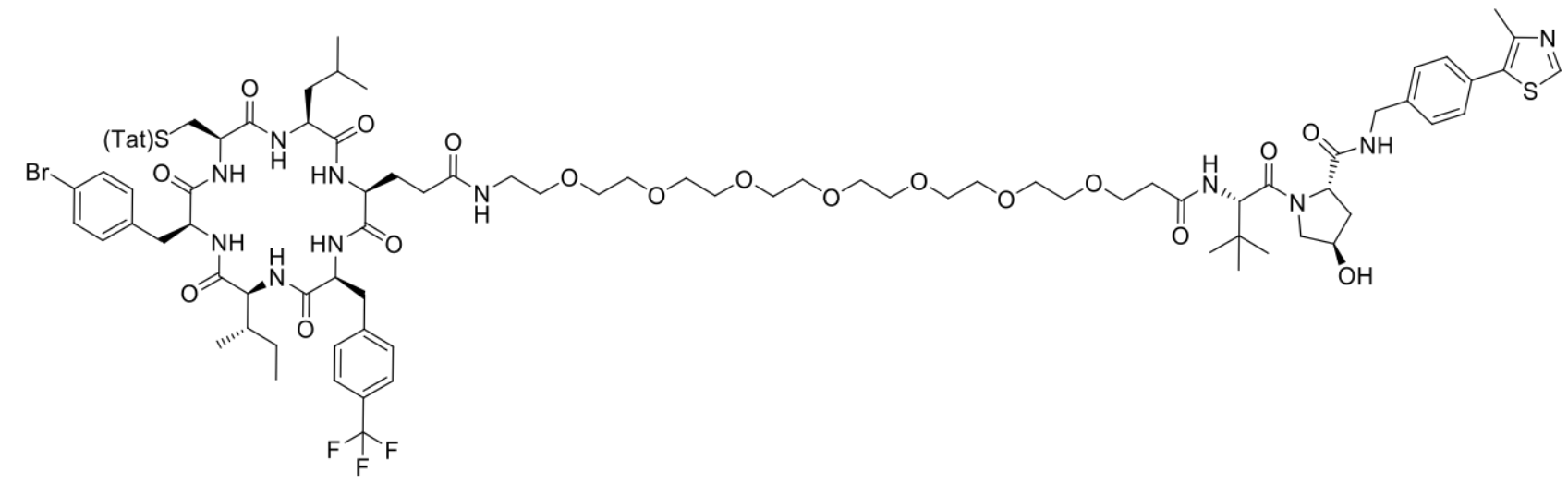

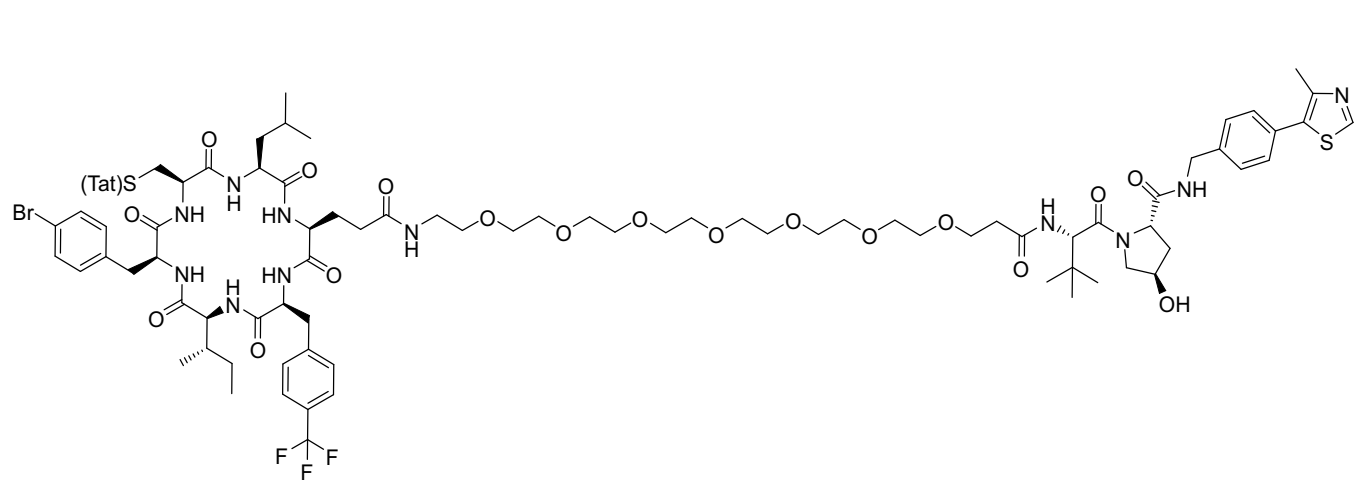

The linear peptide LC(StBu)F(Br)IF(CF_3)E was synthesised using 2-CTC resin SPPS at a 0.2 mmol scale. The linear peptide was then cyclised and subjected to TFA deprotection and concentrated *in vacuo*. The crude mixture was purified via reverse-phase flash chromatography to yield C(StBu)LEF(CF_3)IF(Br) as a white solid (53 mg, 0.053 mmol, 27%). The pure cyclic peptide (13.3 mg, 0.013 mmol) was dissolved in DMF (1 mL) to which was added DIPEA (6.6 µL, 0.039 mmol, 3 eq), HATU (6 mg, 0.016 mmol, 1.2 eq) and tert-Butyl 1 amino-3,6,9,12,15,18,21 heptaoxatetracosan-24-oate (7.1 mgs, 0.016 mmol, 1.2 eq). The reaction was then stirred at ambient temperature and reaction progress was monitored by LC-MS. Once completed, the reaction crude was concentrated *in vacuo* and subjected to TFA deprotection. The TFA reaction mixture was then concentrated *in vacuo* and purified by reverse phase chromatography to give C(StBu)LE(PEG_7)F(CF_3)IF(Br) as a white solid (6.7 mg, 0.005 mmol, 39%). C(StBu)LE(PEG_7)F(CF_3)IF(Br) (6.7 mg, 0.005 mmol) was dissolved in DMF (1 mL) to which was added DIPEA (3 µL, 0.015 mmol, 3 eq), HATU (2.3 mg, 0.006 mmol, 1.2 eq) and VH032 ligand (2.2 mg, 0.006 mmol, 1.2 eq). The reaction was then stirred at ambient temperature and reaction progress monitored by LC-MS. Once completed, the reaction crude was purified by reverse phase chromatography to give C(StBu)LE(PEG_7(VHL))F(CF_3)IF(Br) as a white solid (6.8 mg, 0.0038 mmol, 77%). The StBu group was then removed and the crude purified via reverse-phase HPLC. Fractions were analysed via LC/MS, desired fractions were then pooled, concentrated *in vacuo,* and lyophilised to yield the cyclic peptide CLE(PEG_7(VHL))F(CF_3)IF(Br) (3.7 mg, 0.002 mmol, 52%) as a white solid. *cyclo*- CLE(PEG_7(VHL))F(CF_3)IF(Br) (3.7 mg, 0.002 mmol) was added to a solution of C(Spy)GRKKRRQRRRPPQ (8.5 mg, 0.004 mmol, 2 eq) dissolved in DMF (2 mL) and stirred in an inert atmosphere overnight at ambient temperature. The mixture was then purified by reverse-phase HPLC. Fractions analysed via LC/MS, desired fractions were then pooled, concentrated *in vacuo*, and lyophilised to yield *cyclo*-C(Tat)LE(PEG_7(VHL))F(CF_3)IF(Br) as a white solid (4.6 mg, 0.0013 mmol, 66%).

LR MS (ESI+): m/z $[M+H]^+$ cald for $C_{151}H_{245}BrF_3N_{47}O_{35}S_3$, 3509.71, found; 703.4 $[M+5H]^{5+}$, 586.4 $[M+6H]^{6+}$.
HRMS (ESI+): shows expected model isotope pattern.
Analytical HPLC: Rt: (220 nm) 12.300 min (91% Purity).

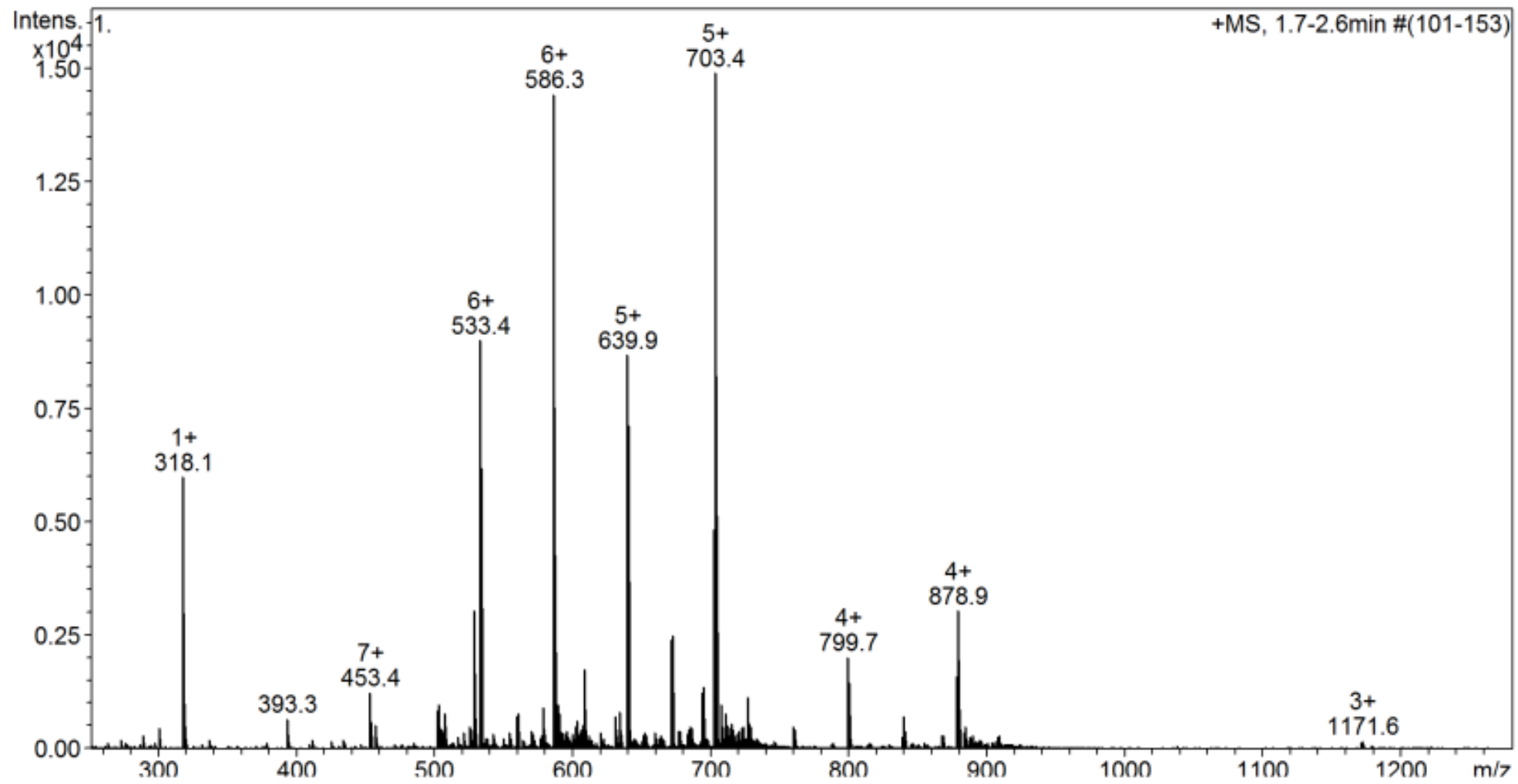

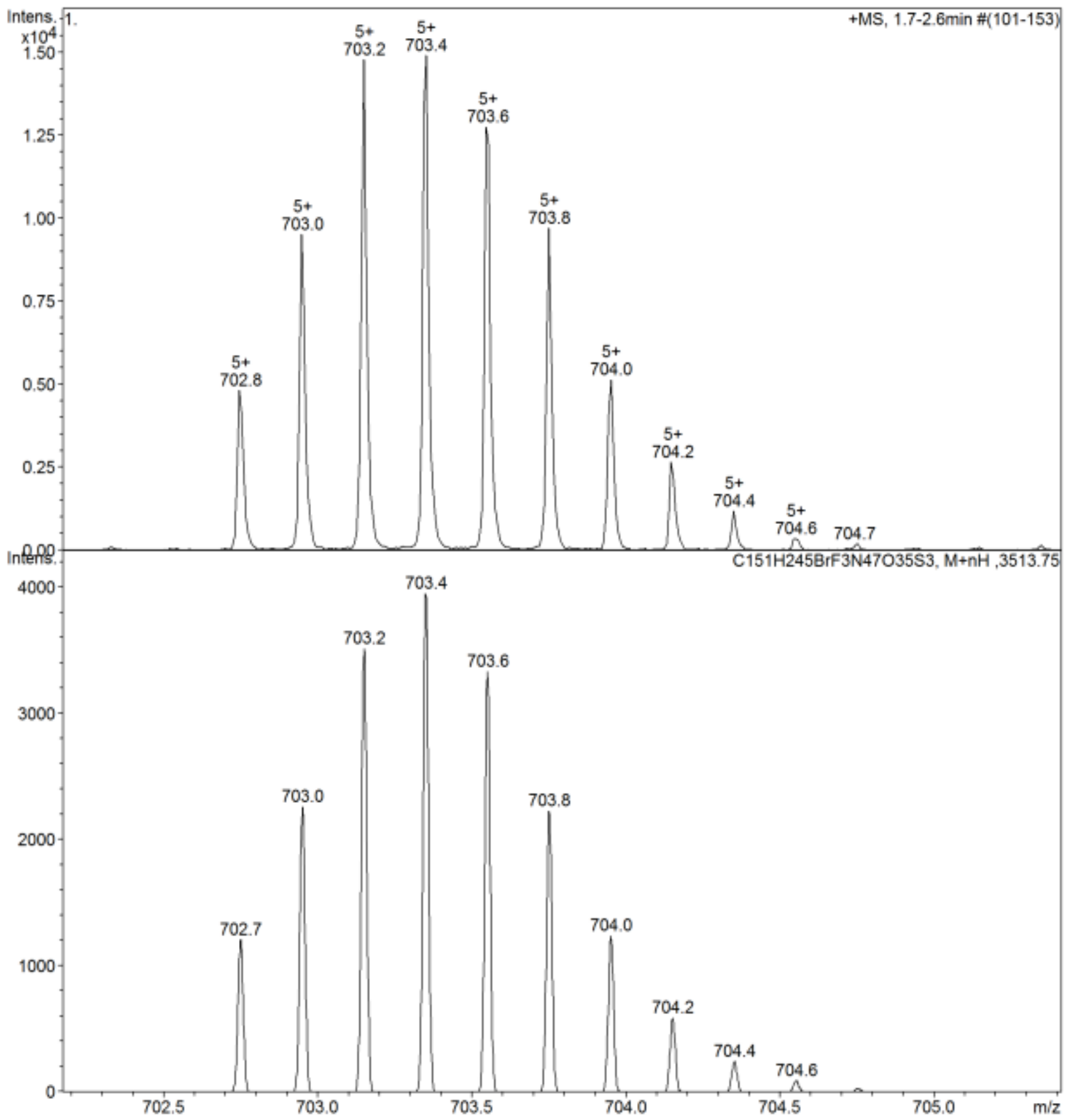

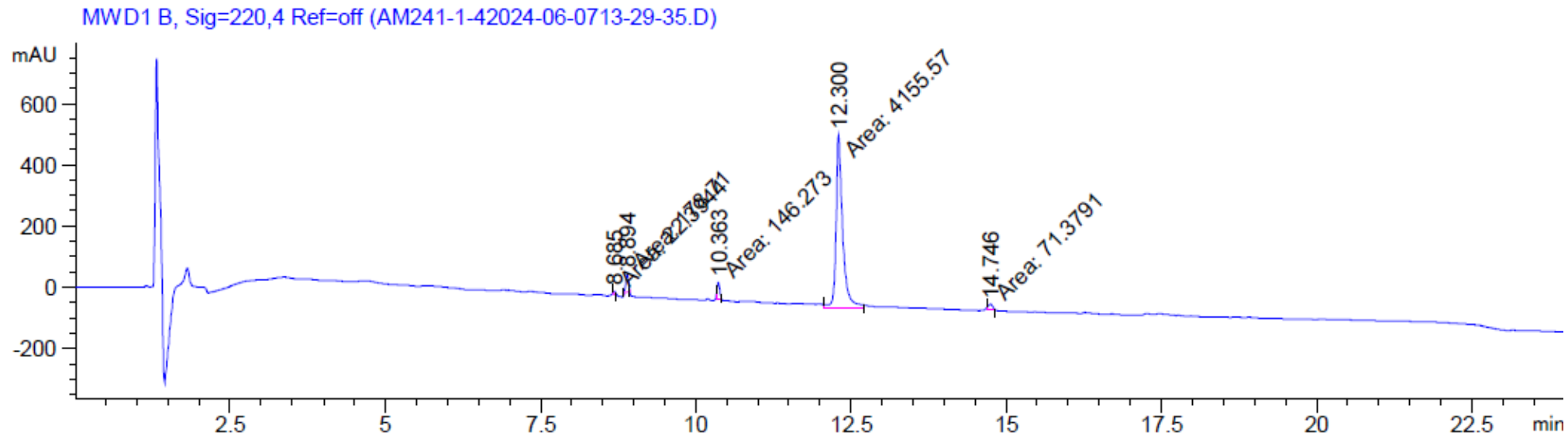
MWD1 B, Sig=220,4 Ref=off (AM241-1-42024-06-0713-29-35.D)
mAU
600
400
200
0
-200
2.5
5
7.5
10
12.5
15
17.5
20
22.5
min
12.300
Area: 4155.57
10.363
Area: 146.273
14.746
Area: 71.3791

References:
Ryan SJ, Zhang Y, Kennan AJ. Convenient access to glutamic acid side chain homologues compatible with solid phase peptide synthesis. *Org Lett*. **2005**; *7*(21): 4765-4767.

Buckley DL, Gustafson JL, Van Molle I, et al. Small-Molecule Inhibitors of the Interaction between the E3 Ligase VHL and HIF1α. *Angewandte Chemie International Edition*. **2012**; *51*(46): 11463-11467.

Voigtritter K, Ghorai S, Lipshutz BH. Rate enhanced olefin cross-metathesis reactions: The copper iodide effect. *Journal of Organic Chemistry*. **2011**; *76*(11): 4697-4702.
